# Discovery of new 1*H*-pyrazolo[3,4-*d*]pyrimidine derivatives as anticancer agents targeting EGFR^WT^ and EGFR^T790M^

**DOI:** 10.1080/14756366.2022.2112575

**Published:** 2022-08-23

**Authors:** Ahmed A. Gaber, Mohamed Sobhy, Abdallah Turky, Hanan Gaber Abdulwahab, Ahmed A. Al-Karmalawy, Mostafa. A. Elhendawy, Mohamed. M. Radwan, Eslam B. Elkaeed, Ibrahim M. Ibrahim, Heba S. A. Elzahabi, Ibrahim H. Eissa

**Affiliations:** a Department of Pharmaceutical Organic Chemistry, Faculty of Pharmacy (Boys), Al-Azhar University, Cairo, Egypt; b Department of Pharmaceutical Medicinal Chemistry and Drug Design, Faculty of Pharmacy (Girls), Al-Azhar University, Cairo, Egypt; c Department of Pharmaceutical Medicinal Chemistry, Faculty of Pharmacy, Horus University-Egypt, New Damietta, Egypt; d Department of Chemistry and Biochemistry, University of Mississippi, MS, USA; e Department of Agriculture Chemistry, Faculty of Agriculture, Damietta University, Damietta, Egypt; f National Center for Natural Products Research, University of Mississippi, University, MS, USA; g Department of Pharmacognosy, Faculty of Pharmacy, Alexandria University, Alexandria, Egypt; h Department of Pharmaceutical Sciences, College of Pharmacy, AlMaarefa University, Riyadh, Saudi Arabia; i Biophysics Department, Faculty of Science, Cairo University, Cairo, Egypt; j Pharmaceutical Medicinal Chemistry & Drug Design Department, Faculty of Pharmacy (Boys), Al-Azhar University, Cairo, Egypt

**Keywords:** Anti-proliferative, apoptosis, EGFR inhibitors, molecular docking, 1*H*-pyrazolo[3,4-*d*]pyrimidine

## Abstract

New 1*H*-pyrazolo[3,4-d]pyrimidine derivatives were designed and synthesised to act as epidermal growth factor receptor inhibitors (EGFRIs). The synthesised derivatives were assessed for their *in vitro* anti-proliferative activities against A549 and HCT-116 cancer cells. Compounds **8, 10, 12a,** and **12b** showed potent anti-proliferative activities. Compound **12b** was the most promising member with IC_50_ values of 8.21 and 19.56 µM against A549 and HCT-116, respectively. Compounds **8, 10, 12a,** and **12b** were evaluated for their kinase inhibitory activities against wild EGFR (EGFR^WT^). Compound **12b** was the most potent member showing an IC_50_ value of 0.016 µM. In addition, compound **12b** showed noticeable activity against mutant EGFR (EGFR^T790M^) (IC_50_ = 0.236 µM). Flow cytometric analyses revealed that compound **12b** is a good apoptotic inducer and can arrest the cell cycle at S and G2/M phases. Furthermore, it produced an 8.8-fold increase in BAX/Bcl-2 ratio. Molecular docking studies were carried out against EGFR^WT^ and EGFR^T790M^.

## Graphical Abstract

**Figure:**
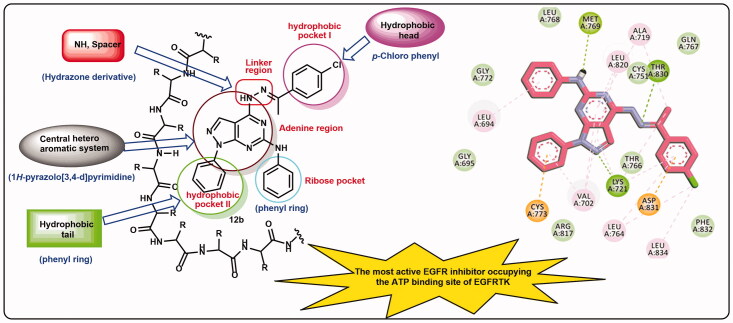

## 1. Introduction

Based on World Health Organisation International Agency for Research on Cancer (IARC), GLOBOCAN digital estimation confirmed the dramatically increased cancer incidence and mortality. The estimated value is about 19.3 million new cancer cases in 2020^1^. In 2022, 1,918,030 new cancer cases and 609,360 cancer deaths are projected to occur in the United States, including approximately 350 deaths per day from lung cancer, the leading cause of cancer death^2^. Also, cancer is a serious health issue in Africa as almost half of the cancer incidences occur in developing countries^3^. Consequently, various drug innovations against cancer were recorded, despite that, the real cause of cancer is inevitably unclear till now. Yet, cancer is mainly referred to as uncontrolled cell proliferation and finally metastasis^4^^,^^5^. The regimen of cancer treatment is greatly modified by increasing the knowledge of molecular and tumour biology^6^. Noticeably, the selectivity of anticancer approaches has a low margin^7^. So, it is a serious concern to develop a new strategy of treating cancer that provides a high selectivity margin.

Regarding molecular targeted therapy against cancer, receptor tyrosine kinases (RTKs) play a vital role in cellular programs e.g. proliferation, migration, apoptosis, survival, and differentiation^8^. The role of RTKs is the phosphorylation of tyrosine residue via transferring a gamma phosphate group from ATP to it, so normal physiological cellular functions are maintained^9–11^. The general architecture of RTKs includes extracellular ligand-binding region, transmembrane helix, and cytoplasmic region that contains protein tyrosine kinase domain decides carboxy C-terminal and juxtamembrane regulatory regions. Abnormalities can alter the regulation of RTKs that become mutated or aberrantly activated, leading to different pathological conditions such as cancer^8^.

Epidermal growth factor receptor (EGFR) is one of the most important RTKs possessing a key role in cell growth^12^^,^^13^. There are many types of tumours with a high level of EGFR overexpression as breast cancer^14^, lung cancer (NSCLC)^15^, and hepatocellular carcinoma (HCC)^16^. EGFR was found to act as a strong prognostic indicator in head and neck, ovarian, cervical, bladder, and oesophageal cancers. In these cancers, increased EGFR expression was associated with reduced recurrence-free or overall survival rates in 70% of studies^16^. EGFRs are thought to be interesting targets for developing novel anticancer drugs^17–21^.

There are many FDA-approved EGFR-tyrosine kinase inhibitors (EGFR-TKIs). The first-generation as erlotinib **I**^22^ has a good effect against wild EGFR (EGFR^WT^). This class has many side effects^23^^,^^24^ in addition to the acquired drug resistance caused by EGFR-TK mutation^25^. The second-generation was discovered to overcome the resistance induced by EGFR^T790M^. Neratinib **II**^26^ is a one of the most famous drug in this generation. Unfortunately, latter class of drugs has a low maximal-tolerated dose producing inadequate clinical efficacy^27^^,^^28^. The third-generation EGFR-TKIs as olmutinib **III** and osimertinib **IV**^29^ showed enhanced actions against mutant EGFR (EGFR^T790M^). However, toxic epidermal necrolysis was associated with these drugs^30^. Hence, there is an urgent need to optimise the approved drugs to reach efficient and less harmful candidates.

**Figure 1.**
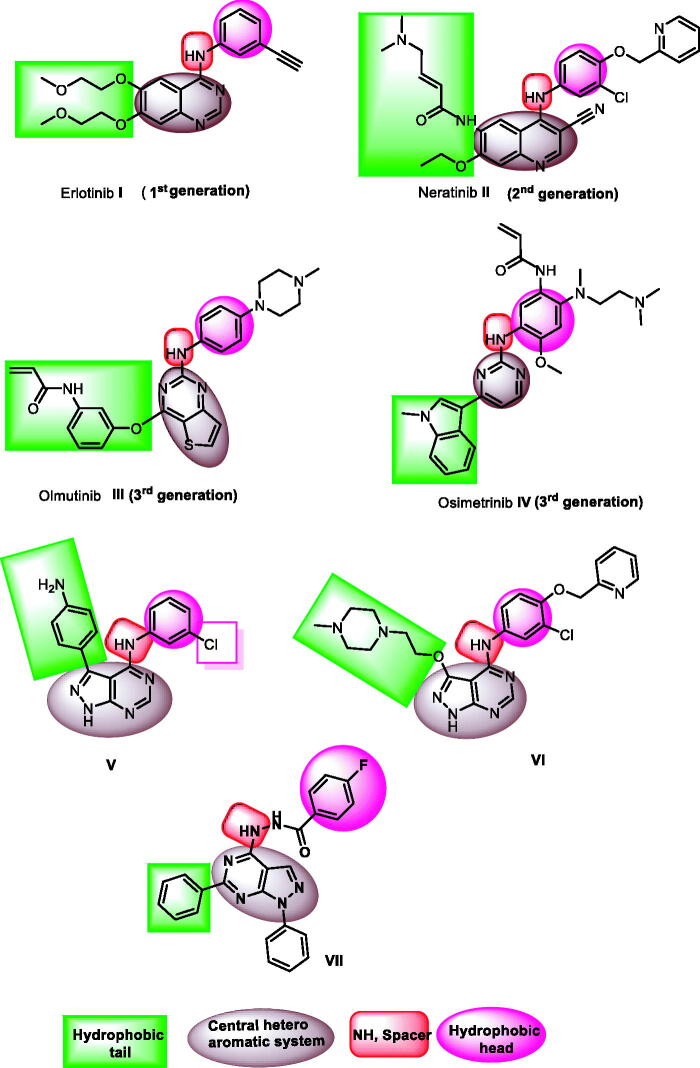
EGFR inhibitors and their pharmacophoric features.

EGFR-TKIs must possess some pharmacophoric features to bind efficiently the ATP binding site and hence exert their inhibitory activities. The first pharmacophore is the flat heteroaromatic system which can occupy the adenine binding pocket of the ATP binding site^31^. The second feature is the terminal hydrophobic head which can occupy the hydrophobic region I of the ATP binding site^32^. The third feature is the spacer moiety which is mainly an amino derivative to form a hydrogen bond in the linker region of the ATP-binding site^33^. The fourth feature is the hydrophobic tail which can occupy the hydrophobic region II of the ATP-binding site^34^^,^^35^. The fifth feature is the ribose binding moiety which can occupy the ribose binding pocket. Till now, there are limitations in research that target the ribose binding pocket^36^ (Figure 1).

1*H*-Pyrazolo[3,4-*d*]pyrimidine moiety is an important scaffold in the field of medicinal chemistry as it is a building block in many anticancer agents^36^ including EGFR-TKIs^30^. Compound **V** was approved as an ATP-competitive inhibitor showing EGFR inhibitory effect at a nanomolar concentration^37^. Compound **VI** is another example of 1*H*-pyrazolo [3,4-*d*]pyrimidine derivative with anti-EGFR activity^38^. Furthermore, our team synthesised 1*H*-pyrazolo[3,4-*d*]pyrimidine derivative (compound **VII)** as EGFR inhibitor. This compound showed good anti-proliferative activity with high inhibitory effect against wild and mutant EGFR^39^ (Figure 1). Due to the high similarity of this scaffold with the adenine moiety of ATP, it was used as a backbone for the design and synthesis of ATP competitive inhibitors, especially the compounds that target RTKs^38^^,^^40^.

Based on the previous reports including the high importance of EGFR as an anticancer target, the generated resistance against the FDA approved anticancer drugs, and the attractiveness of 1*H*-pyrazolo[3,4-*d*]pyrimidine moiety, it was decided to design and synthesise a new 1*H*-pyrazolo[3,4-*d*]pyrimidine derivatives that may have good inhibitory activities against EGFR. The synthesised compounds were designed to have the pharmacophoric features of EGFR inhibitors.

### 1.1. Rationale of molecular design

**Figure 2.**
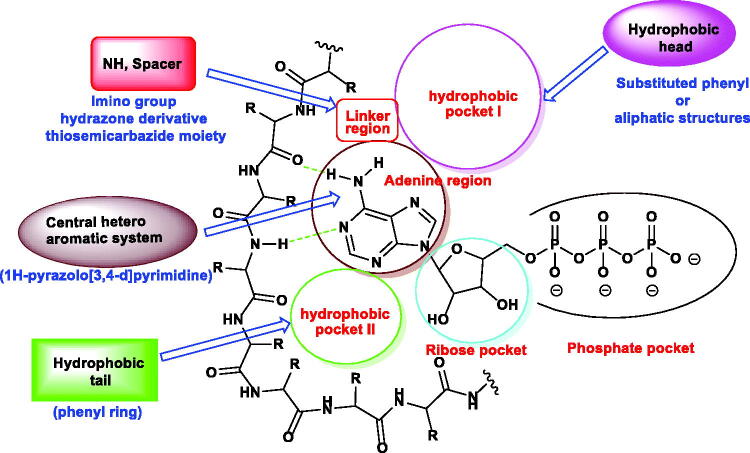
ATP binding site of EGFR-TK cavity composed of five main features^41^ and summary of the possible modifications of EGFR-TK inhibitors.

In this work, new 1*H*-pyrazolo[3,4-*d*]pyrimidine derivatives were designed and synthesised to have the main pharmacophoric features of EGFR-TKIs. In these compounds, many structural modifications for the reported EGFR-TKIs were carried out. The modification processes were achieved at five positions (Figure 2).

**Figure 3.**
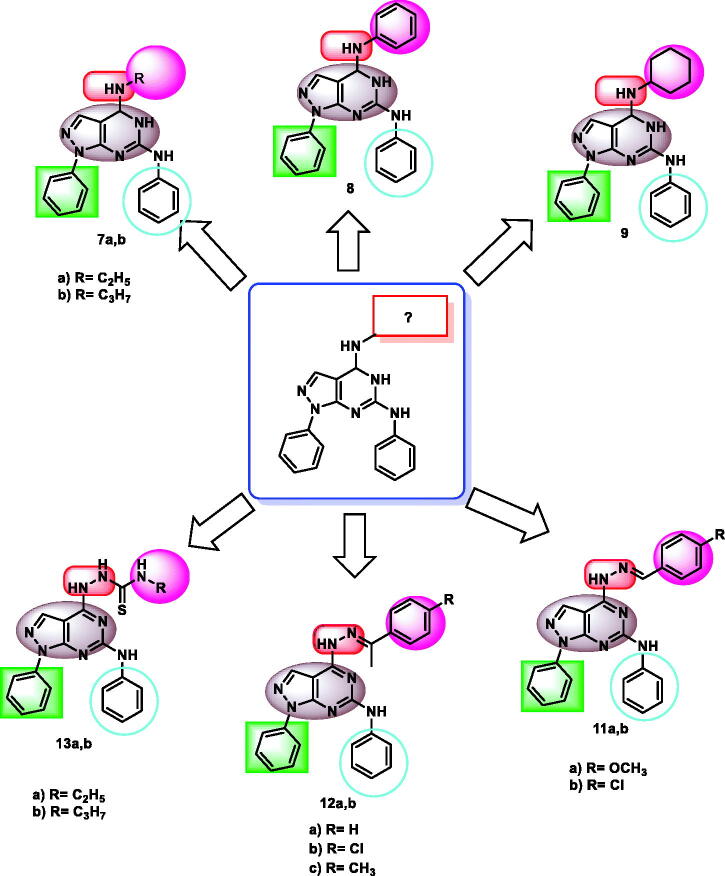
Rationale of molecular design of the new proposed EGFR-TK inhibitors.

Firstly,1*H*-pyrazolo[3,4-*d*]pyrimidine moiety was used as a heteroaromatic system to occupy the adenine binding region^42^^,^^43^. Second, different substituted phenyl or aliphatic structures were utilised as a hydrophobic head to occupy the hydrophobic region I of the ATP-binding site. The third modification was performed on the linker moiety. We used different linkers as imino group (compounds **7a,b, 8,** and **9**), hydrazone derivative (compounds **11a,b** and **12a,b**), and thiosemicarbazide moiety (compounds **13a,b**). For the hydrophobic tail, we used a phenyl ring to occupy the hydrophobic region II of the ATP-binding site. To occupy the ribose-binding pocket, we used an aniline structure. The diversity of modifications gave us good results about the structure-activity relationship of the synthesised compounds as antiproliferative agents targeting EGFR. All modifications were clarified in Figure 3.

## 2. Results and discussion

### 2.1. Chemistry

**Scheme 1.**
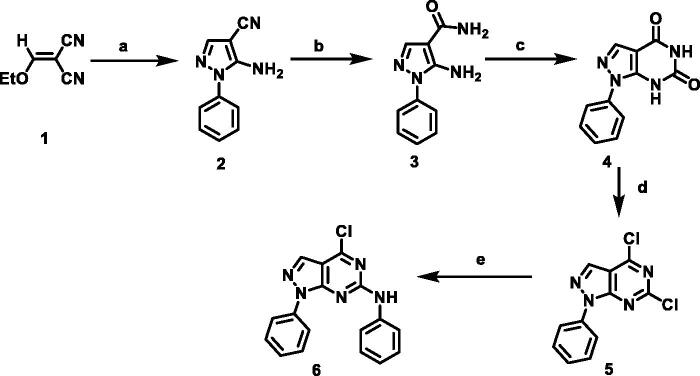
Synthetic protocol of starting compounds. **Reagents and conditions**: (a) phenyl hydrazine, absolute ethanol, reflux, 2 h; (b) sodium hydroxide, absolute ethanol, reflux, 5 h; (c) urea, fusion; (d) POCl_3_, PCl_5_, reflux, 28 h; (e) aniline, absolute ethanol, room temperature.

**Scheme 2.**
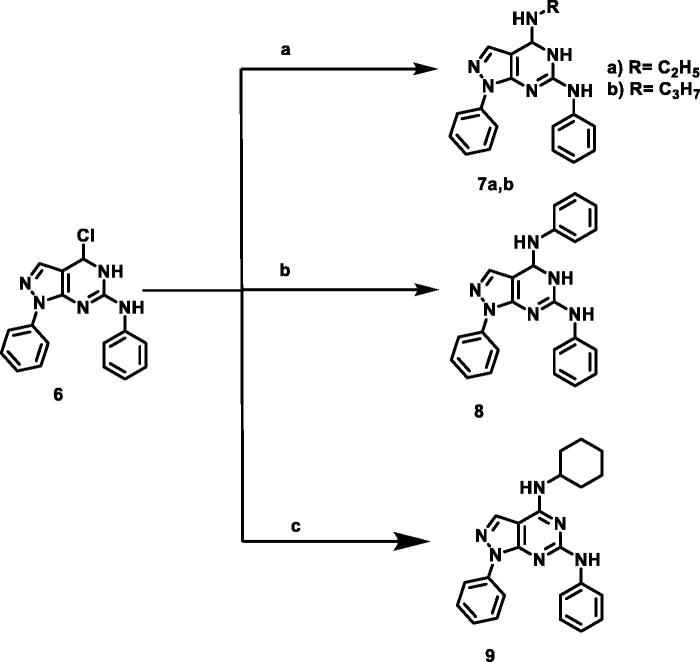
Synthetic protocol of compounds **7a,b, 8,** and **9**. **Reagents and conditions**: (a) aliphatic amines, ethanol, reflux 4 h; (b) aniline, ethanol, reflux 6 h; (c) cyclohexylamine, ethanol, reflux 5 h.

**Scheme 3.**
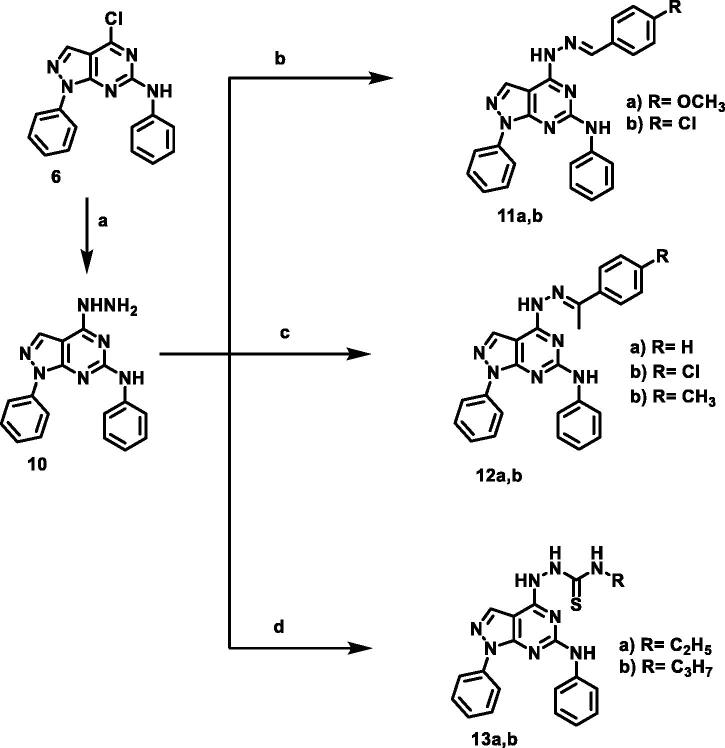
Synthetic protocol of compounds **10, 11a,b, 12a-c,** and **13a,b**. **Reagents and conditions:** (a) hydrazine hydrate 99%, reflux, 6 h; (b) aromatic aldehydes, ethanol, glacial acetic acid, reflux 12 h; (c) acetophenones, ethanol, glacial acetic acid, reflux 14 h.

The designed compounds were synthesised as outlined in Schemes 1–3. Ethoxymethylene malononitrile **1**^41^ was allowed to react with phenylhydrazine to produce 5-amino-1-phenyl-1*H*-pyrazole-4-carbonitrile **2**^44^. Compound **2** underwent partial hydrolysis using alcoholic NaOH to produce carboxamide derivative **3**^45^. Fusion of compound **3** with urea afforded 1-phenyl-1,7-dihydro-4*H*-pyrazolo[3,4-d]pyrimidine-4,6(5*H*)-dion **4.** Chlorination of compound **4** using phosphorus oxychloride and phosphorus pentachloride produced 4,6-dichloro-1-phenyl-1*H*-pyrazolo[3,4-*d*]pyrimidine **5**^46^. Stirring of compound **5** with aniline at room temperature afforded 4-chloro-*N*,1-diphenyl-4,5-dihydro-1*H*-pyrazolo[3,4-*d*]pyrimidin-6-amine **6**^47^. The obtained compound **6** was heated with commercially available different amines, namely ethylamine, propylamine, aniline, and cyclohexylamine in the presence of triethylamine afforded the target compounds **7a,b, 8,** and **9,** respectively. The IR spectra of **7a,b,** and **9** demonstrated stretching bands at a range of 2950 − 2980 cm^−1^ corresponding to CH aliphatic groups. The ^1^H NMR spectra were characterised with abroad singlet at approximately 7–8 ppm due to the additional NH group.

Compound **6** was heated with hydrazine hydrate to afford 4-hydrazinyl-*N*,1-diphenyl-1*H*-pyrazolo[3,4-*d*]pyrimidin-6-amine **10**. The IR spectrum of **10** demonstrated stretching bands at 3444, 3352, and 3190 cm-1 corresponding to NH_2_ and NH, respectively. Moreover, ^1^H NMR of this compound showed two exchangeable signals at *δ* 4.73 and 9.89 ppm corresponding to NH_2_ and NH, respectively. Refluxing of **10** with commercially available aromatic aldehydes or acetophenones in the presence of glacial acetic acid afforded the target compounds **11a,b,** and **12a-c**. ^1^H NMR spectra of hydrazones revealed the presence of D_2_O exchangeable singlet signals of hydrazinyl NH at the range 11.76–12.24 and an increase in the integration of the aromatic protons indicating the presence of an additional aromatic ring.

### 2.2. Biological evaluation

#### 2.2.1. *In vitro* antiproliferative activities

The cytotoxic activities of the synthesised compounds were assessed against two human cancer cell lines (lung, A549) and (colon HCT-116) using an MTT assay. These two cell lines were selected in this test due to the overexpression of EGFR in human lung and colon cancer cell lines^48^^,^^49^. The tested cells were reported to have a high expression level of EGFR.

As presented in Table 1, the tested compounds showed a wide range of anti-proliferative activities. This range varied from potent, moderate to weak cytotoxic effect. Comparing to erlotinib (IC_50_ = 6.77 and 19.22 µM against A549 and HCT-116, respectively), compounds **8** (IC_50_ = 16.75 and 24.16 µM), **10** (IC_50_ = 15.68 and 18.78 µM), **12a** (IC_50_ = 13.72 and 23.33 µM), and **12b** (IC_50_ = 8.21 and 19.56 µM) showed potent anti-proliferative activities. from these result, compounds **12b** is considered the most promising member.

**Table 1.** *In vitro* anti-proliferative activities of the tested compounds against human lung (A549) and colon (HCT-116) cancer cell lines.

Comp.	*In vitro* cytotoxicity IC_50_ (µM)^a^	
	A549	HCT-116
**7a**	64.42	29.62
**7b**	67.38	31.49
**8**	16.75	24.16
**9**	29.07	39.39
**10**	15.68	18.78
**11a**	47.67	46.18
**11b**	75.71	75.11
**12a**	13.72	23.33
**12b**	8.21	19.56
**12c**	28.79	28.69
**13a**	43.12	27.03
**13b**	15.36	36.44
**Erlotinib**	6.77	19.22

**^a^**Data are presented as the mean of the IC_50_ values from three different experiments.

Furthermore, compounds **7a, 7b, 12c,** and **13a** showed moderate activities against HCT-116 with IC_50_ values of 29.62, 31.49, and 27.03 µM, respectively. On the other hand, compounds **9, 11a,** and **11b** showed weak activities against the two tested cell lines while compounds **7a, 7b, 12c,** and **12a** exhibited weak activity against A549 cells.

#### 2.2.2. Structure-activity relationship

**Figure 4.**
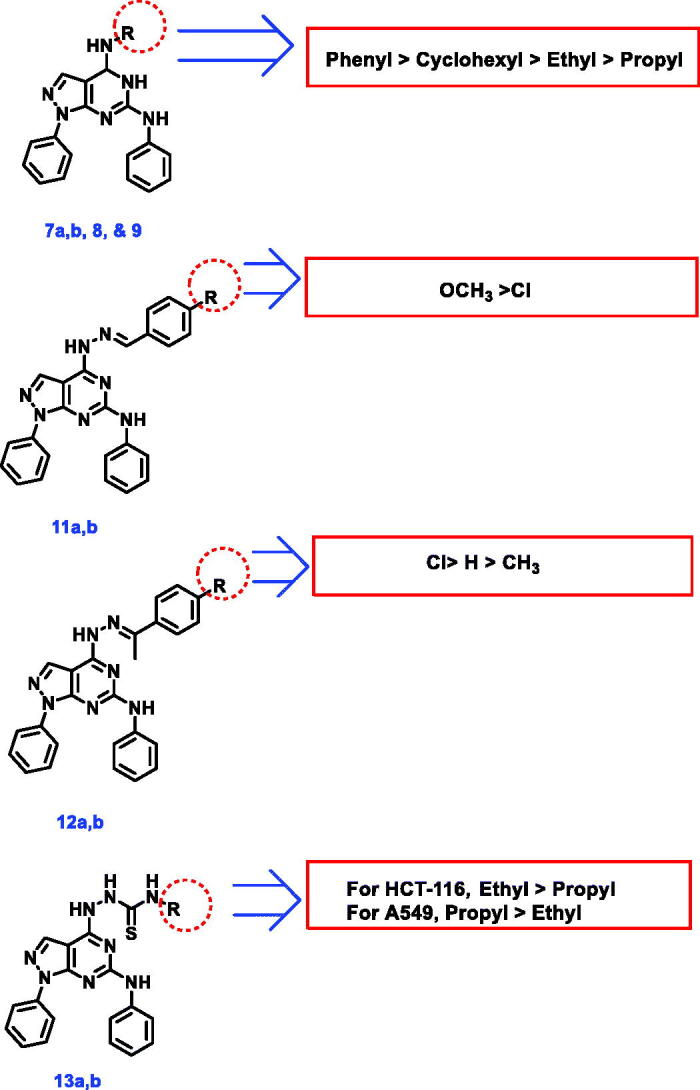
SAR study of the target compounds.

Examining screening results for cytotoxicity assay (Table 1), it was found that the introduction of aliphatic amines as ethyl (compound **7a)**, propyl (compound **7b)**, and cyclohexyl (compound **9**) in the 4-position of pyrazolo[3,4-*d*]pyrimidine scaffold was not beneficial for cytotoxic activity, particularly against A549 cells. On the contrary, the introduction of aniline moiety in the same position afforded compound **8** with enhanced anticancer activity. Additionally, a remarkable decline in cytotoxic activity against the two tested cancer cells was detected upon the condensation of hydrazine derivative **10** with aromatic aldehydes in compounds **11a,b**. Conversely, the condensation of hydrazine **10** with acetophenone and *p*-chloroacetophenone furnished compounds **12a** and **12b**, respectively with better anticancer activity against A549 cells, compared to their parent hydrazine derivative **10**. Compound **12b**, bearing p-chlorophenyl moiety, stood out as the most potent derivative among the tested compounds, presenting excellent cytotoxic activity, comparable/equipotent to that of erlotinib against A549 and HCT-116 cells, respectively. On the other hand, the *p*-tolyl derivative **12c** revealed a noticeable decrease in cytotoxic activity, relative to its parent hydrazine **10** as well as its *p*-chlorophenyl analog **12b**. Finally, in agreement with the poor cytotoxic activity elicited by aliphatic amine derivatives **7a,b**, the addition of aliphatic isothiocyanates as ethyl (compound **13a)** and propyl (compound **13b**) groups to hydrazine derivative **10**, was not beneficial for anticancer activity (Figure 4).

#### 2.2.3. EGFRWT kinase inhibitory assay

The most promising compounds in the cytotoxicity assay (**8, 10, 12a,** and **12b)** were further evaluated for their ability to inhibit the kinase activity of EGFR^WT^, employing erlotinib as a positive control. As shown in Table 2, the screened compounds significantly inhibited EGFR^WT^ at low IC_50_ values ranging from 0.016 to 0.026 µM, relative to erlotinib (IC_50_ = 0.006 µM). Compound **12b** was the most potent member showing an IC_50_ value of 0.016 µM.

**Table 2.** the inhibitory activities of the tested compounds against EGFR**^WT^** and EGFR^T790M^ kinases.

Comp.	EGFR^WT^	EGFR^T790M^
	**IC_50_ (µM)** ^a^	**IC_50_ (µM)** ^a^
**8**	0.026	NT^b^
**10**	0.023	NT^b^
**12a**	0.021	NT^b^
**12b**	0.016	0.236
**Erlotinib**	0.006	0.563

^a^Data were expressed as the mean of three independent experiments.

^b^NT: Compounds not tested.

Consistent with the results obtained from the cytotoxicity assay, it was observed that the introduction of the chlorine atom in the 4-position of phenyl ring (compound **12b**, (EGFR^WT^ IC_50_ =0.016 µM) resulted in a noticeable enhancement of EGFR^WT^ inhibitory activity, comparing to its unsubstituted analog **12a** (IC_50_ =0.021 µM).

#### 2.2.4. Egfrt790m kinase inhibitory assay

To assess the inhibitory activity of the synthesised compound against the mutant EGFR (EGFR^T790M^), the most promising member **12b** was further screened against EGFR^T790M^ utilising erlotinib as a positive control. Noticeably, it was found that compound **12b** (IC_50_ =0.236 5 µM) was 2.4-fold more potent than erlotinib (IC_50_ = 0.563 µM) against EGFR^T790M^ (Table 2).

#### 2.2.5. Cell cycle analysis

**Figure 5.**
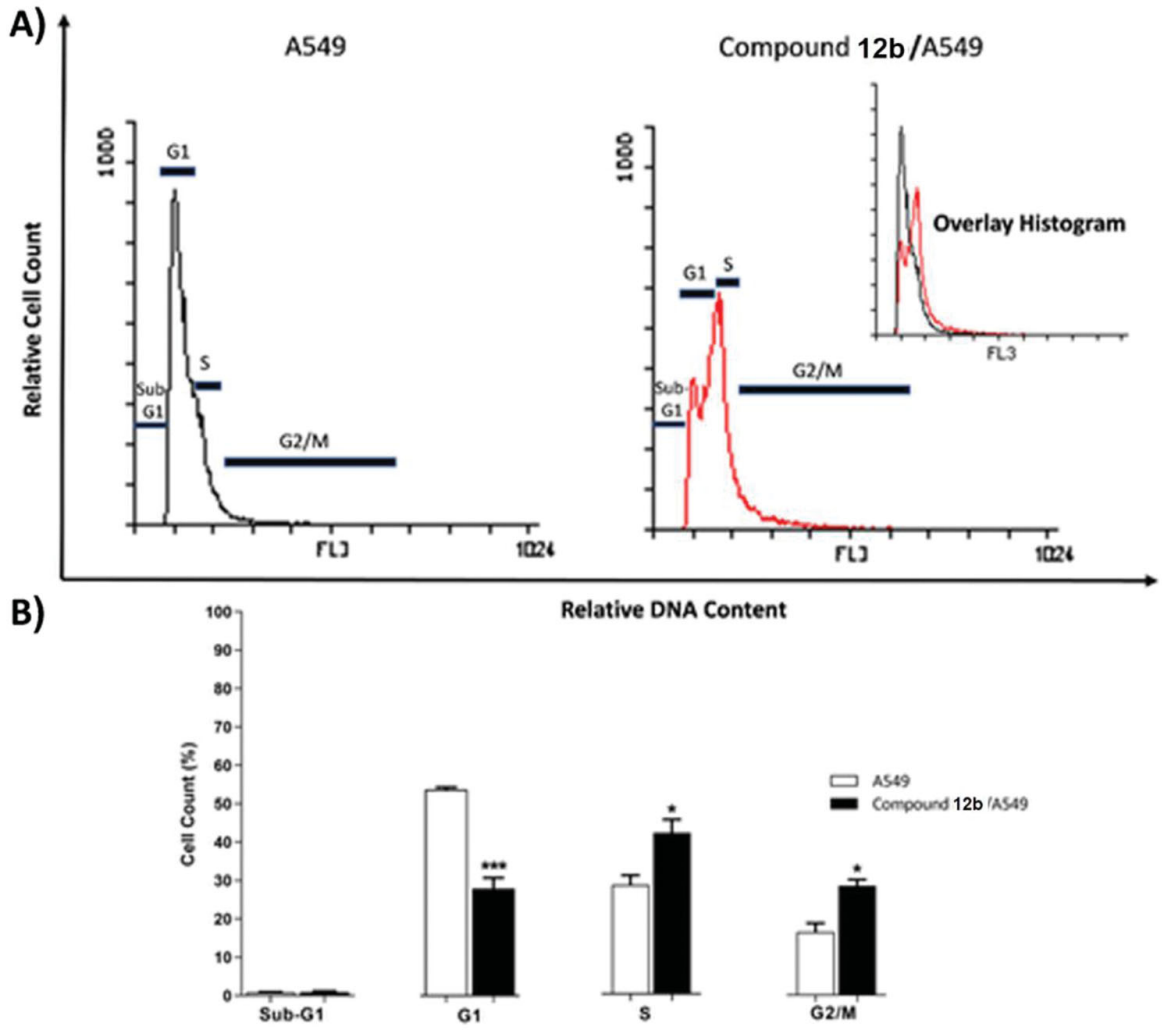
Flow cytometric analysis of cell cycle phases after treatment with compound **12 b**.

To determine the biological phase at which the synthesised compounds can interfere with the cell growth, cell cycle analysis was carried out for the most active member **12b** in A549 cells. The tested cells were treated with **12b** at a concentration of 8.21 µM equal to its IC_50_ for 48 h. The results revealed a remarkable interference with the normal cell cycle distribution. The treated cells revealed about a 2-fold decrease in the percentage of cells in the G1 phase (from 53.87 to 28.04%), compared to untreated cells. Moreover, compound **12b** induced a 1.5-fold increase in the percentage of cells in the S phase (from 28.70 to 42.39%) in addition to a 1.7-fold increase in % G2/M (from 16.56 to 28.55%). From these findings, it can be concluded that compound **12b** can arrest the cell cycle at S and G2/M phases (Table 3 and Figure 5).

**Table 3.** Effect of compound **12 b** on cell cycle progression in A549 cells after 48 h treatment.

Sample	Cell cycle distribution (%)^a^			
	%Sub-G1	%G1	%S	% G2/M
**A549**	0.87 ± 0.13	53.87 ± 0.58	28.70 ± 2.50	16.56 ± 2.30
**Compound 12 b/A549**	0.89 ± 0.31	28.04 ± 2.78***	42.39 ± 3.50*	28.55 ± 1.61*

**^a^**Values are given as mean ± SEM of three independent experiments. *p < 0.05 ***p < 0.001 indicate statistically significant differences from the corresponding control (A549) group in unpaired *t*-tests.

#### 2.2.6. Annexin V-FITC apoptosis assay

**Figure 6.**
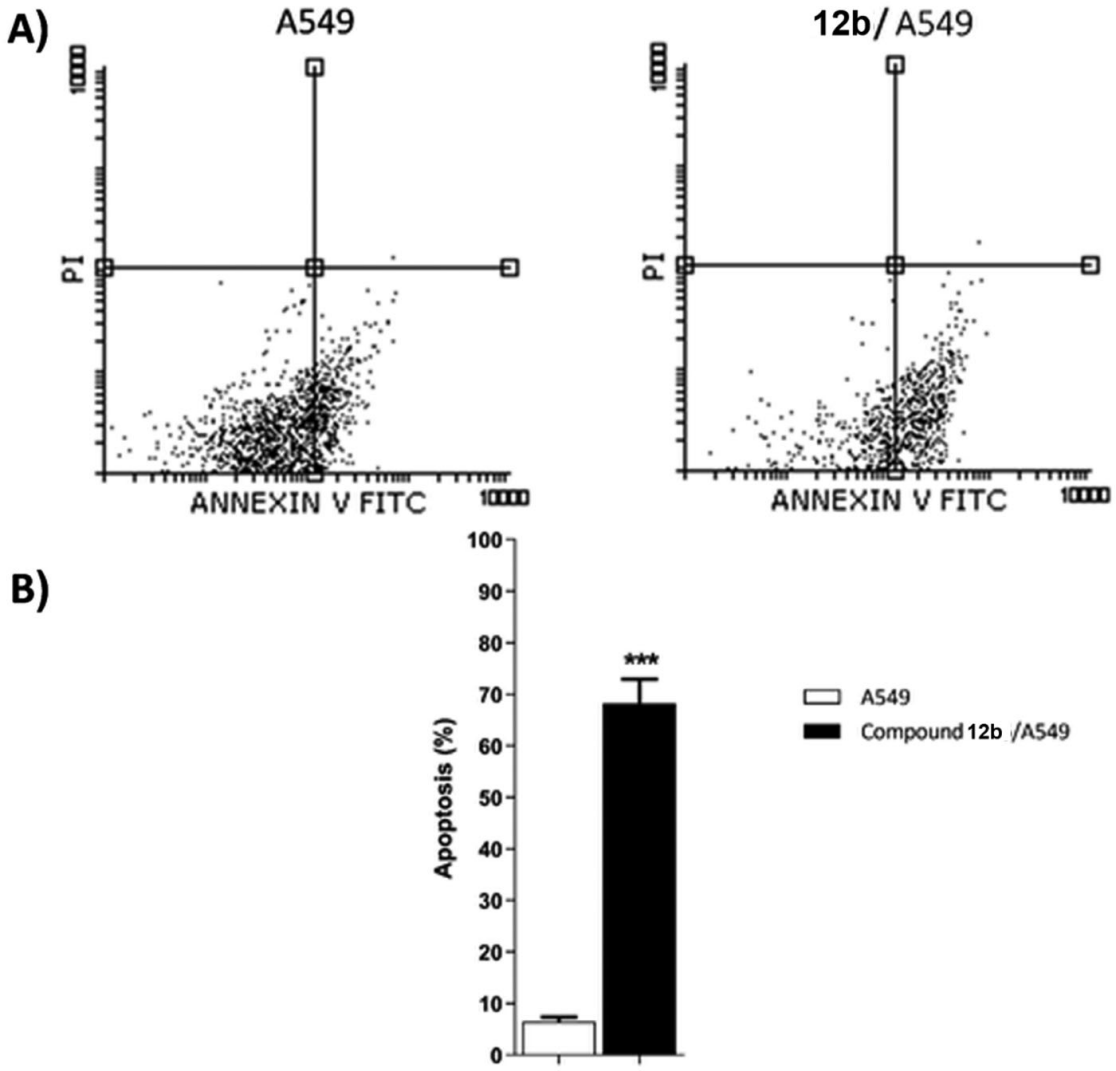
Flow cytometric analysis of apoptosis in A549 cells exposed to compound **12 b**.

As displayed in Table 4 and Figure 6, the treatment of A549 cells with compound **12b** for 48 h resulted in a 3-fold decrease in the ratio of viable cells (Left bottom) from 93.43 to 31.57%. In addition, it exhibited an 11-fold increase in the early apoptosis ratio (Right Bottom) from 6.03 to 67.69% and a 1.5-fold increase in the late apoptosis ratio (Right Top) from 0.43 to 0.64% compared to untreated A549 cells. These results indicated that compound **12b** is a good apoptotic inducer and that apoptosis is most probably the main mechanism by which compound causes cell death.

**Table 4.** Effect of compound **12 b** on stages of the cell death process in A549 cells after 48 h treatment.

Sample	Viable^a^ (Left Bottom)	Apoptosis^a^		Necrosis^a^ (Left Top)
		Early (Right Bottom)	Late (Right Top)	
A549	93.43 ± 0.96	6.03 ± 0.76	0.43 ± 0.19	0.11 ± 0.03
Comp. 12 b/ A549	31.57 ± 4.64	67.69 ± 4.86***	0.64 ± 0.30	0.10 ± 0.02

**^a^**Values are given as mean ± SEM of three independent experiments. ***p < 0.001 indicates a statistically significant difference from the corresponding control (A549) group in unpaired *t*-tests.

#### 2.2.7. Bax/bcl-2 ratio

**Figure 7.**
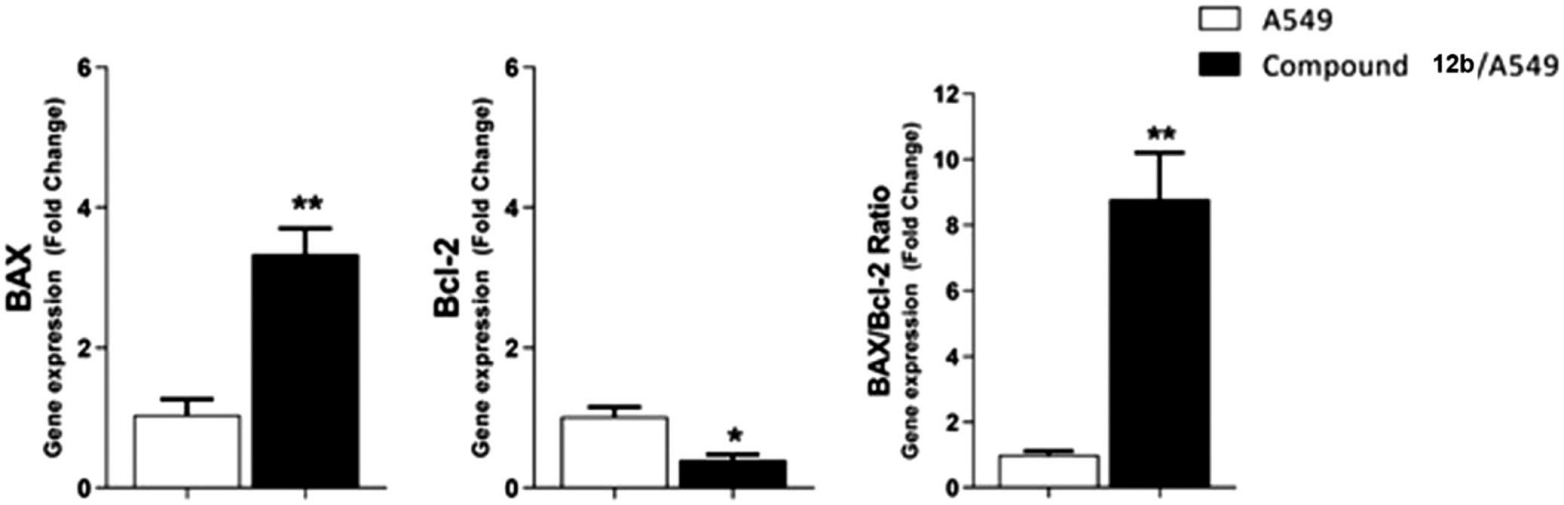
Gene expression analysis for the expression levels of BAX, and Bcl-2 after treatment of A549 with compound **12 b** for 48 h. Normalised data are expressed as the fold changes, with the control set to ‘1’. *p < 0.05 **p < 0.01 indicate statistically significant differences from the corresponding control in unpaired t-tests.

The effect of the most active compound **12b** on the expression levels of the apoptotic (BAX) and anti-apoptotic (Bcl-2) genes was evaluated. As shown in Table 5 and Figure 7, the treatment of A549 cells with compound **12b** for 48 h resulted in a 3.3-fold increase in the level of BAX gene expression in addition to a 2.5-fold decrease in Bcl-2 gene expression. As a result, an 8.8-fold increase in BAX/Bcl-2 ratio was observed for **12b**-treated A549 cells, compared to untreated A549 cells.

**Table 5.** Effect of Compound **12 b** on levels of BAX, and Bcl-2 genes expression in A549 cells treated for48 h.

Sample	Gene expression (Fold Change)^a^		
	BAX	Bcl-2	BAX/Bcl-2 ratio
**A549**	1.00 ± 0.22	1.00 ± 0.13	1.00 ± 0.21
**12b / A549**	3.33 ± 0.37**	0.40 ± 0.08*	8.80 ± 1.41**

**^a^**Values are given as changes from the corresponding control (A549) group, which is set to ‘1’. *p < 0.05 **p < 0.01 indicate statistically significant differences from the corresponding control in unpaired t-tests.

#### 2.2.8. *In vitro* cytotoxicity against normal cell and selectivity index

The *in vitro* cytotoxic effect of the most active compound **12b** against a normal cell line (WI-38) was assessed (Table 6). The results revealed that compound **12b** has low toxicity against the tested cells with IC_50_ value of 39.15 μM. Erlotinib as a reference drug showed an IC_50_ value of 33.75 µM against WI-38 cell line.

**Table 6.** *In vitro* cytotoxicity of **12 b** and erlotinib against normal cells (WI-38).

Comp.	Cytotoxicity WI-38 IC_50_ (µM)^a^	Selectivity index (SI)	
		A549^b^	HCT-116^c^
12b	39.15	4.77	2.00
Erlotinib	33.75	4.99	1.76

^a^The results were the mean of three replicates.

^b^SI = cytotoxicity against WI-38 cells/cytotoxicity against A549 cell line.

^c^SI = cytotoxicity against WI-38 cells/cytotoxicity against HCT-116 cell line.

**Figure 8.**
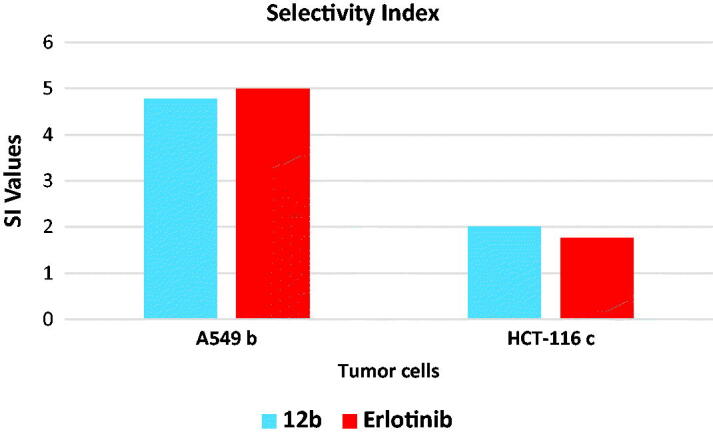
Selectivity indices of compound **12 b**.

The selectivity index (SI) of compound **12b** against tumour cells were shown in Figure 8. This compound showed a SI of 4.77 and 2.00 against A549 and HCT-116, respectively. These indices are comparable to that of erlotinib **(**4.99 and 1.76) against A549 and HCT-116, respectively.

The results revealed that compound **12b** has low toxicity against WI-38 cell line compared to erlotinib. In addition, it showed a high selectivity against the tumour cell lines.

### 2.3. *In silico* studies

#### 2.3.1. Docking studies

To investigate the manner of binding with the hypothesised target, docking studies were performed for the synthesised compounds against the active site of the wild-type (EGFR^WT^, PDB: 4HJO)^50^ and the mutant type (EGFR^T790M^, PDB: 3W2O)^51^. The co-crystallised ligands erlotinib and TAK-285 of EGFR^WT^ and EGFR^T790M^, respectively, were used as reference compounds. The docked compounds showed good binding affinities towards EGFR^WT^, with binding free energies ranging from −19.63 to −23.67 kcal/mol, according to the results of docking studies. For the docking against mutant type, the synthesised compounds showed binding energy ranging from −16.09 to −21.66 (Table 7).

**Table 7.** The docking binding free energies of the synthesised compounds against EGFR^WT^ and EGFR^T790M^

Comp.	Binding free energy (kcal/mol)	
	EGFR^WT^	EGFR^T790M^
7a	−19.63	−16.09
7b	−20.55	−17.79
9	−21.00	−19.01
8	−21.80	−19.60
10	−17.06	−15.83
11a	−21.58	−20.10
11b	−21.67	−20.19
12a	−23.07	−21.14
12b	−23.09	−20.59
12c	−23.67	−21.66
13a	−19.69	−20.30
13b	−21.74	−20.77
**Erlotinib**	−22.59	–
**TAK-285**	–	−21.49

**Figure 9.**
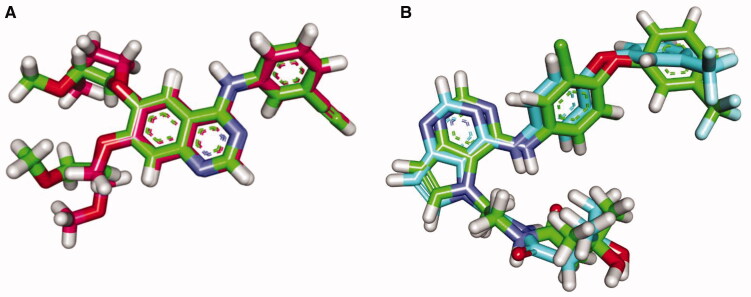
**(A**) Superimposition of the re-docked conformer of erlotinib (pink) over the co-crystallised conformer (green) with an RMSD value of 1.18 Å. (**B**) Superimposition of the re-docked conformer of TAK-285 (pink) over the co-crystallised conformer (green) with an RMSD value of 1.66 Å.

In these investigations, MOE 2019 software was used. The output figures were further visualised using Discovery Studio software 3.0. The docking mechanisms were initially validated by redocking each protein's co-crystallised ligands (Erlotinib and TAK-285) against the active sites of EGFR^WT^ and EGFR^T790M^, respectively. The generated RMSD values between the re-docked conformers and the co-crystallised conformers were 1.18 and 1.66 Å for erlotinib and TAK-285, respectively. As reported, an RMSD value of less than 2 Å suggests that the docking operation is genuine. As a result, the obtained RMSD values confirmed the docking protocol's validity (Figure 9).

**Figure 10.**
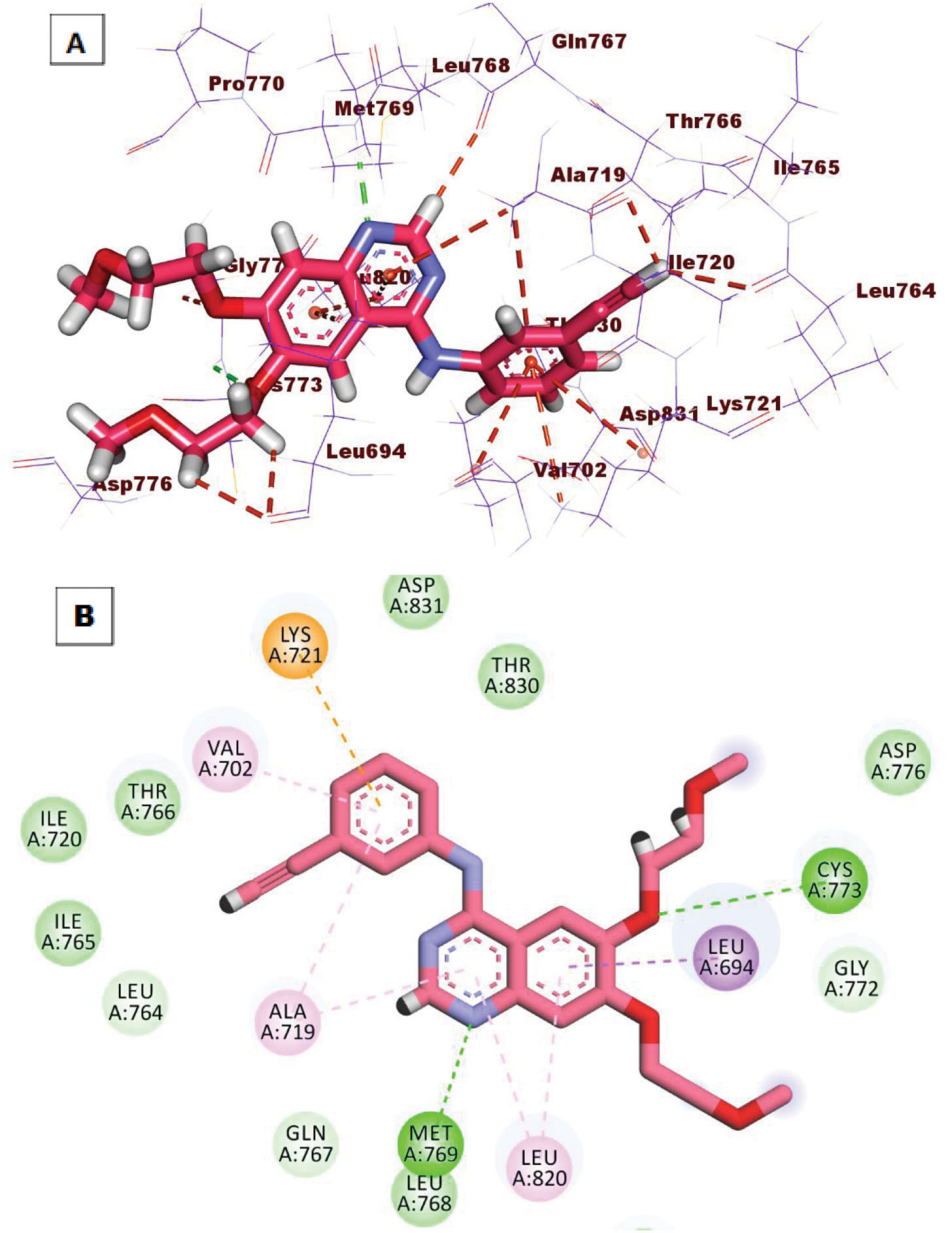
**(A**) 3D interaction of erlotinib docked into the active site of EGFR^WT^. The hydrogen bonds were represented in green dashed lines. The pi interactions were represented in orange lines. (**B**) 2D interaction of erlotinib docked into the active site of EGFR^WT^.

The co-crystallised ligand (erlotinib) of EGFR^WT^ produced a binding score of −22.59 kcal/mol. The binding mode of erlotinib against EGFR^WT^ was shown in Figure 10. The heterocyclic quinazoline moiety was oriented into the adenine pocket forming a hydrogen bond with Met769. In addition, it formed four hydrophobic interactions with Lue694, Ala719, and Leu820. The ethynylphenyl moiety was oriented into the hydrophobic pocket I forming three hydrophobic interactions with Ala719, Lys721, and Val702. The two 2-methoxyethoxy groups occupied the hydrophobic region II forming a hydrogen bond with Cys773 in close contact with Gly772 and Leu694.

**Figure 11.**
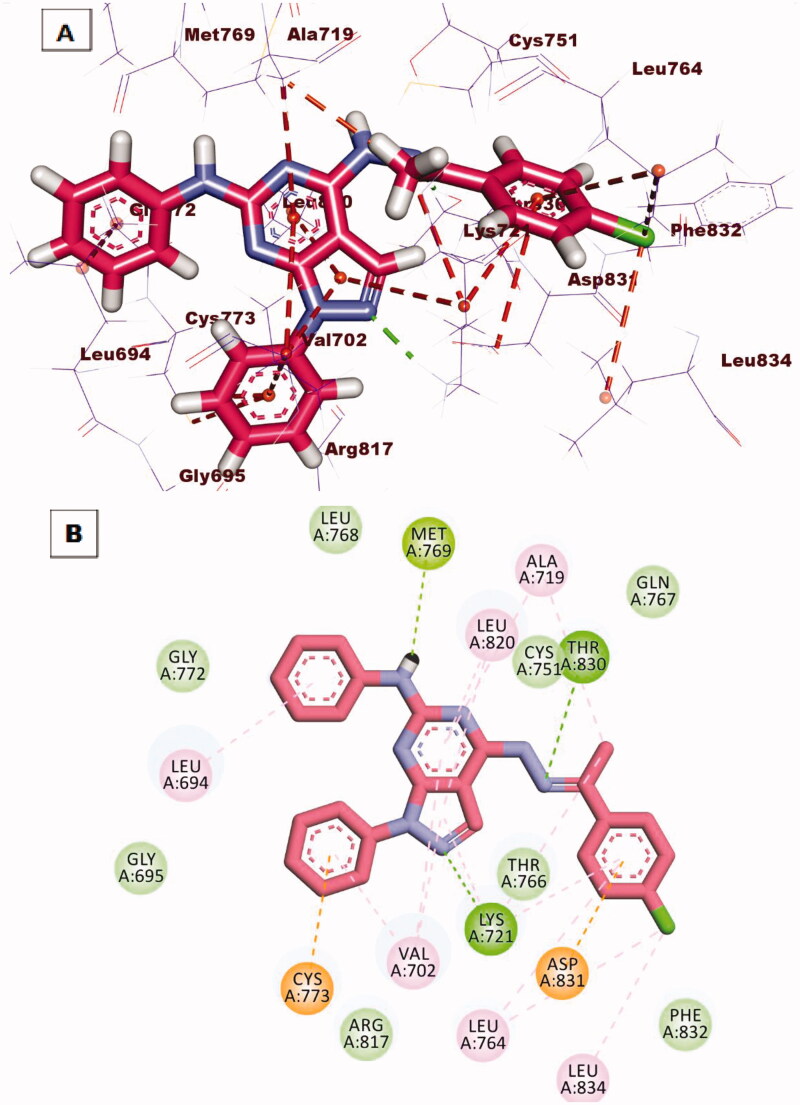
(**A)** 3 D interaction of compound **12 b** docked into the active site of EGFR^WT^. The hydrogen bonds were represented in green dashed lines. The pi interactions were represented in orange lines. (**B)** 2 D interaction of compound **12 b** docked into the active site of EGFR^WT^.

Taking compound **12b** as a representative example, it showed a similar binding pattern to erlotinib. Compound **12b** exhibited a binding score of −23.07 kcal/mol. The 1*H*-pyrazolo[3,4-d]pyrimidin-6-amine moiety occupied the adenine pocket to form two hydrogen bonds with Met769 and Lys721. In addition, it formed four hydrophobic interactions with Val702, Ala719, and Leu820. The *p*-chlorophenyl moiety occupied the hydrophobic pocket I forming four hydrophobic interactions with Lys721, Leu764, and Leu834. In addition, it formed an electrostatic attraction with Asp831. Th hydrazinyl linker formed one hydrogen bond with2 Thr830. The phenyl ring at 1-position of 1*H*-pyrazolo[3,4-d]pyrimidine occupied the hydrophobic region II forming two hydrophobic interactions with Val702 and Cys773 (Figure 11).

Docking of the synthesised compounds against the mutant EGFR(EGFR^T790M^) gave a good insight into its binding pattern. The synthesised compounds showed binding scores ranging from −16.09 to −21.66 kcal/mol (Table 7).

**Figure 12.**
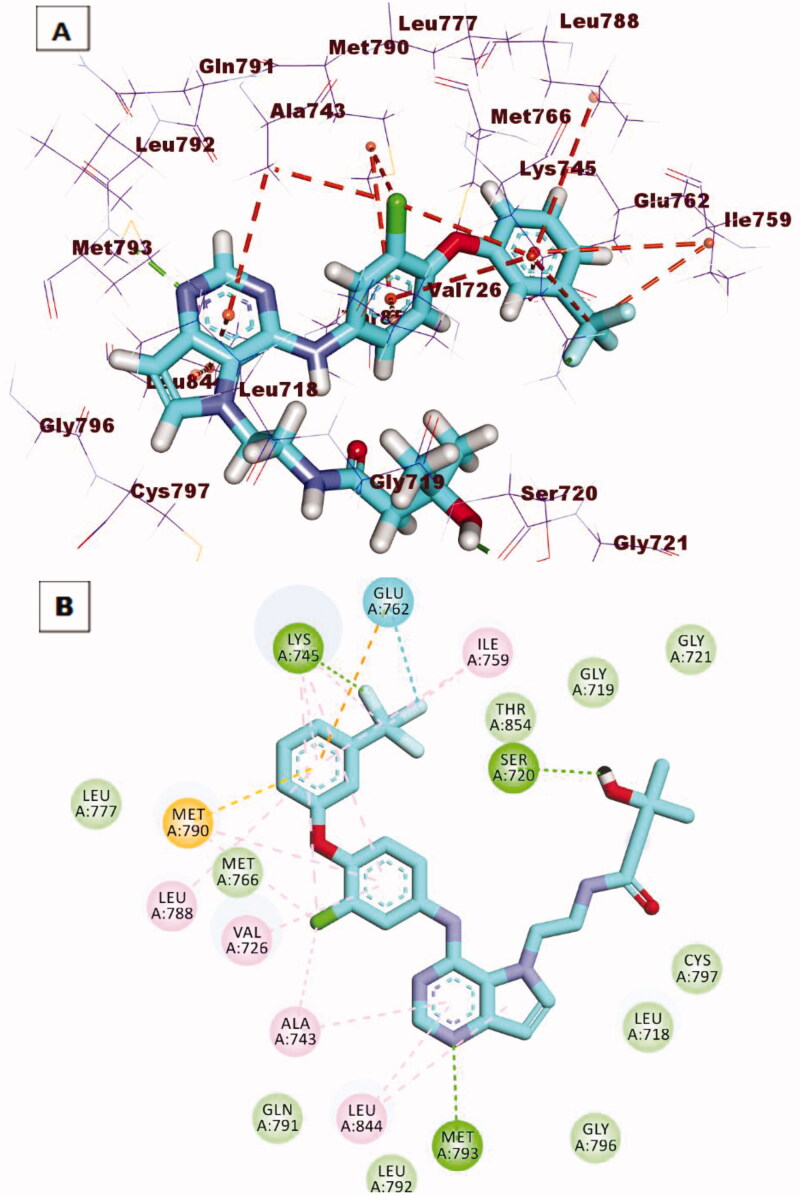
(**A)** 3 D interaction of TAK-285 docked into the active site of EGFR^T790M^. The hydrogen bonds were represented in green dashed lines. The pi interactions were represented in orange lines. (**B)** 2 D interaction of TAK-285docked into the active site of EGFR^T790M^.

2The reference compound (TAK-285) produced a binding score of −21.49 kcal/mol. The heterocyclic 5*H*-pyrrolo[3,2-d]pyrimidine moiety(the main neuclus) was oriented into the adenine pocket to form one hydrogen bond with Met793. In addition, it formed many hydrophobic interactions with Leu844 and Ala743. The 1-chloro-2–(3-(trifluoromethyl)phenoxy)benzene moiety occupied the hydrophobic pocket I to form many hydrophobic interactions with Lys745, Ile759. Lys745, Glu762, Met766, Met790, Val726, Leu788, and Ala743. Additionally, it formed two hydrogen bonds with Lys745 and Glu762. The *N*-ethyl-3-hydroxy-3-methylbutanamide moiety was buried in the hydrophobic region II to form one hydrogen bond with Ser720 (Figure 12).

**Figure 13.**
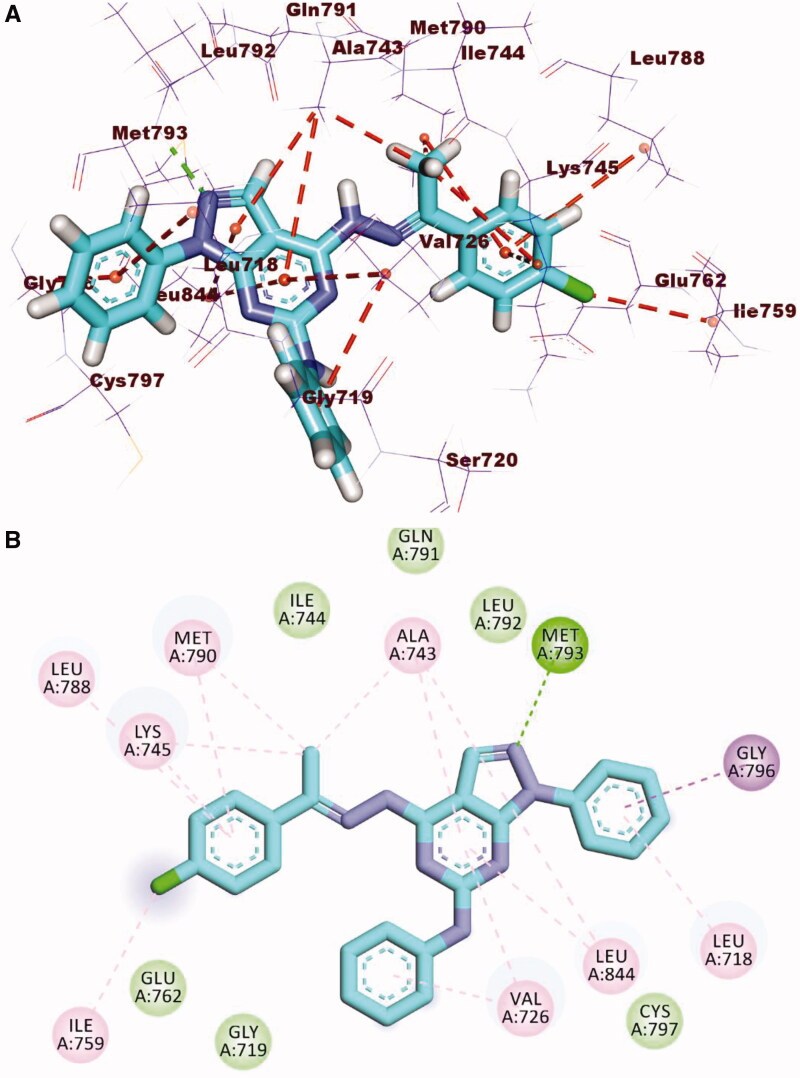
(**A)** 3 D interaction of compound **12 _b_** docked into the active site of EGFR^T790M^. The hydrogen bonds were represented in green dashed lines. The pi interactions were represented in orange lines. (**B)** 2 D interaction of compound **12 _b_** docked into the active site of EGFR^T790M^.

Compound **12b**, as a representative example, showed a binding mode like that of TAK-285 against the mutant EGFR with a binding score of −20.59 kcal/mol. The 1*H*-pyrazolo[3,4-d]pyrimidin-6-amine moiety occupied the adenine pocket to form one hydrogen bond with Met793. Also, it formed five hydrophobic interactions with Leu718, Leu844, Val726, and Ala743. The *p*-chlorophenyl moiety occupied the hydrophobic pocket I forming seven hydrophobic interactions with Ala743, Met790, Leu788, Lys745, and Ile759. The phenyl ring at 1-position of 1*H*-pyrazolo[3,4-d]pyrimidine occupied the hydrophobic region II forming two hydrophobic interactions with Gly796 and Leu718 (Figure 13).

#### 2.3.2. *In silico* ADMET analysis

Discovery studio 4.0 was used to predict ADMET descriptors for all compounds. The predicted descriptors are listed in Table 8. Blood-Brain Barrier (BBB) penetration studies predicted that compounds **8, 11a, 11b, 12a, 12b, 12c, 13a,** and **13b** have very low BBB penetration levels. Accordingly, such compounds were expected to be safe for CNS. All the tested compounds showed low to very low range levels of ADMET aqueous solubility and have good to moderate intestinal absorption levels. Additionally, all compounds were predicted to be cytochrome P450 2D6 non-inhibitors. Consequently, the liver dysfunction side effect maybe not expected upon administration of these compounds. Due to the high planarity of the synthesised compounds, all of them are expected to bind plasma protein over 90% (Table 8 & Supplementary data).

**Table 8.** Predicted ADMET for the designed compounds and reference drugs

Comp.	BBB level^a^	Solubility level^b^	Absorption level^c^	CYP2D6 prediction^d^	PPB prediction^e^
**7a**	2	2	0	false	false
**7b**	2	2	0	false	true
**9**	1	2	0	false	true
**8**	4	1	1	false	true
**10**	3	2	0	false	true
**11a**	4	1	1	false	true
**11b**	4	1	2	false	true
**12a**	4	1	1	false	true
**12b**	4	1	2	false	true
**12c**	4	1	2	false	true
**13a**	4	2	0	false	true
**13b**	4	1	1	false	true
**Erlotinib**	1	2	0	false	true

^a^BBB level, blood brain barrier level, 0 = very high, 1 = high, 2 = medium, 3 = low, 4 = very low.

^b^Solubility level, 1 = very low, 2 = low, 3 = good, 4 = optimal.

^c^Absorption level, 0 = good, 1 = moderate, 2 = poor, 3 = very poor.

^d^CYP2D6, cytochrome P2D6, TRUE = inhibitor, FALSE = non inhibitor.

^e^PBB, plasma protein binding, FALSE means less than 90%, TRUE means more than 90%.

#### 2.3.3. *In silico* toxicity studies

In this work, six toxicity parameters were estimated computationally depending on the constructed toxicity models in Discovery studio software. The results of *in silico* toxicity studies were depicted in Table 9,

**Table 9.** *In silico* toxicity studies of the synthesised compounds and erlotinib

Comp.	Ames mutagenicity	Developmental Toxicity Potential	Carcinogenic Potency TD_50_ (Rat)^a^	Rat Maximum Tolerated Dose (Feed)^b^	Ocular Irritancy	Skin Irritancy
7a	Non-Mutagen	Non-Toxic	34.965	0.287	Mild	None
7b	Non-Mutagen	Non-Toxic	27.147	0.401	Mild	None
9	Non-Mutagen	Non-Toxic	4.054	0.266	Mild	None
8	Non-Mutagen	Non-Toxic	1.528	0.213	Mild	None
10	Non-Mutagen	Non-Toxic	18.673	0.291	Mild	None
11a	Non-Mutagen	Non-Toxic	1.655	0.152	Mild	None
11b	Non-Mutagen	Non-Toxic	2.675	0.391	Mild	None
12a	Non-Mutagen	Non-Toxic	3.791	0.294	Mild	None
12b	Non-Mutagen	Non-Toxic	2.321	0.358	Mild	None
12c	Non-Mutagen	Non-Toxic	2.98	0.139	Mild	None
13a	Non-Mutagen	Non-Toxic	32.038	0.530	Mild	None
13b	Non-Mutagen	Non-Toxic	24.690	0.735	Mild	None
**Erlotinib**	Non-Mutagen	Non-Toxic	8.057	0.083	Mild	None

^a^Unit: mg/kg body weight/day.

^b^Unit: g/kg body weight.

In general, most of the synthesised compounds showed decreased toxicity potential. In detail, all compounds were predicted to be non-mutagenic and non-toxic against Ames mutagenicity and developmental toxicity potential models. In addition, all compounds were anticipated to be non-irritant and mild irritant against Skin Irritancy and Ocular Irritancy models, respectively. For, compounds **7a, 7b, 10, 13a, and 13b** showed carcinogenic potency TD_50_ values ranging from 18.673 to 34.965 mg/kg body weight/day, which were higher than that of erlotinib (8.057 mg/kg body weight/day). the other compounds showed less carcinogenic potency TD_50_ values. In addition, the tested compounds showed rat maximum tolerated dose values ranging from 0.139 to 0.735 g/kg body weight. This range is higher than the rat's maximum tolerated dose value of erlotinib (0.083 g/kg body weight).

#### 2.3.4. Molecular dynamic simulations

To study the stability and the binding strength of the protein-compound **12b** complex, GROMACS 2021 was used to run a 100 ns classical molecular dynamics simulation, and the trajectory was analysed using VMD. RMSD for the protein alone, compound **12b** alone, and the complex (Figure 14(A)), RMSF (Figure 14(B)), SASA (Figure 14(C)), RoG (Figure 14(D)), and the change in the hydrogen bonds for the protein in the protein-compound **12b** complex (Figure 14(E)) were calculated. The distance between the centre of mass of protein and the centre of mass of compound **12b** (Figure 14(F)) was measured throughout the trajectory.

Figure 14.The analyses performed on the trajectory using VMD. (**A**) The RMSD values of the protein only, compound **12 b** only, and protein-compound **12 b** complex during the trajectory. (**B**) The RMSF of the amino acids along the whole trajectory. (**C**) The SASA values of the protein. (**D**) The radius of gyration of the protein. (**E**) The change in the numbers of the hydrogen bonds between amino acids of the protein. (**F**) The change in the distance from the centre of mass of compound **12 b** and the protein.

**Figure:**
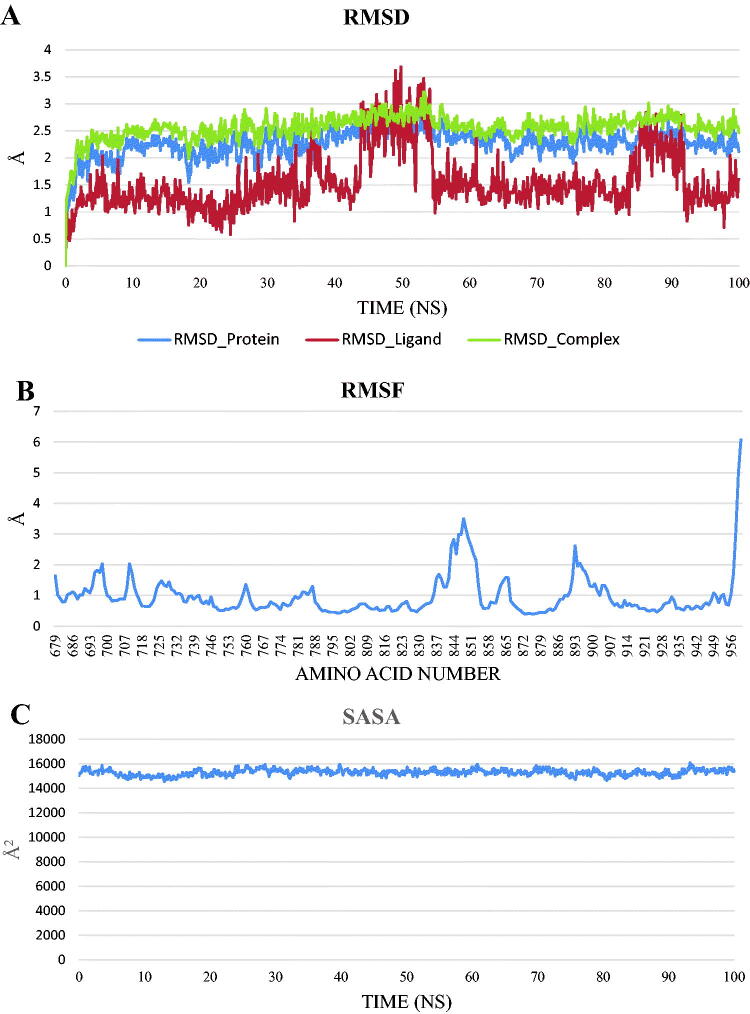

**Figure:**
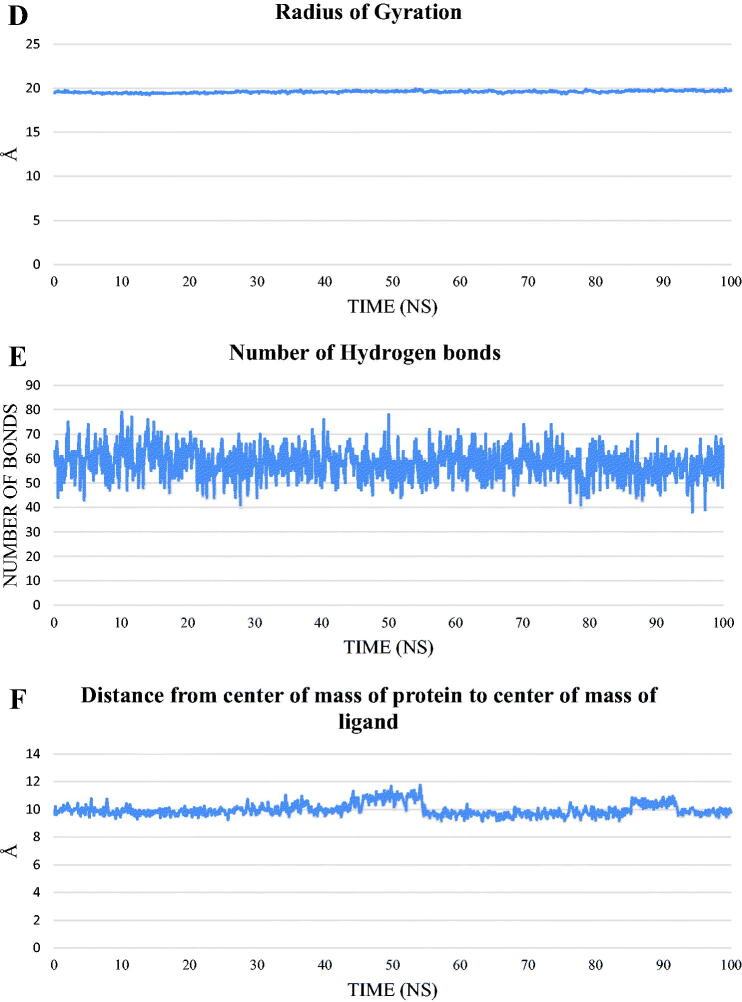

RMSD values show that the system was stable throughout the trajectory with no drastic fluctuation and an average of 2.26 Å for the protein alone. For compound **12b** alone, the RMSD showed a stable trend in almost all the trajectory with two exceptions. The duration from 44.5 ns to 54.6 ns and from 86 ns to 91.6 ns show RMSD of values larger than 2 Å. The RMSD of the complex showed a similar trend to the RMSD of the protein only with slightly larger values. In addition, the amino acids fluctuation depicted in the RMSF values showed that most of the amino acids have fluctuations of less than 2 Å except for the C-terminal (around 6 Å) and the loop from E842:P853 reaching a maximum of 3.5 Å. The SASA (average = 15301 Å^2^), RoG (average = 19.58 Å), and the change in the number of H-bonds (average = 58 bonds) showed that the protein is stable and did not undergo a change in its folded state. The change in the distance between the centre of mass of compound **12b** and that of the protein showed a stable binding with an average distance of 9.95 Å.

**Figure 15.**
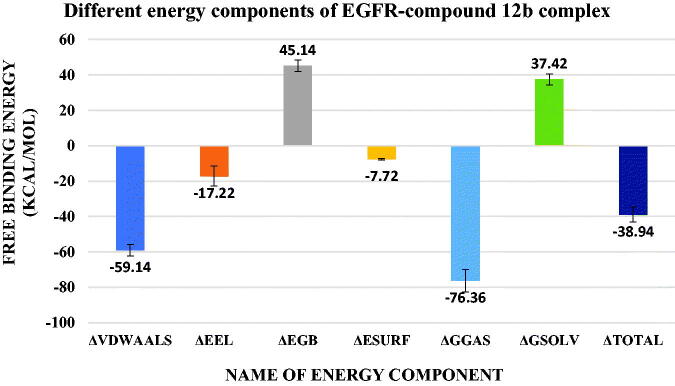
values of different energy components obtained from MM-GBSA analysis. Bars represent the standard deviation values.

**Figure 16.**
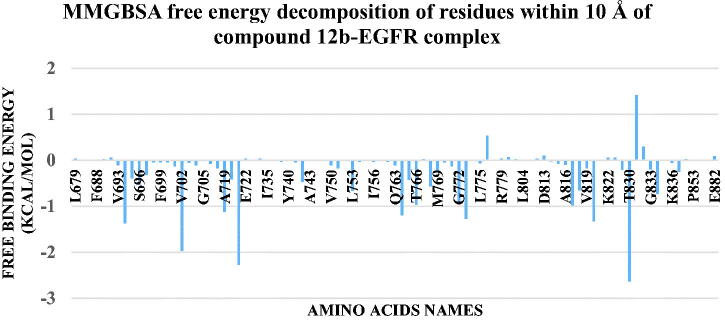
The decomposition of the free binding energy of amino acids around 10 Å of compound.**12b**.

The binding free energy between the protein and compound **12b** was calculated using MM-GBSA implemented in the gmx_MMPBSA tool. Figure 15 showed the values of different energy components produced from MM-GBSA. The predominant type of interaction is the Van Der Waals interactions with an average of −59.14 Kcal/mol followed by electrostatic interactions with an average of −17.22 Kcal/mol. Decomposition analysis was performed to give information on the contribution of amino acids to the binding. Figure 16 showed that L694 (−1.36 Kcal/mol), V702 (−1.96 Kcal/mol), A719 (−1.12 Kcal/mol), K721 (−2.27 Kcal/mol), L764 (−1.19 Kcal/mol), C773 (−1.27 Kcal/mol), L820 (−1.32 Kcal/mol), and T830 (−2.63 Kcal/mol) have a contribution of values stronger (more negative) than −1 Kcal/mol. Only one amino acid (D831) shows a positive contribution to the binding with a value of +1.4 Kcal/mol.

**Figure 17.**
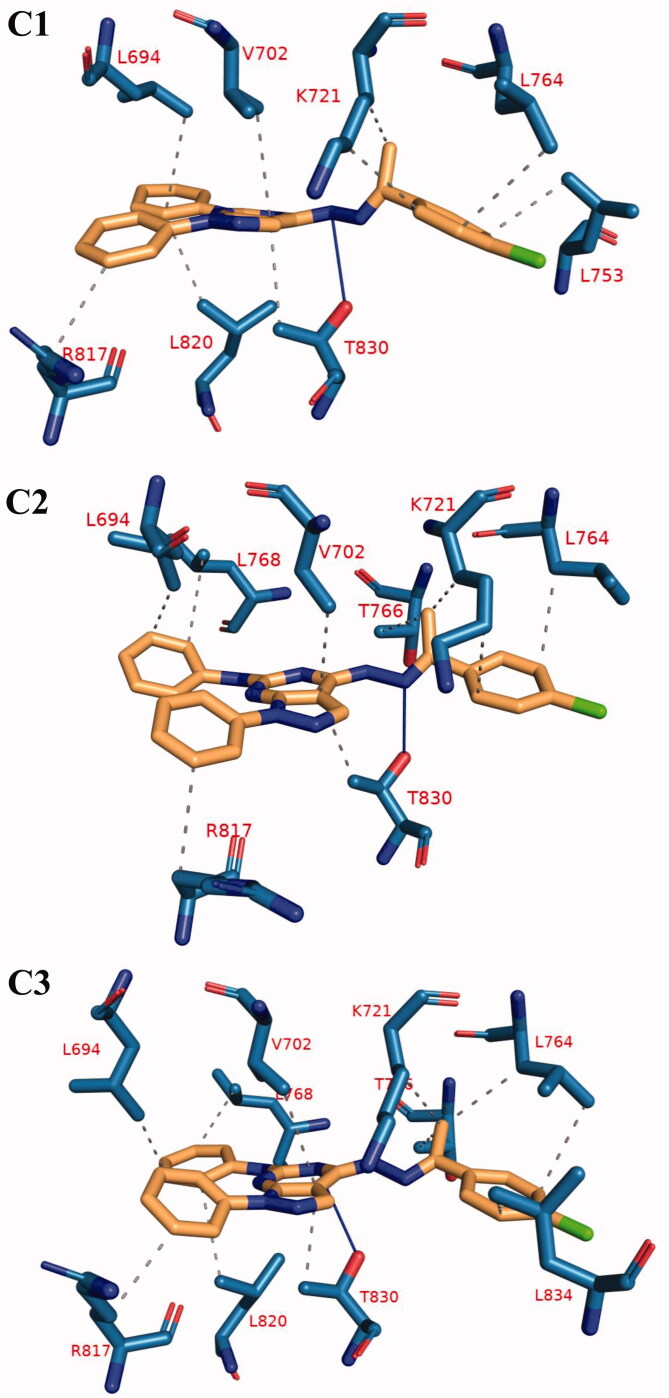
The 3 D interaction between compound **12 b** and EGFR in each of the representative frame for each cluster. Amino acids are shown as blue sticks. compound **12 b** is shown as brown sticks. Grey dashed lines: hydrophobic interaction. Blue solid lines: hydrogen bonds.

The trajectory was clustered using TTClust to obtain the different clusters and a representative frame for each one. To know the different types and numbers of interactions, PLIP was utilised to detect the interactions between compound **12b** and the protein in the representative frames for each cluster. Table 10 showed the types and numbers of interactions produced from PLIP. Most of the interactions are hydrophobic with only one amino acid forming a hydrogen bond with the compound **12b**. Figure 17 showed the 3D conformations for the complex in representative frames of each cluster.

**Table 10.** The number and types of interactions between compound **12 b** and **EGFR** as obtained from PLIP webserver for the representative frame of each cluster. Amino acids in bold are common in all of the clusters representative.

Cluster number	Number of hydrophobic interactions	Amino acids in receptor	Number of hydrogen bonds	Amino acids in receptor
**C1**	9	L694 - V702 - K721 (2) - L753 - L764 - R817 - L820 - T830	1	T830
**C2**	9	L694 - V702 - K721 (2) - L764 - T766 - L768 - R817 - T830	1	T830
**C3**	11	L694 - V702 - K721 - L764 (2) - T766 - L768 - R817 - L820 - T830 - L834	1	T830

## 3. Conclusion

Twelve 1*H*-pyrazolo[3,4-d]pyrimidine derivatives having the essential pharmacophoric features of EGFR inhibitors have been designed and synthesised. Four compounds **8, 10, 12a,** and **12b** showed potent anti-proliferative activities against A549 and HCT-116 cell lines. Compound **12b** (IC_50_ = 8.21 and 19.56 µM) exhibited the highest activity against A549 and HCT-116, respectively. The inhibitory activities of compound **12b** against EGFR^WT^ and EGFR^T790M^ were 0.016 and 0.236 µM, respectively compared to erlotinib (IC_50_ = 0.006 and 0.563 µM, respectively). SAR study revealed that the introduction of aliphatic amines in the 4-position of pyrazolo[3,4-*d*]pyrimidine scaffold was not beneficial for cytotoxic activity. On the contrary, the introduction of aniline moiety in the same position enhanced the anticancer activity. Additionally, the condensation of hydrazine derivative **10** with *p*-chloroacetophenone gave better anticancer activity against A549 cells. The effect of compound **12b** on cell cycle distribution and apoptosis induction was analysed. Such a compound provoked apoptosis and arrested the cell cycle at *S* and G2/M phases. Moreover, compound **12b** induced a high expression level of BAX and a low expression level of Bcl-2 in A549 cells indicating its apoptotic behaviour. Compound **12b** showed low toxicity against WI-38 cell line compared to erlotinib. In addition, it showed a high selectivity against the tumour cell lines. Docking studies suggested that the synthesised compounds have good binding modes against EGFR^WT^ and EGFR^T790M^ crystal structures.

## 4. Experimental

### 4.1. Chemistry

#### 4.1.1. General

^1^H NMR spectra were run at 400 MHz and ^13 ^C NMR spectra were determined at 101 MHz in deuterated dimethyl sulfoxide (DMSO-d_6_) on a Varian Mercury VX-400 NMR spectrometer. Chemical shifts are given in parts per million (ppm) on the delta (d) scale. Chemical shifts were calibrated relative to those of the solvents. The progress of reactions was monitored with Merck silica gel IB2-F plates (0.25 mm thickness). The infra-red spectra were recorded in potassium bromide discs on Pye Unicam SP 3300 and Shimadzu FT IR 8101 PC infra-red spectrophotometer. Elemental analyses (C, H, N) were performed on a CHN analyser, and all compounds were within ± 0.4 of the theoretical values. Compounds **2**, **3**, **4**, and **5** were prepared according to reported procedures ^44–46^.

#### 4.1.2. 4-Chloro-N,1-diphenyl-4,5-dihydro-1H-pyrazolo[3,4-d]pyrimidin-6-amine (6)

A solution of 4,6-dichloro-1-phenyl-1*H*-pyrazolo[3,4-d]pyrimidine **5** (2.65 g, 0.01 mol) and aniline (0.01 mol) in absolute ethanol (20 ml) was heated under reflux for 6 h. Then, the solvent was evaporated under reduced pressure. The resulting precipitate was filtered, dried, and crystallised from absolute ethanol to afford the corresponding target compound **6**.

#### 4.1.3. General procedure for the synthesis of compounds 7a,b, 8, and 9

A mixture of 4-chloro-*N*,1-diphenyl-4,5-dihydro-1*H*-pyrazolo[3,4-*d*]pyrimidin-6-amine **6** (0.5 g, 1.54 mmol) and appropriate amines namely, ethylamine, propylamine, aniline, and cyclohexylamine in the presence of TEA (15 ml) was refluxed for 4 h. Then, the solvent was evaporated under reduced pressure. The resulting precipitate was filtered, dried, and crystallised from absolute ethanol to afford the corresponding target compounds ***7a,b, 8,*** *and* ***9,*** respectively.

##### 4.1.3.1. N4-Ethyl-N6,1-diphenyl-1H-pyrazolo[3,4-d]pyrimidine-4,6-diamine (7a)

White solid, yield: 85%; m.p. 213–215 °C. IR (KBr) cm^−1^: 3282 (NH), 3028 (CH aromatic), 2924 (CH aliphatic); ^1^H NMR (400 MHz, DMSO-d_6_) δ 8.64 (*s*, 1H), 8.35 (*s*, 1H), 8.26 − 8.19 (*m*, 2H), 7.76 (dd, *J* = 20.5, 6.8 Hz, 3H), 7.56 − 7.48 (*m*, 2H), 7.35 (*t*, *J* = 7.7 Hz, 2H), 7.25 (*t*, *J* = 7.4 Hz, 1H), 6.95 (*t*, *J* = 7.3 Hz, 1H), 3.68 − 3.57 (*m*, 2H), 1.26 (*t*, *J* = 7.1 Hz, 3H); ^13 ^C NMR (101 MHz, DMSO-d_6_) δ 157.13, 156.56, 153.52, 144.58, 142.39, 139.64, 129.47(2 C), 129.29 (2 C), 125.27, 121.10, 120.25(2 C), 117.91(2 C), 93.51, 35.73, 15.09; DEPT (DMSO-d_6_) (ppm)δ 157.13, 129.47, 129.29, 125.27, 121.10, 120.25, 117.91, 35.73, 15.09; (C_19_H_18_N_6_) (M.W. = 331).

##### 4.1.3.2. N4-Propyl-N6,1-diphenyl-1H-pyrazolo[3,4-d]pyrimidine-4,6-diamine (7b)

White solid, Yield: 82%; m.p. 222–224 °C; IR (KBr) cm-1: 3275 (NH), 3099 (CH aromatic), 2927 (CH aliphatic); ^1^H NMR (400 MHz, DMSO-d_6_) δ 8.65 (*s*, 1H), 8.35 (*s*, 1H), 8.22 (d, *J* = 8.1 Hz, 2H), 7.74 (dd, *J* = 17.4, 6.9 Hz, 3H), 7.56 − 7.48 (*m*, 2H), 7.39 − 7.31 (*m*, 2H), 7.25 (*t*, *J* = 7.4 Hz, 1H), 6.95 (*t*, *J* = 7.3 Hz, 1H), 3.54 (dd, *J* = 7.9, 5.9 Hz, 2H), 1.67 (h, *J* = 7.4 Hz, 2H), 0.95 (*t*, *J* = 7.4 Hz, 3H); ^13 ^C NMR (101 MHz, DMSO-d_6_) δ 157.12, 156.73, 153.55, 144.58, 142.41, 139.63, 129.47(2 C), 129.30(2 C), 125.28, 121.12, 120.27 (2 C), 117.92 (2 C), 93.51, 42.58, 22.66, 11.88; DEPT (DMSO-d_6_) (ppm)δ 157.12, 129.47, 129.30, 125.28, 121.12, 120.27, 117.92, 42.58, 22.66, 11.88; Anal. Calc. for: (C_20_H_20_N_6_) (M.W. = 344).

##### 4.1.3.3. N4,N6,1-Triphenyl-1H-pyrazolo[3,4-d]pyrimidine-4,6-diamine (8)

White solid, Yield: 87%; m.p. 255–257 °C. IR (KBr) cm^−1^: 3387 (NH), 3086 (CH aromatic). ^1^H NMR (400 MHz, DMSO-d_6_) δ 9.28 (*s*, 1H), 8.94 (*s*, 1H), 8.43 (*s*, 1H), 8.24 (d, *J* = 8.1 Hz, 2H), 7.74 (d, *J* = 8.1 Hz, 2H), 7.68 (d, *J* = 7.9 Hz, 2H), 7.55 (*t*, *J* = 7.8 Hz, 2H), 7.40 (dt, *J* = 24.4, 7.7 Hz, 4H), 7.29 (*t*, *J* = 7.3 Hz, 1H), 7.20 (*t*, *J* = 7.4 Hz, 1H), 6.97 (*t*, *J* = 7.3 Hz, 1H); ^13 ^C NMR (101 MHz, DMSO-d_6_) *δ* 156.78, 155.16, 153.80, 144.40, 142.37, 139.43, 138.87, 129.57 (2 C), 129.36, 129.09 (2 C), 125.61, 124.87, 123.79 (2 C), 121.23, 120.46(2 C), 117.93 (2 C), 94.58; DEPT (DMSO-d_6_) (ppm) *δ* 156.78, 129.57, 129.36, 129.09, 125.60, 124.87, 123.79, 121.23, 120.46, 117.93; (C_23_H_18_N_6_) (M.W. = 378).

##### 4.1.3.4. N4-Cyclohexyl-N6,1-diphenyl-1H-pyrazolo[3,4-d]pyrimidine-4,6-diamine (9)

White solid, Yield: 80%; m.p. 235–237 °C. IR (KBr) cm^−1^: 3294 (NH), 3066 (CH aromatic), 2947 (CH aliphatic); ^1^H NMR (400 MHz, DMSO-d_6_) δ 8.84 (*s*, 1H), 8.35 (*s*, 1H), 8.22 (d, *J* = 8.1 Hz, 2H), 7.66 (d, *J* = 8.0 Hz, 2H), 7.52 (*t*, *J* = 7.8 Hz, 2H), 7.35 (*t*, *J* = 7.8 Hz, 2H), 7.26 (*t*, *J* = 7.4 Hz, 1H), 7.19 (d, *J* = 7.7 Hz, 1H), 6.96 (*t*, *J* = 7.5 Hz, 1H), 4.22 (*s*, 1H), 2.52 (*s*, 2H), 1.99 (*s*, 2H), 1.76 (*s*, 2H), 1.66 (d, *J* = 12.9 Hz, 1H), 1.35 (d, *J* = 9.8 Hz, 2H), 1.19 (*s*, 1H); ^13 ^C NMR (101 MHz, DMSO-d_6_) δ 157.12, 155.93, 153.73, 144.52, 142.54, 139.58, 129.48(2 C), 129.40(2 C), 125.36, 121.16, 120.33(2 C), 117.89(2 C), 93.75, 49.89, 32.64(2 C), 25.78, 25.35(2 C); (C_23_H_24_N_6_) (M.W. = 384).

##### 4.1.3.5. 4-Hydrazinyl-N,1-diphenyl-1H-pyrazolo[3,4-d]pyrimidin-6-amine (10)

A mixture of 4-chloro-1,6-diphenyl-1*H*-pyrazolo[3,4-*d*]pyrimidine (3.21 g, 0.01 mol) and hydrazine hydrate (99%, 5 ml, 0. 1 mol) was heated under reflux for 4 h. After cooling, the formed solid was collected by filtration, washed with hot ethanol (95%, 10 ml), and crystallised form isopropanol to yield the desired product **10**.

#### 4.1.4. General procedure for synthesis of compounds 11a,b, 12a,b, and 13a,b

A mixture of hydrazide derivative **10** (0.31 g, 0.001 mol), appropriate aromatic aldehydes or acetophenones (0.001 mol), and a catalytic amount of glacial acetic acid (0.5 ml) was heated under reflux in absolute ethanol (20 ml) for a specific time. The formed precipitate was filtered and crystallised from ethanol to yield the title compounds ***11a,b, 12a,b,*** *and* ***13a,b.***

##### 4.1.4.1. 4–(2-(4-Methoxybenzylidene)hydrazinyl)-N,1-diphenyl-1H-pyrazolo[3,4-d] pyrimidin-6-amine (11a)

White solid, Yield: 80%; m.p. 265–267 °C; IR (KBr) cm^−1^: 3290 (NH), 3055 (CH aromatic), 2935 (CH aliphatic). ^1^H NMR (400 MHz, DMSO-d_6_) *δ* 12.11 (*s*, 1H), 9.91 (s, 1H), 8.44 (d, *J* = 11.5 Hz, 1H), 8.28 (d, *J* = 8.1 Hz, 1H), 8.14 (d, *J* = 8.5 Hz, 1H), 7.77 − 7.66 (*m*, 2H), 7.59 − 7.46 (*m*, 4H), 7.38 (*t*, *J* = 8.0 Hz, 1H), 7.34 − 7.23 (*m*, 3H), 7.09 − 7.02 (*m*, 2H), 6.99 − 6.90 (*m*, 1H), 3.84 (*s*, 3H); ^13 ^C NMR (101 MHz, DMSO-d_6_) δ 161.76, 161.27, 149.42, 147.77, 147. 49, 144.81, 139.50, 132, 28, 129.84, 129.73, 129.58, 129.50, 129.24, 128.38, 126.10, 125.45, 121.19, 120.63, 120.44, 118.06, 116.81, 115.17, 114.62, 92.87, 55.95; DEPT (DMSO-d_6_) (ppm) *δ* 153.77, 149.42, 129.84, 129.73, 129.50, 129.24, 125.45, 121.19, 120.63, 120.44, 118.06, 116.81, 115.17, 114.62, 55.95; (C_25_H_21_N_7_O) (M.W. = 435).

##### 4.1.4.2. 4–(2-(4-Chlorobenzylidene)hydrazinyl)-N,1-diphenyl-1H-pyrazolo[3,4-d]pyrimidin-6-amine (11b)

White solid, Yield: 78%; m.p. 274–276 °C; IR (KBr) cm^−1^: 3390 (NH), 3074 (CH aromatic), 2981 (CH aliphatic). ^1^H NMR (400 MHz, DMSO-d_6_) *δ* 12.23 (*s*, 1H), 9.69 (*s*, 1H), 8.59 (*s*, 1H), 8.49 (d, *J* = 15.0 Hz, 1H), 8.28 (d, *J* = 8.1 Hz, 1H), 8.20 (*s*, 1H), 8.14 (d, *J* = 8.0 Hz, 1H), 8.03 − 7.98 (*m*, 1H), 7.76 (*t*, *J* = 7.1 Hz, 2H), 7.56 (d, *J* = 7.9 Hz, 3H), 7.40 (dd, *J* = 14.8, 7.4 Hz, 2H), 7.33 − 7.24 (*m*, 2H), 6.95 (dt, *J* = 13.9, 7.3 Hz, 1H); ^13 ^C NMR (101 MHz, DMSO-d_6_) *δ* 152.65, 149.38, 148.81, 147.53, 141.08, 140.94, 139.14, 135.58, 134.73, 129.79, 129.75, 129.67, 129.61, 129.54, 129.25, 129.21, 126.10, 121.27, 120.69, 120.51, 118.01, 116.85, 93.37, 93.13; DEPT (DMSO-d_6_) (ppm) *δ* 152.65, 149.39, 129.75, 129.67, 129.61, 129.54, 129.25, 126.10, 125.57, 121.27, 121.07, 120.69, 120.51, 118.00, 116.85. (C_24_H_18_ClN_7_) (M.W. = 439).

##### 4.1.4.3. N,1-Diphenyl-4–(2-(1-phenylethylidene)hydrazinyl)-1H-pyrazolo[3,4-d]pyrimidin-6-amine (12a)

White solid, Yield: 70%; m.p. 244–246 °C; IR (KBr) cm^−1^: 3402 (NH), 3032 (CH aromatic), 2982 (CH aliphatic). ^1^H NMR (400 MHz, DMSO-d_6_) *δ* 11.72 (*s*, 1H), 10.46 (*s*, 1H), 9.98 (*s*, 1H), 8.37 (d, *J* = 28.7 Hz, 1H), 8.29 − 8.20 (*m*, 2H), 8.11 (dd, *J* = 21.8, 7.7 Hz, 2H), 7.97 (*s*, 1H), 7.73 (*s*, 1H), 7.64 (d, *J* = 8.0 Hz, 1H), 7.52 (*t*, *J* = 8.0 Hz, 3H), 7.39 − 7.35 (*m*, 1H), 7.29 − 7.24 (*m*, 1H), 7.00 − 6.94 (*m*, 1H), 6.77 (d, *J* = 8.3 Hz, 1H), 1.93 (*s*, 3H); ^13 ^C NMR (101 MHz, DMSO-d_6_) *δ* 172.49, 156.24, 149.42, 148.00, 144.57, 141.42, 141.01, 139.13, 130.18, 129.57, 128.81, 128.60, 127.77, 127.14, 125.89, 125.25, 120.93, 120.55, 120.31, 117.22, 117.19, 117.13, 116.57, 93.39, 21.52; (C_25_H_21_N_7_) (M.W. = 419).

##### 4.1.4.4. 4–(2-(1–(4-Chlorophenyl)ethylidene)hydrazinyl)-N,1-diphen yl-1H-pyrazolo[3,4-d]pyrimidin-6-amine (12b)

White solid; Yield: 80%; m.p. 248–250 °C; IR (KBr) cm^−1^: 3387 (NH), 3098 (CH aromatic), 2961 (CH aliphatic); ^1^H NMR (400 MHz, DMSO-d_6_) *δ* 12.06 (*s*, 1H), 9.76 (*s*, 1H), 8.47 (d, *J* = 13.8 Hz, 1H), 8.23 (*s*, 1H), 8.14 (dd, *J* = 8.2, 5.6 Hz, 2H), 8.02 (*s*, 1H), 7.78 (d, *J* = 8.1 Hz, 1H), 7.66 (d, *J* = 8.0 Hz, 1H), 7.53 (dd, *J* = 21.4, 8.2 Hz, 3H), 7.39 (*t*, *J* = 7.7 Hz, 1H), 7.32 (*t*, *J* = 7.6 Hz, 1H), 7.05 (*t*, *J* = 7.8 Hz, 1H), 6.97 (*t*, *J* = 7.3 Hz, 1H), 6.80 (d, *J* = 8.2 Hz, 1H), 2.52 (*s*, 3H); ^13 ^C NMR (101 MHz, DMSO-d_6_) δ 156.80, 149.51, 148.70, 147.63, 147.55, 140.99, 139.19, 137.82, 134.32, 130.57, 129.71, 129.63, 129.60, 129.52, 129.26, 128.89, 128.79, 128.63, 126.04, 121.06, 120.60, 117.19, 116.62, 94.07, 14.25; DEPT (DMSO-d_6_) (ppm) *δ* 149.51, 130.57, 129.71, 129.60, 129.52, 129.26, 128.89, 128.79, 128.63, 126.04, 121.05, 120.73, 120.60, 117.19, 116.62, 14.24; (C_25_H_20_ClN_7_) (M.W. = 453).

##### 4.1.4.5. N,1-Diphenyl-4–(2-(1-(p-tolyl)ethylidene)hydrazinyl)-1H-pyrazolo [3,4-d] pyrimidin-6-amine (12c)

White solid, Yield: 85%; m.p. 251–253 °C; IR (KBr) cm^−1^: 3367 (NH), 3047 (CH aromatic), 2968 (CH aliphatic). ^1^H NMR (400 MHz, DMSO-d_6_) *δ* 11.87 (*s*, 1H), 10.50 (*s*, 1H), 8.38 (*s*, 1H), 8.28 (d, *J* = 8.0 Hz, 2H), 7.76 (d, *J* = 6.8 Hz, 3H), 7.55 (*t*, *J* = 7.4 Hz, 3H), 7.39 (*t*, *J* = 7.1 Hz, 3H), 7.28 (*t*, *J* = 7.3 Hz, 2H), 6.96 (d, *J* = 7.4 Hz, 1H), 2.31 − 2.12 (*m*, 6H); ^13 ^C NMR (101 MHz, DMSO-d_6_) *δ* 156.36, 154.03, 144.62, 144.58, 141.40, 139.53, 136.31, 129.67, 129.60, 129.50, 129.27, 128.80, 128.71, 125.38, 121.04, 212.55, 121.04, 120.45, 120.55, 120.37, 117.58, 117.46, 117.24, 92.34, 18.87, 18.49; DEPT (DMSO-d_6_) (ppm) *δ* 129.67, 129.60, 129.50, 129.20, 125.39, 121.55, 121.04, 120.37, 117.58, 117.51, 117.24, 18.87, 18.49; (C_26_H_23_N_7_) (M.W. = 433).

##### 4.1.4.6. N-Ethyl-2–(1-phenyl-6-(phenylamino)-1H-pyrazolo[3,4-d]pyrimidin-4-yl) hydrazine-1-carbothioamide (13a)

Yellowish solid, Yield: 72%; m.p. 250–252 °C. IR (KBr) cm^−1^: 3250 (NH), 3060 (CH aromatic), 2950 (CH aliphatic). ^1^H NMR (300 MHz, DMSO-d_6_) δ 9.89 (*s*, 1H), 9.65 (*s*, 1H), 8.50 (*s*, 1H), 8.28 − 8.17 (*m*, 2H), 8.10 (dd, *J* = 12.6, 6.2 Hz, 1H), 7.85 (*s*, 1H), 7.58 − 7.51 (*m*, 3H), 7.36 − 7.27 (*m*, 3H), 7.03 − 6.85 (*m*, 2H), 3.50 (*m*, 2H), 1.24 − 1.12 (*t*, 3H); (C_20_H_20_N_8_S) (M.W = 404).

##### 4.1.4.7. 2–(1-Phenyl-6-(phenylamino)-1H-pyrazolo[3,4-d]pyrimidin-4-yl)-N-propyl hydrazine-1-carbothioamide (13b)

Yellowish solid, yield: 70%; m.p. 244–246 °C. IR (KBr) cm^−1^: 3256 (NH), 3035 (CH aromatic), 2934 (CH aliphatic). ^1^H NMR (300 MHz, DMSO-d_6_) δ 9.65 (*s*, 1H), 8.51 (*s*, 1H), 8.41 (*s*, 1H), 8.25 (d, *J* = 8.4 Hz, 1H), 8.14 − 8.04 (*m*, 1H), 7.89 − 7.81 (*m*, 1H), 7.76 (d, *J* = 6.0 Hz, 1H), 7.55 (dt, *J* = 13.8, 6.8 Hz, 3H), 7.42 − 7.25 (*m*, 4H), 7.01 − 6.88 (*m*, 1H), 3.62 − 3.38 (*t*, 2H), 1.69 − 1.43 (*m*, 2H), 0.97 − 0.71 (*t*, 3H); (C_21_H_22_N_8_S) (M.W = 418).

### 4.2. Biological evaluation

#### 4.2.1. *In vitro* cytotoxic activity

*In vitro* cytotoxicity was carried out for the synthesised compounds against A549, HCT-116, and WI-38 cell lines using the MTT assay protocol^52–55^ as described in Supplementary data.

#### 4.2.2. *In vitro* EGFR kinase assay

*In vitro* **EGFR** inhibitory activity was assessed using a Homogeneous time-resolved fluorescence (HTRF) assay^56^ as described in **Supplementary data**.

#### 4.2.3. Cell cycle analysis

The effect of compound **12b** on cell cycle distribution was performed using the propidium iodide (PI) staining technique as described in **Supplementary data**^56–58^.

#### 4.2.4. Apoptosis analysis

The effect of compound **12b** on cell apoptosis was investigated as described in **Supplementary data**^59^.

#### 4.2.5. Quantitative Real-Time Reverse-Transcriptase PCR (qRT-PCR) technique

The effect of compound **12b** on the expression of BAX and Bcl‐2 was determined using qRT-PCR as described in **Supplementary data**^60–62^.

### 4.3. *In silico* studies

#### 4.3.1. Docking studies

Molecular docking studies of the synthesised compounds were carried out against EGFR^WT^ (PDB ID: 4HJO, resolution 2.75 Å and EGFR^T790M^ (PDB ID: 3W2O, resolution 2.35 Å) as described in **Supplementary data**^38^.

#### 4.3.2. ADMET studies

ADMET descriptors were determined using Discovery studio 4.0 according to the reported method^63^^,^^64^ (**Supplementary data**).

#### 4.3.3. Toxicity studies

The toxicity parameters of the synthesised compounds were calculated using Discovery studio 4.0^65^^,^^66^ as described in **Supplementary data**.

#### 4.3.4. M D Simulations and MM-GBSA

CHARMM-GUI web server was employed and GROMACS 2021 was used as an MD engine as outlined thoroughly in **Supplementary data.** The Gmx_MMPBSA package was used as outlined thoroughly in **Supplementary data**^67–70^.

## Supplementary Material

Supplemental Material
