# Supplementary material for: Discovery of new 1H-pyrazolo[3,4-d]pyrimidine derivatives as anticancer agents targeting EGFRWT and EGFRT790M
Source: J Enzyme Inhib Med Chem. 2022 Aug 23;37(1):2283–303. doi: 10.1080/14756366.2022.2112575 (PMC9466626; doi:10.1080/14756366.2022.2112575)
Supplement: Supplemental Material [file IENZ_A_2112575_SM4683.pdf]

Discovery of new 1*H*-pyrazolo[3,4-*d*]pyrimidine derivatives as anticancer agents targeting EGFR^{WT} and EGFR^{T790M}

Ahmed A. Gaber[a], Mohamed Sobhy[a], Abdallah Turky[a], Hanan Gaber Abdulwahab[b], Ahmed A. Al-Karmalawy[c], Mostafa. A. Elhendawy[d,e], Mohamed. M. Radwan[d,f], Eslam B. Elkaeed[g], Ibrahim M. Ibrahim[h], Heba S. A. Elzahabi[b], Ibrahim H. Eissa[i],

[a] Department of Pharmaceutical Organic Chemistry, Faculty of Pharmacy (Boys), Al-Azhar University, Cairo 11884, Egypt.

[b] Department of Pharmaceutical Medicinal Chemistry and Drug Design, Faculty of Pharmacy (Girls), Al-Azhar University, Cairo, Egypt

[c] Department of Pharmaceutical Medicinal Chemistry, Faculty of Pharmacy, Horus University-Egypt, New Damietta 34518, Egypt.

[d] National Center for Natural Products Research, University of Mississippi, MS 38677, USA.

[e] Department of Agriculture Chemistry, Faculty of Agriculture, Damietta University, Damietta, Egypt

[f] Department of Pharmacognosy, Faculty of Pharmacy, Alexandria University, Alexandria, Egypt.

[g] Department of Pharmaceutical Sciences, College of Pharmacy, AlMaarefa University, Riyadh 13713, Saudi Arabia.

[h] Biophysics Department, Faculty of Science, Cairo University. Cairo 12613, Egypt.

[i] Pharmaceutical Medicinal Chemistry & Drug Design Department, Faculty of Pharmacy (Boys), Al-Azhar University, Cairo 11884, Egypt.

* **Corresponding author:**

Ibrahim H. Eissa

Pharmaceutical Medicinal Chemistry & Drug Design Department, Faculty of Pharmacy (Boys), Al-Azhar University, Cairo 11884, Egypt

Email: Ibrahimeissa@azhar.edu.eg

Content

**1**	Biological tests
**2**	In silico studies (Docking, ADMET, and toxicity procedures, MD simulations)
**3**	IC_{50} Graphs
**4**	Spectral data

1. Biological tests

1.1. In vitro antiproliferative activities

The *in vitro* antiproliferative activities of all the synthesized compounds against two human tumor cell lines namely, (lung, A549) and (colon HCT-116) and one normal cell line (WI-38) were evaluated quantitatively as described in the literature, using MTT assay protocol. two commercially available drugs (erlotinib) were used in this test as positive controls. The anti-proliferative activity was assessed quantitatively as follows.

Human cancer cell lines were dropped in 96-well plates at a density of 3-8 × 10^3 cells/well. Next, the wells were incubated for 12 h in a 5% CO_2 incubator at 37 °C. Then, for each well, the growth medium was exchanged with 0.1 ml of fresh medium containing graded concentrations of the test compounds to be or equal DMSO and incubated for two days. Then 10 µl MTT solution (5 µg/ml) was added to each well, and the cells were incubated for additional 4 h. The crystals of MTT-formazan were dissolved in 100 µl of DMSO; the absorbance of each well was measured at 490 nm using an automatic ELISA reader system (TECAN, CHE). The IC_{50} values were calculated using the nonlinear regression fitting models (Graph Pad, Prism Version 5). The data represented the mean of three independent experiments in triplicate and were expressed as means ± SD. The IC_{50} value was defined as the concentration at which 50% of the cells could survive.

The results were expressed as growth inhibitory concentration (IC_{50}) values and summarized in **Table 1**.

4.1.1. EGFR^{WT} and EGFR^{T790M} kinase inhibitory assay

The most active cytotoxic compounds (**8, 10, 12a,** and **12b)** that showed promising IC_{50} values against four cancer cell lines were further examined for their inhibitory activities against both EGFR^{WT}. compound 12b was examined against EGFR^{T790M}. Homogeneous time resolved fluorescence (HTRF) assay was applied in this test. EGFR^{WT}, EGFR^{T790M} (Sigma). firstly, the EGFR^{WT} and/or EGFR^{T790M} and their substrates were incubated with the tested compounds in enzymatic buffer for 5 min. ATP (1.65 µM) was added into the reaction mixture to allow starting the enzymatic reaction. The assay was conducted for 30 min at room temperature. The reaction was stopped by addition of detection reagents which contain EDTA. The detection step continued for 1 h, and then the IC_{50} values were determined by GraphPad Prism 5.0. Three independent experiments were performed for each concentration.

4.1.2. *In-vitro* DNA-Flow cytometric (cell cycle) analysis.

A549 cells were exposed to the most active member **12b** at concentrations of 8.21 µM µM for 48 h. Then, the tested cells were collected by trypsinization and washed in PBS. Ice-cold absolute ethanol was used for fixation of the collected cells. The cells were stained with Cycle TESTTM PLUS DNA Reagent Kit (BD Biosciences, San Jose, CA) according to the manufacturer's instructions. Cell-cycle distribution was evaluated using a flow cytometer.

4.1.3. Annexin V-FITC apoptosis assay.

To detect the apoptosis induced by compound **12b**, a549 cells were seeded and incubated overnight and then treated with compound **12b** at concentrations of 8.21 µM µM for 48 h. DMSO was chosen as the negative control. After that, the cells were collected and washed with PBS two successive times. The cells were exposed to centrifugation. Apoptosis detection kit (BD Biosciences, San Jose, CA) was used in this test. According to the manufacturer's protocol the cells were stained by Annexin V-FITC and propidium iodide (PI) in the binding buffer for 20min at room temperature in the dark. Using a flow cytometer, Annexin V-FITC and PI binding were analyzed. flowjo software was used to analyze the frequencies in all quadrants [88].

Docking studies

The crystal structures of the target enzymes $EGFR^{WT}$ (PDB ID: 4HJO, resolution 2.75 Å and $EGFR^{T790M}$ (PDB ID: 3W2O, resolution 2.35 Å) were downloaded from Protein Data Bank (http://www.pdb.org). Molecular Operating Environment (MOE) was used for the docking analysis [91]. In these studies, the free energies and binding modes of the designed molecules against $EGFR^{WT}$ and $EGFR^{T790M}$ were determined. At first, the water molecules were removed from the crystal structures of $EGFR^{WT}$ and $EGFR^{T790M}$, retaining only one chain in each enzyme. Erlotinib and TAK-285 (The co-crystallized ligands) were utilized as references in the docking processes against both $EGFR^{WT}$ and $EGFR^{T790M}$, respectively. After that, in order to prepare the target molecules for binding with the designed compounds, the target proteins were subjected to protonation step. Then, the hydrogen atoms were hidden to make the areas of interaction clearer. Next, the energy of all systems was minimized followed by identification the binding pockets of the target proteins.

The structures of the designed compounds and the co-crystallized ligands, erlotinib and TAK-285 were drawn using ChemBioDraw Ultra 14.0 and saved as SDF format. Then, the saved files were opened using MOE 2019 and 3D structures were protonated. Next, the energy of the molecules was minimized. Validation process was performed for each target by running the docking process for only the co-crystallized ligand. Low RMSD values between docked and crystal conformations indicate valid performance. The docking procedures were carried out utilizing a default protocol. In each case, 10 docked structures were generated using genetic algorithm searches. The output from of MOE2019 was further analyzed and visualized using Discovery Studio software.

ADMET studies

ADMET descriptors (absorption, distribution, metabolism, excretion and toxicity) of the compounds were determined using Discovery studio 4.0. At first, the CHARMM force field was applied then the tested compounds were prepared and minimized according to the preparation of small molecule protocol. Then ADMET descriptors protocol was applied to carry out these studies.

Toxicity studies

The toxicity parameters of the synthesized compounds were calculated using Discovery studio 4.0. Sorafenib was used as a reference drug. At first, the CHARMM force field was applied then the compounds were prepared and minimized according to the preparation of small molecule protocol. Then different parameters were calculated from toxicity prediction (extensible) protocol.

Mapping surface showing erlotinib occupying the active pocket of EGFR^{WT}.

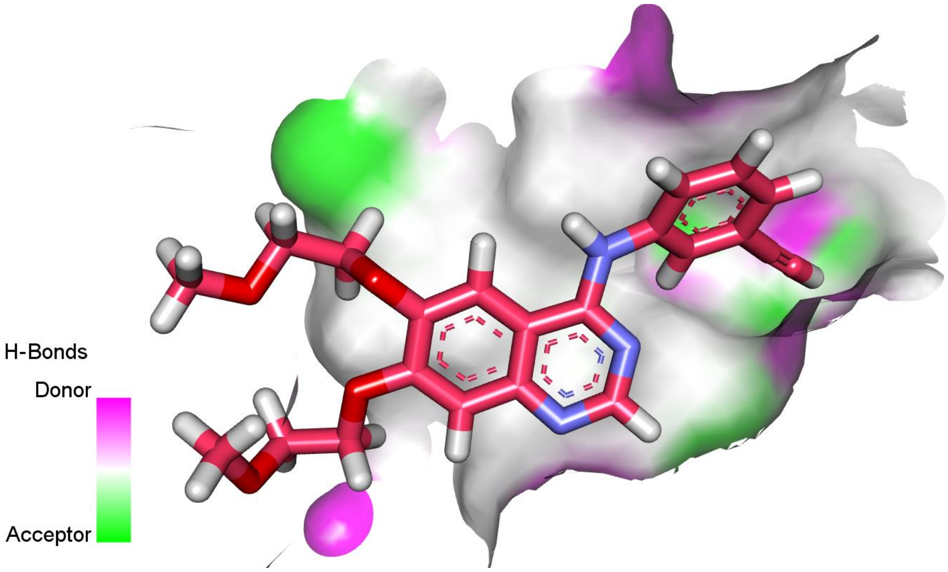

Mapping surface showing compound 12b occupying the active pocket of EGFR^{WT}.

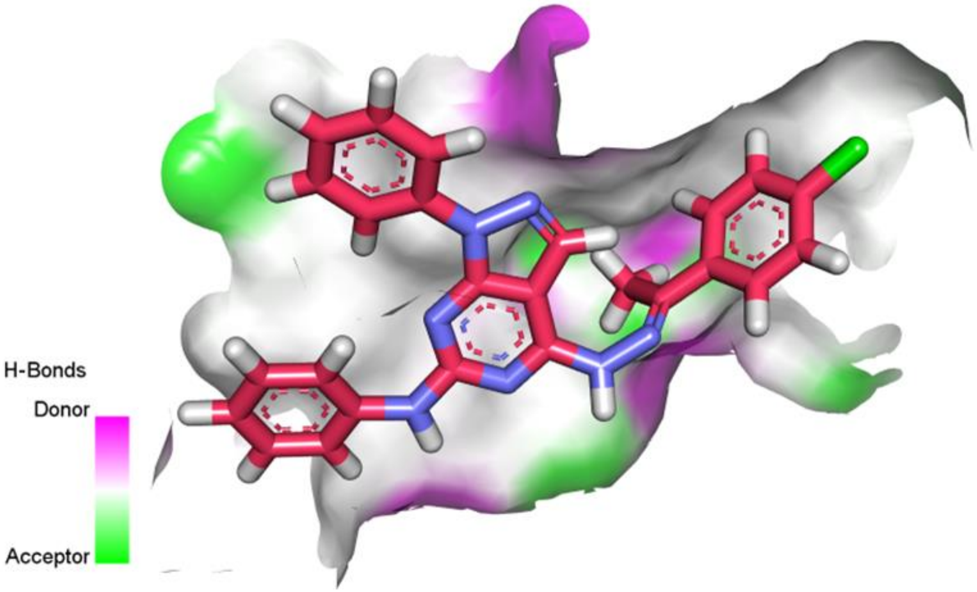

Mapping surface showing TAK-285occupyin g the active pocket of EGFR^{T790M}.

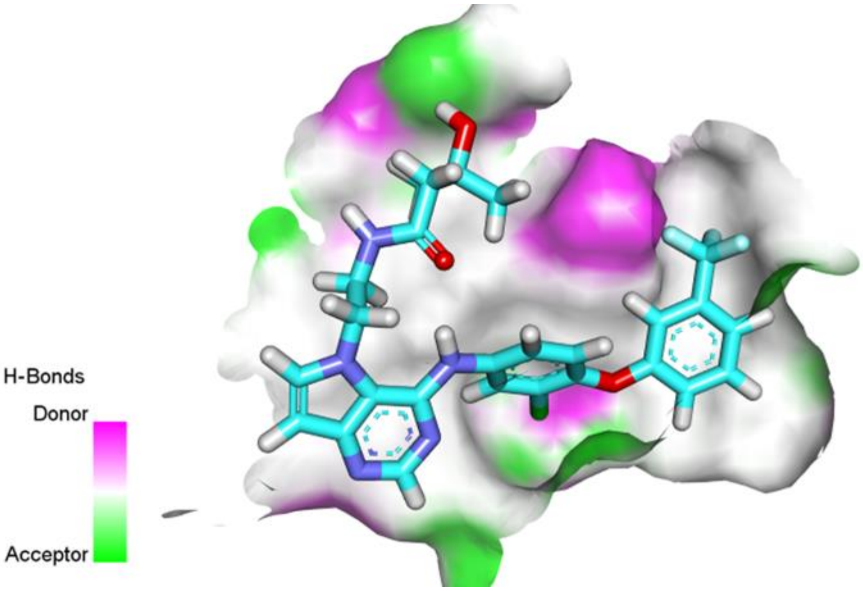

Mapping surface showing compound 12b occupying the active pocket of EGFR^{T790M}.

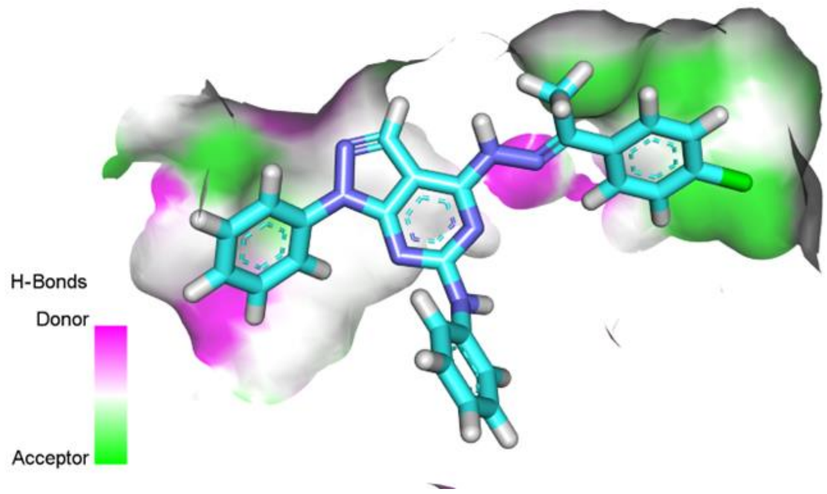

The expected ADMET study

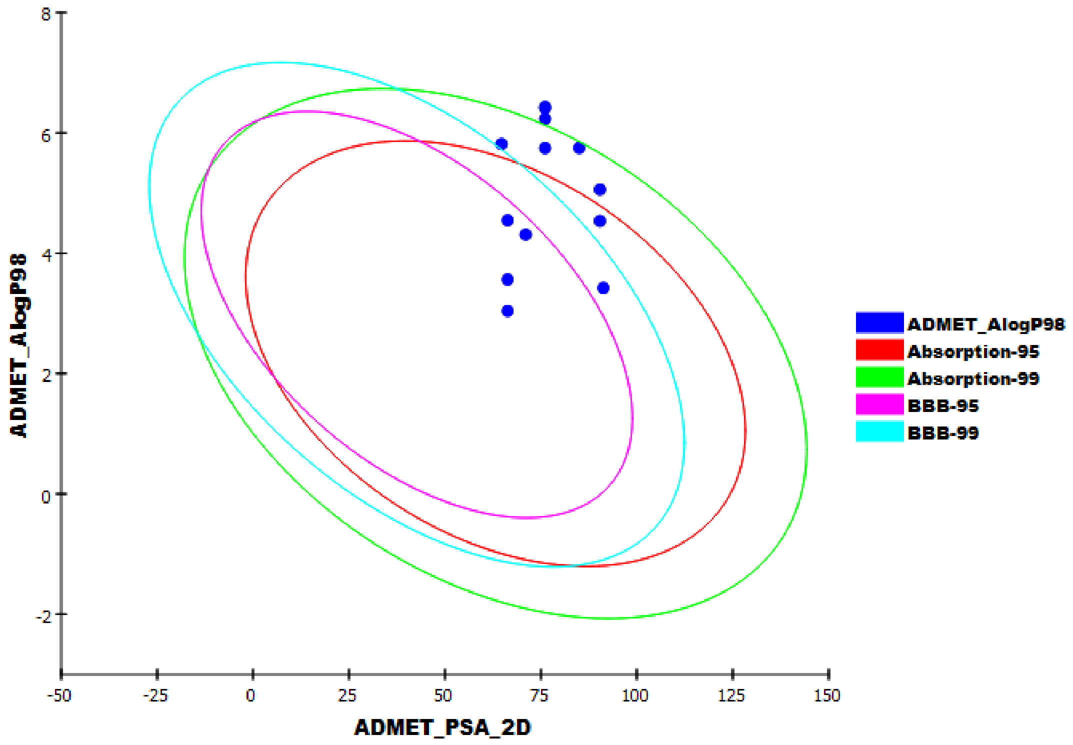

Molecular Dynamic Simulation:

To prepare the protein-ligand complex, we utilized the input generator module in the CHARMM-GUI webserver to prepare the necessary files for molecular dynamic (MD) simulation (1–4). We used the TIP3P water model with a padding of 10 Å to solvate the system in a cubic box then the system was neutralized by adding Na^+ and Cl^- ions to reach the physiological concentration of 0.154 M. CHAMM36m force field was used to parameterize the protein while CHARMM general force field (CGenFF) tool implemented in CHARMM-GUI was utilized to parameterize the ligand. GROMACS 2021 (5) was utilized as an MD engine to perform the simulation with periodic boundary conditions (PBC). First, a minimization step was conducted to remove any clashes between atoms using the steepest descent algorithm. The minimization stopped when the maximum force reached less than 100 $KJ.mol^{-1}.nm^{-1}$. Two subsequent equilibration steps were performed starting with a constant number of atoms, constant volume, and constant temperature (NVT) ensemble followed by a constant number of atoms, constant pressure, and constant temperature (NPT) ensemble. The temperature was set to 310 K and was maintained by V-rescale algorithm (6). While the pressure was set to 1 atmospheric pressure and was maintained through Berendsen barostat. Finally, a production run of 100 ns in NVT ensemble was performed. In each step, the bond length of hydrogen-bonded atoms was constrained using LINear Constraint Solver (LINCS) algorithm (7). The calculation of electrostatics was performed using Particle Mesh Ewald (PME) (8) algorithm with a cutoff of 1.2 nm. Leap-frog algorithm was used to integrate the Newtonian equations of motions with a time step of 1 femtosecond for the equilibration steps and 2 femtoseconds for the production step. The production run was saved every 100 picoseconds with a total of 1000 frames for each system. Before analyzing the trajectory, the PBC was removed. The analysis of the production trajectory was performed using VMD TK scripts (9). Root mean square deviation (RMSD), root mean square fluctuation (RMSF), solvent accessible surface area (SASA), radius of gyration (RoG), and the number of hydrogen bonds were calculated. Moreover, the distance between the center of mass of the protein and the center of mass of the ligand were calculated. Afterwards, the trajectory was clustered using TTClust (10) to get a representative frame for each cluster. First, backbone alignment was performed before determining the optimum number of clusters using the elbow method. For each representative frame, protein-ligand interaction profiler (PLIP) was used to detect the number and types of interactions (11).

References:

1. Brooks BR, Brooks III CL, Mackerell Jr AD, Nilsson L, Petrella RJ, Roux B, et al. CHARMM: the biomolecular simulation program. J Comput Chem. 2009;30(10):1545.

2. Jo S, Cheng X, Islam SM, Huang L, Rui H, Zhu A, et al. Chapter Eight - CHARMM-GUI PDB Manipulator for Advanced Modeling and Simulations of Proteins Containing Nonstandard Residues. In: Karabencheva-Christova T, editor. Biomolecular Modelling and Simulations [Internet]. Academic Press; 2014. p. 235–65. (Advances in Protein Chemistry and Structural Biology; vol. 96). Available from: https://www.sciencedirect.com/science/article/pii/S1876162314000030

3. Jo S, Kim T, Iyer VG, Im W. CHARMM-GUI: A web-based graphical user interface for CHARMM. J Comput Chem [Internet]. 2008;29(11):1859–65. Available from: https://onlinelibrary.wiley.com/doi/abs/10.1002/jcc.20945

4. Lee J, Cheng X, Swails JM, Yeom MS, Eastman PK, Lemkul JA, et al. CHARMM-GUI input generator for NAMD, GROMACS, AMBER, OpenMM, and CHARMM/OpenMM simulations using the CHARMM36 additive force field. J Chem Theory Comput. 2016;12(1):405–13.

5. Abraham MJ, Murtola T, Schulz R, Páll S, Smith JC, Hess B, et al. GROMACS: High performance molecular simulations through multi-level parallelism from laptops to supercomputers. SoftwareX. 2015;1:19–25.

6. Bussi G, Donadio D, Parrinello M. Canonical sampling through velocity rescaling. J Chem Phys. 2007;126(1):14101.

7. Hess B, Bekker H, Berendsen HJC, Fraaije JGEM. LINCS: a linear constraint solver for molecular simulations. J Comput Chem. 1997;18(12):1463–72.

8. Essmann U, Perera L, Berkowitz ML, Darden T, Lee H, Pedersen LG. A smooth particle mesh Ewald method. J Chem Phys. 1995;103(19):8577–93.

9. Humphrey W, Dalke A, Schulten K. VMD: visual molecular dynamics. J Mol Graph. 1996;14(1):33–8.

10. Tubiana T, Carvaillo J-C, Boulard Y, Bressanelli S. TTClust: a versatile molecular simulation trajectory clustering program with graphical summaries. J Chem Inf Model. 2018;58(11):2178–82.

11. Salentin S, Schreiber S, Haupt VJ, Adasme MF, Schroeder M. PLIP: fully automated protein–ligand interaction profiler. Nucleic Acids Res [Internet]. 2015 Jul 1;43(W1):W443–7. Available from: https://doi.org/10.1093/nar/gkv315

Best-fit values	
LogIC50	1.809
HillSlope	-0.4567
IC50	64.42

7a **Cl3** A549

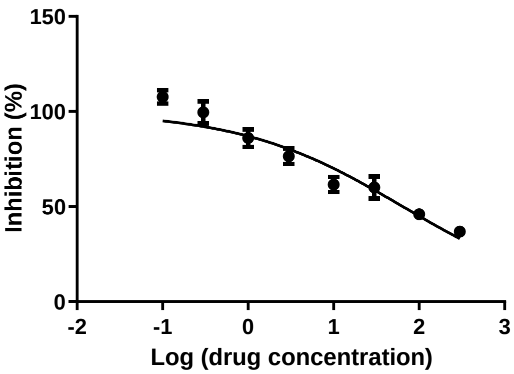

Best-fit values	
LogIC50	1.224
HillSlope	-0.3605
IC50	16.75

8 **Cl2** A549

IC_{50} = 16.75
R^2 = 0.8354

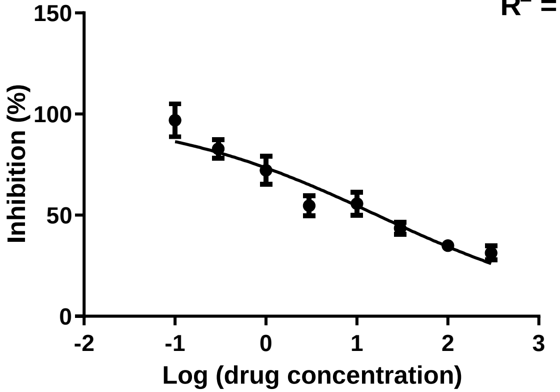

Best-fit values	
LogIC50	1.879
HillSlope	-0.3813
IC50	75.71

11b **HH3** A549

IC_{50} = 75.71
R^2 = 0.8801

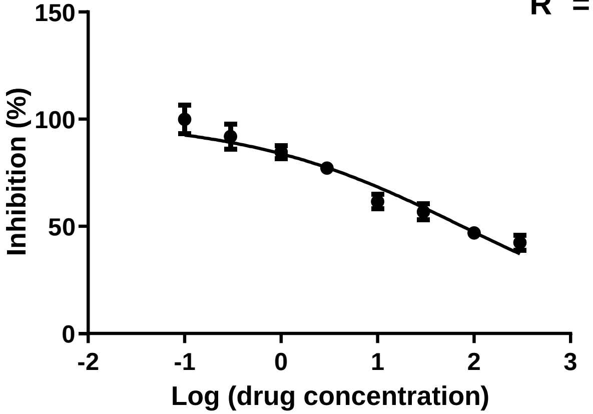

Best-fit values	
LogIC50	1.459
HillSlope	-0.4080
IC50	28.79

12c **HH7** A549

IC_{50} = 28.79
R^2 = 0.9398

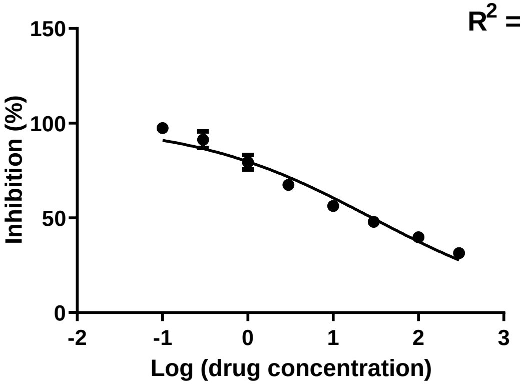

Best-fit values	
LogIC50	1.829
HillSlope	-0.4700
IC50	67.38

7b **Cl9**
A549

IC_{50} = 67.38
R^2 = 0.8800

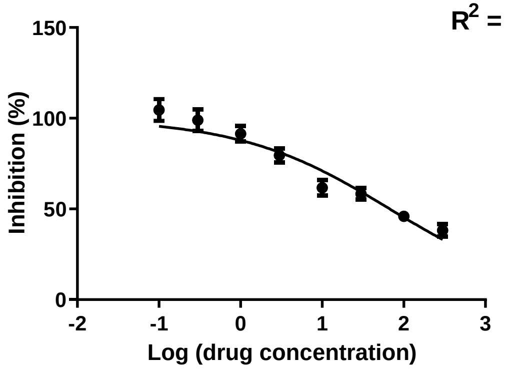

Best-fit values	
LogIC50	1.195
HillSlope	-0.3765
IC50	15.68

10 **B22**
A549

IC_{50} = 15.68
R^2 = 0.8797

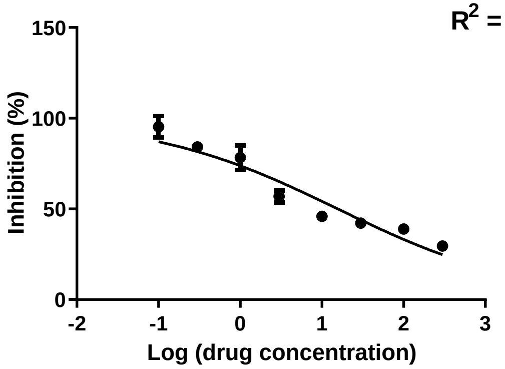

Best-fit values	
LogIC50	1.137
HillSlope	-0.3395
IC50	13.72

12a **HH1**
A549

IC_{50} = 13.72
R^2 = 0.9085

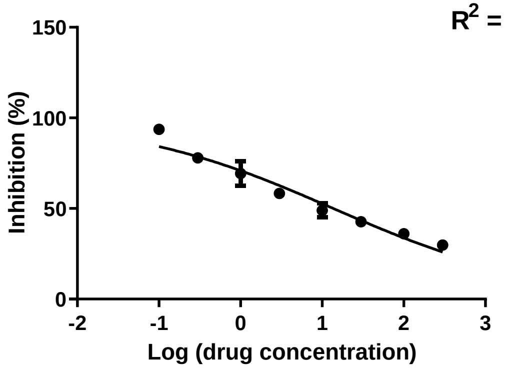

Best-fit values	
LogIC50	1.635
HillSlope	-0.4011
IC50	43.12

13a **B19**
A549

IC_{50} = 43.12
R^2 = 0.9121

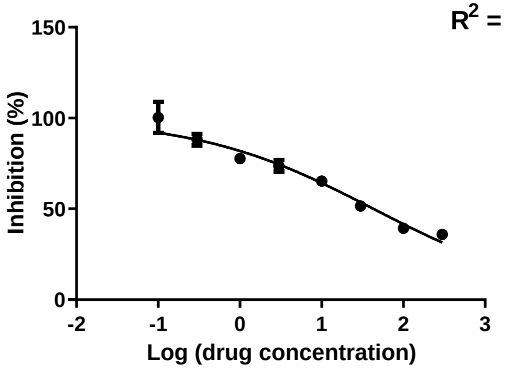

Best-fit values	
LogIC50	1.463
HillSlope	-0.3708
IC50	29.07

9 **Cl1** A549

IC_{50} = 29.07

R^2 = 0.9189

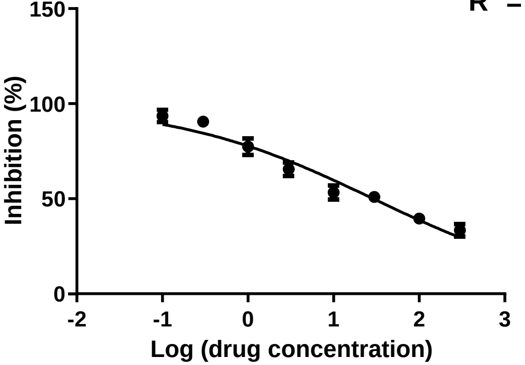

Best-fit values	
LogIC50	1.678
HillSlope	-0.4514
IC50	47.67

11a **HH4** A549

IC_{50} = 47.67

R^2 = 0.8710

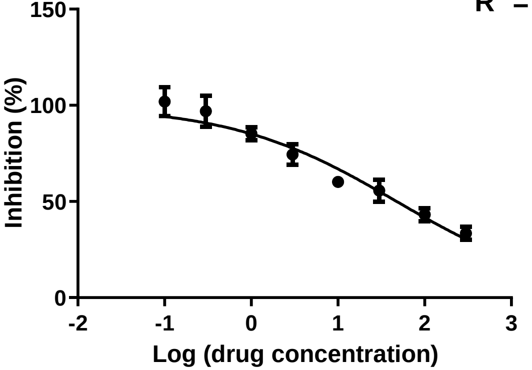

Best-fit values	
LogIC50	0.9146
HillSlope	-0.3430
IC50	8.216

12b **HH6** A549

IC_{50} = 8.216

R^2 = 0.7844

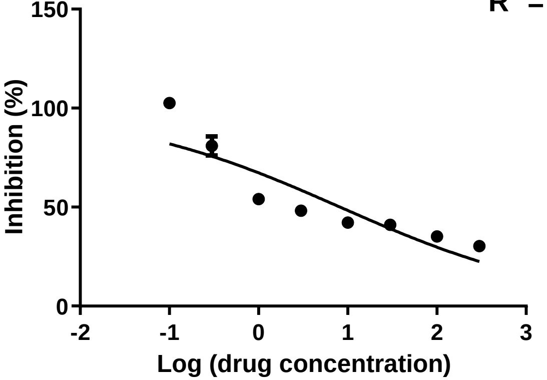

Best-fit values	
LogIC50	1.186
HillSlope	-0.3921
IC50	15.36

13b **B20** A549

IC_{50} = 15.36

R^2 = 0.8341

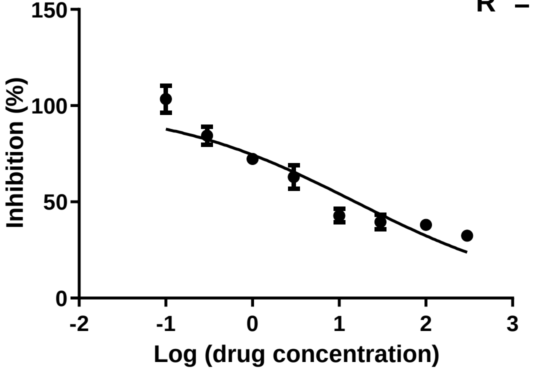

7a

Cl3
HCT-116

Best-fit values	
LogIC50	1.472
HillSlope	-0.4339
IC50	29.62

IC_{50} = 29.62
R^2 = 0.8949

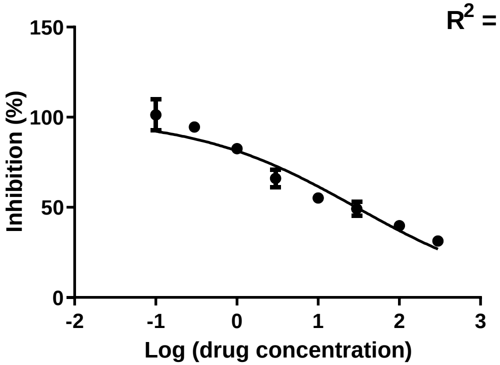

8

Cl2
HCT-116

Best-fit values	
LogIC50	1.383
HillSlope	-0.4231
IC50	24.16

IC_{50} = 24.16
R^2 = 0.8728

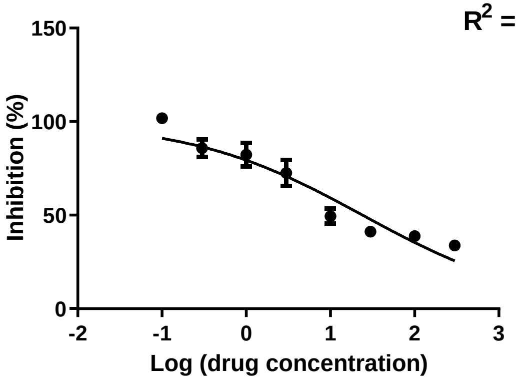

11b

HH3
HCT-116

Best-fit values	
LogIC50	1.876
HillSlope	-0.5652
IC50	75.11

IC_{50} = 75.11
R^2 = 0.7839

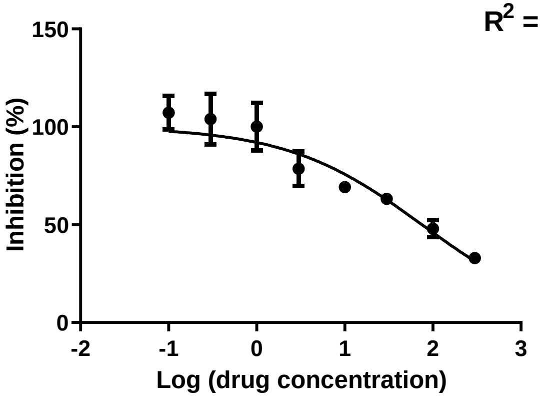

12c

HH7
HCT-116

Best-fit values	
LogIC50	1.458
HillSlope	-0.4467
IC50	28.69

IC_{50} = 28.69
R^2 = 0.8710

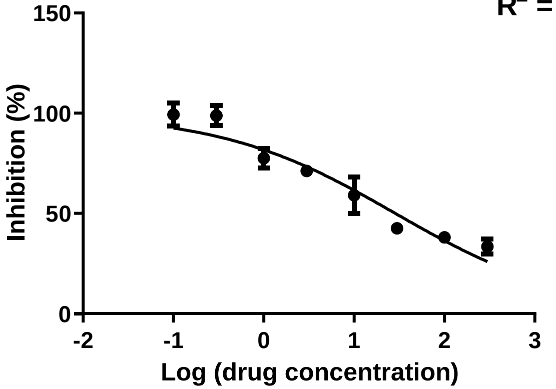

7b

Cl9
HCT-116

Best-fit values	
LogIC50	1.498
HillSlope	-0.4549
IC50	31.49

IC_{50} = 31.49
R^2 = 0.8729

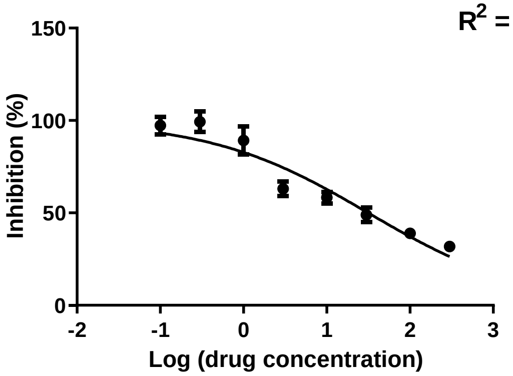

10

B22
HCT-116

Best-fit values	
LogIC50	1.274
HillSlope	-0.3276
IC50	18.78

IC_{50} = 18.78
R^2 = 0.9009

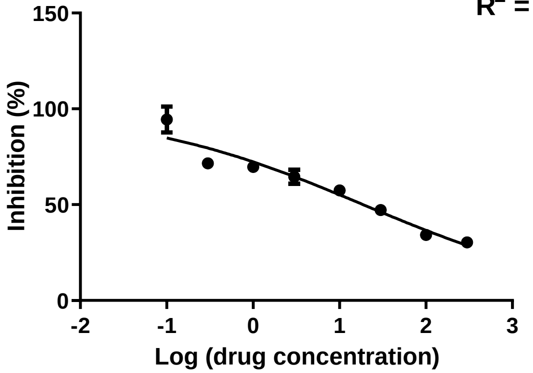

12a

HH1
HCT-116

Best-fit values	
LogIC50	1.368
HillSlope	-0.4063
IC50	23.33

IC_{50} = 23.33
R^2 = 0.9246

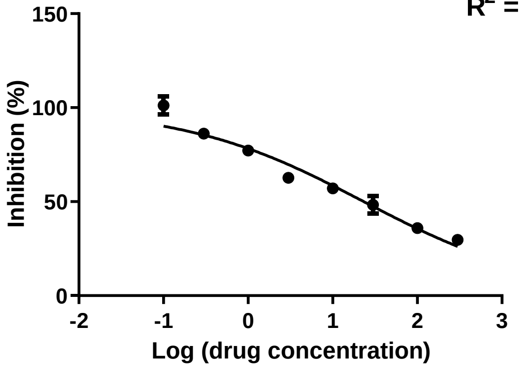

13a

B19
HCT-116

Best-fit values	
LogIC50	1.432
HillSlope	-0.4156
IC50	27.03

IC_{50} = 27.03
R^2 = 0.9279

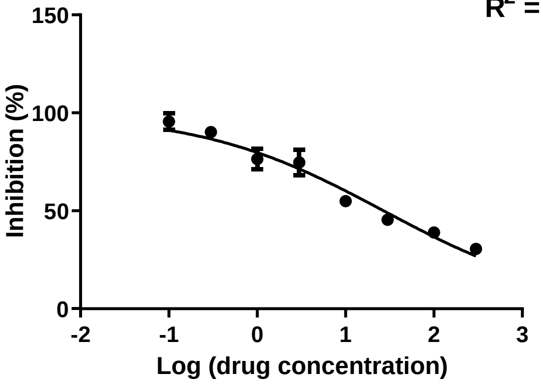

9

Cl1
HCT-116

IC_{50} = 39.39
R^2 = 0.9532

Best-fit values	
LogIC50	1.595
HillSlope	-0.3946
IC50	39.39

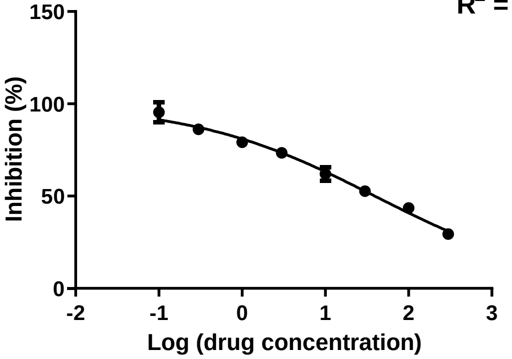

11a

HH4
HCT-116

IC_{50} = 46.18
R^2 = 0.8919

Best-fit values	
LogIC50	1.664
HillSlope	-0.4760
IC50	46.18

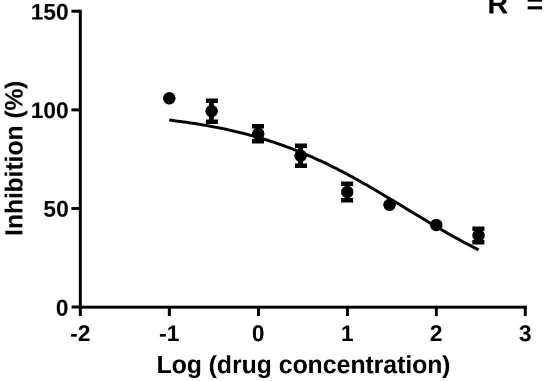

12b

HH6
HCT-116

IC_{50} = 19.56
R^2 = 0.8914

Best-fit values	
LogIC50	1.291
HillSlope	-0.3805
IC50	19.56

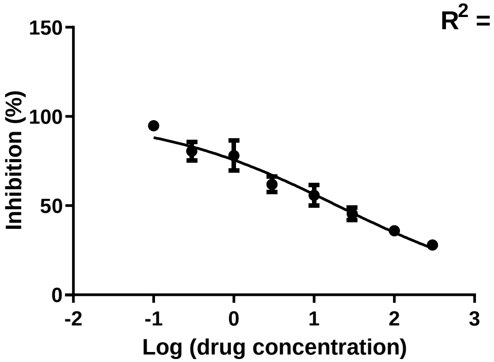

13b

B20
HCT-116

IC_{50} = 36.44
R^2 = 0.9234

Best-fit values	
LogIC50	1.562
HillSlope	-0.3703
IC50	36.44

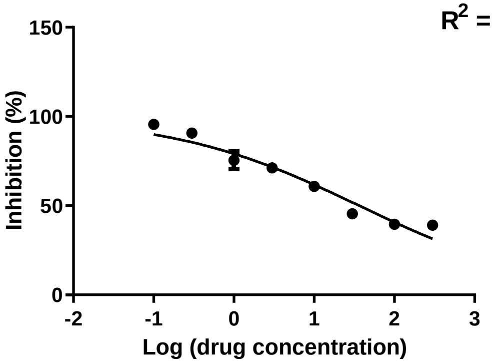

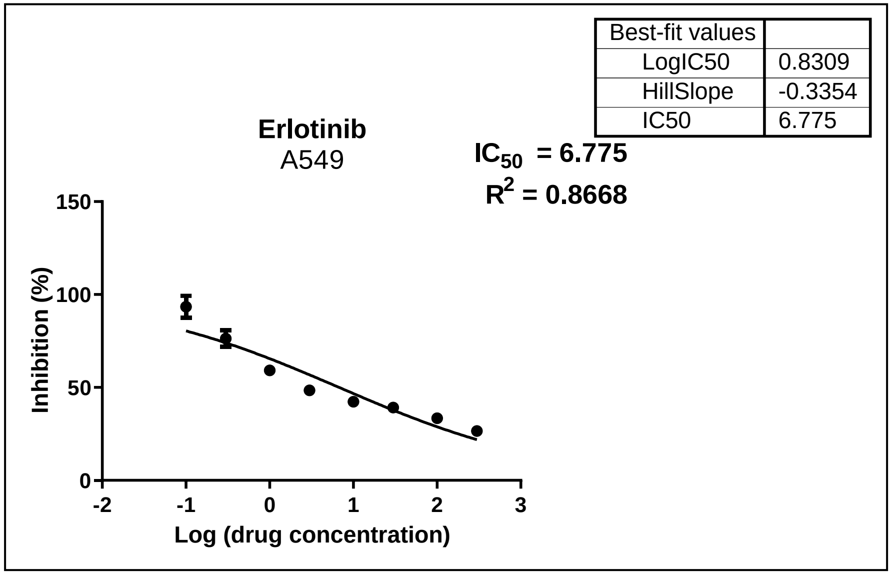
Best-fit values
LogIC50 0.8309
HillSlope -0.3354
IC50 6.775
Erlotinib
A549
IC50 = 6.775
R2 = 0.8668
Inhibition (%)
Log (drug concentration)

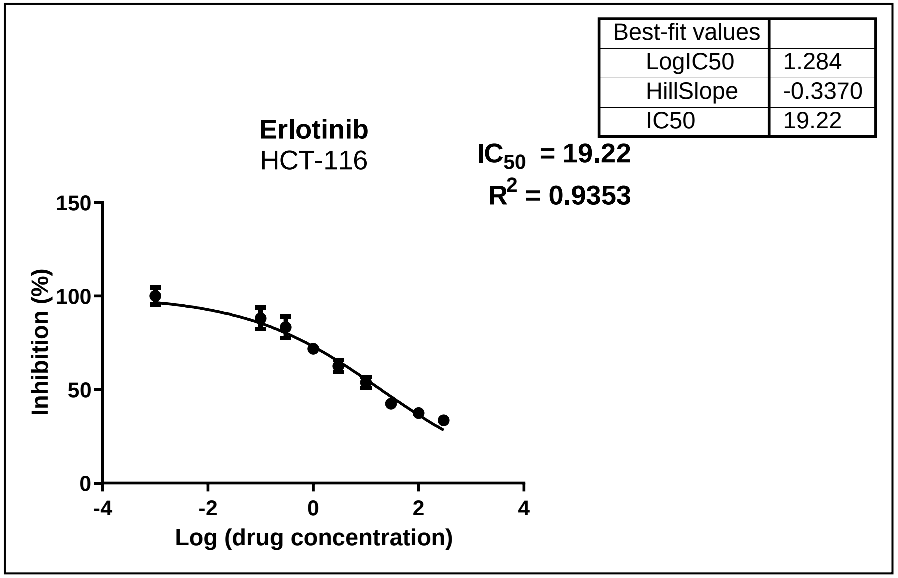
Best-fit values
LogIC50 1.284
HillSlope -0.3370
IC50 19.22
Erlotinib
HCT-116
IC50 = 19.22
R2 = 0.9353
Inhibition (%)
Log (drug concentration)

Best-fit values	
LogIC50	1.415
HillSlope	-0.3385
IC50	25.98

8 Cl2

IC_{50} = 25.98

R^2 = 0.8707

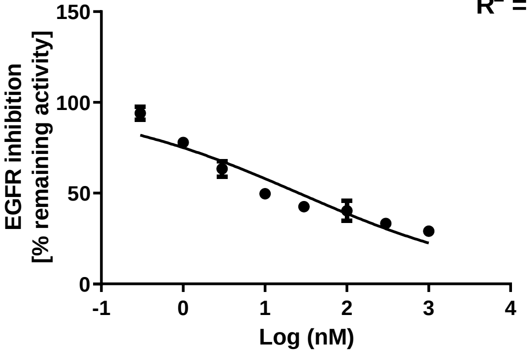

Best-fit values	
LogIC50	1.366
HillSlope	-0.3619
IC50	23.21

10 B22

IC_{50} = 23.21

R^2 = 0.9124

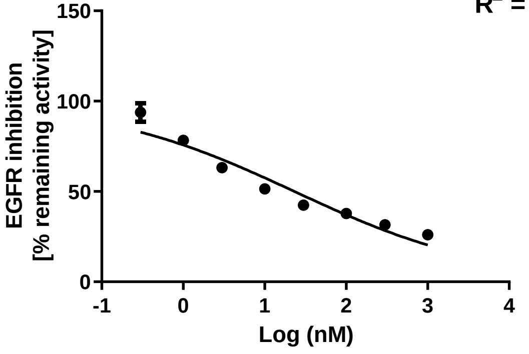

Best-fit values	
LogIC50	1.321
HillSlope	-0.3296
IC50	20.96

12a HH1

IC_{50} = 20.96

R^2 = 0.8843

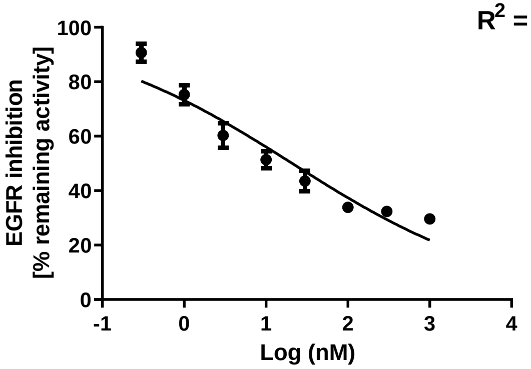

Best-fit values	
LogIC50	1.193
HillSlope	-0.3524
IC50	15.59

12b HH6

IC_{50} = 15.59

R^2 = 0.7840

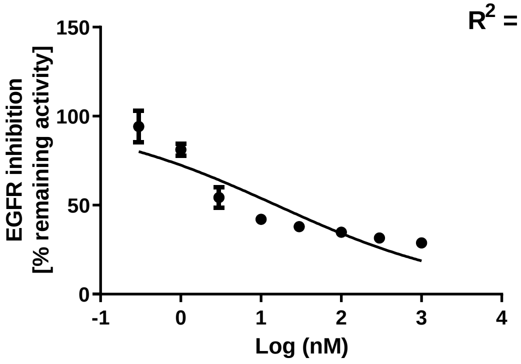

Best-fit values	
LogIC50	2.372
HillSlope	-0.3418
IC50	235.5

12b **HH6**

IC_{50} = 235.5
R^2 = 0.8643

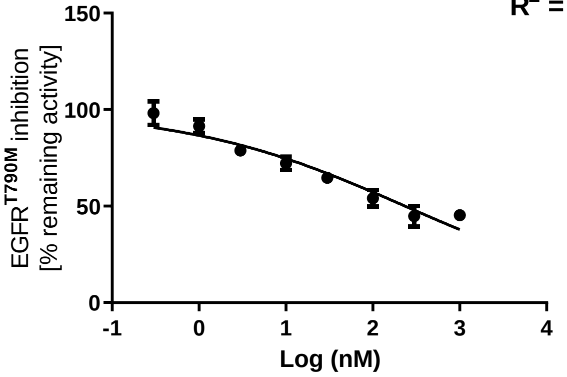

Best-fit values	
LogIC50	0.7552
HillSlope	-0.3123
IC50	5.691

Erlotinib

IC_{50} = 5.691
R^2 = 0.8027

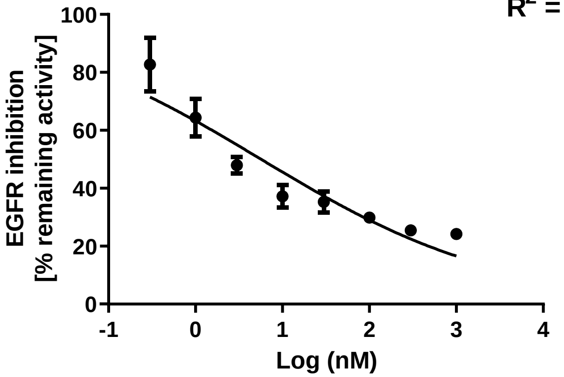

Best-fit values	
LogIC50	2.751
HillSlope	-0.4999
IC50	563.4

Erlotinib

IC_{50} = 563.4
R^2 = 0.8548

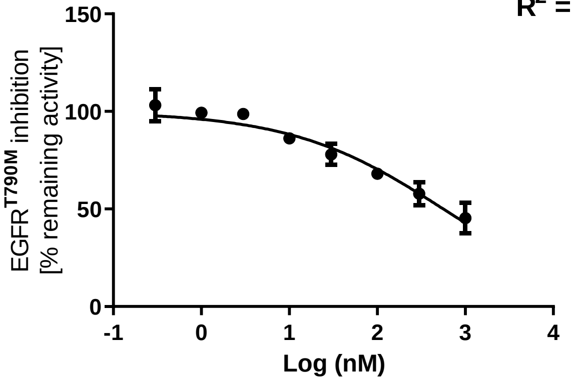

Compound 12b

Best-fit values	
LogIC50	1.593
HillSlope	-0.4504
IC50	39.15

HH6
WI-38

IC_{50} = 39.15
R^2 = 0.9445

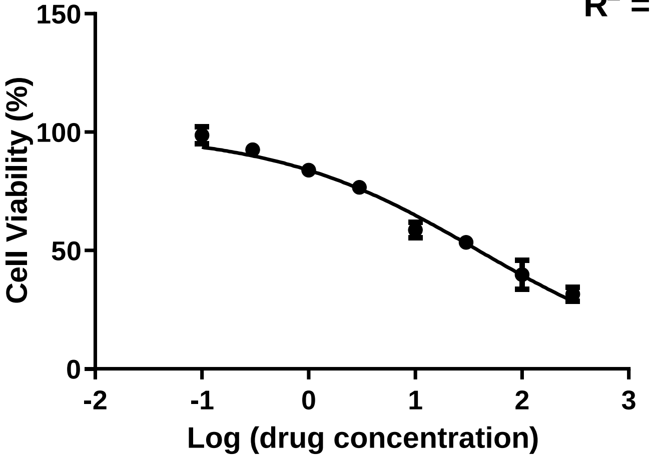

Best-fit values	
LogIC50	1.528
HillSlope	-0.4750
IC50	33.75

Erlotinib
WI-38

IC_{50} = 33.75
R^2 = 0.8865

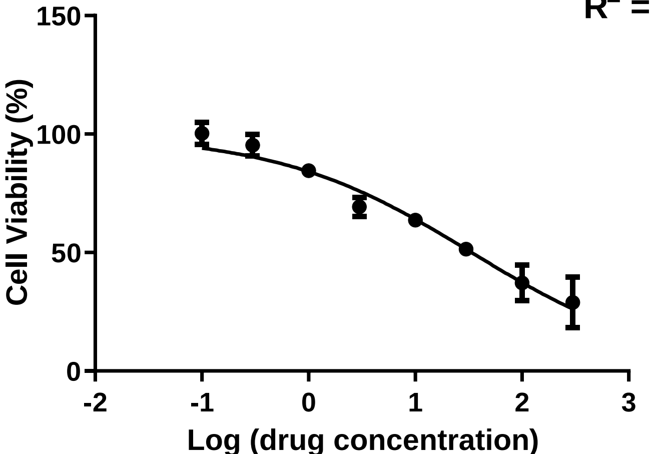

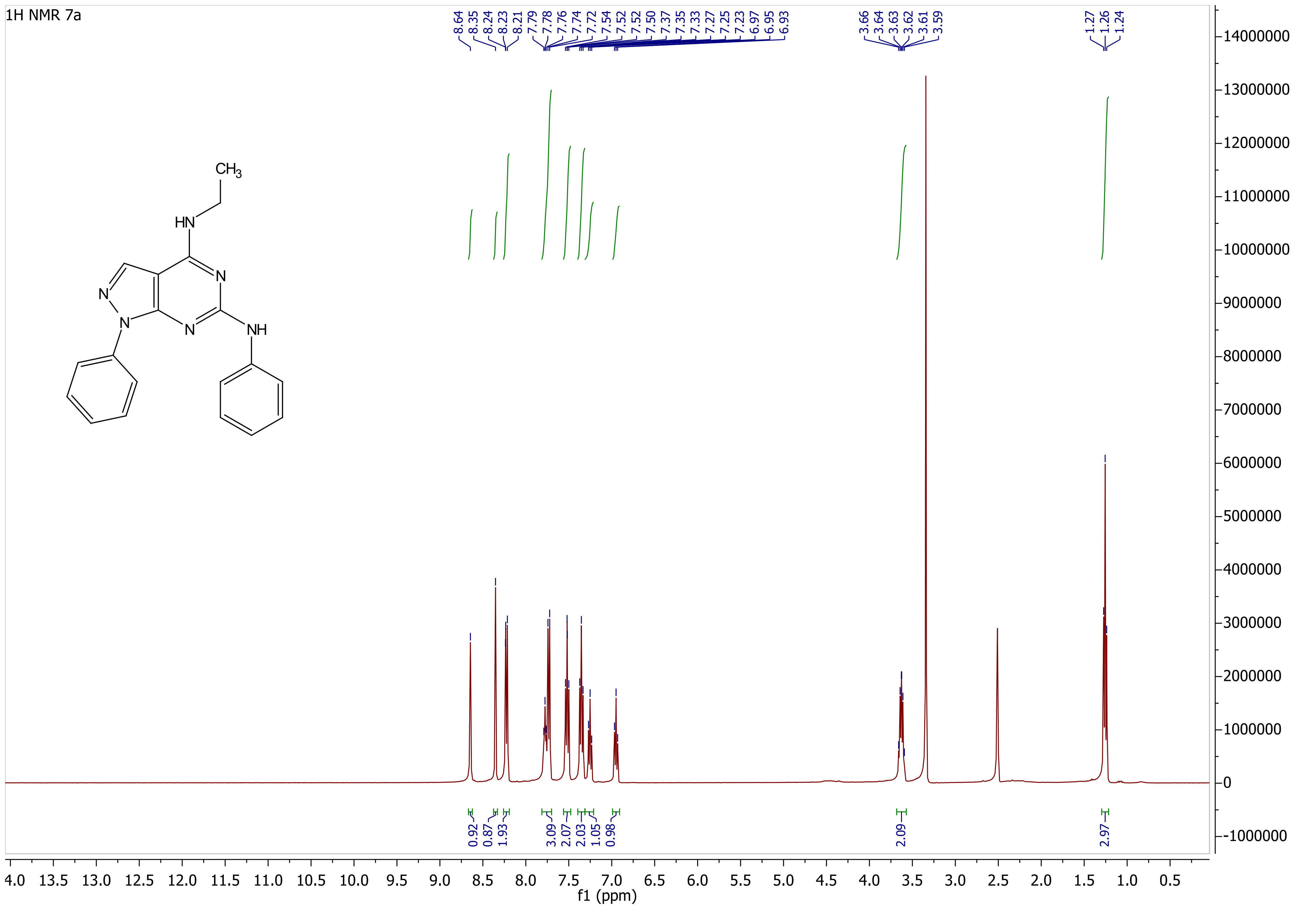
1H NMR 7a
8.64 8.35 8.24 8.23 8.21 7.79 7.78 7.76 7.74 7.72 7.54 7.52 7.52 7.50 7.37 7.35 7.33 7.27 7.25 7.23 6.97 6.95 6.93
3.66 3.64 3.63 3.62 3.61 3.59
1.27 1.26 1.24
CH3
HN
N
N
N
N
NH
0.92 0.87 1.93 3.09 2.07 2.03 1.05 0.98 2.09 2.97
14000000
13000000
12000000
11000000
10000000
9000000
8000000
7000000
6000000
5000000
4000000
3000000
2000000
1000000
0
-1000000
4.0 13.5 13.0 12.5 12.0 11.5 11.0 10.5 10.0 9.5 9.0 8.5 8.0 7.5 7.0 6.5 6.0 5.5 5.0 4.5 4.0 3.5 3.0 2.5 2.0 1.5 1.0 0.5
f1 (ppm)

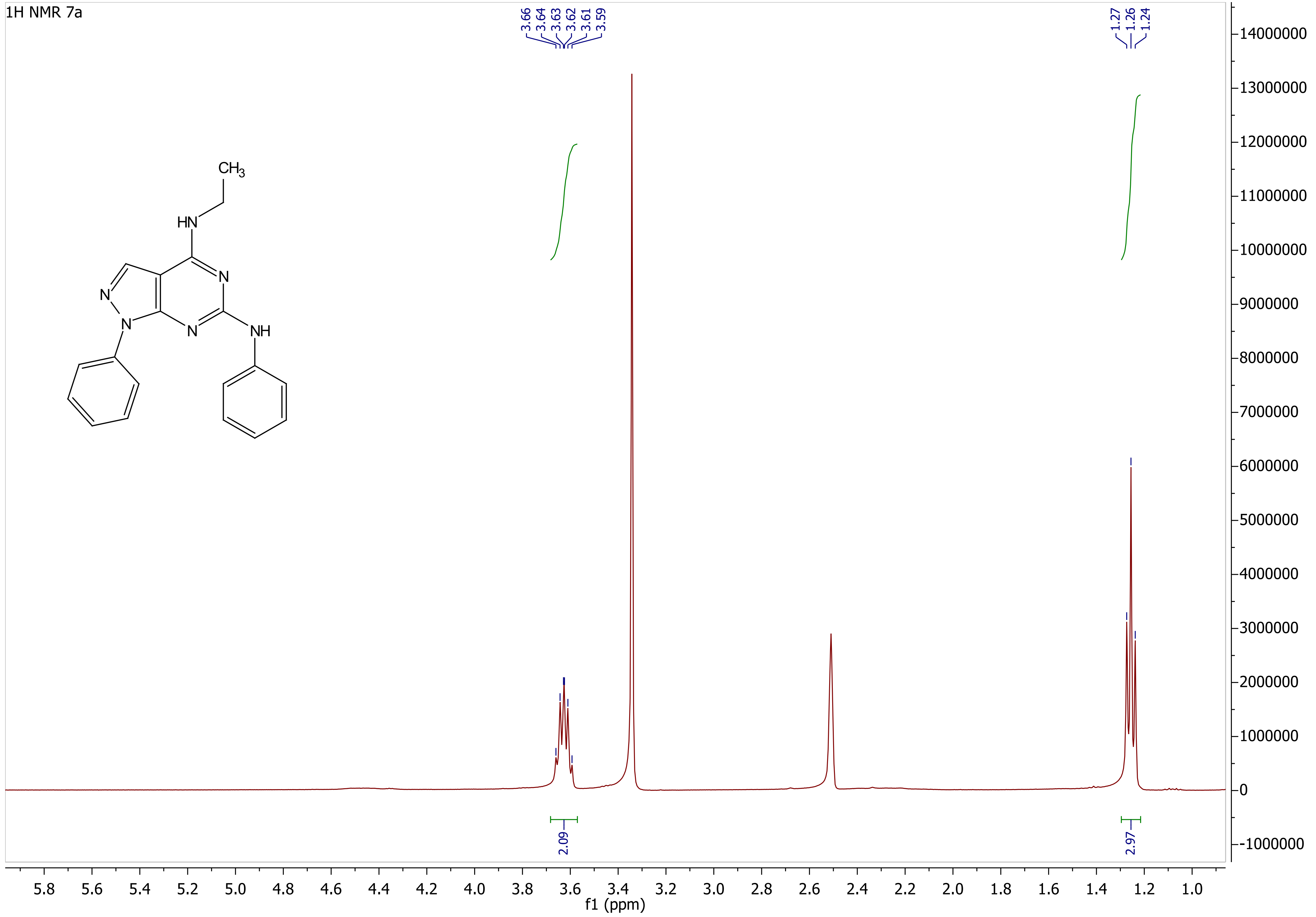
1H NMR 7a
3.66
3.64
3.63
3.62
3.61
3.59
1.27
1.26
1.24
CH3
HN
N
N
N
N
NH
2.09
2.97
14000000
13000000
12000000
11000000
10000000
9000000
8000000
7000000
6000000
5000000
4000000
3000000
2000000
1000000
0
-1000000
5.8 5.6 5.4 5.2 5.0 4.8 4.6 4.4 4.2 4.0 3.8 3.6 3.4 3.2 3.0 2.8 2.6 2.4 2.2 2.0 1.8 1.6 1.4 1.2 1.0
f1 (ppm)

1H NMR 7a

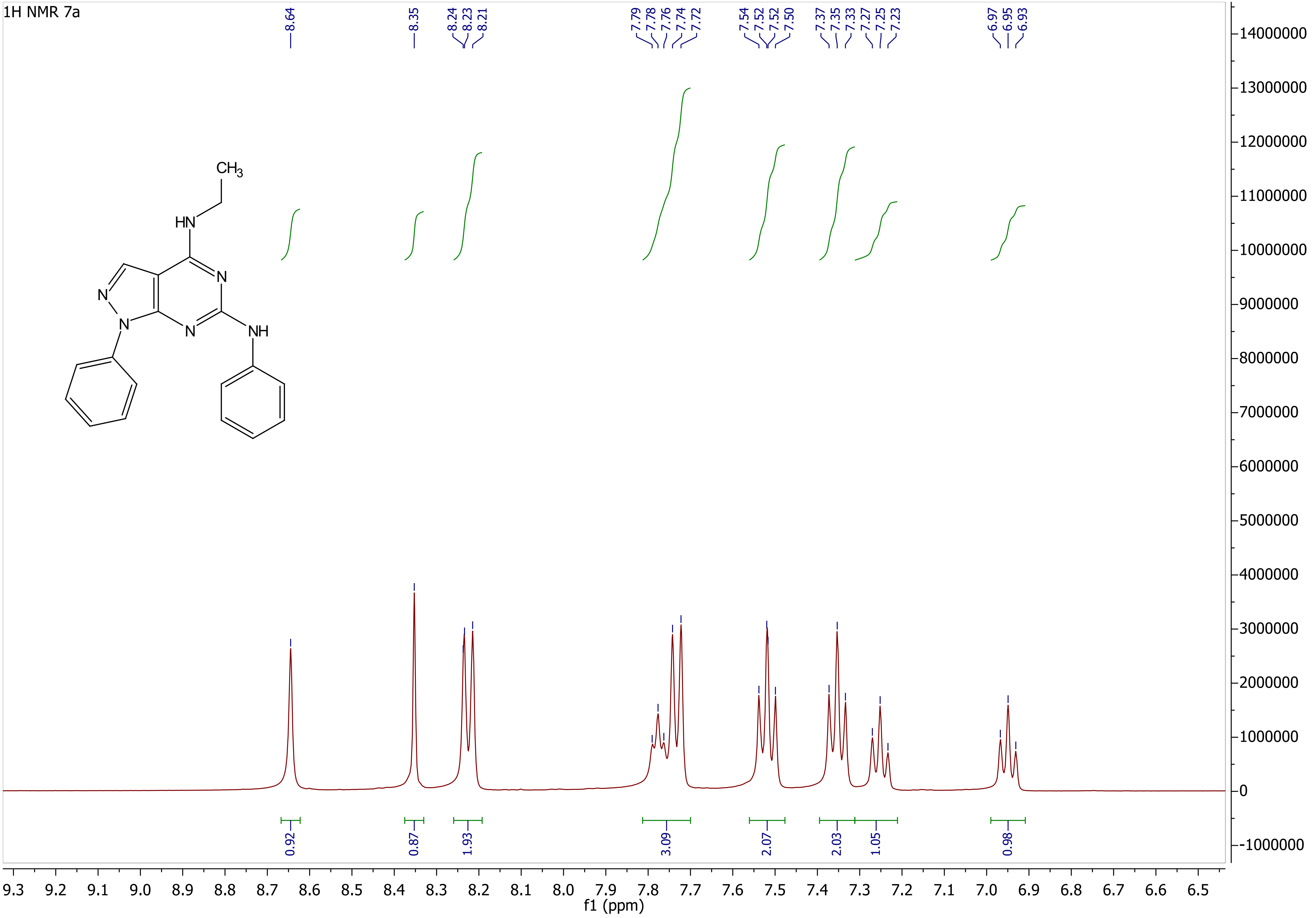
8.64
8.35
8.24
8.23
8.21
7.79
7.78
7.76
7.74
7.72
7.54
7.52
7.52
7.50
7.37
7.35
7.33
7.27
7.25
7.23
6.97
6.95
6.93
0.92
0.87
1.93
3.09
2.07
2.03
1.05
0.98
f1 (ppm)

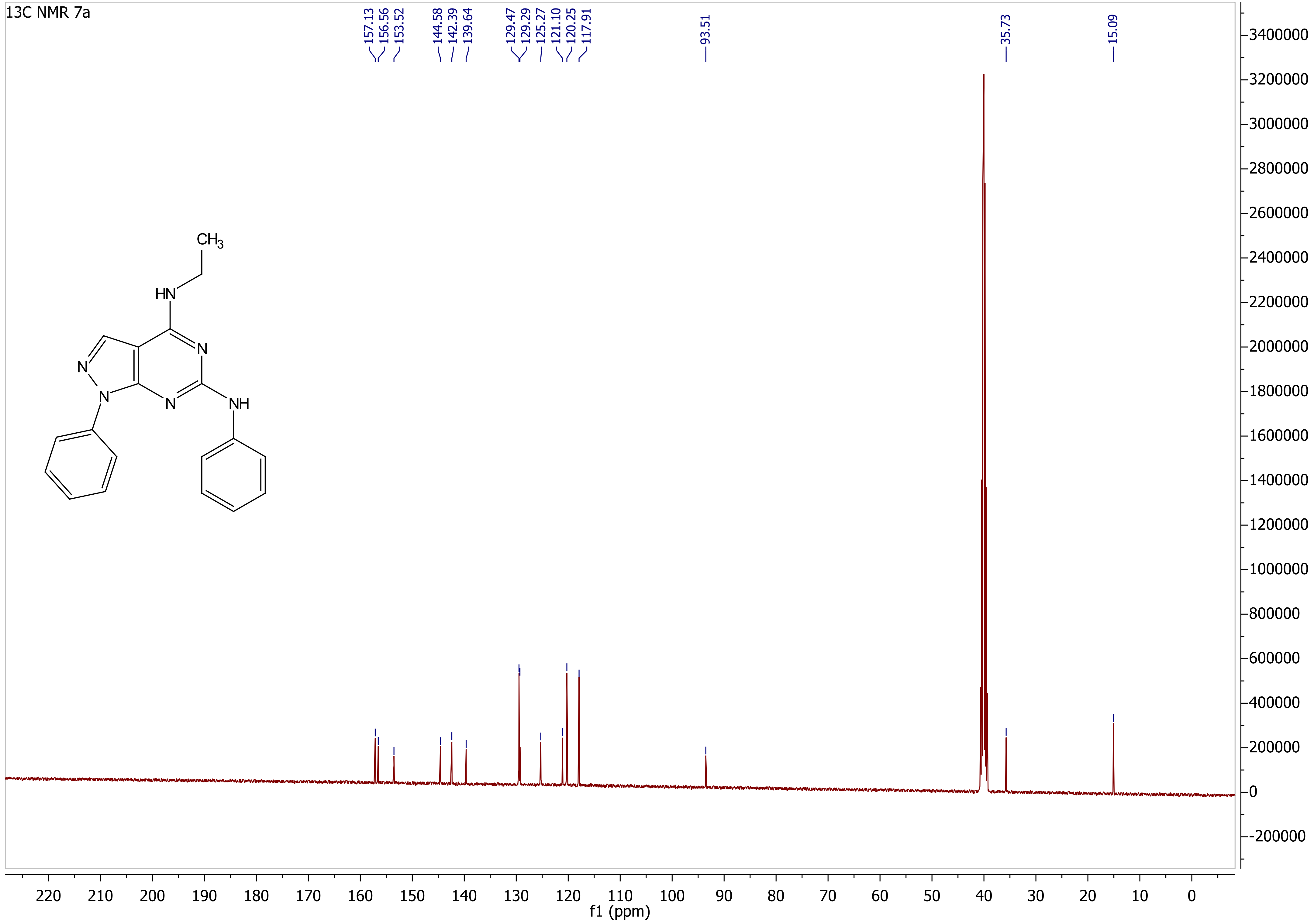
13C NMR 7a
157.13
156.56
153.52
144.58
142.39
139.64
129.47
129.29
125.27
121.10
120.25
117.91
93.51
35.73
15.09
CH3
HN
N
N
N
N
NH
f1 (ppm)
220 210 200 190 180 170 160 150 140 130 120 110 100 90 80 70 60 50 40 30 20 10 0
3400000 3200000 3000000 2800000 2600000 2400000 2200000 2000000 1800000 1600000 1400000 1200000 1000000 800000 600000 400000 200000 0 -200000

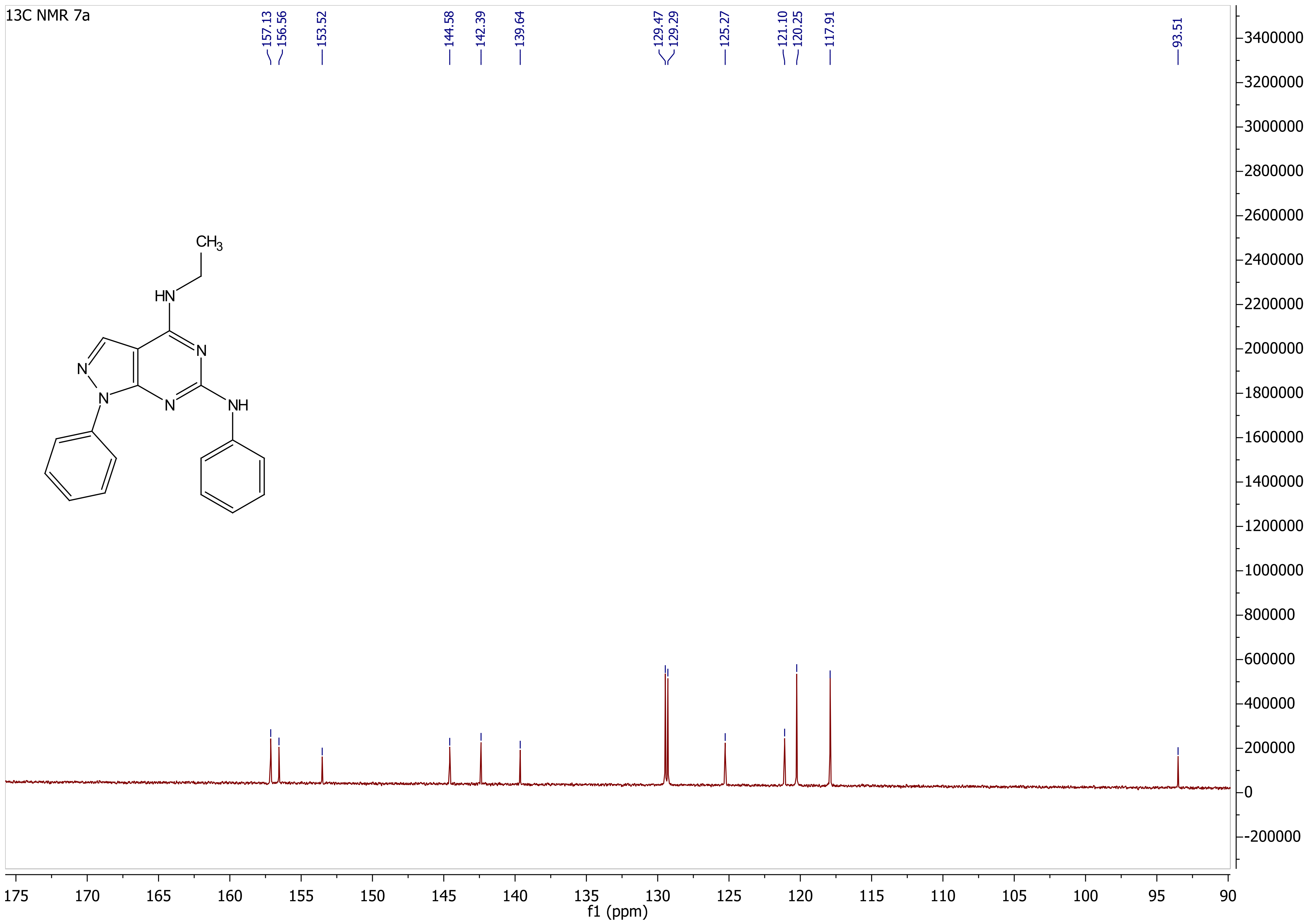
13C NMR 7a
157.13
156.56
153.52
144.58
142.39
139.64
129.47
129.29
125.27
121.10
120.25
117.91
93.51
CH3
HN
N
N
N
N
NH
f1 (ppm)
175
170
165
160
155
150
145
140
135
130
125
120
115
110
105
100
95
90
3400000
3200000
3000000
2800000
2600000
2400000
2200000
2000000
1800000
1600000
1400000
1200000
1000000
800000
600000
400000
200000
0
-200000

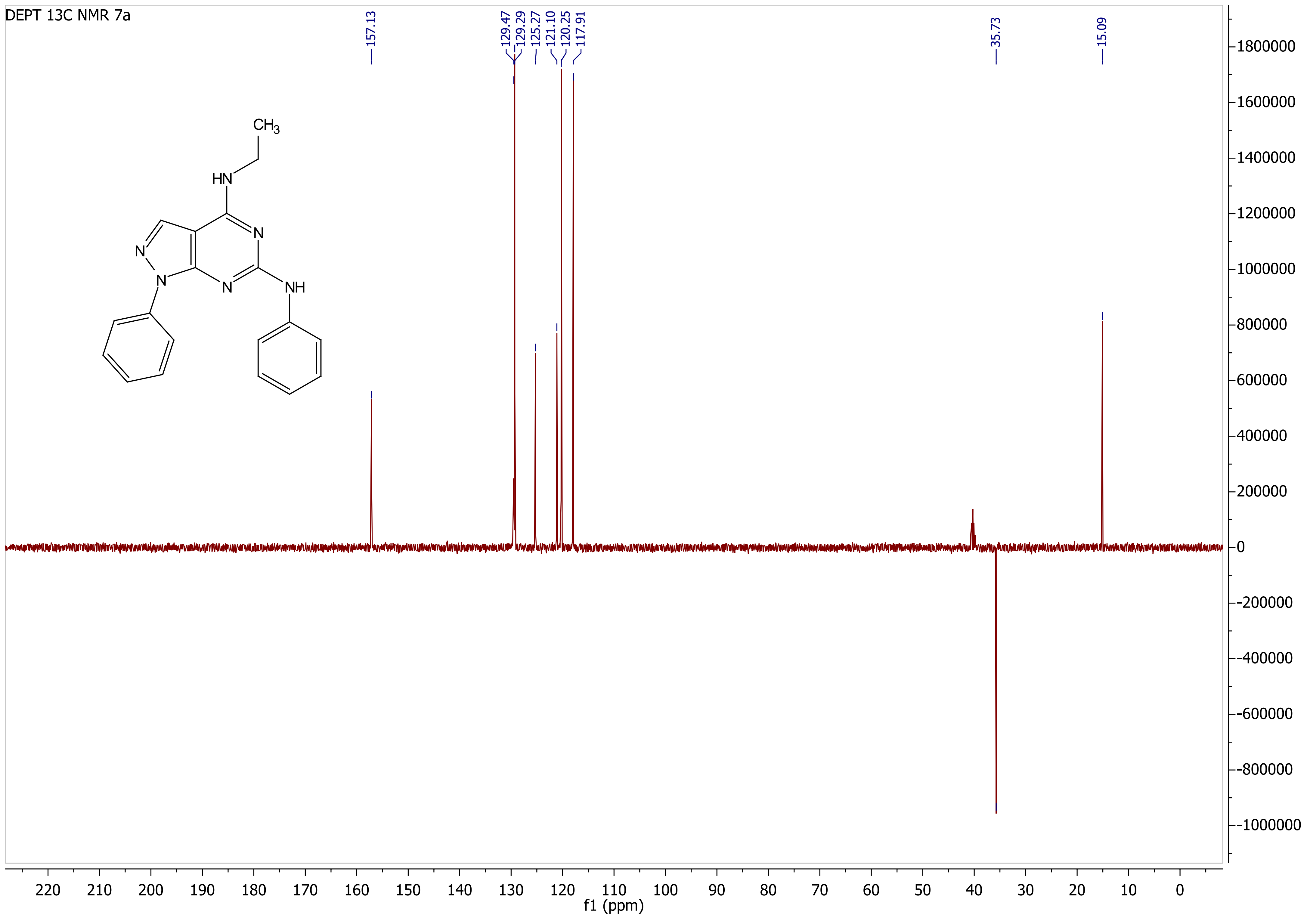
DEPT 13C NMR 7a
157.13
129.47
129.29
125.27
121.10
120.25
117.91
35.73
15.09
CH3
HN
N
N
N
N
NH
f1 (ppm)

DEPT 13C NMR 7a

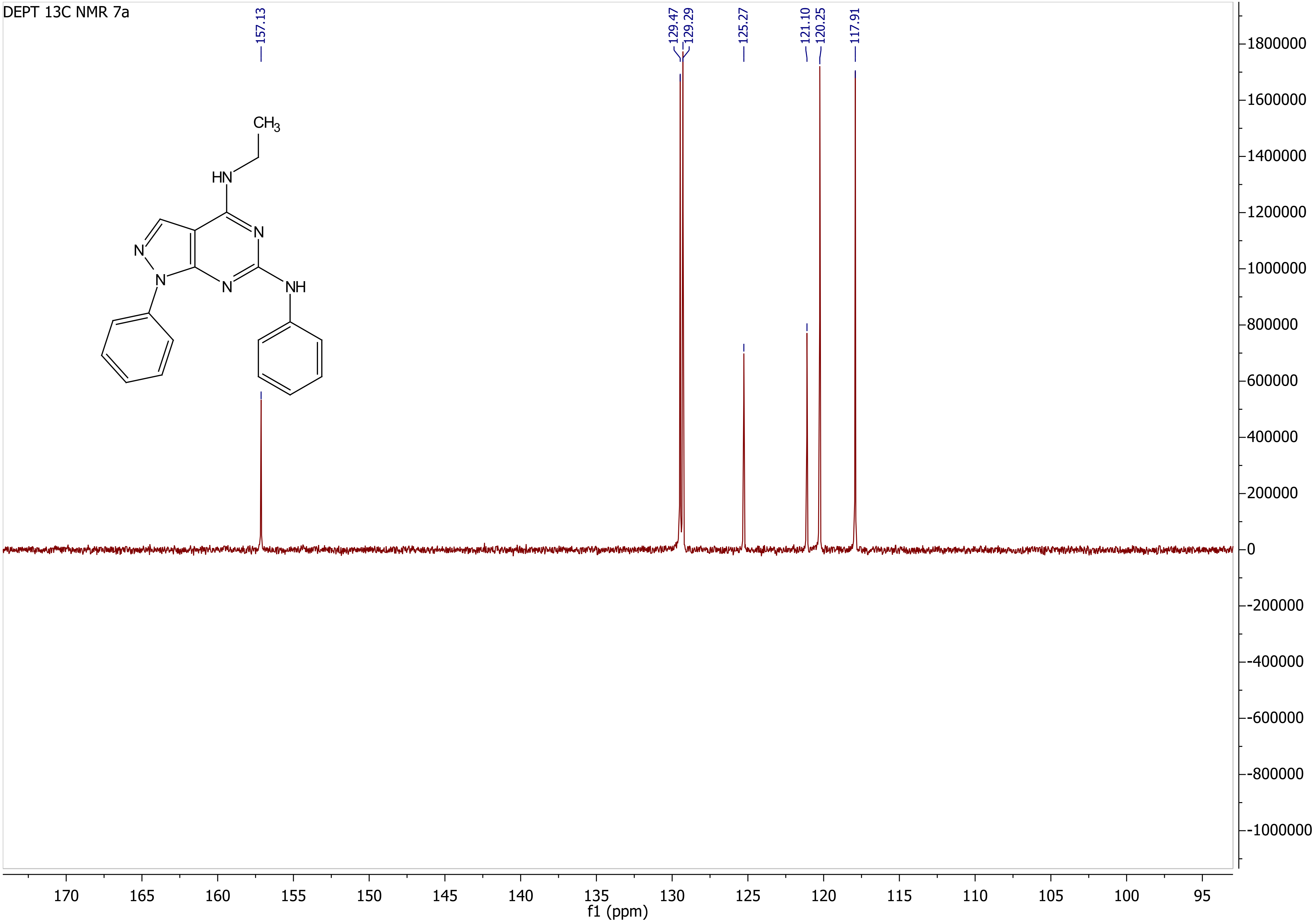

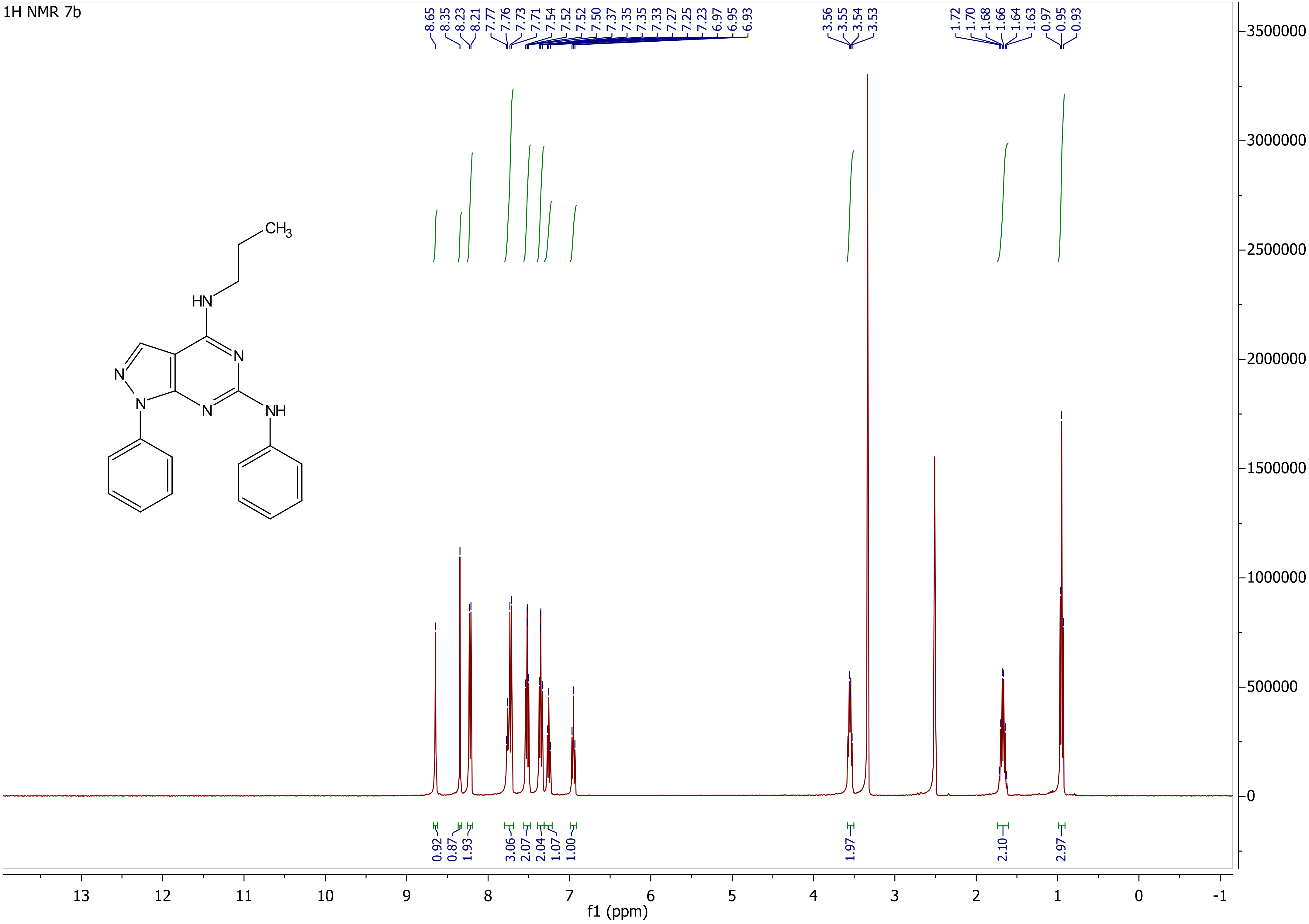
1H NMR 7b
8.65
8.35
8.23
8.21
7.77
7.76
7.73
7.71
7.54
7.52
7.52
7.50
7.37
7.35
7.35
7.33
7.27
7.25
7.23
6.97
6.95
6.93
3.56
3.55
3.54
3.53
1.72
1.70
1.68
1.66
1.64
1.63
0.97
0.95
0.93
CH3
HN
N
N
N
N
NH
0.92
0.87
1.93
3.06
2.07
2.04
1.07
1.00
1.97
2.10
2.97
3500000
3000000
2500000
2000000
1500000
1000000
500000
0
13
12
11
10
9
8
7
6
5
4
3
2
1
0
-1
f1 (ppm)

1H NMR 7b

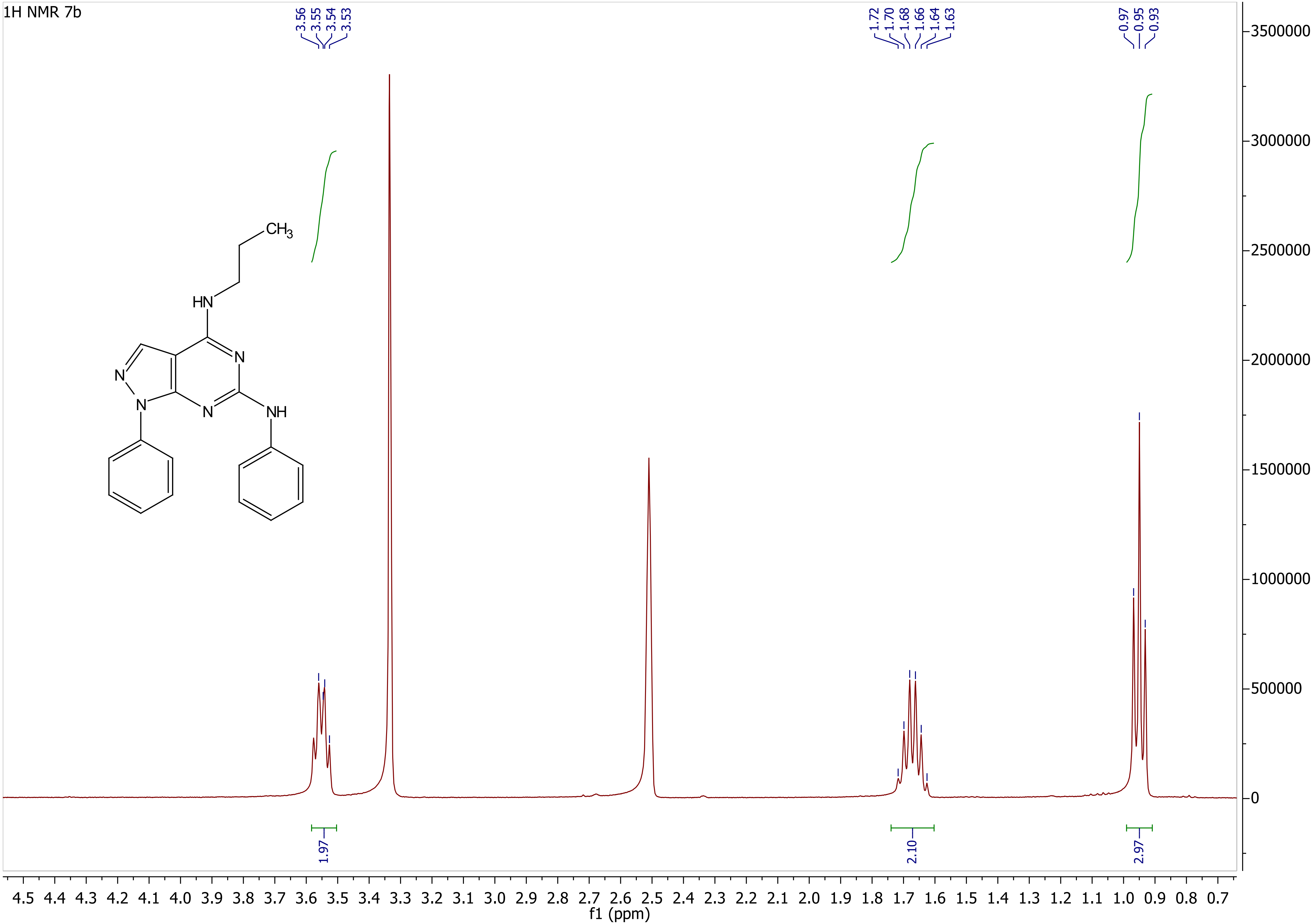

3.56
3.55
3.54
3.53
1.72
1.70
1.68
1.66
1.64
1.63
0.97
0.95
0.93
CH3
HN
N
N
N
N
NH
1.97
2.10
2.97
3500000
3000000
2500000
2000000
1500000
1000000
500000
0
4.5 4.4 4.3 4.2 4.1 4.0 3.9 3.8 3.7 3.6 3.5 3.4 3.3 3.2 3.1 3.0 2.9 2.8 2.7 2.6 2.5 2.4 2.3 2.2 2.1 2.0 1.9 1.8 1.7 1.6 1.5 1.4 1.3 1.2 1.1 1.0 0.9 0.8 0.7
f1 (ppm)

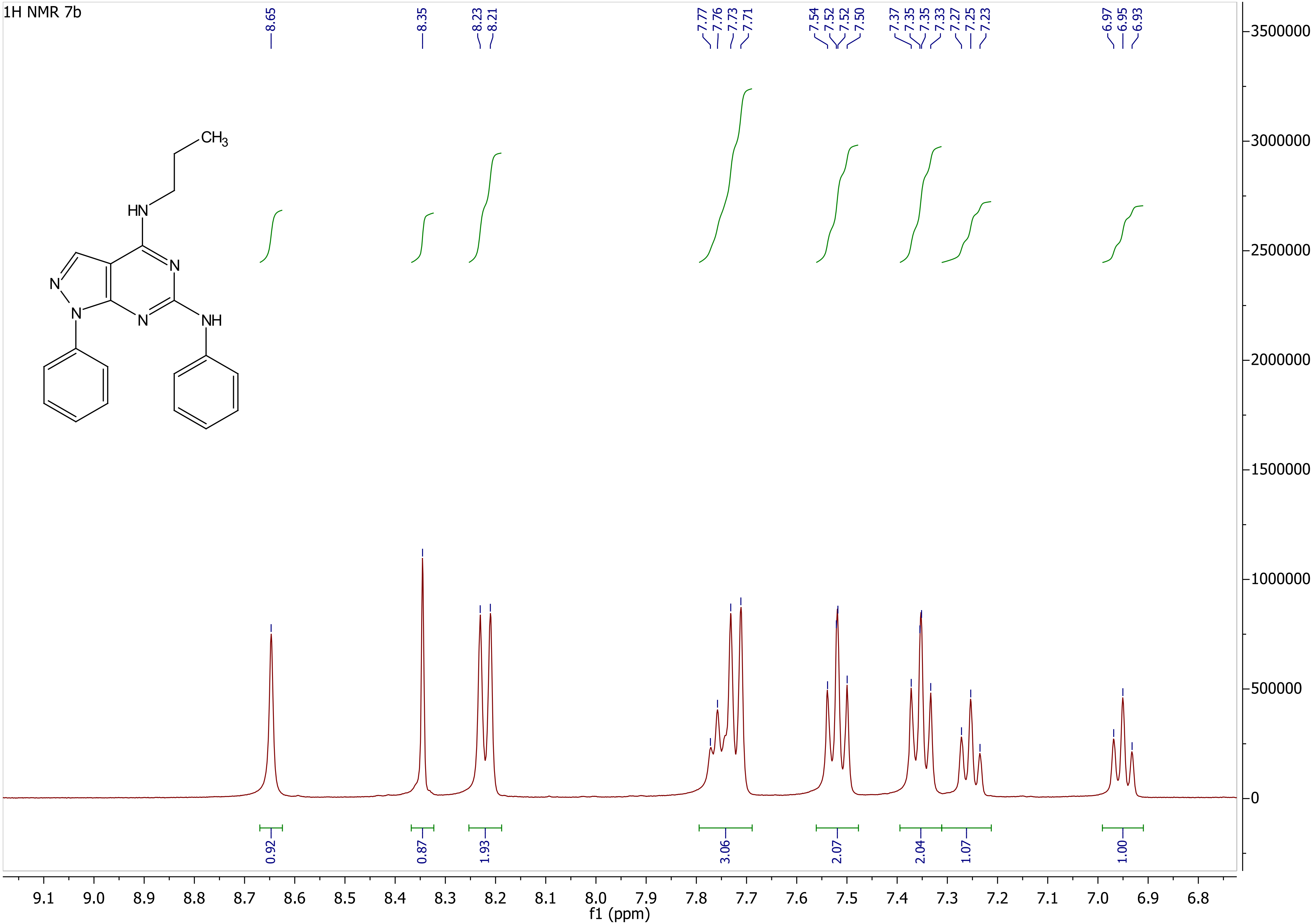

1H NMR 7b
8.65
8.35
8.23
8.21
7.77
7.76
7.73
7.71
7.54
7.52
7.52
7.50
7.37
7.35
7.35
7.33
7.27
7.25
7.23
6.97
6.95
6.93
CH3
HN
N
N
N
N
NH
3500000
3000000
2500000
2000000
1500000
1000000
500000
0
0.92
0.87
1.93
3.06
2.07
2.04
1.07
1.00
9.1 9.0 8.9 8.8 8.7 8.6 8.5 8.4 8.3 8.2 8.1 8.0 7.9 7.8 7.7 7.6 7.5 7.4 7.3 7.2 7.1 7.0 6.9 6.8
f1 (ppm)

13C NMR 7b

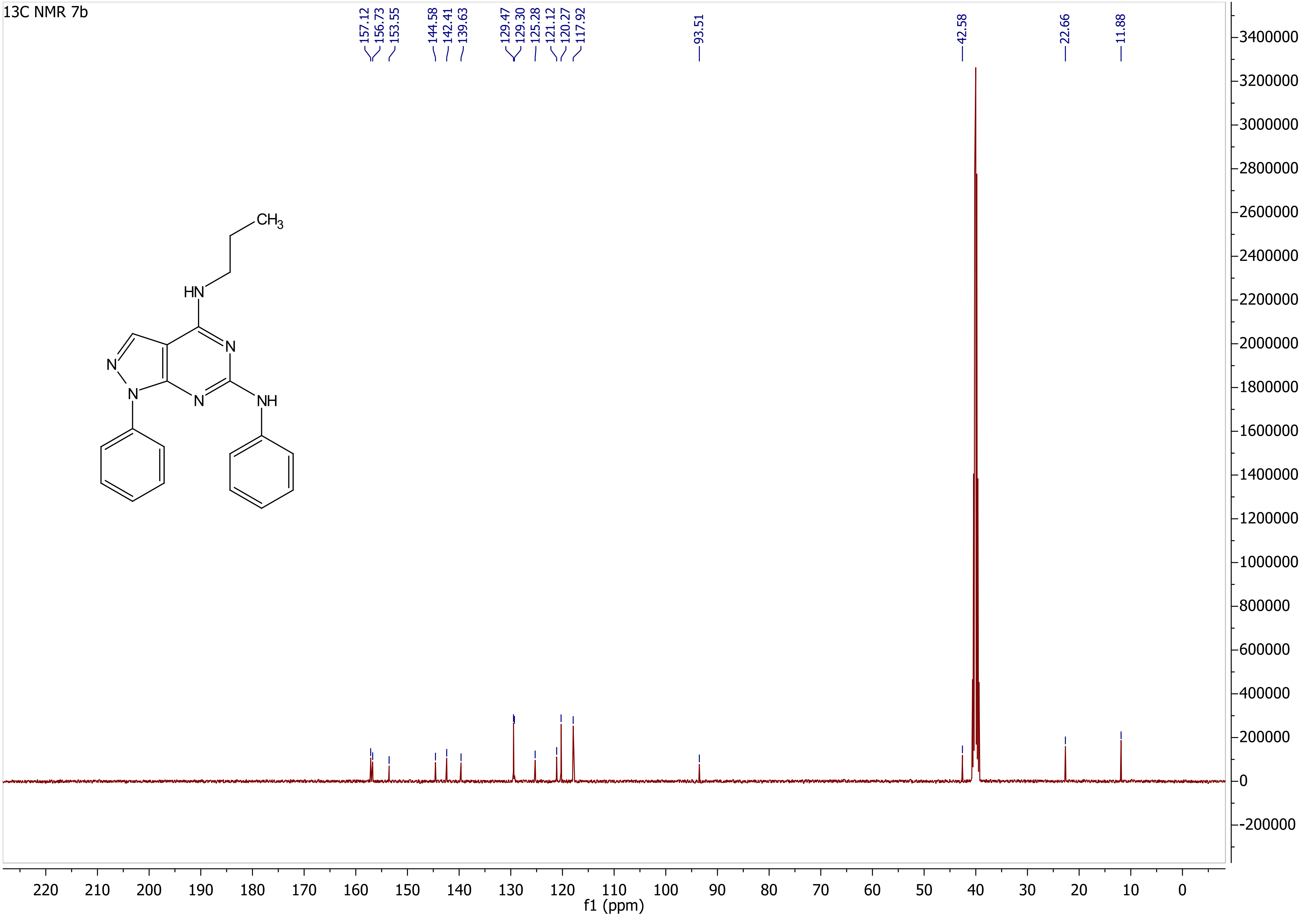
157.12
156.73
153.55
144.58
142.41
139.63
129.47
129.30
125.28
121.12
120.27
117.92
93.51
42.58
22.66
11.88
CH3
HN
N
N
N
N
NH
f1 (ppm)

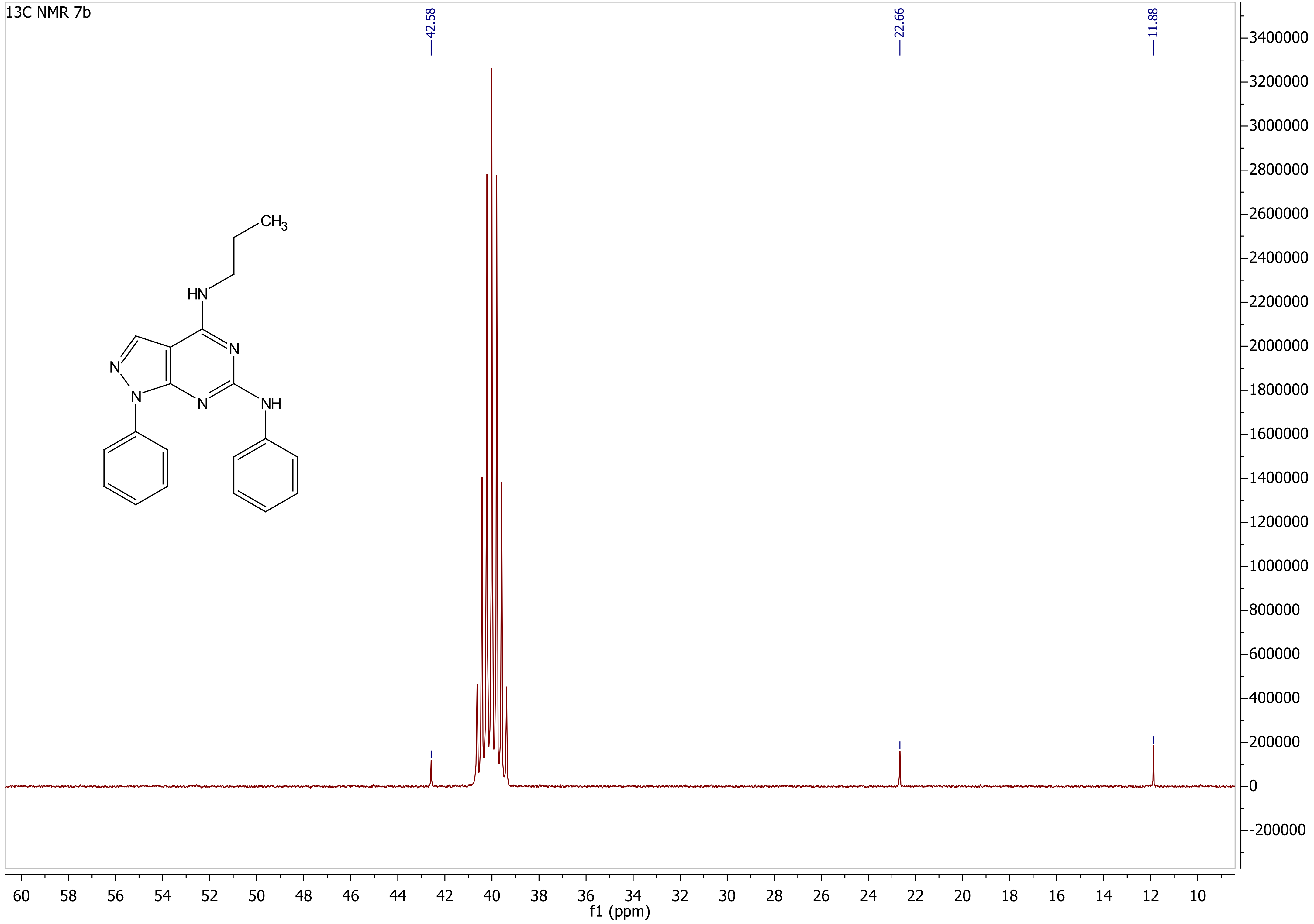
13C NMR 7b
42.58
22.66
11.88
CH3
HN
N
N
N
N
NH
f1 (ppm)

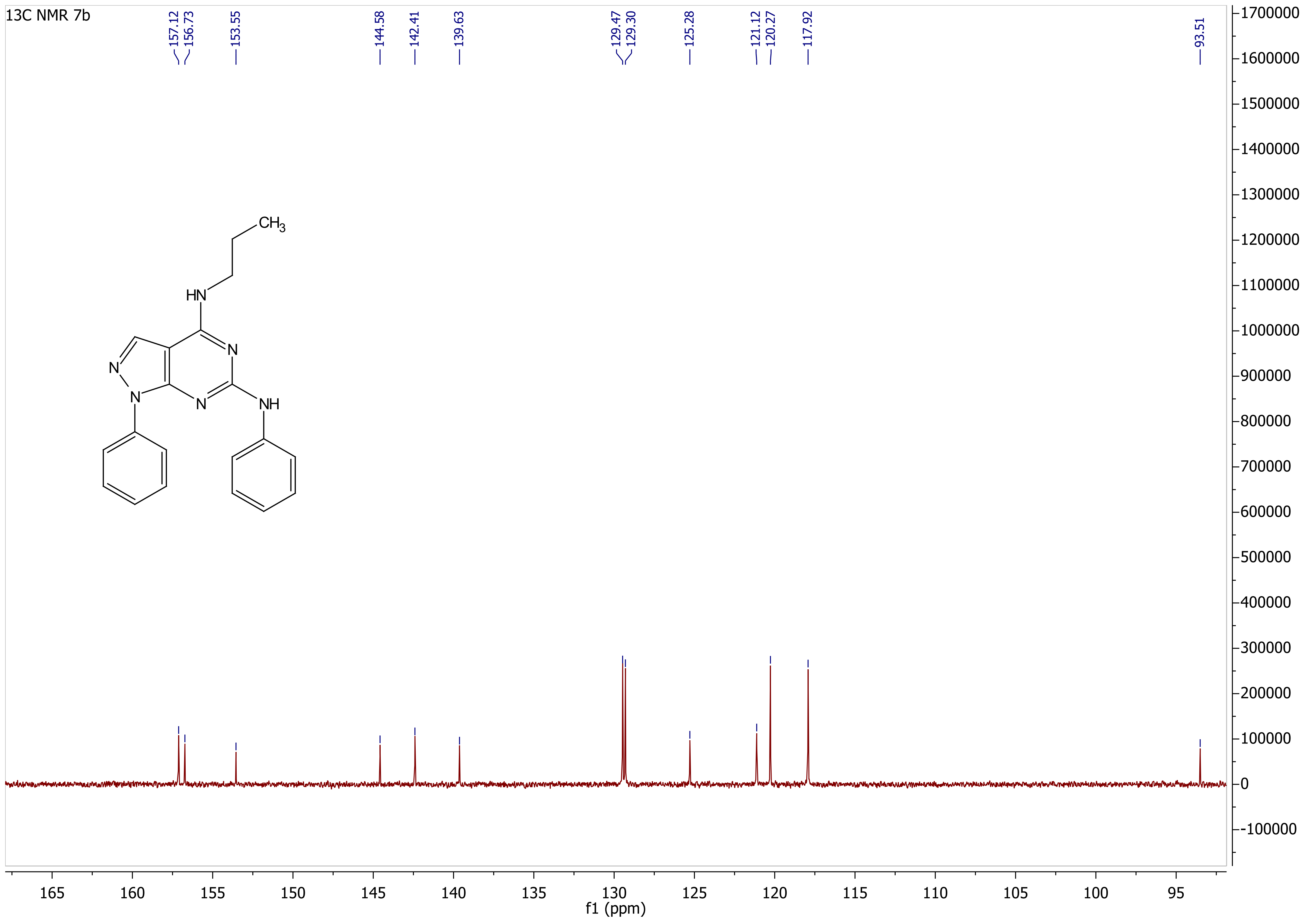

13C NMR 7b
157.12
156.73
153.55
144.58
142.41
139.63
129.47
129.30
125.28
121.12
120.27
117.92
93.51
CH3
HN
N
N
N
N
NH
f1 (ppm)

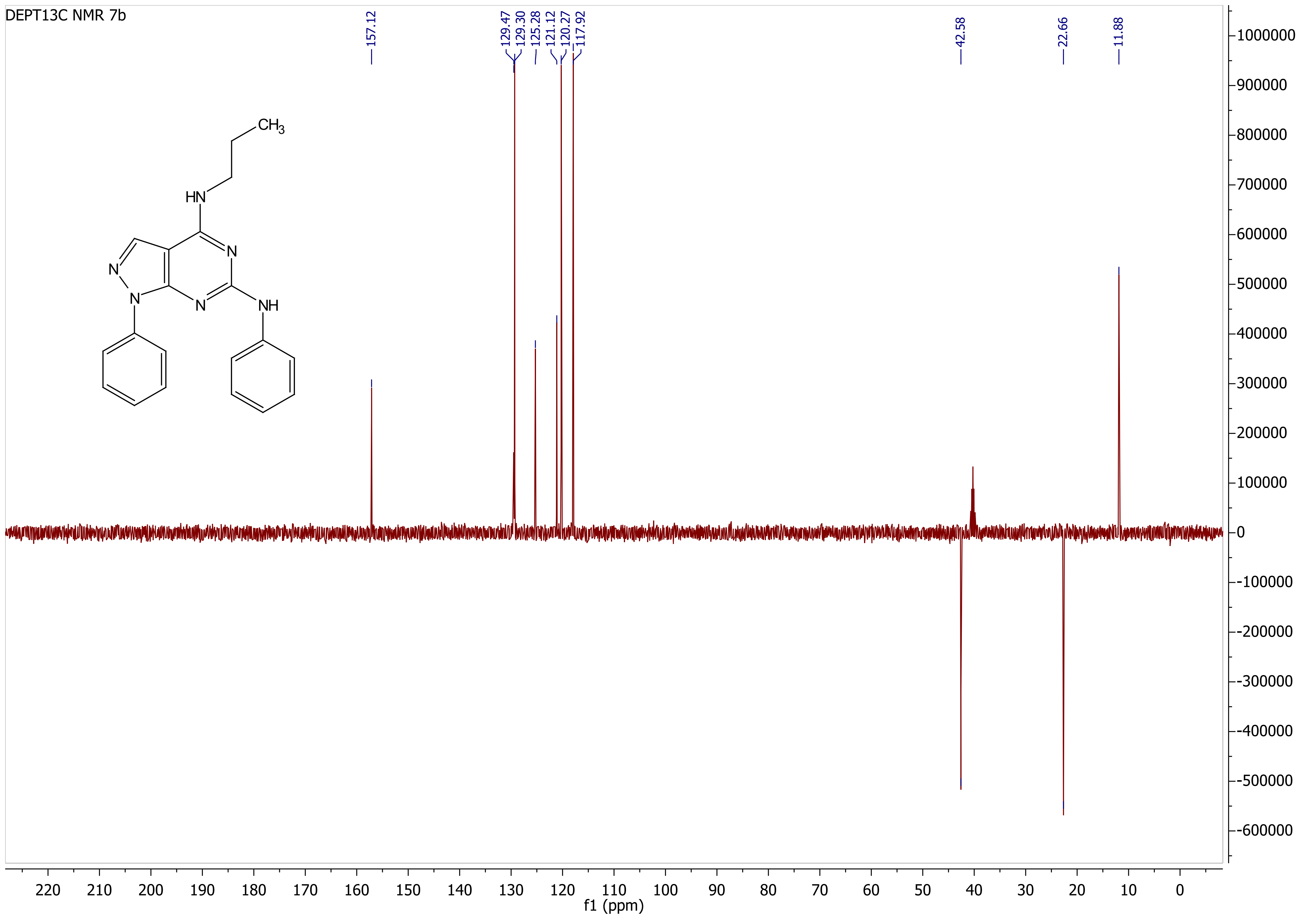
DEPT13C NMR 7b
157.12
129.47
129.30
125.28
121.12
120.27
117.92
42.58
22.66
11.88
CH3
HN
N
N
N
N
NH
f1 (ppm)
220 210 200 190 180 170 160 150 140 130 120 110 100 90 80 70 60 50 40 30 20 10 0
1000000 900000 800000 700000 600000 500000 400000 300000 200000 100000 0 -100000 -200000 -300000 -400000 -500000 -600000

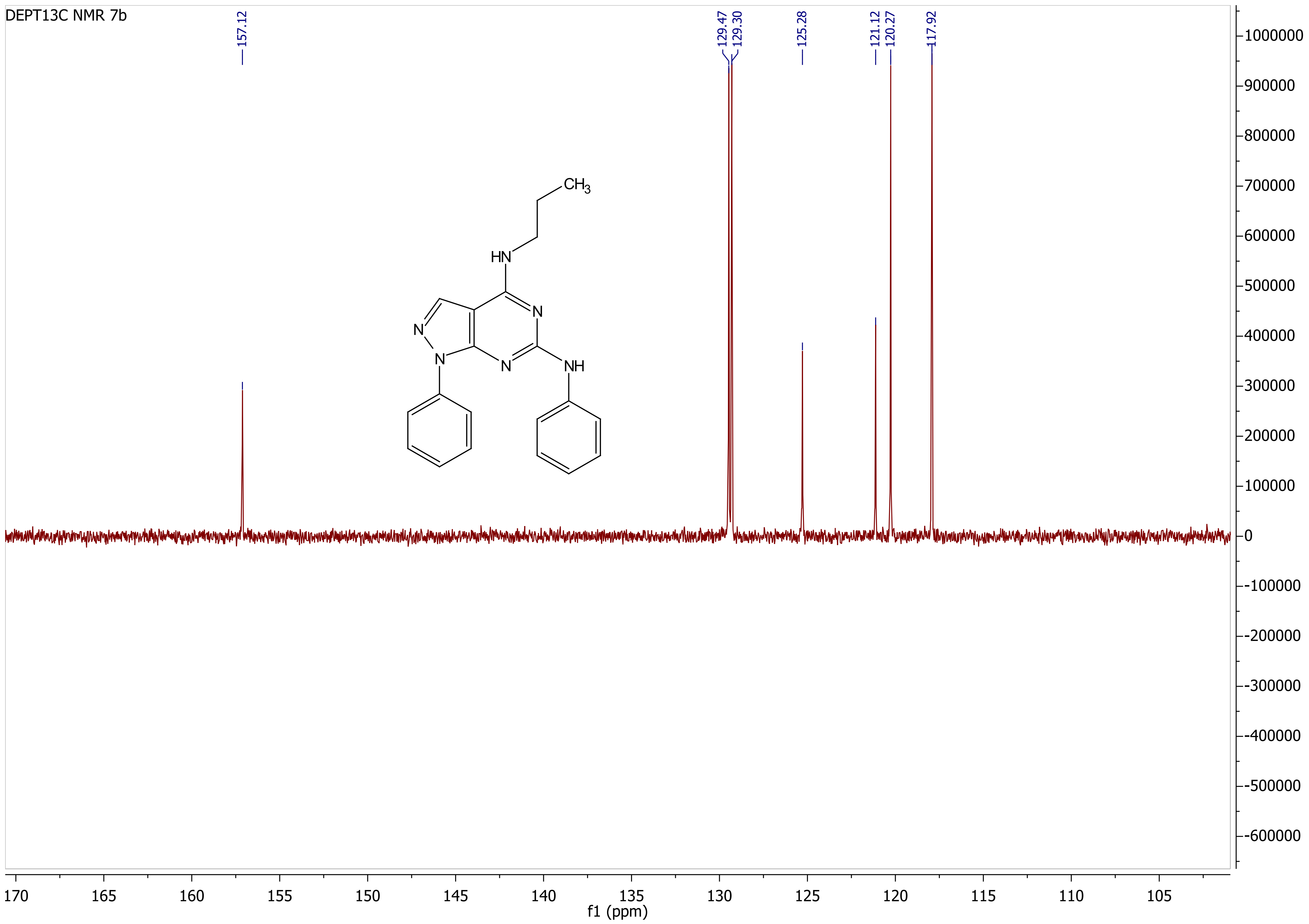
DEPT13C NMR 7b
157.12
129.47
129.30
125.28
121.12
120.27
117.92
CH3
HN
N
N
N
N
NH
1000000
900000
800000
700000
600000
500000
400000
300000
200000
100000
0
-100000
-200000
-300000
-400000
-500000
-600000
170
165
160
155
150
145
140
135
130
125
120
115
110
105
f1 (ppm)

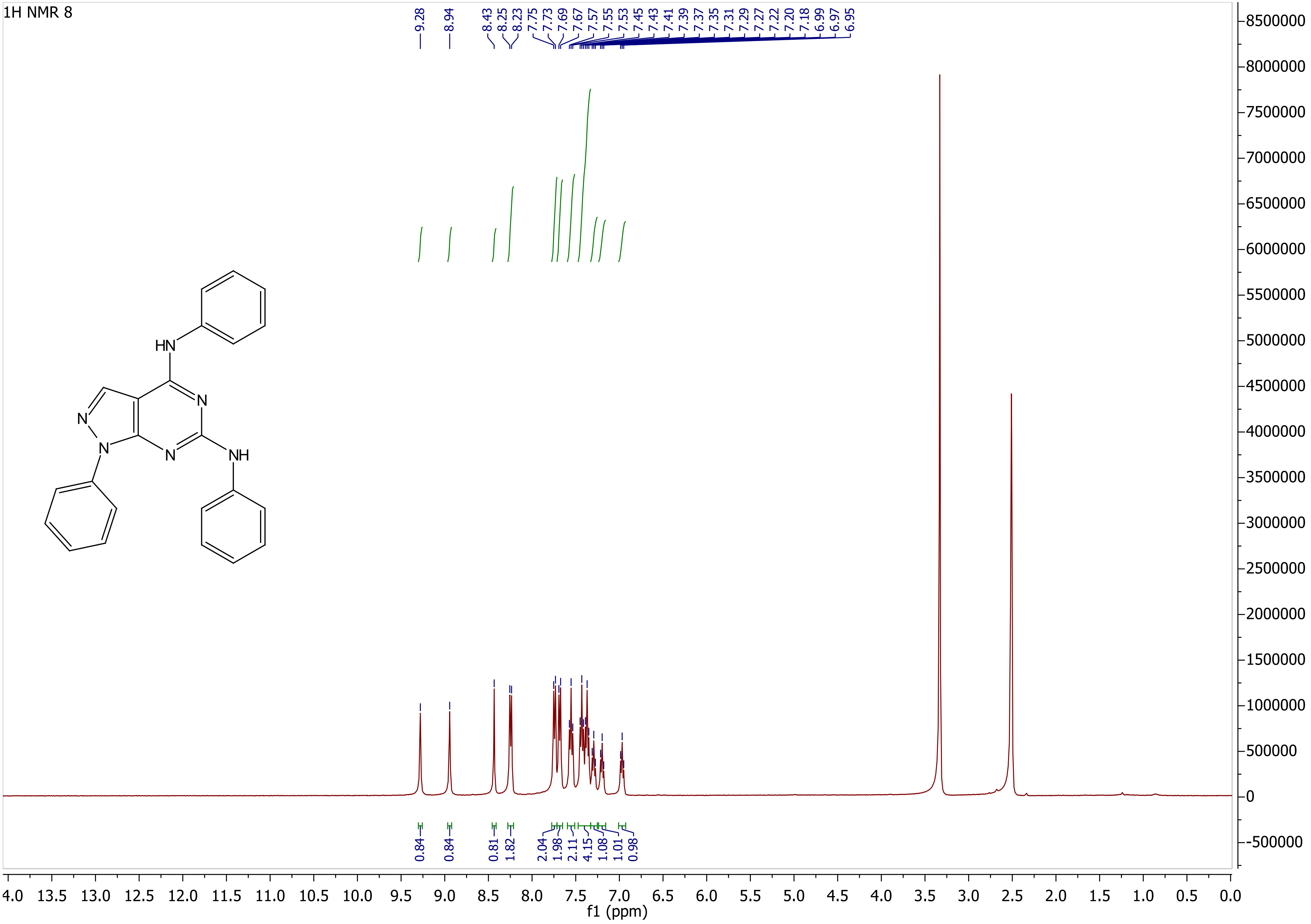
1H NMR 8
9.28
8.94
8.43
8.25
8.23
7.75
7.73
7.69
7.67
7.57
7.55
7.53
7.45
7.43
7.41
7.39
7.37
7.35
7.31
7.29
7.27
7.22
7.20
7.18
6.99
6.97
6.95
HN
N
N
N
N
NH
0.84
0.84
0.81
1.82
2.04
1.98
2.11
4.15
1.08
1.01
0.98
f1 (ppm)

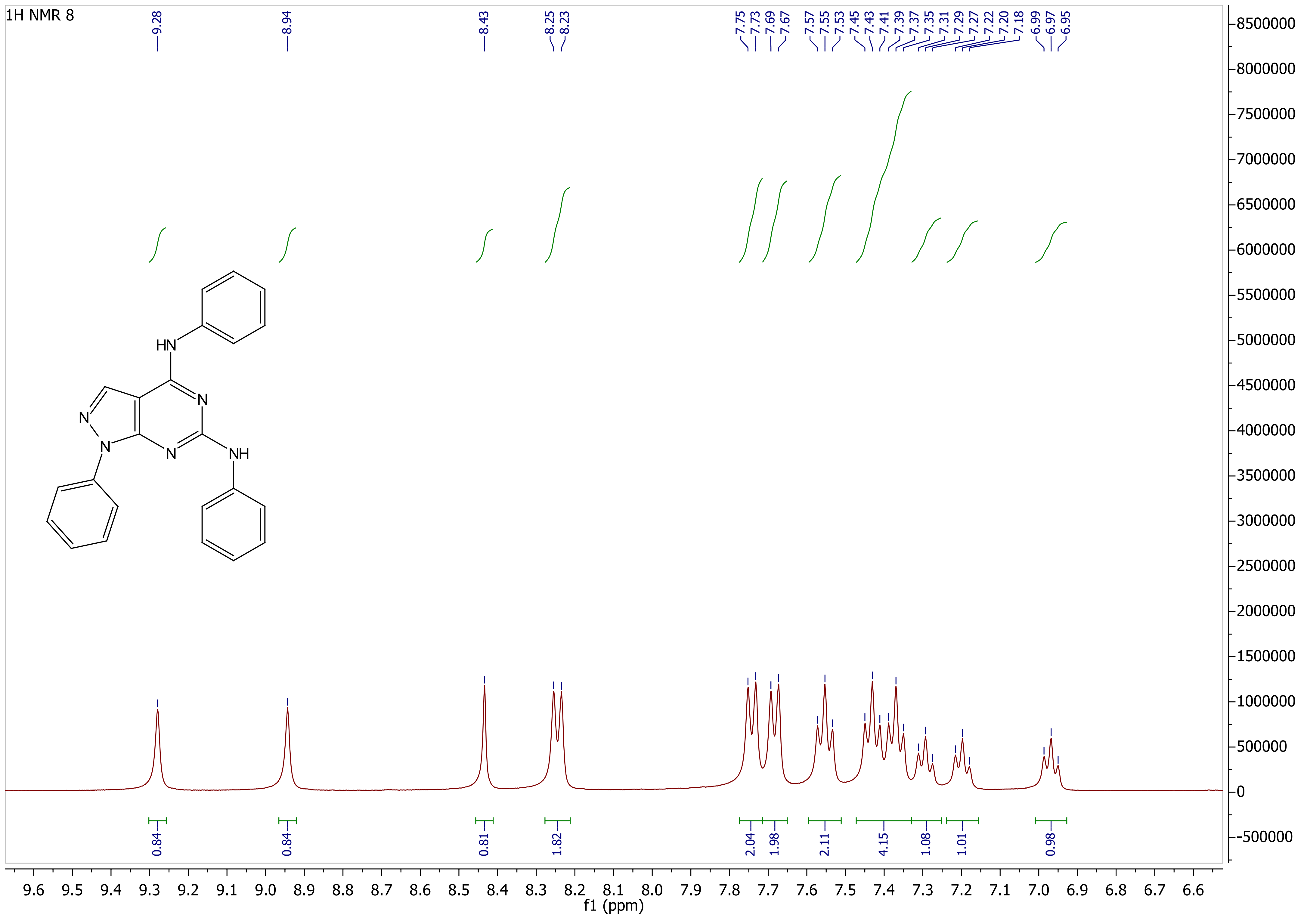
1H NMR 8
9.28
8.94
8.43
8.25
8.23
7.75
7.73
7.69
7.67
7.57
7.55
7.53
7.45
7.43
7.41
7.39
7.37
7.35
7.31
7.29
7.27
7.22
7.20
7.18
6.99
6.97
6.95
HN
N
N
N
N
NH
0.84
0.84
0.81
1.82
2.04
1.98
2.11
4.15
1.08
1.01
0.98
f1 (ppm)

13C NMR 8

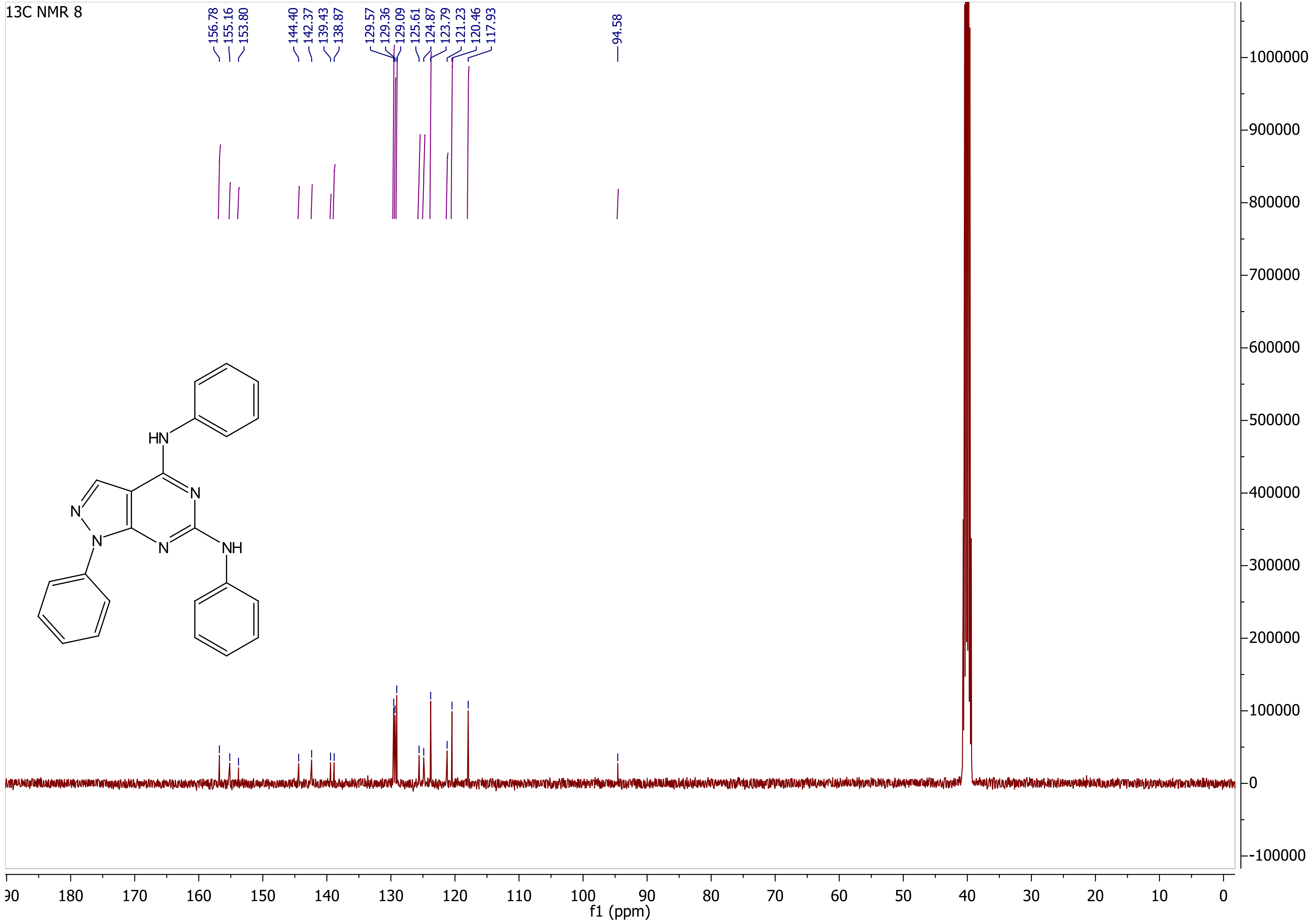
156.78
155.16
153.80
144.40
142.37
139.43
138.87
129.57
129.36
129.09
125.61
124.87
123.79
121.23
120.46
117.93
94.58
HN
N
N
N
N
NH
f1 (ppm)

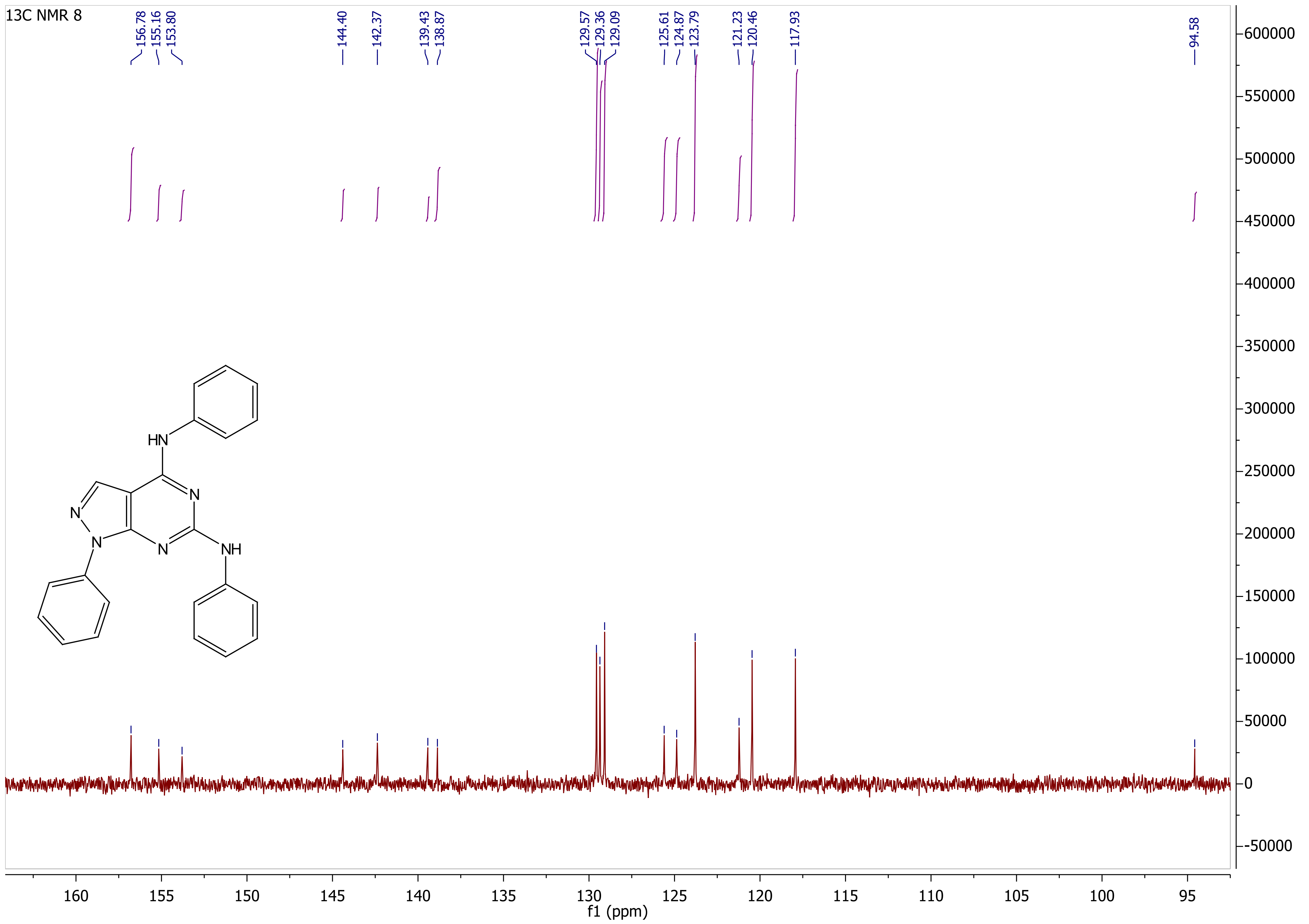
13C NMR 8
156.78
155.16
153.80
144.40
142.37
139.43
138.87
129.57
129.36
129.09
125.61
124.87
123.79
121.23
120.46
117.93
94.58
HN
N
N
N
N
NH
600000
550000
500000
450000
400000
350000
300000
250000
200000
150000
100000
50000
0
-50000
160
155
150
145
140
135
130
125
120
115
110
105
100
95
f1 (ppm)

DEPT 13C NMR 8

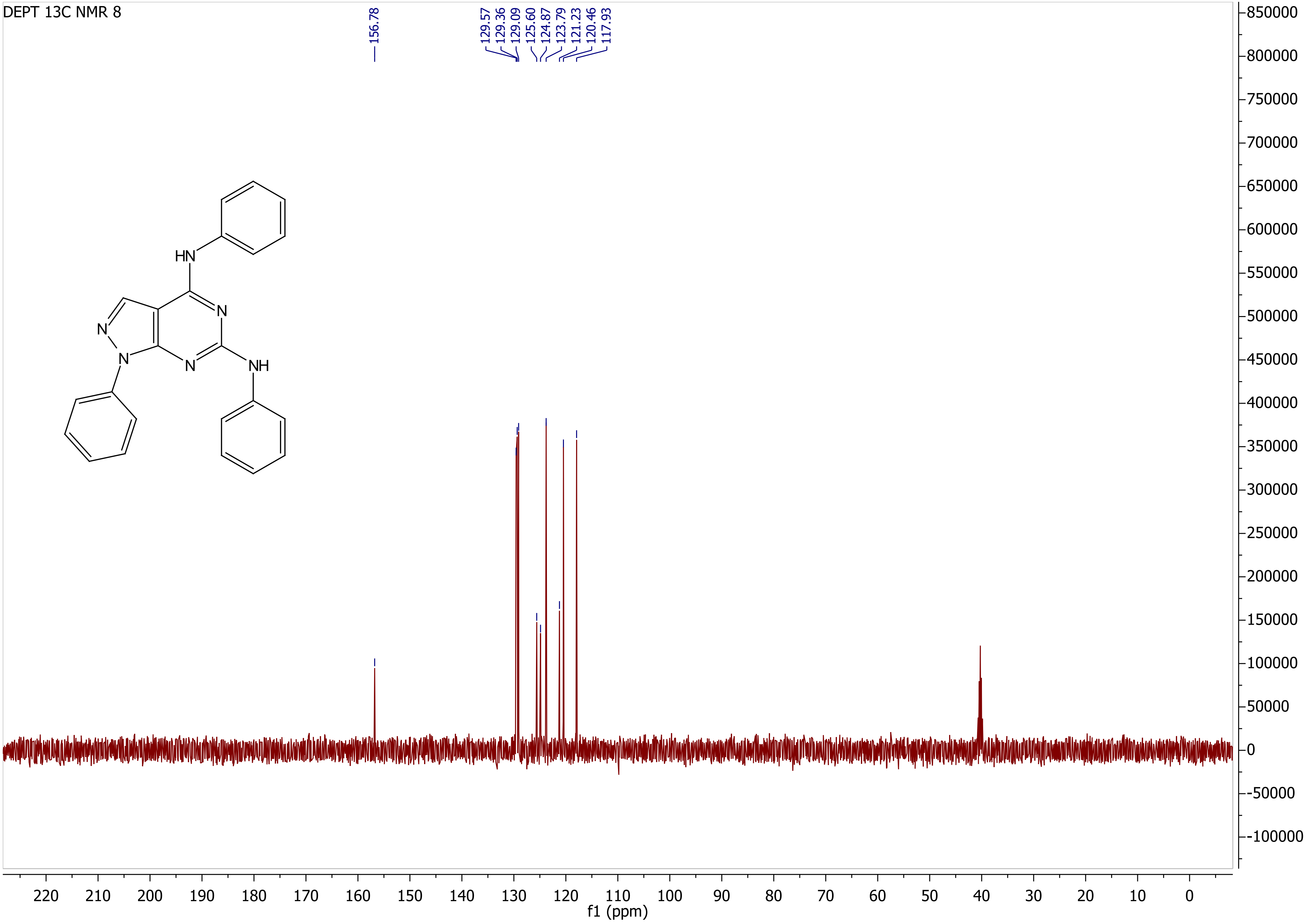

DEPT 13C NMR 8

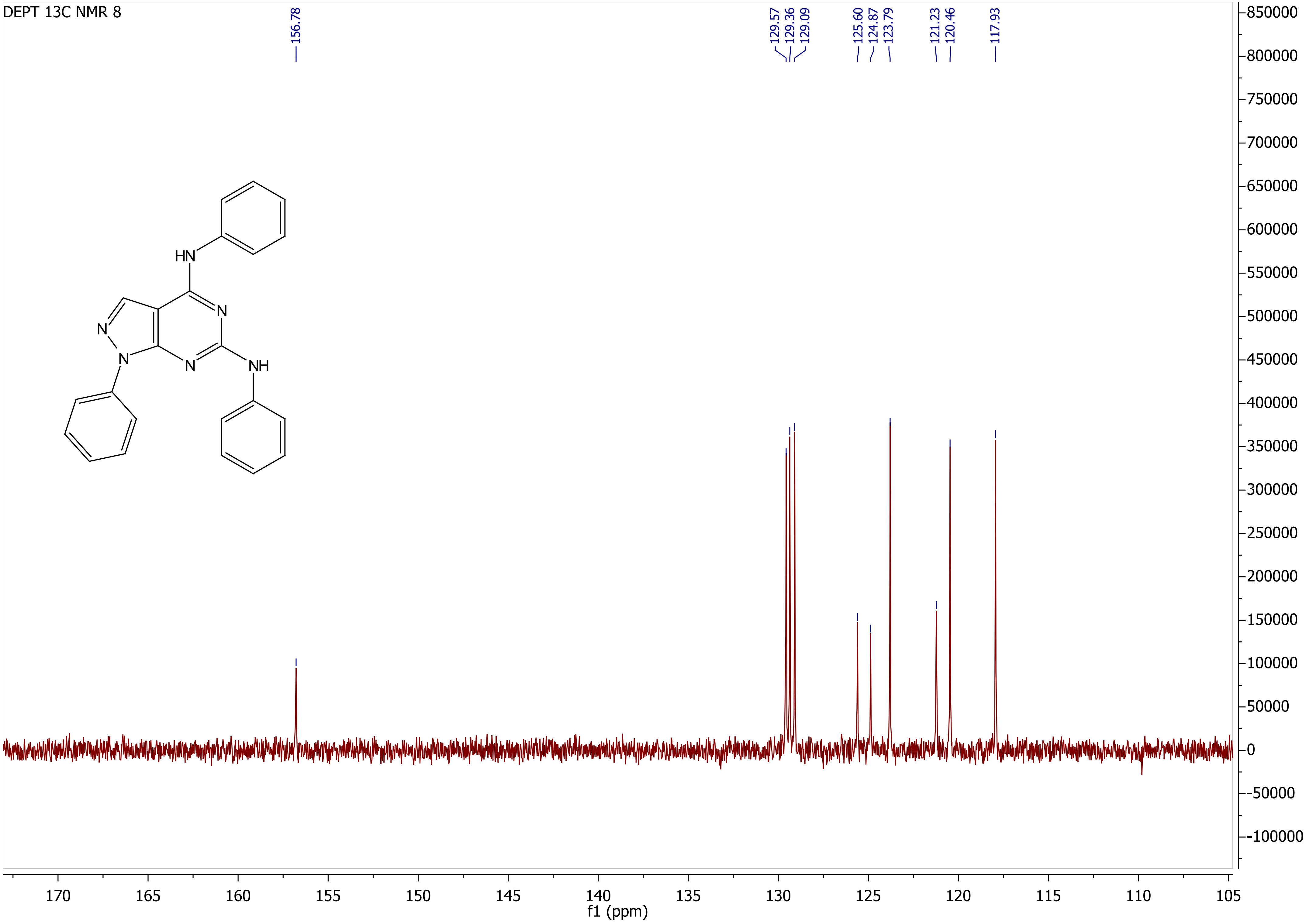

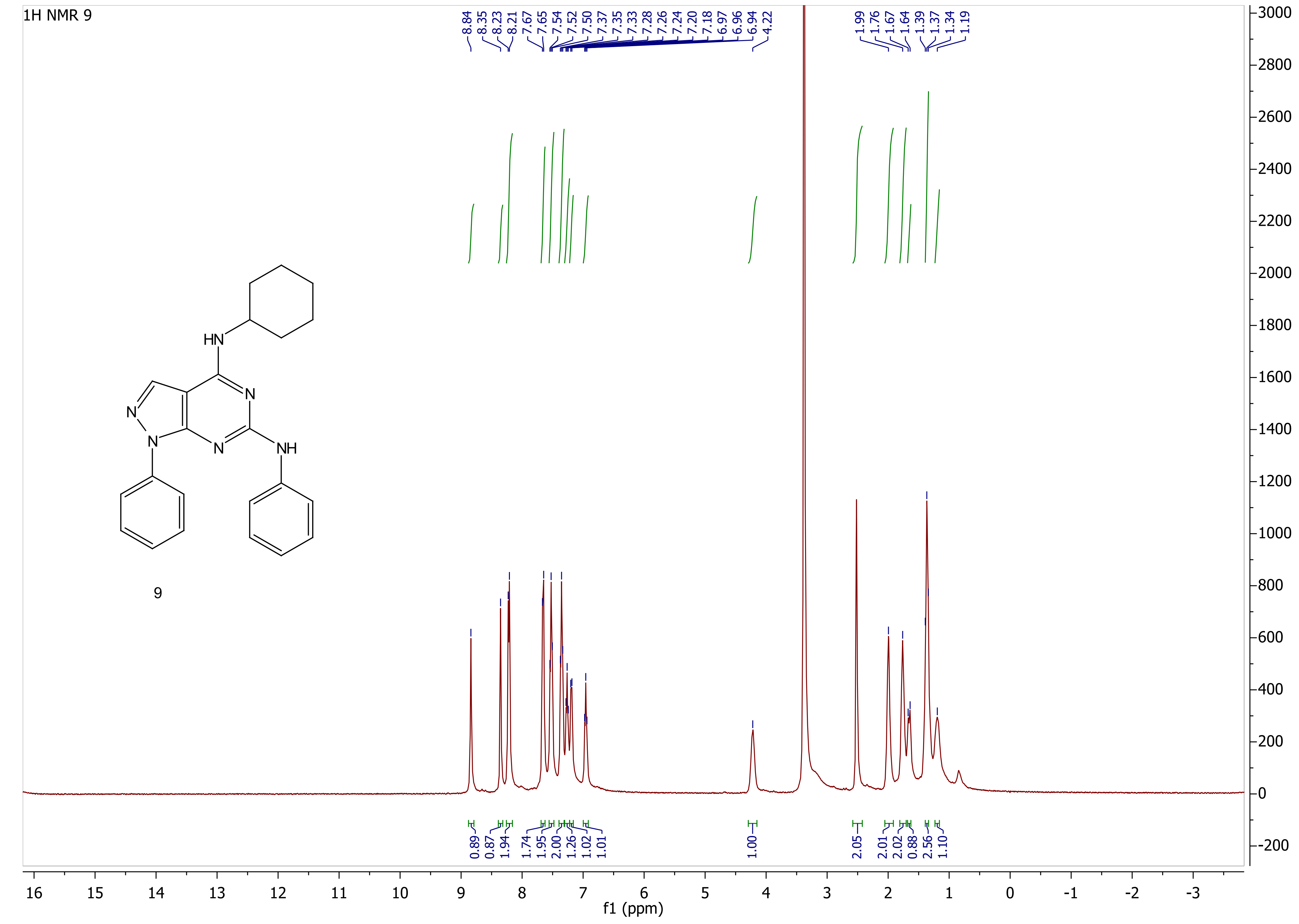
1H NMR 9
8.84
8.35
8.23
8.21
7.67
7.65
7.54
7.52
7.50
7.37
7.35
7.33
7.28
7.26
7.24
7.20
7.18
6.97
6.96
6.94
4.22
1.99
1.76
1.67
1.64
1.39
1.37
1.34
1.19
HN
N
N
N
N
NH
9
0.89
0.87
1.94
1.74
1.95
2.00
1.26
1.02
1.01
1.00
2.05
2.01
2.02
0.88
2.56
1.10
f1 (ppm)

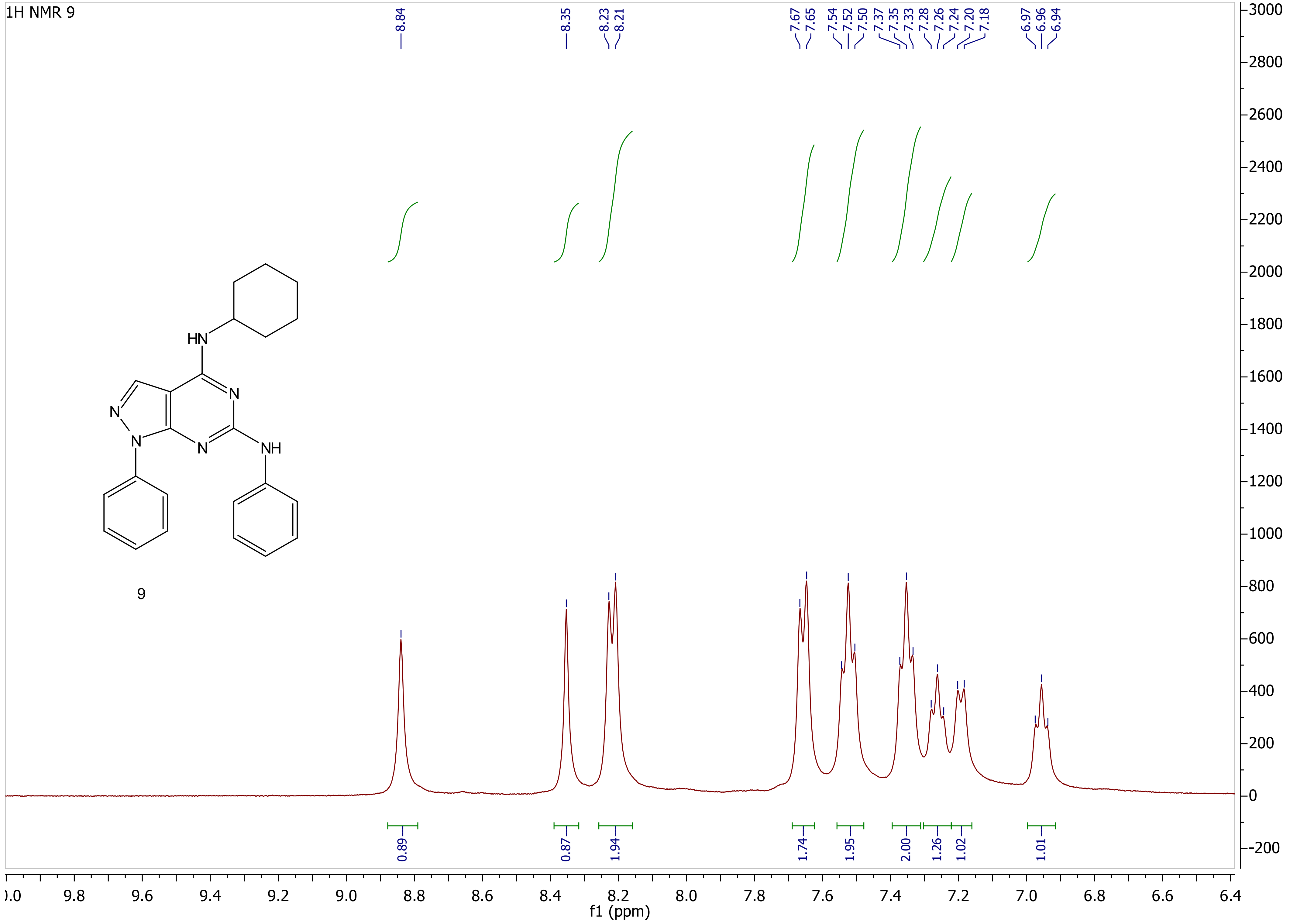

1H NMR 9
8.84
8.35
8.23
8.21
7.67
7.65
7.54
7.52
7.50
7.37
7.35
7.33
7.28
7.26
7.24
7.20
7.18
6.97
6.96
6.94
HN
N
N
N
N
NH
9
0.89
0.87
1.94
1.74
1.95
2.00
1.26
1.02
1.01
3000
2800
2600
2400
2200
2000
1800
1600
1400
1200
1000
800
600
400
200
0
-200
9.8
9.6
9.4
9.2
9.0
8.8
8.6
8.4
8.2
8.0
7.8
7.6
7.4
7.2
7.0
6.8
6.6
6.4
f1 (ppm)

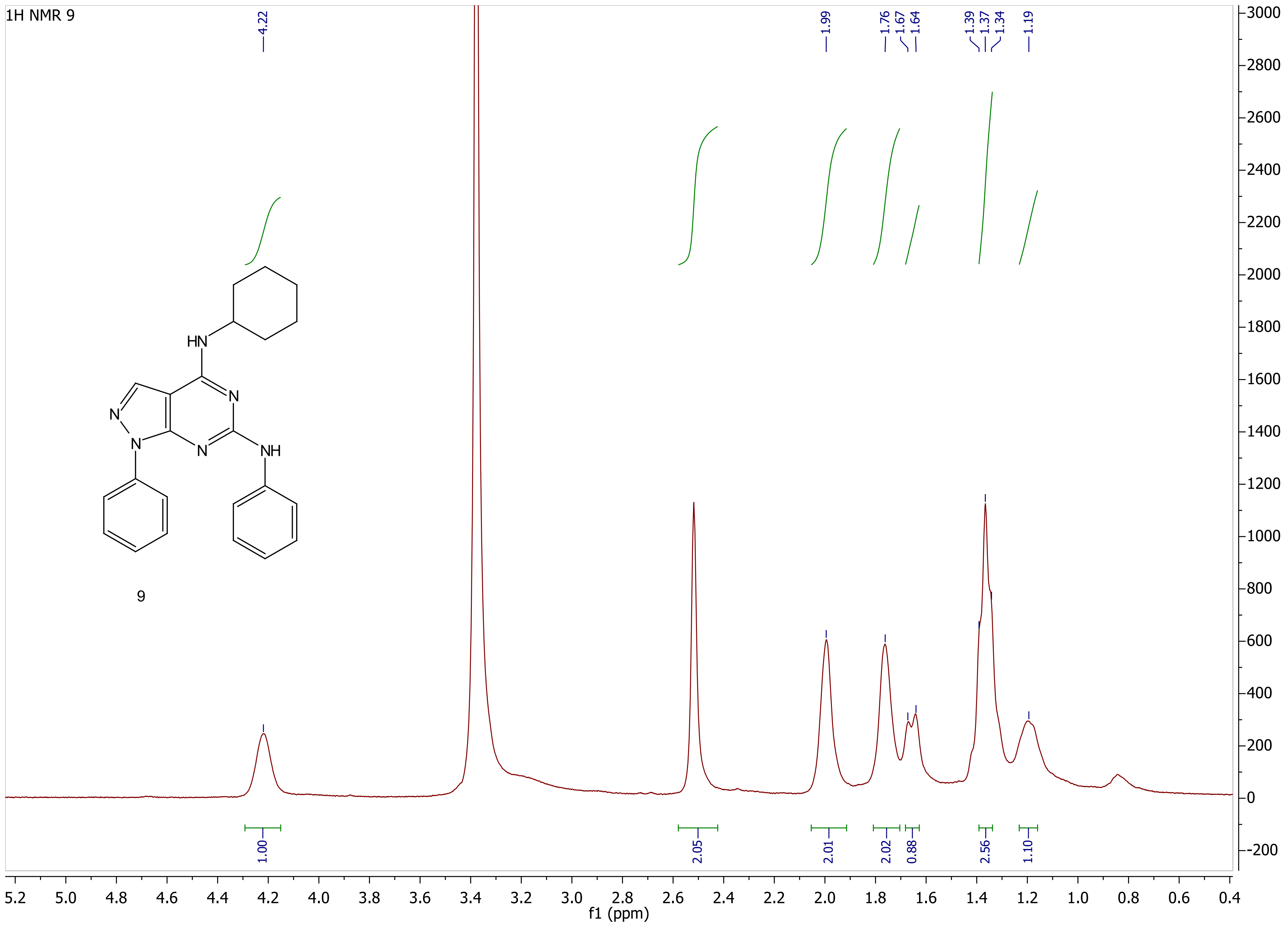
1H NMR 9
4.22
1.99
1.76
1.67
1.64
1.39
1.37
1.34
1.19
HN
N
N
N
N
NH
9
1.00
2.05
2.01
2.02
0.88
2.56
1.10
f1 (ppm)

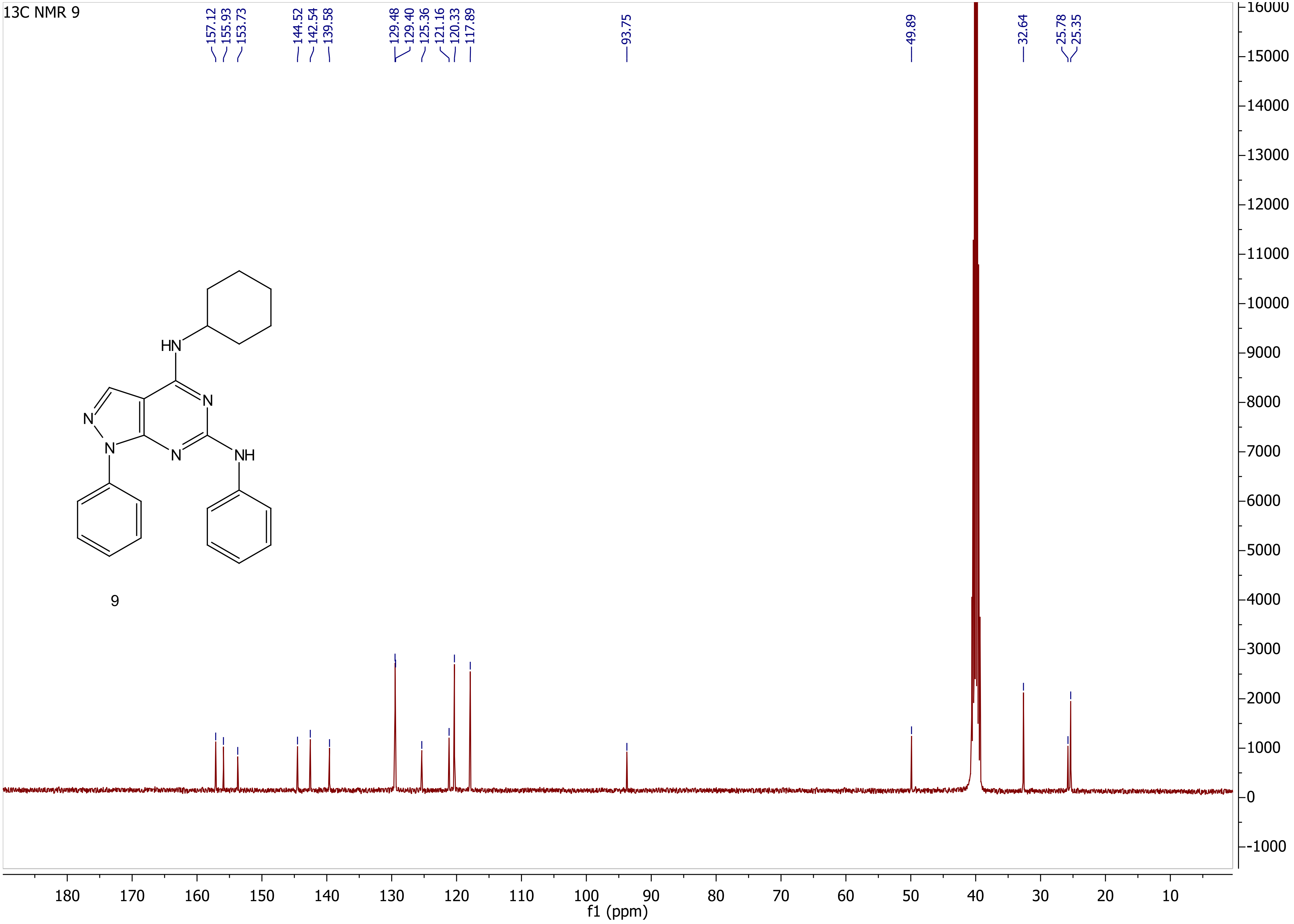

13C NMR 9
157.12
155.93
153.73
144.52
142.54
139.58
129.48
129.40
125.36
121.16
120.33
117.89
93.75
49.89
32.64
25.78
25.35
HN
N
N
N
N
NH
9
16000
15000
14000
13000
12000
11000
10000
9000
8000
7000
6000
5000
4000
3000
2000
1000
0
-1000
180
170
160
150
140
130
120
110
100
90
80
70
60
50
40
30
20
10
f1 (ppm)

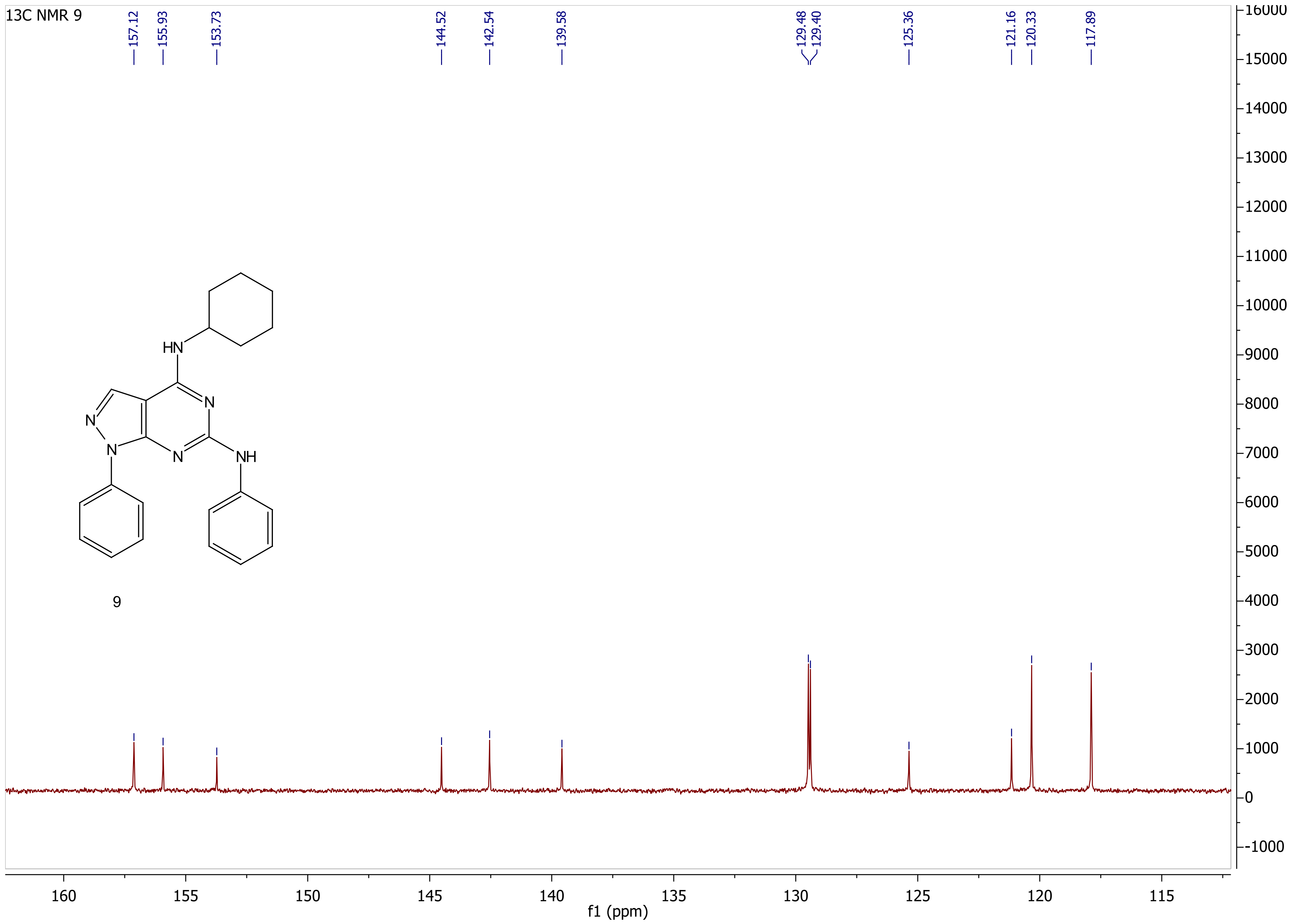
13C NMR 9
157.12
155.93
153.73
144.52
142.54
139.58
129.48
129.40
125.36
121.16
120.33
117.89
HN
N
N
N
N
NH
9
16000
15000
14000
13000
12000
11000
10000
9000
8000
7000
6000
5000
4000
3000
2000
1000
0
-1000
160
155
150
145
140
135
130
125
120
115
f1 (ppm)

13C NMR 9

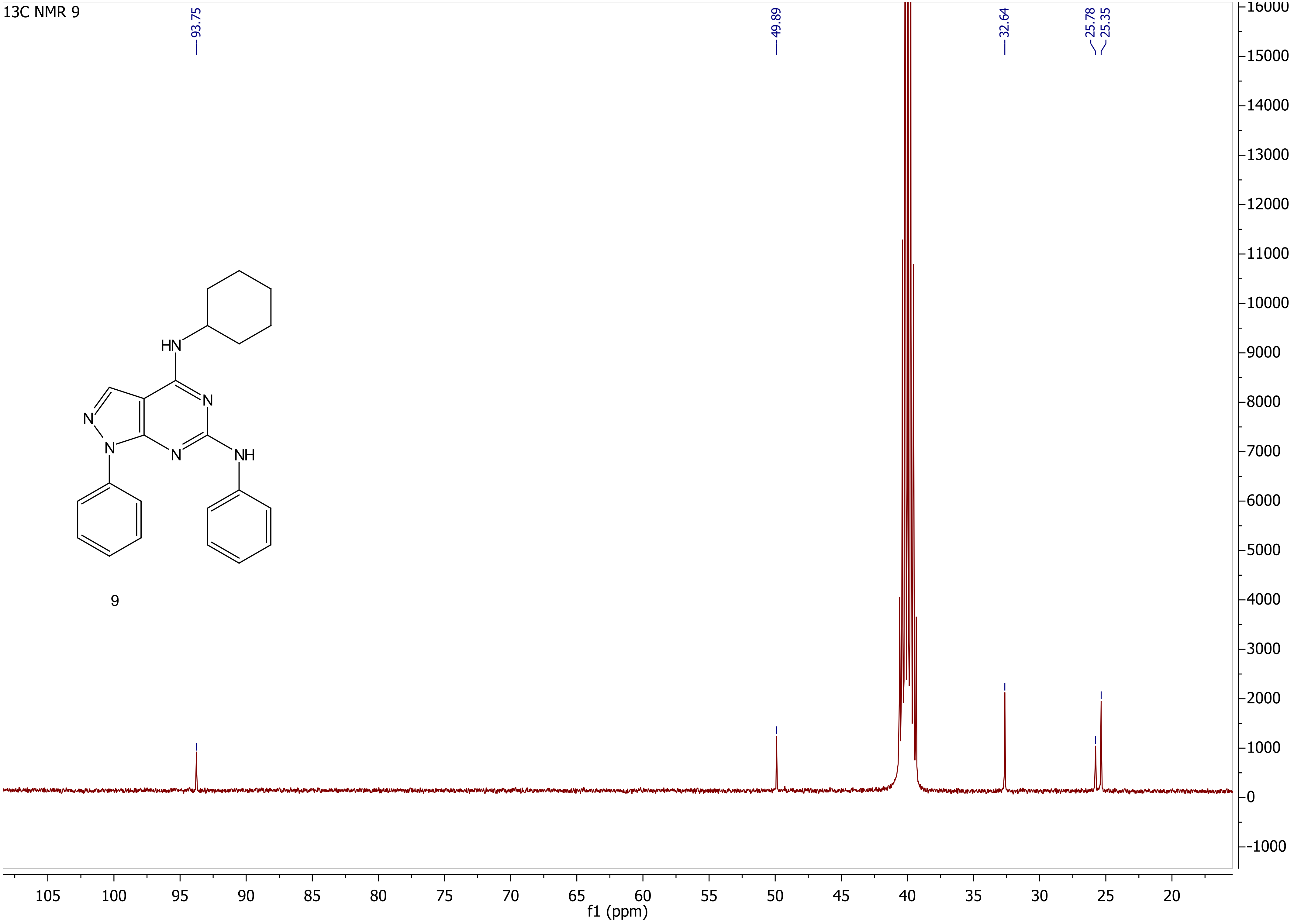
93.75
49.89
32.64
25.78
25.35
16000
15000
14000
13000
12000
11000
10000
9000
8000
7000
6000
5000
4000
3000
2000
1000
0
-1000
105
100
95
90
85
80
75
70
65
60
55
50
45
40
35
30
25
20
f1 (ppm)
HN
N
N
N
N
NH
9

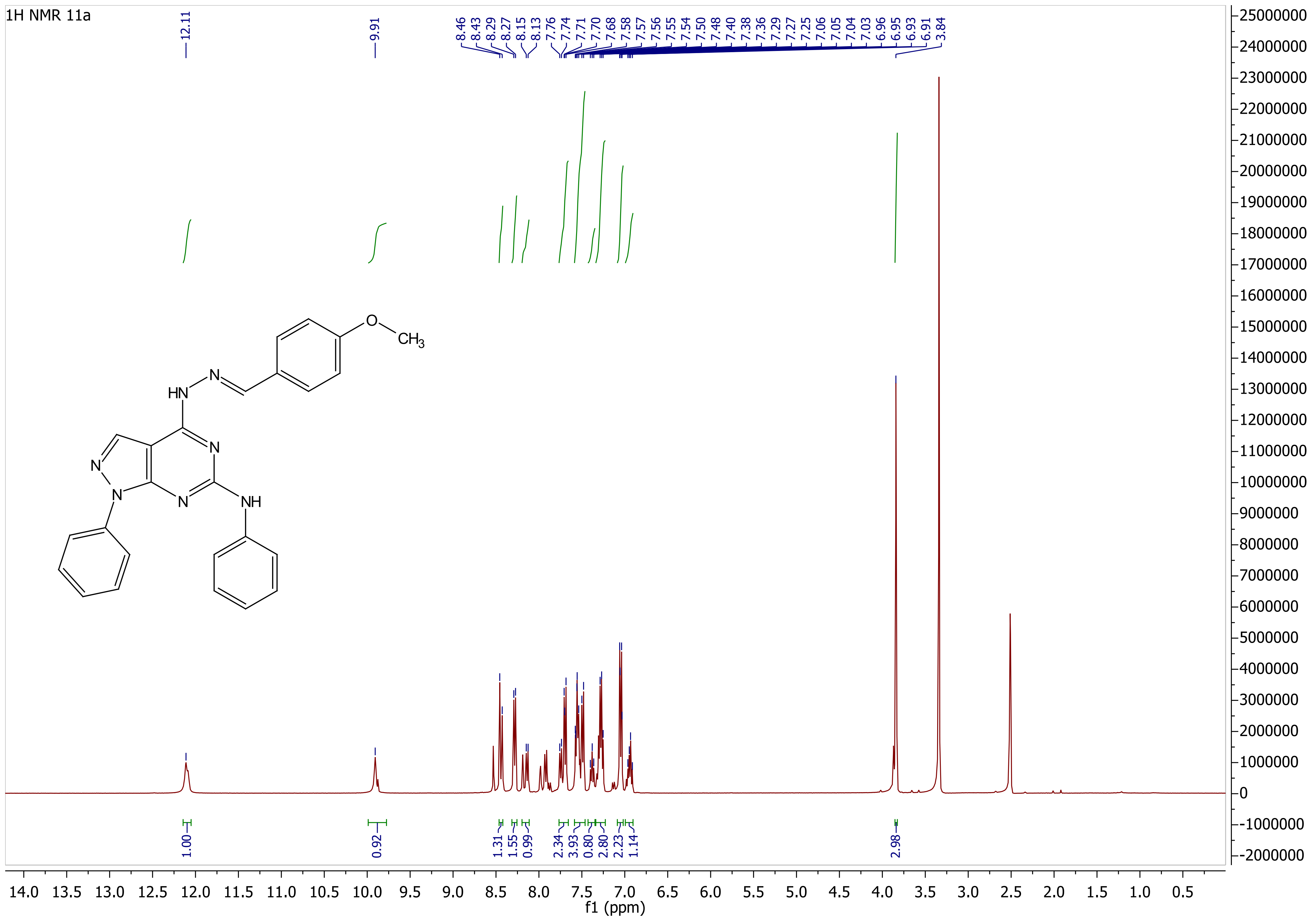
1H NMR 11a
12.11
9.91
8.46 8.43 8.29 8.27 8.15 8.13 7.76 7.74 7.71 7.70 7.68 7.58 7.57 7.56 7.55 7.54 7.50 7.48 7.40 7.38 7.36 7.29 7.27 7.25 7.06 7.05 7.04 7.03 6.96 6.95 6.93 6.91 3.84
1.00 0.92 1.31 1.55 0.99 2.34 3.93 0.80 2.80 2.23 1.14 2.98
f1 (ppm)
HN
N
O
CH3
N
N
N
N
NH

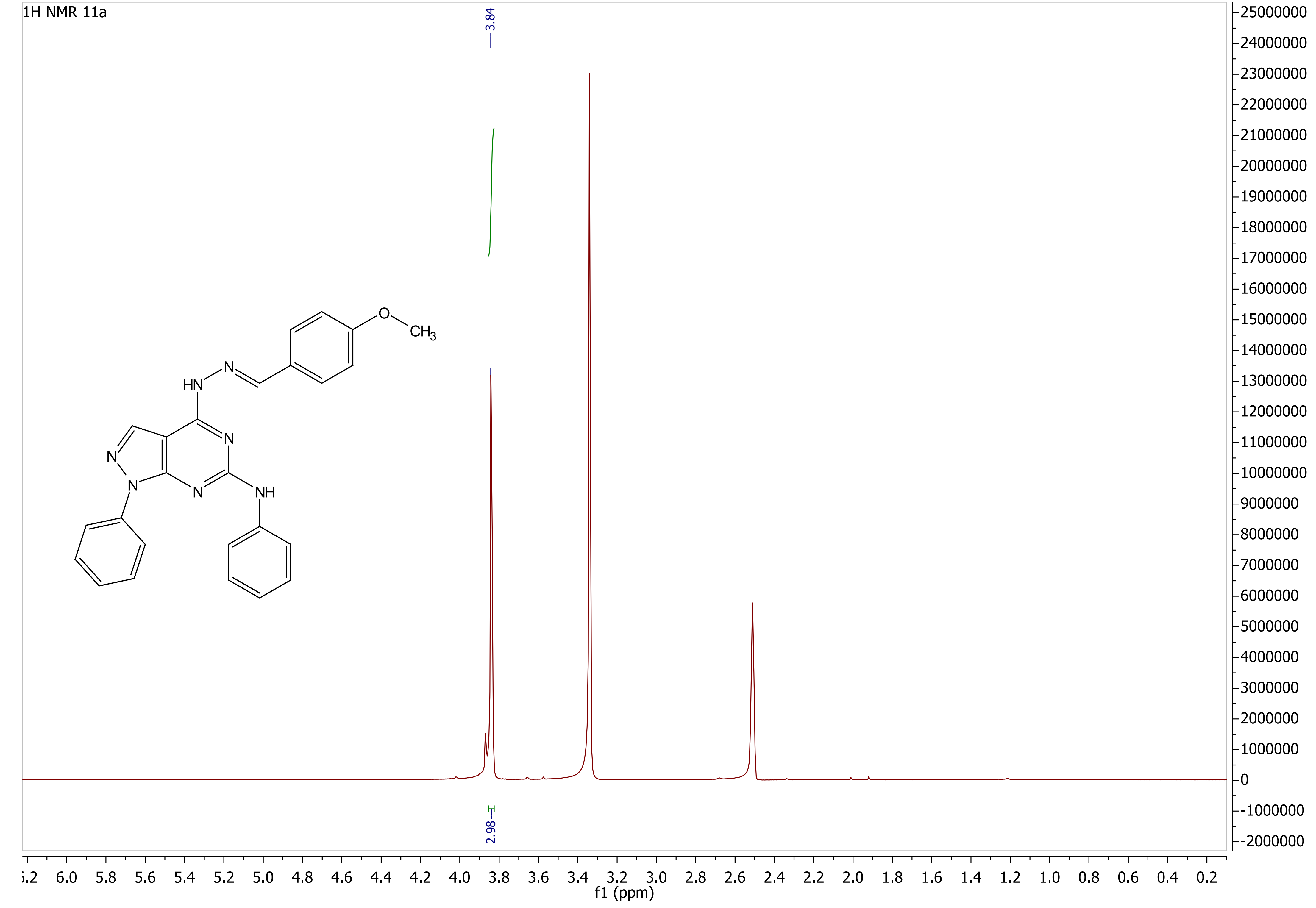
1H NMR 11a
3.84
2.98
O
CH3
N
HN
N
N
N
N
NH
f1 (ppm)
6.0 5.8 5.6 5.4 5.2 5.0 4.8 4.6 4.4 4.2 4.0 3.8 3.6 3.4 3.2 3.0 2.8 2.6 2.4 2.2 2.0 1.8 1.6 1.4 1.2 1.0 0.8 0.6 0.4 0.2
25000000 24000000 23000000 22000000 21000000 20000000 19000000 18000000 17000000 16000000 15000000 14000000 13000000 12000000 11000000 10000000 9000000 8000000 7000000 6000000 5000000 4000000 3000000 2000000 1000000 0 -1000000 -2000000

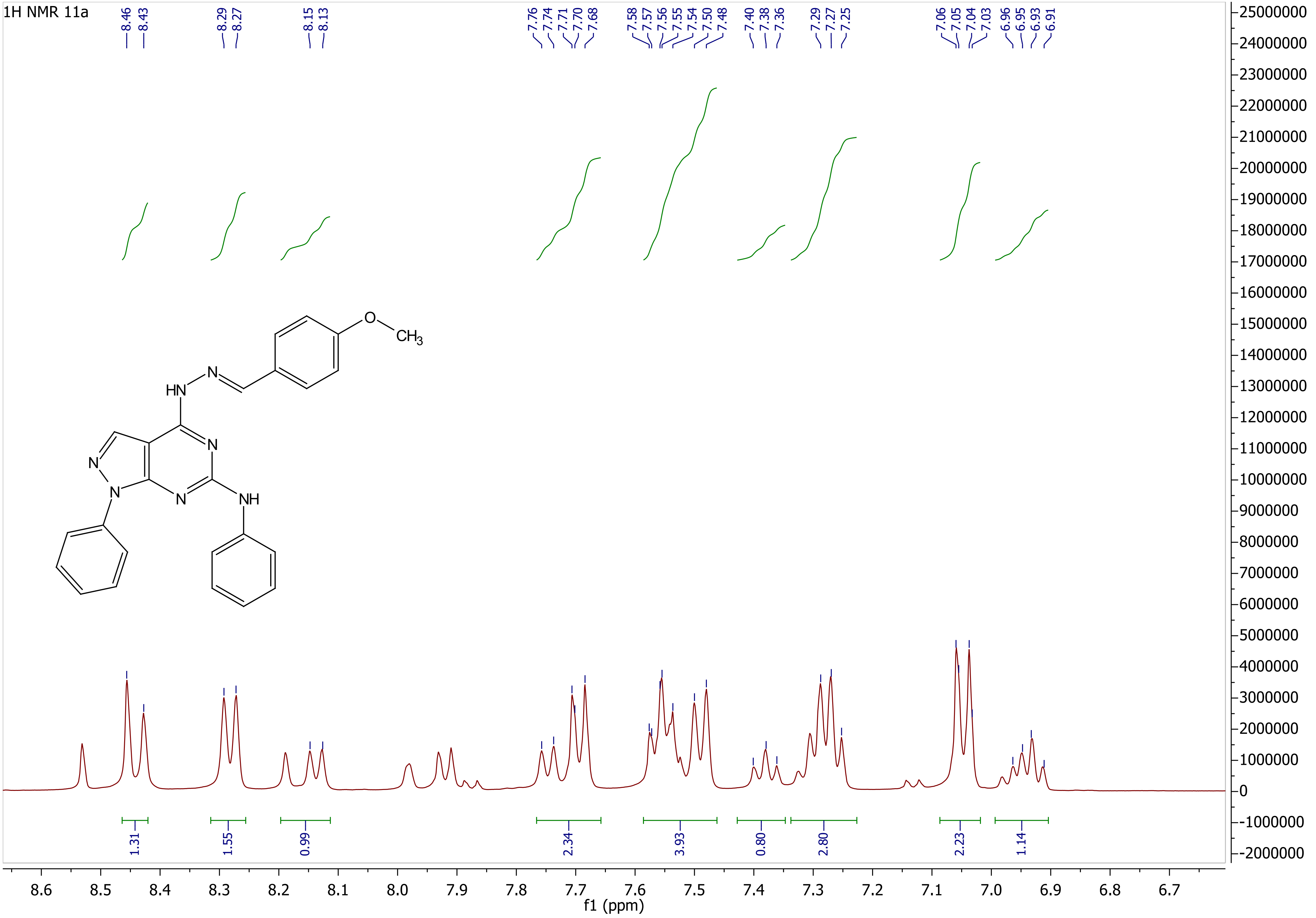
1H NMR 11a
8.46
8.43
8.29
8.27
8.15
8.13
7.76
7.74
7.71
7.70
7.68
7.58
7.57
7.56
7.55
7.54
7.50
7.48
7.40
7.38
7.36
7.29
7.27
7.25
7.06
7.05
7.04
7.03
6.96
6.95
6.93
6.91
1.31
1.55
0.99
2.34
3.93
0.80
2.80
2.23
1.14
f1 (ppm)

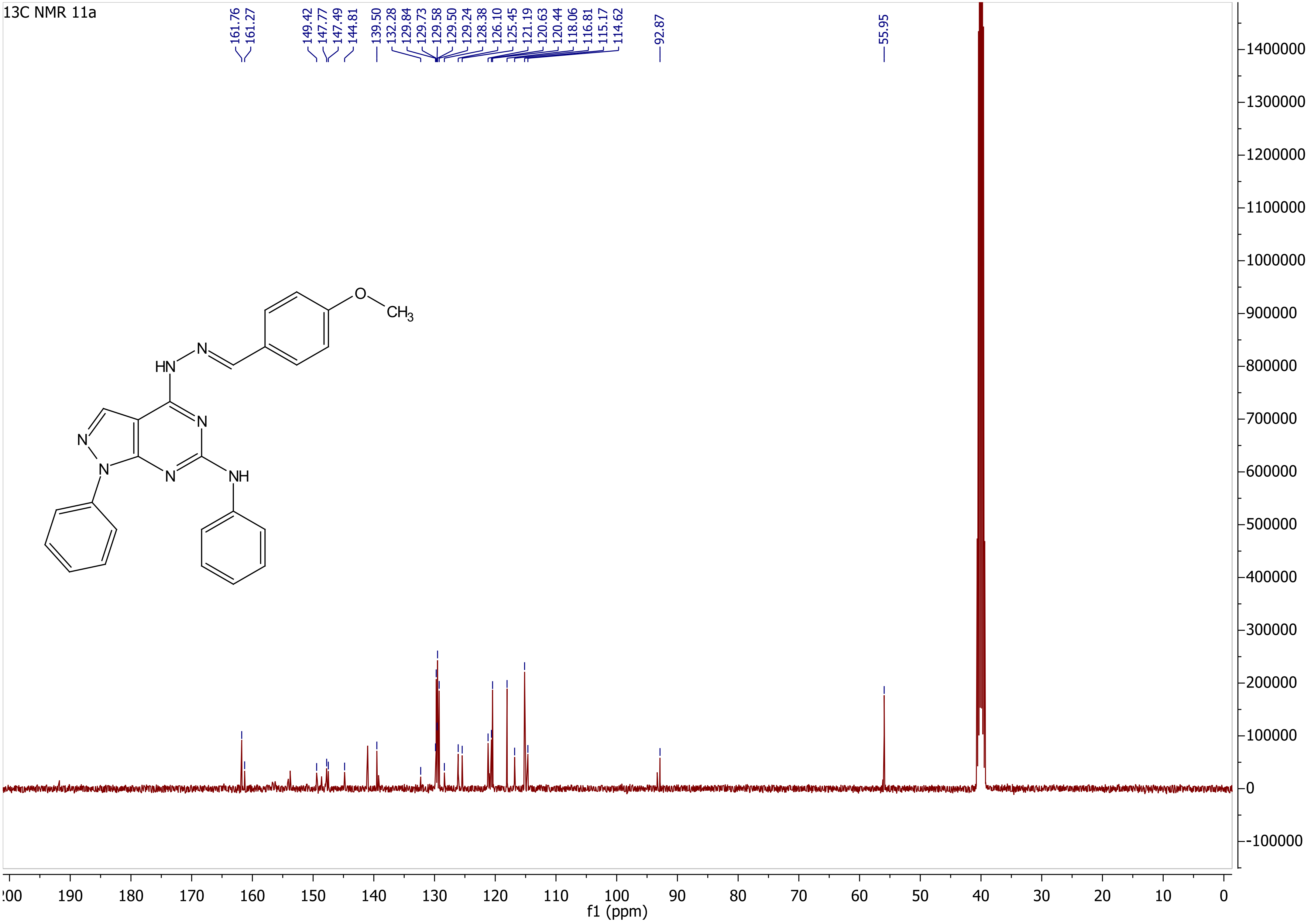
13C NMR 11a
161.76
161.27
149.42
147.77
147.49
144.81
139.50
132.28
129.84
129.73
129.58
129.50
129.24
128.38
126.10
125.45
121.19
120.63
120.44
118.06
116.81
115.17
114.62
92.87
55.95
HN
N
O
CH3
N
N
N
N
NH
f1 (ppm)

13C NMR 11a

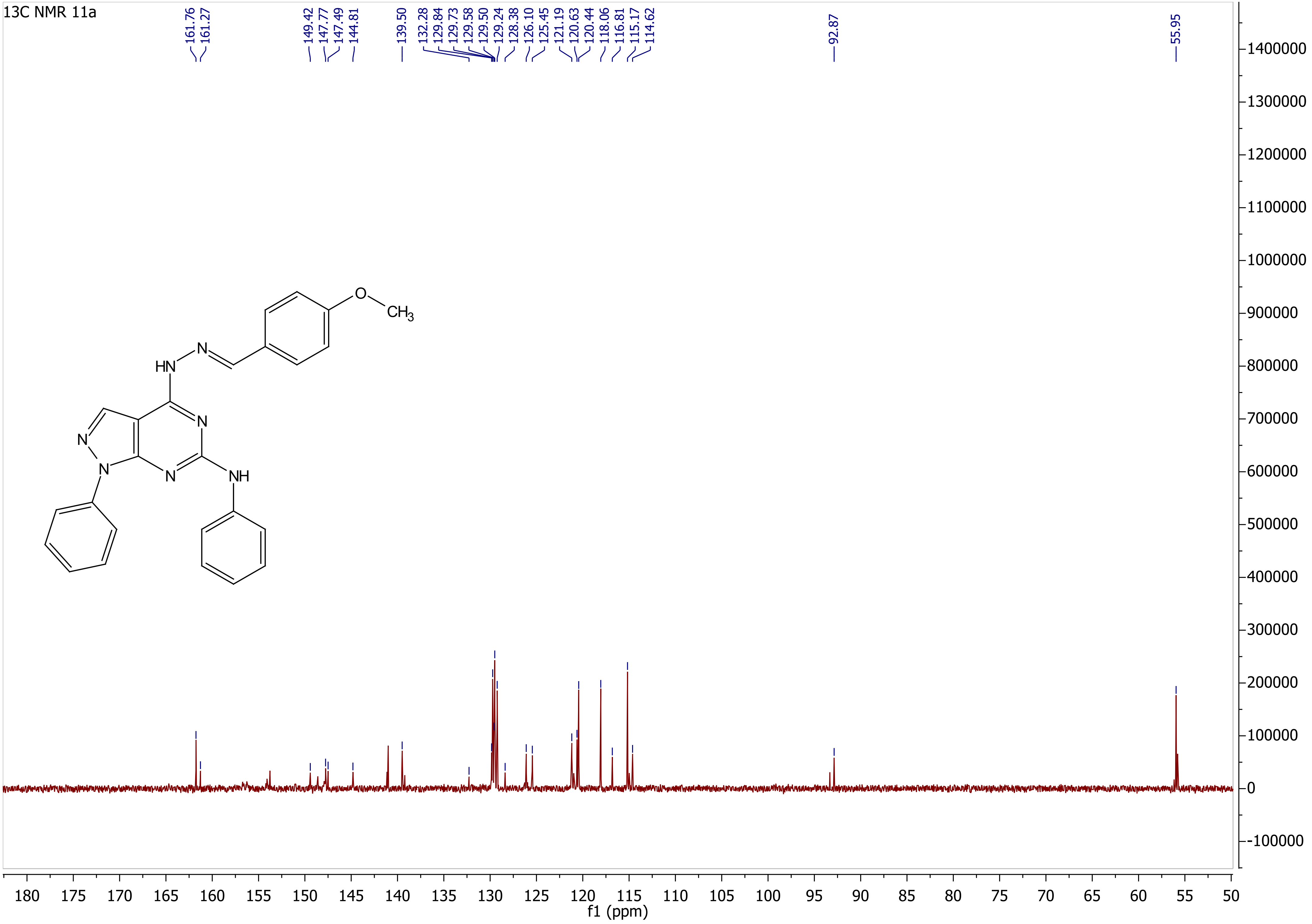

161.76
161.27
149.42
147.77
147.49
144.81
139.50
132.28
129.84
129.73
129.58
129.50
129.24
128.38
126.10
125.45
121.19
120.63
120.44
118.06
116.81
115.17
114.62
92.87
55.95
O
CH3
HN
N
N
N
N
N
NH
f1 (ppm)

DEPT13C NMR 11a

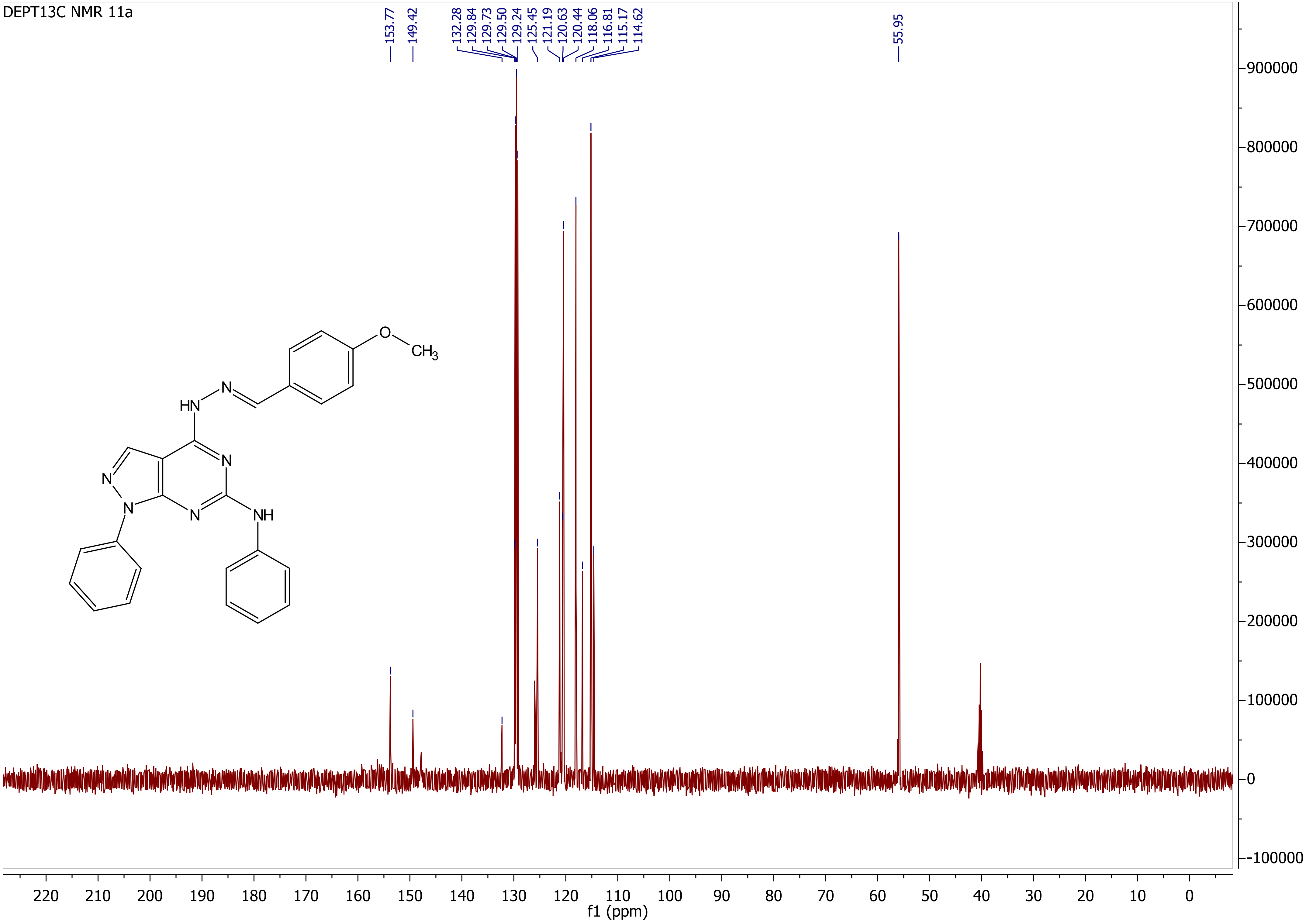

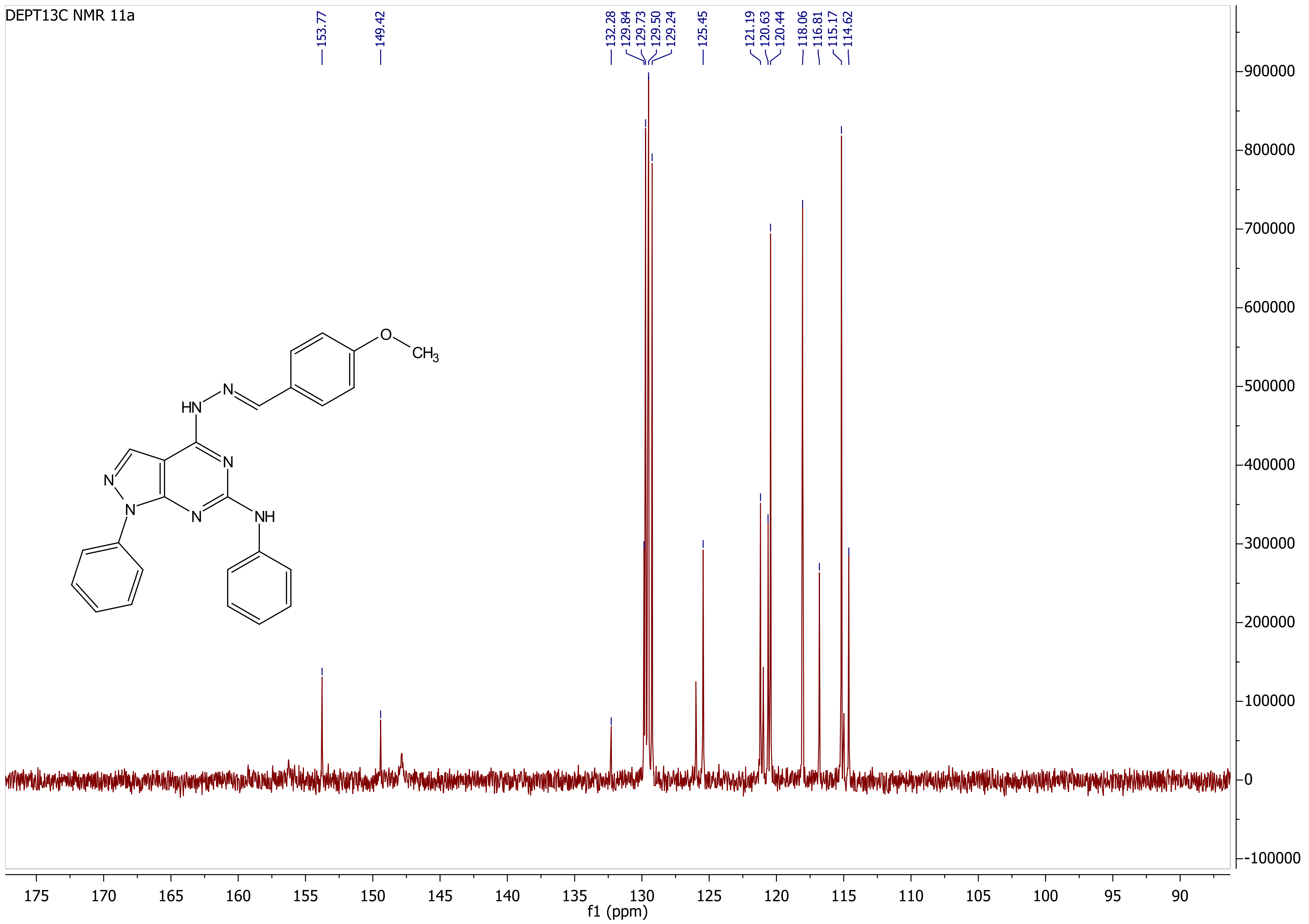
DEPT13C NMR 11a
153.77
149.42
132.28
129.84
129.73
129.50
129.24
125.45
121.19
120.63
120.44
118.06
116.81
115.17
114.62
f1 (ppm)

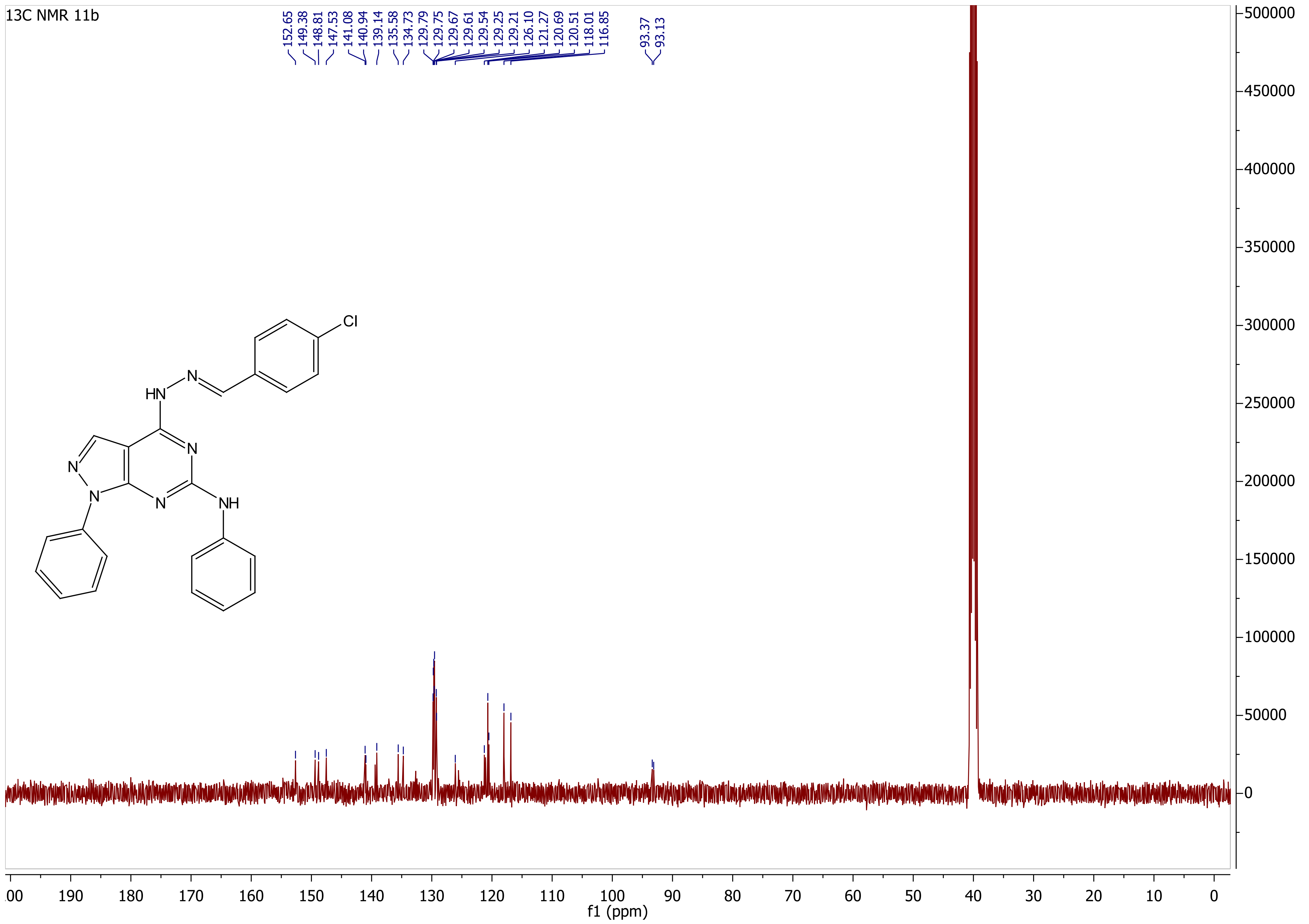
13C NMR 11b
152.65
149.38
148.81
147.53
141.08
140.94
139.14
135.58
134.73
129.79
129.75
129.67
129.61
129.54
129.25
129.21
126.10
121.27
120.69
120.51
118.01
116.85
93.37
93.13
Cl
N
HN
N
N
N
N
NH
f1 (ppm)

13C NMR 11b

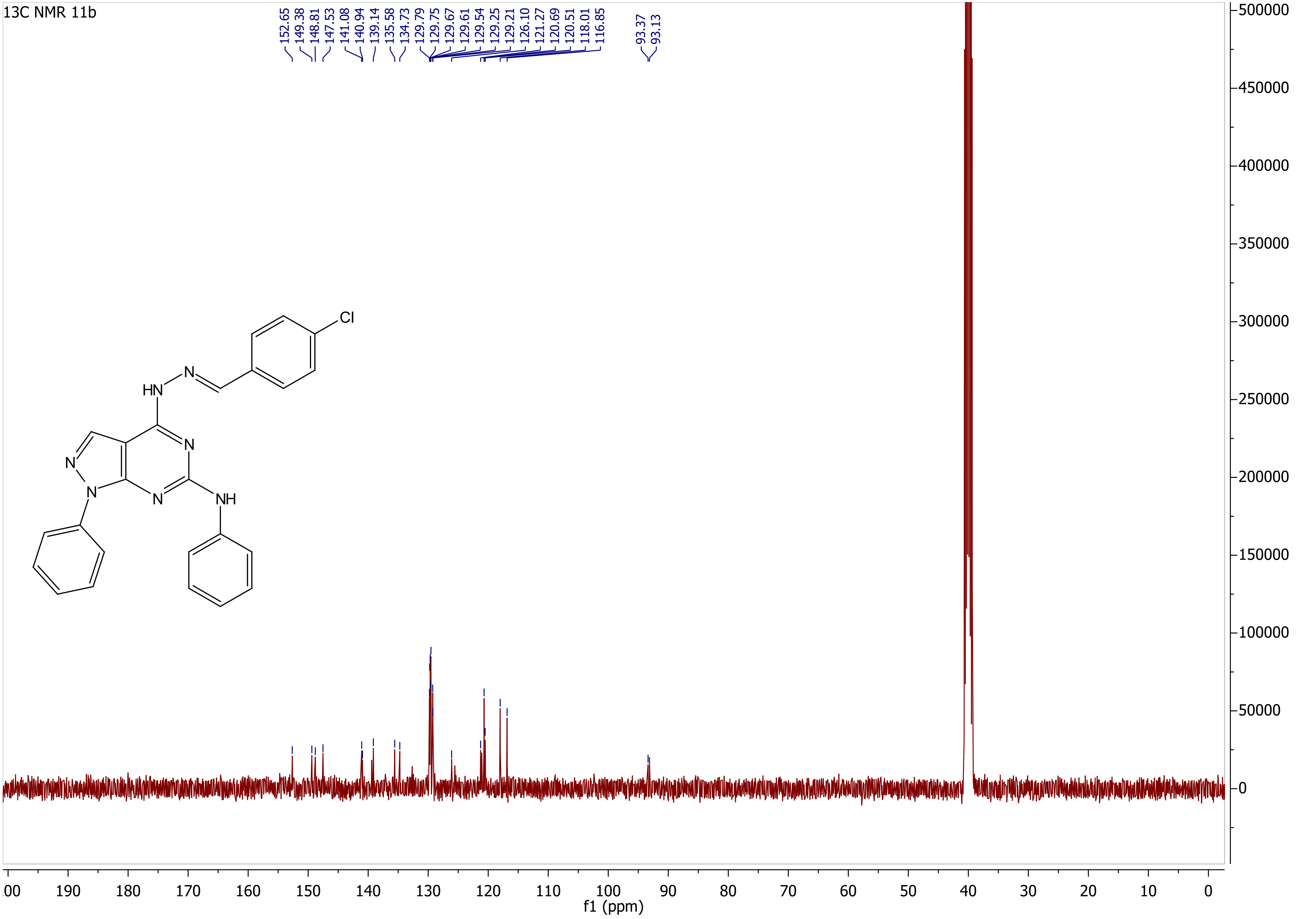

152.65
149.38
148.81
147.53
141.08
140.94
139.14
135.58
134.73
129.79
129.75
129.67
129.61
129.54
129.25
129.21
126.10
121.27
120.69
120.51
118.01
116.85
93.37
93.13
Cl
N
HN
N
N
N
N
NH
500000
450000
400000
350000
300000
250000
200000
150000
100000
50000
0
00
190
180
170
160
150
140
130
120
110
100
90
80
70
60
50
40
30
20
10
0
f1 (ppm)

DEPT13C NMR 11b

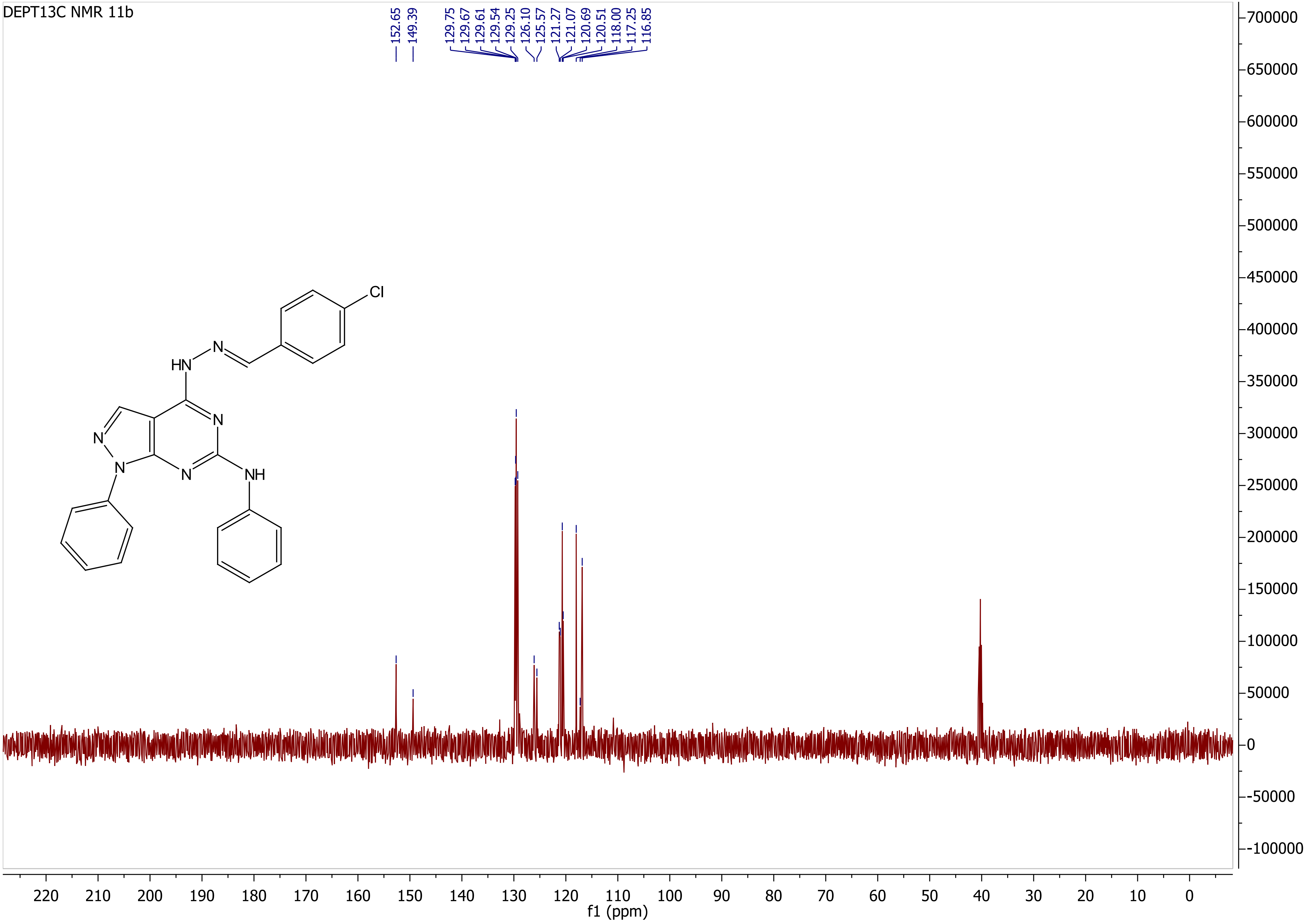

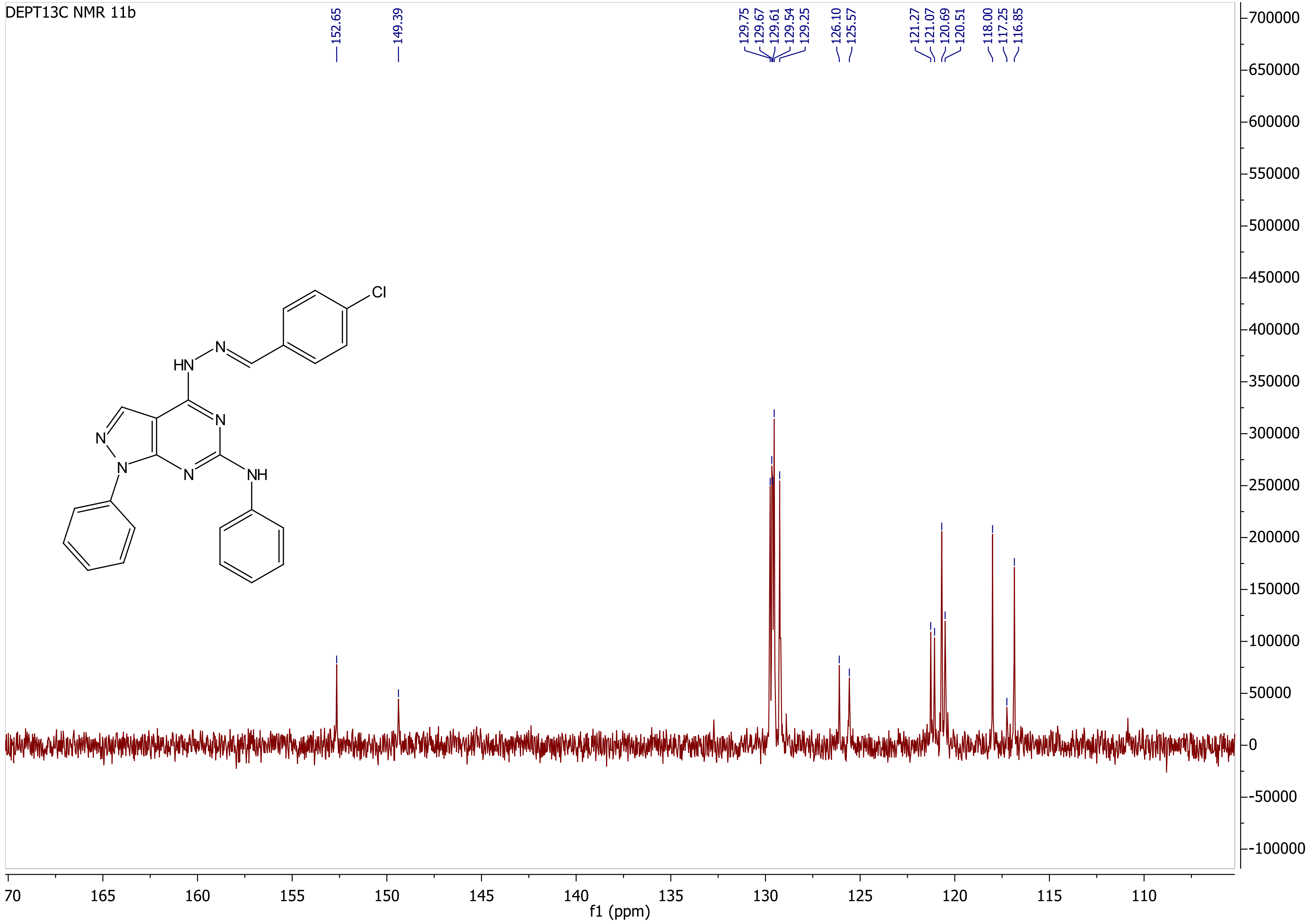
DEPT13C NMR 11b
152.65
149.39
129.75
129.67
129.61
129.54
129.25
126.10
125.57
121.27
121.07
120.69
120.51
118.00
117.25
116.85
Cl
N
HN
N
N
N
N
NH
f1 (ppm)

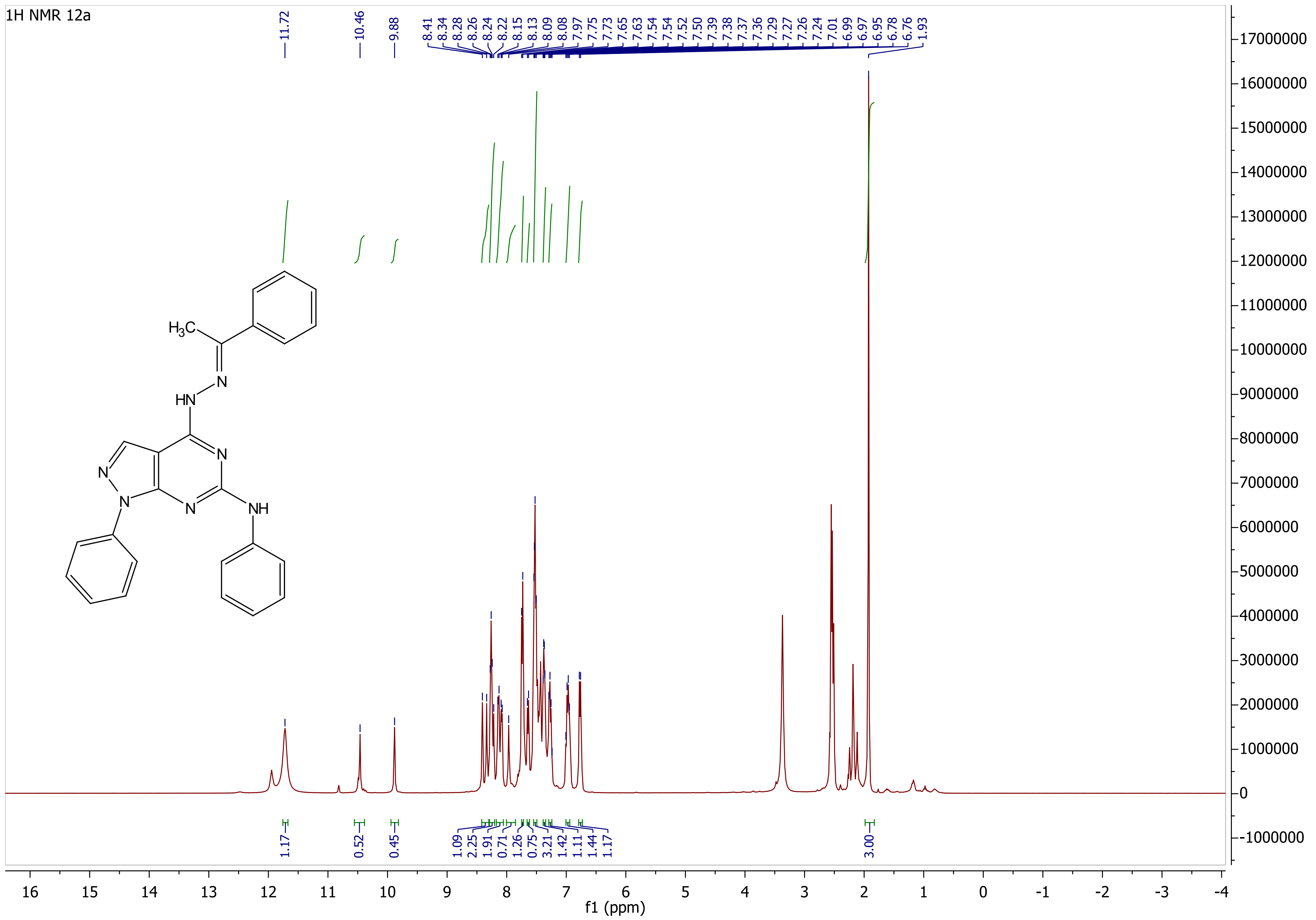
1H NMR 12a
11.72
10.46
9.88
8.41
8.34
8.28
8.26
8.24
8.22
8.15
8.13
8.09
8.08
7.97
7.75
7.73
7.65
7.63
7.54
7.54
7.52
7.50
7.39
7.38
7.37
7.36
7.29
7.27
7.26
7.24
7.01
6.99
6.97
6.95
6.78
6.76
1.93
H3C
N
HN
N
N
N
N
NH
1.17
0.52
0.45
1.09
2.25
1.91
0.71
1.26
0.75
3.21
1.42
1.11
1.44
1.17
3.00
f1 (ppm)

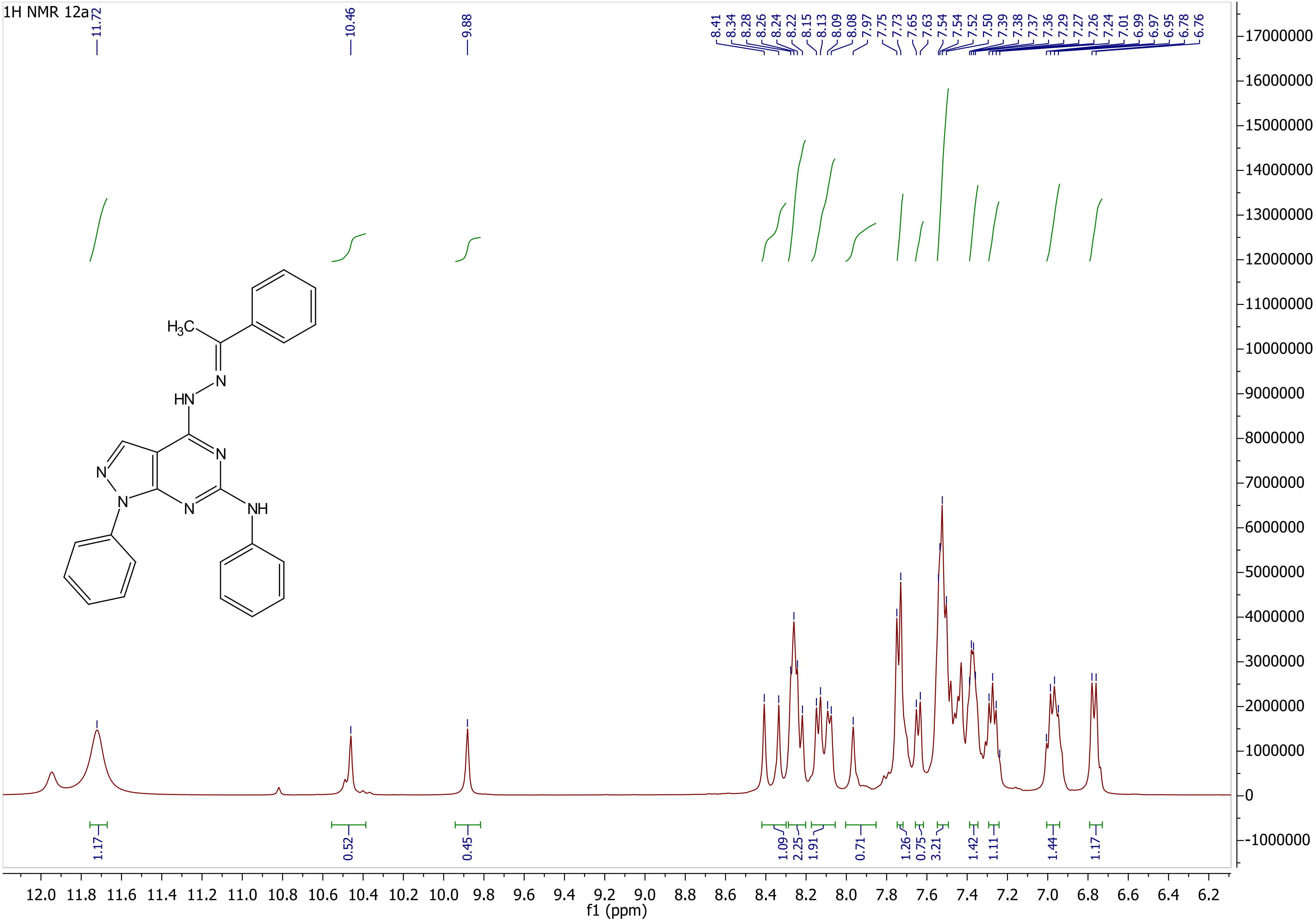
1H NMR 12a
11.72
10.46
9.88
8.41 8.34 8.28 8.26 8.24 8.22 8.15 8.13 8.09 8.08 7.97 7.75 7.73 7.65 7.63 7.54 7.54 7.52 7.50 7.39 7.38 7.37 7.36 7.29 7.27 7.26 7.24 7.01 6.99 6.97 6.95 6.78 6.76
1.17 0.52 0.45 1.09 2.25 1.91 0.71 1.26 0.75 3.21 1.42 1.11 1.44 1.17
f1 (ppm)
H3C
N
HN
N
N
N
N
NH

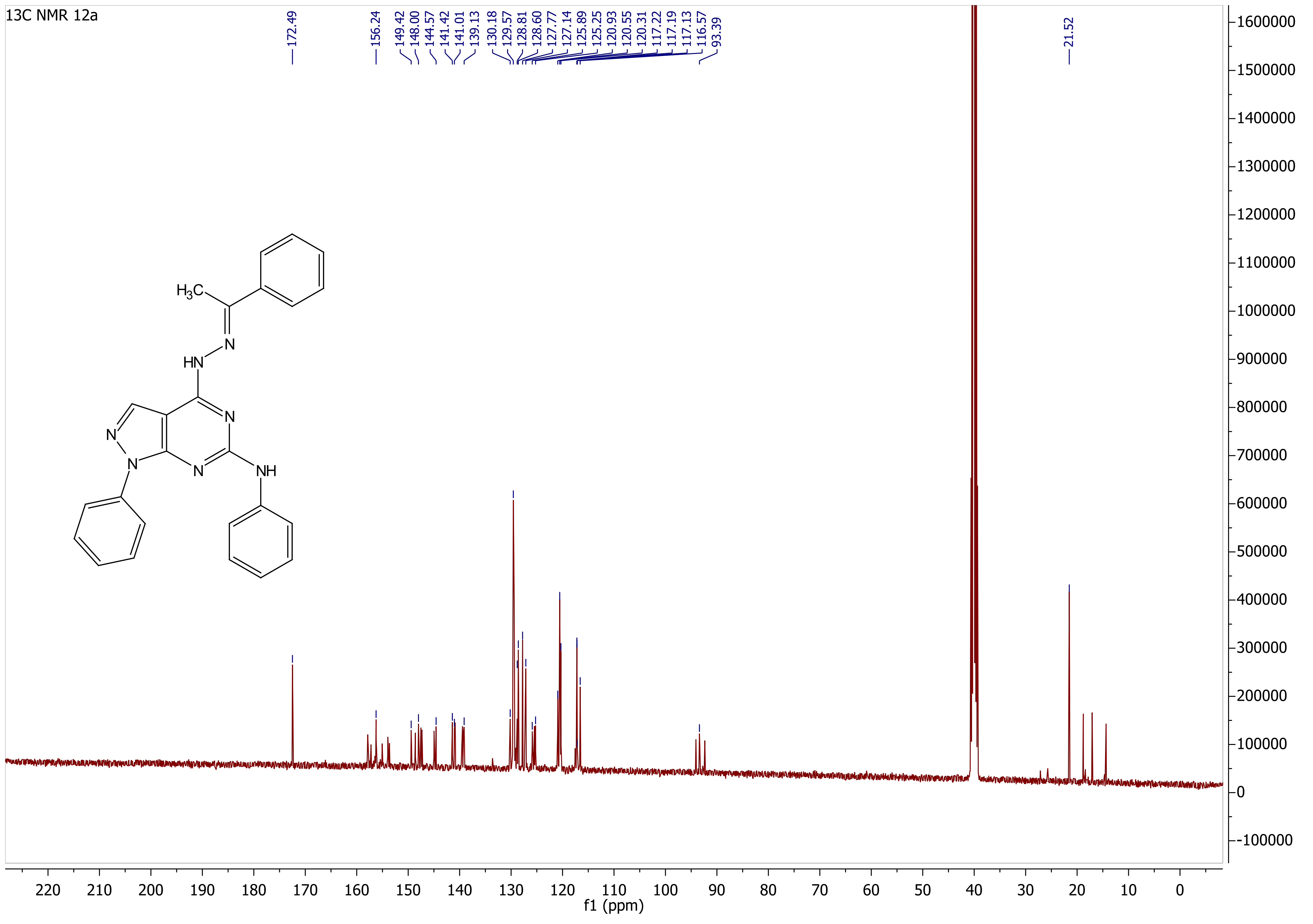
13C NMR 12a
172.49
156.24
149.42
148.00
144.57
141.42
141.01
139.13
130.18
129.57
128.81
128.60
127.77
127.14
125.89
125.25
120.93
120.55
120.31
117.22
117.19
117.13
116.57
93.39
21.52
H3C
N
HN
N
N
N
N
NH
f1 (ppm)

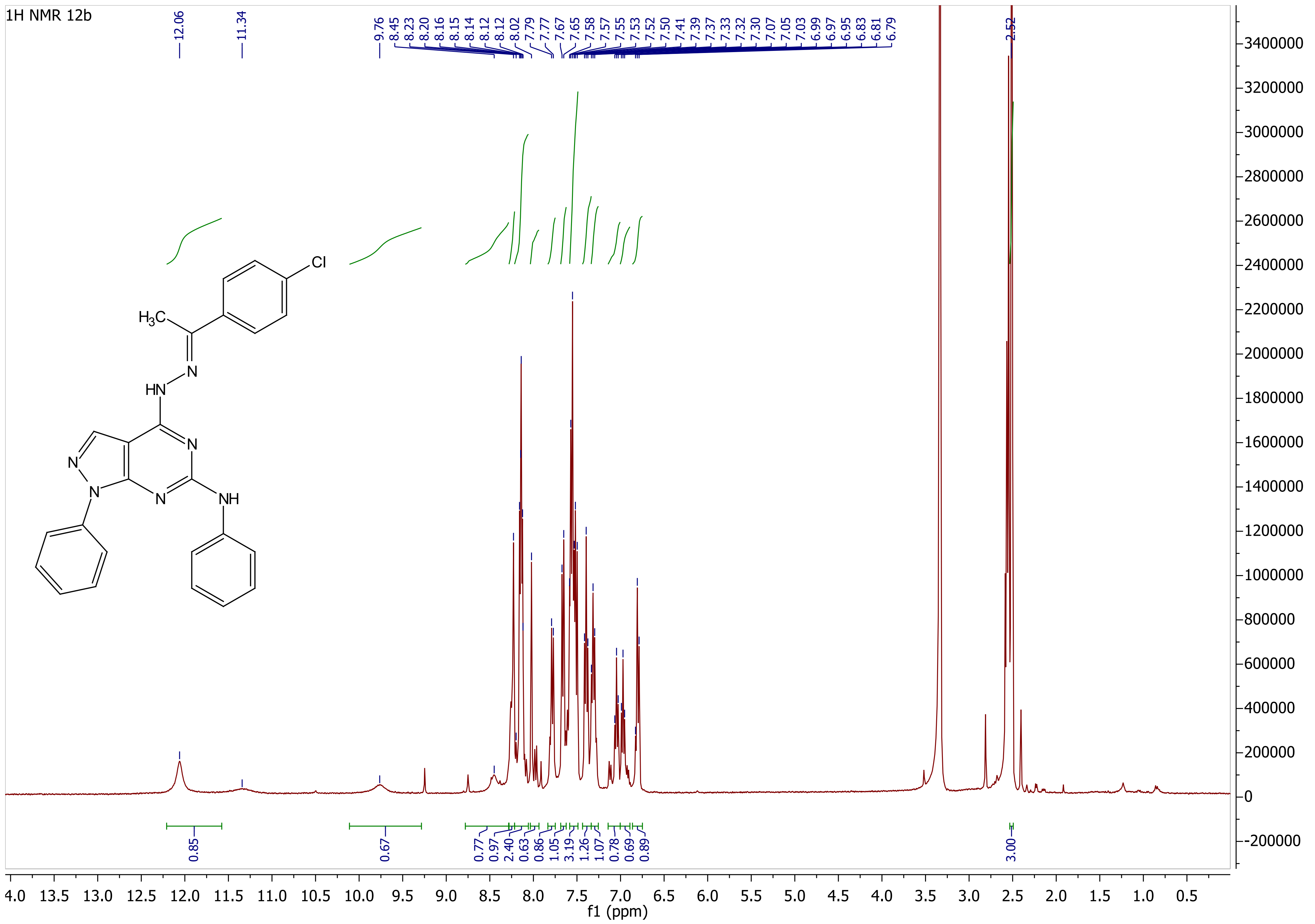
1H NMR 12b
12.06
11.34
9.76
8.45
8.23
8.20
8.16
8.15
8.14
8.12
8.12
8.02
7.79
7.77
7.67
7.65
7.58
7.57
7.55
7.53
7.52
7.50
7.41
7.39
7.37
7.33
7.32
7.30
7.07
7.05
7.03
6.99
6.97
6.95
6.83
6.81
6.79
2.52
Cl
H_3C
N
HN
N
N
N
N
NH
0.85
0.67
0.77
0.97
2.40
0.63
0.86
1.05
3.19
1.26
1.07
0.78
0.69
0.89
3.00
f1 (ppm)

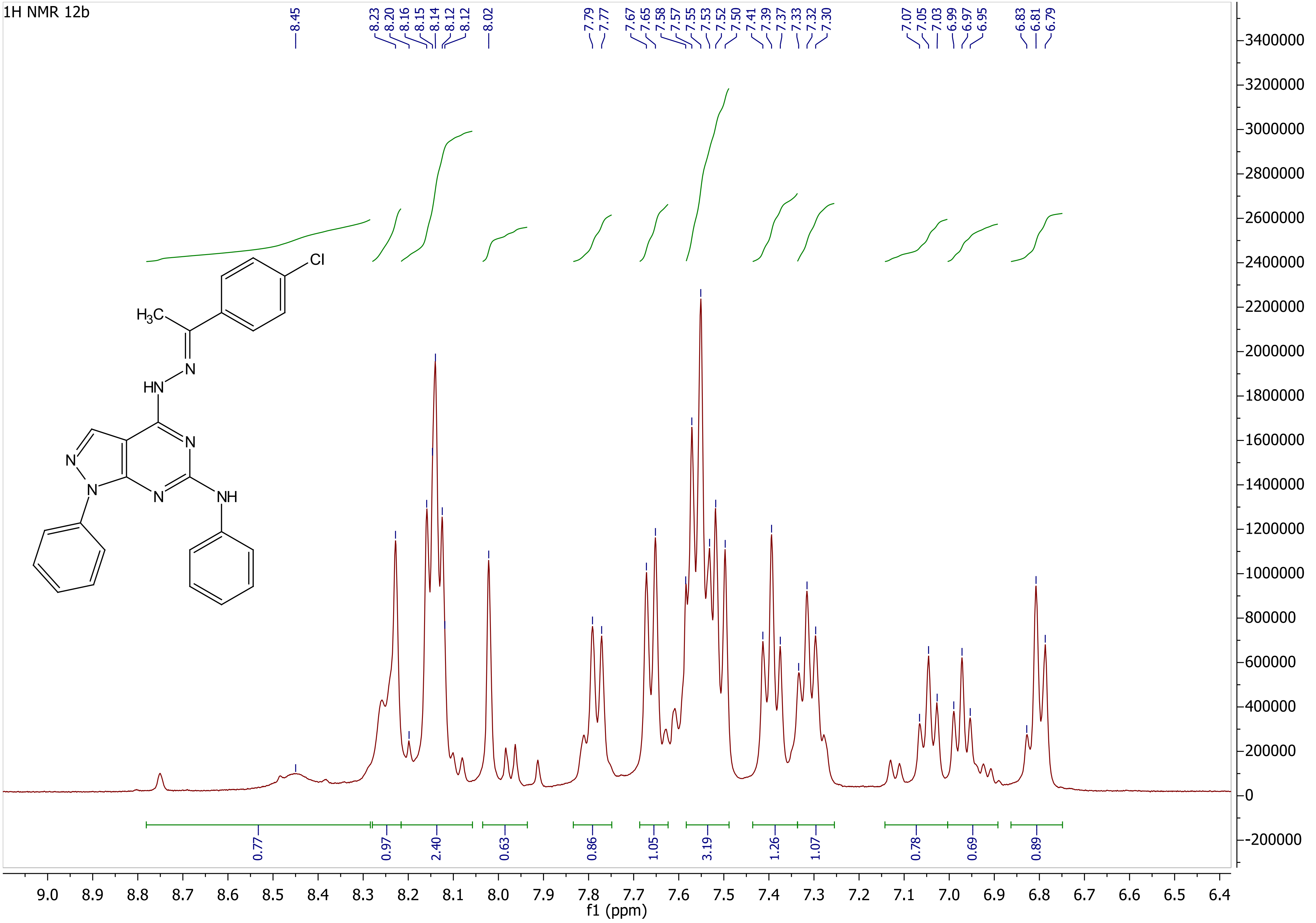
1H NMR 12b
8.45
8.23 8.20 8.16 8.15 8.14 8.12 8.12
8.02
7.79 7.77
7.67 7.65 7.58 7.57 7.55 7.53 7.52 7.50 7.41 7.39 7.37 7.33 7.32 7.30
7.07 7.05 7.03 6.99 6.97 6.95
6.83 6.81 6.79
0.77 0.97 2.40 0.63 0.86 1.05 3.19 1.26 1.07 0.78 0.69 0.89
Cl
H3C
N
HN
N
N
N
N
NH
9.0 8.9 8.8 8.7 8.6 8.5 8.4 8.3 8.2 8.1 8.0 7.9 7.8 7.7 7.6 7.5 7.4 7.3 7.2 7.1 7.0 6.9 6.8 6.7 6.6 6.5 6.4
f1 (ppm)
3400000 3200000 3000000 2800000 2600000 2400000 2200000 2000000 1800000 1600000 1400000 1200000 1000000 800000 600000 400000 200000 0 -200000

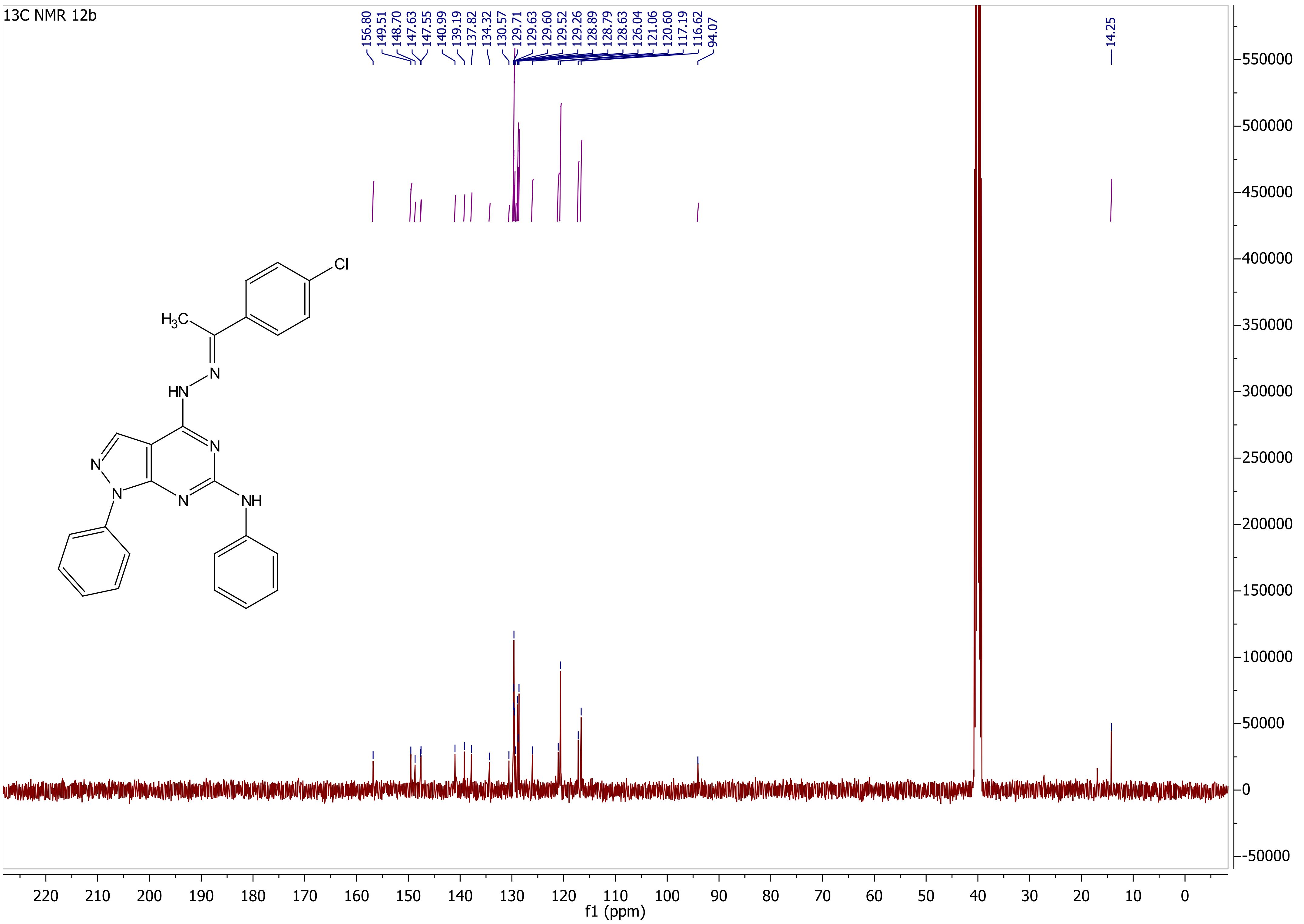
13C NMR 12b
156.80
149.51
148.70
147.63
147.55
140.99
139.19
137.82
134.32
130.57
129.71
129.63
129.60
129.52
129.26
128.89
128.79
128.63
126.04
121.06
120.60
117.19
116.62
94.07
14.25
Cl
H3C
N
HN
N
N
N
NH
N
550000
500000
450000
400000
350000
300000
250000
200000
150000
100000
50000
0
-50000
220
210
200
190
180
170
160
150
140
130
120
110
100
90
80
70
60
50
40
30
20
10
0
f1 (ppm)

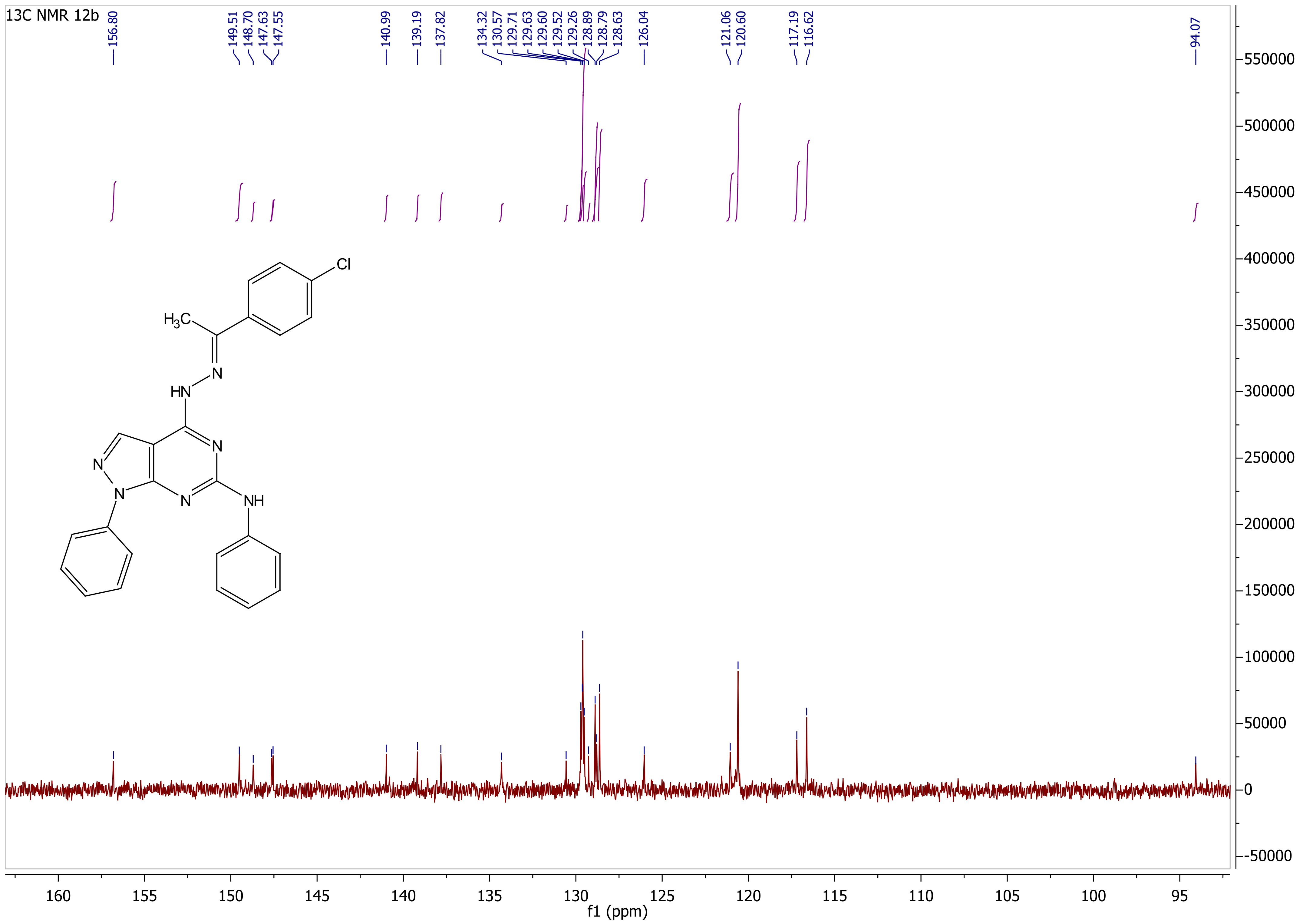
13C NMR 12b
156.80
149.51
148.70
147.63
147.55
140.99
139.19
137.82
134.32
130.57
129.71
129.63
129.60
129.52
129.26
128.89
128.79
128.63
126.04
121.06
120.60
117.19
116.62
94.07
Cl
H3C
N
HN
N
N
N
N
NH
f1 (ppm)

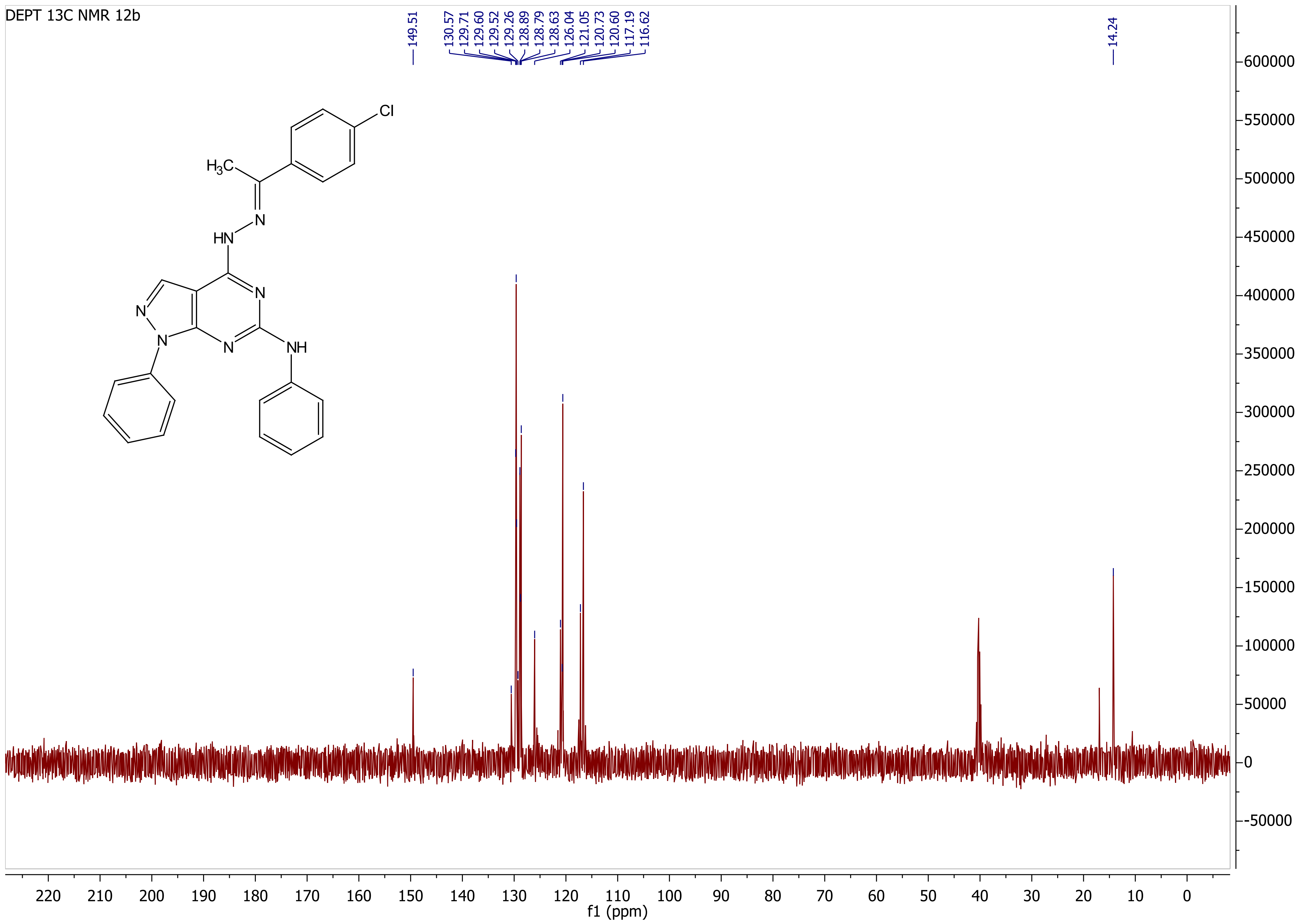
DEPT 13C NMR 12b
149.51
130.57
129.71
129.60
129.52
129.26
128.89
128.79
128.63
126.04
121.05
120.73
120.60
117.19
116.62
14.24
Cl
H3C
N
HN
N
N
N
N
NH
600000
550000
500000
450000
400000
350000
300000
250000
200000
150000
100000
50000
0
-50000
220 210 200 190 180 170 160 150 140 130 120 110 100 90 80 70 60 50 40 30 20 10 0
f1 (ppm)

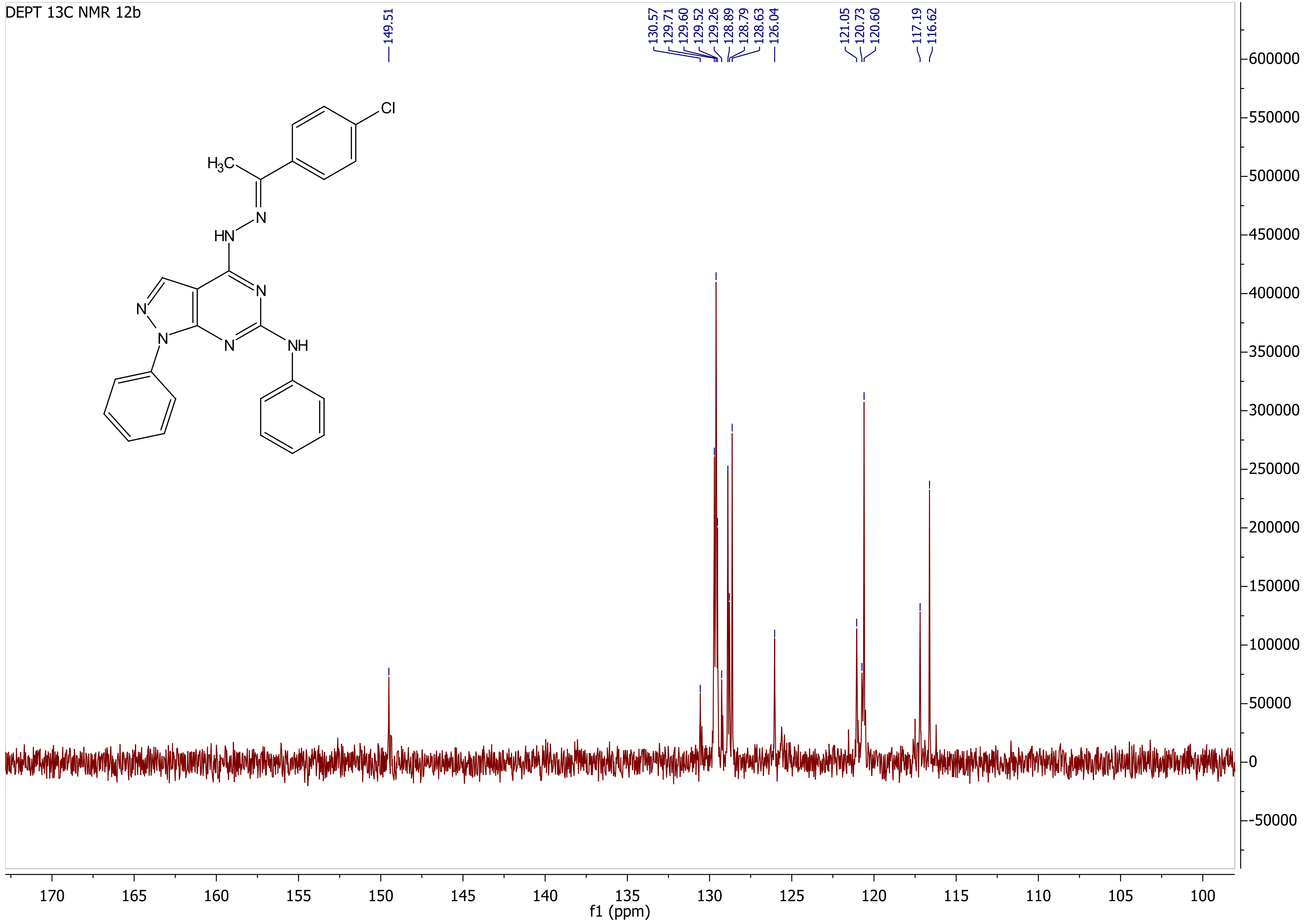
DEPT 13C NMR 12b
149.51
130.57
129.71
129.60
129.52
129.26
128.89
128.79
128.63
126.04
121.05
120.73
120.60
117.19
116.62
Cl
H3C
N
HN
N
N
N
N
NH
600000
550000
500000
450000
400000
350000
300000
250000
200000
150000
100000
50000
0
-50000
170
165
160
155
150
145
140
135
130
125
120
115
110
105
100
f1 (ppm)

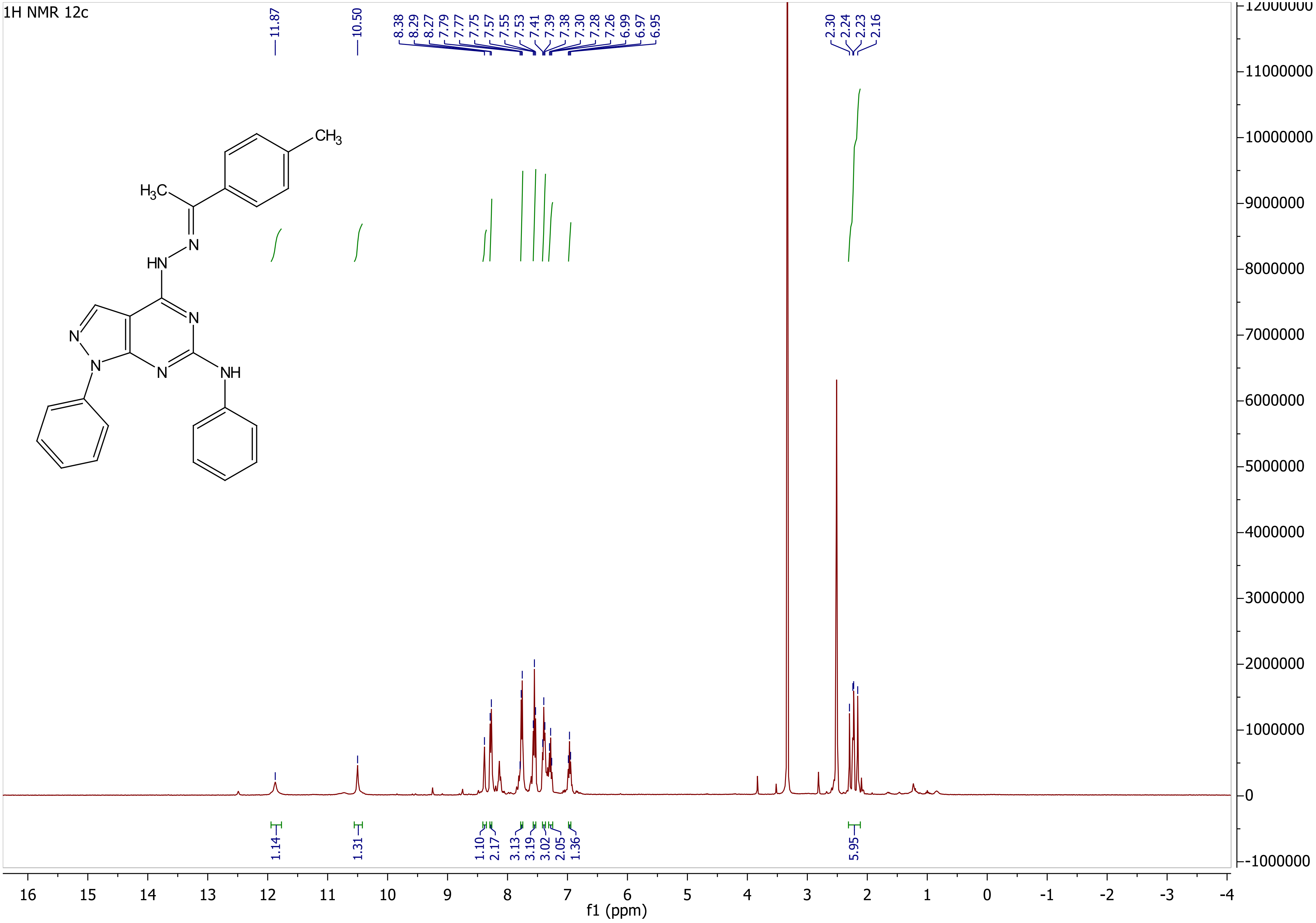
1H NMR 12c
11.87
10.50
8.38 8.29 8.27 7.79 7.77 7.75 7.57 7.55 7.53 7.41 7.39 7.38 7.30 7.28 7.26 6.99 6.97 6.95
2.30 2.24 2.23 2.16
CH3
H3C
N
HN
N
N
N
N
NH
1.14
1.31
1.10 2.17 3.13 3.19 3.02 2.05 1.36
5.95
f1 (ppm)
16 15 14 13 12 11 10 9 8 7 6 5 4 3 2 1 0 -1 -2 -3 -4
12000000 11000000 10000000 9000000 8000000 7000000 6000000 5000000 4000000 3000000 2000000 1000000 0 -1000000

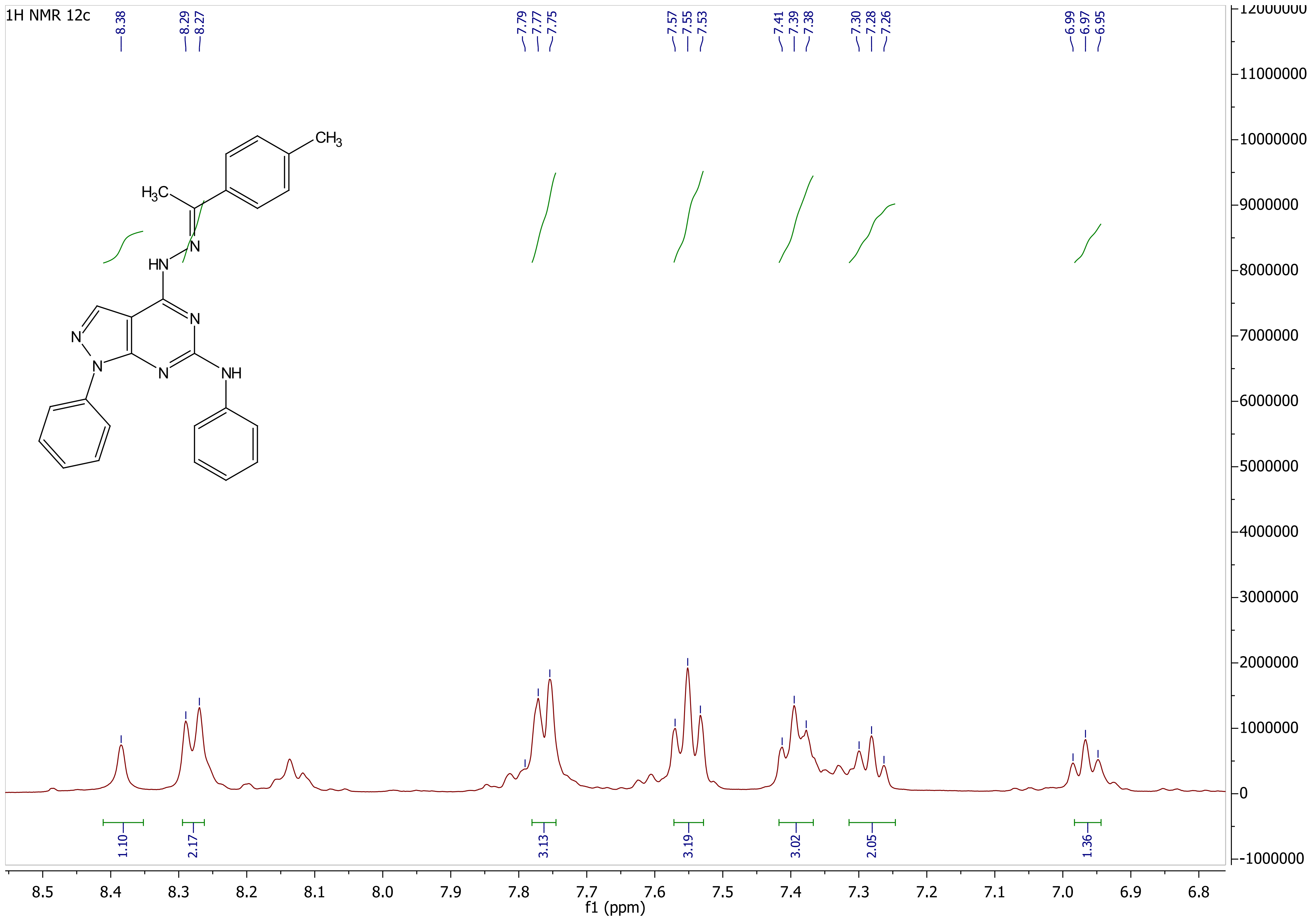
1H NMR 12c
8.38
8.29
8.27
7.79
7.77
7.75
7.57
7.55
7.53
7.41
7.39
7.38
7.30
7.28
7.26
6.99
6.97
6.95
CH3
H3C
N
HN
N
N
N
N
NH
1.10
2.17
3.13
3.19
3.02
2.05
1.36
f1 (ppm)

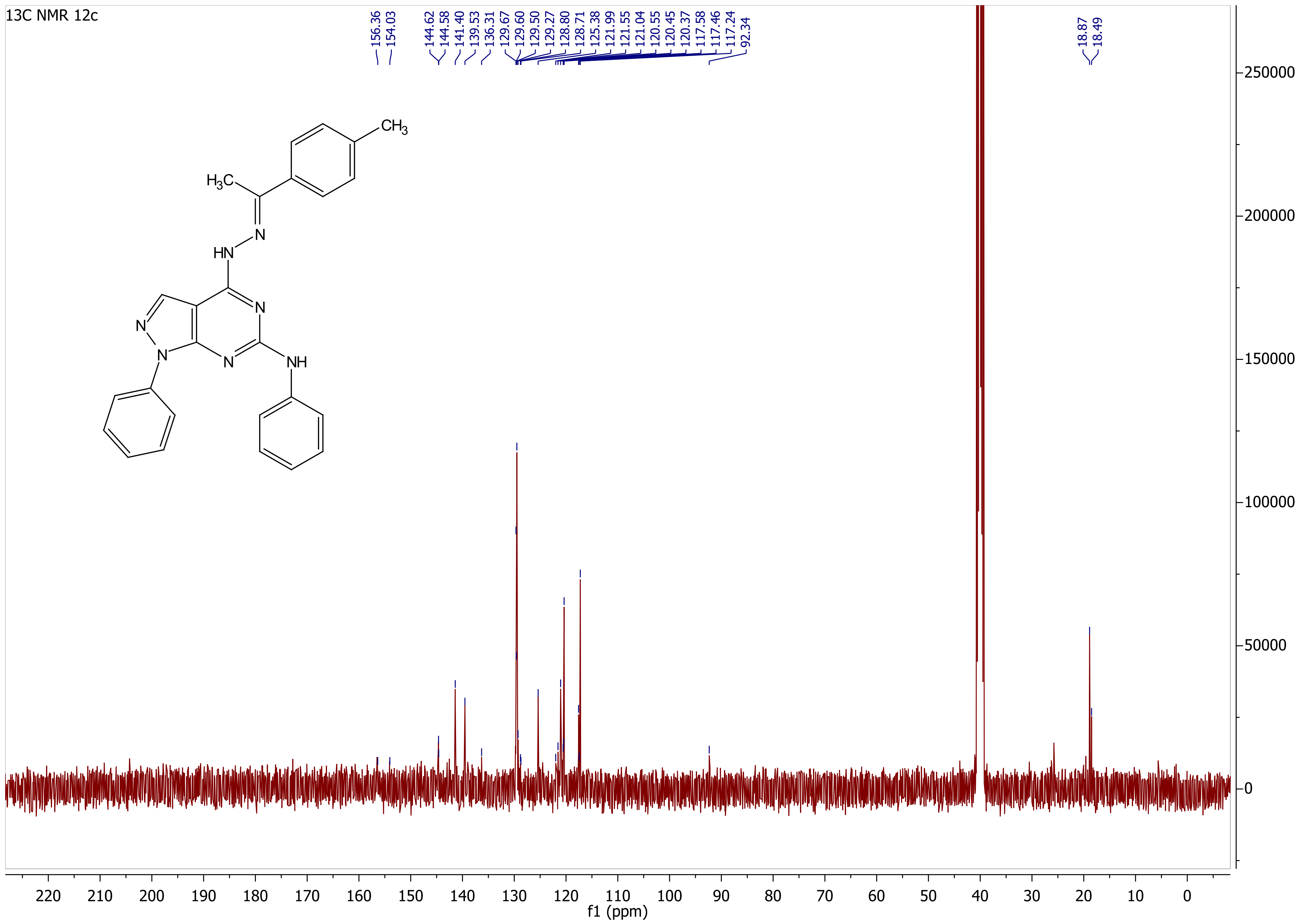
13C NMR 12c
156.36
154.03
144.62
144.58
141.40
139.53
136.31
129.67
129.60
129.50
129.27
128.80
128.71
125.38
121.99
121.55
121.04
120.55
120.45
120.37
117.58
117.46
117.24
92.34
18.87
18.49
CH3
H3C
N
HN
N
N
N
NH
N
f1 (ppm)

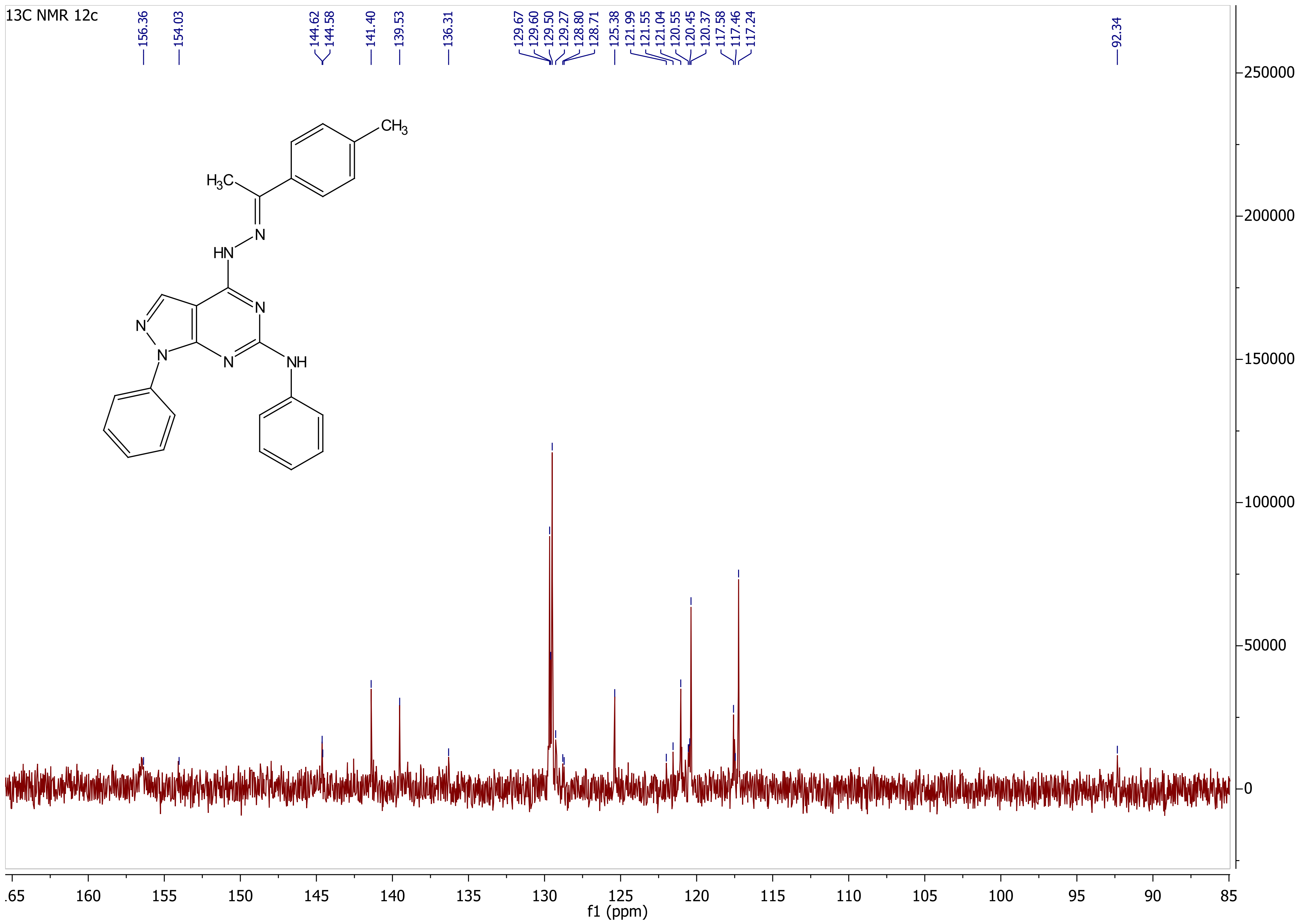
13C NMR 12c
156.36
154.03
144.62
144.58
141.40
139.53
136.31
129.67
129.60
129.50
129.27
128.80
128.71
125.38
121.99
121.55
121.04
120.55
120.45
120.37
117.58
117.46
117.24
92.34
CH3
H3C
N
HN
N
N
N
N
NH
250000
200000
150000
100000
50000
0
.65
160
155
150
145
140
135
130
125
120
115
110
105
100
95
90
85
f1 (ppm)

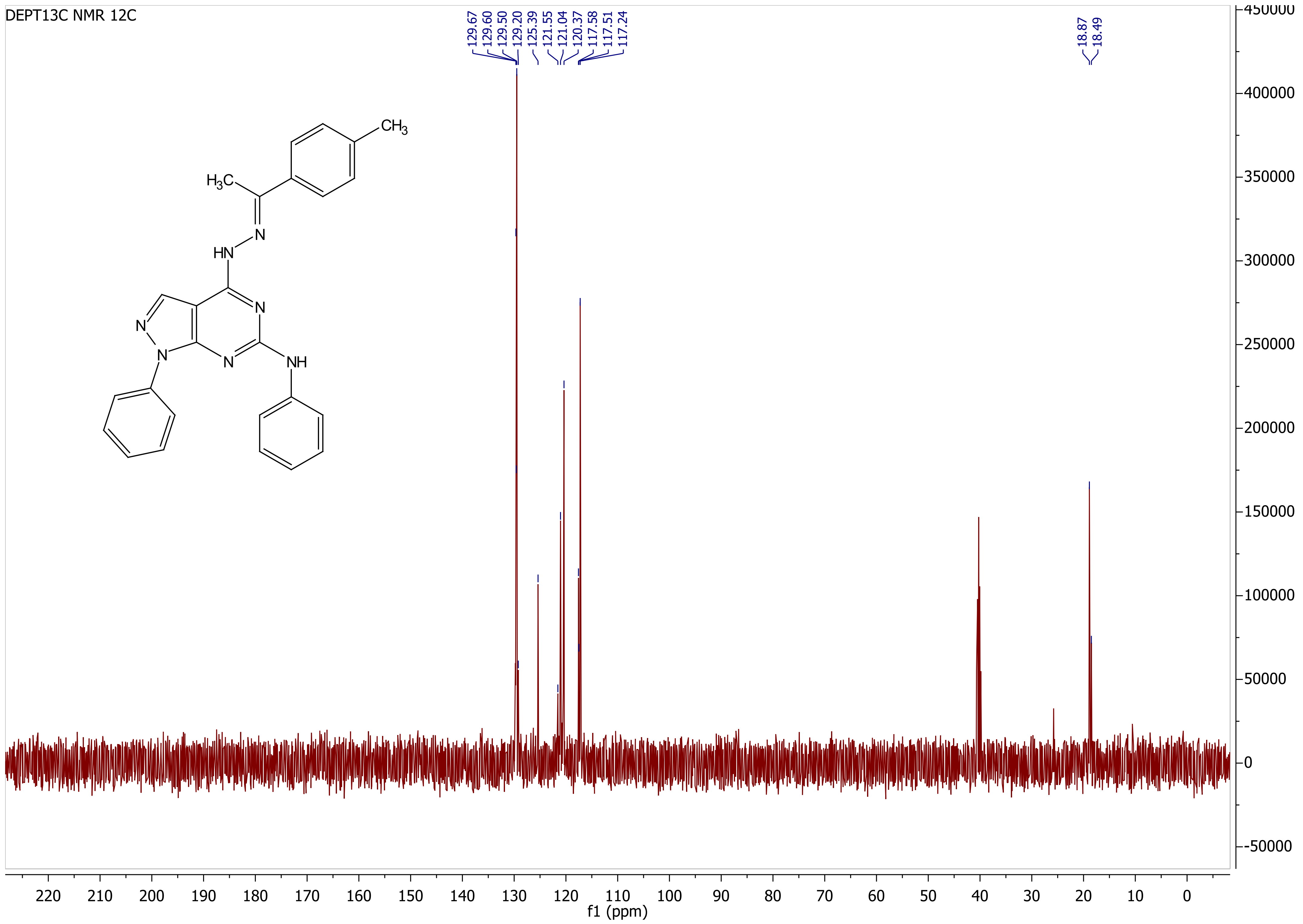
DEPT13C NMR 12C
129.67
129.60
129.50
129.20
125.39
121.55
121.04
120.37
117.58
117.51
117.24
18.87
18.49
CH_3
H_3C
N
HN
N
N
N
N
NH
f1 (ppm)

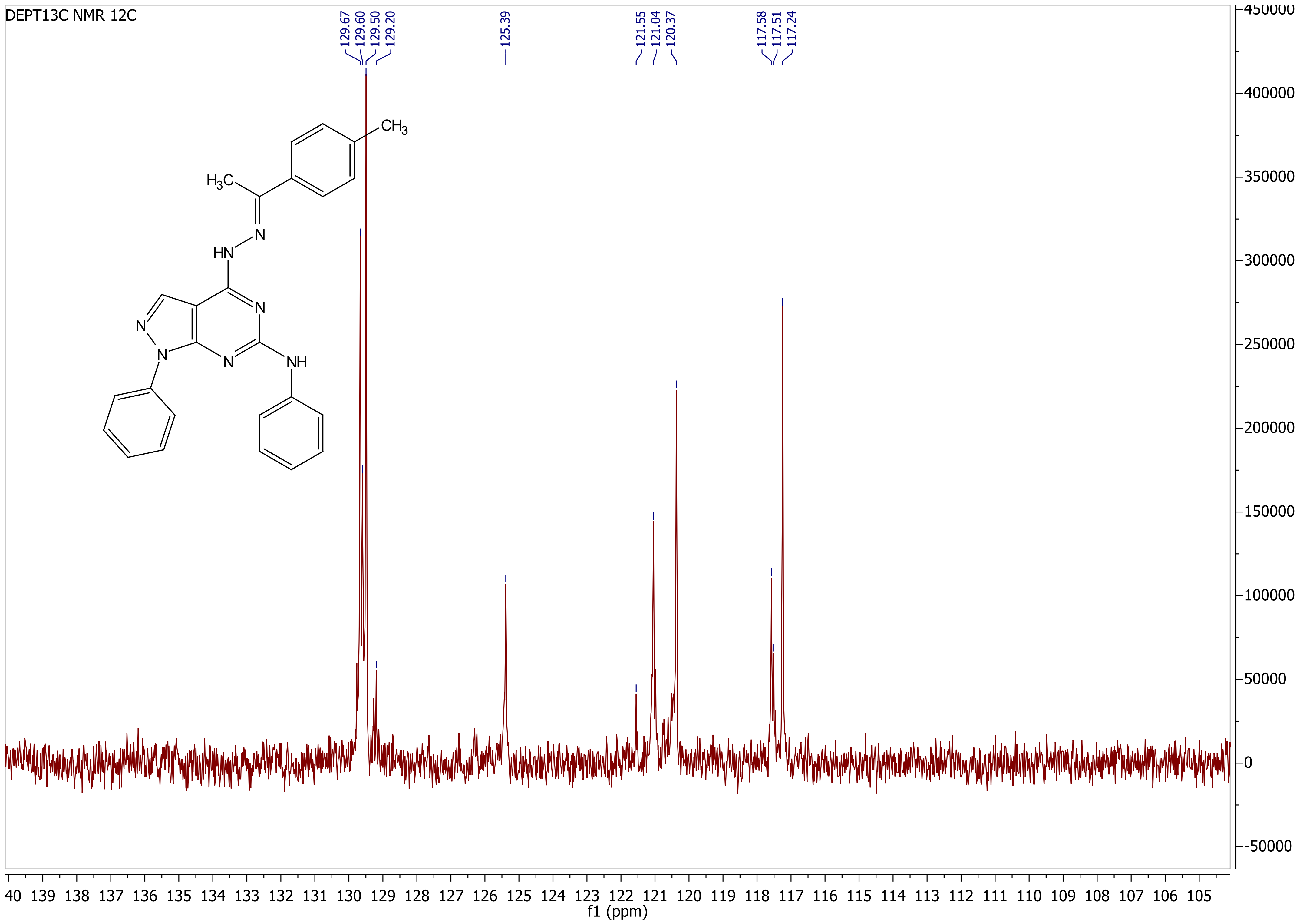

DEPT13C NMR 12C
129.67
129.60
129.50
129.20
125.39
121.55
121.04
120.37
117.58
117.51
117.24
CH3
H3C
N
HN
N
N
N
N
N
NH
450000
400000
350000
300000
250000
200000
150000
100000
50000
0
-50000
140 139 138 137 136 135 134 133 132 131 130 129 128 127 126 125 124 123 122 121 120 119 118 117 116 115 114 113 112 111 110 109 108 107 106 105
f1 (ppm)

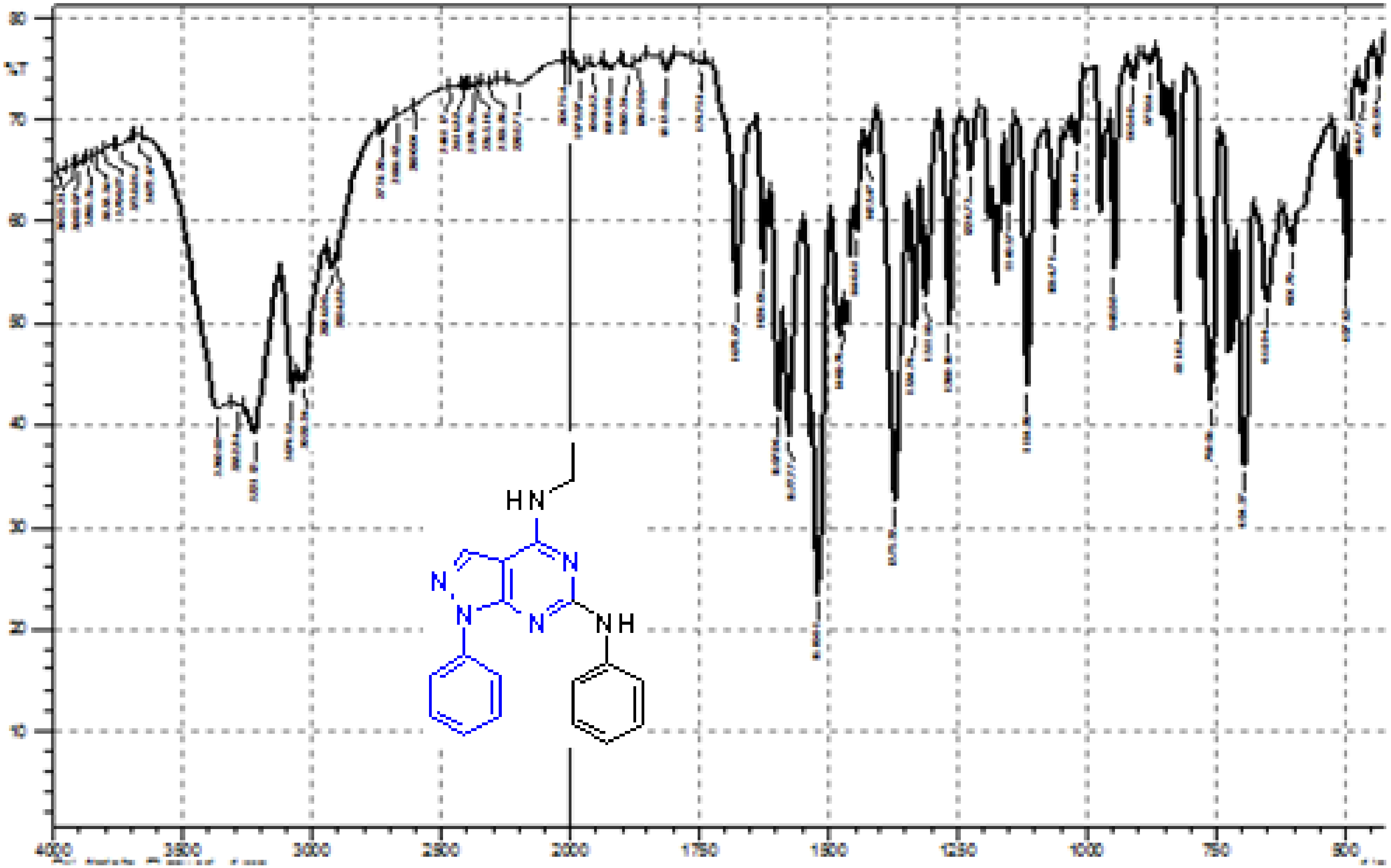
80
%T
70
60
50
40
30
20
10
4000
3500
3000
2500
2000
1750
1500
1250
1000
750
500
HN
N
N
N
N
N
NH

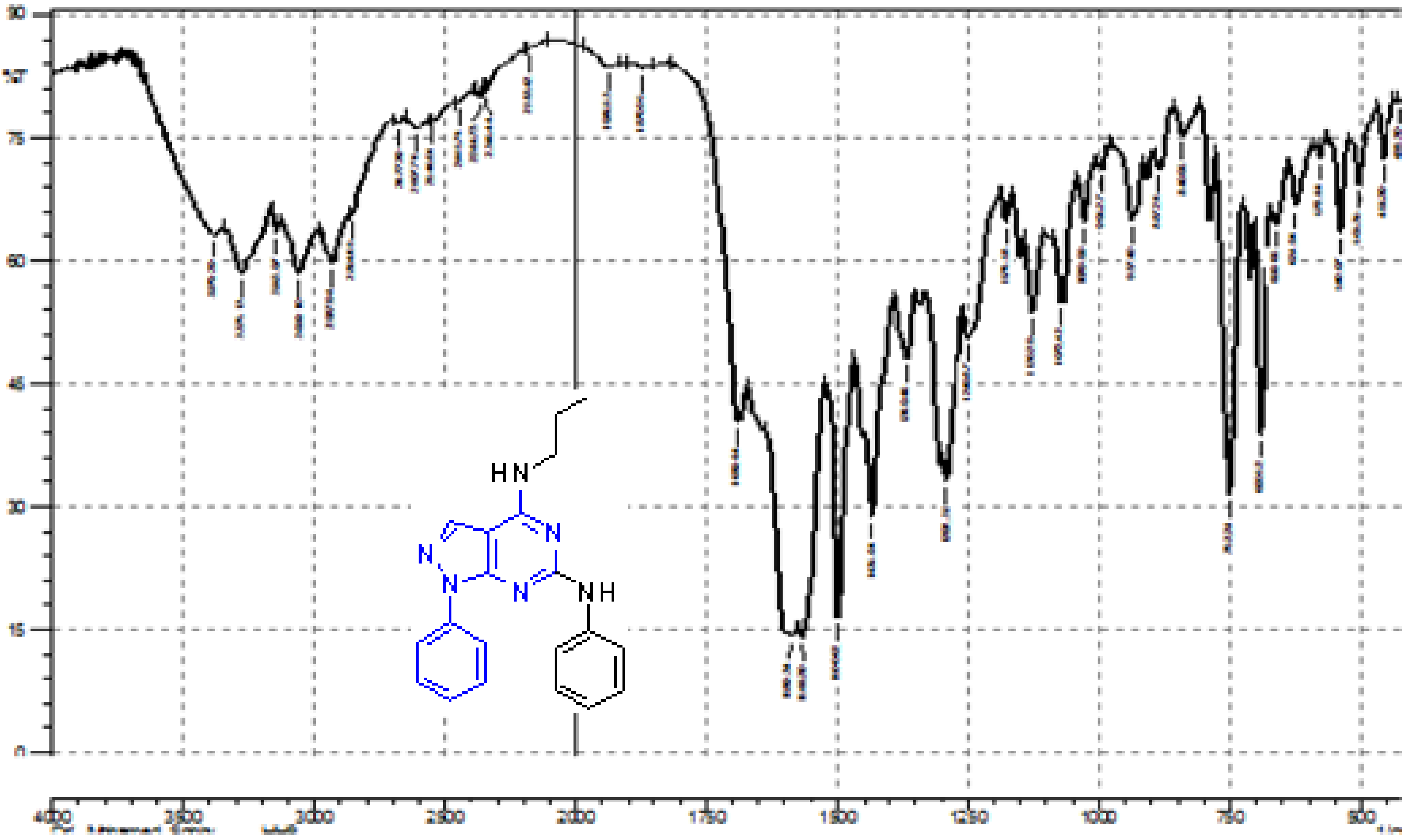
90
75
60
45
30
15
0
4000
3500
3000
2500
2000
1750
1500
1250
1000
750
500
HN
N
N
N
N
NH

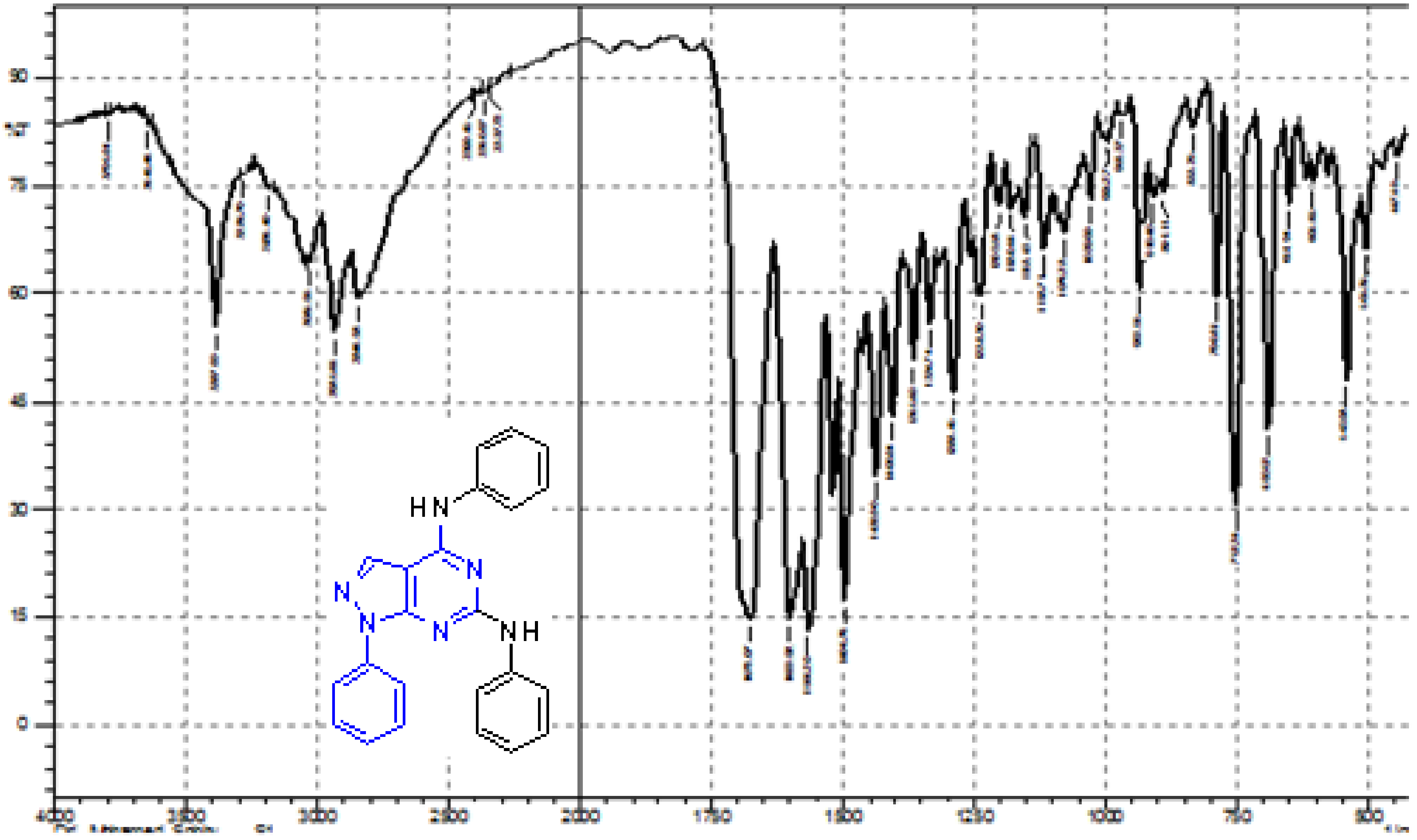

HN
N
N
N
N
NH

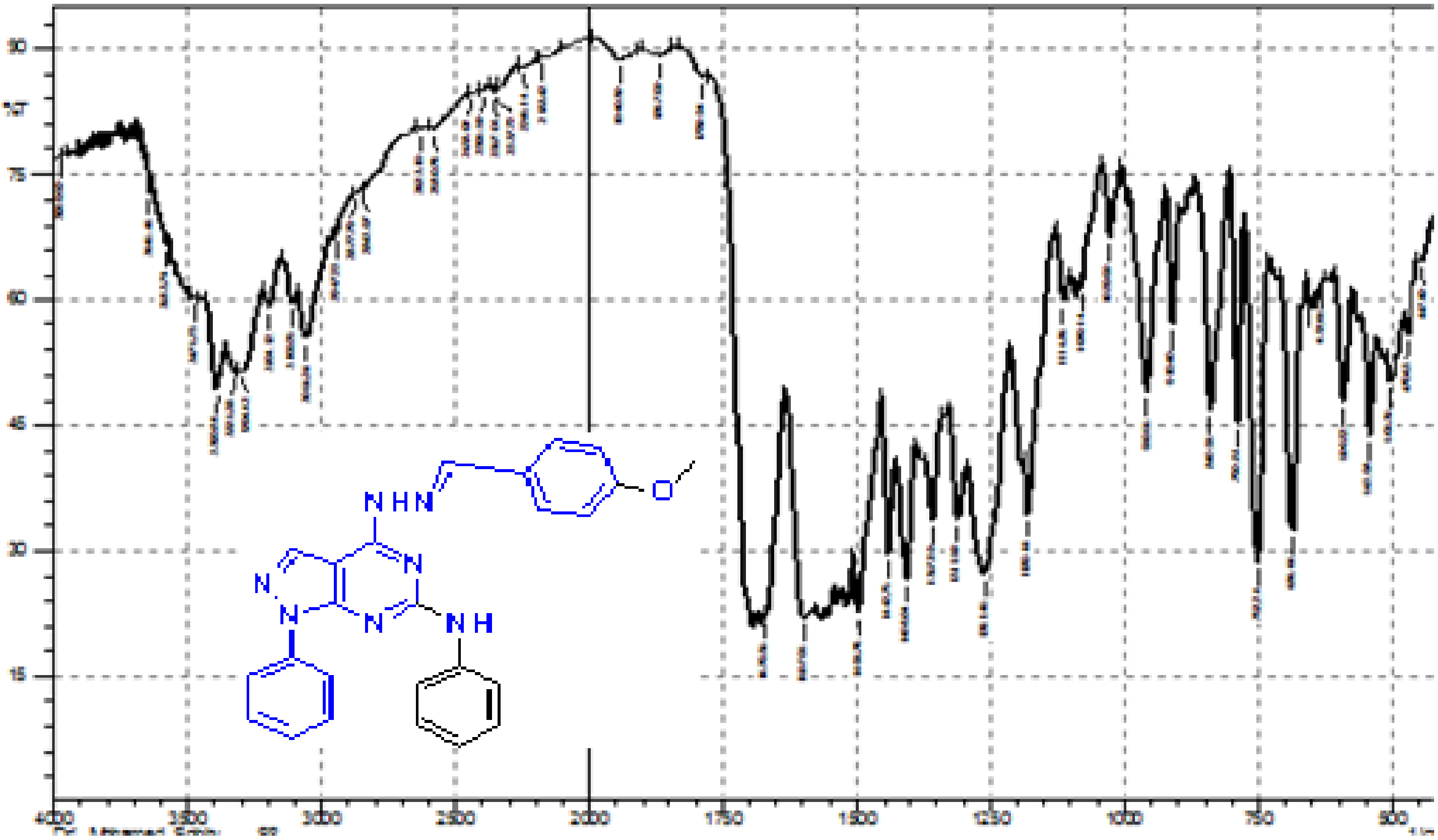
90
75
60
45
30
15
4000
3500
3000
2500
2000
1750
1500
1250
1000
750
500
NHN
N
N
N
N
NH
O

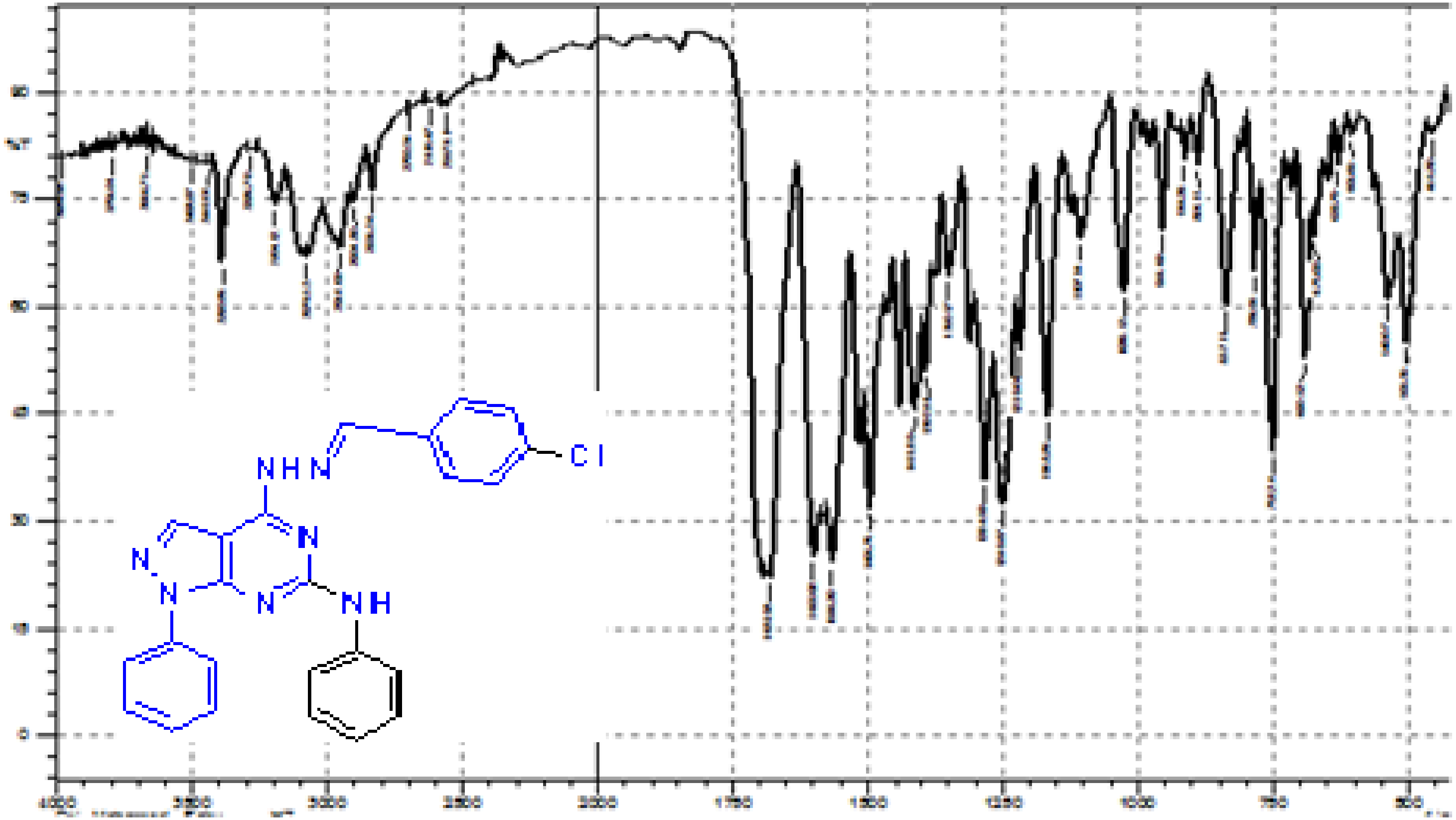
4000
3500
3000
2500
2000
1750
1500
1250
1000
750
500
NHN
Cl
N
N
N
N
NH

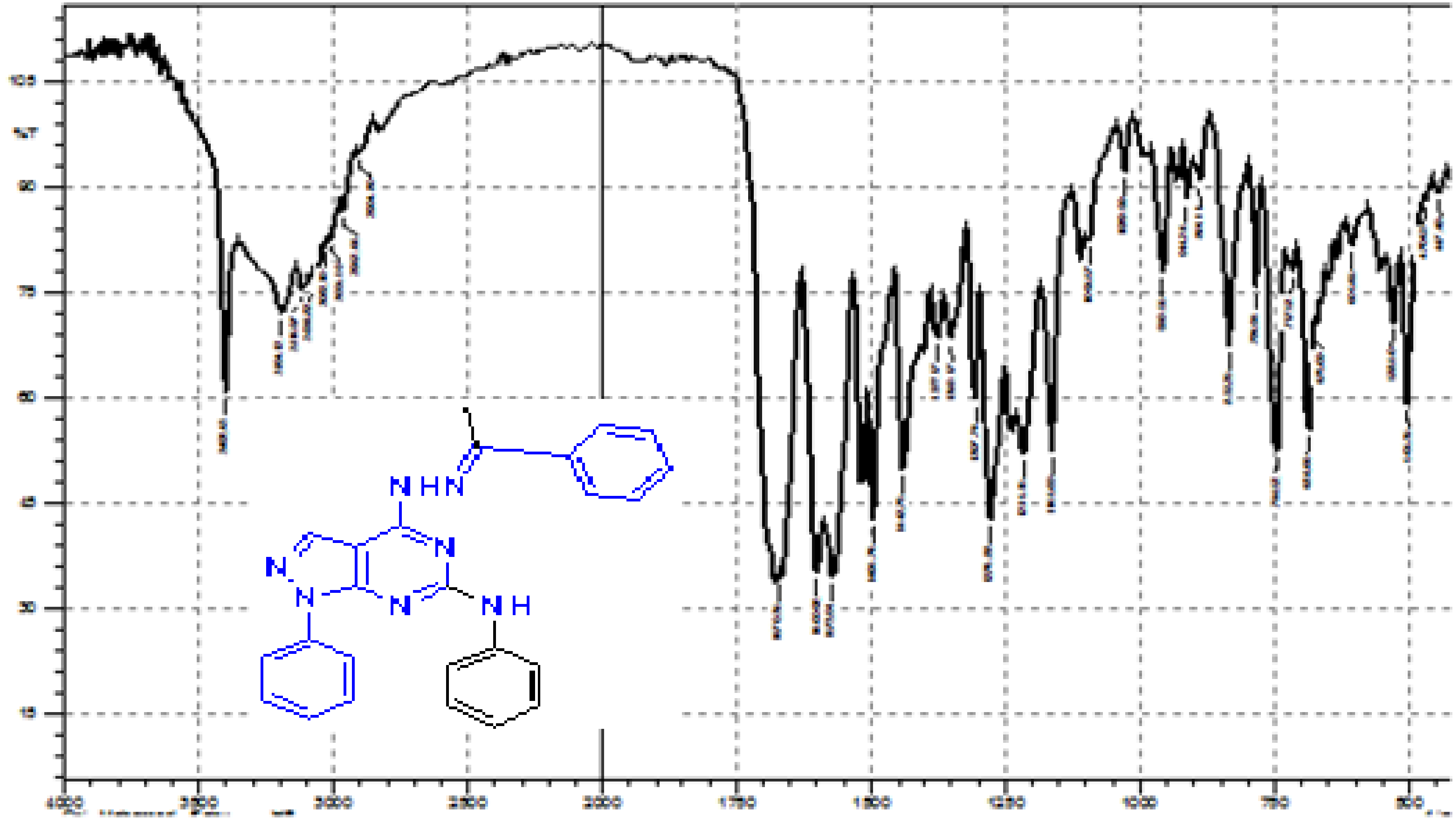
%T
NHN
N
N
N
N
NH
4000
3000
2500
2000
1750
1500
1250
1000
750
500

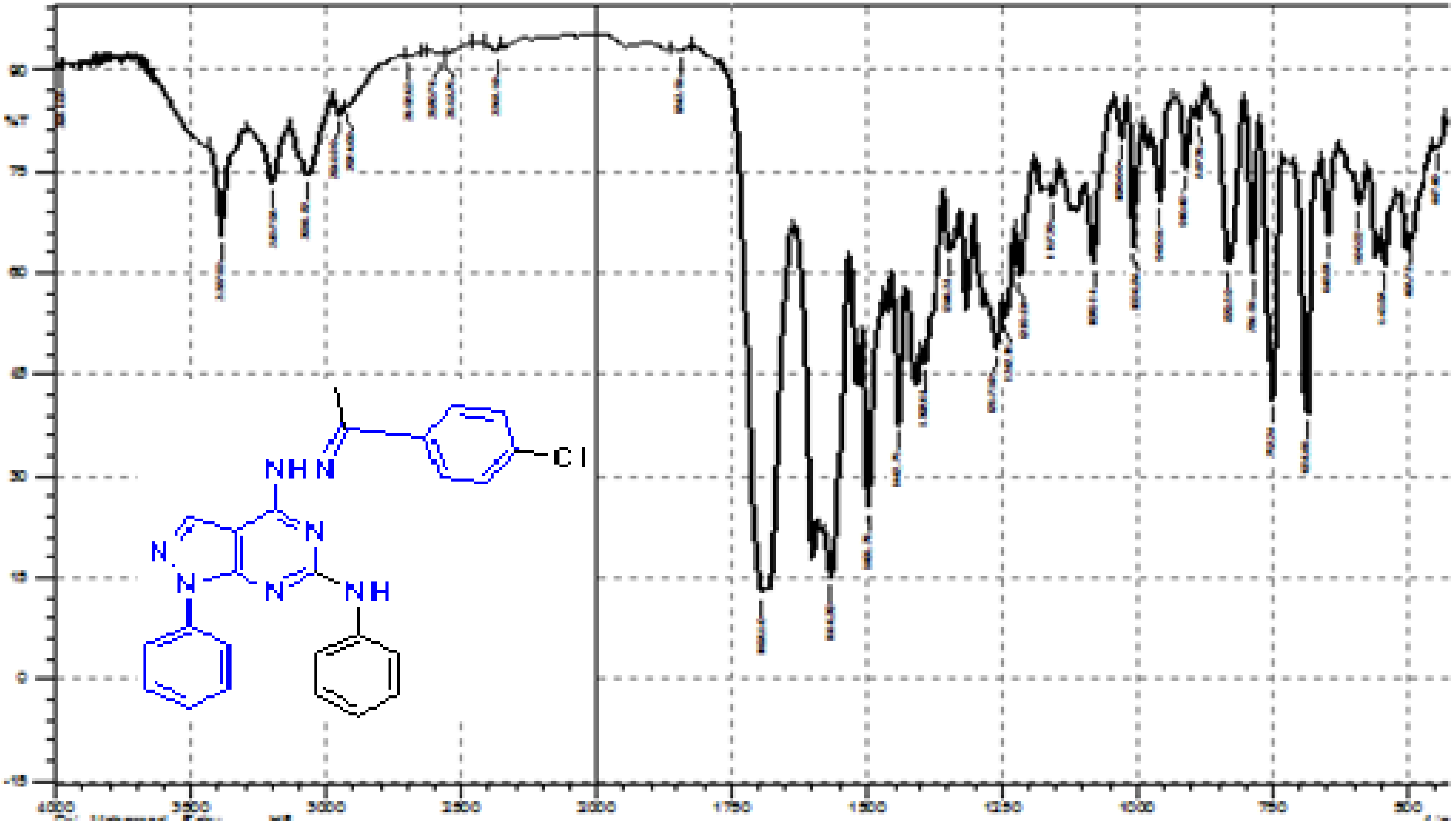

90
75
60
45
30
15
0
-15
4000
3500
3000
2500
2000
1750
1500
1250
1000
750
500
NH N
Cl
N
N
N
N
NH

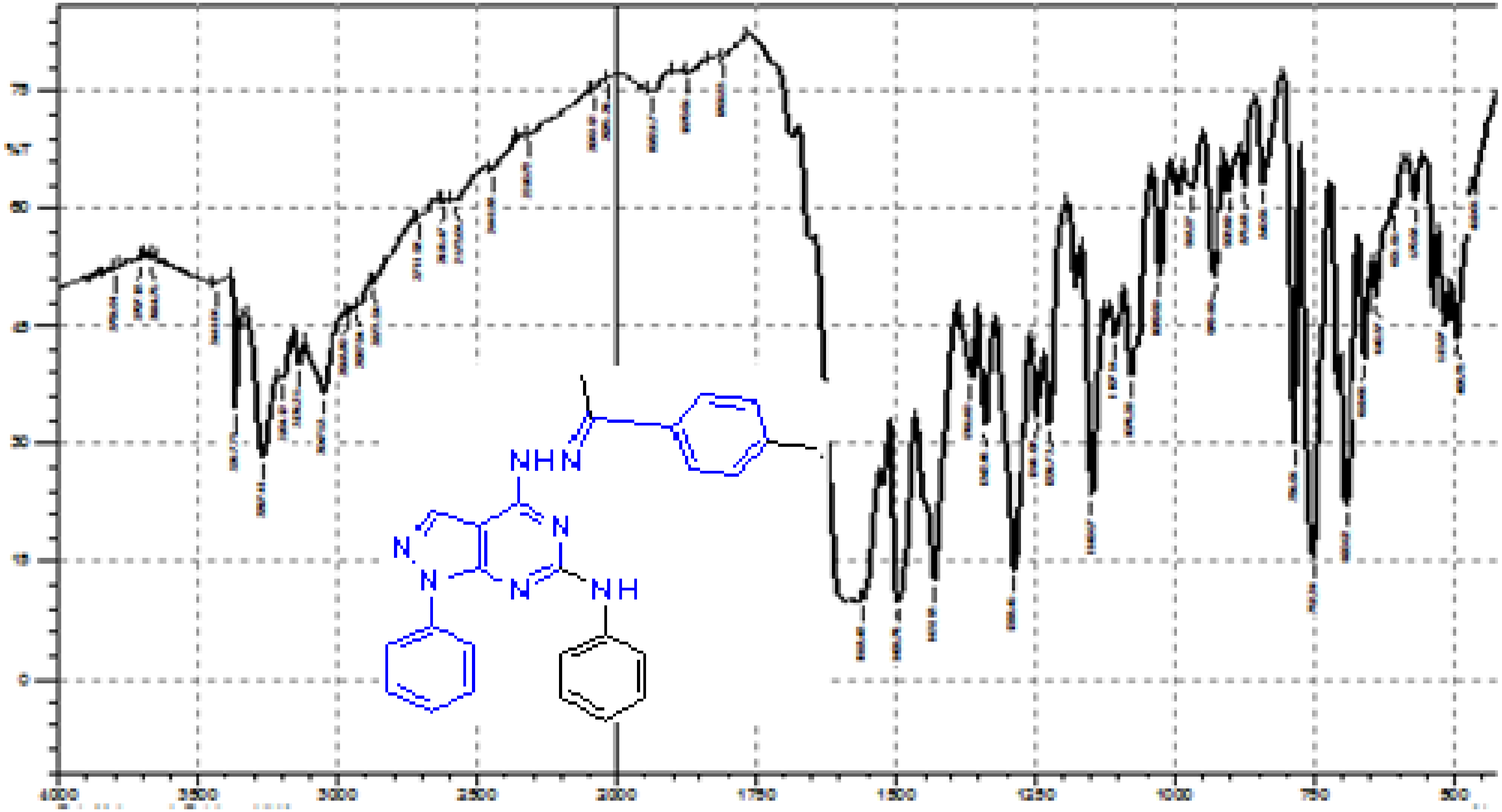
%T
75
60
45
30
15
0
4000
3500
3000
2500
2000
1750
1500
1250
1000
750
500
NH
N
N
N
N
N
NH

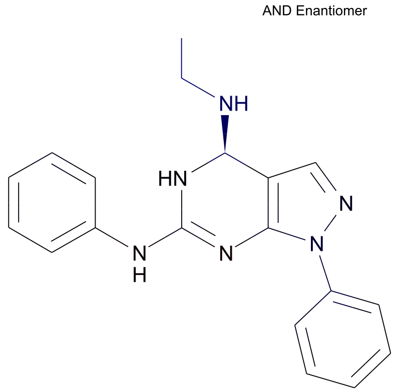

$C_{19}H_{20}N_6$
Molecular Weight: 332.4023
ALogP: 3.039
Rotatable Bonds: 5
Acceptors: 5
Donors: 3

Model Prediction

Prediction: Non-Mutagen
Probability: 0.657
Enrichment: 1.18
Bayesian Score: -3.32
Mahalanobis Distance: 8.25
Mahalanobis Distance p-value: 0.979

Prediction: Positive if the Bayesian score is above the estimated best cutoff value from minimizing the false positive and false negative rate.
Probability: The esimated probability that the sample is in the positive category. This assumes that the Bayesian score follows a normal distribution and is different from the prediction using a cutoff.
Enrichment: An estimate of enrichment, that is, the increased likelihood (versus random) of this sample being in the category.
Bayesian Score: The standard Laplacian-modified Bayesian score.
Mahalanobis Distance: The Mahalanobis distance (MD) is the distance to the center of the training data. The larger the MD, the less trustworthy the prediction.
Mahalanobis Distance p-value: The p-value gives the fraction of training data with an MD greater than or equal to the one for the given sample, assuming normally distributed data. The smaller the p-value, the less trustworthy the prediciton. For highly non-normal X properties (e.g., fingerprints), the MD p-value is wildly inaccurate.

Structural Similar Compounds

Name	18559-59-6	6724-53-4	101901-08-0
Structure	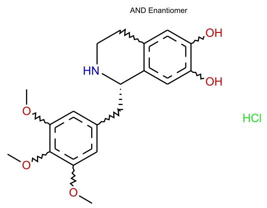	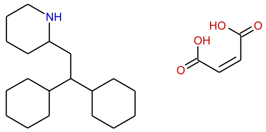	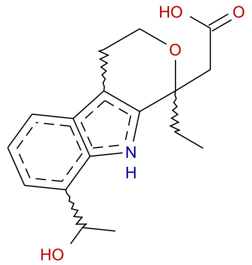
Actual Endpoint	Non-Mutagen	Non-Mutagen	Non-Mutagen
Predicted Endpoint	Non-Mutagen	Non-Mutagen	Non-Mutagen
Distance	0.570	0.577	0.598
Reference	Kazius et. al., J. Med. Chem. (2005) 48, 312-320	Kazius et. al., J. Med. Chem. (2005) 48, 312-320	Kazius et. al., J. Med. Chem. (2005) 48, 312-320

Model Applicability

Unknown features are fingerprint features in the query molecule, but not found or appearing too infrequently in the training set.

1. All properties and OPS components are within expected ranges.

Feature Contribution

Top features for positive contribution

Fingerprint	Bit/Smiles	Feature Structure	Score	Mutagen in training set
SCFP_12	-967249036	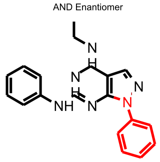 [*]:n(:[*])[c]1:[cH]:[cH]:[cH]:[cH]:[cH]:1	0.337	2 out of 2

SCFP_12	10	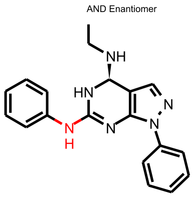 [*]N[*]	0.306	1774 out of 2287
SCFP_12	-1380909229	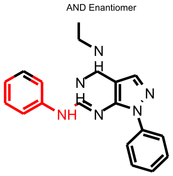 [*]N[c]1:[cH]:[*]:[cH]:[cH]:[cH]:1	0.304	957 out of 1235

Top Features for negative contribution

Fingerprint	Bit/Smiles	Feature Structure	Score	Mutagen in training set
SCFP_12	1725363918	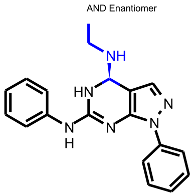 [*]C([*])NCC	-1.19	0 out of 4
SCFP_12	-56048396	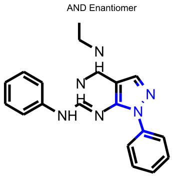 [*][c]1:[*]:[*]:n:n:1[c](:[*]):[*]	-0.762	0 out of 2
SCFP_12	136597326	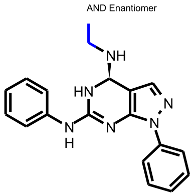 [*]CC	-0.439	584 out of 1586

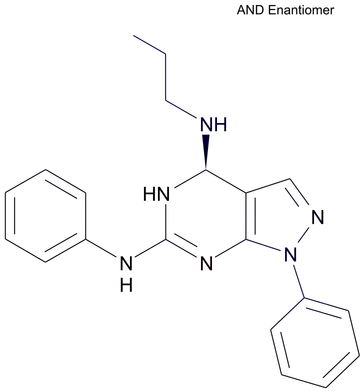

$C_{20}H_{22}N_6$
Molecular Weight: 346.42888
ALogP: 3.562
Rotatable Bonds: 6
Acceptors: 5
Donors: 3

Model Prediction

Prediction: Non-Mutagen
Probability: 0.652
Enrichment: 1.17
Bayesian Score: -3.49
Mahalanobis Distance: 8.86
Mahalanobis Distance p-value: 0.875

Prediction: Positive if the Bayesian score is above the estimated best cutoff value from minimizing the false positive and false negative rate.
Probability: The esimated probability that the sample is in the positive category. This assumes that the Bayesian score follows a normal distribution and is different from the prediction using a cutoff.
Enrichment: An estimate of enrichment, that is, the increased likelihood (versus random) of this sample being in the category.
Bayesian Score: The standard Laplacian-modified Bayesian score.
Mahalanobis Distance: The Mahalanobis distance (MD) is the distance to the center of the training data. The larger the MD, the less trustworthy the prediction.
Mahalanobis Distance p-value: The p-value gives the fraction of training data with an MD greater than or equal to the one for the given sample, assuming normally distributed data. The smaller the p-value, the less trustworthy the prediciton. For highly non-normal X properties (e.g., fingerprints), the MD p-value is wildly inaccurate.

Structural Similar Compounds

Name	6724-53-4	18559-59-6	38914-96-4
Structure	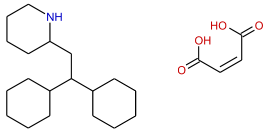	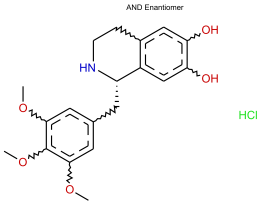	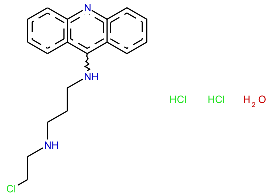
Actual Endpoint	Non-Mutagen	Non-Mutagen	Mutagen
Predicted Endpoint	Non-Mutagen	Non-Mutagen	Mutagen
Distance	0.553	0.577	0.586
Reference	Kazius et. al., J. Med. Chem. (2005) 48, 312-320	Kazius et. al., J. Med. Chem. (2005) 48, 312-320	Kazius et. al., J. Med. Chem. (2005) 48, 312-320

Model Applicability

Unknown features are fingerprint features in the query molecule, but not found or appearing too infrequently in the training set.

1. All properties and OPS components are within expected ranges.

Feature Contribution

Top features for positive contribution				
**Fingerprint**	**Bit/Smiles**	**Feature Structure**	**Score**	**Mutagen in training set**
SCFP_12	-967249036	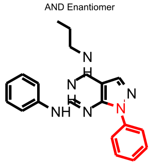 [*]:n(:[*])[c]1:[cH]:[cH]:[cH]:[cH]:[cH]:1	0.337	2 out of 2

SCFP_12	10	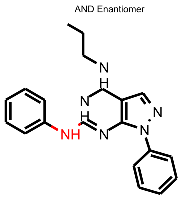 [*]N[*]	0.306	1774 out of 2287
SCFP_12	-1380909229	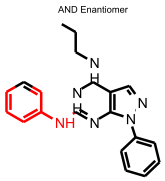 [*]N[c]1:[cH]:[*]:[cH]:[cH]:[cH]:1	0.304	957 out of 1235
**Top Features for negative contribution**				
**Fingerprint**	**Bit/Smiles**	**Feature Structure**	**Score**	**Mutagen in training set**
SCFP_12	-56048396	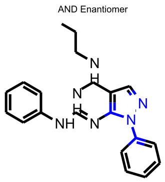 [*][c]1:[*]:[*]:n:n:1 [c](:[*]):[*]	-0.762	0 out of 2
SCFP_12	-1272798659	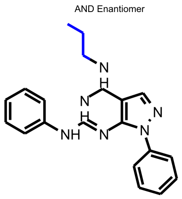 [*]CCC	-0.466	439 out of 1225
SCFP_12	136597326	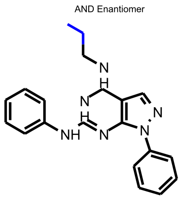 [*]CC	-0.439	584 out of 1586

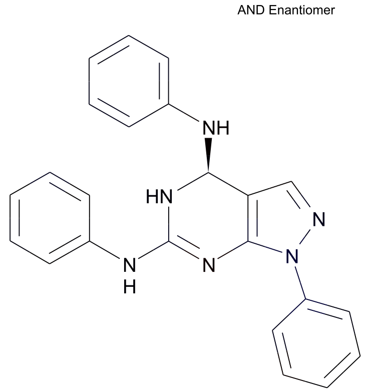

$C_{23}H_{20}N_6$

Molecular Weight: 380.4451

ALogP: 4.544

Rotatable Bonds: 5

Acceptors: 5

Donors: 3

Model Prediction

Prediction: Non-Mutagen

Probability: 0.703

Enrichment: 1.26

Bayesian Score: -1.64

Mahalanobis Distance: 7.86

Mahalanobis Distance p-value: 0.996

Prediction: Positive if the Bayesian score is above the estimated best cutoff value from minimizing the false positive and false negative rate.
Probability: The esimated probability that the sample is in the positive category. This assumes that the Bayesian score follows a normal distribution and is different from the prediction using a cutoff.
Enrichment: An estimate of enrichment, that is, the increased likelihood (versus random) of this sample being in the category.
Bayesian Score: The standard Laplacian-modified Bayesian score.
Mahalanobis Distance: The Mahalanobis distance (MD) is the distance to the center of the training data. The larger the MD, the less trustworthy the prediction.
Mahalanobis Distance p-value: The p-value gives the fraction of training data with an MD greater than or equal to the one for the given sample, assuming normally distributed data. The smaller the p-value, the less trustworthy the prediciton. For highly non-normal X properties (e.g., fingerprints), the MD p-value is wildly inaccurate.

Structural Similar Compounds

Name	83-44-3	URSODIOL	128-13-2
Structure	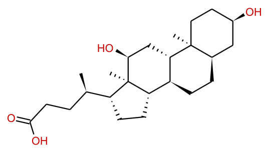	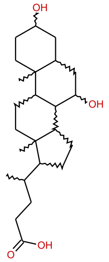	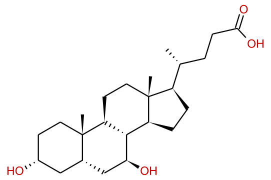
Actual Endpoint	Non-Mutagen	Non-Mutagen	Non-Mutagen
Predicted Endpoint	Non-Mutagen	Non-Mutagen	Non-Mutagen
Distance	0.589	0.592	0.592
Reference	Kazius et. al., J. Med. Chem. (2005) 48, 312-320	PDR 1994	Kazius et. al., J. Med. Chem. (2005) 48, 312-320

Model Applicability

Unknown features are fingerprint features in the query molecule, but not found or appearing too infrequently in the training set.

1. All properties and OPS components are within expected ranges.

Feature Contribution

Top features for positive contribution				
**Fingerprint**	**Bit/Smiles**	**Feature Structure**	**Score**	**Mutagen in training set**
SCFP_12	-967249036	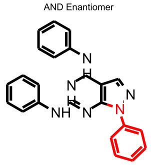 [*]:n(:[*])[c]1:[cH]:[cH]:[cH]:[cH]:[cH]:1	0.337	2 out of 2

SCFP_12	10	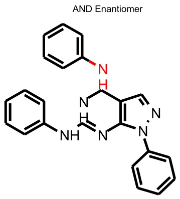 [*]N[*]	0.306	1774 out of 2287
SCFP_12	-1380909229	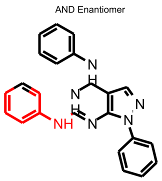 [*]N[c]1:[cH]:[*]:[cH]:[cH]:[cH]:1	0.304	957 out of 1235
**Top Features for negative contribution**				
**Fingerprint**	**Bit/Smiles**	**Feature Structure**	**Score**	**Mutagen in training set**
SCFP_12	-56048396	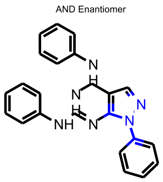 [*][c]1:[*]:[*]:n:n:1 [c](:[*]):[*]	-0.762	0 out of 2
SCFP_12	-1378896970	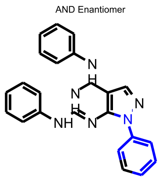 [*]:n(:[*])[c]1:[cH]: [*]:[cH]:[cH]:[cH]:1	-0.269	5 out of 12
SCFP_12	1076504043	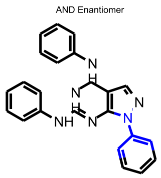 [*]:[cH]:[c](:[cH]:[*])n(:[*]):[*]	-0.269	5 out of 12

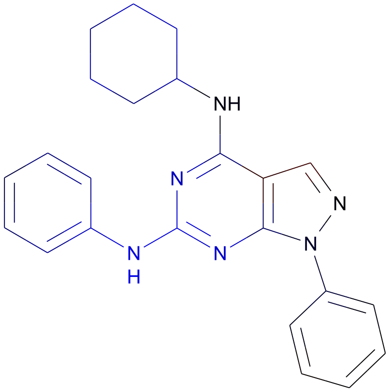

$C_{23}H_{24}N_6$
Molecular Weight: 384.47686
ALogP: 5.953
Rotatable Bonds: 5
Acceptors: 5
Donors: 2

Structural Similar Compounds

**Name**	**Methylene-(N;N'-bis)9-aminoacridine**	**86-42-0**	**3-(4'-Phenoxybenzylidenamino)-5H-1;2;3-triazin-[5;4b]indol-4-one**
Structure	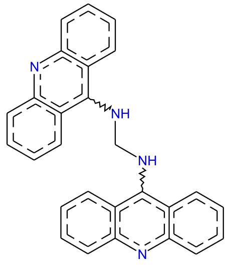	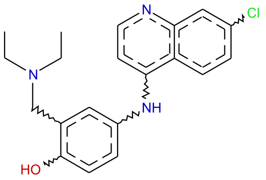	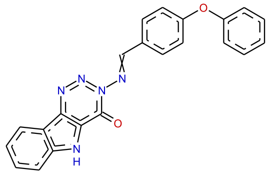
Actual Endpoint	Mutagen	Non-Mutagen	Non-Mutagen
Predicted Endpoint	Mutagen	Non-Mutagen	Mutagen
Distance	0.564	0.616	0.620
Reference	Mut. Res. 232:337-343;1990	Kazius et. al., J. Med. Chem. (2005) 48, 312-320	Mutagenesis 7(1):37-39; 1992

Model Prediction

Prediction: Non-Mutagen
Probability: 0.495
Enrichment: 0.887
Bayesian Score: -7.62
Mahalanobis Distance: 11
Mahalanobis Distance p-value: 0.0302

Prediction: Positive if the Bayesian score is above the estimated best cutoff value from minimizing the false positive and false negative rate.
Probability: The esimated probability that the sample is in the positive category. This assumes that the Bayesian score follows a normal distribution and is different from the prediction using a cutoff.
Enrichment: An estimate of enrichment, that is, the increased likelihood (versus random) of this sample being in the category.
Bayesian Score: The standard Laplacian-modified Bayesian score.
Mahalanobis Distance: The Mahalanobis distance (MD) is the distance to the center of the training data. The larger the MD, the less trustworthy the prediction.
Mahalanobis Distance p-value: The p-value gives the fraction of training data with an MD greater than or equal to the one for the given sample, assuming normally distributed data. The smaller the p-value, the less trustworthy the prediciton. For highly non-normal X properties (e.g., fingerprints), the MD p-value is wildly inaccurate.

Model Applicability

Unknown features are fingerprint features in the query molecule, but not found or appearing too infrequently in the training set.

1. All properties and OPS components are within expected ranges.

Feature Contribution

Top features for positive contribution

Fingerprint	Bit/Smiles	Feature Structure	Score	Mutagen in training set
SCFP_12	-1231050790	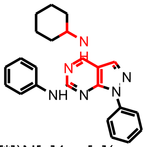 [*]C([*])N[c]1:n:[c]([*]):[*]:[c]2:[*]:[*]:[cH]:[c]:1:2	0.388	3 out of 3

SCFP_12	112346096	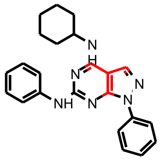<br>[*][c](:[*]):[c]1:[cH<br>]:[*]:[*]:[c]:1:[*]	0.36	1035 out of 1263
SCFP_12	916880471	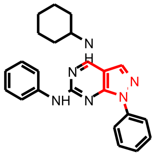<br>[*]n1:n:[cH]:[c](:[c]<br>([*]):[*]):[c]:1:[*]	0.337	14 out of 17
**Top Features for negative contribution**				
**Fingerprint**	**Bit/Smiles**	**Feature Structure**	**Score**	**Mutagen in training set**
SCFP_12	-1525101452	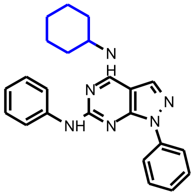<br>[*]C1CCCCC1	-1.17	27 out of 156
SCFP_12	1175638033	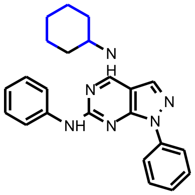<br>[*]C1[*]CCCC1	-1.05	67 out of 337
SCFP_12	-930313022	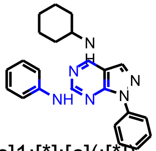<br>[*][c]1:[*]:[c](:[*])<br>:n:[c](N[c](:[*]):[*<br>]):n:1	-0.998	0 out of 3

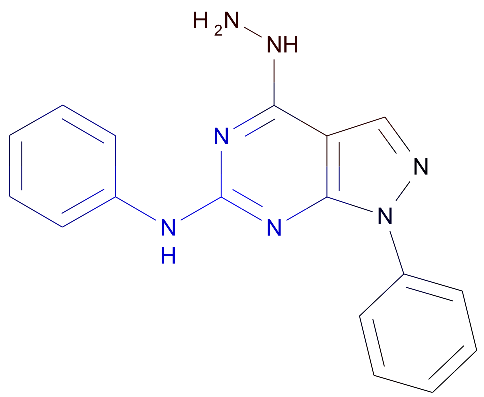

$C_{17}H_{15}N_7$
Molecular Weight: 317.3479
ALogP: 3.562
Rotatable Bonds: 4
Acceptors: 6
Donors: 3

Model Prediction

Prediction: Non-Mutagen
Probability: 0.669
Enrichment: 1.2
Bayesian Score: -2.91
Mahalanobis Distance: 7.22
Mahalanobis Distance p-value: 1

Prediction: Positive if the Bayesian score is above the estimated best cutoff value from minimizing the false positive and false negative rate.
Probability: The esimated probability that the sample is in the positive category. This assumes that the Bayesian score follows a normal distribution and is different from the prediction using a cutoff.
Enrichment: An estimate of enrichment, that is, the increased likelihood (versus random) of this sample being in the category.
Bayesian Score: The standard Laplacian-modified Bayesian score.
Mahalanobis Distance: The Mahalanobis distance (MD) is the distance to the center of the training data. The larger the MD, the less trustworthy the prediction.
Mahalanobis Distance p-value: The p-value gives the fraction of training data with an MD greater than or equal to the one for the given sample, assuming normally distributed data. The smaller the p-value, the less trustworthy the prediciton. For highly non-normal X properties (e.g., fingerprints), the MD p-value is wildly inaccurate.

Structural Similar Compounds

Name	134785-76-5	135561-93-2	4825-86-9
Structure	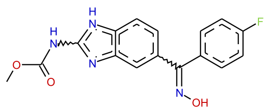	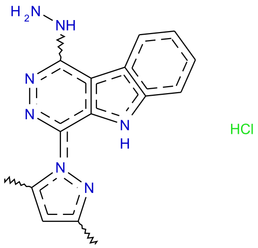	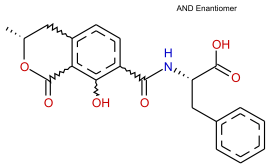
Actual Endpoint	Mutagen	Non-Mutagen	Non-Mutagen
Predicted Endpoint	Mutagen	Non-Mutagen	Non-Mutagen
Distance	0.557	0.575	0.610
Reference	Kazius et. al., J. Med. Chem. (2005) 48, 312-320	Kazius et. al., J. Med. Chem. (2005) 48, 312-320	Kazius et. al., J. Med. Chem. (2005) 48, 312-320

Model Applicability

Unknown features are fingerprint features in the query molecule, but not found or appearing too infrequently in the training set.

1. All properties and OPS components are within expected ranges.

Feature Contribution

Top features for positive contribution

Fingerprint	Bit/Smiles	Feature Structure	Score	Mutagen in training set
SCFP_12	-915345805	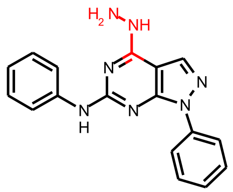 [*]:[c](:[*])NN	0.442	13 out of 14

SCFP_12	260535663	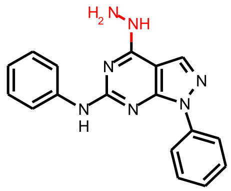 [*]NN	0.442	13 out of 14
SCFP_12	112346096	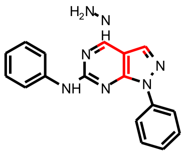 [*][c](:[*]):[c]1:[cH]:[*]:[*]:[c]:1:[*]	0.36	1035 out of 1263

Top Features for negative contribution

Fingerprint	Bit/Smiles	Feature Structure	Score	Mutagen in training set
SCFP_12	1328231062	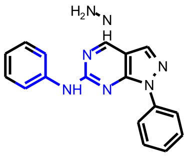 [*]:[cH]:[c](N[c](:n: [*]):n:[*]):[cH]:[*]	-0.998	0 out of 3
SCFP_12	-930313022	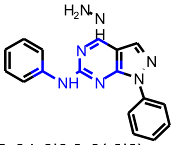 [*][c]1:[*]:[c](:[*]) :n:[c](N[c](:[*]):[*]):n:1	-0.998	0 out of 3
SCFP_12	-56048396	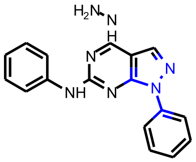 [*][c]1:[*]:[*]:n:n:1 [c](:[*]):[*]	-0.762	0 out of 2

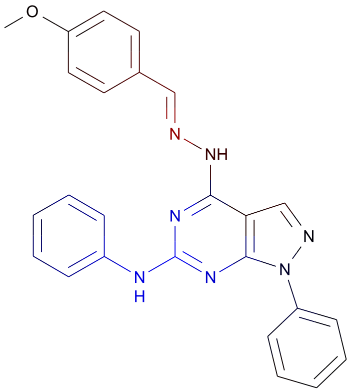

$C_{25}H_{21}N_7O$
Molecular Weight: 435.48054
ALogP: 5.889
Rotatable Bonds: 7
Acceptors: 7
Donors: 2

Model Prediction

Prediction: Non-Mutagen
Probability: 0.674
Enrichment: 1.21
Bayesian Score: -2.75
Mahalanobis Distance: 6.9
Mahalanobis Distance p-value: 1

Prediction: Positive if the Bayesian score is above the estimated best cutoff value from minimizing the false positive and false negative rate.
Probability: The esimated probability that the sample is in the positive category. This assumes that the Bayesian score follows a normal distribution and is different from the prediction using a cutoff.
Enrichment: An estimate of enrichment, that is, the increased likelihood (versus random) of this sample being in the category.
Bayesian Score: The standard Laplacian-modified Bayesian score.
Mahalanobis Distance: The Mahalanobis distance (MD) is the distance to the center of the training data. The larger the MD, the less trustworthy the prediction.
Mahalanobis Distance p-value: The p-value gives the fraction of training data with an MD greater than or equal to the one for the given sample, assuming normally distributed data. The smaller the p-value, the less trustworthy the prediciton. For highly non-normal X properties (e.g., fingerprints), the MD p-value is wildly inaccurate.

Structural Similar Compounds

Name	110004-69-8	633-03-4	1330-78-5
Structure	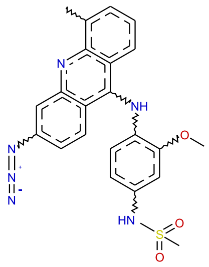	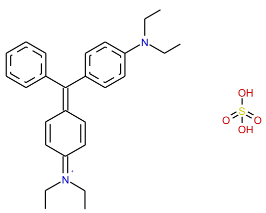	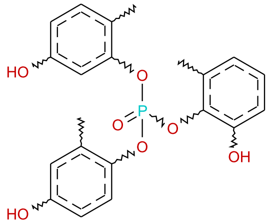
Actual Endpoint	Mutagen	Mutagen	Non-Mutagen
Predicted Endpoint	Mutagen	Mutagen	Non-Mutagen
Distance	0.618	0.618	0.633
Reference	Kazius et. al., J. Med. Chem. (2005) 48, 312-320	Kazius et. al., J. Med. Chem. (2005) 48, 312-320	Kazius et. al., J. Med. Chem. (2005) 48, 312-320

Model Applicability

Unknown features are fingerprint features in the query molecule, but not found or appearing too infrequently in the training set.

1. All properties and OPS components are within expected ranges.

Feature Contribution

Top features for positive contribution

Fingerprint	Bit/Smiles	Feature Structure	Score	Mutagen in training set
SCFP_12	427899304	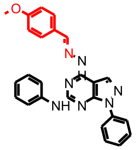 [*]\N=C\[c]1:[cH]:[cH]:[c](OC):[cH]:[cH]:1	0.48	8 out of 8

SCFP_12	2037424534	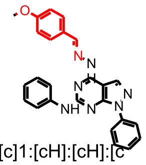<br>[*]O[c]1:[cH]:[cH]:[c<br>](\C=N\[*]):[cH]:[cH<br>]:1	0.442	13 out of 14
SCFP_12	1390366442	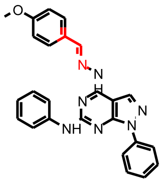<br>[*]\N=C\[c](:[*]):[*]	0.426	60 out of 68

Top Features for negative contribution

Fingerprint	Bit/Smiles	Feature Structure	Score	Mutagen in training set
SCFP_12	-930313022	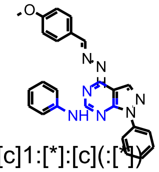<br>[*][c]1:[*]:[c](:[*])<br>:n:[c](N[c](:[*]):[*<br>]):n:1	-0.998	0 out of 3
SCFP_12	1328231062	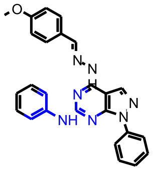<br>[*]:[cH]:[c](N[c](:n:<br>[*]):n:[*]):[cH]:[*]	-0.998	0 out of 3
SCFP_12	-822848031	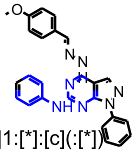<br>[*][c]1:[*]:[c](:[*])<br>:n:[c](N[c]2:[cH]:[c<br>H]:[*]:[cH]:[cH]:2):<br>n:1	-0.762	0 out of 2

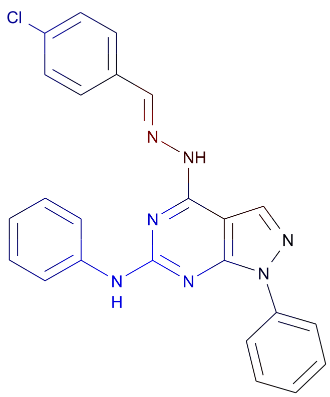

$C_{24}H_{18}ClN_7$

Molecular Weight: 439.89962

ALogP: 6.57

Rotatable Bonds: 6

Acceptors: 6

Donors: 2

Structural Similar Compounds

Name	633-03-4	Pigment red 2	989-38-8
Structure	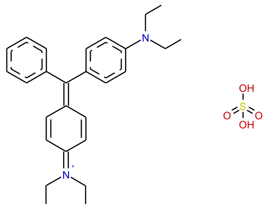	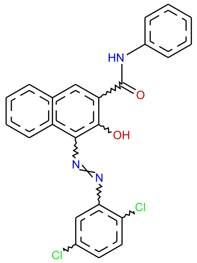	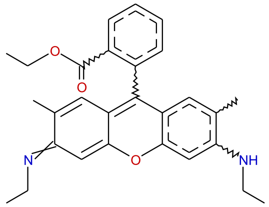
Actual Endpoint	Mutagen	Mutagen	Non-Mutagen
Predicted Endpoint	Mutagen	Mutagen	Non-Mutagen
Distance	0.609	0.635	0.661
Reference	Kazius et. al., J. Med. Chem. (2005) 48, 312-320	Environ. Mol. Mut. 19(21):1992	Helma, C., Cramer, T., Kramer, S., and De Raedt, L., J. Chem. Inf. Comput. Sci., 2004, pp. 1402-1411

Model Prediction

Prediction: Non-Mutagen

Probability: 0.571

Enrichment: 1.02

Bayesian Score: -5.81

Mahalanobis Distance: 6.97

Mahalanobis Distance p-value: 1

Prediction: Positive if the Bayesian score is above the estimated best cutoff value from minimizing the false positive and false negative rate.
Probability: The esimated probability that the sample is in the positive category. This assumes that the Bayesian score follows a normal distribution and is different from the prediction using a cutoff.
Enrichment: An estimate of enrichment, that is, the increased likelihood (versus random) of this sample being in the category.
Bayesian Score: The standard Laplacian-modified Bayesian score.
Mahalanobis Distance: The Mahalanobis distance (MD) is the distance to the center of the training data. The larger the MD, the less trustworthy the prediction.
Mahalanobis Distance p-value: The p-value gives the fraction of training data with an MD greater than or equal to the one for the given sample, assuming normally distributed data. The smaller the p-value, the less trustworthy the prediciton. For highly non-normal X properties (e.g., fingerprints), the MD p-value is wildly inaccurate.

Model Applicability

Unknown features are fingerprint features in the query molecule, but not found or appearing too infrequently in the training set.

1. All properties and OPS components are within expected ranges.

Feature Contribution

Top features for positive contribution

Fingerprint	Bit/Smiles	Feature Structure	Score	Mutagen in training set
SCFP_12	1390366442	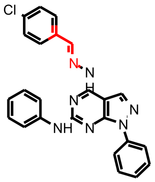 [*]\N=C\[c](:[*]):[*]	0.426	60 out of 68

SCFP_12	346293585	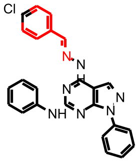 [*]\N=C\[c]1:[cH]:[cH]:[*]:[cH]:[cH]:1	0.392	25 out of 29
SCFP_12	-1325991669	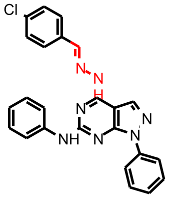 [*]N\N=C\[*]	0.362	7 out of 8

Top Features for negative contribution

Fingerprint	Bit/Smiles	Feature Structure	Score	Mutagen in training set
SCFP_12	-930313022	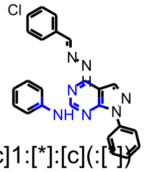 [*][c]1:[*]:[c](:[*]):n:[c](N[c](:[*]):[*]):n:1	-0.998	0 out of 3
SCFP_12	1328231062	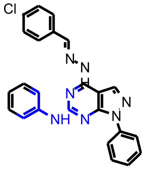 [*]:[cH]:[c](N[c](:n:[*]):n:[*]):[cH]:[*]	-0.998	0 out of 3
SCFP_12	-56048396	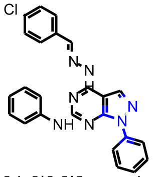 [*][c]1:[*]:[*]:n:n:1[c](:[*]):[*]	-0.762	0 out of 2

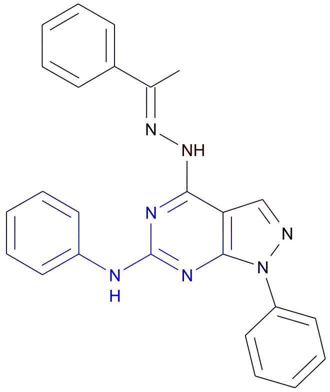

$C_{25}H_{21}N_7$
Molecular Weight: 419.48114
ALogP: 5.886
Rotatable Bonds: 6
Acceptors: 6
Donors: 2

Model Prediction

Prediction: Non-Mutagen
Probability: 0.592
Enrichment: 1.06
Bayesian Score: -5.26
Mahalanobis Distance: 7.38
Mahalanobis Distance p-value: 1

Prediction: Positive if the Bayesian score is above the estimated best cutoff value from minimizing the false positive and false negative rate.
Probability: The esimated probability that the sample is in the positive category. This assumes that the Bayesian score follows a normal distribution and is different from the prediction using a cutoff.
Enrichment: An estimate of enrichment, that is, the increased likelihood (versus random) of this sample being in the category.
Bayesian Score: The standard Laplacian-modified Bayesian score.
Mahalanobis Distance: The Mahalanobis distance (MD) is the distance to the center of the training data. The larger the MD, the less trustworthy the prediction.
Mahalanobis Distance p-value: The p-value gives the fraction of training data with an MD greater than or equal to the one for the given sample, assuming normally distributed data. The smaller the p-value, the less trustworthy the prediciton. For highly non-normal X properties (e.g., fingerprints), the MD p-value is wildly inaccurate.

Structural Similar Compounds

Name	633-03-4	989-38-8	989-38-8
Structure	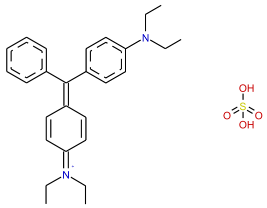	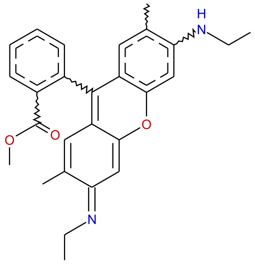	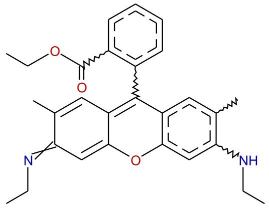
Actual Endpoint	Mutagen	Non-Mutagen	Non-Mutagen
Predicted Endpoint	Mutagen	Non-Mutagen	Non-Mutagen
Distance	0.588	0.629	0.634
Reference	Kazius et. al., J. Med. Chem. (2005) 48, 312-320	Kazius et. al., J. Med. Chem. (2005) 48, 312-320	Helma, C., Cramer, T., Kramer, S., and De Raedt, L., J. Chem. Inf. Comput. Sci., 2004, pp. 1402-1411

Model Applicability

Unknown features are fingerprint features in the query molecule, but not found or appearing too infrequently in the training set.

1. All properties and OPS components are within expected ranges.

Feature Contribution

Top features for positive contribution				
**Fingerprint**	**Bit/Smiles**	**Feature Structure**	**Score**	**Mutagen in training set**
SCFP_12	-1325991669	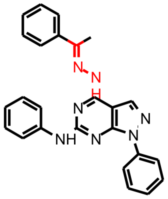 [*]N\N=C\[*]	0.362	7 out of 8

SCFP_12	112346096	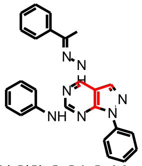 [*][c](:[*]):[c]1:[cH]:[*]:[*]:[c]:1:[*]	0.36	1035 out of 1263
SCFP_12	916880471	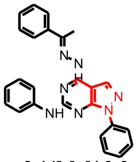 [*]n1:n:[cH]:[c](:[c] ([*]):[*]):[c]:1:[*]	0.337	14 out of 17
**Top Features for negative contribution**				
**Fingerprint**	**Bit/Smiles**	**Feature Structure**	**Score**	**Mutagen in training set**
SCFP_12	1328231062	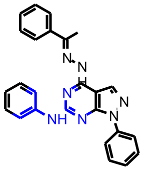 [*]:[cH]:[c](N[c](:n: [*]):n:[*]):[cH]:[*]	-0.998	0 out of 3
SCFP_12	-930313022	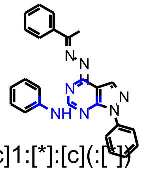 [*][c]1:[*]:[c](:[*]) :n:[c](N[c](:[*]):[*]):n:1	-0.998	0 out of 3
SCFP_12	-822848031	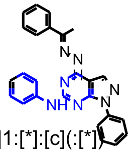 [*][c]1:[*]:[c](:[*]) :n:[c](N[c]2:[cH]:[c H]:[*]:[cH]:[cH]:2): n:1	-0.762	0 out of 2

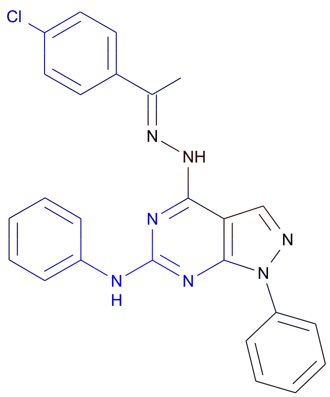

$C_{25}H_{20}ClN_7$
Molecular Weight: 453.9262
ALogP: 6.55
Rotatable Bonds: 6
Acceptors: 6
Donors: 2

Model Prediction

Prediction: Non-Mutagen
Probability: 0.468
Enrichment: 0.837
Bayesian Score: -8.24
Mahalanobis Distance: 8.1
Mahalanobis Distance p-value: 0.988

Prediction: Positive if the Bayesian score is above the estimated best cutoff value from minimizing the false positive and false negative rate.
Probability: The esimated probability that the sample is in the positive category. This assumes that the Bayesian score follows a normal distribution and is different from the prediction using a cutoff.
Enrichment: An estimate of enrichment, that is, the increased likelihood (versus random) of this sample being in the category.
Bayesian Score: The standard Laplacian-modified Bayesian score.
Mahalanobis Distance: The Mahalanobis distance (MD) is the distance to the center of the training data. The larger the MD, the less trustworthy the prediction.
Mahalanobis Distance p-value: The p-value gives the fraction of training data with an MD greater than or equal to the one for the given sample, assuming normally distributed data. The smaller the p-value, the less trustworthy the prediciton. For highly non-normal X properties (e.g., fingerprints), the MD p-value is wildly inaccurate.

Structural Similar Compounds

Name	633-03-4	Pigment red 2	54854-14-7
Structure	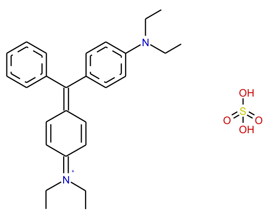	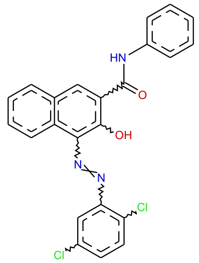	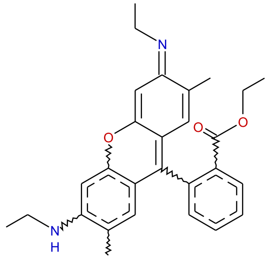
Actual Endpoint	Mutagen	Mutagen	Non-Mutagen
Predicted Endpoint	Mutagen	Mutagen	Non-Mutagen
Distance	0.597	0.637	0.652
Reference	Kazius et. al., J. Med. Chem. (2005) 48, 312-320	Environ. Mol. Mut. 19(21):1992	Kazius et. al., J. Med. Chem. (2005) 48, 312-320

Model Applicability

Unknown features are fingerprint features in the query molecule, but not found or appearing too infrequently in the training set.

1. All properties and OPS components are within expected ranges.

Feature Contribution

Top features for positive contribution

Fingerprint	Bit/Smiles	Feature Structure	Score	Mutagen in training set
SCFP_12	-1325991669	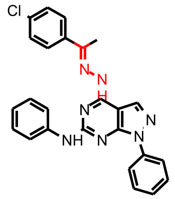 [*]N\N=C\[*]	0.362	7 out of 8

SCFP_12	112346096	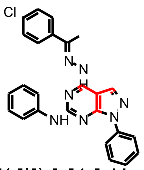 [*][c](:[*]):[c]1:[cH]:[*]:[*]:[c]:1:[*]	0.36	1035 out of 1263
SCFP_12	916880471	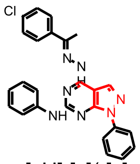 [*]n1:n:[cH]:[c](:[c]([*]):[*]):[c]:1:[*]	0.337	14 out of 17

Top Features for negative contribution

Fingerprint	Bit/Smiles	Feature Structure	Score	Mutagen in training set
SCFP_12	1328231062	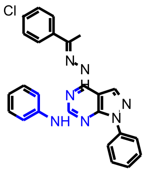 [*]:[cH]:[c](N[c](:n:[*]):n:[*]):[cH]:[*]	-0.998	0 out of 3
SCFP_12	-930313022	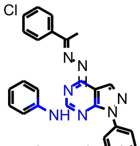 [*][c]1:[*]:[c](:[*]):n:[c](N[c](:[*]):[*]):n:1	-0.998	0 out of 3
SCFP_12	-331724199	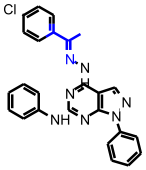 [*]\N=C(/C)\[c](:[*]):[*]	-0.762	0 out of 2

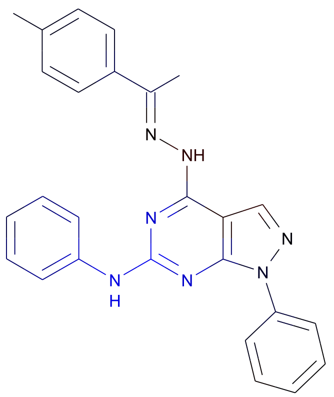

$C_{26}H_{23}N_7$
Molecular Weight: 433.50772
ALogP: 6.372
Rotatable Bonds: 6
Acceptors: 6
Donors: 2

Structural Similar Compounds

Name	633-03-4	989-38-8	54854-14-7
Structure	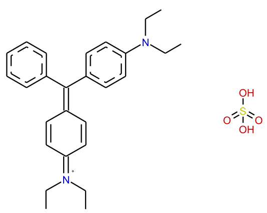	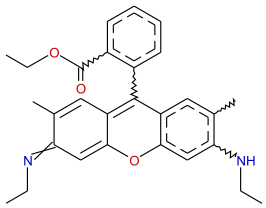	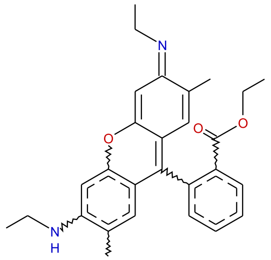
Actual Endpoint	Mutagen	Non-Mutagen	Non-Mutagen
Predicted Endpoint	Mutagen	Non-Mutagen	Non-Mutagen
Distance	0.598	0.639	0.639
Reference	Kazius et. al., J. Med. Chem. (2005) 48, 312-320	Helma, C., Cramer, T., Kramer, S., and De Raedt, L., J. Chem. Inf. Comput. Sci., 2004, pp. 1402-1411	Kazius et. al., J. Med. Chem. (2005) 48, 312-320

Model Prediction

Prediction: Non-Mutagen
Probability: 0.585
Enrichment: 1.05
Bayesian Score: -5.45
Mahalanobis Distance: 7.47
Mahalanobis Distance p-value: 0.999

Prediction: Positive if the Bayesian score is above the estimated best cutoff value from minimizing the false positive and false negative rate.
Probability: The esimated probability that the sample is in the positive category. This assumes that the Bayesian score follows a normal distribution and is different from the prediction using a cutoff.
Enrichment: An estimate of enrichment, that is, the increased likelihood (versus random) of this sample being in the category.
Bayesian Score: The standard Laplacian-modified Bayesian score.
Mahalanobis Distance: The Mahalanobis distance (MD) is the distance to the center of the training data. The larger the MD, the less trustworthy the prediction.
Mahalanobis Distance p-value: The p-value gives the fraction of training data with an MD greater than or equal to the one for the given sample, assuming normally distributed data. The smaller the p-value, the less trustworthy the prediciton. For highly non-normal X properties (e.g., fingerprints), the MD p-value is wildly inaccurate.

Model Applicability

Unknown features are fingerprint features in the query molecule, but not found or appearing too infrequently in the training set.

1. All properties and OPS components are within expected ranges.

Feature Contribution

Top features for positive contribution				
**Fingerprint**	**Bit/Smiles**	**Feature Structure**	**Score**	**Mutagen in training set**
SCFP_12	-1325991669	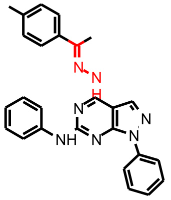 [*]N\N=C\[*]	0.362	7 out of 8

SCFP_12	112346096	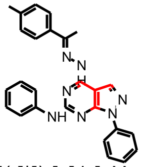 [*][c](:[*]):[c]1:[cH]:[*]:[*]:[c]:1:[*]	0.36	1035 out of 1263
SCFP_12	916880471	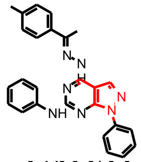 [*]n1:n:[cH]:[c](:[c]([*]):[*]):[c]:1:[*]	0.337	14 out of 17

Top Features for negative contribution

Fingerprint	Bit/Smiles	Feature Structure	Score	Mutagen in training set
SCFP_12	1328231062	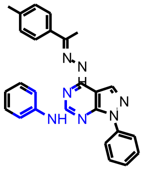 [*]:[cH]:[c](N[c](:n:[*]):n:[*]):[cH]:[*]	-0.998	0 out of 3
SCFP_12	-930313022	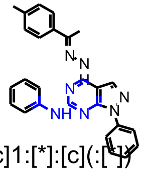 [*][c]1:[*]:[c](:[*]):n:[c](N[c](:[*]):[*]):n:1	-0.998	0 out of 3
SCFP_12	-822848031	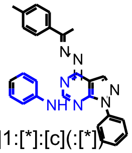 [*][c]1:[*]:[c](:[*]):n:[c](N[c]2:[cH]:[cH]:[*]:[cH]:[cH]:2):n:1	-0.762	0 out of 2

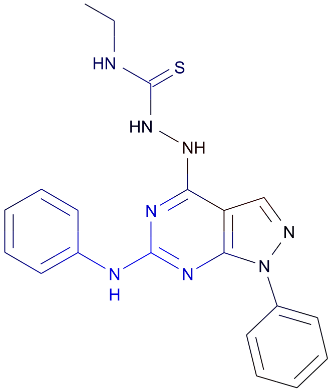

$C_{20}H_{20}N_8S$
Molecular Weight: 404.4914
ALogP: 4.99
Rotatable Bonds: 8
Acceptors: 6
Donors: 4

Model Prediction

Prediction: Non-Mutagen
Probability: 0.511
Enrichment: 0.916
Bayesian Score: -7.26
Mahalanobis Distance: 12.2
Mahalanobis Distance p-value: 0.000301

Prediction: Positive if the Bayesian score is above the estimated best cutoff value from minimizing the false positive and false negative rate.
Probability: The esimated probability that the sample is in the positive category. This assumes that the Bayesian score follows a normal distribution and is different from the prediction using a cutoff.
Enrichment: An estimate of enrichment, that is, the increased likelihood (versus random) of this sample being in the category.
Bayesian Score: The standard Laplacian-modified Bayesian score.
Mahalanobis Distance: The Mahalanobis distance (MD) is the distance to the center of the training data. The larger the MD, the less trustworthy the prediction.
Mahalanobis Distance p-value: The p-value gives the fraction of training data with an MD greater than or equal to the one for the given sample, assuming normally distributed data. The smaller the p-value, the less trustworthy the prediciton. For highly non-normal X properties (e.g., fingerprints), the MD p-value is wildly inaccurate.

Structural Similar Compounds

Name	522-40-7	65089-17-0	1330-78-5
Structure	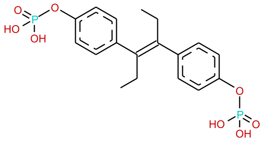	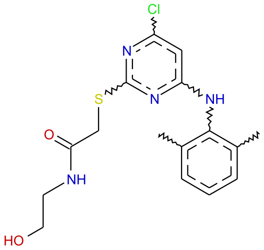	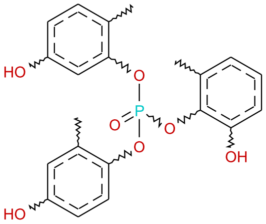
Actual Endpoint	Non-Mutagen	Non-Mutagen	Non-Mutagen
Predicted Endpoint	Non-Mutagen	Non-Mutagen	Non-Mutagen
Distance	0.638	0.654	0.665
Reference	Kazius et. al., J. Med. Chem. (2005) 48, 312-320	Kazius et. al., J. Med. Chem. (2005) 48, 312-320	Kazius et. al., J. Med. Chem. (2005) 48, 312-320

Model Applicability

Unknown features are fingerprint features in the query molecule, but not found or appearing too infrequently in the training set.

1. All properties and OPS components are within expected ranges.

Feature Contribution

Top features for positive contribution				
**Fingerprint**	**Bit/Smiles**	**Feature Structure**	**Score**	**Mutagen in training set**
SCFP_12	-1848869096	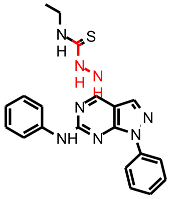 [*]NNC(=[*])[*]	0.38	18 out of 21

SCFP_12	112346096	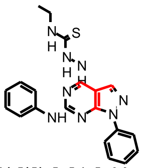 [*][c](:[*]):[c]1:[cH]:[*]:[*]:[c]:1:[*]	0.36	1035 out of 1263
SCFP_12	916880471	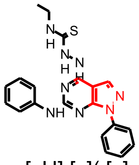 [*]n1:n:[cH]:[c](:[c] ([*]):[*]):[c]:1:[*]	0.337	14 out of 17

Top Features for negative contribution

Fingerprint	Bit/Smiles	Feature Structure	Score	Mutagen in training set
SCFP_12	1328231062	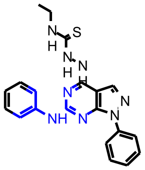 [*]:[cH]:[c](N[c](:n: [*]):n:[*]):[cH]:[*]	-0.998	0 out of 3
SCFP_12	-930313022	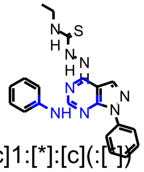 [*][c]1:[*]:[c](:[*]) :n:[c](N[c](:[*]):[*]):n:1	-0.998	0 out of 3
SCFP_12	382734644	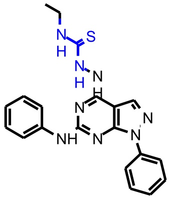 [*]NC(=S)N[*]	-0.811	3 out of 14

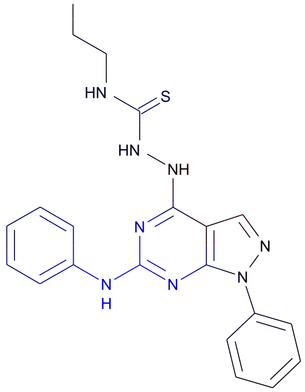

$C_{21}H_{22}N_8S$
Molecular Weight: 418.51798
ALogP: 5.514
Rotatable Bonds: 9
Acceptors: 6
Donors: 4

Model Prediction

Prediction: Non-Mutagen
Probability: 0.529
Enrichment: 0.948
Bayesian Score: -6.83
Mahalanobis Distance: 12.7
Mahalanobis Distance p-value: 2.55e-005

Prediction: Positive if the Bayesian score is above the estimated best cutoff value from minimizing the false positive and false negative rate.
Probability: The esimated probability that the sample is in the positive category. This assumes that the Bayesian score follows a normal distribution and is different from the prediction using a cutoff.
Enrichment: An estimate of enrichment, that is, the increased likelihood (versus random) of this sample being in the category.
Bayesian Score: The standard Laplacian-modified Bayesian score.
Mahalanobis Distance: The Mahalanobis distance (MD) is the distance to the center of the training data. The larger the MD, the less trustworthy the prediction.
Mahalanobis Distance p-value: The p-value gives the fraction of training data with an MD greater than or equal to the one for the given sample, assuming normally distributed data. The smaller the p-value, the less trustworthy the prediciton. For highly non-normal X properties (e.g., fingerprints), the MD p-value is wildly inaccurate.

Structural Similar Compounds

Name	522-40-7	36167-69-8	1330-78-5
Structure	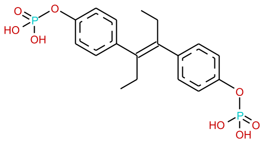	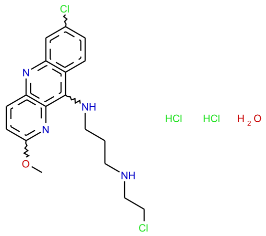	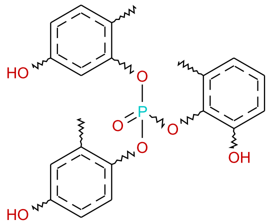
Actual Endpoint	Non-Mutagen	Mutagen	Non-Mutagen
Predicted Endpoint	Non-Mutagen	Mutagen	Non-Mutagen
Distance	0.667	0.684	0.688
Reference	Kazius et. al., J. Med. Chem. (2005) 48, 312-320	Kazius et. al., J. Med. Chem. (2005) 48, 312-320	Kazius et. al., J. Med. Chem. (2005) 48, 312-320

Model Applicability

Unknown features are fingerprint features in the query molecule, but not found or appearing too infrequently in the training set.

1. All properties and OPS components are within expected ranges.

Feature Contribution

Top features for positive contribution				
**Fingerprint**	**Bit/Smiles**	**Feature Structure**	**Score**	**Mutagen in training set**
SCFP_12	-1848869096	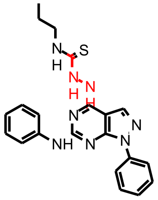 [*]NNC(=[*])[*]	0.38	18 out of 21

SCFP_12	112346096	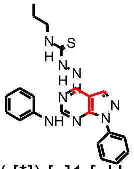 [*][c](:[*]):[c]1:[cH]:[*]:[*]:[c]:1:[*]	0.36	1035 out of 1263
SCFP_12	916880471	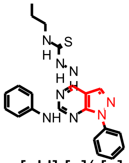 [*]n1:n:[cH]:[c](:[c]([*]):[*]):[c]:1:[*]	0.337	14 out of 17
**Top Features for negative contribution**				
**Fingerprint**	**Bit/Smiles**	**Feature Structure**	**Score**	**Mutagen in training set**
SCFP_12	-930313022	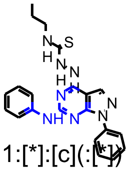 [*][c]1:[*]:[c](:[*]):n:[c](N[c](:[*]):[*]):n:1	-0.998	0 out of 3
SCFP_12	1328231062	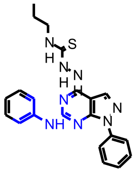 [*]:[cH]:[c](N[c](:n:[*]):n:[*]):[cH]:[*]	-0.998	0 out of 3
SCFP_12	382734644	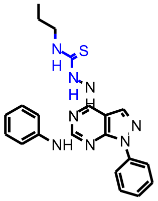 [*]NC(=S)N[*]	-0.811	3 out of 14

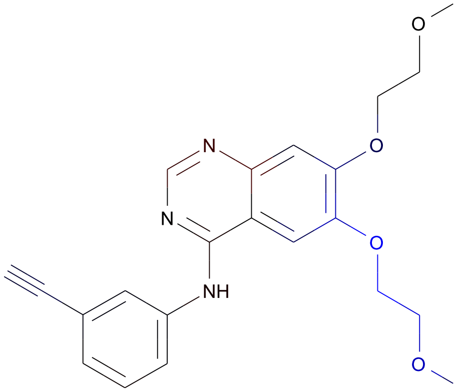

$C_{22}H_{23}N_3O_4$

Molecular Weight: 393.43572

ALogP: 4.309

Rotatable Bonds: 10

Acceptors: 7

Donors: 1

Structural Similar Compounds

Name	Carvedilol	99522-79-9	HYCANTHONE FUROATE
Structure	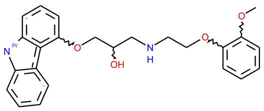	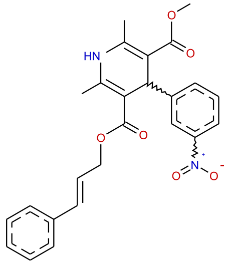	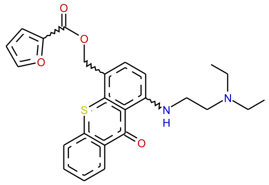
Actual Endpoint	Non-Mutagen	Non-Mutagen	Mutagen
Predicted Endpoint	Non-Mutagen	Non-Mutagen	Mutagen
Distance	0.594	0.598	0.606
Reference	Contrera, J.F., Matthews, E.J., Kruhlak, N.L., and Benz, R.D., Regulatory Toxicology and Pharmacology 2005, 313-323.	Kazius et. al., J. Med. Chem. (2005) 48, 312-320	EMIC

Model Prediction

Prediction: Non-Mutagen

Probability: 0.6

Enrichment: 1.07

Bayesian Score: -5.04

Mahalanobis Distance: 14.6

Mahalanobis Distance p-value: 6.74e-011

Prediction: Positive if the Bayesian score is above the estimated best cutoff value from minimizing the false positive and false negative rate.
Probability: The esimated probability that the sample is in the positive category. This assumes that the Bayesian score follows a normal distribution and is different from the prediction using a cutoff.
Enrichment: An estimate of enrichment, that is, the increased likelihood (versus random) of this sample being in the category.
Bayesian Score: The standard Laplacian-modified Bayesian score.
Mahalanobis Distance: The Mahalanobis distance (MD) is the distance to the center of the training data. The larger the MD, the less trustworthy the prediction.
Mahalanobis Distance p-value: The p-value gives the fraction of training data with an MD greater than or equal to the one for the given sample, assuming normally distributed data. The smaller the p-value, the less trustworthy the prediciton. For highly non-normal X properties (e.g., fingerprints), the MD p-value is wildly inaccurate.

Model Applicability

Unknown features are fingerprint features in the query molecule, but not found or appearing too infrequently in the training set.

1. All properties and OPS components are within expected ranges.

Feature Contribution

Top features for positive contribution

Fingerprint	Bit/Smiles	Feature Structure	Score	Mutagen in training set
SCFP_12	112346096	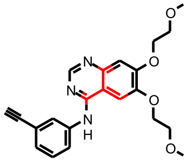 [*][c](:[*]):[c]1:[cH]:[*]:[*]:[c]:1:[*]	0.36	1035 out of 1263

SCFP_12	10	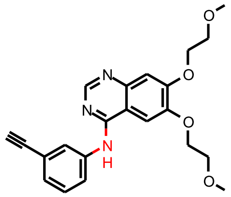 [*]N[*]	0.306	1774 out of 2287
SCFP_12	-1380909229	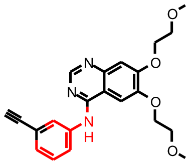 [*]N[c]1:[cH]:[*]:[cH]:[cH]:[cH]:1	0.304	957 out of 1235
**Top Features for negative contribution**				
**Fingerprint**	**Bit/Smiles**	**Feature Structure**	**Score**	**Mutagen in training set**
SCFP_12	-1099149596	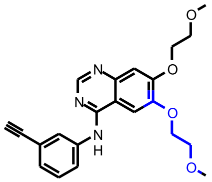 [*]OCCO[c](:[*]):[*]	-1.12	1 out of 9
SCFP_12	-677502852	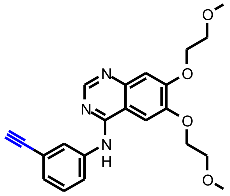 [*]C#C	-0.863	4 out of 19
SCFP_12	-417738003	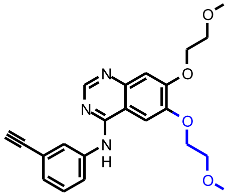 [*]OCCOC	-0.782	12 out of 48

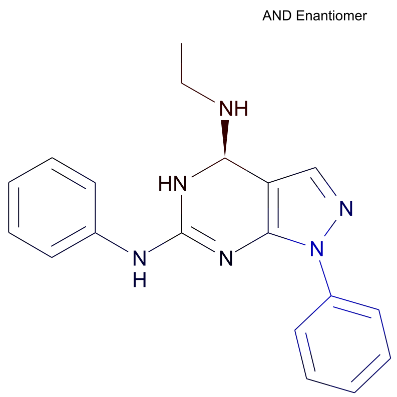

$C_{19}H_{20}N_6$
Molecular Weight: 332.4023
ALogP: 3.039
Rotatable Bonds: 5
Acceptors: 5
Donors: 3

Model Prediction

Prediction: Non-Toxic
Probability: 0.376
Enrichment: 0.714
Bayesian Score: -5.31
Mahalanobis Distance: 12.6
Mahalanobis Distance p-value: 2.3e-006

Prediction: Positive if the Bayesian score is above the estimated best cutoff value from minimizing the false positive and false negative rate.
Probability: The esimated probability that the sample is in the positive category. This assumes that the Bayesian score follows a normal distribution and is different from the prediction using a cutoff.
Enrichment: An estimate of enrichment, that is, the increased likelihood (versus random) of this sample being in the category.
Bayesian Score: The standard Laplacian-modified Bayesian score.
Mahalanobis Distance: The Mahalanobis distance (MD) is the distance to the center of the training data. The larger the MD, the less trustworthy the prediction.
Mahalanobis Distance p-value: The p-value gives the fraction of training data with an MD greater than or equal to the one for the given sample, assuming normally distributed data. The smaller the p-value, the less trustworthy the prediciton. For highly non-normal X properties (e.g., fingerprints), the MD p-value is wildly inaccurate.

Structural Similar Compounds

Name	Clebopride Malate	Clenbuterol	Ritodrine .HCl (Free base form)
Structure	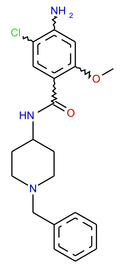	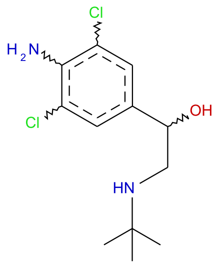	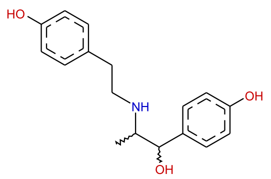
Actual Endpoint	Non-Toxic	Toxic	Toxic
Predicted Endpoint	Non-Toxic	Toxic	Toxic
Distance	0.611	0.611	0.632
Reference	Kiso to Rinsho 16:5649-5660; 1982	Iyakuhin Kenkyu 15(4):597-613; 1984	Kiso to Rinsho 19:2002-2018; 1985

Model Applicability

Unknown features are fingerprint features in the query molecule, but not found or appearing too infrequently in the training set.

1. All properties and OPS components are within expected ranges.

Feature Contribution

Top features for positive contribution				
**Fingerprint**	**Bit/Smiles**	**Feature Structure**	**Score**	**Toxic in training set**
SCFP_6	-627385064	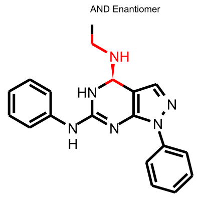 [*]CNC([*])[*]	0.414	14 out of 17

SCFP_6	2088704928	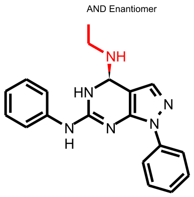 [*]NCC	0.303	38 out of 53
SCFP_6	1725363918	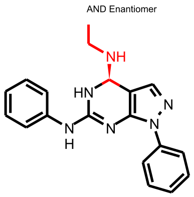 [*]C([*])NCC	0.271	1 out of 1
**Top Features for negative contribution**				
**Fingerprint**	**Bit/Smiles**	**Feature Structure**	**Score**	**Toxic in training set**
SCFP_6	-1378896970	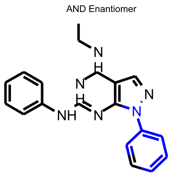 [*]:n(:[*])[c]1:[cH]:[*]:[cH]:[cH]:[cH]:1	-1.13	0 out of 4
SCFP_6	1076504043	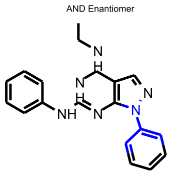 [*]:[cH]:[c](:[cH]:[*])n(:[*]):[*]	-1.13	0 out of 4
SCFP_6	-1380909229	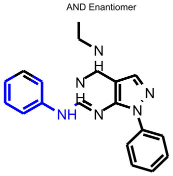 [*]N[c]1:[cH]:[*]:[cH]:[cH]:[cH]:1	-0.449	6 out of 19

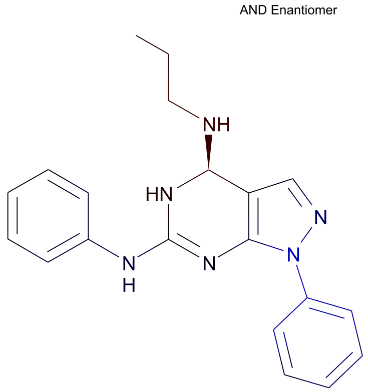

$C_{20}H_{22}N_6$
Molecular Weight: 346.42888
ALogP: 3.562
Rotatable Bonds: 6
Acceptors: 5
Donors: 3

Model Prediction

Prediction: Non-Toxic
Probability: 0.39
Enrichment: 0.742
Bayesian Score: -4.76
Mahalanobis Distance: 12.6
Mahalanobis Distance p-value: 2.35e-006

Prediction: Positive if the Bayesian score is above the estimated best cutoff value from minimizing the false positive and false negative rate.
Probability: The esimated probability that the sample is in the positive category. This assumes that the Bayesian score follows a normal distribution and is different from the prediction using a cutoff.
Enrichment: An estimate of enrichment, that is, the increased likelihood (versus random) of this sample being in the category.
Bayesian Score: The standard Laplacian-modified Bayesian score.
Mahalanobis Distance: The Mahalanobis distance (MD) is the distance to the center of the training data. The larger the MD, the less trustworthy the prediction.
Mahalanobis Distance p-value: The p-value gives the fraction of training data with an MD greater than or equal to the one for the given sample, assuming normally distributed data. The smaller the p-value, the less trustworthy the prediciton. For highly non-normal X properties (e.g., fingerprints), the MD p-value is wildly inaccurate.

Structural Similar Compounds

Name	Dobutamine .HCl (Free base form)	Clebopride Malate	Amsacrine
Structure	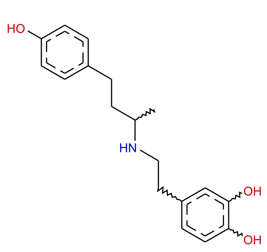	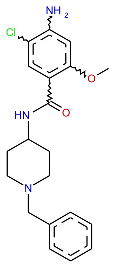	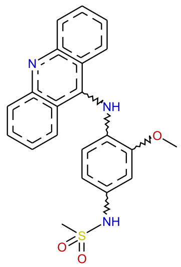
Actual Endpoint	Toxic	Non-Toxic	Toxic
Predicted Endpoint	Toxic	Non-Toxic	Toxic
Distance	0.625	0.628	0.630
Reference	Yakuri to Chiryo 7:1707-1730; 1979	Kiso to Rinsho 16:5649-5660; 1982	Fundam Appl Toxicol 7(2):214-20; 1986

Model Applicability

Unknown features are fingerprint features in the query molecule, but not found or appearing too infrequently in the training set.

1. All properties and OPS components are within expected ranges.

Feature Contribution

Top features for positive contribution

Fingerprint	Bit/Smiles	Feature Structure	Score	Toxic in training set
SCFP_6	-627385064	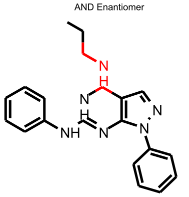 [*]CNC([*])[*]	0.414	14 out of 17

SCFP_6	-788967098	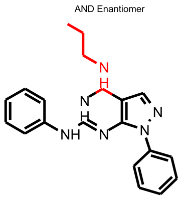 [*]C([*])NCCC	0.381	2 out of 2
SCFP_6	2088704928	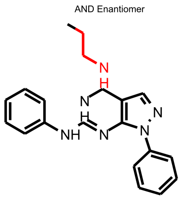 [*]NCC	0.303	38 out of 53
**Top Features for negative contribution**				
**Fingerprint**	**Bit/Smiles**	**Feature Structure**	**Score**	**Toxic in training set**
SCFP_6	-1378896970	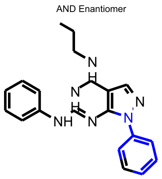 [*]:n(:[*])[c]1:[cH]:[*]:[cH]:[cH]:[cH]:1	-1.13	0 out of 4
SCFP_6	1076504043	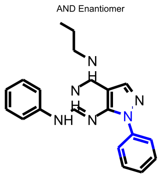 [*]:[cH]:[c](:[cH]:[*])n(:[*]):[*]	-1.13	0 out of 4
SCFP_6	-1380909229	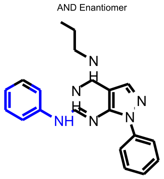 [*]N[c]1:[cH]:[*]:[cH]:[cH]:[cH]:1	-0.449	6 out of 19

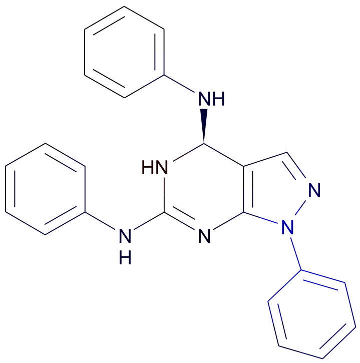

$C_{23}H_{20}N_6$
Molecular Weight: 380.4451
ALogP: 4.544
Rotatable Bonds: 5
Acceptors: 5
Donors: 3

Model Prediction

Prediction: Non-Toxic
Probability: 0.343
Enrichment: 0.653
Bayesian Score: -6.63
Mahalanobis Distance: 11.8
Mahalanobis Distance p-value: 5.8e-005

Prediction: Positive if the Bayesian score is above the estimated best cutoff value from minimizing the false positive and false negative rate.
Probability: The esimated probability that the sample is in the positive category. This assumes that the Bayesian score follows a normal distribution and is different from the prediction using a cutoff.
Enrichment: An estimate of enrichment, that is, the increased likelihood (versus random) of this sample being in the category.
Bayesian Score: The standard Laplacian-modified Bayesian score.
Mahalanobis Distance: The Mahalanobis distance (MD) is the distance to the center of the training data. The larger the MD, the less trustworthy the prediction.
Mahalanobis Distance p-value: The p-value gives the fraction of training data with an MD greater than or equal to the one for the given sample, assuming normally distributed data. The smaller the p-value, the less trustworthy the prediciton. For highly non-normal X properties (e.g., fingerprints), the MD p-value is wildly inaccurate.

Structural Similar Compounds

Name	Chenodiol	Amsacrine	Ochratoxin a
Structure	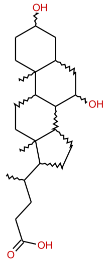	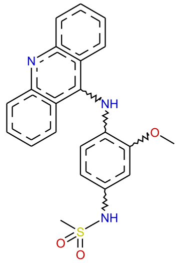	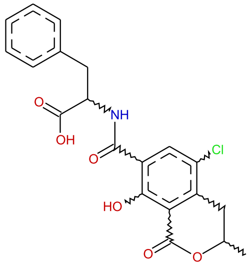
Actual Endpoint	Toxic	Toxic	Toxic
Predicted Endpoint	Toxic	Toxic	Toxic
Distance	0.592	0.625	0.674
Reference	Arch Int Pharm 246:149-158; 1980	Fundam Appl Toxicol 7(2):214-20; 1986	Toxicol Appl Pharmacol 37(2):331-8; 1976

Model Applicability

Unknown features are fingerprint features in the query molecule, but not found or appearing too infrequently in the training set.

1. All properties and OPS components are within expected ranges.

Feature Contribution

Top features for positive contribution				
Fingerprint	Bit/Smiles	Feature Structure	Score	Toxic in training set
SCFP_6	-627355273	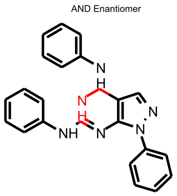 [*]C([*])NC(=[*])[*]	0.255	3 out of 4

SCFP_6	5	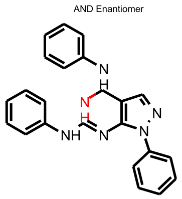 [*]N[*]	0.228	51 out of 77

Top Features for negative contribution

Fingerprint	Bit/Smiles	Feature Structure	Score	Toxic in training set
SCFP_6	-1378896970	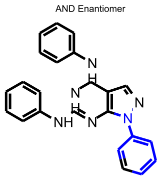 [*]:n(:[*])[c]1:[cH]:[*]:[cH]:[cH]:[cH]:1	-1.13	0 out of 4
SCFP_6	1076504043	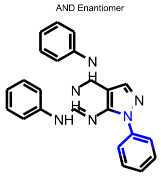 [*]:[cH]:[c](:[cH]:[*])n(:[*]):[*]	-1.13	0 out of 4
SCFP_6	18117904	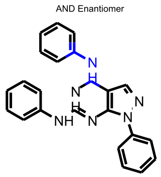 [*]C([*])N[c](:[*]):[*]	-0.945	0 out of 3

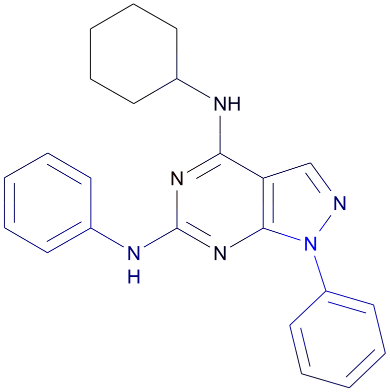

$C_{23}H_{24}N_6$

Molecular Weight: 384.47686

ALogP: 5.953

Rotatable Bonds: 5

Acceptors: 5

Donors: 2

Structural Similar Compounds

Name	Amsacrine	Perphenazine	Meclofenamate Sodium (Free acid form)
Structure	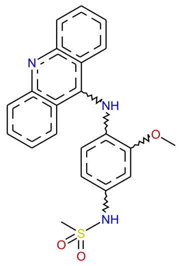	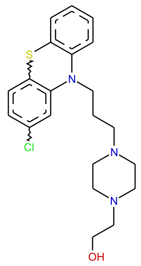	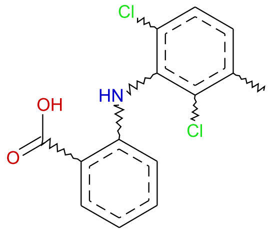
Actual Endpoint	Toxic	Toxic	Non-Toxic
Predicted Endpoint	Toxic	Toxic	Non-Toxic
Distance	0.658	0.684	0.687
Reference	Fundam Appl Toxicol 7(2):214-20; 1986	Toxicol Appl Pharmacol 21(2):230-6; 1972	Fundam Appl Toxicol 5:665-671; 1985

Model Prediction

Prediction: Non-Toxic

Probability: 0.274

Enrichment: 0.521

Bayesian Score: -9.37

Mahalanobis Distance: 9.32

Mahalanobis Distance p-value: 0.0987

Prediction: Positive if the Bayesian score is above the estimated best cutoff value from minimizing the false positive and false negative rate.
Probability: The esimated probability that the sample is in the positive category. This assumes that the Bayesian score follows a normal distribution and is different from the prediction using a cutoff.
Enrichment: An estimate of enrichment, that is, the increased likelihood (versus random) of this sample being in the category.
Bayesian Score: The standard Laplacian-modified Bayesian score.
Mahalanobis Distance: The Mahalanobis distance (MD) is the distance to the center of the training data. The larger the MD, the less trustworthy the prediction.
Mahalanobis Distance p-value: The p-value gives the fraction of training data with an MD greater than or equal to the one for the given sample, assuming normally distributed data. The smaller the p-value, the less trustworthy the prediciton. For highly non-normal X properties (e.g., fingerprints), the MD p-value is wildly inaccurate.

Model Applicability

Unknown features are fingerprint features in the query molecule, but not found or appearing too infrequently in the training set.

1. OPS PC8 out of range. Value: 4.8661. Training min, max, SD, explained variance: -3.1563, 4.7297, 1.569, 0.0361.

Feature Contribution

Top features for positive contribution

Fingerprint	Bit/Smiles	Feature Structure	Score	Toxic in training set
SCFP_6	-1545804258	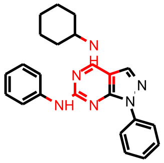 [*]N[c]1:n:[*]:[c](:[*]):[c](N[*]):n:1	0.381	2 out of 2

SCFP_6	-1272798659	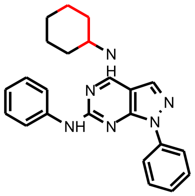 [*]CCC	0.0708	44 out of 78
SCFP_6	-1525101452	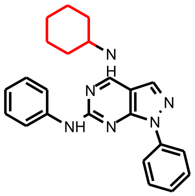 [*]C1CCCCC1	0.0677	4 out of 7

Top Features for negative contribution

Fingerprint	Bit/Smiles	Feature Structure	Score	Toxic in training set
SCFP_6	-1378896970	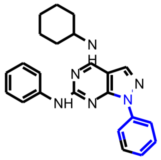 [*]:n(:[*])[c]1:[cH]:[*]:[cH]:[cH]:[cH]:1	-1.13	0 out of 4
SCFP_6	1076504043	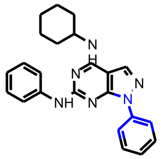 [*]:[cH]:[c](:[cH]:[*])n(:[*]):[*]	-1.13	0 out of 4
SCFP_6	18117904	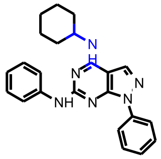 [*]C([*])N[c](:[*]):[*]	-0.945	0 out of 3

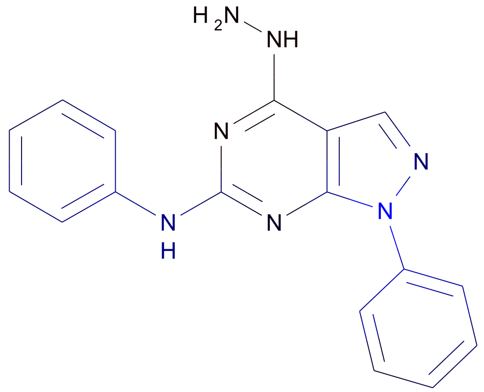

$C_{17}H_{15}N_7$
Molecular Weight: 317.3479
ALogP: 3.562
Rotatable Bonds: 4
Acceptors: 6
Donors: 3

Model Prediction

Prediction: Non-Toxic
Probability: 0.286
Enrichment: 0.544
Bayesian Score: -8.92
Mahalanobis Distance: 8.93
Mahalanobis Distance p-value: 0.198

Prediction: Positive if the Bayesian score is above the estimated best cutoff value from minimizing the false positive and false negative rate.
Probability: The esimated probability that the sample is in the positive category. This assumes that the Bayesian score follows a normal distribution and is different from the prediction using a cutoff.
Enrichment: An estimate of enrichment, that is, the increased likelihood (versus random) of this sample being in the category.
Bayesian Score: The standard Laplacian-modified Bayesian score.
Mahalanobis Distance: The Mahalanobis distance (MD) is the distance to the center of the training data. The larger the MD, the less trustworthy the prediction.
Mahalanobis Distance p-value: The p-value gives the fraction of training data with an MD greater than or equal to the one for the given sample, assuming normally distributed data. The smaller the p-value, the less trustworthy the prediciton. For highly non-normal X properties (e.g., fingerprints), the MD p-value is wildly inaccurate.

Structural Similar Compounds

Name	Ochratoxin a	Amsacrine	D&C Yellow 8
Structure	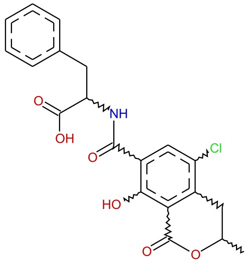	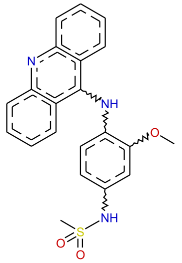	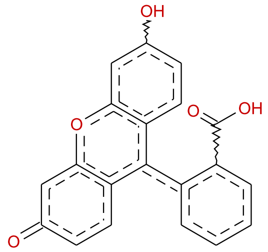
Actual Endpoint	Toxic	Toxic	Non-Toxic
Predicted Endpoint	Toxic	Toxic	Non-Toxic
Distance	0.611	0.655	0.681
Reference	Toxicol Appl Pharmacol 37(2):331-8; 1976	Fundam Appl Toxicol 7(2):214-20; 1986	Food Chem Toxicol 24:819-823; 1986

Model Applicability

Unknown features are fingerprint features in the query molecule, but not found or appearing too infrequently in the training set.

1. All properties and OPS components are within expected ranges.

Feature Contribution

Top features for positive contribution

Fingerprint	Bit/Smiles	Feature Structure	Score	Toxic in training set
SCFP_6	-1545804258	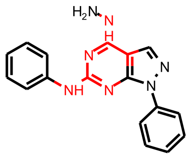 [*]N[c]1:n:[*]:[c](:[*]):[c](N[*]):n:1	0.381	2 out of 2

SCFP_6	5	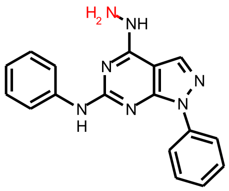 [*]N[*]	0.228	51 out of 77
**Top Features for negative contribution**				
**Fingerprint**	**Bit/Smiles**	**Feature Structure**	**Score**	**Toxic in training set**
SCFP_6	1076504043	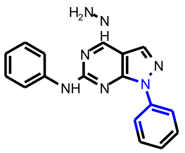 [*]:[cH]:[c](:[cH]:[*])n(:[*]):[*]	-1.13	0 out of 4
SCFP_6	-1378896970	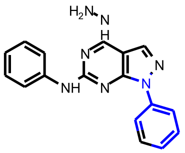 [*]:n(:[*])[c]1:[cH]:[*]:[cH]:[cH]:[cH]:1	-1.13	0 out of 4
SCFP_6	-572674910	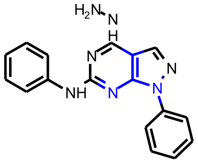 [*]n1:[*]:[*]:[c](:[*]):[c]:1:n:[*]	-0.945	0 out of 3

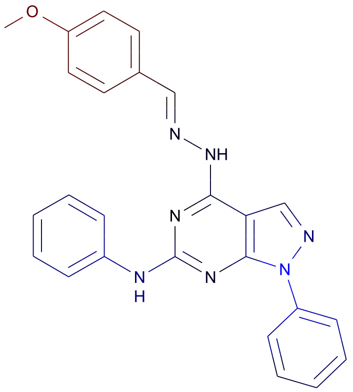

$C_{25}H_{21}N_7O$

Molecular Weight: 435.48054

ALogP: 5.889

Rotatable Bonds: 7

Acceptors: 7

Donors: 2

Model Prediction

Prediction: Non-Toxic

Probability: 0.338

Enrichment: 0.643

Bayesian Score: -6.86

Mahalanobis Distance: 10.4

Mahalanobis Distance p-value: 0.00806

Prediction: Positive if the Bayesian score is above the estimated best cutoff value from minimizing the false positive and false negative rate.
Probability: The esimated probability that the sample is in the positive category. This assumes that the Bayesian score follows a normal distribution and is different from the prediction using a cutoff.
Enrichment: An estimate of enrichment, that is, the increased likelihood (versus random) of this sample being in the category.
Bayesian Score: The standard Laplacian-modified Bayesian score.
Mahalanobis Distance: The Mahalanobis distance (MD) is the distance to the center of the training data. The larger the MD, the less trustworthy the prediction.
Mahalanobis Distance p-value: The p-value gives the fraction of training data with an MD greater than or equal to the one for the given sample, assuming normally distributed data. The smaller the p-value, the less trustworthy the prediciton. For highly non-normal X properties (e.g., fingerprints), the MD p-value is wildly inaccurate.

Structural Similar Compounds

Name	Estramustine Phosphate Disodium (Free acid form)	Acemetacin	Amsacrine
Structure	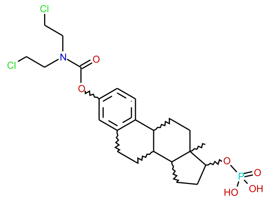	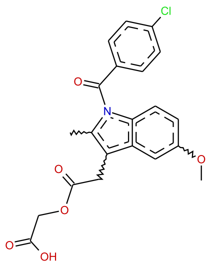	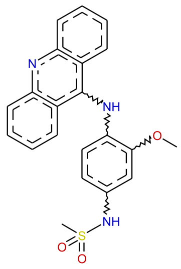
Actual Endpoint	Non-Toxic	Non-Toxic	Toxic
Predicted Endpoint	Non-Toxic	Non-Toxic	Toxic
Distance	0.627	0.672	0.697
Reference	Oyo Yakuri 20(6):1219-1236; 1980	Oyo Yakuri 22(6):777-786; 1981	Fundam Appl Toxicol 7(2):214-20; 1986

Model Applicability

Unknown features are fingerprint features in the query molecule, but not found or appearing too infrequently in the training set.

1. OPS PC17 out of range. Value: 3.4675. Training min, max, SD, explained variance: -2.7025, 2.8536, 1.067, 0.0167.

Feature Contribution

Top features for positive contribution

Fingerprint	Bit/Smiles	Feature Structure	Score	Toxic in training set
SCFP_6	1237755852	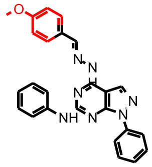 CO[c]1:[cH]:[cH]:[*]:[cH]:[cH]:1	0.453	8 out of 9

SCFP_6	591469355	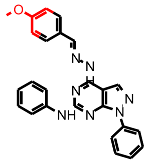 [*]:[cH]:[c](OC):[cH]:[*]	0.411	10 out of 12
SCFP_6	-1545804258	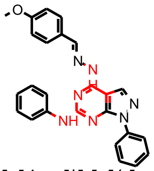 [*]N[c]1:n:[*]:[c](:[*]):[c](N[*]):n:1	0.381	2 out of 2

Top Features for negative contribution

Fingerprint	Bit/Smiles	Feature Structure	Score	Toxic in training set
SCFP_6	1076504043	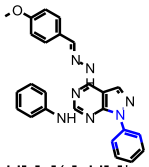 [*]:[cH]:[c](:[cH]:[*])n(:[*]):[*]	-1.13	0 out of 4
SCFP_6	-1378896970	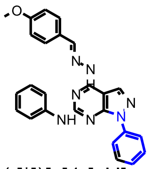 [*]:n(:[*])[c]1:[cH]:[*]:[cH]:[cH]:[cH]:1	-1.13	0 out of 4
SCFP_6	-572674910	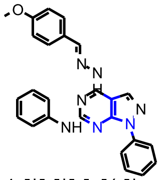 [*]n1:[*]:[*]:[c](:[*]):[c]:1:n:[*]	-0.945	0 out of 3

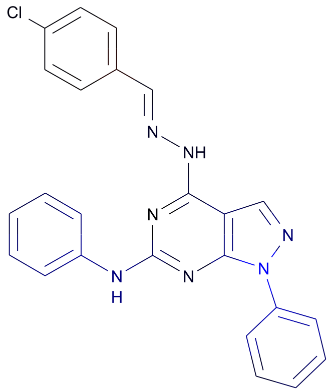

$C_{24}H_{18}ClN_7$
Molecular Weight: 439.89962
ALogP: 6.57
Rotatable Bonds: 6
Acceptors: 6
Donors: 2

Model Prediction

Prediction: Non-Toxic
Probability: 0.274
Enrichment: 0.521
Bayesian Score: -9.37
Mahalanobis Distance: 9.69
Mahalanobis Distance p-value: 0.0449

Prediction: Positive if the Bayesian score is above the estimated best cutoff value from minimizing the false positive and false negative rate.
Probability: The esimated probability that the sample is in the positive category. This assumes that the Bayesian score follows a normal distribution and is different from the prediction using a cutoff.
Enrichment: An estimate of enrichment, that is, the increased likelihood (versus random) of this sample being in the category.
Bayesian Score: The standard Laplacian-modified Bayesian score.
Mahalanobis Distance: The Mahalanobis distance (MD) is the distance to the center of the training data. The larger the MD, the less trustworthy the prediction.
Mahalanobis Distance p-value: The p-value gives the fraction of training data with an MD greater than or equal to the one for the given sample, assuming normally distributed data. The smaller the p-value, the less trustworthy the prediciton. For highly non-normal X properties (e.g., fingerprints), the MD p-value is wildly inaccurate.

Structural Similar Compounds

Name	Estramustine Phosphate Disodium (Free acid form)	Perphenazine	Acemetacin
Structure	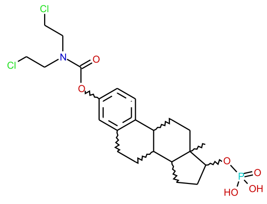	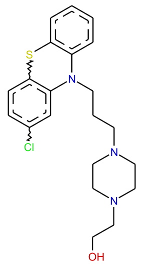	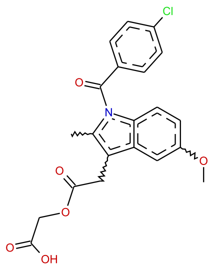
Actual Endpoint	Non-Toxic	Toxic	Non-Toxic
Predicted Endpoint	Non-Toxic	Toxic	Non-Toxic
Distance	0.683	0.726	0.728
Reference	Oyo Yakuri 20(6):1219-1236; 1980	Toxicol Appl Pharmacol 21(2):230-6; 1972	Oyo Yakuri 22(6):777-786; 1981

Model Applicability

Unknown features are fingerprint features in the query molecule, but not found or appearing too infrequently in the training set.

1. All properties and OPS components are within expected ranges.

Feature Contribution

Top features for positive contribution

Fingerprint	Bit/Smiles	Feature Structure	Score	Toxic in training set
SCFP_6	-1545804258	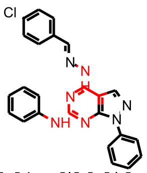 [*]N[c]1:n:[*]:[c](:[*]):[c](N[*]):n:1	0.381	2 out of 2

SCFP_6	1390366442	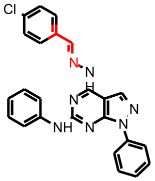<br>[*]\N=C\[c](:[*]):[*]	0.381	2 out of 2
SCFP_6	-650738059	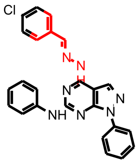<br>[*]N\N=C\[c](:[cH]:[*<br>]):[cH]:[*]	0.271	1 out of 1

Top Features for negative contribution

Fingerprint	Bit/Smiles	Feature Structure	Score	Toxic in training set
SCFP_6	-1378896970	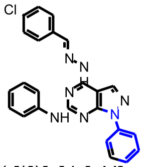<br>[*]:n(:[*])[c]1:[cH]:<br>[*]:[cH]:[cH]:[cH]:1	-1.13	0 out of 4
SCFP_6	1076504043	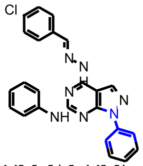<br>[*]:[cH]:[c](:[cH]:[*<br>])n(:[*]):[*]	-1.13	0 out of 4
SCFP_6	-572674910	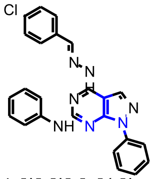<br>[*]n1:[*]:[*]:[c](:[*<br>]):[c]:1:n:[*]	-0.945	0 out of 3

12a TOPKAT_Developmental_Toxicity_Potential

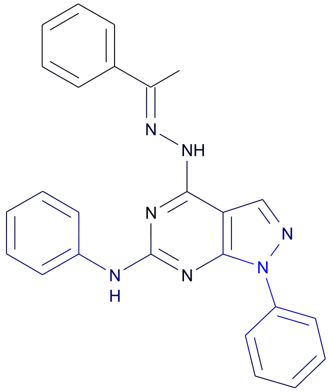

$C_{25}H_{21}N_7$

Molecular Weight: 419.48114

ALogP: 5.886

Rotatable Bonds: 6

Acceptors: 6

Donors: 2

Structural Similar Compounds

Name	Amsacrine	Estramustine Phosphate Disodium (Free acid form)	Perphenazine
Structure	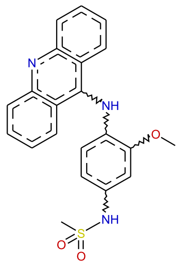	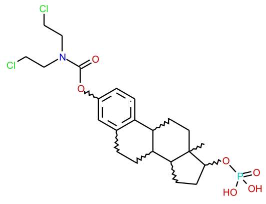	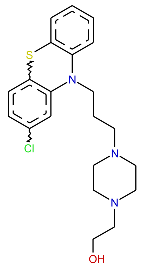
Actual Endpoint	Toxic	Non-Toxic	Toxic
Predicted Endpoint	Toxic	Non-Toxic	Toxic
Distance	0.671	0.672	0.693
Reference	Fundam Appl Toxicol 7(2):214-20; 1986	Oyo Yakuri 20(6):1219-1236; 1980	Toxicol Appl Pharmacol 21(2):230-6; 1972

Model Prediction

Prediction: Non-Toxic

Probability: 0.263

Enrichment: 0.501

Bayesian Score: -9.79

Mahalanobis Distance: 10.3

Mahalanobis Distance p-value: 0.00896

Prediction: Positive if the Bayesian score is above the estimated best cutoff value from minimizing the false positive and false negative rate.
Probability: The esimated probability that the sample is in the positive category. This assumes that the Bayesian score follows a normal distribution and is different from the prediction using a cutoff.
Enrichment: An estimate of enrichment, that is, the increased likelihood (versus random) of this sample being in the category.
Bayesian Score: The standard Laplacian-modified Bayesian score.
Mahalanobis Distance: The Mahalanobis distance (MD) is the distance to the center of the training data. The larger the MD, the less trustworthy the prediction.
Mahalanobis Distance p-value: The p-value gives the fraction of training data with an MD greater than or equal to the one for the given sample, assuming normally distributed data. The smaller the p-value, the less trustworthy the prediciton. For highly non-normal X properties (e.g., fingerprints), the MD p-value is wildly inaccurate.

Model Applicability

Unknown features are fingerprint features in the query molecule, but not found or appearing too infrequently in the training set.

1. OPS PC17 out of range. Value: 3.0133. Training min, max, SD, explained variance: -2.7025, 2.8536, 1.067, 0.0167.

Feature Contribution

Top features for positive contribution				
**Fingerprint**	**Bit/Smiles**	**Feature Structure**	**Score**	**Toxic in training set**
SCFP_6	-1545804258	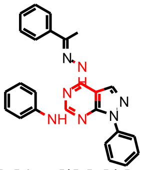 [*]N[c]1:n:[*]:[c](:[*]):[c](N[*]):n:1	0.381	2 out of 2

SCFP_6	-331724199	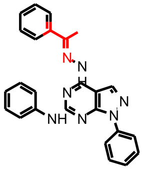 [*]\N=C(/C)\[c](:[*]) :[*]	0.271	1 out of 1
SCFP_6	-2056718782	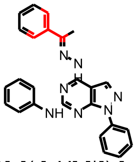 [*]=C[c](:[cH]:[*]):[cH]:[*]	0.0786	33 out of 58
**Top Features for negative contribution**				
**Fingerprint**	**Bit/Smiles**	**Feature Structure**	**Score**	**Toxic in training set**
SCFP_6	1076504043	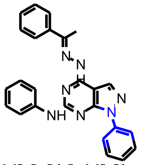 [*]:[cH]:[c](:[cH]:[*])n(:[*]):[*]	-1.13	0 out of 4
SCFP_6	-1378896970	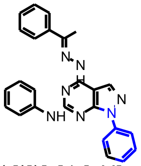 [*]:n(:[*])[c]1:[cH]: [*]:[cH]:[cH]:[cH]:1	-1.13	0 out of 4
SCFP_6	-572674910	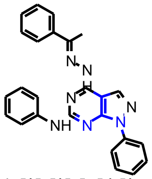 [*]n1:[*]:[*]:[c](:[*]):[c]:1:n:[*]	-0.945	0 out of 3

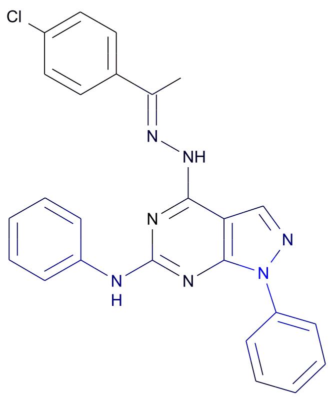

$C_{25}H_{20}ClN_7$
Molecular Weight: 453.9262
ALogP: 6.55
Rotatable Bonds: 6
Acceptors: 6
Donors: 2

Model Prediction

Prediction: Non-Toxic
Probability: 0.262
Enrichment: 0.498
Bayesian Score: -9.84
Mahalanobis Distance: 10.7
Mahalanobis Distance p-value: 0.00255

Prediction: Positive if the Bayesian score is above the estimated best cutoff value from minimizing the false positive and false negative rate.
Probability: The esimated probability that the sample is in the positive category. This assumes that the Bayesian score follows a normal distribution and is different from the prediction using a cutoff.
Enrichment: An estimate of enrichment, that is, the increased likelihood (versus random) of this sample being in the category.
Bayesian Score: The standard Laplacian-modified Bayesian score.
Mahalanobis Distance: The Mahalanobis distance (MD) is the distance to the center of the training data. The larger the MD, the less trustworthy the prediction.
Mahalanobis Distance p-value: The p-value gives the fraction of training data with an MD greater than or equal to the one for the given sample, assuming normally distributed data. The smaller the p-value, the less trustworthy the prediciton. For highly non-normal X properties (e.g., fingerprints), the MD p-value is wildly inaccurate.

Structural Similar Compounds

Name	Estramustine Phosphate Disodium (Free acid form)	Perphenazine	Acemetacin
Structure	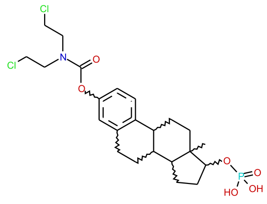	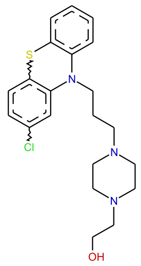	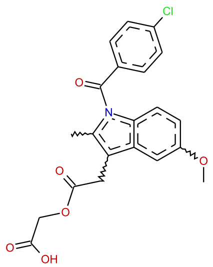
Actual Endpoint	Non-Toxic	Toxic	Non-Toxic
Predicted Endpoint	Non-Toxic	Toxic	Non-Toxic
Distance	0.672	0.722	0.728
Reference	Oyo Yakuri 20(6):1219-1236; 1980	Toxicol Appl Pharmacol 21(2):230-6; 1972	Oyo Yakuri 22(6):777-786; 1981

Model Applicability

Unknown features are fingerprint features in the query molecule, but not found or appearing too infrequently in the training set.

1. All properties and OPS components are within expected ranges.

Feature Contribution

Top features for positive contribution

Fingerprint	Bit/Smiles	Feature Structure	Score	Toxic in training set
SCFP_6	-1545804258	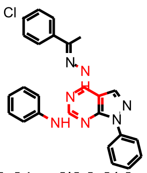 [*]N[c]1:n:[*]:[c](:[*]):[c](N[*]):n:1	0.381	2 out of 2

SCFP_6	-331724199	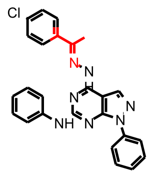 [*]\N=C(/C)\[c](:[*]) :[*]	0.271	1 out of 1
SCFP_6	1905487031	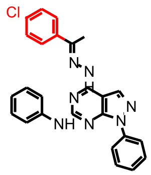 [*][c]1:[cH]:[cH]:[c] (Cl):[cH]:[cH]:1	0.153	2 out of 3
**Top Features for negative contribution**				
**Fingerprint**	**Bit/Smiles**	**Feature Structure**	**Score**	**Toxic in training set**
SCFP_6	-1378896970	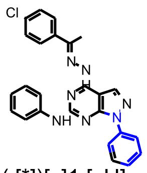 [*]:n(:[*])[c]1:[cH]: [*]:[cH]:[cH]:[cH]:1	-1.13	0 out of 4
SCFP_6	1076504043	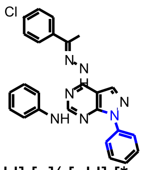 [*]:[cH]:[c](:[cH]:[*])n(:[*]):[*]	-1.13	0 out of 4
SCFP_6	-572674910	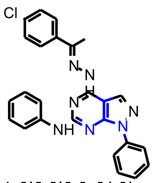 [*]n1:[*]:[*]:[c](:[*]):[c]:1:n:[*]	-0.945	0 out of 3

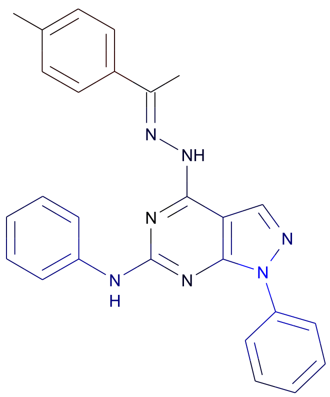

$C_{26}H_{23}N_7$

Molecular Weight: 433.50772

ALogP: 6.372

Rotatable Bonds: 6

Acceptors: 6

Donors: 2

Model Prediction

Prediction: Non-Toxic

Probability: 0.275

Enrichment: 0.522

Bayesian Score: -9.35

Mahalanobis Distance: 11.1

Mahalanobis Distance p-value: 0.000665

Prediction: Positive if the Bayesian score is above the estimated best cutoff value from minimizing the false positive and false negative rate.
Probability: The esimated probability that the sample is in the positive category. This assumes that the Bayesian score follows a normal distribution and is different from the prediction using a cutoff.
Enrichment: An estimate of enrichment, that is, the increased likelihood (versus random) of this sample being in the category.
Bayesian Score: The standard Laplacian-modified Bayesian score.
Mahalanobis Distance: The Mahalanobis distance (MD) is the distance to the center of the training data. The larger the MD, the less trustworthy the prediction.
Mahalanobis Distance p-value: The p-value gives the fraction of training data with an MD greater than or equal to the one for the given sample, assuming normally distributed data. The smaller the p-value, the less trustworthy the prediciton. For highly non-normal X properties (e.g., fingerprints), the MD p-value is wildly inaccurate.

Structural Similar Compounds

Name	Estramustine Phosphate Disodium (Free acid form)	Amsacrine	Perphenazine
Structure	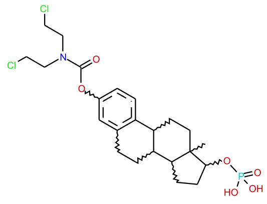	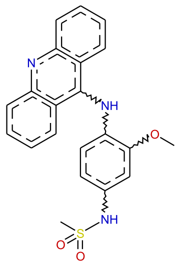	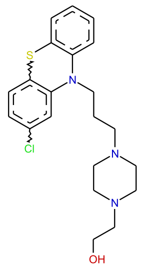
Actual Endpoint	Non-Toxic	Toxic	Toxic
Predicted Endpoint	Non-Toxic	Toxic	Toxic
Distance	0.674	0.719	0.726
Reference	Oyo Yakuri 20(6):1219-1236; 1980	Fundam Appl Toxicol 7(2):214-20; 1986	Toxicol Appl Pharmacol 21(2):230-6; 1972

Model Applicability

Unknown features are fingerprint features in the query molecule, but not found or appearing too infrequently in the training set.

1. All properties and OPS components are within expected ranges.

Feature Contribution

Top features for positive contribution

Fingerprint	Bit/Smiles	Feature Structure	Score	Toxic in training set
SCFP_6	-1545804258	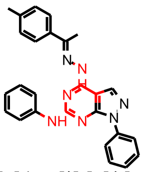 [*]N[c]1:n:[*]:[c](:[*]):[c](N[*]):n:1	0.381	2 out of 2

SCFP_6	2048398673	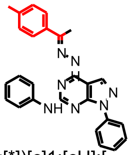<br>[*]C(=[*])[c]1:[cH]:[<br>cH]:[c](C):[cH]:[cH]<br>:1	0.271	1 out of 1
SCFP_6	795925860	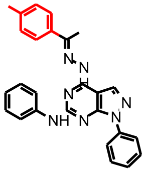<br>[*][c]1:[cH]:[cH]:[c]<br>(C):[cH]:[cH]:1	0.271	1 out of 1

Top Features for negative contribution

Fingerprint	Bit/Smiles	Feature Structure	Score	Toxic in training set
SCFP_6	1076504043	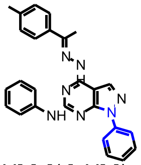<br>[*]:[cH]:[c](:[cH]:[*<br>])n(:[*]):[*]	-1.13	0 out of 4
SCFP_6	-1378896970	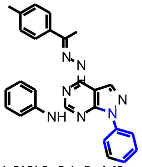<br>[*]:n(:[*])[c]1:[cH]:<br>[*]:[cH]:[cH]:[cH]:1	-1.13	0 out of 4
SCFP_6	-572674910	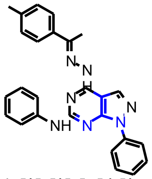<br>[*]n1:[*]:[*]:[c](:[*<br>]):[c]:1:n:[*]	-0.945	0 out of 3

13a

TOPKAT_Developmental_Toxicity_Potential

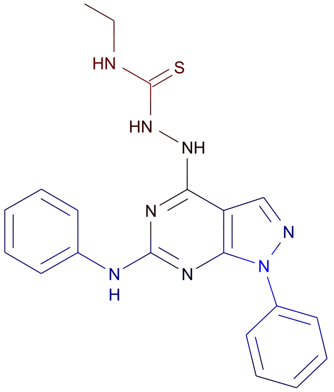

$C_{20}H_{20}N_8S$
Molecular Weight: 404.4914
ALogP: 4.99
Rotatable Bonds: 8
Acceptors: 6
Donors: 4

Model Prediction

Prediction: Non-Toxic
Probability: 0.366
Enrichment: 0.697
Bayesian Score: -5.67
Mahalanobis Distance: 10.6
Mahalanobis Distance p-value: 0.00349

Prediction: Positive if the Bayesian score is above the estimated best cutoff value from minimizing the false positive and false negative rate.
Probability: The esimated probability that the sample is in the positive category. This assumes that the Bayesian score follows a normal distribution and is different from the prediction using a cutoff.
Enrichment: An estimate of enrichment, that is, the increased likelihood (versus random) of this sample being in the category.
Bayesian Score: The standard Laplacian-modified Bayesian score.
Mahalanobis Distance: The Mahalanobis distance (MD) is the distance to the center of the training data. The larger the MD, the less trustworthy the prediction.
Mahalanobis Distance p-value: The p-value gives the fraction of training data with an MD greater than or equal to the one for the given sample, assuming normally distributed data. The smaller the p-value, the less trustworthy the prediciton. For highly non-normal X properties (e.g., fingerprints), the MD p-value is wildly inaccurate.

Structural Similar Compounds

Name	Dobutamine .HCl (Free base form)	Ochratoxin a	Amsacrine
Structure	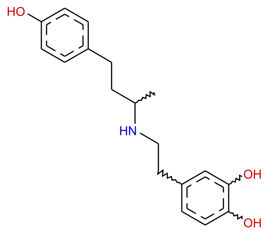	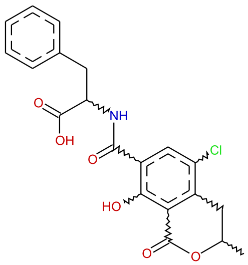	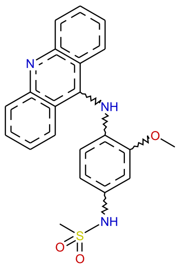
Actual Endpoint	Toxic	Toxic	Toxic
Predicted Endpoint	Toxic	Toxic	Toxic
Distance	0.737	0.738	0.801
Reference	Yakuri to Chiryo 7:1707-1730; 1979	Toxicol Appl Pharmacol 37(2):331-8; 1976	Fundam Appl Toxicol 7(2):214-20; 1986

Model Applicability

Unknown features are fingerprint features in the query molecule, but not found or appearing too infrequently in the training set.

1. All properties and OPS components are within expected ranges.

Feature Contribution

Top features for positive contribution				
**Fingerprint**	**Bit/Smiles**	**Feature Structure**	**Score**	**Toxic in training set**
SCFP_6	1435188938	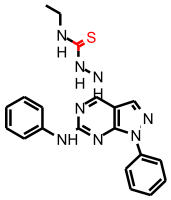 [*]C(=S)[*]	0.478	4 out of 4

SCFP_6	18058322	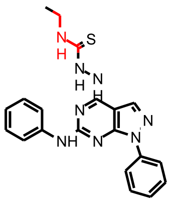 [*]CNC(=[*])[*]	0.478	4 out of 4
SCFP_6	382734644	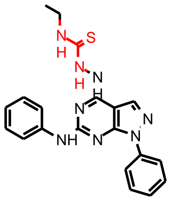 [*]NC(=S)N[*]	0.478	4 out of 4
**Top Features for negative contribution**				
**Fingerprint**	**Bit/Smiles**	**Feature Structure**	**Score**	**Toxic in training set**
SCFP_6	-1378896970	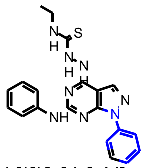 [*]:n(:[*])[c]1:[cH]:[*]:[cH]:[cH]:[cH]:1	-1.13	0 out of 4
SCFP_6	1076504043	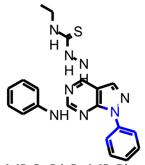 [*]:[cH]:[c](:[cH]:[*])n(:[*]):[*]	-1.13	0 out of 4
SCFP_6	-572674910	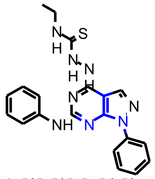 [*]n1:[*]:[*]:[c](:[*]):[c]:1:n:[*]	-0.945	0 out of 3

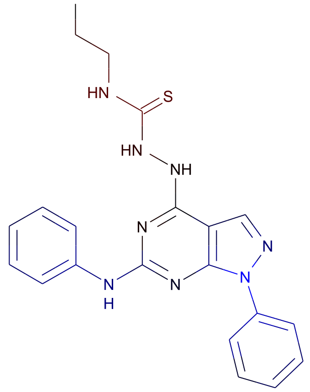

$C_{21}H_{22}N_8S$
Molecular Weight: 418.51798
ALogP: 5.514
Rotatable Bonds: 9
Acceptors: 6
Donors: 4

Model Prediction

Prediction: Non-Toxic
Probability: 0.361
Enrichment: 0.687
Bayesian Score: -5.87
Mahalanobis Distance: 11.2
Mahalanobis Distance p-value: 0.000555

Prediction: Positive if the Bayesian score is above the estimated best cutoff value from minimizing the false positive and false negative rate.
Probability: The esimated probability that the sample is in the positive category. This assumes that the Bayesian score follows a normal distribution and is different from the prediction using a cutoff.
Enrichment: An estimate of enrichment, that is, the increased likelihood (versus random) of this sample being in the category.
Bayesian Score: The standard Laplacian-modified Bayesian score.
Mahalanobis Distance: The Mahalanobis distance (MD) is the distance to the center of the training data. The larger the MD, the less trustworthy the prediction.
Mahalanobis Distance p-value: The p-value gives the fraction of training data with an MD greater than or equal to the one for the given sample, assuming normally distributed data. The smaller the p-value, the less trustworthy the prediciton. For highly non-normal X properties (e.g., fingerprints), the MD p-value is wildly inaccurate.

Structural Similar Compounds

Name	Dobutamine .HCl (Free base form)	Estramustine Phosphate Disodium (Free acid form)	Ochratoxin a
Structure	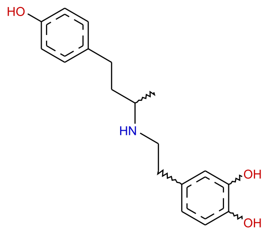	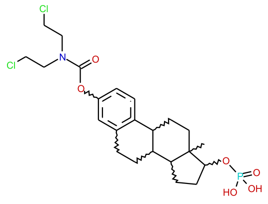	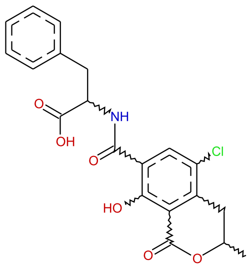
Actual Endpoint	Toxic	Non-Toxic	Toxic
Predicted Endpoint	Toxic	Non-Toxic	Toxic
Distance	0.778	0.792	0.807
Reference	Yakuri to Chiryo 7:1707-1730; 1979	Oyo Yakuri 20(6):1219-1236; 1980	Toxicol Appl Pharmacol 37(2):331-8; 1976

Model Applicability

Unknown features are fingerprint features in the query molecule, but not found or appearing too infrequently in the training set.

1. All properties and OPS components are within expected ranges.

Feature Contribution

Top features for positive contribution

Fingerprint	Bit/Smiles	Feature Structure	Score	Toxic in training set
SCFP_6	382734644	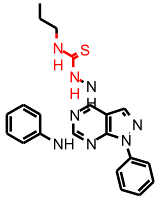 [*]NC(=S)N[*]	0.478	4 out of 4

SCFP_6	18058322	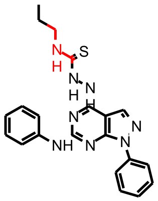 [*]CNC(=[*])[*]	0.478	4 out of 4
SCFP_6	1435188938	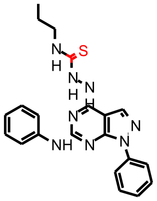 [*]C(=S)[*]	0.478	4 out of 4

Top Features for negative contribution

**Fingerprint**	**Bit/Smiles**	**Feature Structure**	**Score**	**Toxic in training set**
SCFP_6	1076504043	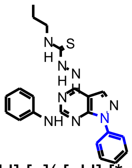 [*]:[cH]:[c](:[cH]:[*])n(:[*]):[*]	-1.13	0 out of 4
SCFP_6	-1378896970	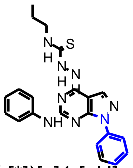 [*]:n(:[*])[c]1:[cH]:[*]:[cH]:[cH]:[cH]:1	-1.13	0 out of 4
SCFP_6	-572674910	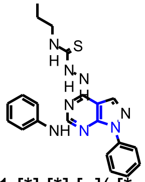 [*]n1:[*]:[*]:[c](:[*]):[c]:1:n:[*]	-0.945	0 out of 3

Erlotinib

TOPKAT_Developmental_Toxicity_Potential

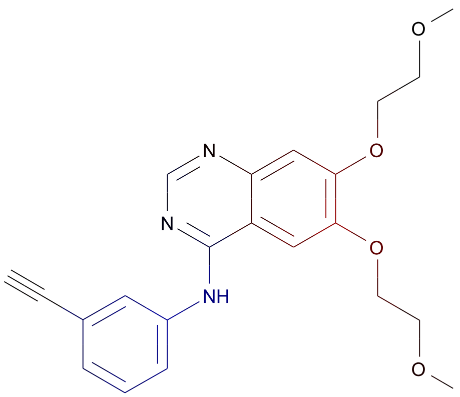

$C_{22}H_{23}N_3O_4$
Molecular Weight: 393.43572
ALogP: 4.309
Rotatable Bonds: 10
Acceptors: 7
Donors: 1

Model Prediction

Prediction: Non-Toxic
Probability: 0.504
Enrichment: 0.959
Bayesian Score: -1.17
Mahalanobis Distance: 8.76
Mahalanobis Distance p-value: 0.258

Prediction: Positive if the Bayesian score is above the estimated best cutoff value from minimizing the false positive and false negative rate.
Probability: The esimated probability that the sample is in the positive category. This assumes that the Bayesian score follows a normal distribution and is different from the prediction using a cutoff.
Enrichment: An estimate of enrichment, that is, the increased likelihood (versus random) of this sample being in the category.
Bayesian Score: The standard Laplacian-modified Bayesian score.
Mahalanobis Distance: The Mahalanobis distance (MD) is the distance to the center of the training data. The larger the MD, the less trustworthy the prediction.
Mahalanobis Distance p-value: The p-value gives the fraction of training data with an MD greater than or equal to the one for the given sample, assuming normally distributed data. The smaller the p-value, the less trustworthy the prediciton. For highly non-normal X properties (e.g., fingerprints), the MD p-value is wildly inaccurate.

Structural Similar Compounds

Name	Nicardipine	Suxibuzone	Etofenamate
Structure	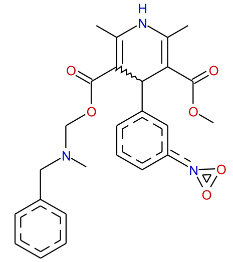	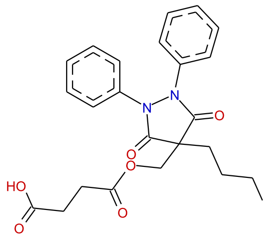	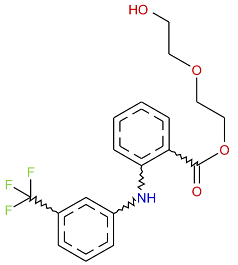
Actual Endpoint	Non-Toxic	Toxic	Non-Toxic
Predicted Endpoint	Non-Toxic	Toxic	Non-Toxic
Distance	0.608	0.620	0.635
Reference	Kiso to Rinsho 13:1149-1159; 1979	Oyo Yakuri 20:377-386; 1980	Iyakuhin Kenkyu 13(4):896-909; 1982

Model Applicability

Unknown features are fingerprint features in the query molecule, but not found or appearing too infrequently in the training set.

1. All properties and OPS components are within expected ranges.

Feature Contribution

Top features for positive contribution

Fingerprint	Bit/Smiles	Feature Structure	Score	Toxic in training set
SCFP_6	123285475	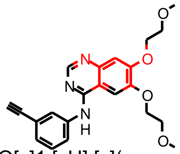 [*]O[c]1:[cH]:[c](:n:[*]):[c](:[*]):[*]:[c]:1[*]	0.478	4 out of 4

SCFP_6	446954673	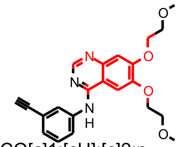<br>[*]CO[c]1:[cH]:[c]2:n:[cH]:[*]:[c]([*]):[c]:2:[cH]:[c]:1O[*]	0.381	2 out of 2
SCFP_6	-1814968949	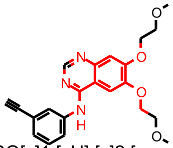<br>[*]CO[c]1:[cH]:[c]2:[c](N[*]):n:[*]:n:[c]:2:[cH]:[c]:1O[*]	0.381	2 out of 2

Top Features for negative contribution

Fingerprint	Bit/Smiles	Feature Structure	Score	Toxic in training set
SCFP_6	-2020651081	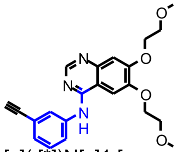<br>[*]:[c](:[*])N[c]1:[cH]:[cH]:[cH]:[cH]:[cH]:1	-0.718	0 out of 2
SCFP_6	-300914917	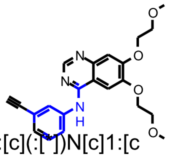<br>[*]:[c](:[*])N[c]1:[cH]:[cH]:[*]:[cH]:[cH]:1	-0.718	0 out of 2
SCFP_6	2142015375	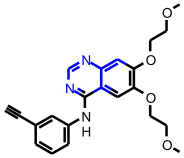<br>[*]:[cH]:[c]1:n:[cH]:n:[*]:[c]:1:[*]	-0.718	0 out of 2

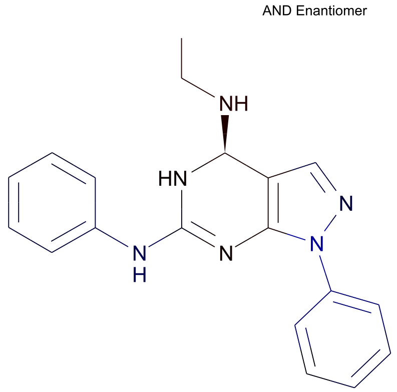

$C_{19}H_{20}N_6$
Molecular Weight: 332.4023
ALogP: 3.039
Rotatable Bonds: 5
Acceptors: 5
Donors: 3

Model Prediction

Prediction: Mild
Probability: 0.692
Enrichment: 1.01
Bayesian Score: -3.66
Mahalanobis Distance: 7.71
Mahalanobis Distance p-value: 0.968

Prediction: Positive if the Bayesian score is above the estimated best cutoff value from minimizing the false positive and false negative rate.
Probability: The esimated probability that the sample is in the positive category. This assumes that the Bayesian score follows a normal distribution and is different from the prediction using a cutoff.
Enrichment: An estimate of enrichment, that is, the increased likelihood (versus random) of this sample being in the category.
Bayesian Score: The standard Laplacian-modified Bayesian score.
Mahalanobis Distance: The Mahalanobis distance (MD) is the distance to the center of the training data. The larger the MD, the less trustworthy the prediction.
Mahalanobis Distance p-value: The p-value gives the fraction of training data with an MD greater than or equal to the one for the given sample, assuming normally distributed data. The smaller the p-value, the less trustworthy the prediciton. For highly non-normal X properties (e.g., fingerprints), the MD p-value is wildly inaccurate.

Structural Similar Compounds

Name	ANTHRAQUINONE; 1-((2-HYDROXYETHYL)AMINO)-4-(METHYLAMINO)-	METHANE;TRIS(4-AMINOPHENYL)-	1-AMINO-4-BENZOYLAMINO-ANTHRAQUINONE
Structure	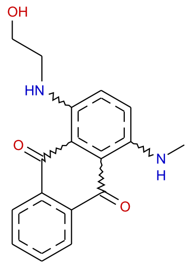	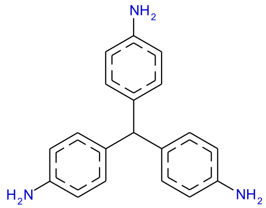	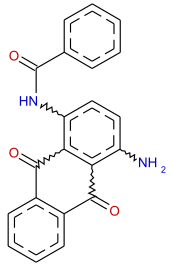
Actual Endpoint	Mild	Moderate_Severe	Mild
Predicted Endpoint	Mild	Moderate_Severe	Mild
Distance	0.616	0.686	0.738
Reference	28ZPAK 245;72	28ZPAK-;73;72	28ZPAK-;124;72

Model Applicability

Unknown features are fingerprint features in the query molecule, but not found or appearing too infrequently in the training set.

1. All properties and OPS components are within expected ranges.
2. Unknown FCFP_2 feature: 179977000: [*][c]1:[*]:[*]:n:n:1[c](:[*]):[*]
3. Unknown FCFP_2 feature: 2119857014: [*]NC(N[*])[c](:[*]):[*]

Feature Contribution

Top features for positive contribution

Fingerprint	Bit/Smiles	Feature Structure	Score	Moderate_Severe in training set
FCFP_10	-1939253119	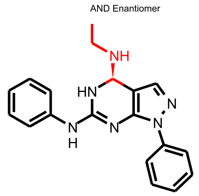 [*]C([*])NCC	0.332	5 out of 5

FCFP_10	-1272709286	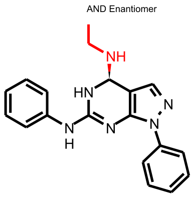 [*]NCC	0.285	234 out of 266
FCFP_10	-124655670	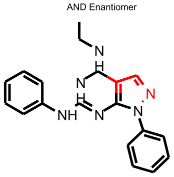 [*][c]1:[*]:[*]:n:[cH]:1	0.259	14 out of 16
**Top Features for negative contribution**				
**Fingerprint**	**Bit/Smiles**	**Feature Structure**	**Score**	**Moderate_Severe in training set**
FCFP_10	4427049	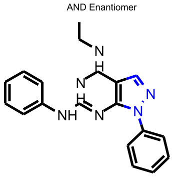 [*]n1:[*]:[*]:[cH]:n: 1	-1.29	0 out of 4
FCFP_10	-1732563065	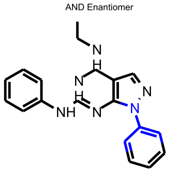 [*]:[cH]:[c](:[cH]:[*])n(:[*]):[*]	-1.09	0 out of 3
FCFP_10	1860261331	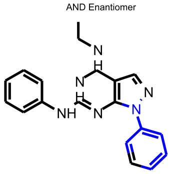 [*]:n(:[*])[c]1:[cH]: [*]:[cH]:[cH]:[cH]:1	-0.842	0 out of 2

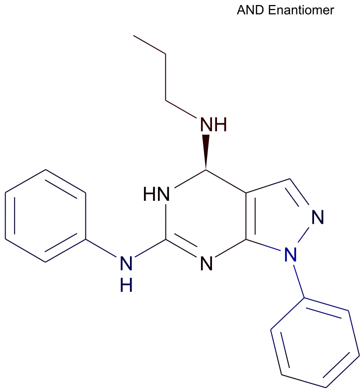

$C_{20}H_{22}N_6$
Molecular Weight: 346.42888
ALogP: 3.562
Rotatable Bonds: 6
Acceptors: 5
Donors: 3

Model Prediction

Prediction: Mild
Probability: 0.709
Enrichment: 1.03
Bayesian Score: -3.36
Mahalanobis Distance: 8.11
Mahalanobis Distance p-value: 0.898

Prediction: Positive if the Bayesian score is above the estimated best cutoff value from minimizing the false positive and false negative rate.
Probability: The esimated probability that the sample is in the positive category. This assumes that the Bayesian score follows a normal distribution and is different from the prediction using a cutoff.
Enrichment: An estimate of enrichment, that is, the increased likelihood (versus random) of this sample being in the category.
Bayesian Score: The standard Laplacian-modified Bayesian score.
Mahalanobis Distance: The Mahalanobis distance (MD) is the distance to the center of the training data. The larger the MD, the less trustworthy the prediction.
Mahalanobis Distance p-value: The p-value gives the fraction of training data with an MD greater than or equal to the one for the given sample, assuming normally distributed data. The smaller the p-value, the less trustworthy the prediciton. For highly non-normal X properties (e.g., fingerprints), the MD p-value is wildly inaccurate.

Structural Similar Compounds

Name	ANTHRAQUINONE; 1-((2-HYDROXYETHYL)AMINO)-4-(METHYLAMINO)-	METHANE;TRIS(4-AMINOPHENYL)-	ANTHRAQUINONE; 1-AMINO-4-HYDROXY-2-PHENOXY-
Structure	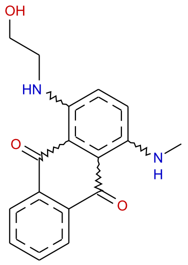	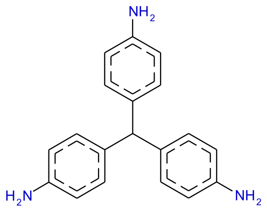	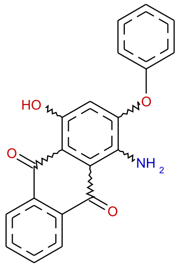
Actual Endpoint	Mild	Moderate_Severe	Mild
Predicted Endpoint	Mild	Moderate_Severe	Mild
Distance	0.680	0.720	0.760
Reference	28ZPAK 245;72	28ZPAK-;73;72	28ZPAK 239;72

Model Applicability

Unknown features are fingerprint features in the query molecule, but not found or appearing too infrequently in the training set.

1. All properties and OPS components are within expected ranges.
2. Unknown FCFP_2 feature: 179977000: [*][c]1:[*]:[*]:n:n:1[c](:[*]):[*]
3. Unknown FCFP_2 feature: 2119857014: [*]NC(N[*])[c](:[*]):[*]

Feature Contribution

Top features for positive contribution

Fingerprint	Bit/Smiles	Feature Structure	Score	Moderate_Severe in training set
FCFP_10	159404153	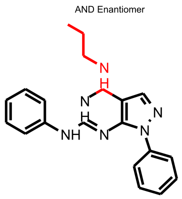 [*]C([*])NCCC	0.324	15 out of 16

FCFP_10	-1272709286	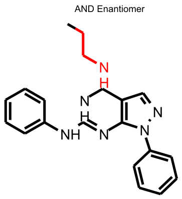 [*]NCC	0.285	234 out of 266
FCFP_10	-124655670	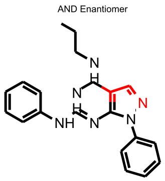 [*][c]1:[*]:[*]:n:[cH]:1	0.259	14 out of 16
**Top Features for negative contribution**				
**Fingerprint**	**Bit/Smiles**	**Feature Structure**	**Score**	**Moderate_Severe in training set**
FCFP_10	4427049	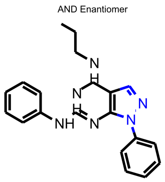 [*]n1:[*]:[*]:[cH]:n: 1	-1.29	0 out of 4
FCFP_10	-1732563065	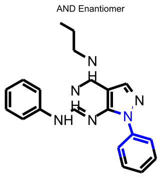 [*]:[cH]:[c](:[cH]:[*])n(:[*]):[*]	-1.09	0 out of 3
FCFP_10	1860261331	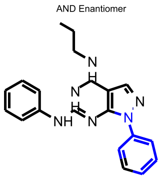 [*]:n(:[*])[c]1:[cH]: [*]:[cH]:[cH]:[cH]:1	-0.842	0 out of 2

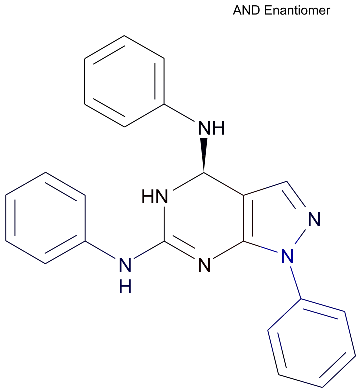

$C_{23}H_{20}N_6$
Molecular Weight: 380.4451
ALogP: 4.544
Rotatable Bonds: 5
Acceptors: 5
Donors: 3

Model Prediction

Prediction: Mild
Probability: 0.648
Enrichment: 0.941
Bayesian Score: -4.35
Mahalanobis Distance: 8.2
Mahalanobis Distance p-value: 0.875

Prediction: Positive if the Bayesian score is above the estimated best cutoff value from minimizing the false positive and false negative rate.
Probability: The esimated probability that the sample is in the positive category. This assumes that the Bayesian score follows a normal distribution and is different from the prediction using a cutoff.
Enrichment: An estimate of enrichment, that is, the increased likelihood (versus random) of this sample being in the category.
Bayesian Score: The standard Laplacian-modified Bayesian score.
Mahalanobis Distance: The Mahalanobis distance (MD) is the distance to the center of the training data. The larger the MD, the less trustworthy the prediction.
Mahalanobis Distance p-value: The p-value gives the fraction of training data with an MD greater than or equal to the one for the given sample, assuming normally distributed data. The smaller the p-value, the less trustworthy the prediciton. For highly non-normal X properties (e.g., fingerprints), the MD p-value is wildly inaccurate.

Structural Similar Compounds

Name	4;4'-DIAMINO-1;1'-DIANTHRIMIDE	METHANE;TRIS(4-AMINOPHENYL)-	ANTHRAQUINONE; 1-((2-HYDROXYETHYL)AMINO)-4-(METHYLAMINO)-
Structure	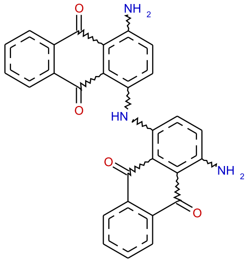	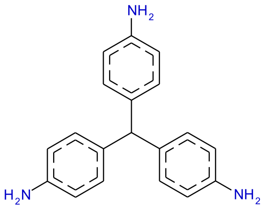	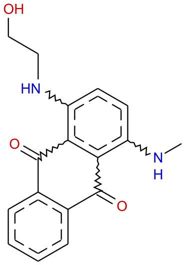
Actual Endpoint	Mild	Moderate_Severe	Mild
Predicted Endpoint	Mild	Moderate_Severe	Mild
Distance	0.778	0.784	0.787
Reference	28ZPAK-;125;72	28ZPAK-;73;72	28ZPAK 245;72

Model Applicability

Unknown features are fingerprint features in the query molecule, but not found or appearing too infrequently in the training set.

1. All properties and OPS components are within expected ranges.
2. Unknown FCFP_2 feature: 179977000: [*][c]1:[*]:[*]:n:n:1[c](:[*]):[*]
3. Unknown FCFP_2 feature: 2119857014: [*]NC(N[*])[c](:[*]):[*]

Feature Contribution

Top features for positive contribution

Fingerprint	Bit/Smiles	Feature Structure	Score	Moderate_Severe in training set
FCFP_10	-124655670	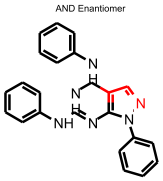 [*][c]1:[*]:[*]:n:[cH]:1	0.259	14 out of 16

FCFP_10	580453787	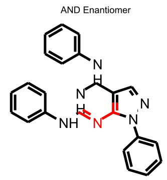 [*]C(=N[c](:[*]):[*])[*]	0.256	2 out of 2
FCFP_10	-1410049896	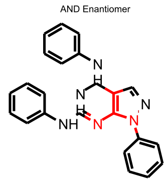 [*]n1:[*]:[*]:[c]([*]):[c]:1N=[*]	0.256	2 out of 2

Top Features for negative contribution

Fingerprint	Bit/Smiles	Feature Structure	Score	Moderate_Severe in training set
FCFP_10	4427049	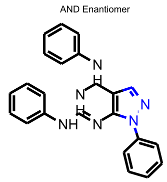 [*]n1:[*]:[*]:[cH]:n:1	-1.29	0 out of 4
FCFP_10	-1732563065	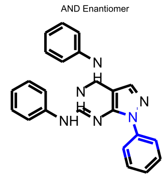 [*]:[cH]:[c](:[cH]:[*])n(:[*]):[*]	-1.09	0 out of 3
FCFP_10	1860261331	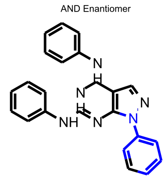 [*]:n(:[*])[c]1:[cH]:[*]:[cH]:[cH]:[cH]:1	-0.842	0 out of 2

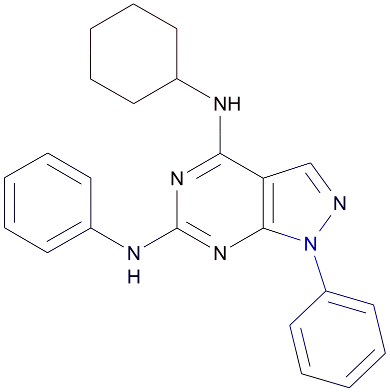

$C_{23}H_{24}N_6$
Molecular Weight: 384.47686
ALogP: 5.953
Rotatable Bonds: 5
Acceptors: 5
Donors: 2

Structural Similar Compounds

Name	ANTHRAQUINONE; 1;4-BIS(p-TOLYLAMINO)-	ANTHRAQUINONE; 1;1'-IMINODI-	DINAPHTHO(1;2;3-CD:3';2';1'-IM)PERYLENE-5;10-DIONE;16;17-DIHYDROXY
Structure	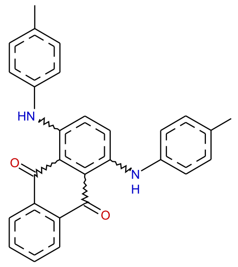	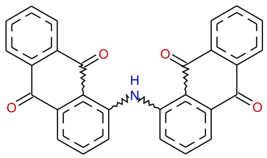	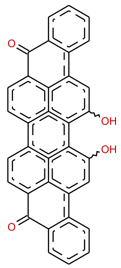
Actual Endpoint	Moderate_Severe	Mild	Mild
Predicted Endpoint	Mild	Mild	Mild
Distance	0.620	0.734	0.739
Reference	28ZPAK -;124;72	28ZPAK-;125;72	28ZPAK-;104;72

Model Prediction

Prediction: Mild
Probability: 0.668
Enrichment: 0.97
Bayesian Score: -4.05
Mahalanobis Distance: 9.18
Mahalanobis Distance p-value: 0.417

Prediction: Positive if the Bayesian score is above the estimated best cutoff value from minimizing the false positive and false negative rate.
Probability: The esimated probability that the sample is in the positive category. This assumes that the Bayesian score follows a normal distribution and is different from the prediction using a cutoff.
Enrichment: An estimate of enrichment, that is, the increased likelihood (versus random) of this sample being in the category.
Bayesian Score: The standard Laplacian-modified Bayesian score.
Mahalanobis Distance: The Mahalanobis distance (MD) is the distance to the center of the training data. The larger the MD, the less trustworthy the prediction.
Mahalanobis Distance p-value: The p-value gives the fraction of training data with an MD greater than or equal to the one for the given sample, assuming normally distributed data. The smaller the p-value, the less trustworthy the prediciton. For highly non-normal X properties (e.g., fingerprints), the MD p-value is wildly inaccurate.

Model Applicability

Unknown features are fingerprint features in the query molecule, but not found or appearing too infrequently in the training set.

1. All properties and OPS components are within expected ranges.
2. Unknown FCFP_2 feature: -1564473960: [*]n1:[*]:[*]:[c](:[*]):[c]:1:n:[*]
3. Unknown FCFP_2 feature: 179977000: [*][c]1:[*]:[*]:n:n:1[c](:[*]):[*]
4. Unknown FCFP_2 feature: -1151884458: [*]N[c](:n:[*]):[c](:[*]):[*]

Feature Contribution

Top features for positive contribution

Fingerprint	Bit/Smiles	Feature Structure	Score	Moderate_Severe in training set
FCFP_10	-124655670	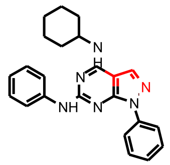 [*][c]1:[*]:[*]:n:[cH]:1	0.259	14 out of 16

FCFP_10	354117335	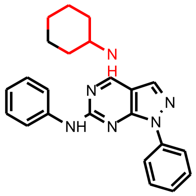 [*]NC1C[*]CCC1	0.226	16 out of 19
FCFP_10	-1043250487	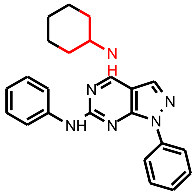 [*]CC(C[*])N[*]	0.22	62 out of 75
**Top Features for negative contribution**				
**Fingerprint**	**Bit/Smiles**	**Feature Structure**	**Score**	**Moderate_Severe in training set**
FCFP_10	4427049	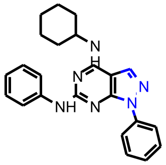 [*]n1:[*]:[*]:[cH]:n:1	-1.29	0 out of 4
FCFP_10	-1732563065	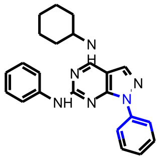 [*]:[cH]:[c](:[cH]:[*])n(:[*]):[*]	-1.09	0 out of 3
FCFP_10	1860261331	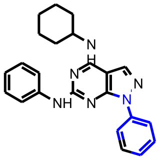 [*]:n(:[*])[c]1:[cH]:[*]:[cH]:[cH]:[cH]:1	-0.842	0 out of 2

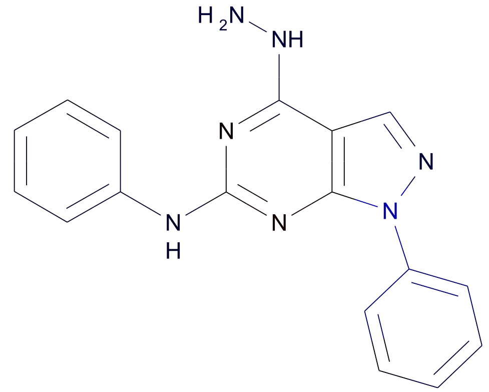

$C_{17}H_{15}N_7$
Molecular Weight: 317.3479
ALogP: 3.562
Rotatable Bonds: 4
Acceptors: 6
Donors: 3

Model Prediction

Prediction: Mild
Probability: 0.57
Enrichment: 0.827
Bayesian Score: -5.38
Mahalanobis Distance: 7.23
Mahalanobis Distance p-value: 0.995

Prediction: Positive if the Bayesian score is above the estimated best cutoff value from minimizing the false positive and false negative rate.
Probability: The esimated probability that the sample is in the positive category. This assumes that the Bayesian score follows a normal distribution and is different from the prediction using a cutoff.
Enrichment: An estimate of enrichment, that is, the increased likelihood (versus random) of this sample being in the category.
Bayesian Score: The standard Laplacian-modified Bayesian score.
Mahalanobis Distance: The Mahalanobis distance (MD) is the distance to the center of the training data. The larger the MD, the less trustworthy the prediction.
Mahalanobis Distance p-value: The p-value gives the fraction of training data with an MD greater than or equal to the one for the given sample, assuming normally distributed data. The smaller the p-value, the less trustworthy the prediciton. For highly non-normal X properties (e.g., fingerprints), the MD p-value is wildly inaccurate.

Structural Similar Compounds

Name	ANTHRAQUINONE; 1-((2-HYDROXYETHYL)AMINO)-4-(METHYLAMINO)-	ANTHRAQUINONE; 1-AMINO-4-HYDROXY-2-PHENOXY-	4;4'-DIAMINO-1;1'-DIANTHRIMIDE
Structure	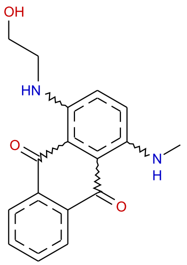	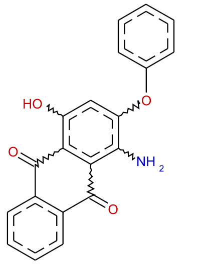	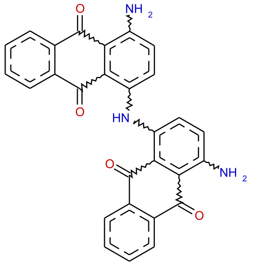
Actual Endpoint	Mild	Mild	Mild
Predicted Endpoint	Mild	Mild	Mild
Distance	0.678	0.732	0.739
Reference	28ZPAK 245;72	28ZPAK 239;72	28ZPAK-;125;72

Model Applicability

Unknown features are fingerprint features in the query molecule, but not found or appearing too infrequently in the training set.

1. All properties and OPS components are within expected ranges.
2. Unknown FCFP_2 feature: -1564473960: [*]n1:[*]:[*]:[c](:[*]):[c]:1:n:[*]
3. Unknown FCFP_2 feature: 179977000: [*][c]1:[*]:[*]:n:n:1[c](:[*]):[*]
4. Unknown FCFP_2 feature: -1151884458: [*]N[c](:n:[*]):[c](:[*]):[*]

Feature Contribution

Top features for positive contribution

Fingerprint	Bit/Smiles	Feature Structure	Score	Moderate_Severe in training set
FCFP_10	-124655670	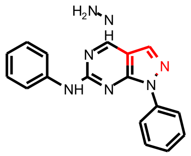 [*][c]1:[*]:[*]:n:[cH]:1	0.259	14 out of 16

FCFP_10	-656492378	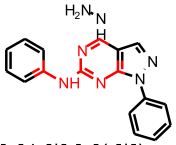 [*][c]1:[*]:[c](:[*]):n:[c](N[c](:[*]):[*]):n:1	0.186	1 out of 1
FCFP_10	1551257511	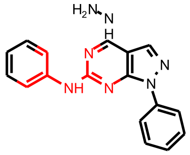 [*]:[cH]:[c](N[c](:n:[*]):n:[*]):[cH]:[*]	0.186	1 out of 1
**Top Features for negative contribution**				
**Fingerprint**	**Bit/Smiles**	**Feature Structure**	**Score**	**Moderate_Severe in training set**
FCFP_10	4427049	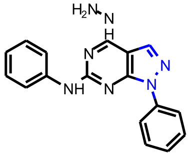 [*]n1:[*]:[*]:[cH]:n:1	-1.29	0 out of 4
FCFP_10	-1732563065	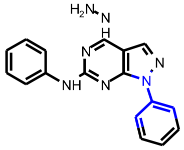 [*]:[cH]:[c](:[cH]:[*])n(:[*]):[*]	-1.09	0 out of 3
FCFP_10	1860261331	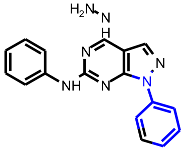 [*]:n(:[*])[c]1:[cH]:[*]:[cH]:[cH]:[cH]:1	-0.842	0 out of 2

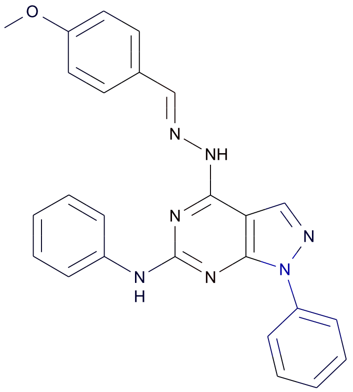

$C_{25}H_{21}N_7O$
Molecular Weight: 435.48054
ALogP: 5.889
Rotatable Bonds: 7
Acceptors: 7
Donors: 2

Model Prediction

Prediction: Mild
Probability: 0.576
Enrichment: 0.836
Bayesian Score: -5.3
Mahalanobis Distance: 7.44
Mahalanobis Distance p-value: 0.988

Prediction: Positive if the Bayesian score is above the estimated best cutoff value from minimizing the false positive and false negative rate.
Probability: The esimated probability that the sample is in the positive category. This assumes that the Bayesian score follows a normal distribution and is different from the prediction using a cutoff.
Enrichment: An estimate of enrichment, that is, the increased likelihood (versus random) of this sample being in the category.
Bayesian Score: The standard Laplacian-modified Bayesian score.
Mahalanobis Distance: The Mahalanobis distance (MD) is the distance to the center of the training data. The larger the MD, the less trustworthy the prediction.
Mahalanobis Distance p-value: The p-value gives the fraction of training data with an MD greater than or equal to the one for the given sample, assuming normally distributed data. The smaller the p-value, the less trustworthy the prediciton. For highly non-normal X properties (e.g., fingerprints), the MD p-value is wildly inaccurate.

Structural Similar Compounds

Name	ANTHRAQUINONE; 1;4-BIS(p-TOLYLAMINO)-	ANTHRAQUINONE; 1;1'-IMINODI-	4;4'-DIAMINO-1;1'-DIANTHRIMIDE
Structure	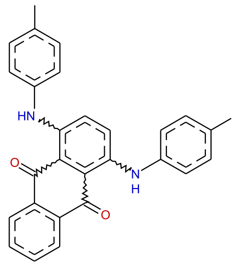	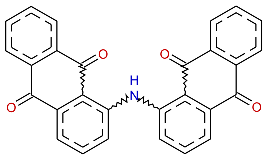	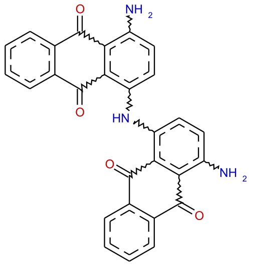
Actual Endpoint	Moderate_Severe	Mild	Mild
Predicted Endpoint	Mild	Mild	Mild
Distance	0.779	0.792	0.836
Reference	28ZPAK -;124;72	28ZPAK-;125;72	28ZPAK-;125;72

Model Applicability

Unknown features are fingerprint features in the query molecule, but not found or appearing too infrequently in the training set.

1. All properties and OPS components are within expected ranges.
2. Unknown FCFP_2 feature: -1564473960: [*]n1:[*]:[*]:[c](:[*]):[c]:1:n:[*]
3. Unknown FCFP_2 feature: 179977000: [*][c]1:[*]:[*]:n:n:1[c](:[*]):[*]
4. Unknown FCFP_2 feature: -1151884458: [*]N[c](:n:[*]):[c](:[*]):[*]
5. Unknown FCFP_2 feature: 1294285001: [*]=NN[c](:[*]):[*]
6. Unknown FCFP_2 feature: 581019816: [*]N\N=C\[*]

Feature Contribution

Top features for positive contribution

Fingerprint	Bit/Smiles	Feature Structure	Score	Moderate_Severe in training set
FCFP_10	-1034142694	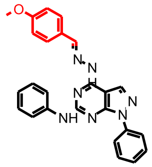 [*]=C[c]1:[cH]:[cH]:[c](OC):[cH]:[cH]:1	0.338	18 out of 19

FCFP_10	-124655670	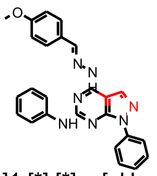 [*][c]1:[*]:[*]:n:[cH]:1	0.259	14 out of 16
FCFP_10	-2100785893	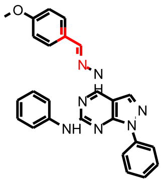 [*]\N=C\[c](:[*]):[*]	0.256	2 out of 2

Top Features for negative contribution

Fingerprint	Bit/Smiles	Feature Structure	Score	Moderate_Severe in training set
FCFP_10	4427049	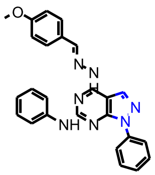 [*]n1:[*]:[*]:[cH]:n: 1	-1.29	0 out of 4
FCFP_10	-1732563065	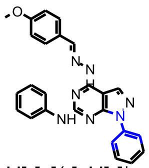 [*]:[cH]:[c](:[cH]:[*])n(:[*]):[*]	-1.09	0 out of 3
FCFP_10	1860261331	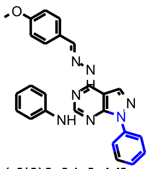 [*]:n(:[*])[c]1:[cH]: [*]:[cH]:[cH]:[cH]:1	-0.842	0 out of 2

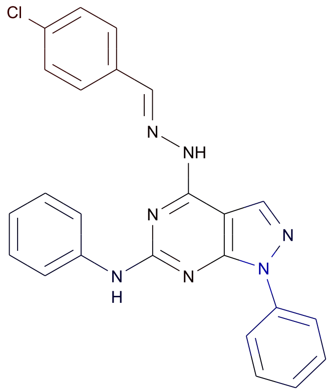

$C_{24}H_{18}ClN_7$
Molecular Weight: 439.89962
ALogP: 6.57
Rotatable Bonds: 6
Acceptors: 6
Donors: 2

Structural Similar Compounds

Name	ANTHRAQUINONE; 1;4-BIS(p-TOLYLAMINO)-	DINAPHTHO(1;2;3-CD:3';2';1'-IM)PERYLENE-5;10-DIONE;16;17-DIHYDROXY	ANTHRAQUINONE; 1;1'-IMINODI-
Structure	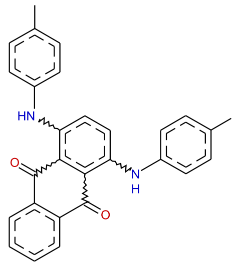	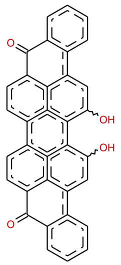	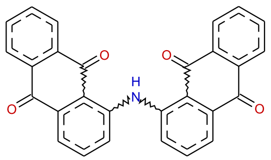
Actual Endpoint	Moderate_Severe	Mild	Mild
Predicted Endpoint	Mild	Mild	Mild
Distance	0.668	0.742	0.760
Reference	28ZPAK -;124;72	28ZPAK-;104;72	28ZPAK-;125;72

Model Prediction

Prediction: Mild
Probability: 0.709
Enrichment: 1.03
Bayesian Score: -3.36
Mahalanobis Distance: 7.4
Mahalanobis Distance p-value: 0.989

Prediction: Positive if the Bayesian score is above the estimated best cutoff value from minimizing the false positive and false negative rate.
Probability: The esimated probability that the sample is in the positive category. This assumes that the Bayesian score follows a normal distribution and is different from the prediction using a cutoff.
Enrichment: An estimate of enrichment, that is, the increased likelihood (versus random) of this sample being in the category.
Bayesian Score: The standard Laplacian-modified Bayesian score.
Mahalanobis Distance: The Mahalanobis distance (MD) is the distance to the center of the training data. The larger the MD, the less trustworthy the prediction.
Mahalanobis Distance p-value: The p-value gives the fraction of training data with an MD greater than or equal to the one for the given sample, assuming normally distributed data. The smaller the p-value, the less trustworthy the prediciton. For highly non-normal X properties (e.g., fingerprints), the MD p-value is wildly inaccurate.

Model Applicability

Unknown features are fingerprint features in the query molecule, but not found or appearing too infrequently in the training set.

1. All properties and OPS components are within expected ranges.
2. Unknown FCFP_2 feature: -1564473960: [*]n1:[*]:[*]:[c](:[*]):[c]:1:n:[*]
3. Unknown FCFP_2 feature: 179977000: [*][c]1:[*]:[*]:n:n:1[c](:[*]):[*]
4. Unknown FCFP_2 feature: -1151884458: [*]N[c](:n:[*]):[c](:[*]):[*]
5. Unknown FCFP_2 feature: 1294285001: [*]=NN[c](:[*]):[*]
6. Unknown FCFP_2 feature: 581019816: [*]N\N=C\[*]

Feature Contribution

Top features for positive contribution				
**Fingerprint**	**Bit/Smiles**	**Feature Structure**	**Score**	**Moderate_Severe in training set**

FCFP_10	-149636017	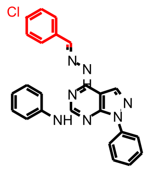 [*]=C[c]1:[cH]:[cH]:[c](Cl):[cH]:[cH]:1	0.352	7 out of 7
FCFP_10	-1508180856	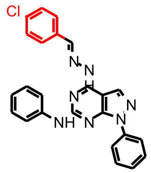 [*][c]1:[cH]:[cH]:[c] (Cl):[cH]:[cH]:1	0.329	16 out of 17
FCFP_10	-745491832	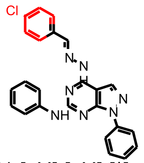 Cl[c]1:[cH]:[cH]:[*]: [cH]:[cH]:1	0.304	29 out of 32

Top Features for negative contribution

Fingerprint	Bit/Smiles	Feature Structure	Score	Moderate_Severe in training set
FCFP_10	4427049	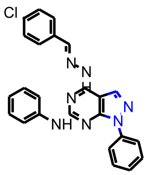 [*]n1:[*]:[*]:[cH]:n: 1	-1.29	0 out of 4
FCFP_10	-1732563065	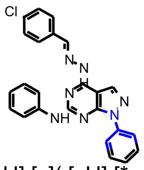 [*]:[cH]:[c](:[cH]:[*])n(:[*]):[*]	-1.09	0 out of 3

FCFP_10	1860261331	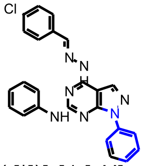<br>[*]:n(:[*])[c]1:[cH]:<br>[*]:[cH]:[cH]:[cH]:1	-0.842	0 out of 2

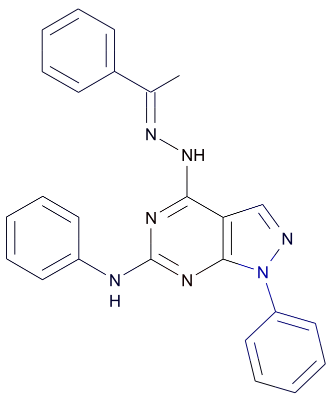

$C_{25}H_{21}N_7$
Molecular Weight: 419.48114
ALogP: 5.886
Rotatable Bonds: 6
Acceptors: 6
Donors: 2

Structural Similar Compounds

Name	ANTHRAQUINONE; 1;4-BIS(p-TOLYLAMINO)-	ANTHRAQUINONE; 1;1'-IMINODI-	DINAPHTHO(1;2;3-CD:3';2';1'-IM)PERYLENE-5;10-DIONE;16;17-DIHYDROXY
Structure	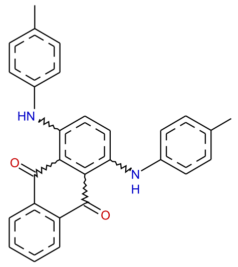	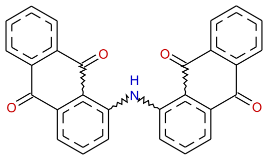	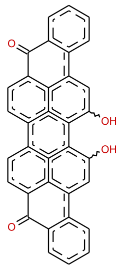
Actual Endpoint	Moderate_Severe	Mild	Mild
Predicted Endpoint	Mild	Mild	Mild
Distance	0.679	0.731	0.759
Reference	28ZPAK -;124;72	28ZPAK-;125;72	28ZPAK-;104;72

Model Prediction

Prediction: Mild
Probability: 0.567
Enrichment: 0.824
Bayesian Score: -5.41
Mahalanobis Distance: 6.8
Mahalanobis Distance p-value: 0.999

Prediction: Positive if the Bayesian score is above the estimated best cutoff value from minimizing the false positive and false negative rate.
Probability: The esimated probability that the sample is in the positive category. This assumes that the Bayesian score follows a normal distribution and is different from the prediction using a cutoff.
Enrichment: An estimate of enrichment, that is, the increased likelihood (versus random) of this sample being in the category.
Bayesian Score: The standard Laplacian-modified Bayesian score.
Mahalanobis Distance: The Mahalanobis distance (MD) is the distance to the center of the training data. The larger the MD, the less trustworthy the prediction.
Mahalanobis Distance p-value: The p-value gives the fraction of training data with an MD greater than or equal to the one for the given sample, assuming normally distributed data. The smaller the p-value, the less trustworthy the prediciton. For highly non-normal X properties (e.g., fingerprints), the MD p-value is wildly inaccurate.

Model Applicability

Unknown features are fingerprint features in the query molecule, but not found or appearing too infrequently in the training set.

1. All properties and OPS components are within expected ranges.
2. Unknown FCFP_2 feature: -1564473960: [*]n1:[*]:[*]:[c](:[*]):[c]:1:n:[*]
3. Unknown FCFP_2 feature: 179977000: [*][c]1:[*]:[*]:n:n:1[c](:[*]):[*]
4. Unknown FCFP_2 feature: -1151884458: [*]N[c](:n:[*]):[c](:[*]):[*]
5. Unknown FCFP_2 feature: 1294285001: [*]=NN[c](:[*]):[*]
6. Unknown FCFP_2 feature: 581019816: [*]N\N=C\[*]

Feature Contribution

Top features for positive contribution				
Fingerprint	Bit/Smiles	Feature Structure	Score	Moderate_Severe in training set

FCFP_10	-124655670	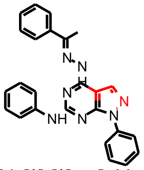 [*][c]1:[*]:[*]:n:[cH]:1	0.259	14 out of 16
FCFP_10	1551257511	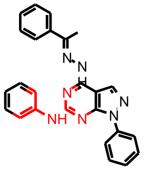 [*]:[cH]:[c](N[c](:n:[*]):n:[*]):[cH]:[*]	0.186	1 out of 1
FCFP_10	-656492378	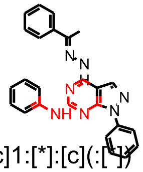 [*][c]1:[*]:[c](:[*]):n:[c](N[c](:[*]):[*]):n:1	0.186	1 out of 1

Top Features for negative contribution

Fingerprint	Bit/Smiles	Feature Structure	Score	Moderate_Severe in training set
FCFP_10	4427049	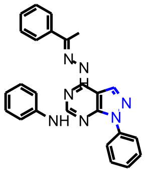 [*]n1:[*]:[*]:[cH]:n:1	-1.29	0 out of 4
FCFP_10	-1732563065	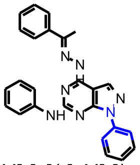 [*]:[cH]:[c](:[cH]:[*])n(:[*]):[*]	-1.09	0 out of 3

FCFP_10	1860261331	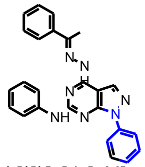 [*]:n(:[*])[c]1:[cH]:[*]:[cH]:[cH]:[cH]:1	-0.842	0 out of 2

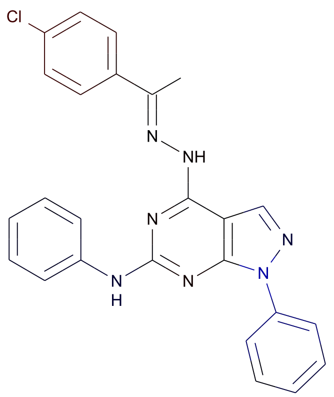

$C_{25}H_{20}ClN_7$
Molecular Weight: 453.9262
ALogP: 6.55
Rotatable Bonds: 6
Acceptors: 6
Donors: 2

Structural Similar Compounds

Name	ANTHRAQUINONE; 1;4-BIS(p-TOLYLAMINO)-	DINAPHTHO(1;2;3-CD:3';2';1'-IM)PERYLENE-5;10-DIONE;16;17-DIHYDROXY	ANTHRAQUINONE; 1;1'-IMINODI-
Structure	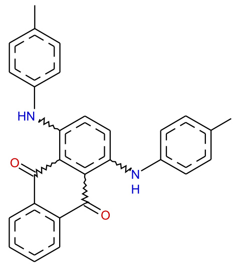	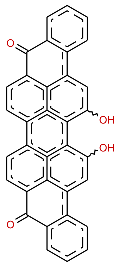	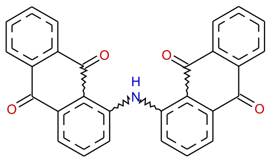
Actual Endpoint	Moderate_Severe	Mild	Mild
Predicted Endpoint	Mild	Mild	Mild
Distance	0.670	0.736	0.763
Reference	28ZPAK -;124;72	28ZPAK-;104;72	28ZPAK-;125;72

Model Prediction

Prediction: Mild
Probability: 0.689
Enrichment: 1
Bayesian Score: -3.72
Mahalanobis Distance: 7.77
Mahalanobis Distance p-value: 0.962

Prediction: Positive if the Bayesian score is above the estimated best cutoff value from minimizing the false positive and false negative rate.
Probability: The esimated probability that the sample is in the positive category. This assumes that the Bayesian score follows a normal distribution and is different from the prediction using a cutoff.
Enrichment: An estimate of enrichment, that is, the increased likelihood (versus random) of this sample being in the category.
Bayesian Score: The standard Laplacian-modified Bayesian score.
Mahalanobis Distance: The Mahalanobis distance (MD) is the distance to the center of the training data. The larger the MD, the less trustworthy the prediction.
Mahalanobis Distance p-value: The p-value gives the fraction of training data with an MD greater than or equal to the one for the given sample, assuming normally distributed data. The smaller the p-value, the less trustworthy the prediciton. For highly non-normal X properties (e.g., fingerprints), the MD p-value is wildly inaccurate.

Model Applicability

Unknown features are fingerprint features in the query molecule, but not found or appearing too infrequently in the training set.

1. All properties and OPS components are within expected ranges.
2. Unknown FCFP_2 feature: -1564473960: [*]n1:[*]:[*]:[c](:[*]):[c]:1:n:[*]
3. Unknown FCFP_2 feature: 179977000: [*][c]1:[*]:[*]:n:n:1[c](:[*]):[*]
4. Unknown FCFP_2 feature: -1151884458: [*]N[c](:n:[*]):[c](:[*]):[*]
5. Unknown FCFP_2 feature: 1294285001: [*]=NN[c](:[*]):[*]
6. Unknown FCFP_2 feature: 581019816: [*]N\N=C\[*]

Feature Contribution

Top features for positive contribution

Fingerprint	Bit/Smiles	Feature Structure	Score	Moderate_Severe in training set

FCFP_10	-149636017	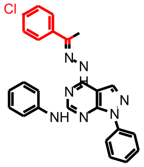[*]=C[c]1:[cH]:[cH]:[c](Cl):[cH]:[cH]:1	0.352	7 out of 7
FCFP_10	-1508180856	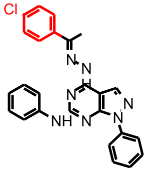[*][c]1:[cH]:[cH]:[c] (Cl):[cH]:[cH]:1	0.329	16 out of 17
FCFP_10	-745491832	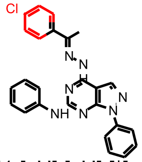Cl[c]1:[cH]:[cH]:[*]: [cH]:[cH]:1	0.304	29 out of 32
**Top Features for negative contribution**				
**Fingerprint**	**Bit/Smiles**	**Feature Structure**	**Score**	**Moderate_Severe in training set**
FCFP_10	4427049	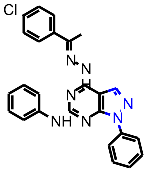[*]n1:[*]:[*]:[cH]:n: 1	-1.29	0 out of 4
FCFP_10	-1732563065	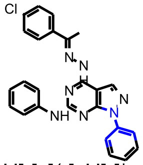[*]:[cH]:[c](:[cH]:[*])n(:[*]):[*]	-1.09	0 out of 3

FCFP_10	1860261331	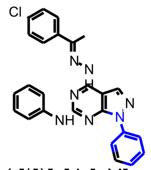 [*]:n(:[*])[c]1:[cH]:[*]:[cH]:[cH]:[cH]:1	-0.842	0 out of 2

12c

TOPKAT_Ocular_Irritancy_Mild_vs_Moderate_Severe

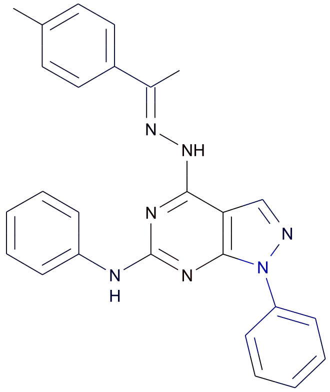

$C_{26}H_{23}N_7$
Molecular Weight: 433.50772
ALogP: 6.372
Rotatable Bonds: 6
Acceptors: 6
Donors: 2

Model Prediction

Prediction: Mild

Probability: 0.583

Enrichment: 0.846

Bayesian Score: -5.22

Mahalanobis Distance: 6.97

Mahalanobis Distance p-value: 0.998

Prediction: Positive if the Bayesian score is above the estimated best cutoff value from minimizing the false positive and false negative rate.
Probability: The esimated probability that the sample is in the positive category. This assumes that the Bayesian score follows a normal distribution and is different from the prediction using a cutoff.
Enrichment: An estimate of enrichment, that is, the increased likelihood (versus random) of this sample being in the category.
Bayesian Score: The standard Laplacian-modified Bayesian score.
Mahalanobis Distance: The Mahalanobis distance (MD) is the distance to the center of the training data. The larger the MD, the less trustworthy the prediction.
Mahalanobis Distance p-value: The p-value gives the fraction of training data with an MD greater than or equal to the one for the given sample, assuming normally distributed data. The smaller the p-value, the less trustworthy the prediciton. For highly non-normal X properties (e.g., fingerprints), the MD p-value is wildly inaccurate.

Structural Similar Compounds

Name	ANTHRAQUINONE; 1;4-BIS(p-TOLYLAMINO)-	DINAPHTHO(1;2;3-CD:3';2';1'-IM)PERYLENE-5;10-DIONE;16;17-DIHYDROXY	ANTHRAQUINONE; 1;1'-IMINODI-
Structure	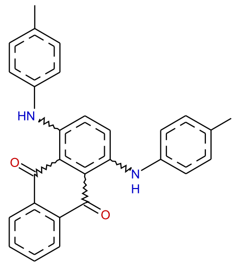	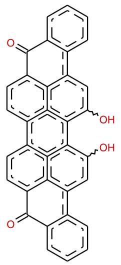	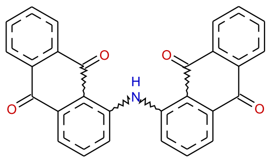
Actual Endpoint	Moderate_Severe	Mild	Mild
Predicted Endpoint	Mild	Mild	Mild
Distance	0.653	0.746	0.752
Reference	28ZPAK -;124;72	28ZPAK-;104;72	28ZPAK-;125;72

Model Applicability

Unknown features are fingerprint features in the query molecule, but not found or appearing too infrequently in the training set.

1. All properties and OPS components are within expected ranges.
2. Unknown FCFP_2 feature: -1564473960: [*]n1:[*]:[*]:[c](:[*]):[c]:1:n:[*]
3. Unknown FCFP_2 feature: 179977000: [*][c]1:[*]:[*]:n:n:1[c](:[*]):[*]
4. Unknown FCFP_2 feature: -1151884458: [*]N[c](:n:[*]):[c](:[*]):[*]
5. Unknown FCFP_2 feature: 1294285001: [*]=NN[c](:[*]):[*]
6. Unknown FCFP_2 feature: 581019816: [*]N\N=C\[*]

Feature Contribution

Top features for positive contribution				
**Fingerprint**	**Bit/Smiles**	**Feature Structure**	**Score**	**Moderate_Severe in training set**

FCFP_10	-124655670	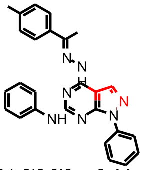 [*][c]1:[*]:[*]:n:[cH]:1	0.259	14 out of 16
FCFP_10	136120670	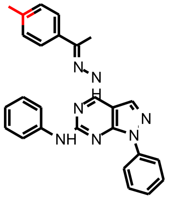 [*]:[c](:[*])C	0.206	53 out of 65
FCFP_10	-656492378	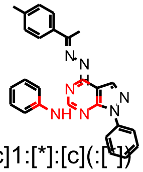 [*][c]1:[*]:[c](:[*]):n:[c](N[c](:[*]):[*]):n:1	0.186	1 out of 1

Top Features for negative contribution

Fingerprint	Bit/Smiles	Feature Structure	Score	Moderate_Severe in training set
FCFP_10	4427049	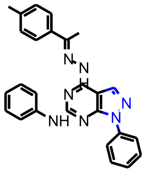 [*]n1:[*]:[*]:[cH]:n:1	-1.29	0 out of 4
FCFP_10	-1732563065	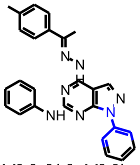 [*]:[cH]:[c](:[cH]:[*])n(:[*]):[*]	-1.09	0 out of 3

FCFP_10	1860261331	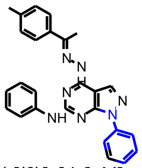 [*]:n(:[*])[c]1:[cH]:[*]:[cH]:[cH]:[cH]:1	-0.842	0 out of 2

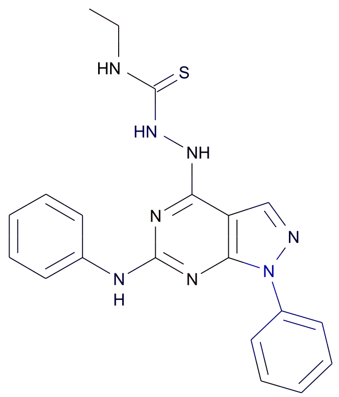

$C_{20}H_{20}N_8S$
Molecular Weight: 404.4914
ALogP: 4.99
Rotatable Bonds: 8
Acceptors: 6
Donors: 4

Structural Similar Compounds

Name	4;4'-DIAMINO-1;1'-DIANTHRIMIDE	ANTHRAQUINONE; 1;5-DIAMINO-4;8-DIHYDROXY-3-(p-METHOXYPHENYL)-	SULFIDE; BIS(DIHYDROXYPHENYL)
Structure	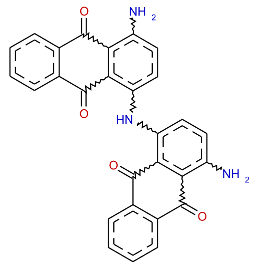	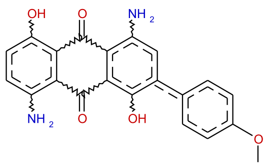	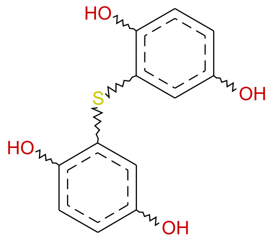
Actual Endpoint	Mild	Mild	Moderate_Severe
Predicted Endpoint	Mild	Mild	Moderate_Severe
Distance	0.797	0.825	0.922
Reference	28ZPAK-;125;72	28ZPAK 245;72	IHFCAY 6;1;67

Model Prediction

Prediction: Mild
Probability: 0.599
Enrichment: 0.869
Bayesian Score: -5.03
Mahalanobis Distance: 8.82
Mahalanobis Distance p-value: 0.609

Prediction: Positive if the Bayesian score is above the estimated best cutoff value from minimizing the false positive and false negative rate.
Probability: The esimated probability that the sample is in the positive category. This assumes that the Bayesian score follows a normal distribution and is different from the prediction using a cutoff.
Enrichment: An estimate of enrichment, that is, the increased likelihood (versus random) of this sample being in the category.
Bayesian Score: The standard Laplacian-modified Bayesian score.
Mahalanobis Distance: The Mahalanobis distance (MD) is the distance to the center of the training data. The larger the MD, the less trustworthy the prediction.
Mahalanobis Distance p-value: The p-value gives the fraction of training data with an MD greater than or equal to the one for the given sample, assuming normally distributed data. The smaller the p-value, the less trustworthy the prediciton. For highly non-normal X properties (e.g., fingerprints), the MD p-value is wildly inaccurate.

Model Applicability

Unknown features are fingerprint features in the query molecule, but not found or appearing too infrequently in the training set.

1. All properties and OPS components are within expected ranges.
2. Unknown FCFP_2 feature: -1564473960: [*]n1:[*]:[*]:[c](:[*]):[c]:1:n:[*]
3. Unknown FCFP_2 feature: 179977000: [*][c]1:[*]:[*]:n:n:1[c](:[*]):[*]
4. Unknown FCFP_2 feature: -1151884458: [*]N[c](:n:[*]):[c](:[*]):[*]
5. Unknown FCFP_2 feature: -885461129: [*]NNC(=[*])[*]

Feature Contribution

Top features for positive contribution				
Fingerprint	Bit/Smiles	Feature Structure	Score	Moderate_Severe in training set
FCFP_10	-1939253119	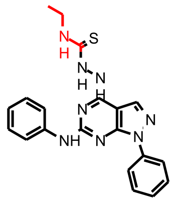 [*]C([*])NCC	0.332	5 out of 5

FCFP_10	-1272709286	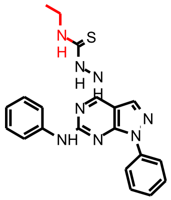 [*]NCC	0.285	234 out of 266
FCFP_10	-124655670	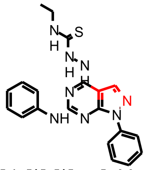 [*][c]1:[*]:[*]:n:[cH]:1	0.259	14 out of 16

Top Features for negative contribution

**Fingerprint**	**Bit/Smiles**	**Feature Structure**	**Score**	**Moderate_Severe in training set**
FCFP_10	4427049	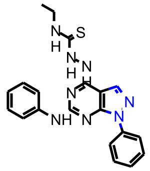 [*]n1:[*]:[*]:[cH]:n: 1	-1.29	0 out of 4
FCFP_10	-1732563065	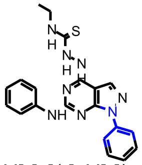 [*]:[cH]:[c](:[cH]:[*])n(:[*]):[*]	-1.09	0 out of 3
FCFP_10	1860261331	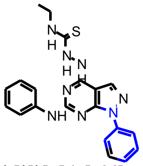 [*]:n(:[*])[c]1:[cH]: [*]:[cH]:[cH]:[cH]:1	-0.842	0 out of 2

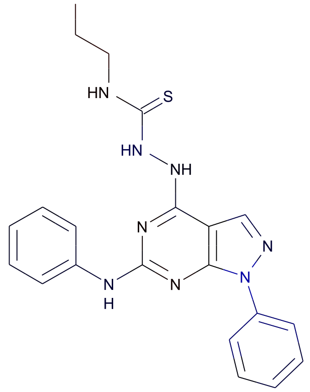

$C_{21}H_{22}N_8S$
Molecular Weight: 418.51798
ALogP: 5.514
Rotatable Bonds: 9
Acceptors: 6
Donors: 4

Structural Similar Compounds

Name	4;4'-DIAMINO-1;1'-DIANTHRIMIDE	ANTHRAQUINONE; 1;5-DIAMINO-4;8-DIHYDROXY-3-(p-METHOXYPHENYL)-	2-Naphthalenesulfonic acid; 5;6'-iminobis(1-hydroxy-
Structure	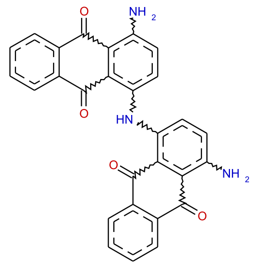	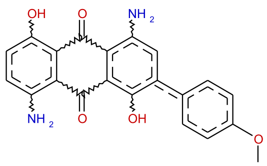	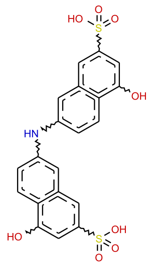
Actual Endpoint	Mild	Mild	Mild
Predicted Endpoint	Mild	Mild	Mild
Distance	0.831	0.890	0.961
Reference	28ZPAK-;125;72	28ZPAK 245;72	Prehled Prumyslove Toxikologie; Organicke Latky; Marhold; J. pp 1065;86

Model Prediction

Prediction: Mild
Probability: 0.622
Enrichment: 0.902
Bayesian Score: -4.73
Mahalanobis Distance: 9.37
Mahalanobis Distance p-value: 0.32

Prediction: Positive if the Bayesian score is above the estimated best cutoff value from minimizing the false positive and false negative rate.
Probability: The esimated probability that the sample is in the positive category. This assumes that the Bayesian score follows a normal distribution and is different from the prediction using a cutoff.
Enrichment: An estimate of enrichment, that is, the increased likelihood (versus random) of this sample being in the category.
Bayesian Score: The standard Laplacian-modified Bayesian score.
Mahalanobis Distance: The Mahalanobis distance (MD) is the distance to the center of the training data. The larger the MD, the less trustworthy the prediction.
Mahalanobis Distance p-value: The p-value gives the fraction of training data with an MD greater than or equal to the one for the given sample, assuming normally distributed data. The smaller the p-value, the less trustworthy the prediciton. For highly non-normal X properties (e.g., fingerprints), the MD p-value is wildly inaccurate.

Model Applicability

Unknown features are fingerprint features in the query molecule, but not found or appearing too infrequently in the training set.

1. All properties and OPS components are within expected ranges.
2. Unknown FCFP_2 feature: -1564473960: [*]n1:[*]:[*]:[c](:[*]):[c]:1:n:[*]
3. Unknown FCFP_2 feature: 179977000: [*][c]1:[*]:[*]:n:n:1[c](:[*]):[*]
4. Unknown FCFP_2 feature: -1151884458: [*]N[c](:n:[*]):[c](:[*]):[*]
5. Unknown FCFP_2 feature: -885461129: [*]NNC(=[*])[*]

Feature Contribution

Top features for positive contribution				
Fingerprint	Bit/Smiles	Feature Structure	Score	Moderate_Severe in training set

FCFP_10	159404153	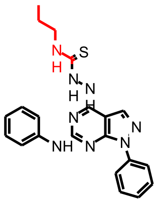 [*]C([*])NCCC	0.324	15 out of 16
FCFP_10	-1272709286	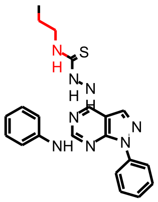 [*]NCC	0.285	234 out of 266
FCFP_10	-124655670	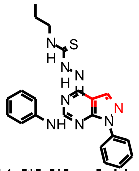 [*][c]1:[*]:[*]:n:[cH]:1	0.259	14 out of 16
**Top Features for negative contribution**				
**Fingerprint**	**Bit/Smiles**	**Feature Structure**	**Score**	**Moderate_Severe in training set**
FCFP_10	4427049	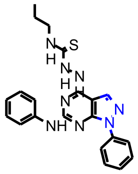 [*]n1:[*]:[*]:[cH]:n:1	-1.29	0 out of 4
FCFP_10	-1732563065	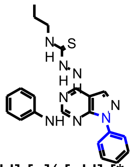 [*]:[cH]:[c](:[cH]:[*])n(:[*]):[*]	-1.09	0 out of 3

FCFP_10	1860261331	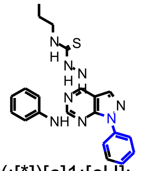 [*]:n(:[*])[c]1:[cH]:[*]:[cH]:[cH]:[cH]:1	-0.842	0 out of 2

Erlotinib

TOPKAT_Ocular_Irritancy_Mild_vs_Moderate_Severe

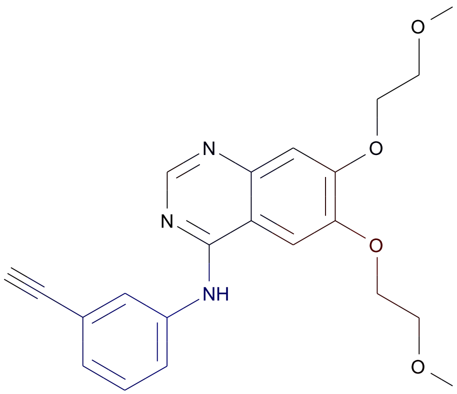

$C_{22}H_{23}N_3O_4$
Molecular Weight: 393.43572
ALogP: 4.309
Rotatable Bonds: 10
Acceptors: 7
Donors: 1

Structural Similar Compounds

Name	Benzoic acid; p-(N-butyl-2-(butylamino)acetamido)-; butyl ester;	COLCHICINE	Benzoic acid; p-(N-butyl-2-(piperidino)acetamido)-; butyl ester;
Structure	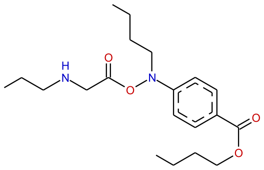	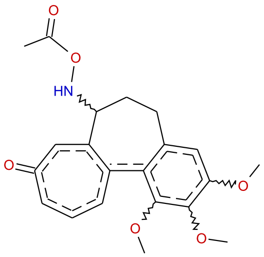	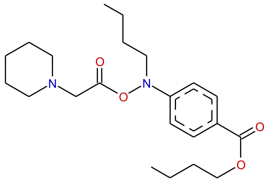
Actual Endpoint	Moderate_Severe	Moderate_Severe	Moderate_Severe
Predicted Endpoint	Moderate_Severe	Moderate_Severe	Moderate_Severe
Distance	0.648	0.707	0.750
Reference	Arzneimittel-Forschung 8;609;58	AJOPAA 31;837;48	Arzneimittel-Forschung 8;609;58

Model Prediction

Prediction: Mild
Probability: 0.766
Enrichment: 1.11
Bayesian Score: -2.1
Mahalanobis Distance: 10.4
Mahalanobis Distance p-value: 0.0368

Prediction: Positive if the Bayesian score is above the estimated best cutoff value from minimizing the false positive and false negative rate.
Probability: The esimated probability that the sample is in the positive category. This assumes that the Bayesian score follows a normal distribution and is different from the prediction using a cutoff.
Enrichment: An estimate of enrichment, that is, the increased likelihood (versus random) of this sample being in the category.
Bayesian Score: The standard Laplacian-modified Bayesian score.
Mahalanobis Distance: The Mahalanobis distance (MD) is the distance to the center of the training data. The larger the MD, the less trustworthy the prediction.
Mahalanobis Distance p-value: The p-value gives the fraction of training data with an MD greater than or equal to the one for the given sample, assuming normally distributed data. The smaller the p-value, the less trustworthy the prediciton. For highly non-normal X properties (e.g., fingerprints), the MD p-value is wildly inaccurate.

Model Applicability

Unknown features are fingerprint features in the query molecule, but not found or appearing too infrequently in the training set.

1. All properties and OPS components are within expected ranges.
2. Unknown FCFP_2 feature: -124685461: [*]:n:c:n:[*]
3. Unknown FCFP_2 feature: -1151884458: [*]N[c](:n:[*]):[c](:[*]):[*]
4. Unknown FCFP_2 feature: 902193919: [*]:[c](:[*])C#C

Feature Contribution

Top features for positive contribution

Fingerprint	Bit/Smiles	Feature Structure	Score	Moderate_Severe in training set
FCFP_10	365650923	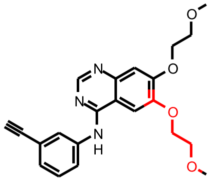 [*]OCCO[c](:[*]):[*]	0.386	17 out of 17

FCFP_10	-1059904848	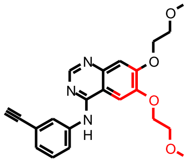<br>[*][c](:[*]):[c](OCCO<br>C):[cH]:[*]	0.386	17 out of 17
FCFP_10	-1716224640	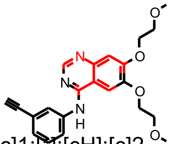<br>[*][c]1:[*]:[cH]:[c]2<br>:[c]([*]):[*]:[cH]:n<br>:[c]:2:[cH]:1	0.294	3 out of 3

Top Features for negative contribution

Fingerprint	Bit/Smiles	Feature Structure	Score	Moderate_Severe in training set
FCFP_10	-1699003333	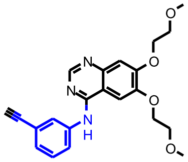<br>[*]N[c]1:[cH]:[cH]:[c<br>H]:[c](:[cH]:1)C#[*]	-1.09	2 out of 12
FCFP_10	1679603620	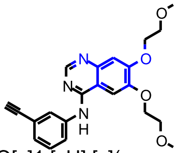<br>[*]O[c]1:[cH]:[c](:n:<br>[*]):[c](:[*]):[*]:[<br>c]:1[*]	-0.507	0 out of 1
FCFP_10	341504799	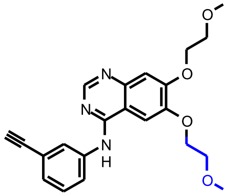<br>[*]CCOC	-0.425	7 out of 17

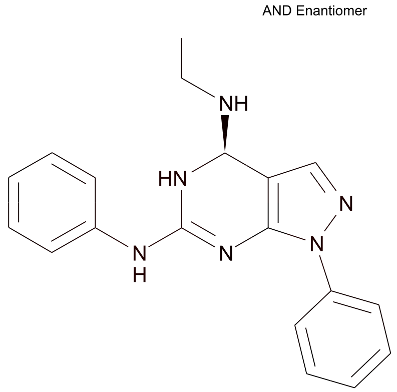

$C_{19}H_{20}N_6$
Molecular Weight: 332.4023
ALogP: 3.039
Rotatable Bonds: 5
Acceptors: 5
Donors: 3

Structural Similar Compounds

Name	ANTHRAQUINONE; 1-((2-HYDROXYETHYL)AMINO)-4-(METHYLAMINO)-	Disperse Black 9	METHANE;TRIS(4-AMINOPHENYL)-
Structure	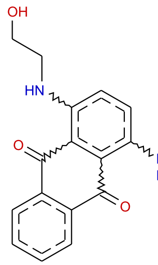	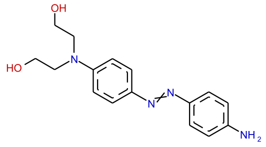	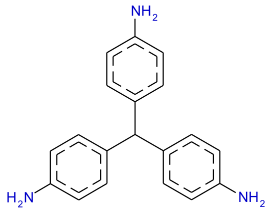
Actual Endpoint	Irritant	Non-Irritant	Irritant
Predicted Endpoint	Irritant	Non-Irritant	Irritant
Distance	0.607	0.646	0.680
Reference	28ZPAK 245;72	J. Am. Coll. Toxicol. 5(3):205;1986	28ZPAK-;73;72

Model Prediction

Prediction: Irritant
Probability: 1
Enrichment: 1.18
Bayesian Score: 1.86
Mahalanobis Distance: 6.98
Mahalanobis Distance p-value: 0.998

Prediction: Positive if the Bayesian score is above the estimated best cutoff value from minimizing the false positive and false negative rate.
Probability: The esimated probability that the sample is in the positive category. This assumes that the Bayesian score follows a normal distribution and is different from the prediction using a cutoff.
Enrichment: An estimate of enrichment, that is, the increased likelihood (versus random) of this sample being in the category.
Bayesian Score: The standard Laplacian-modified Bayesian score.
Mahalanobis Distance: The Mahalanobis distance (MD) is the distance to the center of the training data. The larger the MD, the less trustworthy the prediction.
Mahalanobis Distance p-value: The p-value gives the fraction of training data with an MD greater than or equal to the one for the given sample, assuming normally distributed data. The smaller the p-value, the less trustworthy the prediciton. For highly non-normal X properties (e.g., fingerprints), the MD p-value is wildly inaccurate.

Model Applicability

Unknown features are fingerprint features in the query molecule, but not found or appearing too infrequently in the training set.

1. All properties and OPS components are within expected ranges.
2. Unknown FCFP_2 feature: 179977000: [*][c]1:[*]:[*]:n:n:1[c](:[*]):[*]
3. Unknown FCFP_2 feature: 2119857014: [*]NC(N[*])[c](:[*]):[*]

Feature Contribution

Top features for positive contribution

Fingerprint	Bit/Smiles	Feature Structure	Score	Irritant in training set
FCFP_12	-124655670	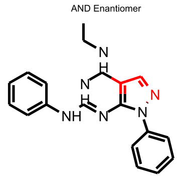 [*][c]1:[*]:[*]:n:[cH]:1	0.2	16 out of 16

FCFP_12	17	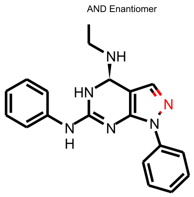 [*]:n:[*]	0.189	48 out of 49
FCFP_12	-885550502	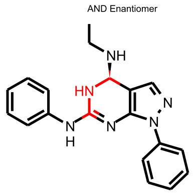 [*]C([*])NC(=[*])[*]	0.18	64 out of 66
**Top Features for negative contribution**				
**Fingerprint**	**Bit/Smiles**	**Feature Structure**	**Score**	**Irritant in training set**
FCFP_12	580453787	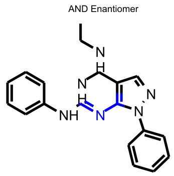 [*]C(=N[c](:[*]):[*])[*]	-0.132	2 out of 3
FCFP_12	0	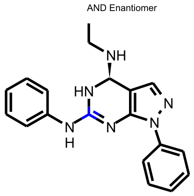 [*]C(=[*])[*]	0	1184 out of 1397
FCFP_12	-792685140	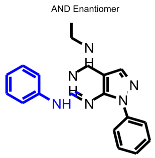 [*]C(=[*])N[c]1:[cH]:[cH]:[cH]:[cH]:[cH]:1	0	5 out of 6

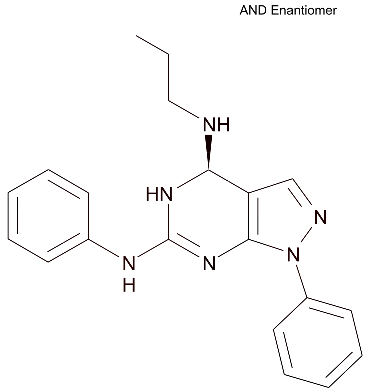

$C_{20}H_{22}N_6$
Molecular Weight: 346.42888
ALogP: 3.562
Rotatable Bonds: 6
Acceptors: 5
Donors: 3

Structural Similar Compounds

Name	ANTHRAQUINONE; 1-((2-HYDROXYETHYL)AMINO)-4-(METHYLAMINO)-	Disperse Black 9	METHANE;TRIS(4-AMINOPHENYL)-
Structure	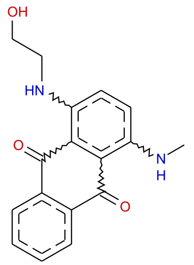	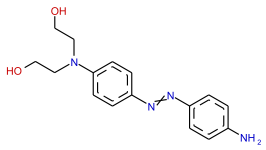	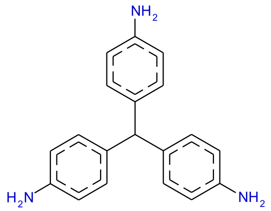
Actual Endpoint	Irritant	Non-Irritant	Irritant
Predicted Endpoint	Irritant	Non-Irritant	Irritant
Distance	0.664	0.676	0.711
Reference	28ZPAK 245;72	J. Am. Coll. Toxicol. 5(3):205;1986	28ZPAK-;73;72

Model Prediction

Prediction: Irritant
Probability: 1
Enrichment: 1.18
Bayesian Score: 1.9
Mahalanobis Distance: 7.62
Mahalanobis Distance p-value: 0.977

Prediction: Positive if the Bayesian score is above the estimated best cutoff value from minimizing the false positive and false negative rate.
Probability: The esimated probability that the sample is in the positive category. This assumes that the Bayesian score follows a normal distribution and is different from the prediction using a cutoff.
Enrichment: An estimate of enrichment, that is, the increased likelihood (versus random) of this sample being in the category.
Bayesian Score: The standard Laplacian-modified Bayesian score.
Mahalanobis Distance: The Mahalanobis distance (MD) is the distance to the center of the training data. The larger the MD, the less trustworthy the prediction.
Mahalanobis Distance p-value: The p-value gives the fraction of training data with an MD greater than or equal to the one for the given sample, assuming normally distributed data. The smaller the p-value, the less trustworthy the prediciton. For highly non-normal X properties (e.g., fingerprints), the MD p-value is wildly inaccurate.

Model Applicability

Unknown features are fingerprint features in the query molecule, but not found or appearing too infrequently in the training set.

1. All properties and OPS components are within expected ranges.
2. Unknown FCFP_2 feature: 179977000: [*][c]1:[*]:[*]:n:n:1[c](:[*]):[*]
3. Unknown FCFP_2 feature: 2119857014: [*]NC(N[*])[c](:[*]):[*]

Feature Contribution

Top features for positive contribution

Fingerprint	Bit/Smiles	Feature Structure	Score	Irritant in training set
FCFP_12	-124655670	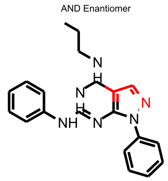 [*][c]1:[*]:[*]:n:[cH]:1	0.2	16 out of 16

FCFP_12	17	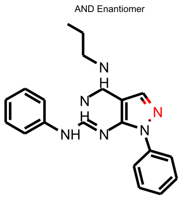<br>[*]:n:[*]	0.189	48 out of 49
FCFP_12	-885550502	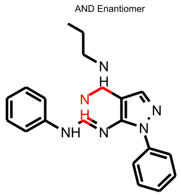<br>[*]C([*])NC(=[*])[*]	0.18	64 out of 66
**Top Features for negative contribution**				
**Fingerprint**	**Bit/Smiles**	**Feature Structure**	**Score**	**Irritant in training set**
FCFP_12	580453787	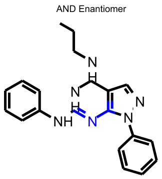<br>[*]C(=N[c](:[*]):[*])<br>[*]	-0.132	2 out of 3
FCFP_12	203677720	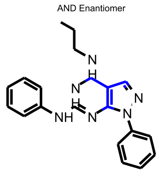<br>[*]C([*])[c]1:[cH]:[*<br>]:[*]:[c]:1[*]	0	319 out of 382
FCFP_12	0	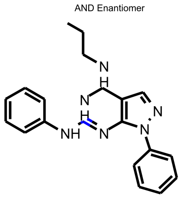<br>[*]C(=[*])[*]	0	1184 out of 1397

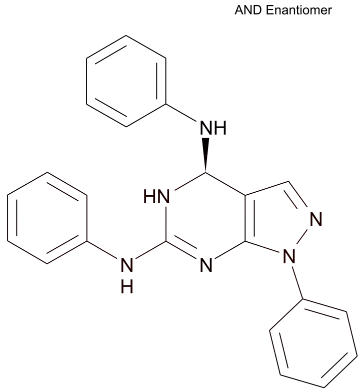

$C_{23}H_{20}N_6$
Molecular Weight: 380.4451
ALogP: 4.544
Rotatable Bonds: 5
Acceptors: 5
Donors: 3

Structural Similar Compounds

Name	ANTHRAQUINONE; 1-((2-HYDROXYETHYL)AMINO)-4-(METHYLAMINO)-	Disperse Black 9	METHANE;TRIS(4-AMINOPHENYL)-
Structure	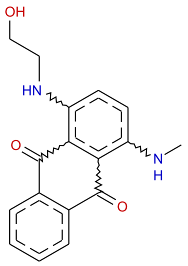	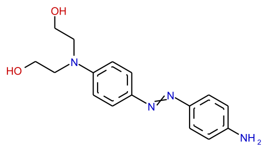	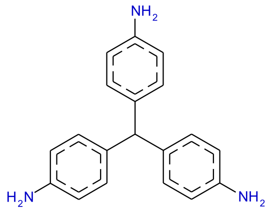
Actual Endpoint	Irritant	Non-Irritant	Irritant
Predicted Endpoint	Irritant	Non-Irritant	Irritant
Distance	0.759	0.761	0.767
Reference	28ZPAK 245;72	J. Am. Coll. Toxicol. 5(3):205;1986	28ZPAK-;73;72

Model Prediction

Prediction: Irritant
Probability: 1
Enrichment: 1.18
Bayesian Score: 1.57
Mahalanobis Distance: 6.09
Mahalanobis Distance p-value: 1

Prediction: Positive if the Bayesian score is above the estimated best cutoff value from minimizing the false positive and false negative rate.
Probability: The esimated probability that the sample is in the positive category. This assumes that the Bayesian score follows a normal distribution and is different from the prediction using a cutoff.
Enrichment: An estimate of enrichment, that is, the increased likelihood (versus random) of this sample being in the category.
Bayesian Score: The standard Laplacian-modified Bayesian score.
Mahalanobis Distance: The Mahalanobis distance (MD) is the distance to the center of the training data. The larger the MD, the less trustworthy the prediction.
Mahalanobis Distance p-value: The p-value gives the fraction of training data with an MD greater than or equal to the one for the given sample, assuming normally distributed data. The smaller the p-value, the less trustworthy the prediciton. For highly non-normal X properties (e.g., fingerprints), the MD p-value is wildly inaccurate.

Model Applicability

Unknown features are fingerprint features in the query molecule, but not found or appearing too infrequently in the training set.

1. All properties and OPS components are within expected ranges.
2. Unknown FCFP_2 feature: 179977000: [*][c]1:[*]:[*]:n:n:1[c](:[*]):[*]
3. Unknown FCFP_2 feature: 2119857014: [*]NC(N[*])[c](:[*]):[*]

Feature Contribution

Top features for positive contribution

Fingerprint	Bit/Smiles	Feature Structure	Score	Irritant in training set
FCFP_12	-124655670	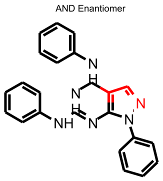 [*][c]1:[*]:[*]:n:[cH]:1	0.2	16 out of 16

FCFP_12	17	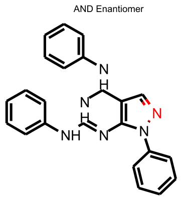 [*]:n:[*]	0.189	48 out of 49
FCFP_12	-885550502	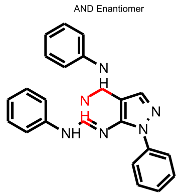 [*]C([*])NC(=[*])[*]	0.18	64 out of 66

Top Features for negative contribution

Fingerprint	Bit/Smiles	Feature Structure	Score	Irritant in training set
FCFP_12	580453787	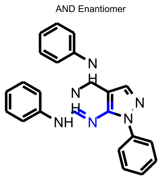 [*]C(=N[c](:[*]):[*]) [*]	-0.132	2 out of 3
FCFP_12	0	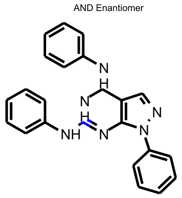 [*]C(=[*])[*]	0	1184 out of 1397
FCFP_12	1	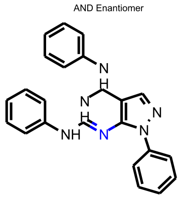 [*]N=[*]	0	872 out of 1051

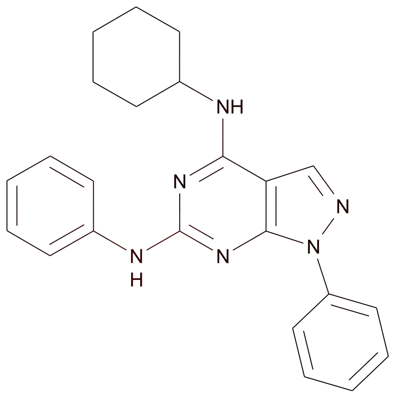

$C_{23}H_{24}N_6$
Molecular Weight: 384.47686
ALogP: 5.953
Rotatable Bonds: 5
Acceptors: 5
Donors: 2

Structural Similar Compounds

Name	ANTHRAQUINONE; 1;4-BIS(p-TOLYLAMINO)-	BENZANILIDE;2';2'''-DITHIOBIS-	DINAPHTHO(1;2;3-CD:3';2';1'-IM)PERYLENE-5;10-DIONE;16;17-DIHYDROXY
Structure	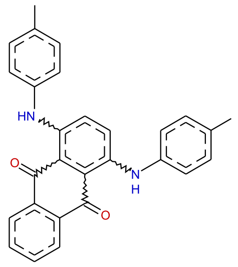	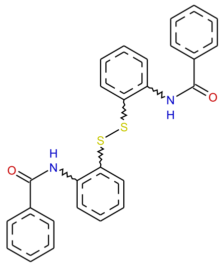	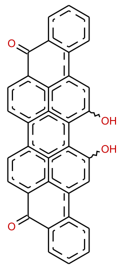
Actual Endpoint	Irritant	Non-Irritant	Irritant
Predicted Endpoint	Non-Irritant	Non-Irritant	Irritant
Distance	0.612	0.647	0.723
Reference	28ZPAK -;124;72	28ZPAK-;173;72	28ZPAK-;104;72

Model Prediction

Prediction: Irritant
Probability: 1
Enrichment: 1.18
Bayesian Score: 1.77
Mahalanobis Distance: 8.91
Mahalanobis Distance p-value: 0.559

Prediction: Positive if the Bayesian score is above the estimated best cutoff value from minimizing the false positive and false negative rate.
Probability: The esimated probability that the sample is in the positive category. This assumes that the Bayesian score follows a normal distribution and is different from the prediction using a cutoff.
Enrichment: An estimate of enrichment, that is, the increased likelihood (versus random) of this sample being in the category.
Bayesian Score: The standard Laplacian-modified Bayesian score.
Mahalanobis Distance: The Mahalanobis distance (MD) is the distance to the center of the training data. The larger the MD, the less trustworthy the prediction.
Mahalanobis Distance p-value: The p-value gives the fraction of training data with an MD greater than or equal to the one for the given sample, assuming normally distributed data. The smaller the p-value, the less trustworthy the prediciton. For highly non-normal X properties (e.g., fingerprints), the MD p-value is wildly inaccurate.

Model Applicability

Unknown features are fingerprint features in the query molecule, but not found or appearing too infrequently in the training set.

1. All properties and OPS components are within expected ranges.
2. Unknown FCFP_2 feature: -1564473960: [*]n1:[*]:[*]:[c](:[*]):[c]:1:n:[*]
3. Unknown FCFP_2 feature: 179977000: [*][c]1:[*]:[*]:n:n:1[c](:[*]):[*]
4. Unknown FCFP_2 feature: -1151884458: [*]N[c](:n:[*]):[c](:[*]):[*]

Feature Contribution

Top features for positive contribution

Fingerprint	Bit/Smiles	Feature Structure	Score	Irritant in training set
FCFP_12	1747237384	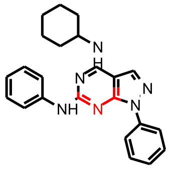 [*][c](:[*]):n:[c](:[*]):[*]	0.208	44 out of 44

FCFP_12	-124655670	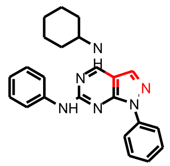 [*][c]1:[*]:[*]:n:[cH]:1	0.2	16 out of 16
FCFP_12	-1151914249	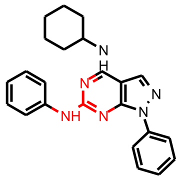 [*]N[c](:n:[*]):n:[*]	0.19	9 out of 9

Top Features for negative contribution

Fingerprint	Bit/Smiles	Feature Structure	Score	Irritant in training set
FCFP_12	1175638033	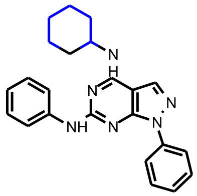 [*]C1[*]CCCC1	-0.133	207 out of 293
FCFP_12	-1525101452	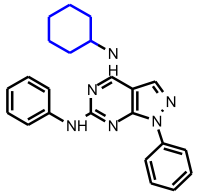 [*]C1CCCCC1	-0.127	108 out of 152
FCFP_12	-773983804	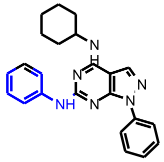 [*]N[c]1:[cH]:[*]:[cH]:[cH]:[cH]:1	0	102 out of 121

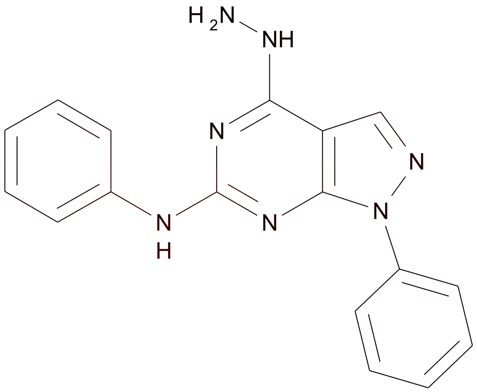

$C_{17}H_{15}N_7$
Molecular Weight: 317.3479
ALogP: 3.562
Rotatable Bonds: 4
Acceptors: 6
Donors: 3

Structural Similar Compounds

Name	Disperse Black 9	ANTHRAQUINONE; 1-((2-HYDROXYETHYL)AMINO)-4-(METHYLAMINO)-	4;4'-DIAMINO-1;1'-DIANTHRIMIDE
Structure	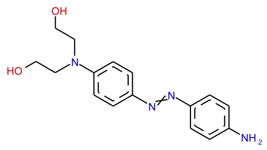	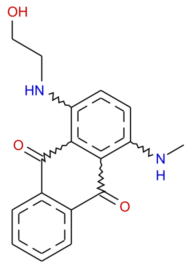	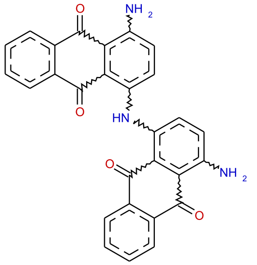
Actual Endpoint	Non-Irritant	Irritant	Irritant
Predicted Endpoint	Non-Irritant	Irritant	Irritant
Distance	0.604	0.659	0.726
Reference	J. Am. Coll. Toxicol. 5(3):205;1986	28ZPAK 245;72	28ZPAK-;125;72

Model Prediction

Prediction: Irritant
Probability: 1
Enrichment: 1.18
Bayesian Score: 2.27
Mahalanobis Distance: 6.44
Mahalanobis Distance p-value: 1

Prediction: Positive if the Bayesian score is above the estimated best cutoff value from minimizing the false positive and false negative rate.
Probability: The esimated probability that the sample is in the positive category. This assumes that the Bayesian score follows a normal distribution and is different from the prediction using a cutoff.
Enrichment: An estimate of enrichment, that is, the increased likelihood (versus random) of this sample being in the category.
Bayesian Score: The standard Laplacian-modified Bayesian score.
Mahalanobis Distance: The Mahalanobis distance (MD) is the distance to the center of the training data. The larger the MD, the less trustworthy the prediction.
Mahalanobis Distance p-value: The p-value gives the fraction of training data with an MD greater than or equal to the one for the given sample, assuming normally distributed data. The smaller the p-value, the less trustworthy the prediciton. For highly non-normal X properties (e.g., fingerprints), the MD p-value is wildly inaccurate.

Model Applicability

Unknown features are fingerprint features in the query molecule, but not found or appearing too infrequently in the training set.

1. All properties and OPS components are within expected ranges.
2. Unknown FCFP_2 feature: -1564473960: [*]n1:[*]:[*]:[c](:[*]):[c]:1:n:[*]
3. Unknown FCFP_2 feature: 179977000: [*][c]1:[*]:[*]:n:n:1[c](:[*]):[*]
4. Unknown FCFP_2 feature: -1151884458: [*]N[c](:n:[*]):[c](:[*]):[*]

Feature Contribution

Top features for positive contribution

Fingerprint	Bit/Smiles	Feature Structure	Score	Irritant in training set
FCFP_12	1747237384	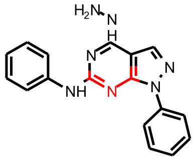 [*][c](:[*]):n:[c](:[*]):[*]	0.208	44 out of 44

FCFP_12	-124655670	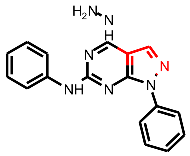 [*][c]1:[*]:[*]:n:[cH]:1	0.2	16 out of 16
FCFP_12	-1151914249	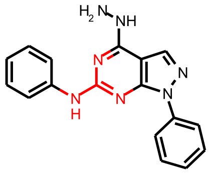 [*]N[c](:n:[*]):n:[*]	0.19	9 out of 9

Top Features for negative contribution

Fingerprint	Bit/Smiles	Feature Structure	Score	Irritant in training set
FCFP_12	307419094	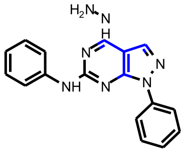 [*][c](:[*]):[c]1:[cH]:[*]:[*]:[c]:1:[*]	0	43 out of 52
FCFP_12	-2093839777	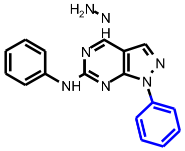 [*][c]1:[cH]:[cH]:[cH]:[cH]:[cH]:1	0	94 out of 121
FCFP_12	-773983804	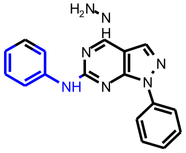 [*]N[c]1:[cH]:[*]:[cH]:[cH]:[cH]:1	0	102 out of 121

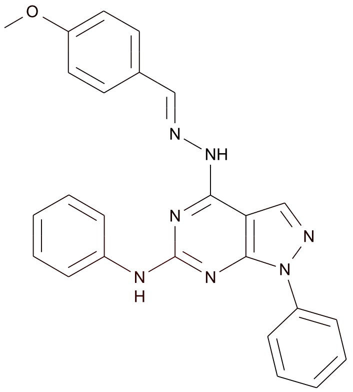

$C_{25}H_{21}N_7O$
Molecular Weight: 435.48054
ALogP: 5.889
Rotatable Bonds: 7
Acceptors: 7
Donors: 2

Model Prediction

Prediction: Irritant
Probability: 1
Enrichment: 1.18
Bayesian Score: 2.08
Mahalanobis Distance: 7.04
Mahalanobis Distance p-value: 0.998

Prediction: Positive if the Bayesian score is above the estimated best cutoff value from minimizing the false positive and false negative rate.
Probability: The esimated probability that the sample is in the positive category. This assumes that the Bayesian score follows a normal distribution and is different from the prediction using a cutoff.
Enrichment: An estimate of enrichment, that is, the increased likelihood (versus random) of this sample being in the category.
Bayesian Score: The standard Laplacian-modified Bayesian score.
Mahalanobis Distance: The Mahalanobis distance (MD) is the distance to the center of the training data. The larger the MD, the less trustworthy the prediction.
Mahalanobis Distance p-value: The p-value gives the fraction of training data with an MD greater than or equal to the one for the given sample, assuming normally distributed data. The smaller the p-value, the less trustworthy the prediciton. For highly non-normal X properties (e.g., fingerprints), the MD p-value is wildly inaccurate.

Structural Similar Compounds

Name	BENZANILIDE;2';2'''-DITHIOBIS-	ANTHRAQUINONE; 1;4-BIS(p-TOLYLAMINO)-	ANTHRAQUINONE; 1;1'-IMINODI-
Structure	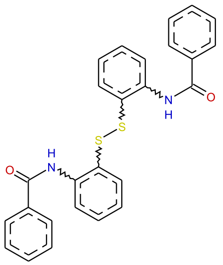	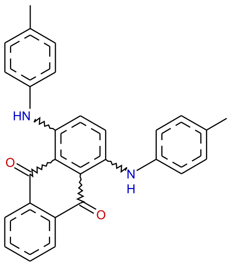	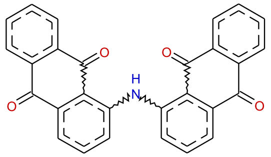
Actual Endpoint	Non-Irritant	Irritant	Irritant
Predicted Endpoint	Non-Irritant	Non-Irritant	Irritant
Distance	0.731	0.769	0.786
Reference	28ZPAK-;173;72	28ZPAK -;124;72	28ZPAK-;125;72

Model Applicability

Unknown features are fingerprint features in the query molecule, but not found or appearing too infrequently in the training set.

1. All properties and OPS components are within expected ranges.
2. Unknown FCFP_2 feature: -1564473960: [*]n1:[*]:[*]:[c](:[*]):[c]:1:n:[*]
3. Unknown FCFP_2 feature: 179977000: [*][c]1:[*]:[*]:n:n:1[c](:[*]):[*]
4. Unknown FCFP_2 feature: -1151884458: [*]N[c](:n:[*]):[c](:[*]):[*]
5. Unknown FCFP_2 feature: 1294285001: [*]=NN[c](:[*]):[*]
6. Unknown FCFP_2 feature: 581019816: [*]N\N=C\[*]

Feature Contribution

Top features for positive contribution				
Fingerprint	Bit/Smiles	Feature Structure	Score	Irritant in training set
FCFP_12	1747237384	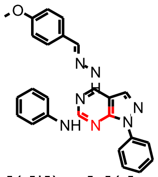 [*][c](:[*]):n:[c](:[*]):[*]	0.208	44 out of 44

FCFP_12	-124655670	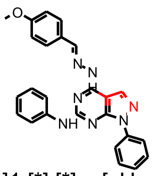 [*][c]1:[*]:[*]:n:[cH]:1	0.2	16 out of 16
FCFP_12	-1151914249	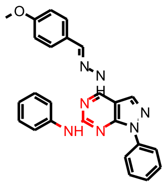 [*]N[c](:n:[*]):n:[*]	0.19	9 out of 9
**Top Features for negative contribution**				
**Fingerprint**	**Bit/Smiles**	**Feature Structure**	**Score**	**Irritant in training set**
FCFP_12	-1034142694	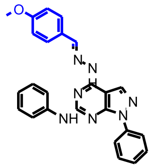 [*]=C[c]1:[cH]:[cH]:[c](OC):[cH]:[cH]:1	-0.0579	19 out of 25
FCFP_12	136627117	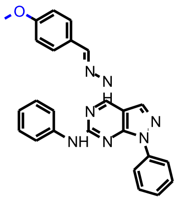 [*]OC	0	96 out of 113
FCFP_12	991735244	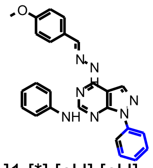 [*][c]1:[*]:[cH]:[cH]:[cH]:[cH]:1	0	237 out of 291

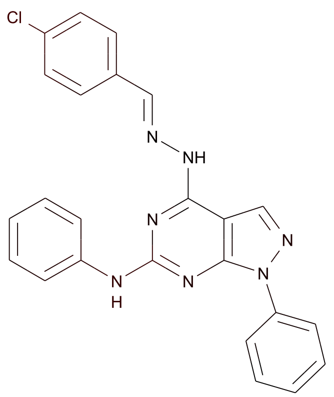

$C_{24}H_{18}ClN_7$
Molecular Weight: 439.89962
ALogP: 6.57
Rotatable Bonds: 6
Acceptors: 6
Donors: 2

Structural Similar Compounds

Name	BENZANILIDE;2';2'''-DITHIOBIS-	ANTHRAQUINONE; 1;4-BIS(p-TOLYLAMINO)-	DINAPHTHO(1;2;3-CD:3';2';1'-IM)PERYLENE-5;10-DIONE;16;17-DIHYDROXY
Structure	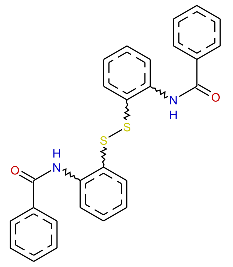	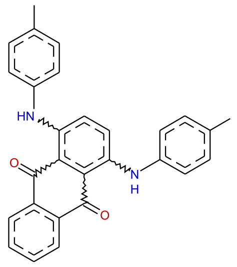	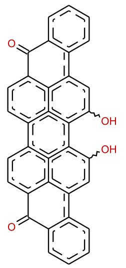
Actual Endpoint	Non-Irritant	Irritant	Irritant
Predicted Endpoint	Non-Irritant	Non-Irritant	Irritant
Distance	0.663	0.664	0.728
Reference	28ZPAK-;173;72	28ZPAK -;124;72	28ZPAK-;104;72

Model Prediction

Prediction: Irritant
Probability: 1
Enrichment: 1.18
Bayesian Score: 2.95
Mahalanobis Distance: 6
Mahalanobis Distance p-value: 1

Prediction: Positive if the Bayesian score is above the estimated best cutoff value from minimizing the false positive and false negative rate.
Probability: The esimated probability that the sample is in the positive category. This assumes that the Bayesian score follows a normal distribution and is different from the prediction using a cutoff.
Enrichment: An estimate of enrichment, that is, the increased likelihood (versus random) of this sample being in the category.
Bayesian Score: The standard Laplacian-modified Bayesian score.
Mahalanobis Distance: The Mahalanobis distance (MD) is the distance to the center of the training data. The larger the MD, the less trustworthy the prediction.
Mahalanobis Distance p-value: The p-value gives the fraction of training data with an MD greater than or equal to the one for the given sample, assuming normally distributed data. The smaller the p-value, the less trustworthy the prediciton. For highly non-normal X properties (e.g., fingerprints), the MD p-value is wildly inaccurate.

Model Applicability

Unknown features are fingerprint features in the query molecule, but not found or appearing too infrequently in the training set.

1. All properties and OPS components are within expected ranges.
2. Unknown FCFP_2 feature: -1564473960: [*]n1:[*]:[*]:[c](:[*]):[c]:1:n:[*]
3. Unknown FCFP_2 feature: 179977000: [*][c]1:[*]:[*]:n:n:1[c](:[*]):[*]
4. Unknown FCFP_2 feature: -1151884458: [*]N[c](:n:[*]):[c](:[*]):[*]
5. Unknown FCFP_2 feature: 1294285001: [*]=NN[c](:[*]):[*]
6. Unknown FCFP_2 feature: 581019816: [*]N\N=C\[*]

Feature Contribution

Top features for positive contribution				
Fingerprint	Bit/Smiles	Feature Structure	Score	Irritant in training set

FCFP_12	1747237384	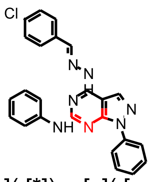 [*][c](:[*]):n:[c](:[*]):[*]	0.208	44 out of 44
FCFP_12	-1508180856	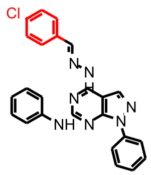 [*][c]1:[cH]:[cH]:[c] (Cl):[cH]:[cH]:1	0.2	17 out of 17
FCFP_12	-124655670	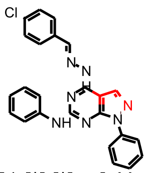 [*][c]1:[*]:[*]:n:[cH]:1	0.2	16 out of 16

Top Features for negative contribution

Fingerprint	Bit/Smiles	Feature Structure	Score	Irritant in training set
FCFP_12	-453677277	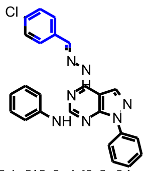 [*][c]1:[*]:[cH]:[c](C=[*]):[cH]:[cH]:1	0	264 out of 323
FCFP_12	-773983804	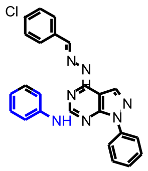 [*]N[c]1:[cH]:[*]:[cH]:[cH]:[cH]:1	0	102 out of 121

FCFP_12	-2093839777	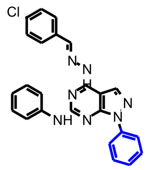<br>[*][c]1:[cH]:[cH]:[cH]:[cH]:1	0	94 out of 121

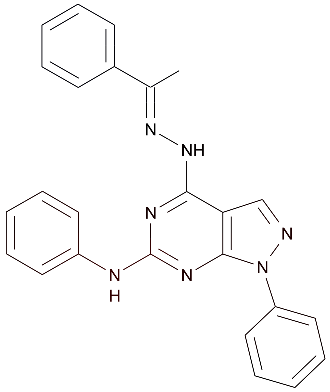

$C_{25}H_{21}N_7$
Molecular Weight: 419.48114
ALogP: 5.886
Rotatable Bonds: 6
Acceptors: 6
Donors: 2

Model Prediction

Prediction: Irritant
Probability: 1
Enrichment: 1.18
Bayesian Score: 1.48
Mahalanobis Distance: 6.31
Mahalanobis Distance p-value: 1

Prediction: Positive if the Bayesian score is above the estimated best cutoff value from minimizing the false positive and false negative rate.
Probability: The esimated probability that the sample is in the positive category. This assumes that the Bayesian score follows a normal distribution and is different from the prediction using a cutoff.
Enrichment: An estimate of enrichment, that is, the increased likelihood (versus random) of this sample being in the category.
Bayesian Score: The standard Laplacian-modified Bayesian score.
Mahalanobis Distance: The Mahalanobis distance (MD) is the distance to the center of the training data. The larger the MD, the less trustworthy the prediction.
Mahalanobis Distance p-value: The p-value gives the fraction of training data with an MD greater than or equal to the one for the given sample, assuming normally distributed data. The smaller the p-value, the less trustworthy the prediciton. For highly non-normal X properties (e.g., fingerprints), the MD p-value is wildly inaccurate.

Structural Similar Compounds

Name	BENZANILIDE;2';2'''-DITHIOBIS-	ANTHRAQUINONE; 1;4-BIS(p-TOLYLAMINO)-	ANTHRAQUINONE; 1;1'-IMINODI-
Structure	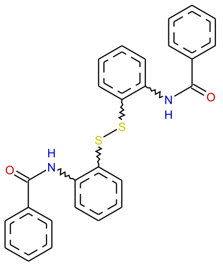	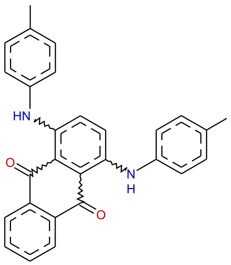	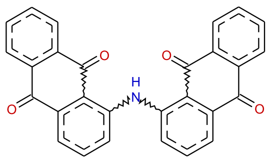
Actual Endpoint	Non-Irritant	Irritant	Irritant
Predicted Endpoint	Non-Irritant	Non-Irritant	Irritant
Distance	0.661	0.672	0.729
Reference	28ZPAK-;173;72	28ZPAK -;124;72	28ZPAK-;125;72

Model Applicability

Unknown features are fingerprint features in the query molecule, but not found or appearing too infrequently in the training set.

1. All properties and OPS components are within expected ranges.
2. Unknown FCFP_2 feature: -1564473960: [*]n1:[*]:[*]:[c](:[*]):[c]:1:n:[*]
3. Unknown FCFP_2 feature: 179977000: [*][c]1:[*]:[*]:n:n:1[c](:[*]):[*]
4. Unknown FCFP_2 feature: -1151884458: [*]N[c](:n:[*]):[c](:[*]):[*]
5. Unknown FCFP_2 feature: 1294285001: [*]=NN[c](:[*]):[*]
6. Unknown FCFP_2 feature: 581019816: [*]N\N=C\[*]

Feature Contribution

Top features for positive contribution

Fingerprint	Bit/Smiles	Feature Structure	Score	Irritant in training set
FCFP_12	1747237384	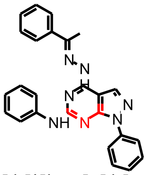 [*][c](:[*]):n:[c](:[*]):[*]	0.208	44 out of 44

FCFP_12	-124655670	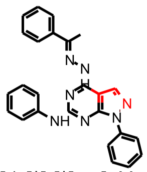 [*][c]1:[*]:[*]:n:[cH]:1	0.2	16 out of 16
FCFP_12	-1151914249	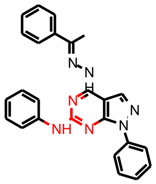 [*]N[c](:n:[*]):n:[*]	0.19	9 out of 9
**Top Features for negative contribution**				
**Fingerprint**	**Bit/Smiles**	**Feature Structure**	**Score**	**Irritant in training set**
FCFP_12	-1698724694	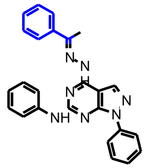 [*]C(=[*])[c]1:[cH]:[cH]:[cH]:[cH]:[cH]:1	-0.0964	107 out of 146
FCFP_12	975909016	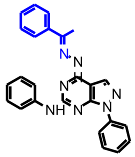 [*]\N=C(/C)\[c]1:[cH]:[cH]:[cH]:[cH]:[cH]:1	-0.0639	6 out of 8
FCFP_12	307419094	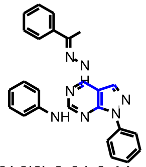 [*][c](:[*]):[c]1:[cH]:[*]:[*]:[c]:1:[*]	0	43 out of 52

12b TOPKAT_Ocular_Irritancy_None_vs_Irritant

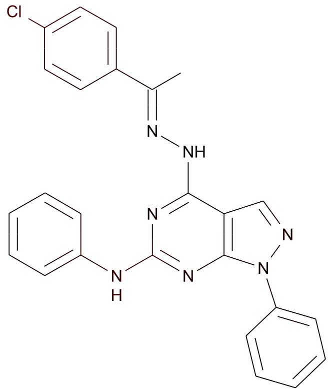

$C_{25}H_{20}ClN_7$
Molecular Weight: 453.9262
ALogP: 6.55
Rotatable Bonds: 6
Acceptors: 6
Donors: 2

Structural Similar Compounds

Name	ANTHRAQUINONE; 1;4-BIS(p-TOLYLAMINO)-	BENZANILIDE;2';2'''-DITHIOBIS-	DINAPHTHO(1;2;3-CD:3';2';1'-IM)PERYLENE-5;10-DIONE;16;17-DIHYDROXY
Structure	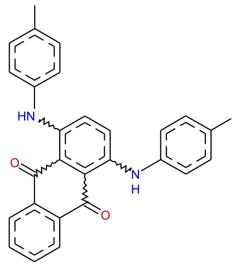	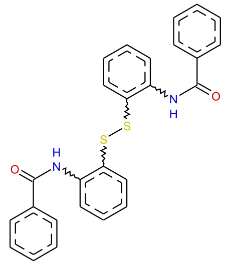	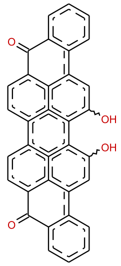
Actual Endpoint	Irritant	Non-Irritant	Irritant
Predicted Endpoint	Non-Irritant	Non-Irritant	Irritant
Distance	0.666	0.666	0.722
Reference	28ZPAK -;124;72	28ZPAK-;173;72	28ZPAK-;104;72

Model Prediction

Prediction: Irritant
Probability: 1
Enrichment: 1.18
Bayesian Score: 2.97
Mahalanobis Distance: 6.49
Mahalanobis Distance p-value: 1

Prediction: Positive if the Bayesian score is above the estimated best cutoff value from minimizing the false positive and false negative rate.
Probability: The esimated probability that the sample is in the positive category. This assumes that the Bayesian score follows a normal distribution and is different from the prediction using a cutoff.
Enrichment: An estimate of enrichment, that is, the increased likelihood (versus random) of this sample being in the category.
Bayesian Score: The standard Laplacian-modified Bayesian score.
Mahalanobis Distance: The Mahalanobis distance (MD) is the distance to the center of the training data. The larger the MD, the less trustworthy the prediction.
Mahalanobis Distance p-value: The p-value gives the fraction of training data with an MD greater than or equal to the one for the given sample, assuming normally distributed data. The smaller the p-value, the less trustworthy the prediciton. For highly non-normal X properties (e.g., fingerprints), the MD p-value is wildly inaccurate.

Model Applicability

Unknown features are fingerprint features in the query molecule, but not found or appearing too infrequently in the training set.

1. All properties and OPS components are within expected ranges.
2. Unknown FCFP_2 feature: -1564473960: [*]n1:[*]:[*]:[c](:[*]):[c]:1:n:[*]
3. Unknown FCFP_2 feature: 179977000: [*][c]1:[*]:[*]:n:n:1[c](:[*]):[*]
4. Unknown FCFP_2 feature: -1151884458: [*]N[c](:n:[*]):[c](:[*]):[*]
5. Unknown FCFP_2 feature: 1294285001: [*]=NN[c](:[*]):[*]
6. Unknown FCFP_2 feature: 581019816: [*]N\N=C\[*]

Feature Contribution

Top features for positive contribution				
**Fingerprint**	**Bit/Smiles**	**Feature Structure**	**Score**	**Irritant in training set**

FCFP_12	1747237384	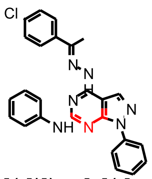 [*][c](:[*]):n:[c](:[*]):[*]	0.208	44 out of 44
FCFP_12	-1508180856	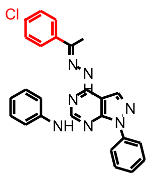 [*][c]1:[cH]:[cH]:[c](Cl):[cH]:[cH]:1	0.2	17 out of 17
FCFP_12	-124655670	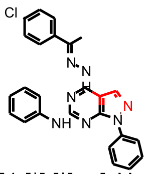 [*][c]1:[*]:[*]:n:[cH]:1	0.2	16 out of 16
**Top Features for negative contribution**				
**Fingerprint**	**Bit/Smiles**	**Feature Structure**	**Score**	**Irritant in training set**
FCFP_12	1	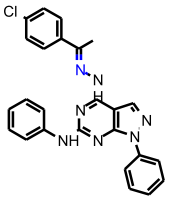 [*]N=[*]	0	872 out of 1051
FCFP_12	0	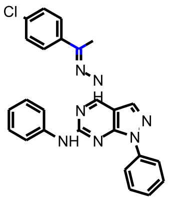 [*]C(=[*])[*]	0	1184 out of 1397

FCFP_12	307419094	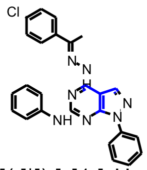 [*][c](:[*]):[c]1:[cH]:[*]:[*]:[c]:1:[*]	0	43 out of 52

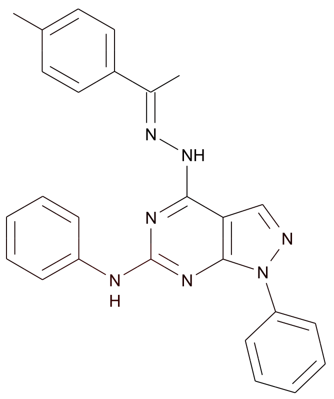

$C_{26}H_{23}N_7$
Molecular Weight: 433.50772
ALogP: 6.372
Rotatable Bonds: 6
Acceptors: 6
Donors: 2

Model Prediction

Prediction: Irritant
Probability: 1
Enrichment: 1.18
Bayesian Score: 1.97
Mahalanobis Distance: 6.44
Mahalanobis Distance p-value: 1

Prediction: Positive if the Bayesian score is above the estimated best cutoff value from minimizing the false positive and false negative rate.
Probability: The esimated probability that the sample is in the positive category. This assumes that the Bayesian score follows a normal distribution and is different from the prediction using a cutoff.
Enrichment: An estimate of enrichment, that is, the increased likelihood (versus random) of this sample being in the category.
Bayesian Score: The standard Laplacian-modified Bayesian score.
Mahalanobis Distance: The Mahalanobis distance (MD) is the distance to the center of the training data. The larger the MD, the less trustworthy the prediction.
Mahalanobis Distance p-value: The p-value gives the fraction of training data with an MD greater than or equal to the one for the given sample, assuming normally distributed data. The smaller the p-value, the less trustworthy the prediciton. For highly non-normal X properties (e.g., fingerprints), the MD p-value is wildly inaccurate.

Structural Similar Compounds

Name	ANTHRAQUINONE; 1;4-BIS(p-TOLYLAMINO)-	BENZANILIDE;2';2'''-DITHIOBIS-	DINAPHTHO(1;2;3-CD:3';2';1'-IM)PERYLENE-5;10-DIONE;16;17-DIHYDROXY
Structure	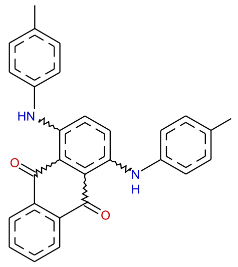	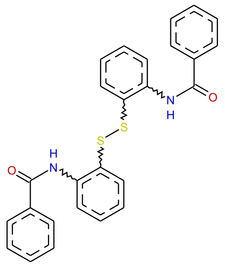	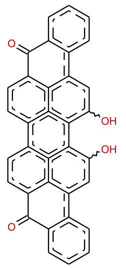
Actual Endpoint	Irritant	Non-Irritant	Irritant
Predicted Endpoint	Non-Irritant	Non-Irritant	Irritant
Distance	0.650	0.667	0.731
Reference	28ZPAK -;124;72	28ZPAK-;173;72	28ZPAK-;104;72

Model Applicability

Unknown features are fingerprint features in the query molecule, but not found or appearing too infrequently in the training set.

1. All properties and OPS components are within expected ranges.
2. Unknown FCFP_2 feature: -1564473960: [*]n1:[*]:[*]:[c](:[*]):[c]:1:n:[*]
3. Unknown FCFP_2 feature: 179977000: [*][c]1:[*]:[*]:n:n:1[c](:[*]):[*]
4. Unknown FCFP_2 feature: -1151884458: [*]N[c](:n:[*]):[c](:[*]):[*]
5. Unknown FCFP_2 feature: 1294285001: [*]=NN[c](:[*]):[*]
6. Unknown FCFP_2 feature: 581019816: [*]N\N=C\[*]

Feature Contribution

Top features for positive contribution				
Fingerprint	Bit/Smiles	Feature Structure	Score	Irritant in training set

FCFP_12	1747237384	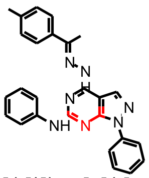 [*][c](:[*]):n:[c](:[*]):[*]	0.208	44 out of 44
FCFP_12	-124655670	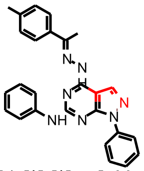 [*][c]1:[*]:[*]:n:[cH]:1	0.2	16 out of 16
FCFP_12	-1151914249	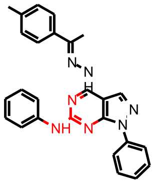 [*]N[c](:n:[*]):n:[*]	0.19	9 out of 9
**Top Features for negative contribution**				
**Fingerprint**	**Bit/Smiles**	**Feature Structure**	**Score**	**Irritant in training set**
FCFP_12	203677720	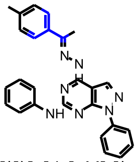 [*]C([*])[c]1:[cH]:[*]:[*]:[c]:1[*]	0	319 out of 382
FCFP_12	0	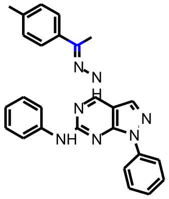 [*]C(=[*])[*]	0	1184 out of 1397

FCFP_12	307419094	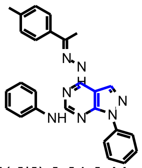 [*][c](:[*]):[c]1:[cH]:[*]:[*]:[c]:1:[*]	0	43 out of 52

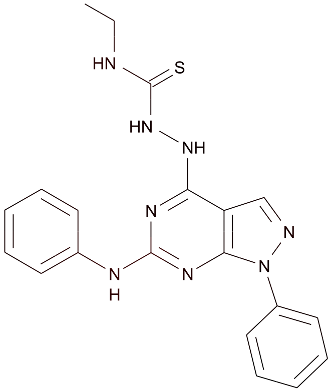

$C_{20}H_{20}N_8S$
Molecular Weight: 404.4914
ALogP: 4.99
Rotatable Bonds: 8
Acceptors: 6
Donors: 4

Structural Similar Compounds

Name	4;4'-DIAMINO-1;1'-DIANTHRIMIDE	ANTHRAQUINONE; 1;5-DIAMINO-4;8-DIHYDROXY-3-(p-METHOXYPHENYL)-	Disperse Black 9
Structure	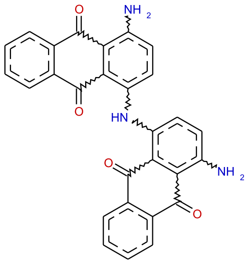	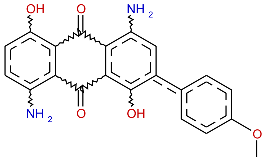	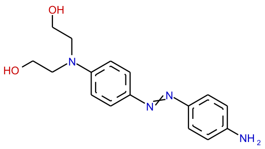
Actual Endpoint	Irritant	Irritant	Non-Irritant
Predicted Endpoint	Irritant	Irritant	Non-Irritant
Distance	0.786	0.791	0.834
Reference	28ZPAK-;125;72	28ZPAK 245;72	J. Am. Coll. Toxicol. 5(3):205;1986

Model Prediction

Prediction: Irritant
Probability: 1
Enrichment: 1.18
Bayesian Score: 2.3
Mahalanobis Distance: 7.49
Mahalanobis Distance p-value: 0.985

Prediction: Positive if the Bayesian score is above the estimated best cutoff value from minimizing the false positive and false negative rate.
Probability: The esimated probability that the sample is in the positive category. This assumes that the Bayesian score follows a normal distribution and is different from the prediction using a cutoff.
Enrichment: An estimate of enrichment, that is, the increased likelihood (versus random) of this sample being in the category.
Bayesian Score: The standard Laplacian-modified Bayesian score.
Mahalanobis Distance: The Mahalanobis distance (MD) is the distance to the center of the training data. The larger the MD, the less trustworthy the prediction.
Mahalanobis Distance p-value: The p-value gives the fraction of training data with an MD greater than or equal to the one for the given sample, assuming normally distributed data. The smaller the p-value, the less trustworthy the prediciton. For highly non-normal X properties (e.g., fingerprints), the MD p-value is wildly inaccurate.

Model Applicability

Unknown features are fingerprint features in the query molecule, but not found or appearing too infrequently in the training set.

1. All properties and OPS components are within expected ranges.
2. Unknown FCFP_2 feature: -1564473960: [*]n1:[*]:[*]:[c](:[*]):[c]:1:n:[*]
3. Unknown FCFP_2 feature: 179977000: [*][c]1:[*]:[*]:n:n:1[c](:[*]):[*]
4. Unknown FCFP_2 feature: -1151884458: [*]N[c](:n:[*]):[c](:[*]):[*]
5. Unknown FCFP_2 feature: -885461129: [*]NNC(=[*])[*]

Feature Contribution

Top features for positive contribution				
Fingerprint	Bit/Smiles	Feature Structure	Score	Irritant in training set

FCFP_12	1747237384	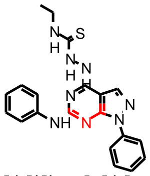 [*][c](:[*]):n:[c](:[*]):[*]	0.208	44 out of 44
FCFP_12	-124655670	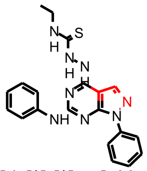 [*][c]1:[*]:[*]:n:[cH]:1	0.2	16 out of 16
FCFP_12	-1151914249	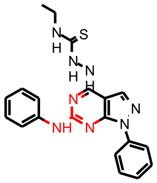 [*]N[c](:n:[*]):n:[*]	0.19	9 out of 9

Top Features for negative contribution

Fingerprint	Bit/Smiles	Feature Structure	Score	Irritant in training set
FCFP_12	991735244	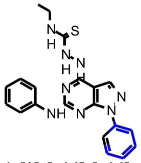 [*][c]1:[*]:[cH]:[cH] :[cH]:[cH]:1	0	237 out of 291
FCFP_12	136597326	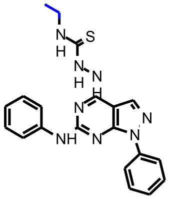 [*]CC	0	612 out of 753

FCFP_12	1872154524	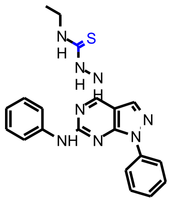<br>[*]C(=S)[*]	0	563 out of 690

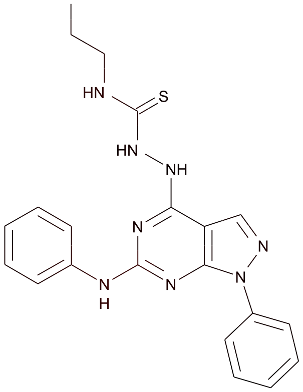

$C_{21}H_{22}N_8S$
Molecular Weight: 418.51798
ALogP: 5.514
Rotatable Bonds: 9
Acceptors: 6
Donors: 4

Structural Similar Compounds

Name	4;4'-DIAMINO-1;1'-DIANTHRIMIDE	ANTHRAQUINONE; 1;5-DIAMINO-4;8-DIHYDROXY-3-(p-METHOXYPHENYL)-	Disperse Black 9
Structure	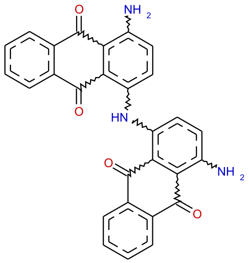	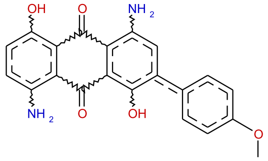	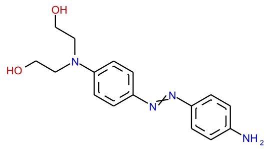
Actual Endpoint	Irritant	Irritant	Non-Irritant
Predicted Endpoint	Irritant	Irritant	Non-Irritant
Distance	0.813	0.849	0.877
Reference	28ZPAK-;125;72	28ZPAK 245;72	J. Am. Coll. Toxicol. 5(3):205;1986

Model Prediction

Prediction: Irritant
Probability: 1
Enrichment: 1.18
Bayesian Score: 2.41
Mahalanobis Distance: 8.23
Mahalanobis Distance p-value: 0.867

Prediction: Positive if the Bayesian score is above the estimated best cutoff value from minimizing the false positive and false negative rate.
Probability: The esimated probability that the sample is in the positive category. This assumes that the Bayesian score follows a normal distribution and is different from the prediction using a cutoff.
Enrichment: An estimate of enrichment, that is, the increased likelihood (versus random) of this sample being in the category.
Bayesian Score: The standard Laplacian-modified Bayesian score.
Mahalanobis Distance: The Mahalanobis distance (MD) is the distance to the center of the training data. The larger the MD, the less trustworthy the prediction.
Mahalanobis Distance p-value: The p-value gives the fraction of training data with an MD greater than or equal to the one for the given sample, assuming normally distributed data. The smaller the p-value, the less trustworthy the prediciton. For highly non-normal X properties (e.g., fingerprints), the MD p-value is wildly inaccurate.

Model Applicability

Unknown features are fingerprint features in the query molecule, but not found or appearing too infrequently in the training set.

1. All properties and OPS components are within expected ranges.
2. Unknown FCFP_2 feature: -1564473960: [*]n1:[*]:[*]:[c](:[*]):[c]:1:n:[*]
3. Unknown FCFP_2 feature: 179977000: [*][c]1:[*]:[*]:n:n:1[c](:[*]):[*]
4. Unknown FCFP_2 feature: -1151884458: [*]N[c](:n:[*]):[c](:[*]):[*]
5. Unknown FCFP_2 feature: -885461129: [*]NNC(=[*])[*]

Feature Contribution

Top features for positive contribution

Fingerprint	Bit/Smiles	Feature Structure	Score	Irritant in training set

FCFP_12	1747237384	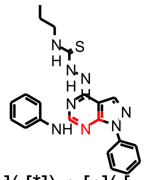 [*][c](:[*]):n:[c](:[*]):[*]	0.208	44 out of 44
FCFP_12	-124655670	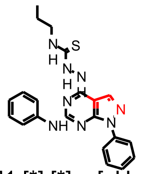 [*][c]1:[*]:[*]:n:[cH]:1	0.2	16 out of 16
FCFP_12	-1151914249	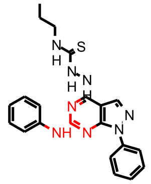 [*]N[c](:n:[*]):n:[*]	0.19	9 out of 9
**Top Features for negative contribution**				
**Fingerprint**	**Bit/Smiles**	**Feature Structure**	**Score**	**Irritant in training set**
FCFP_12	1872154524	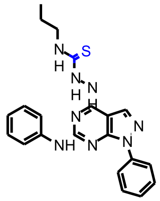 [*]C(=S)[*]	0	563 out of 690
FCFP_12	0	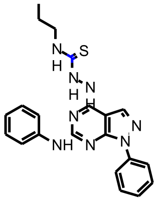 [*]C(=[*])[*]	0	1184 out of 1397

FCFP_12	1	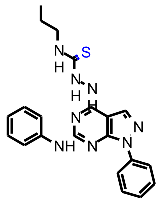 [*]N=[*]	0	872 out of 1051

Erlotinib

TOPKAT_Ocular_Irritancy_None_vs_Irritant

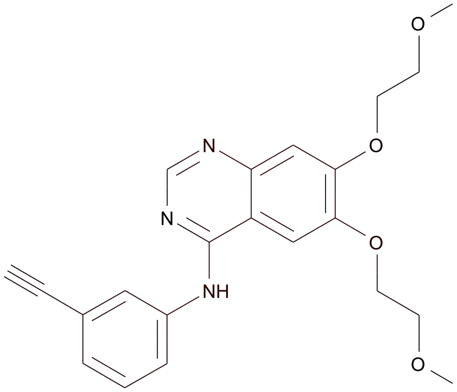

$C_{22}H_{23}N_{3}O_{4}$
Molecular Weight: 393.43572
ALogP: 4.309
Rotatable Bonds: 10
Acceptors: 7
Donors: 1

Structural Similar Compounds

Name	Benzoic acid; p-(N-butyl-2-(butylamino)acetamido)-; butyl ester;	COLCHICINE	Cinchoninamide; 2-butoxy-N-(2-(diethylamino)ethyl)-; monohydrochloride
Structure	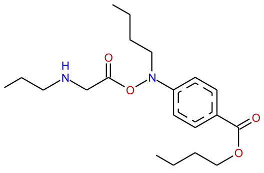	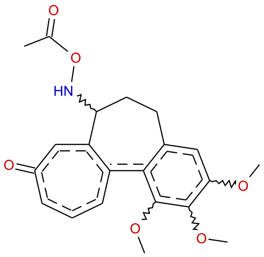	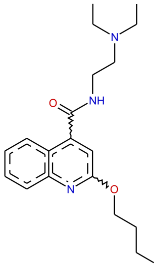
Actual Endpoint	Irritant	Irritant	Irritant
Predicted Endpoint	Non-Irritant	Irritant	Irritant
Distance	0.637	0.682	0.747
Reference	Arzneimittel-Forschung 8;609;58	AJOPAA 31;837;48	Arzneimittel-Forschung 8;181;58

Model Prediction

Prediction: Irritant
Probability: 1
Enrichment: 1.18
Bayesian Score: 2.13
Mahalanobis Distance: 9.75
Mahalanobis Distance p-value: 0.161

Prediction: Positive if the Bayesian score is above the estimated best cutoff value from minimizing the false positive and false negative rate.
Probability: The esimated probability that the sample is in the positive category. This assumes that the Bayesian score follows a normal distribution and is different from the prediction using a cutoff.
Enrichment: An estimate of enrichment, that is, the increased likelihood (versus random) of this sample being in the category.
Bayesian Score: The standard Laplacian-modified Bayesian score.
Mahalanobis Distance: The Mahalanobis distance (MD) is the distance to the center of the training data. The larger the MD, the less trustworthy the prediction.
Mahalanobis Distance p-value: The p-value gives the fraction of training data with an MD greater than or equal to the one for the given sample, assuming normally distributed data. The smaller the p-value, the less trustworthy the prediciton. For highly non-normal X properties (e.g., fingerprints), the MD p-value is wildly inaccurate.

Model Applicability

Unknown features are fingerprint features in the query molecule, but not found or appearing too infrequently in the training set.

1. All properties and OPS components are within expected ranges.
2. Unknown FCFP_2 feature: -124685461: [*]:n:c:n:[*]
3. Unknown FCFP_2 feature: -1151884458: [*]N[c](:n:[*]):[c](:[*]):[*]
4. Unknown FCFP_2 feature: 902193919: [*]:[c](:[*])C#C

Feature Contribution

Top features for positive contribution

Fingerprint	Bit/Smiles	Feature Structure	Score	Irritant in training set
FCFP_12	1747237384	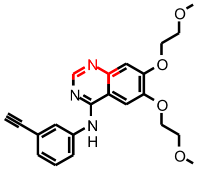 [*][c](:[*]):n:[c](:[*]):[*]	0.208	44 out of 44

FCFP_12	178336375	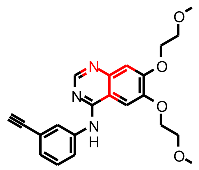 [*]:[cH]:[c](:n:[*]):[c](:[*]):[*]	0.202	19 out of 19
FCFP_12	17	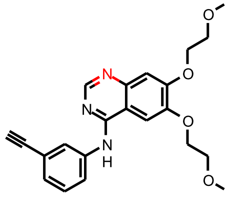 [*]:n:[*]	0.189	48 out of 49
**Top Features for negative contribution**				
**Fingerprint**	**Bit/Smiles**	**Feature Structure**	**Score**	**Irritant in training set**
FCFP_12	203677720	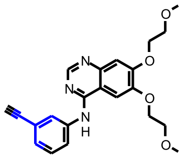 [*]C([*])[c]1:[cH]:[*]:[*]:[c]:1[*]	0	319 out of 382
FCFP_12	-1143715940	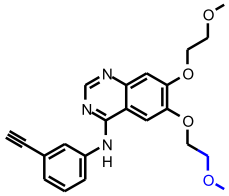 [*]COC	0	454 out of 590
FCFP_12	136627117	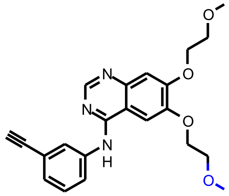 [*]OC	0	96 out of 113

AND Enantiomer

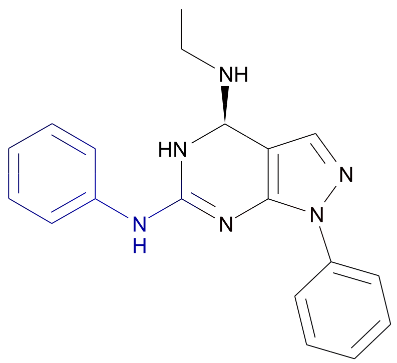

$C_{19}H_{20}N_6$

Molecular Weight: 332.4023

ALogP: 3.039

Rotatable Bonds: 5

Acceptors: 5

Donors: 3

Model Prediction

Prediction: Non-Irritant

Probability: 0.853

Enrichment: 0.927

Bayesian Score: -3.18

Mahalanobis Distance: 8.62

Mahalanobis Distance p-value: 0.629

Prediction: Positive if the Bayesian score is above the estimated best cutoff value from minimizing the false positive and false negative rate.
Probability: The esimated probability that the sample is in the positive category. This assumes that the Bayesian score follows a normal distribution and is different from the prediction using a cutoff.
Enrichment: An estimate of enrichment, that is, the increased likelihood (versus random) of this sample being in the category.
Bayesian Score: The standard Laplacian-modified Bayesian score.
Mahalanobis Distance: The Mahalanobis distance (MD) is the distance to the center of the training data. The larger the MD, the less trustworthy the prediction.
Mahalanobis Distance p-value: The p-value gives the fraction of training data with an MD greater than or equal to the one for the given sample, assuming normally distributed data. The smaller the p-value, the less trustworthy the prediciton. For highly non-normal X properties (e.g., fingerprints), the MD p-value is wildly inaccurate.

Structural Similar Compounds

Name	p-Acetophenetidide, 3'-(bis(2-hydroxyethyl)amino)-	5-Norbornene-2,3-dicarboxylic acid, 1,4,5,6,7,7-hexachloro-	1-Amino-2-bromo-4-hydroxyanthraquinone
Structure	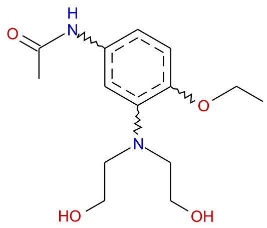	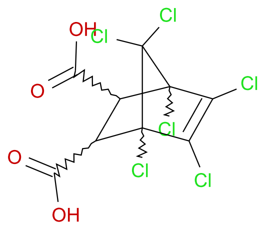	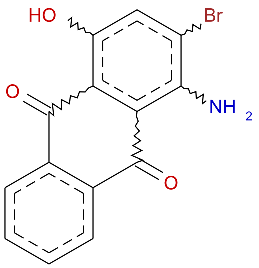
Actual Endpoint	Non-Irritant	Irritant	Non-Irritant
Predicted Endpoint	Non-Irritant	Irritant	Non-Irritant
Distance	0.774	0.793	0.833
Reference	28ZPAK -,100,72	85JCAE "Prehled Prumyslove Toxikologie; Organicke Latky," Marhold, J., Prague , Czechoslovakia, Avicenum, 1986 Volume(issue)/page/year: -,581,1986	28ZPAK -,83,72

Model Applicability

Unknown features are fingerprint features in the query molecule, but not found or appearing too infrequently in the training set.

1. All properties and OPS components are within expected ranges.
2. Unknown FCFP_2 feature: 179977000: [*][c]1:[*]:[*]:n:n:1[c](:[*]):[*]
3. Unknown FCFP_2 feature: 2119857014: [*]NC(N[*])[c](:[*]):[*]

Feature Contribution

Top features for positive contribution				
Fingerprint	Bit/Smiles	Feature Structure	Score	Irritant in training set

FCFP_12	-124655670	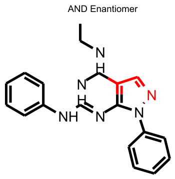 [*][c]1:[*]:[*]:n:[cH]:1	0.0821	13 out of 13
FCFP_12	580453787	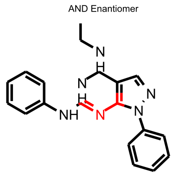 [*]C(=N[c](:[*]):[*])[*]	0.0795	9 out of 9
FCFP_12	-1410049896	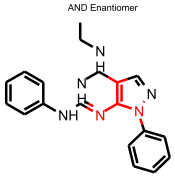 [*]n1:[*]:[*]:[c]([*]):[c]:1N=[*]	0.0734	5 out of 5

Top Features for negative contribution

Fingerprint	Bit/Smiles	Feature Structure	Score	Irritant in training set
FCFP_12	-1838187238	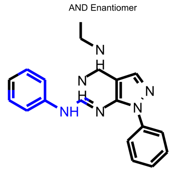 [*]C(=[*])N[c]1:[cH]:[cH]:[*]:[cH]:[cH]:1	-0.692	5 out of 12
FCFP_12	-792685140	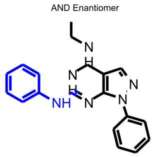 [*]C(=[*])N[c]1:[cH]:[cH]:[cH]:[cH]:[cH]:1	-0.65	0 out of 1

FCFP_12	1294255210	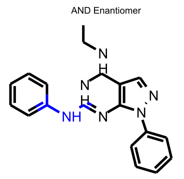<br>[*]C(=[*])N[c](:[*]):[*]	-0.486	12 out of 22

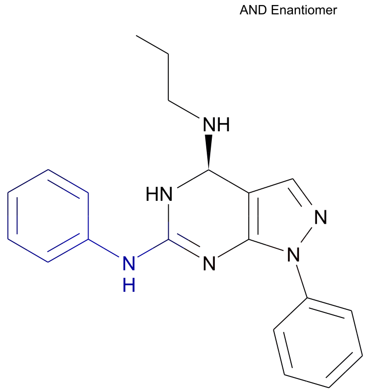

$C_{20}H_{22}N_6$
Molecular Weight: 346.42888
ALogP: 3.562
Rotatable Bonds: 6
Acceptors: 5
Donors: 3

Structural Similar Compounds

Name	p-Acetophenetidide, 3'-(bis(2-hydroxyethyl)amino)-	5-Norbornene-2,3-dicarboxylic acid, 1,4,5,6,7,7-hexachloro-	Disiloxane, 1,3-bis(3-aminopropyl)-1,1,3,3-tetramethyl-
Structure	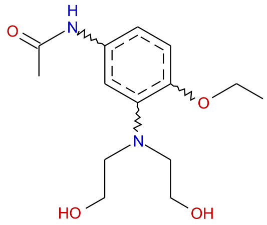	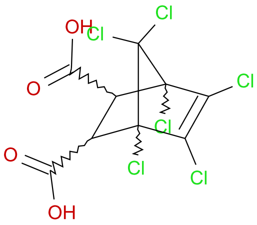	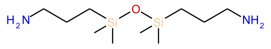
Actual Endpoint	Non-Irritant	Irritant	Irritant
Predicted Endpoint	Non-Irritant	Irritant	Irritant
Distance	0.814	0.821	0.842
Reference	28ZPAK -,100,72	85JCAE "Prehled Prumyslove Toxikologie; Organicke Latky," Marhold, J., Prague , Czechoslovakia, Avicenum, 1986 Volume(issue)/page/year: -,581,1986	NTIS** National Technical Information Service. (Springfield, VA 22161) Forme rly U.S. Clearinghouse for Scientific & Technical Information. Volume(issue)/pag e/year: OTS0535667

Model Prediction

Prediction: Non-Irritant
Probability: 0.836
Enrichment: 0.907
Bayesian Score: -3.3
Mahalanobis Distance: 9.04
Mahalanobis Distance p-value: 0.398

Prediction: Positive if the Bayesian score is above the estimated best cutoff value from minimizing the false positive and false negative rate.
Probability: The esimated probability that the sample is in the positive category. This assumes that the Bayesian score follows a normal distribution and is different from the prediction using a cutoff.
Enrichment: An estimate of enrichment, that is, the increased likelihood (versus random) of this sample being in the category.
Bayesian Score: The standard Laplacian-modified Bayesian score.
Mahalanobis Distance: The Mahalanobis distance (MD) is the distance to the center of the training data. The larger the MD, the less trustworthy the prediction.
Mahalanobis Distance p-value: The p-value gives the fraction of training data with an MD greater than or equal to the one for the given sample, assuming normally distributed data. The smaller the p-value, the less trustworthy the prediciton. For highly non-normal X properties (e.g., fingerprints), the MD p-value is wildly inaccurate.

Model Applicability

Unknown features are fingerprint features in the query molecule, but not found or appearing too infrequently in the training set.

1. All properties and OPS components are within expected ranges.
2. Unknown FCFP_2 feature: 179977000: [*][c]1:[*]:[*]:n:n:1[c](:[*]):[*]
3. Unknown FCFP_2 feature: 2119857014: [*]NC(N[*])[c](:[*]):[*]

Feature Contribution

Top features for positive contribution				
Fingerprint	Bit/Smiles	Feature Structure	Score	Irritant in training set

FCFP_12	-124655670	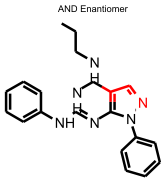 [*][c]1:[*]:[*]:n:[cH]:1	0.0821	13 out of 13
FCFP_12	580453787	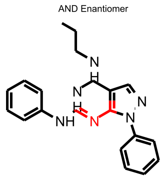 [*]C(=N[c](:[*]):[*])[*]	0.0795	9 out of 9
FCFP_12	4427049	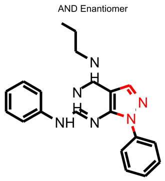 [*]n1:[*]:[*]:[cH]:n:1	0.0734	5 out of 5
**Top Features for negative contribution**				
**Fingerprint**	**Bit/Smiles**	**Feature Structure**	**Score**	**Irritant in training set**
FCFP_12	-1838187238	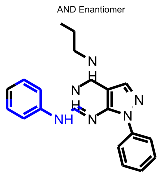 [*]C(=[*])N[c]1:[cH]:[cH]:[*]:[cH]:[cH]:1	-0.692	5 out of 12
FCFP_12	-792685140	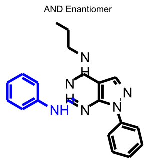 [*]C(=[*])N[c]1:[cH]:[cH]:[cH]:[cH]:[cH]:1	-0.65	0 out of 1

FCFP_12	1294255210	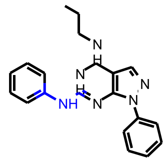 [*]C(=[*])N[c](:[*]):[*]	-0.486	12 out of 22

AND Enantiomer

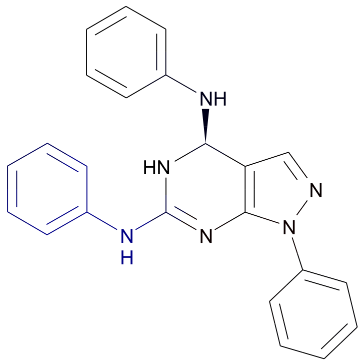

$C_{23}H_{20}N_6$
Molecular Weight: 380.4451
ALogP: 4.544
Rotatable Bonds: 5
Acceptors: 5
Donors: 3

Model Prediction

Prediction: Non-Irritant
Probability: 0.844
Enrichment: 0.917
Bayesian Score: -3.24
Mahalanobis Distance: 8.7
Mahalanobis Distance p-value: 0.581

Prediction: Positive if the Bayesian score is above the estimated best cutoff value from minimizing the false positive and false negative rate.
Probability: The esimated probability that the sample is in the positive category. This assumes that the Bayesian score follows a normal distribution and is different from the prediction using a cutoff.
Enrichment: An estimate of enrichment, that is, the increased likelihood (versus random) of this sample being in the category.
Bayesian Score: The standard Laplacian-modified Bayesian score.
Mahalanobis Distance: The Mahalanobis distance (MD) is the distance to the center of the training data. The larger the MD, the less trustworthy the prediction.
Mahalanobis Distance p-value: The p-value gives the fraction of training data with an MD greater than or equal to the one for the given sample, assuming normally distributed data. The smaller the p-value, the less trustworthy the prediciton. For highly non-normal X properties (e.g., fingerprints), the MD p-value is wildly inaccurate.

Structural Similar Compounds

Name	Sulfide, bis(4-t-butyl-m-cresyl)-	5-Norbornene-2,3-dicarboxylic acid, 1,4,5,6,7,7-hexachloro-	Phenol, 4-(3-carbazolylamino)-
Structure	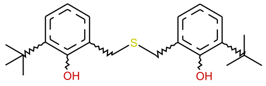	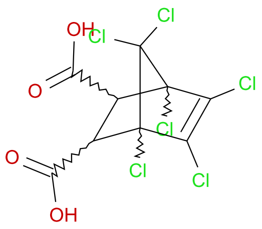	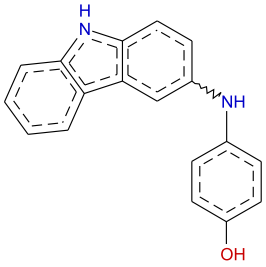
Actual Endpoint	Irritant	Irritant	Irritant
Predicted Endpoint	Irritant	Irritant	Non-Irritant
Distance	0.806	0.830	0.882
Reference	AMIHBC AMA Archives of Industrial Hygiene and Occupational Medicine. (Chicago , IL) V.2-10, 1950-54. For publisher information, see AEHLAU. Volume(issue)/pag e/year: 5,311,1952	85JCAE "Prehled Prumyslove Toxikologie; Organicke Latky," Marhold, J., Prague , Czechoslovakia, Avicenum, 1986 Volume(issue)/page/year: -,581,1986	85JCAE "Prehled Prumyslove Toxikologie; Organicke Latky," Marhold, J., Prague , Czechoslovakia, Avicenum, 1986 Volume(issue)/page/year: -,825,1986

Model Applicability

Unknown features are fingerprint features in the query molecule, but not found or appearing too infrequently in the training set.

1. All properties and OPS components are within expected ranges.
2. Unknown FCFP_2 feature: 179977000: [*][c]1:[*]:[*]:n:n:1[c](:[*]):[*]
3. Unknown FCFP_2 feature: 2119857014: [*]NC(N[*])[c](:[*]):[*]

Feature Contribution

Top features for positive contribution				
Fingerprint	Bit/Smiles	Feature Structure	Score	Irritant in training set

FCFP_12	-124655670	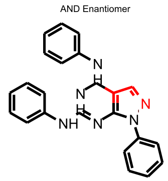 [*][c]1:[*]:[*]:n:[cH]:1	0.0821	13 out of 13
FCFP_12	580453787	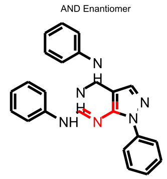 [*]C(=N[c](:[*]):[*])[*]	0.0795	9 out of 9
FCFP_12	4427049	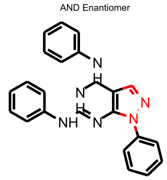 [*]n1:[*]:[*]:[cH]:n:1	0.0734	5 out of 5
**Top Features for negative contribution**				
**Fingerprint**	**Bit/Smiles**	**Feature Structure**	**Score**	**Irritant in training set**
FCFP_12	-1838187238	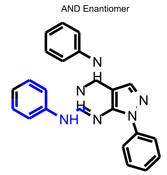 [*]C(=[*])N[c]1:[cH]:[cH]:[*]:[cH]:[cH]:1	-0.692	5 out of 12
FCFP_12	-792685140	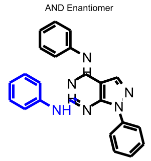 [*]C(=[*])N[c]1:[cH]:[cH]:[cH]:[cH]:[cH]:1	-0.65	0 out of 1

FCFP_12	1294255210	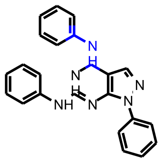 [*]C(=[*])N[c](:[*]):[*]	-0.486	12 out of 22

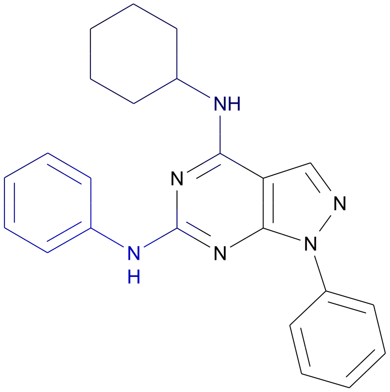

$C_{23}H_{24}N_6$
Molecular Weight: 384.47686
ALogP: 5.953
Rotatable Bonds: 5
Acceptors: 5
Donors: 2

Model Prediction

Prediction: Non-Irritant

Probability: 0.0954

Enrichment: 0.104

Bayesian Score: -5.9

Mahalanobis Distance: 10.1

Mahalanobis Distance p-value: 0.0523

Prediction: Positive if the Bayesian score is above the estimated best cutoff value from minimizing the false positive and false negative rate.
Probability: The esimated probability that the sample is in the positive category. This assumes that the Bayesian score follows a normal distribution and is different from the prediction using a cutoff.
Enrichment: An estimate of enrichment, that is, the increased likelihood (versus random) of this sample being in the category.
Bayesian Score: The standard Laplacian-modified Bayesian score.
Mahalanobis Distance: The Mahalanobis distance (MD) is the distance to the center of the training data. The larger the MD, the less trustworthy the prediction.
Mahalanobis Distance p-value: The p-value gives the fraction of training data with an MD greater than or equal to the one for the given sample, assuming normally distributed data. The smaller the p-value, the less trustworthy the prediciton. For highly non-normal X properties (e.g., fingerprints), the MD p-value is wildly inaccurate.

Structural Similar Compounds

Name	Sulfide, bis(4-t-butyl-m-cresyl)-	Anthraquinone, 1,1'-iminodi-	1-Piperazineacetic acid, 4-(2-hydroxyethyl)-alpha-phenyl-, 2,6-xyly l ester, monohydrochloride
Structure	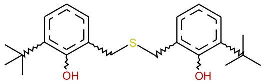	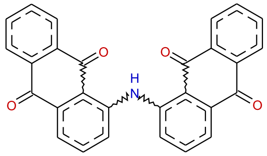	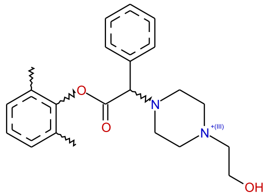
Actual Endpoint	Irritant	Irritant	Irritant
Predicted Endpoint	Irritant	Non-Irritant	Irritant
Distance	0.697	0.747	0.812
Reference	AMIHBC AMA Archives of Industrial Hygiene and Occupational Medicine. (Chicago , IL) V.2-10, 1950-54. For publisher information, see AEHLAU. Volume(issue)/pag e/year: 5,311,1952	85JCAE "Prehled Prumyslove Toxikologie; Organicke Latky," Marhold, J., Prague , Czechoslovakia, Avicenum, 1986 Volume(issue)/page/year: -,735,1986	BCFAAI Bollettino Chimico Farmaceutico. (Societa Editoriale Farmaceutica, Vi a Ausonio 12, 20123 Milan, Italy) V.33- 1894- Volume(issue)/page/year: 107,3 10,1968

Model Applicability

Unknown features are fingerprint features in the query molecule, but not found or appearing too infrequently in the training set.

1. All properties and OPS components are within expected ranges.
2. Unknown FCFP_2 feature: 179977000: [*][c]1:[*]:[*]:n:n:1[c](:[*]):[*]

Feature Contribution

Top features for positive contribution				
Fingerprint	Bit/Smiles	Feature Structure	Score	Irritant in training set

FCFP_12	-124655670	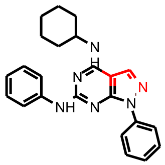 [*][c]1:[*]:[*]:n:[cH]:1	0.0821	13 out of 13
FCFP_12	4427049	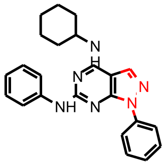 [*]n1:[*]:[*]:[cH]:n:1	0.0734	5 out of 5
FCFP_12	-656492378	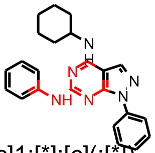 [*][c]1:[*]:[c](:[*]):n:[c](N[c](:[*]):[*]):n:1	0.0658	3 out of 3
**Top Features for negative contribution**				
**Fingerprint**	**Bit/Smiles**	**Feature Structure**	**Score**	**Irritant in training set**
FCFP_12	411414971	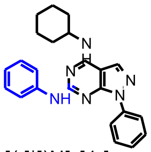 [*]:[c](:[*])N[c]1:[cH]:[cH]:[cH]:[cH]:[cH]:1	-1.31	1 out of 7
FCFP_12	839741273	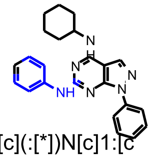 [*]:[c](:[*])N[c]1:[cH]:[cH]:[*]:[cH]:[cH]:1	-0.708	4 out of 10

FCFP_12	-1387302045	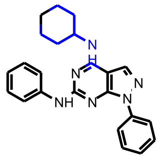 [*]:[c](:[*])NC1CC[*]CC1	-0.65	0 out of 1

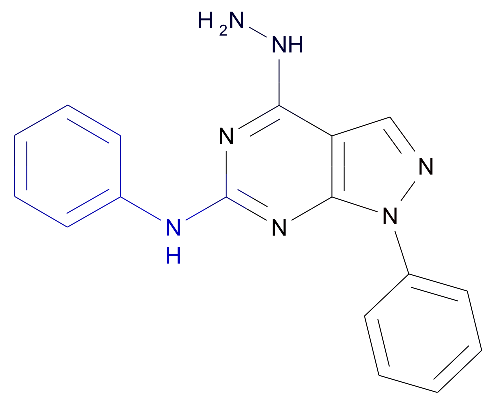

$C_{17}H_{15}N_7$
Molecular Weight: 317.3479
ALogP: 3.562
Rotatable Bonds: 4
Acceptors: 6
Donors: 3

Model Prediction

Prediction: Non-Irritant
Probability: 0.293
Enrichment: 0.318
Bayesian Score: -5.12
Mahalanobis Distance: 8.02
Mahalanobis Distance p-value: 0.879

Prediction: Positive if the Bayesian score is above the estimated best cutoff value from minimizing the false positive and false negative rate.
Probability: The esimated probability that the sample is in the positive category. This assumes that the Bayesian score follows a normal distribution and is different from the prediction using a cutoff.
Enrichment: An estimate of enrichment, that is, the increased likelihood (versus random) of this sample being in the category.
Bayesian Score: The standard Laplacian-modified Bayesian score.
Mahalanobis Distance: The Mahalanobis distance (MD) is the distance to the center of the training data. The larger the MD, the less trustworthy the prediction.
Mahalanobis Distance p-value: The p-value gives the fraction of training data with an MD greater than or equal to the one for the given sample, assuming normally distributed data. The smaller the p-value, the less trustworthy the prediciton. For highly non-normal X properties (e.g., fingerprints), the MD p-value is wildly inaccurate.

Structural Similar Compounds

Name	8-Methylamino-4-hydroxy-2-naphthalene sulfonic acid	Benzenesulfonic acid, 2-anilino-5-nitro-	Anthraquinone, 1,2,4-trihydroxy-
Structure	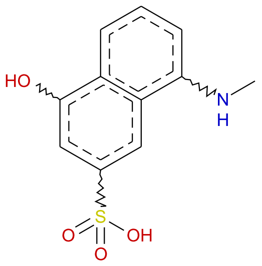	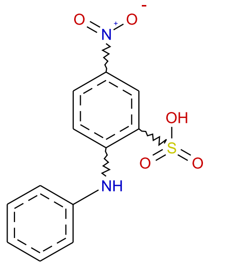	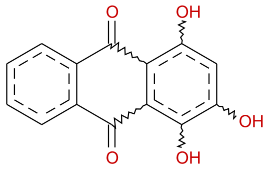
Actual Endpoint	Non-Irritant	Irritant	Non-Irritant
Predicted Endpoint	Non-Irritant	Non-Irritant	Non-Irritant
Distance	0.759	0.777	0.797
Reference	28ZPAK -,190,72	85JCAE "Prehled Prumyslove Toxikologie; Organicke Latky," Marhold, J., Prague , Czechoslovakia, Avicenum, 1986 Volume(issue)/page/year: -,1061,1986	28ZPAK -,103,72

Model Applicability

Unknown features are fingerprint features in the query molecule, but not found or appearing too infrequently in the training set.

1. All properties and OPS components are within expected ranges.
2. Unknown FCFP_2 feature: 179977000: [*][c]1:[*]:[*]:n:n:1[c](:[*]):[*]

Feature Contribution

Top features for positive contribution				
**Fingerprint**	**Bit/Smiles**	**Feature Structure**	**Score**	**Irritant in training set**

FCFP_12	-124655670	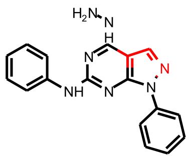 [*][c]1:[*]:[*]:n:[cH]:1	0.0821	13 out of 13
FCFP_12	4427049	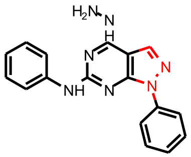 [*]n1:[*]:[*]:[cH]:n: 1	0.0734	5 out of 5
FCFP_12	1551257511	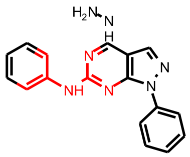 [*]:[cH]:[c](N[c](:n: [*]):n:[*]):[cH]:[*]	0.0658	3 out of 3
**Top Features for negative contribution**				
**Fingerprint**	**Bit/Smiles**	**Feature Structure**	**Score**	**Irritant in training set**
FCFP_12	411414971	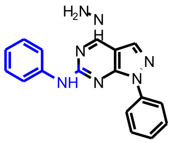 [*]:[c](:[*])N[c]1:[c H]:[cH]:[cH]:[cH]:[c H]:1	-1.31	1 out of 7
FCFP_12	839741273	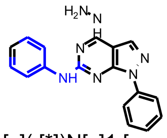 [*]:[c](:[*])N[c]1:[c H]:[cH]:[*]:[cH]:[cH]:1	-0.708	4 out of 10

FCFP_12	1294344583	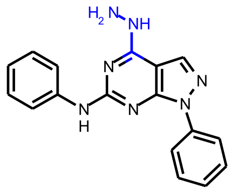 [*]:[c](:[*])NN	-0.65	0 out of 1

11a

TOPKAT_Skin_Irritancy_None_vs_Irritant

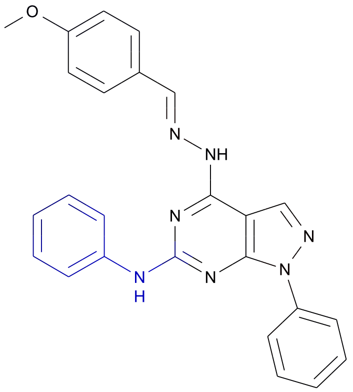

$C_{25}H_{21}N_7O$

Molecular Weight: 435.48054

ALogP: 5.889

Rotatable Bonds: 7

Acceptors: 7

Donors: 2

Structural Similar Compounds

Name	Benzenesulfonic acid, 2,2'-(4,4'-biphenylylenedivinylene)di-, disod ium salt	Anthraquinone, 1,1'-iminodi-	Pregna-1,4-diene-3,20-dione, 21-(acetyloxy)-11-hydroxy-6-methyl-17- (1-oxopropoxy)-, (6-alpha,11-beta)-
Structure	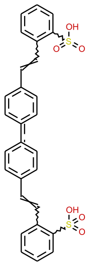	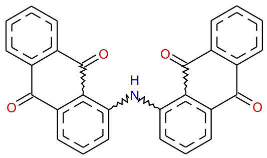	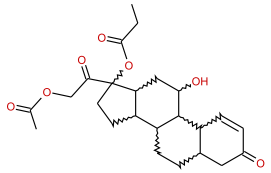
Actual Endpoint	Irritant	Irritant	Irritant
Predicted Endpoint	Non-Irritant	Non-Irritant	Irritant
Distance	0.761	0.829	0.917
Reference	MVCRB3 MVC-Report. (Stockholm, Sweden) No.1-2, 1972-73. Discontinued. Volu me(issue)/page/year: 2,193,1973	85JCAE "Prehled Prumyslove Toxikologie; Organicke Latky," Marhold, J., Prague , Czechoslovakia, Avicenum, 1986 Volume(issue)/page/year: -,735,1986	YACHDS Yakuri to Chiryo. Pharmacology and Therapeutics. (Raifu Saiensu Shup pan K.K., 2-5-13, Yaesu, Chuo-ku, Tokyo 104, Japan) V.1-1972- Volume(issue) /page/year: 19,3103,1991

Model Prediction

Prediction: Non-Irritant

Probability: 0.6

Enrichment: 0.652

Bayesian Score: -4.27

Mahalanobis Distance: 7.84

Mahalanobis Distance p-value: 0.923

Prediction: Positive if the Bayesian score is above the estimated best cutoff value from minimizing the false positive and false negative rate.
Probability: The esimated probability that the sample is in the positive category. This assumes that the Bayesian score follows a normal distribution and is different from the prediction using a cutoff.
Enrichment: An estimate of enrichment, that is, the increased likelihood (versus random) of this sample being in the category.
Bayesian Score: The standard Laplacian-modified Bayesian score.
Mahalanobis Distance: The Mahalanobis distance (MD) is the distance to the center of the training data. The larger the MD, the less trustworthy the prediction.
Mahalanobis Distance p-value: The p-value gives the fraction of training data with an MD greater than or equal to the one for the given sample, assuming normally distributed data. The smaller the p-value, the less trustworthy the prediciton. For highly non-normal X properties (e.g., fingerprints), the MD p-value is wildly inaccurate.

Model Applicability

Unknown features are fingerprint features in the query molecule, but not found or appearing too infrequently in the training set.

1. All properties and OPS components are within expected ranges.
2. Unknown FCFP_2 feature: 179977000: [*][c]1:[*]:[*]:n:n:1[c](:[*]):[*]

Feature Contribution

Top features for positive contribution				
Fingerprint	Bit/Smiles	Feature Structure	Score	Irritant in training set

FCFP_12	-124655670	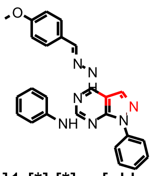 [*][c]1:[*]:[*]:n:[cH]:1	0.0821	13 out of 13
FCFP_12	-2100785893	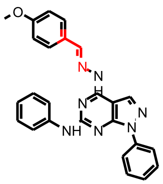 [*]\N=C\[c](:[*]):[*]	0.081	11 out of 11
FCFP_12	4427049	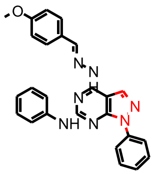 [*]n1:[*]:[*]:[cH]:n:1	0.0734	5 out of 5
**Top Features for negative contribution**				
**Fingerprint**	**Bit/Smiles**	**Feature Structure**	**Score**	**Irritant in training set**
FCFP_12	411414971	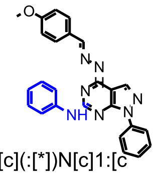 [*]:[c](:[*])N[c]1:[cH]:[cH]:[cH]:[cH]:[cH]:1	-1.31	1 out of 7
FCFP_12	839741273	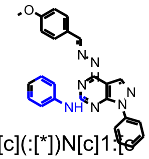 [*]:[c](:[*])N[c]1:[cH]:[cH]:[*]:[cH]:[cH]:1	-0.708	4 out of 10

FCFP_12	-1724769936	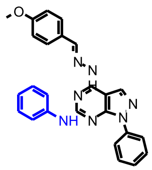 [*]N[c]1:[cH]:[cH]:[cH]:[cH]:[cH]:1	-0.475	11 out of 20

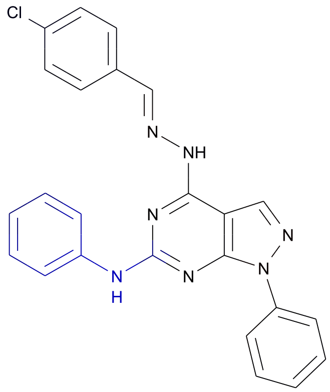

$C_{24}H_{18}ClN_7$
Molecular Weight: 439.89962
ALogP: 6.57
Rotatable Bonds: 6
Acceptors: 6
Donors: 2

Model Prediction

Prediction: Non-Irritant
Probability: 0.549
Enrichment: 0.596
Bayesian Score: -4.41
Mahalanobis Distance: 7.84
Mahalanobis Distance p-value: 0.922

Prediction: Positive if the Bayesian score is above the estimated best cutoff value from minimizing the false positive and false negative rate.
Probability: The esimated probability that the sample is in the positive category. This assumes that the Bayesian score follows a normal distribution and is different from the prediction using a cutoff.
Enrichment: An estimate of enrichment, that is, the increased likelihood (versus random) of this sample being in the category.
Bayesian Score: The standard Laplacian-modified Bayesian score.
Mahalanobis Distance: The Mahalanobis distance (MD) is the distance to the center of the training data. The larger the MD, the less trustworthy the prediction.
Mahalanobis Distance p-value: The p-value gives the fraction of training data with an MD greater than or equal to the one for the given sample, assuming normally distributed data. The smaller the p-value, the less trustworthy the prediciton. For highly non-normal X properties (e.g., fingerprints), the MD p-value is wildly inaccurate.

Structural Similar Compounds

Name	Anthraquinone, 1,1'-iminodi-	Benzenesulfonic acid, 2,2'-(4,4'-biphenylylenedivinylene)di-, disod ium salt	Sulfide, bis(4-t-butyl-m-cresyl)-
Structure	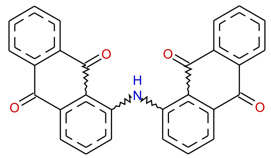	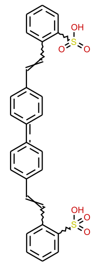	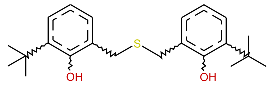
Actual Endpoint	Irritant	Irritant	Irritant
Predicted Endpoint	Non-Irritant	Non-Irritant	Irritant
Distance	0.773	0.788	0.827
Reference	85JCAE "Prehled Prumyslove Toxikologie; Organicke Latky," Marhold, J., Prague , Czechoslovakia, Avicenum, 1986 Volume(issue)/page/year: -,735,1986	MVCRB3 MVC-Report. (Stockholm, Sweden) No.1-2, 1972-73. Discontinued. Volu me(issue)/page/year: 2,193,1973	AMIHBC AMA Archives of Industrial Hygiene and Occupational Medicine. (Chicago , IL) V.2-10, 1950-54. For publisher information, see AEHLAU. Volume(issue)/pag e/year: 5,311,1952

Model Applicability

Unknown features are fingerprint features in the query molecule, but not found or appearing too infrequently in the training set.

1. All properties and OPS components are within expected ranges.
2. Unknown FCFP_2 feature: 179977000: [*][c]1:[*]:[*]:n:n:1[c](:[*]):[*]

Feature Contribution

Top features for positive contribution				
**Fingerprint**	**Bit/Smiles**	**Feature Structure**	**Score**	**Irritant in training set**

FCFP_12	-124655670	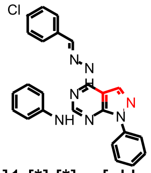 [*][c]1:[*]:[*]:n:[cH]:1	0.0821	13 out of 13
FCFP_12	-2100785893	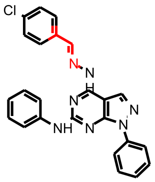 [*]\N=C\[c](:[*]):[*]	0.081	11 out of 11
FCFP_12	-577707014	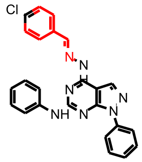 [*]\N=C\[c]1:[cH]:[cH]:[*]:[cH]:[cH]:1	0.0734	5 out of 5

Top Features for negative contribution

Fingerprint	Bit/Smiles	Feature Structure	Score	Irritant in training set
FCFP_12	411414971	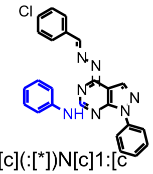 [*]:[c](:[*])N[c]1:[c H]:[cH]:[cH]:[cH]:[c H]:1	-1.31	1 out of 7
FCFP_12	839741273	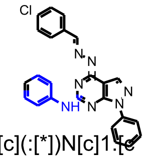 [*]:[c](:[*])N[c]1:[c H]:[cH]:[*]:[cH]:[cH]:1	-0.708	4 out of 10

FCFP_12	-1724769936	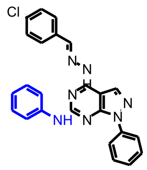 [*]N[c]1:[cH]:[cH]:[cH]:[cH]:[cH]:1	-0.475	11 out of 20

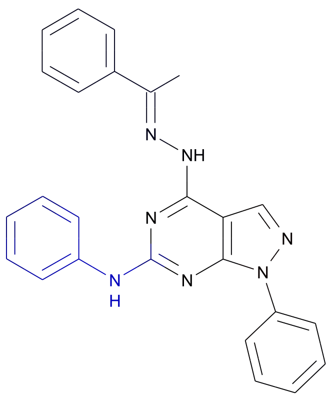

$C_{25}H_{21}N_7$
Molecular Weight: 419.48114
ALogP: 5.886
Rotatable Bonds: 6
Acceptors: 6
Donors: 2

Model Prediction

Prediction: Non-Irritant
Probability: 0.483
Enrichment: 0.524
Bayesian Score: -4.6
Mahalanobis Distance: 7.35
Mahalanobis Distance p-value: 0.984

Prediction: Positive if the Bayesian score is above the estimated best cutoff value from minimizing the false positive and false negative rate.
Probability: The esimated probability that the sample is in the positive category. This assumes that the Bayesian score follows a normal distribution and is different from the prediction using a cutoff.
Enrichment: An estimate of enrichment, that is, the increased likelihood (versus random) of this sample being in the category.
Bayesian Score: The standard Laplacian-modified Bayesian score.
Mahalanobis Distance: The Mahalanobis distance (MD) is the distance to the center of the training data. The larger the MD, the less trustworthy the prediction.
Mahalanobis Distance p-value: The p-value gives the fraction of training data with an MD greater than or equal to the one for the given sample, assuming normally distributed data. The smaller the p-value, the less trustworthy the prediciton. For highly non-normal X properties (e.g., fingerprints), the MD p-value is wildly inaccurate.

Structural Similar Compounds

Name	Anthraquinone, 1,1'-iminodi-	Benzenesulfonic acid, 2,2'-(4,4'-biphenylylenedivinylene)di-, disod ium salt	Sulfide, bis(4-t-butyl-m-cresyl)-
Structure	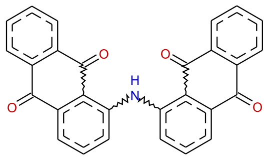	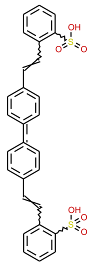	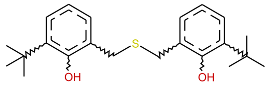
Actual Endpoint	Irritant	Irritant	Irritant
Predicted Endpoint	Non-Irritant	Non-Irritant	Irritant
Distance	0.749	0.803	0.804
Reference	85JCAE "Prehled Prumyslove Toxikologie; Organicke Latky," Marhold, J., Prague , Czechoslovakia, Avicenum, 1986 Volume(issue)/page/year: -,735,1986	MVCRB3 MVC-Report. (Stockholm, Sweden) No.1-2, 1972-73. Discontinued. Volu me(issue)/page/year: 2,193,1973	AMIHBC AMA Archives of Industrial Hygiene and Occupational Medicine. (Chicago , IL) V.2-10, 1950-54. For publisher information, see AEHLAU. Volume(issue)/pag e/year: 5,311,1952

Model Applicability

Unknown features are fingerprint features in the query molecule, but not found or appearing too infrequently in the training set.

1. All properties and OPS components are within expected ranges.
2. Unknown FCFP_2 feature: 179977000: [*][c]1:[*]:[*]:n:n:1[c](:[*]):[*]

Feature Contribution

Top features for positive contribution				
Fingerprint	Bit/Smiles	Feature Structure	Score	Irritant in training set

FCFP_12	-124655670	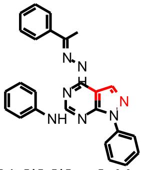 [*][c]1:[*]:[*]:n:[cH]:1	0.0821	13 out of 13
FCFP_12	4427049	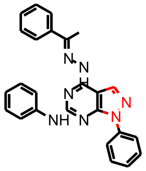 [*]n1:[*]:[*]:[cH]:n:1	0.0734	5 out of 5
FCFP_12	-656492378	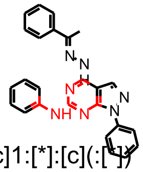 [*][c]1:[*]:[c](:[*]):n:[c](N[c](:[*]):[*]):n:1	0.0658	3 out of 3

Top Features for negative contribution

Fingerprint	Bit/Smiles	Feature Structure	Score	Irritant in training set
FCFP_12	411414971	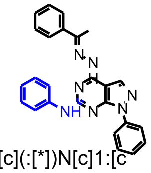 [*]:[c](:[*])N[c]1:[cH]:[cH]:[cH]:[cH]:[cH]:1	-1.31	1 out of 7
FCFP_12	839741273	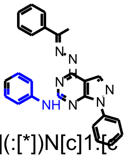 [*]:[c](:[*])N[c]1:[cH]:[cH]:[*]:[cH]:[cH]:1	-0.708	4 out of 10

FCFP_12	-1724769936	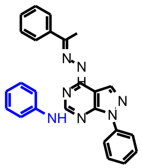 [*]N[c]1:[cH]:[cH]:[cH]:[cH]:1	-0.475	11 out of 20

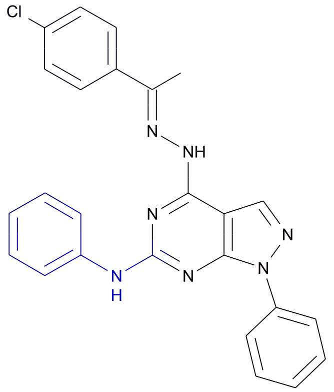

$C_{25}H_{20}ClN_7$
Molecular Weight: 453.9262
ALogP: 6.55
Rotatable Bonds: 6
Acceptors: 6
Donors: 2

Model Prediction

Prediction: Non-Irritant
Probability: 0.436
Enrichment: 0.474
Bayesian Score: -4.72
Mahalanobis Distance: 8.16
Mahalanobis Distance p-value: 0.833

Prediction: Positive if the Bayesian score is above the estimated best cutoff value from minimizing the false positive and false negative rate.
Probability: The esimated probability that the sample is in the positive category. This assumes that the Bayesian score follows a normal distribution and is different from the prediction using a cutoff.
Enrichment: An estimate of enrichment, that is, the increased likelihood (versus random) of this sample being in the category.
Bayesian Score: The standard Laplacian-modified Bayesian score.
Mahalanobis Distance: The Mahalanobis distance (MD) is the distance to the center of the training data. The larger the MD, the less trustworthy the prediction.
Mahalanobis Distance p-value: The p-value gives the fraction of training data with an MD greater than or equal to the one for the given sample, assuming normally distributed data. The smaller the p-value, the less trustworthy the prediciton. For highly non-normal X properties (e.g., fingerprints), the MD p-value is wildly inaccurate.

Structural Similar Compounds

Name	Anthraquinone, 1,1'-iminodi-	Benzenesulfonic acid, 2,2'-(4,4'-biphenylylenedivinylene)di-, disod ium salt	Sulfide, bis(4-t-butyl-m-cresyl)-
Structure	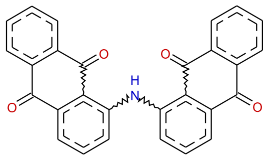	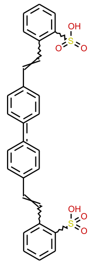	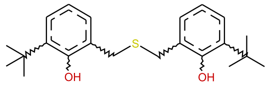
Actual Endpoint	Irritant	Irritant	Irritant
Predicted Endpoint	Non-Irritant	Non-Irritant	Irritant
Distance	0.777	0.777	0.837
Reference	85JCAE "Prehled Prumyslove Toxikologie; Organicke Latky," Marhold, J., Prague , Czechoslovakia, Avicenum, 1986 Volume(issue)/page/year: -,735,1986	MVCRB3 MVC-Report. (Stockholm, Sweden) No.1-2, 1972-73. Discontinued. Volu me(issue)/page/year: 2,193,1973	AMIHBC AMA Archives of Industrial Hygiene and Occupational Medicine. (Chicago , IL) V.2-10, 1950-54. For publisher information, see AEHLAU. Volume(issue)/pag e/year: 5,311,1952

Model Applicability

Unknown features are fingerprint features in the query molecule, but not found or appearing too infrequently in the training set.

1. All properties and OPS components are within expected ranges.
2. Unknown FCFP_2 feature: 179977000: [*][c]1:[*]:[*]:n:n:1[c](:[*]):[*]

Feature Contribution

Top features for positive contribution

Fingerprint	Bit/Smiles	Feature Structure	Score	Irritant in training set

FCFP_12	-124655670	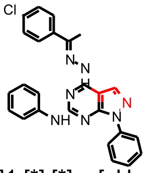 [*][c]1:[*]:[*]:n:[cH]:1	0.0821	13 out of 13
FCFP_12	4427049	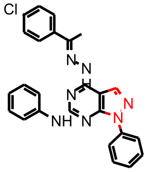 [*]n1:[*]:[*]:[cH]:n:1	0.0734	5 out of 5
FCFP_12	-149636017	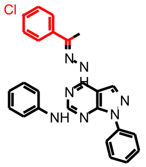 [*]=C[c]1:[cH]:[cH]:[c](Cl):[cH]:[cH]:1	0.0703	4 out of 4
**Top Features for negative contribution**				
**Fingerprint**	**Bit/Smiles**	**Feature Structure**	**Score**	**Irritant in training set**
FCFP_12	411414971	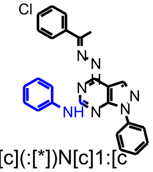 [*]:[c](:[*])N[c]1:[cH]:[cH]:[cH]:[cH]:[cH]:1	-1.31	1 out of 7
FCFP_12	839741273	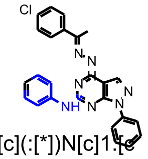 [*]:[c](:[*])N[c]1:[cH]:[cH]:[*]:[cH]:[cH]:1	-0.708	4 out of 10

FCFP_12	-1724769936	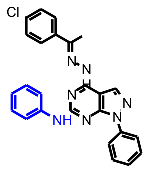 [*]N[c]1:[cH]:[cH]:[cH]:[cH]:[cH]:1	-0.475	11 out of 20

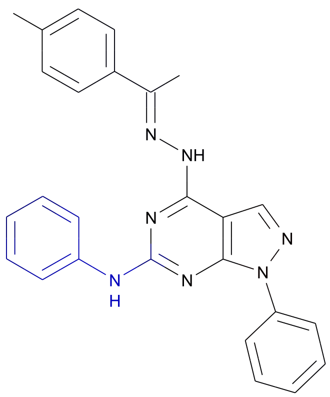

$C_{26}H_{23}N_7$
Molecular Weight: 433.50772
ALogP: 6.372
Rotatable Bonds: 6
Acceptors: 6
Donors: 2

Model Prediction

Prediction: Non-Irritant
Probability: 0.597
Enrichment: 0.648
Bayesian Score: -4.28
Mahalanobis Distance: 7.51
Mahalanobis Distance p-value: 0.972

Prediction: Positive if the Bayesian score is above the estimated best cutoff value from minimizing the false positive and false negative rate.
Probability: The esimated probability that the sample is in the positive category. This assumes that the Bayesian score follows a normal distribution and is different from the prediction using a cutoff.
Enrichment: An estimate of enrichment, that is, the increased likelihood (versus random) of this sample being in the category.
Bayesian Score: The standard Laplacian-modified Bayesian score.
Mahalanobis Distance: The Mahalanobis distance (MD) is the distance to the center of the training data. The larger the MD, the less trustworthy the prediction.
Mahalanobis Distance p-value: The p-value gives the fraction of training data with an MD greater than or equal to the one for the given sample, assuming normally distributed data. The smaller the p-value, the less trustworthy the prediciton. For highly non-normal X properties (e.g., fingerprints), the MD p-value is wildly inaccurate.

Structural Similar Compounds

Name	Anthraquinone, 1,1'-iminodi-	Benzenesulfonic acid, 2,2'-(4,4'-biphenylylenedivinylene)di-, disod ium salt	Sulfide, bis(4-t-butyl-m-cresyl)-
Structure	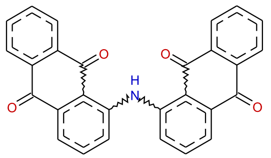	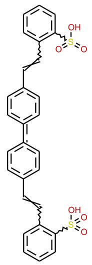	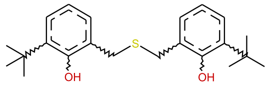
Actual Endpoint	Irritant	Irritant	Irritant
Predicted Endpoint	Non-Irritant	Non-Irritant	Irritant
Distance	0.766	0.796	0.814
Reference	85JCAE "Prehled Prumyslove Toxikologie; Organicke Latky," Marhold, J., Prague , Czechoslovakia, Avicenum, 1986 Volume(issue)/page/year: -,735,1986	MVCRB3 MVC-Report. (Stockholm, Sweden) No.1-2, 1972-73. Discontinued. Volu me(issue)/page/year: 2,193,1973	AMIHBC AMA Archives of Industrial Hygiene and Occupational Medicine. (Chicago , IL) V.2-10, 1950-54. For publisher information, see AEHLAU. Volume(issue)/pag e/year: 5,311,1952

Model Applicability

Unknown features are fingerprint features in the query molecule, but not found or appearing too infrequently in the training set.

1. All properties and OPS components are within expected ranges.
2. Unknown FCFP_2 feature: 179977000: [*][c]1:[*]:[*]:n:n:1[c](:[*]):[*]

Feature Contribution

Top features for positive contribution

Fingerprint	Bit/Smiles	Feature Structure	Score	Irritant in training set

FCFP_12	-124655670	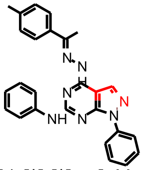 [*][c]1:[*]:[*]:n:[cH]:1	0.0821	13 out of 13
FCFP_12	4427049	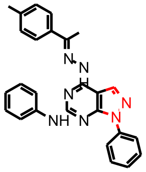 [*]n1:[*]:[*]:[cH]:n:1	0.0734	5 out of 5
FCFP_12	1551257511	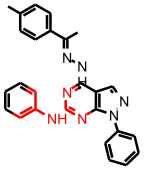 [*]:[cH]:[c](N[c](:n:[*]):n:[*]):[cH]:[*]	0.0658	3 out of 3
**Top Features for negative contribution**				
**Fingerprint**	**Bit/Smiles**	**Feature Structure**	**Score**	**Irritant in training set**
FCFP_12	411414971	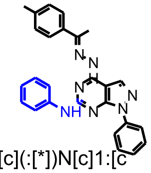 [*]:[c](:[*])N[c]1:[cH]:[cH]:[cH]:[cH]:[cH]:1	-1.31	1 out of 7
FCFP_12	839741273	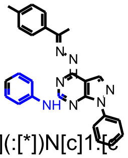 [*]:[c](:[*])N[c]1:[cH]:[cH]:[*]:[cH]:[cH]:1	-0.708	4 out of 10

FCFP_12	-1724769936	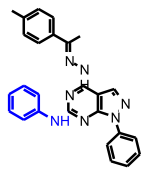 [*]N[c]1:[cH]:[cH]:[cH]:[cH]:1	-0.475	11 out of 20

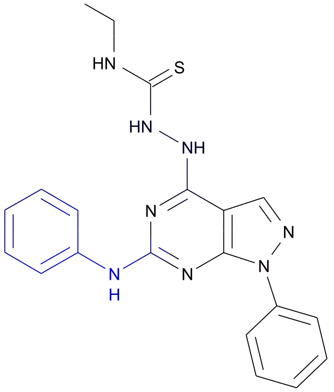

$C_{20}H_{20}N_8S$

Molecular Weight: 404.4914

ALogP: 4.99

Rotatable Bonds: 8

Acceptors: 6

Donors: 4

Model Prediction

Prediction: Non-Irritant

Probability: 0.489

Enrichment: 0.531

Bayesian Score: -4.58

Mahalanobis Distance: 8.97

Mahalanobis Distance p-value: 0.439

Prediction: Positive if the Bayesian score is above the estimated best cutoff value from minimizing the false positive and false negative rate.
Probability: The esimated probability that the sample is in the positive category. This assumes that the Bayesian score follows a normal distribution and is different from the prediction using a cutoff.
Enrichment: An estimate of enrichment, that is, the increased likelihood (versus random) of this sample being in the category.
Bayesian Score: The standard Laplacian-modified Bayesian score.
Mahalanobis Distance: The Mahalanobis distance (MD) is the distance to the center of the training data. The larger the MD, the less trustworthy the prediction.
Mahalanobis Distance p-value: The p-value gives the fraction of training data with an MD greater than or equal to the one for the given sample, assuming normally distributed data. The smaller the p-value, the less trustworthy the prediciton. For highly non-normal X properties (e.g., fingerprints), the MD p-value is wildly inaccurate.

Structural Similar Compounds

Name	Benzenesulfonic acid, 2,2'-(4,4'-biphenylylenedivinylene)di-, disod ium salt	Urea, 1,3-bis(2-benzothiazolylthiomethyl)-	Anthraquinone, 2-bromo-1,8-diamino-4,5-dihydroxy-
Structure	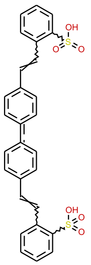	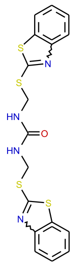	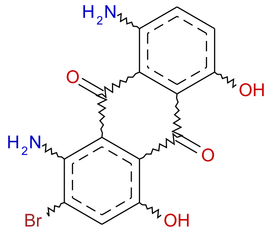
Actual Endpoint	Irritant	Irritant	Irritant
Predicted Endpoint	Non-Irritant	Irritant	Non-Irritant
Distance	0.960	0.995	0.998
Reference	MVCRB3 MVC-Report. (Stockholm, Sweden) No.1-2, 1972-73. Discontinued. Volu me(issue)/page/year: 2,193,1973	AMIHBC AMA Archives of Industrial Hygiene and Occupational Medicine. (Chicago , IL) V.2-10, 1950-54. For publisher information, see AEHLAU. Volume(issue)/pag e/year: 5,311,1952	28ZPAK "Sbornik Vysledku Toxixologickeho Vysetreni Latek A Pripravku," Marhol d, J.V., Institut Pro Vychovu Vedoucicn Pracovniku Chemickeho Prumyclu Praha, Cz echoslovakia, 1972 Volume(issue)/page/year: -,244,1

Model Applicability

Unknown features are fingerprint features in the query molecule, but not found or appearing too infrequently in the training set.

1. All properties and OPS components are within expected ranges.
2. Unknown FCFP_2 feature: 179977000: [*][c]1:[*]:[*]:n:n:1[c](:[*]):[*]

Feature Contribution

Top features for positive contribution				
Fingerprint	Bit/Smiles	Feature Structure	Score	Irritant in training set

FCFP_12	-124655670	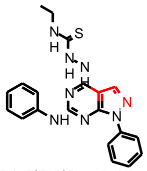<br>[*][c]1:[*]:[*]:n:[cH<br>]:1	0.0821	13 out of 13
FCFP_12	4427049	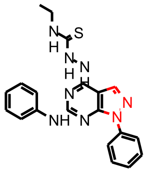<br>[*]n1:[*]:[*]:[cH]:n:<br>1	0.0734	5 out of 5
FCFP_12	1499521844	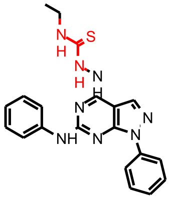<br>[*]NC(=N[*])N[*]	0.0658	3 out of 3
**Top Features for negative contribution**				
**Fingerprint**	**Bit/Smiles**	**Feature Structure**	**Score**	**Irritant in training set**
FCFP_12	411414971	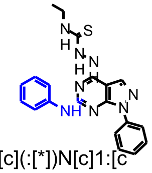<br>[*]:[c](:[*])N[c]1:[c<br>H]:[cH]:[cH]:[cH]:[c<br>H]:1	-1.31	1 out of 7
FCFP_12	839741273	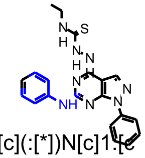<br>[*]:[c](:[*])N[c]1:[c<br>H]:[cH]:[*]:[cH]:[cH<br>]:1	-0.708	4 out of 10

FCFP_12	1294344583	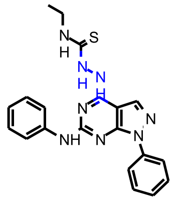 [*]:[c](:[*])NN	-0.65	0 out of 1

TOPKAT_Skin_Irritancy_None_vs_Irritant

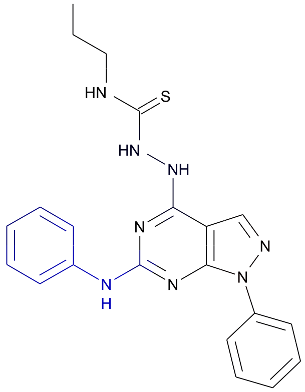

$C_{21}H_{22}N_8S$
Molecular Weight: 418.51798
ALogP: 5.514
Rotatable Bonds: 9
Acceptors: 6
Donors: 4

Structural Similar Compounds

Name	Benzenesulfonic acid, 2,2'-(4,4'-biphenylylenedivinylene)di-, disod ium salt	Urea, 1,3-bis(2-benzothiazolylthiomethyl)-	2-Anthracenesulfonic acid, 1-amino-9,10-dihydro-9,10-dioxo-4-(2,4,6 -trimethylanilino)-, monosodium salt
Structure	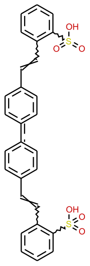	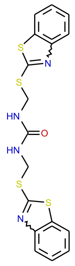	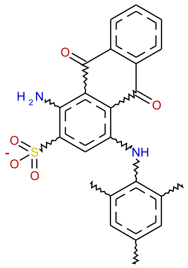
Actual Endpoint	Irritant	Irritant	Irritant
Predicted Endpoint	Non-Irritant	Irritant	Non-Irritant
Distance	0.945	1.013	1.064
Reference	MVCRB3 MVC-Report. (Stockholm, Sweden) No.1-2, 1972-73. Discontinued. Volu me(issue)/page/year: 2,193,1973	AMIHBC AMA Archives of Industrial Hygiene and Occupational Medicine. (Chicago , IL) V.2-10, 1950-54. For publisher information, see AEHLAU. Volume(issue)/pag e/year: 5,311,1952	85JCAE "Prehled Prumyslove Toxikologie; Organicke Latky," Marhold, J., Prague , Czechoslovakia, Avicenum, 1986 Volume(issue)/page/year: -,1327,1986

Model Prediction

Prediction: Non-Irritant
Probability: 0.443
Enrichment: 0.481
Bayesian Score: -4.7
Mahalanobis Distance: 9.48
Mahalanobis Distance p-value: 0.198

Prediction: Positive if the Bayesian score is above the estimated best cutoff value from minimizing the false positive and false negative rate.
Probability: The esimated probability that the sample is in the positive category. This assumes that the Bayesian score follows a normal distribution and is different from the prediction using a cutoff.
Enrichment: An estimate of enrichment, that is, the increased likelihood (versus random) of this sample being in the category.
Bayesian Score: The standard Laplacian-modified Bayesian score.
Mahalanobis Distance: The Mahalanobis distance (MD) is the distance to the center of the training data. The larger the MD, the less trustworthy the prediction.
Mahalanobis Distance p-value: The p-value gives the fraction of training data with an MD greater than or equal to the one for the given sample, assuming normally distributed data. The smaller the p-value, the less trustworthy the prediciton. For highly non-normal X properties (e.g., fingerprints), the MD p-value is wildly inaccurate.

Model Applicability

Unknown features are fingerprint features in the query molecule, but not found or appearing too infrequently in the training set.

1. All properties and OPS components are within expected ranges.
2. Unknown FCFP_2 feature: 179977000: [*][c]1:[*]:[*]:n:n:1[c](:[*]):[*]

Feature Contribution

Top features for positive contribution

Fingerprint	Bit/Smiles	Feature Structure	Score	Irritant in training set

FCFP_12	-124655670	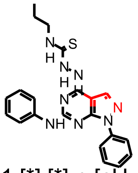 [*][c]1:[*]:[*]:n:[cH]:1	0.0821	13 out of 13
FCFP_12	4427049	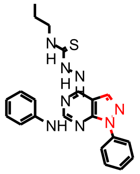 [*]n1:[*]:[*]:[cH]:n:1	0.0734	5 out of 5
FCFP_12	1499521844	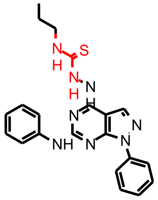 [*]NC(=N[*])N[*]	0.0658	3 out of 3

Top Features for negative contribution

Fingerprint	Bit/Smiles	Feature Structure	Score	Irritant in training set
FCFP_12	411414971	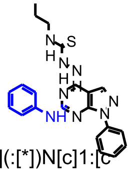 [*]:[c](:[*])N[c]1:[cH]:[cH]:[cH]:[cH]:[cH]:1	-1.31	1 out of 7
FCFP_12	839741273	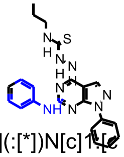 [*]:[c](:[*])N[c]1:[cH]:[cH]:[*]:[cH]:[cH]:1	-0.708	4 out of 10

FCFP_12	1294344583	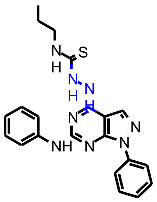 [*]:[c](:[*])NN	-0.65	0 out of 1

Erlotinib

TOPKAT_Skin_Irritancy_None_vs_Irritant

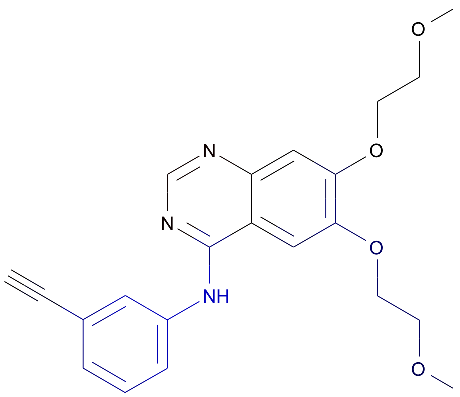

$C_{22}H_{23}N_3O_4$

Molecular Weight: 393.43572

ALogP: 4.309

Rotatable Bonds: 10

Acceptors: 7

Donors: 1

Model Prediction

Prediction: Non-Irritant

Probability: 0.119

Enrichment: 0.13

Bayesian Score: -5.76

Mahalanobis Distance: 10.2

Mahalanobis Distance p-value: 0.0333

Prediction: Positive if the Bayesian score is above the estimated best cutoff value from minimizing the false positive and false negative rate.
Probability: The esimated probability that the sample is in the positive category. This assumes that the Bayesian score follows a normal distribution and is different from the prediction using a cutoff.
Enrichment: An estimate of enrichment, that is, the increased likelihood (versus random) of this sample being in the category.
Bayesian Score: The standard Laplacian-modified Bayesian score.
Mahalanobis Distance: The Mahalanobis distance (MD) is the distance to the center of the training data. The larger the MD, the less trustworthy the prediction.
Mahalanobis Distance p-value: The p-value gives the fraction of training data with an MD greater than or equal to the one for the given sample, assuming normally distributed data. The smaller the p-value, the less trustworthy the prediciton. For highly non-normal X properties (e.g., fingerprints), the MD p-value is wildly inaccurate.

Structural Similar Compounds

Name	Propanoic acid, 2-(4-((5-(trifluoromethyl)-2-pyridinyl)oxy)phenoxy) -, butyl ester	Carbamic acid, ((dibutylamino)thio)methyl-, 2,2-dimethyl-2,3-dihydro-7-benzofuranyl ester	1,4-Pentadien-3-one, 1,5-bis(p-azidophenyl)-
Structure	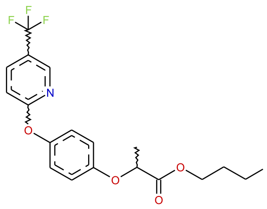	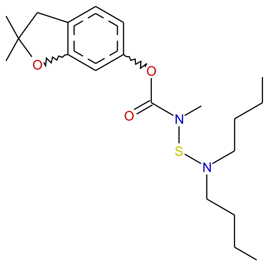	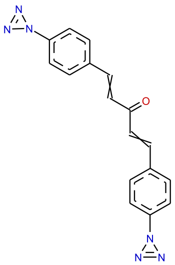
Actual Endpoint	Irritant	Irritant	Irritant
Predicted Endpoint	Irritant	Irritant	Irritant
Distance	0.776	0.777	0.803
Reference	NNGADV Nippon Noyaku Gakkaishi. Journal of the Pesticide Science Society of Japan. (Nippon Noyaku Gakkai, 1-43-11, Komagome, Toshima-ku, Tokyo 170, Japan) V.1- 1976- Volume(issue)/page/year: 15,305,1990	NTIS** National Technical Information Service. (Springfield, VA 22161) Forme rly U.S. Clearinghouse for Scientific & Technical Information. Volume(issue)/pag e/year: OTS0539690	85JCAE "Prehled Prumyslove Toxikologie; Organicke Latky," Marhold, J., Prague , Czechoslovakia, Avicenum, 1986 Volume(issue)/page/year: -,733,1986

Model Applicability

Unknown features are fingerprint features in the query molecule, but not found or appearing too infrequently in the training set.

1. All properties and OPS components are within expected ranges.
2. Unknown FCFP_2 feature: 902193919: [*]:[c](:[*])C#C

Feature Contribution

Top features for positive contribution				
Fingerprint	Bit/Smiles	Feature Structure	Score	Irritant in training set

FCFP_12	-124685461	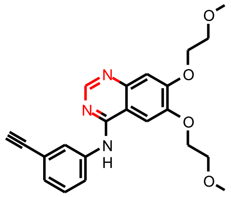<br>[*]:n:[cH]:n:[*]	0.0734	5 out of 5
FCFP_12	-475316933	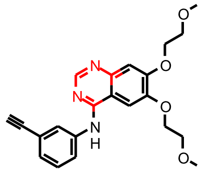<br>[*][c]1:[*]:[c](:[*])<br>:n:[cH]:n:1	0.0703	4 out of 4
FCFP_12	131784192	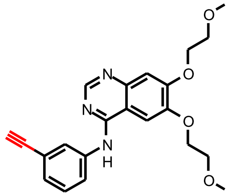<br>[*]C#C	0.0658	3 out of 3
**Top Features for negative contribution**				
**Fingerprint**	**Bit/Smiles**	**Feature Structure**	**Score**	**Irritant in training set**
FCFP_12	411414971	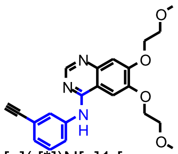<br>[*]:[c](:[*])N[c]1:[c<br>H]:[cH]:[cH]:[cH]:[c<br>H]:1	-1.31	1 out of 7
FCFP_12	-1059904848	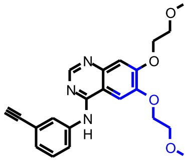<br>[*][c](:[*]):[c](OCCO<br>C):[cH]:[*]	-1.04	0 out of 2

FCFP_12	839741273	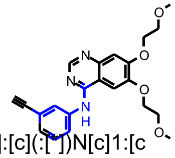 [*]:[c](:[*])N[c]1:[cH]:[cH]:[*]:[cH]:[cH]:1	-0.708	4 out of 10

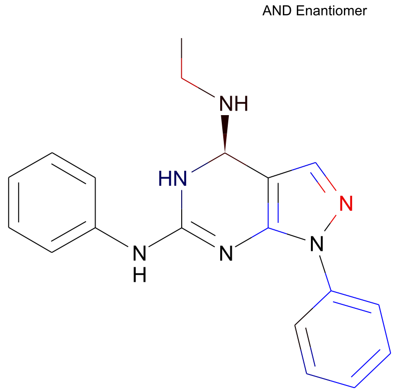

$C_{19}H_{20}N_6$

Molecular Weight: 332.4023

ALogP: 3.039

Rotatable Bonds: 5

Acceptors: 5

Donors: 3

Model Prediction

Prediction: 5.48

Unit: mg/kg_body_weight/day

Mahalanobis Distance: 11.6

Mahalanobis Distance p-value: 0.00015

Mahalanobis Distance: The Mahalanobis distance (MD) is a generalization of the Euclidean distance that accounts for correlations among the X properties. It is calculated as the distance to the center of the training data. The larger the MD, the less trustworthy the prediction.
Mahalanobis Distance p-value: The p-value gives the fraction of training data with an MD greater than or equal to the one for the given sample, assuming normally distributed data. The smaller the p-value, the less trustworthy the prediciton. For highly non-normal X properties (e.g., fingerprints), the MD p-value is wildly inaccurate.

Structural Similar Compounds

Name	Phenolphthalein	693	Oxazepam
Structure	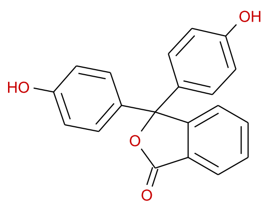	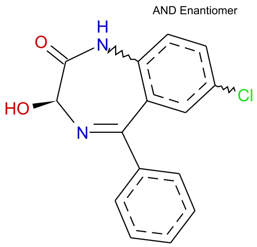	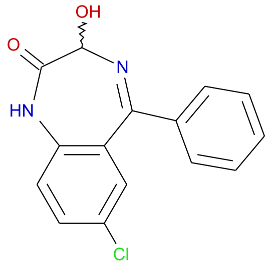
Actual Endpoint (-log C)	2.43468	3.90356	3.90356
Predicted Endpoint (-log C)	3.66084	3.39677	3.39677
Distance	0.690	0.783	0.783
Reference	CPDB	CPDB	CPDB

Model Applicability

Unknown features are fingerprint features in the query molecule, but not found or appearing too infrequently in the training set.

1. All properties and OPS components are within expected ranges.
2. Unknown ECFP_2 feature: -961383061: [*]n1:[*]:[*]:[c]([*]):[c]:1N=[*]
3. Unknown ECFP_2 feature: -676555381: [*]n1:[*]:[*]:c:n:1
4. Unknown ECFP_2 feature: 1001947487: [*][c]1:[*]:[*]:n:n:1[c](:[*]):[*]
5. Unknown ECFP_2 feature: -24640926: [*]NC(=N[*])N[*]
6. Unknown ECFP_2 feature: 2072098278: [*]NC(N[*])[c](:[*]):[*]
7. Unknown ECFP_2 feature: 496787418: [*]CNC([*])[*]
8. Unknown ECFP_2 feature: -174914108: [*]:n(:[*])[c](:c:[*]):c:[*]

Feature Contribution

Top features for positive contribution

Fingerprint	Bit/Smiles	Feature Structure	Score

ECFP_6	655739385	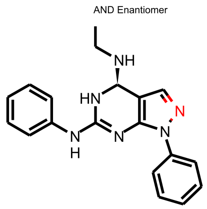 [*]:n:[*]	0.229
ECFP_6	1559650422	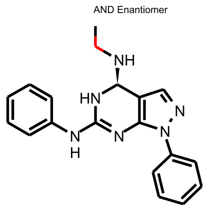 [*]C[*]	0.203
ECFP_6	-167460056	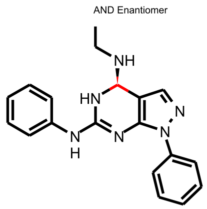 [*]C([*])[*]	0.0596
**Top Features for negative contribution**			
**Fingerprint**	**Bit/Smiles**	**Feature Structure**	**Score**
ECFP_6	1996767644	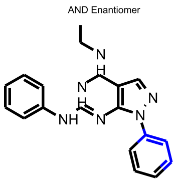 [*][c](:[*]):[cH]:[cH]:[*]	-0.251
ECFP_6	642810091	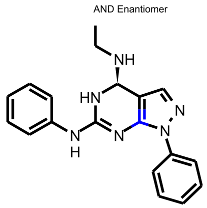 [*][c](:[*]):[*]	-0.247

ECFP_6	-182236392	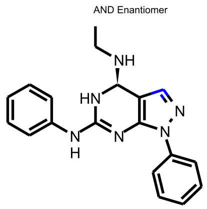<br>[*]:[cH]:[*]	-0.232

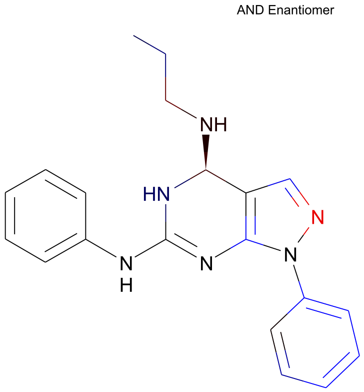

$C_{20}H_{22}N_6$

Molecular Weight: 346.42888

ALogP: 3.562

Rotatable Bonds: 6

Acceptors: 5

Donors: 3

Model Prediction

Prediction: 7

Unit: mg/kg_body_weight/day

Mahalanobis Distance: 11.7

Mahalanobis Distance p-value: 9.9e-005

Mahalanobis Distance: The Mahalanobis distance (MD) is a generalization of the Euclidean distance that accounts for correlations among the X properties. It is calculated as the distance to the center of the training data. The larger the MD, the less trustworthy the prediction.
Mahalanobis Distance p-value: The p-value gives the fraction of training data with an MD greater than or equal to the one for the given sample, assuming normally distributed data. The smaller the p-value, the less trustworthy the prediciton. For highly non-normal X properties (e.g., fingerprints), the MD p-value is wildly inaccurate.

Structural Similar Compounds

Name	Phenolphthalein	542	Ochratoxin A
Structure	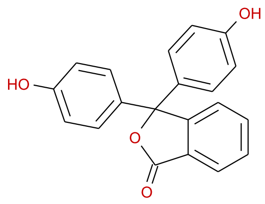	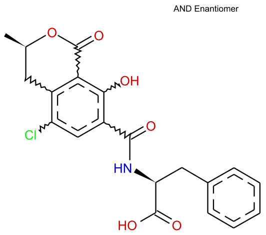	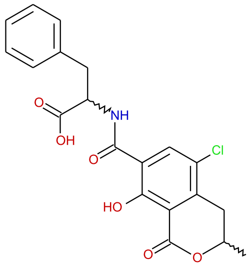
Actual Endpoint (-log C)	2.43468	4.79932	4.79932
Predicted Endpoint (-log C)	3.66084	3.6353	3.6353
Distance	0.719	0.786	0.786
Reference	CPDB	CPDB	CPDB

Model Applicability

Unknown features are fingerprint features in the query molecule, but not found or appearing too infrequently in the training set.

1. All properties and OPS components are within expected ranges.
2. Unknown ECFP_2 feature: -961383061: [*]n1:[*]:[*]:[c]([*]):[c]:1N=[*]
3. Unknown ECFP_2 feature: -676555381: [*]n1:[*]:[*]:c:n:1
4. Unknown ECFP_2 feature: 1001947487: [*][c]1:[*]:[*]:n:n:1[c](:[*]):[*]
5. Unknown ECFP_2 feature: -24640926: [*]NC(=N[*])N[*]
6. Unknown ECFP_2 feature: 2072098278: [*]NC(N[*])[c](:[*]):[*]
7. Unknown ECFP_2 feature: 496787418: [*]CNC([*])[*]
8. Unknown ECFP_2 feature: -174914108: [*]:n(:[*])[c](:c:[*]):c:[*]

Feature Contribution

Top features for positive contribution

Fingerprint	Bit/Smiles	Feature Structure	Score

ECFP_6	655739385	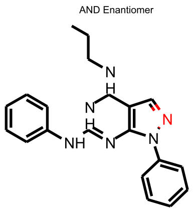 [*]:n:[*]	0.229
ECFP_6	1559650422	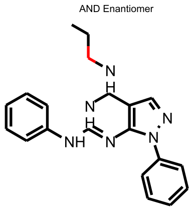 [*]C[*]	0.203
ECFP_6	-167460056	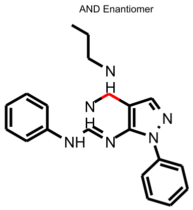 [*]C([*])[*]	0.0596
**Top Features for negative contribution**			
**Fingerprint**	**Bit/Smiles**	**Feature Structure**	**Score**
ECFP_6	1996767644	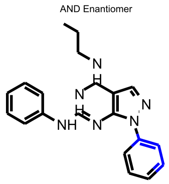 [*][c](:[*]):[cH]:[cH]:[*]	-0.251
ECFP_6	642810091	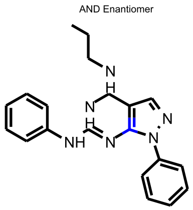 [*][c](:[*]):[*]	-0.247

ECFP_6	-182236392	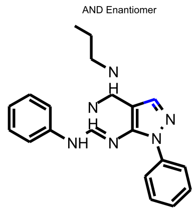 [*]:[cH]:[*]	-0.232

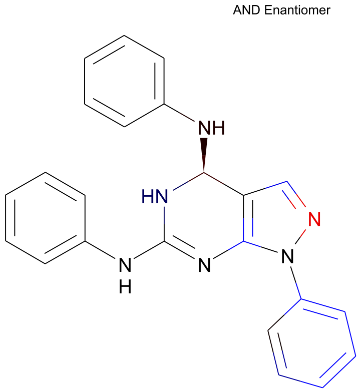

$C_{23}H_{20}N_6$
Molecular Weight: 380.4451
ALogP: 4.544
Rotatable Bonds: 5
Acceptors: 5
Donors: 3

Model Prediction

Prediction: 2.96
Unit: mg/kg_body_weight/day
Mahalanobis Distance: 12.4
Mahalanobis Distance p-value: 2.43e-006

Mahalanobis Distance: The Mahalanobis distance (MD) is a generalization of the Euclidean distance that accounts for correlations among the X properties. It is calculated as the distance to the center of the training data. The larger the MD, the less trustworthy the prediction.
Mahalanobis Distance p-value: The p-value gives the fraction of training data with an MD greater than or equal to the one for the given sample, assuming normally distributed data. The smaller the p-value, the less trustworthy the prediciton. For highly non-normal X properties (e.g., fingerprints), the MD p-value is wildly inaccurate.

Structural Similar Compounds

Name	Phenolphthalein	646	542
Structure	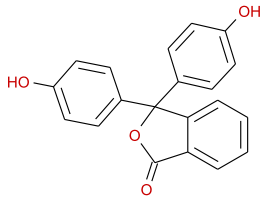	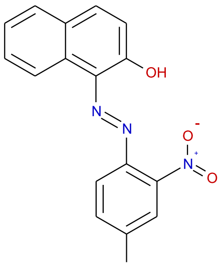	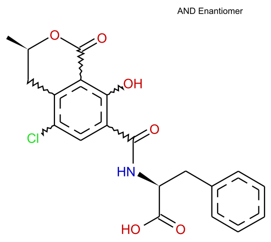
Actual Endpoint (-log C)	2.43468	0.937339	4.79932
Predicted Endpoint (-log C)	3.66084	3.26294	3.6353
Distance	0.767	0.888	0.935
Reference	CPDB	CPDB	CPDB

Model Applicability

Unknown features are fingerprint features in the query molecule, but not found or appearing too infrequently in the training set.

1. All properties and OPS components are within expected ranges.
2. Unknown ECFP_2 feature: -961383061: [*]n1:[*]:[*]:[c]([*]):[c]:1N=[*]
3. Unknown ECFP_2 feature: -676555381: [*]n1:[*]:[*]:c:n:1
4. Unknown ECFP_2 feature: 1001947487: [*][c]1:[*]:[*]:n:n:1[c](:[*]):[*]
5. Unknown ECFP_2 feature: -24640926: [*]NC(=N[*])N[*]
6. Unknown ECFP_2 feature: 2072098278: [*]NC(N[*])[c](:[*]):[*]
7. Unknown ECFP_2 feature: -1237219435: [*]C([*])N[c](:[*]):[*]
8. Unknown ECFP_2 feature: -174914108: [*]:n(:[*])[c](:c:[*]):c:[*]

Feature Contribution

Top features for positive contribution			
**Fingerprint**	**Bit/Smiles**	**Feature Structure**	**Score**

ECFP_6	655739385	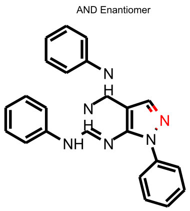 [*]:n:[*]	0.229
ECFP_6	-167460056	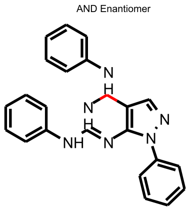 [*]C([*])[*]	0.0596
ECFP_6	-1897341097	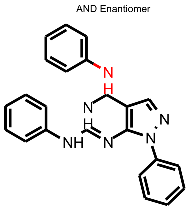 [*]N[*]	0.0284

Top Features for negative contribution

Fingerprint	Bit/Smiles	Feature Structure	Score
ECFP_6	1996767644	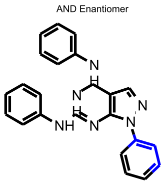 [*][c](:[*]):[cH]:[cH]:[*]	-0.251
ECFP_6	642810091	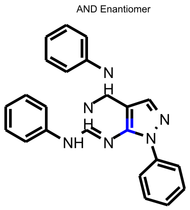 [*][c](:[*]):[*]	-0.247

ECFP_6	-182236392	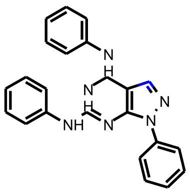 [*]:[cH]:[*]	-0.232

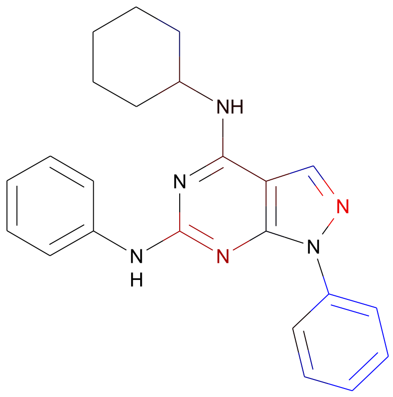

$C_{23}H_{24}N_6$

Molecular Weight: 384.47686

ALogP: 5.953

Rotatable Bonds: 5

Acceptors: 5

Donors: 2

Model Prediction

Prediction: 1.84

Unit: mg/kg_body_weight/day

Mahalanobis Distance: 12

Mahalanobis Distance p-value: 1.85e-005

Mahalanobis Distance: The Mahalanobis distance (MD) is a generalization of the Euclidean distance that accounts for correlations among the X properties. It is calculated as the distance to the center of the training data. The larger the MD, the less trustworthy the prediction.
Mahalanobis Distance p-value: The p-value gives the fraction of training data with an MD greater than or equal to the one for the given sample, assuming normally distributed data. The smaller the p-value, the less trustworthy the prediciton. For highly non-normal X properties (e.g., fingerprints), the MD p-value is wildly inaccurate.

Structural Similar Compounds

Name	Phenolphthalein	646	C.I. vat yellow 4
Structure	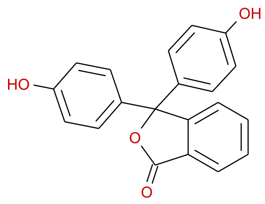	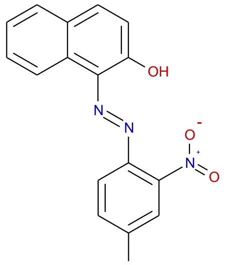	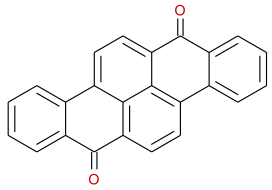
Actual Endpoint (-log C)	2.43468	0.937339	1.48417
Predicted Endpoint (-log C)	3.66084	3.26294	4.45029
Distance	0.773	0.825	0.912
Reference	CPDB	CPDB	CPDB

Model Applicability

Unknown features are fingerprint features in the query molecule, but not found or appearing too infrequently in the training set.

1. All properties and OPS components are within expected ranges.
2. Unknown ECFP_2 feature: -676555381: [*]n1:[*]:[*]:c:n:1
3. Unknown ECFP_2 feature: 1001947487: [*][c]1:[*]:[*]:n:n:1[c](:[*]):[*]
4. Unknown ECFP_2 feature: -1237219435: [*]C([*])N[c](:[*]):[*]
5. Unknown ECFP_2 feature: -174914108: [*]:n(:[*])[c](:c:[*]):c:[*]

Feature Contribution

Top features for positive contribution

Fingerprint	Bit/Smiles	Feature Structure	Score
ECFP_6	655739385	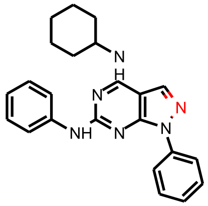 [*]:n:[*]	0.229

ECFP_6	834876373	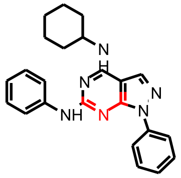 [*][c](:[*]):n:[c](:[*]):[*]	0.163
ECFP_6	1333660716	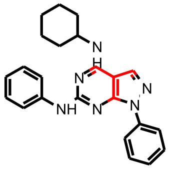 [*][c](:[*]):[c]1:[cH]:[*]:[*]:[c]:1:[*]	0.0746
**Top Features for negative contribution**			
**Fingerprint**	**Bit/Smiles**	**Feature Structure**	**Score**
ECFP_6	1996767644	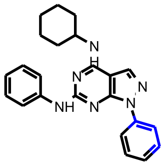 [*][c](:[*]):[cH]:[cH]:[*]	-0.251
ECFP_6	642810091	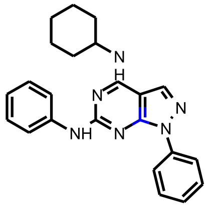 [*][c](:[*]):[*]	-0.247
ECFP_6	-182236392	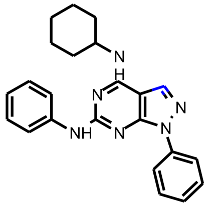 [*]:[cH]:[*]	-0.232

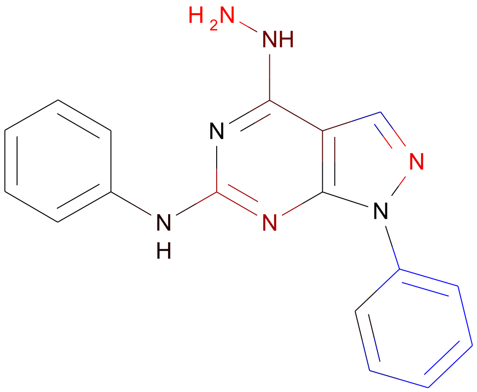

$C_{17}H_{15}N_7$
Molecular Weight: 317.3479
ALogP: 3.562
Rotatable Bonds: 4
Acceptors: 6
Donors: 3

Model Prediction

Prediction: 2.01
Unit: mg/kg_body_weight/day
Mahalanobis Distance: 10.7
Mahalanobis Distance p-value: 0.00432

Mahalanobis Distance: The Mahalanobis distance (MD) is a generalization of the Euclidean distance that accounts for correlations among the X properties. It is calculated as the distance to the center of the training data. The larger the MD, the less trustworthy the prediction.
Mahalanobis Distance p-value: The p-value gives the fraction of training data with an MD greater than or equal to the one for the given sample, assuming normally distributed data. The smaller the p-value, the less trustworthy the prediciton. For highly non-normal X properties (e.g., fingerprints), the MD p-value is wildly inaccurate.

Structural Similar Compounds

Name	Phenolphthalein	Triamterene	646
Structure	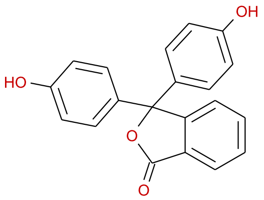	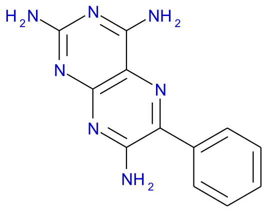	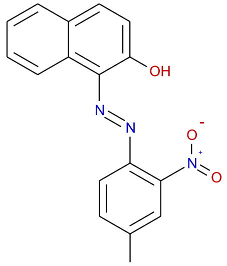
Actual Endpoint (-log C)	2.43468	3.62397	0.937339
Predicted Endpoint (-log C)	3.66084	4.35116	3.26294
Distance	0.757	0.814	0.816
Reference	CPDB	CPDB	CPDB

Model Applicability

Unknown features are fingerprint features in the query molecule, but not found or appearing too infrequently in the training set.

1. OPS PC16 out of range. Value: 4.1901. Training min, max, SD, explained variance: -3.1026, 4.016, 1.245, 0.0193.
2. Unknown ECFP_2 feature: -676555381: [*]n1:[*]:[*]:c:n:1
3. Unknown ECFP_2 feature: 1001947487: [*][c]1:[*]:[*]:n:n:1[c](:[*]):[*]
4. Unknown ECFP_2 feature: -174914108: [*]:n(:[*])[c](:c:[*]):c:[*]

Feature Contribution

Top features for positive contribution

Fingerprint	Bit/Smiles	Feature Structure	Score
ECFP_6	655739385	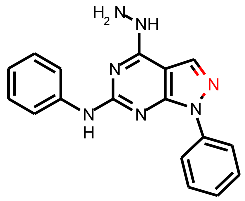 [*]:n:[*]	0.229

ECFP_6	1572579716	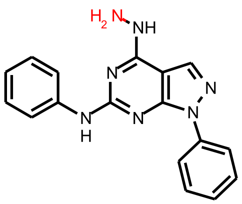 [*]N	0.225
ECFP_6	834876373	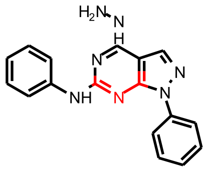 [*][c](:[*]):n:[c](:[*]):[*]	0.163

Top Features for negative contribution

Fingerprint	Bit/Smiles	Feature Structure	Score
ECFP_6	1996767644	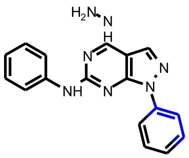 [*][c](:[*]):[cH]:[cH]:[*]	-0.251
ECFP_6	642810091	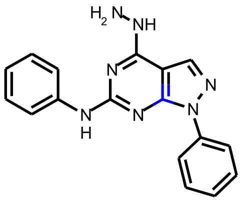 [*][c](:[*]):[*]	-0.247
ECFP_6	-182236392	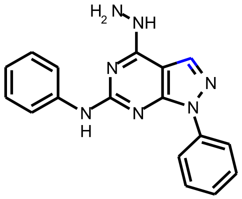 [*]:[cH]:[*]	-0.232

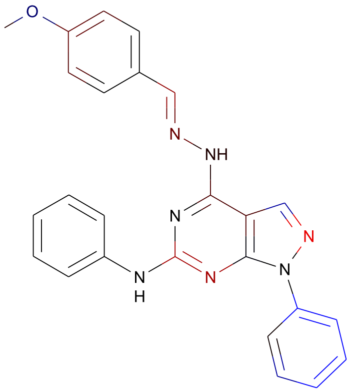

$C_{25}H_{21}N_7O$
Molecular Weight: 435.48054
ALogP: 5.889
Rotatable Bonds: 7
Acceptors: 7
Donors: 2

Model Prediction

Prediction: 1.21
Unit: mg/kg_body_weight/day
Mahalanobis Distance: 14.2
Mahalanobis Distance p-value: 7.96e-011

Mahalanobis Distance: The Mahalanobis distance (MD) is a generalization of the Euclidean distance that accounts for correlations among the X properties. It is calculated as the distance to the center of the training data. The larger the MD, the less trustworthy the prediction.
Mahalanobis Distance p-value: The p-value gives the fraction of training data with an MD greater than or equal to the one for the given sample, assuming normally distributed data. The smaller the p-value, the less trustworthy the prediciton. For highly non-normal X properties (e.g., fingerprints), the MD p-value is wildly inaccurate.

Structural Similar Compounds

Name	646	223	Phenolphthalein
Structure	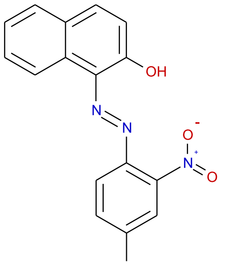	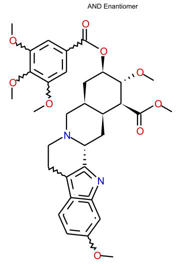	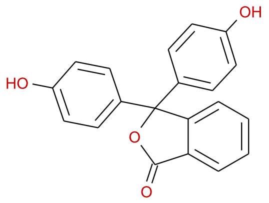
Actual Endpoint (-log C)	0.937339	5.08368	2.43468
Predicted Endpoint (-log C)	3.26294	5.08273	3.66084
Distance	0.965	0.968	0.978
Reference	CPDB	CPDB	CPDB

Model Applicability

Unknown features are fingerprint features in the query molecule, but not found or appearing too infrequently in the training set.

1. OPS PC16 out of range. Value: 4.2838. Training min, max, SD, explained variance: -3.1026, 4.016, 1.245, 0.0193.
2. Unknown ECFP_2 feature: -676555381: [*]n1:[*]:[*]:c:n:1
3. Unknown ECFP_2 feature: 1001947487: [*][c]1:[*]:[*]:n:n:1[c](:[*]):[*]
4. Unknown ECFP_2 feature: -174914108: [*]:n(:[*])[c](:c:[*]):c:[*]

Feature Contribution

Top features for positive contribution

Fingerprint	Bit/Smiles	Feature Structure	Score
ECFP_6	655739385	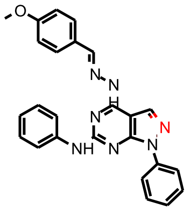 [*]:n:[*]	0.229

ECFP_6	834876373	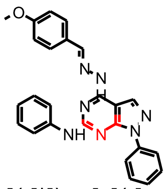 [*][c](:[*]):n:[c](:[*]):[*]	0.163
ECFP_6	-1925046727	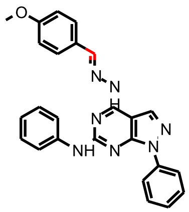 [*]C=[*]	0.145
**Top Features for negative contribution**			
**Fingerprint**	**Bit/Smiles**	**Feature Structure**	**Score**
ECFP_6	1996767644	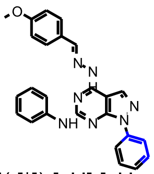 [*][c](:[*]):[cH]:[cH]:[*]	-0.251
ECFP_6	642810091	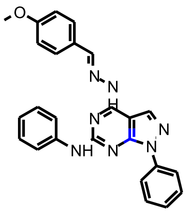 [*][c](:[*]):[*]	-0.247
ECFP_6	-182236392	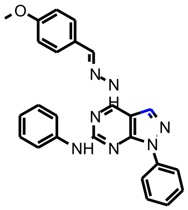 [*]:[cH]:[*]	-0.232

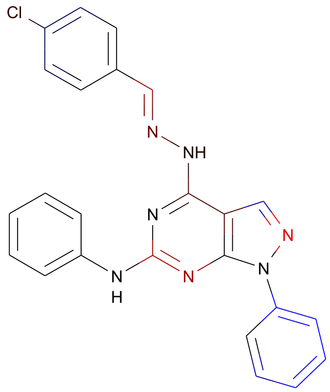

$C_{24}H_{18}ClN_7$

Molecular Weight: 439.89962

ALogP: 6.57

Rotatable Bonds: 6

Acceptors: 6

Donors: 2

Structural Similar Compounds

Name	Phenolphthalein	646	C.I. vat yellow 4
Structure	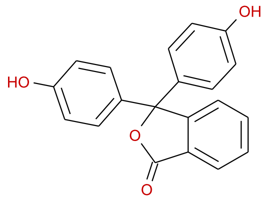	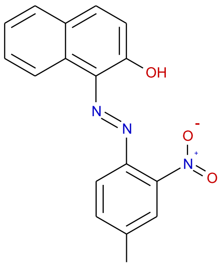	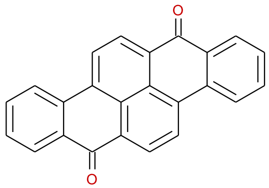
Actual Endpoint (-log C)	2.43468	0.937339	1.48417
Predicted Endpoint (-log C)	3.66084	3.26294	4.45029
Distance	0.947	0.950	1.029
Reference	CPDB	CPDB	CPDB

Model Prediction

Prediction: 0.71

Unit: mg/kg_body_weight/day

Mahalanobis Distance: 14.1

Mahalanobis Distance p-value: 2.14e-010

Mahalanobis Distance: The Mahalanobis distance (MD) is a generalization of the Euclidean distance that accounts for correlations among the X properties. It is calculated as the distance to the center of the training data. The larger the MD, the less trustworthy the prediction.
Mahalanobis Distance p-value: The p-value gives the fraction of training data with an MD greater than or equal to the one for the given sample, assuming normally distributed data. The smaller the p-value, the less trustworthy the prediciton. For highly non-normal X properties (e.g., fingerprints), the MD p-value is wildly inaccurate.

Model Applicability

Unknown features are fingerprint features in the query molecule, but not found or appearing too infrequently in the training set.

1. OPS PC16 out of range. Value: 5.1834. Training min, max, SD, explained variance: -3.1026, 4.016, 1.245, 0.0193.
2. OPS PC26 out of range. Value: -3.6723. Training min, max, SD, explained variance: -2.9667, 3.5042, 1.009, 0.0127.
3. Unknown ECFP_2 feature: -676555381: [*]n1:[*]:[*]:c:n:1
4. Unknown ECFP_2 feature: 1001947487: [*][c]1:[*]:[*]:n:n:1[c](:[*]):[*]
5. Unknown ECFP_2 feature: -174914108: [*]:n(:[*])[c](:c:[*]):c:[*]

Feature Contribution

Top features for positive contribution

Fingerprint	Bit/Smiles	Feature Structure	Score
ECFP_6	655739385	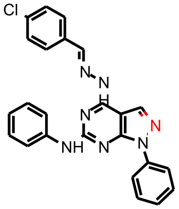 [*]:n:[*]	0.229

ECFP_6	834876373	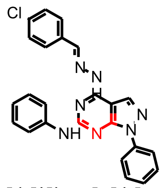 [*][c](:[*]):n:[c](:[*]):[*]	0.163
ECFP_6	-1925046727	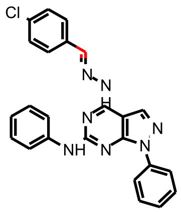 [*]C=[*]	0.145
**Top Features for negative contribution**			
**Fingerprint**	**Bit/Smiles**	**Feature Structure**	**Score**
ECFP_6	1996767644	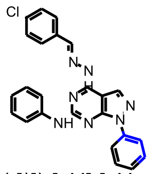 [*][c](:[*]):[cH]:[cH]:[*]	-0.251
ECFP_6	642810091	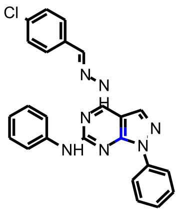 [*][c](:[*]):[*]	-0.247
ECFP_6	-182236392	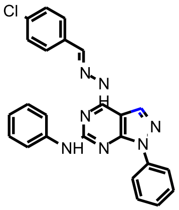 [*]:[cH]:[*]	-0.232

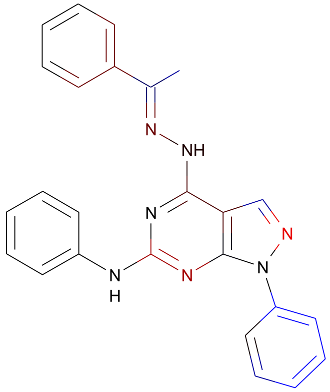

$C_{25}H_{21}N_7$
Molecular Weight: 419.48114
ALogP: 5.886
Rotatable Bonds: 6
Acceptors: 6
Donors: 2

Model Prediction

Prediction: 1.56
Unit: mg/kg_body_weight/day
Mahalanobis Distance: 13.6
Mahalanobis Distance p-value: 4.3e-009

Mahalanobis Distance: The Mahalanobis distance (MD) is a generalization of the Euclidean distance that accounts for correlations among the X properties. It is calculated as the distance to the center of the training data. The larger the MD, the less trustworthy the prediction.
Mahalanobis Distance p-value: The p-value gives the fraction of training data with an MD greater than or equal to the one for the given sample, assuming normally distributed data. The smaller the p-value, the less trustworthy the prediciton. For highly non-normal X properties (e.g., fingerprints), the MD p-value is wildly inaccurate.

Structural Similar Compounds

Name	Phenolphthalein	646	C.I. vat yellow 4
Structure	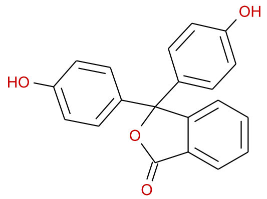	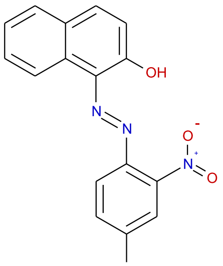	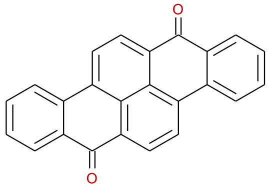
Actual Endpoint (-log C)	2.43468	0.937339	1.48417
Predicted Endpoint (-log C)	3.66084	3.26294	4.45029
Distance	0.923	0.930	1.015
Reference	CPDB	CPDB	CPDB

Model Applicability

Unknown features are fingerprint features in the query molecule, but not found or appearing too infrequently in the training set.

1. All properties and OPS components are within expected ranges.
2. Unknown ECFP_2 feature: -676555381: [*]n1:[*]:[*]:c:n:1
3. Unknown ECFP_2 feature: 1001947487: [*][c]1:[*]:[*]:n:n:1[c](:[*]):[*]
4. Unknown ECFP_2 feature: -174914108: [*]:n(:[*])[c](:c:[*]):c:[*]
5. Unknown ECFP_2 feature: 560380707: [*]NN=C([*])[*]
6. Unknown ECFP_2 feature: 128986386: [*]\N=C(/C)\[c](:[*]):[*]

Feature Contribution

Top features for positive contribution

Fingerprint	Bit/Smiles	Feature Structure	Score
ECFP_6	655739385	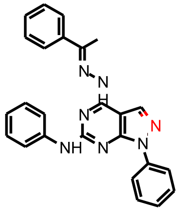 [*]:n:[*]	0.229

ECFP_6	834876373	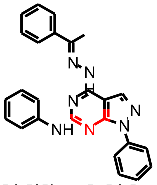 [*][c](:[*]):n:[c](:[*]):[*]	0.163
ECFP_6	-175146122	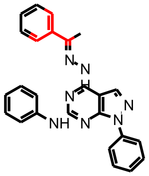 [*]C(=[*])[c](:[cH]:[*]):[cH]:[*]	0.107
**Top Features for negative contribution**			
**Fingerprint**	**Bit/Smiles**	**Feature Structure**	**Score**
ECFP_6	1996767644	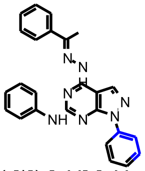 [*][c](:[*]):[cH]:[cH]:[*]	-0.251
ECFP_6	642810091	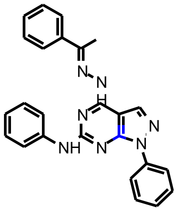 [*][c](:[*]):[*]	-0.247
ECFP_6	-182236392	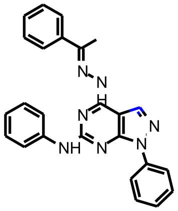 [*]:[cH]:[*]	-0.232

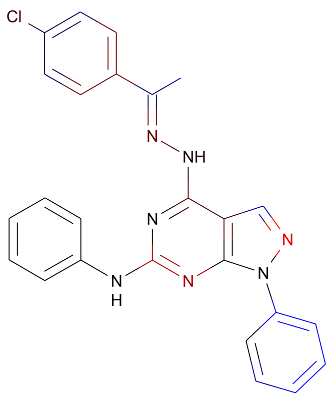

$C_{25}H_{20}ClN_7$
Molecular Weight: 453.9262
ALogP: 6.55
Rotatable Bonds: 6
Acceptors: 6
Donors: 2

Model Prediction

Prediction: 1.09
Unit: mg/kg_body_weight/day
Mahalanobis Distance: 15
Mahalanobis Distance p-value: 5.9e-013

Mahalanobis Distance: The Mahalanobis distance (MD) is a generalization of the Euclidean distance that accounts for correlations among the X properties. It is calculated as the distance to the center of the training data. The larger the MD, the less trustworthy the prediction.
Mahalanobis Distance p-value: The p-value gives the fraction of training data with an MD greater than or equal to the one for the given sample, assuming normally distributed data. The smaller the p-value, the less trustworthy the prediciton. For highly non-normal X properties (e.g., fingerprints), the MD p-value is wildly inaccurate.

Structural Similar Compounds

Name	646	Phenolphthalein	C.I. vat yellow 4
Structure	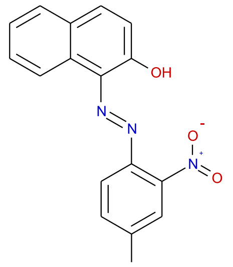	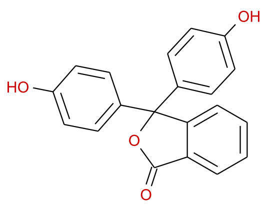	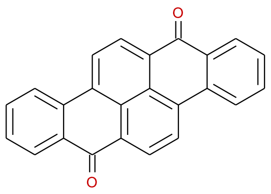
Actual Endpoint (-log C)	0.937339	2.43468	1.48417
Predicted Endpoint (-log C)	3.26294	3.66084	4.45029
Distance	0.952	0.952	1.033
Reference	CPDB	CPDB	CPDB

Model Applicability

Unknown features are fingerprint features in the query molecule, but not found or appearing too infrequently in the training set.

1. OPS PC16 out of range. Value: 4.0519. Training min, max, SD, explained variance: -3.1026, 4.016, 1.245, 0.0193.
2. Unknown ECFP_2 feature: -676555381: [*]n1:[*]:[*]:c:n:1
3. Unknown ECFP_2 feature: 1001947487: [*][c]1:[*]:[*]:n:n:1[c](:[*]):[*]
4. Unknown ECFP_2 feature: -174914108: [*]:n(:[*])[c](:c:[*]):c:[*]
5. Unknown ECFP_2 feature: 560380707: [*]NN=C([*])[*]
6. Unknown ECFP_2 feature: 128986386: [*]\N=C(/C)\[c](:[*]):[*]

Feature Contribution

Top features for positive contribution

Fingerprint	Bit/Smiles	Feature Structure	Score
ECFP_6	655739385	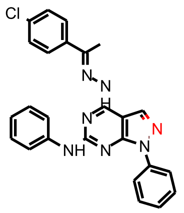 [*]:n:[*]	0.229

ECFP_6	834876373	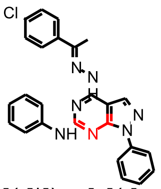 [*][c](:[*]):n:[c](:[*]):[*]	0.163
ECFP_6	-817402818	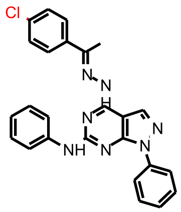 [*]Cl	0.129
**Top Features for negative contribution**			
**Fingerprint**	**Bit/Smiles**	**Feature Structure**	**Score**
ECFP_6	1996767644	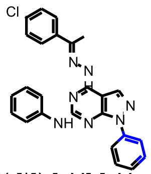 [*][c](:[*]):[cH]:[cH]:[*]	-0.251
ECFP_6	642810091	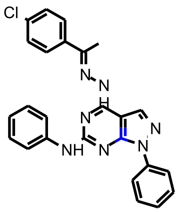 [*][c](:[*]):[*]	-0.247
ECFP_6	-182236392	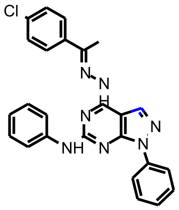 [*]:[cH]:[*]	-0.232

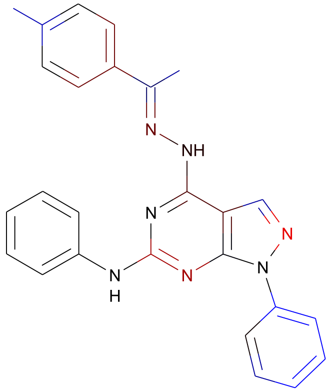

$C_{26}H_{23}N_7$
Molecular Weight: 433.50772
ALogP: 6.372
Rotatable Bonds: 6
Acceptors: 6
Donors: 2

Model Prediction

Prediction: 2.03
Unit: mg/kg_body_weight/day
Mahalanobis Distance: 13.8
Mahalanobis Distance p-value: 1.03e-009

Mahalanobis Distance: The Mahalanobis distance (MD) is a generalization of the Euclidean distance that accounts for correlations among the X properties. It is calculated as the distance to the center of the training data. The larger the MD, the less trustworthy the prediction.
Mahalanobis Distance p-value: The p-value gives the fraction of training data with an MD greater than or equal to the one for the given sample, assuming normally distributed data. The smaller the p-value, the less trustworthy the prediciton. For highly non-normal X properties (e.g., fingerprints), the MD p-value is wildly inaccurate.

Structural Similar Compounds

Name	646	Phenolphthalein	C.I. vat yellow 4
Structure	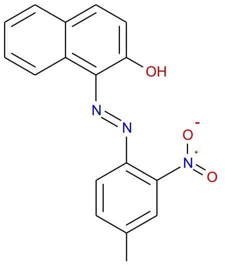	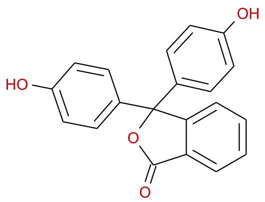	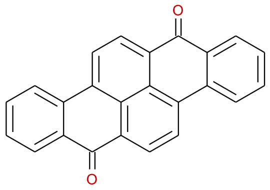
Actual Endpoint (-log C)	0.937339	2.43468	1.48417
Predicted Endpoint (-log C)	3.26294	3.66084	4.45029
Distance	0.928	0.940	1.025
Reference	CPDB	CPDB	CPDB

Model Applicability

Unknown features are fingerprint features in the query molecule, but not found or appearing too infrequently in the training set.

1. All properties and OPS components are within expected ranges.
2. Unknown ECFP_2 feature: -676555381: [*]n1:[*]:[*]:c:n:1
3. Unknown ECFP_2 feature: 1001947487: [*][c]1:[*]:[*]:n:n:1[c](:[*]):[*]
4. Unknown ECFP_2 feature: -174914108: [*]:n(:[*])[c](:c:[*]):c:[*]
5. Unknown ECFP_2 feature: 560380707: [*]NN=C([*])[*]
6. Unknown ECFP_2 feature: 128986386: [*]\N=C(/C)\[c](:[*]):[*]

Feature Contribution

Top features for positive contribution

Fingerprint	Bit/Smiles	Feature Structure	Score
ECFP_6	655739385	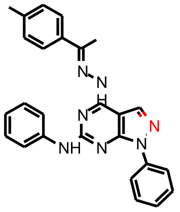 [*]:n:[*]	0.229

ECFP_6	834876373	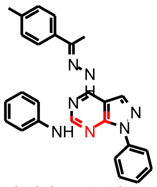 [*][c](:[*]):n:[c](:[*]):[*]	0.163
ECFP_6	-175146122	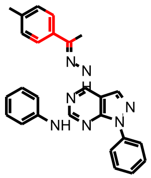 [*]C(=[*])[c](:[cH]:[*]):[cH]:[*]	0.107

Top Features for negative contribution

Fingerprint	Bit/Smiles	Feature Structure	Score
ECFP_6	1996767644	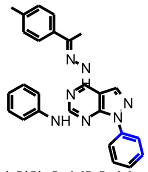 [*][c](:[*]):[cH]:[cH]:[*]	-0.251
ECFP_6	642810091	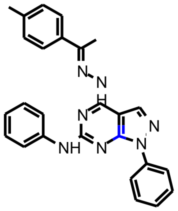 [*][c](:[*]):[*]	-0.247
ECFP_6	-182236392	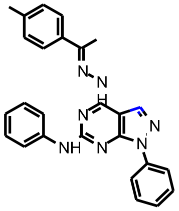 [*]:[cH]:[*]	-0.232

TOPKAT_Carcinogenic_Potency_TD50_Mouse

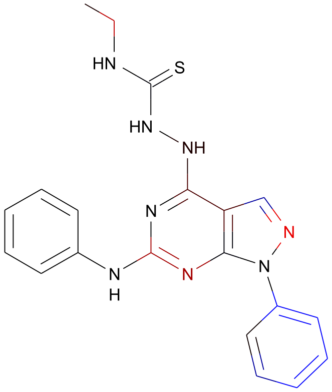

$C_{20}H_{20}N_8S$

Molecular Weight: 404.4914

ALogP: 4.99

Rotatable Bonds: 8

Acceptors: 6

Donors: 4

Structural Similar Compounds

Name	Ochratoxin A	542	4-Chloro-6-(2,3-xylidino)-2-pyri-mi-dinylthio(N-b-hydroxy-ethyl) acetamide
Structure	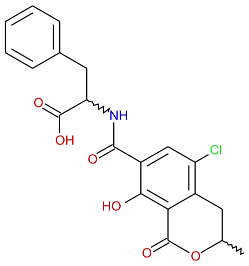	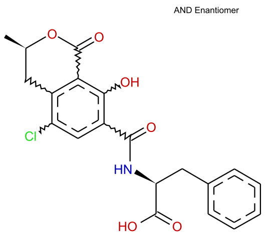	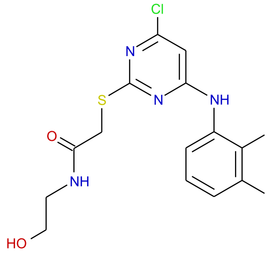
Actual Endpoint (-log C)	4.79932	4.79932	3.91517
Predicted Endpoint (-log C)	3.6353	3.6353	3.92186
Distance	0.862	0.862	0.882
Reference	CPDB	CPDB	CPDB

Model Prediction

Prediction: 2.3

Unit: mg/kg_body_weight/day

Mahalanobis Distance: 12.2

Mahalanobis Distance p-value: 6.91e-006

Mahalanobis Distance: The Mahalanobis distance (MD) is a generalization of the Euclidean distance that accounts for correlations among the X properties. It is calculated as the distance to the center of the training data. The larger the MD, the less trustworthy the prediction.

Mahalanobis Distance p-value: The p-value gives the fraction of training data with an MD greater than or equal to the one for the given sample, assuming normally distributed data. The smaller the p-value, the less trustworthy the prediciton. For highly non-normal X properties (e.g., fingerprints), the MD p-value is wildly inaccurate.

Model Applicability

Unknown features are fingerprint features in the query molecule, but not found or appearing too infrequently in the training set.

1. All properties and OPS components are within expected ranges.
2. Unknown ECFP_2 feature: -676555381: [*]n1:[*]:[*]:c:n:1
3. Unknown ECFP_2 feature: 1001947487: [*][c]1:[*]:[*]:n:n:1[c](:[*]):[*]
4. Unknown ECFP_2 feature: -174914108: [*]:n(:[*])[c](:c:[*]):c:[*]
5. Unknown ECFP_2 feature: 150794520: [*]NC(=S)N[*]

Feature Contribution

Top features for positive contribution

Fingerprint	Bit/Smiles	Feature Structure	Score
ECFP_6	655739385	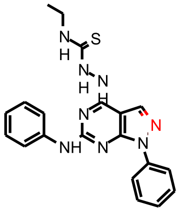 [*]:n:[*]	0.229

ECFP_6	1559650422	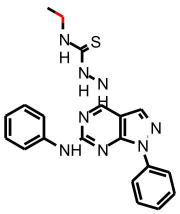 [*]C[*]	0.203
ECFP_6	834876373	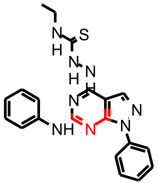 [*][c](:[*]):n:[c](:[*]):[*]	0.163

Top Features for negative contribution

Fingerprint	Bit/Smiles	Feature Structure	Score
ECFP_6	1996767644	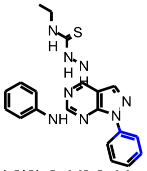 [*][c](:[*]):[cH]:[cH]:[*]	-0.251
ECFP_6	642810091	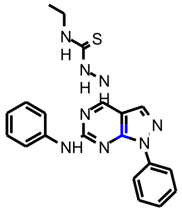 [*][c](:[*]):[*]	-0.247
ECFP_6	-182236392	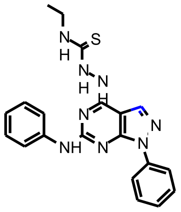 [*]:[cH]:[*]	-0.232

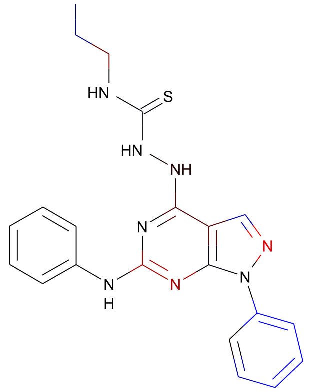

$C_{21}H_{22}N_8S$

Molecular Weight: 418.51798

ALogP: 5.514

Rotatable Bonds: 9

Acceptors: 6

Donors: 4

Model Prediction

Prediction: 2.91

Unit: mg/kg_body_weight/day

Mahalanobis Distance: 12.4

Mahalanobis Distance p-value: 3.54e-006

Mahalanobis Distance: The Mahalanobis distance (MD) is a generalization of the Euclidean distance that accounts for correlations among the X properties. It is calculated as the distance to the center of the training data. The larger the MD, the less trustworthy the prediction.
Mahalanobis Distance p-value: The p-value gives the fraction of training data with an MD greater than or equal to the one for the given sample, assuming normally distributed data. The smaller the p-value, the less trustworthy the prediciton. For highly non-normal X properties (e.g., fingerprints), the MD p-value is wildly inaccurate.

Structural Similar Compounds

Name	Ochratoxin A	542	4-Chloro-6-(2,3-xylidino)-2-pyri-mi-dinylthio(N-b-hydroxy-ethyl) acetamide
Structure	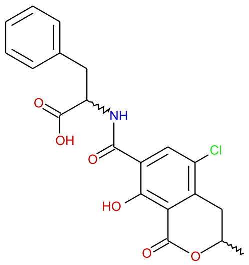	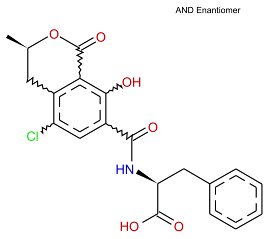	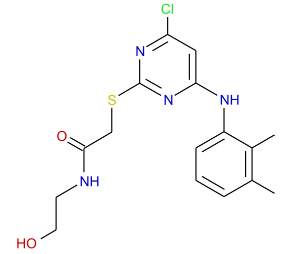
Actual Endpoint (-log C)	4.79932	4.79932	3.91517
Predicted Endpoint (-log C)	3.6353	3.6353	3.92186
Distance	0.894	0.894	0.899
Reference	CPDB	CPDB	CPDB

Model Applicability

Unknown features are fingerprint features in the query molecule, but not found or appearing too infrequently in the training set.

1. All properties and OPS components are within expected ranges.
2. Unknown ECFP_2 feature: -676555381: [*]n1:[*]:[*]:c:n:1
3. Unknown ECFP_2 feature: 1001947487: [*][c]1:[*]:[*]:n:n:1[c](:[*]):[*]
4. Unknown ECFP_2 feature: -174914108: [*]:n(:[*])[c](:c:[*]):c:[*]
5. Unknown ECFP_2 feature: 150794520: [*]NC(=S)N[*]

Feature Contribution

Top features for positive contribution

Fingerprint	Bit/Smiles	Feature Structure	Score
ECFP_6	655739385	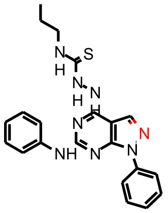 [*]:n:[*]	0.229

ECFP_6	1559650422	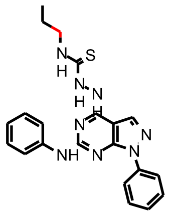 [*]C[*]	0.203
ECFP_6	834876373	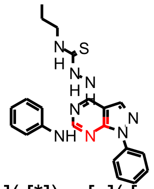 [*][c](:[*]):n:[c](:[*]):[*]	0.163
**Top Features for negative contribution**			
**Fingerprint**	**Bit/Smiles**	**Feature Structure**	**Score**
ECFP_6	1996767644	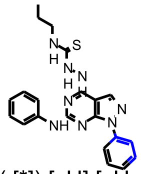 [*][c](:[*]):[cH]:[cH]:[*]	-0.251
ECFP_6	642810091	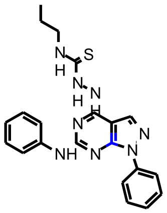 [*][c](:[*]):[*]	-0.247
ECFP_6	-182236392	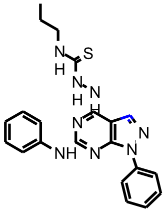 [*]:[cH]:[*]	-0.232

Erlotinib

TOPKAT_Carcinogenic_Potency_TD50_Mouse

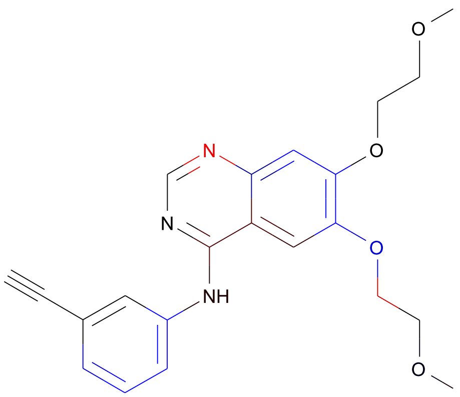

$C_{22}H_{23}N_3O_4$
Molecular Weight: 393.43572
ALogP: 4.309
Rotatable Bonds: 10
Acceptors: 7
Donors: 1

Model Prediction

Prediction: 39.8
Unit: mg/kg_body_weight/day
Mahalanobis Distance: 16.1
Mahalanobis Distance p-value: 1.15e-016

Mahalanobis Distance: The Mahalanobis distance (MD) is a generalization of the Euclidean distance that accounts for correlations among the X properties. It is calculated as the distance to the center of the training data. The larger the MD, the less trustworthy the prediction.
Mahalanobis Distance p-value: The p-value gives the fraction of training data with an MD greater than or equal to the one for the given sample, assuming normally distributed data. The smaller the p-value, the less trustworthy the prediciton. For highly non-normal X properties (e.g., fingerprints), the MD p-value is wildly inaccurate.

Structural Similar Compounds

Name	Compound LY171883	832	5,5´-(1,1´-Biphenyl)-2,5-dyl-bis (oxy)(2,2-dimethylpentanoic acid)
Structure	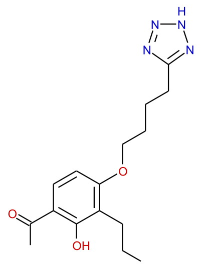	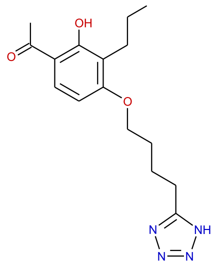	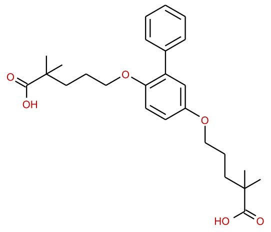
Actual Endpoint (-log C)	3.45372	3.45372	3.90166
Predicted Endpoint (-log C)	2.84749	2.80429	2.75893
Distance	0.772	0.782	0.796
Reference	CPDB	CPDB	CPDB

Model Applicability

Unknown features are fingerprint features in the query molecule, but not found or appearing too infrequently in the training set.

1. OPS PC13 out of range. Value: -3.2209. Training min, max, SD, explained variance: -3.068, 3.6909, 1.329, 0.0220.
2. Unknown ECFP_2 feature: -182178874: [*]#C[c](:c:[*]):c:[*]
3. Unknown ECFP_2 feature: 1139738044: [*]:[c](:[*])C#C
4. Unknown ECFP_2 feature: -1253653003: [*]COC

Feature Contribution

Top features for positive contribution

Fingerprint	Bit/Smiles	Feature Structure	Score
ECFP_6	655739385	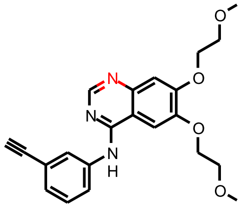 [*]:n:[*]	0.229

ECFP_6	1559650422	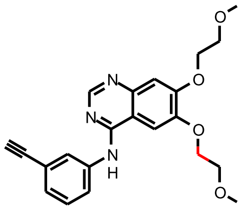 [*]C[*]	0.203
ECFP_6	1333660716	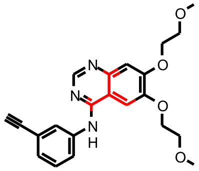 [*][c](:[*]):[c]1:[cH]:[*]:[*]:[c]:1:[*]	0.0746

Top Features for negative contribution

Fingerprint	Bit/Smiles	Feature Structure	Score
ECFP_6	1996767644	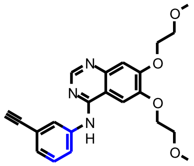 [*][c](:[*]):[cH]:[cH]:[*]	-0.251
ECFP_6	642810091	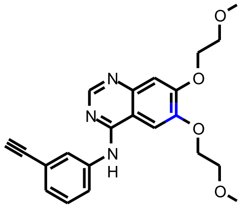 [*][c](:[*]):[*]	-0.247
ECFP_6	-182236392	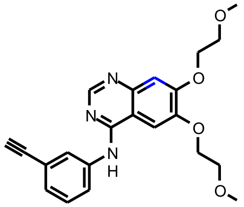 [*]:[cH]:[*]	-0.232

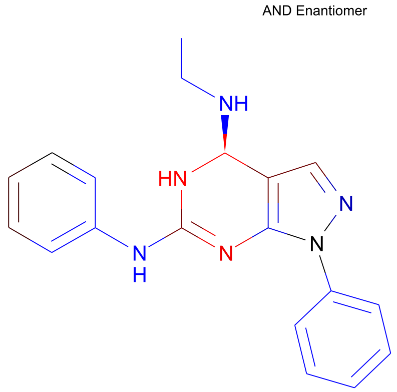

$C_{19}H_{20}N_6$

Molecular Weight: 332.4023

ALogP: 3.039

Rotatable Bonds: 5

Acceptors: 5

Donors: 3

Model Prediction

Prediction: 35

Unit: mg/kg_body_weight/day

Mahalanobis Distance: 15

Mahalanobis Distance p-value: 3.23e-011

Mahalanobis Distance: The Mahalanobis distance (MD) is a generalization of the Euclidean distance that accounts for correlations among the X properties. It is calculated as the distance to the center of the training data. The larger the MD, the less trustworthy the prediction.
Mahalanobis Distance p-value: The p-value gives the fraction of training data with an MD greater than or equal to the one for the given sample, assuming normally distributed data. The smaller the p-value, the less trustworthy the prediciton. For highly non-normal X properties (e.g., fingerprints), the MD p-value is wildly inaccurate.

Structural Similar Compounds

Name	796	Phenolphthalein	Fluvastatin
Structure	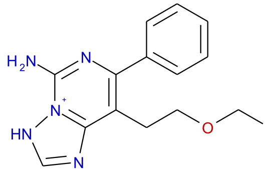	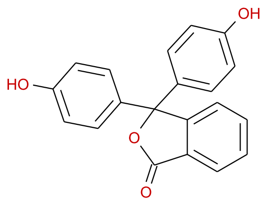	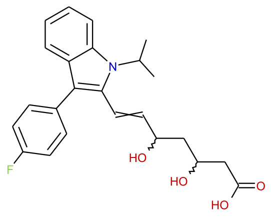
Actual Endpoint (-log C)	2.71505	2.54766	3.51742
Predicted Endpoint (-log C)	4.45918	3.7508	5.41573
Distance	0.653	0.680	0.716
Reference	CPDB	CPDB	CPDB

Model Applicability

Unknown features are fingerprint features in the query molecule, but not found or appearing too infrequently in the training set.

1. All properties and OPS components are within expected ranges.
2. Unknown FCFP_2 feature: 179977000: [*][c]1:[*]:[*]:n:n:1[c](:[*]):[*]
3. Unknown FCFP_2 feature: -1732563065: [*]:n(:[*])[c](:c:[*]):c:[*]

Feature Contribution

Top features for positive contribution

Fingerprint	Bit/Smiles	Feature Structure	Score
FCFP_6	1	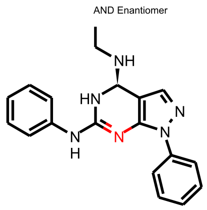 [*]N=[*]	0.234

FCFP_6	-885550502	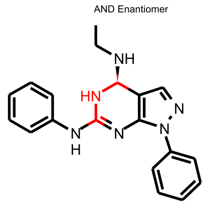 [*]C([*])NC(=[*])[*]	0.229
FCFP_6	203677720	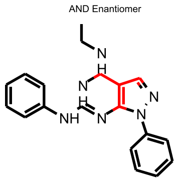 [*]C([*])[c]1:[cH]:[*]:[*]:[c]:1[*]	0.137

Top Features for negative contribution

Fingerprint	Bit/Smiles	Feature Structure	Score
FCFP_6	-1272709286	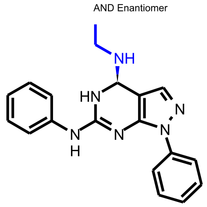 [*]NCC	-0.526
FCFP_6	991735244	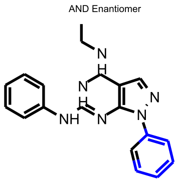 [*][c]1:[*]:[cH]:[cH]:[cH]:[cH]:1	-0.422
FCFP_6	-2093839777	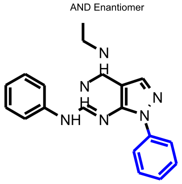 [*][c]1:[cH]:[cH]:[cH]:[cH]:[cH]:1	-0.378

7b TOPKAT_Carcinogenic_Potency_TD50_Rat

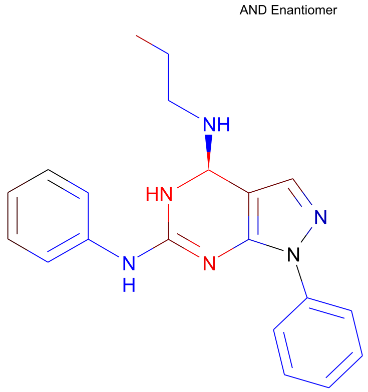

$C_{20}H_{22}N_6$
Molecular Weight: 346.42888
ALogP: 3.562
Rotatable Bonds: 6
Acceptors: 5
Donors: 3

Model Prediction

Prediction: 27.1
Unit: mg/kg_body_weight/day
Mahalanobis Distance: 15.2
Mahalanobis Distance p-value: 9.52e-012

Mahalanobis Distance: The Mahalanobis distance (MD) is a generalization of the Euclidean distance that accounts for correlations among the X properties. It is calculated as the distance to the center of the training data. The larger the MD, the less trustworthy the prediction.
Mahalanobis Distance p-value: The p-value gives the fraction of training data with an MD greater than or equal to the one for the given sample, assuming normally distributed data. The smaller the p-value, the less trustworthy the prediciton. For highly non-normal X properties (e.g., fingerprints), the MD p-value is wildly inaccurate.

Structural Similar Compounds

Name	913	Fluvastatin	796
Structure	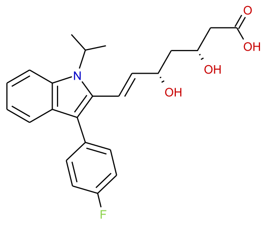	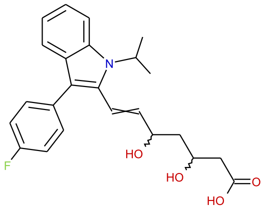	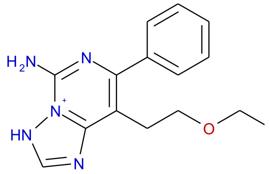
Actual Endpoint (-log C)	3.51742	3.51742	2.71505
Predicted Endpoint (-log C)	5.41573	5.41573	4.45918
Distance	0.675	0.675	0.681
Reference	CPDB	CPDB	CPDB

Model Applicability

Unknown features are fingerprint features in the query molecule, but not found or appearing too infrequently in the training set.

1. All properties and OPS components are within expected ranges.
2. Unknown FCFP_2 feature: 179977000: [*][c]1:[*]:[*]:n:n:1[c](:[*]):[*]
3. Unknown FCFP_2 feature: -1732563065: [*]:n(:[*])[c](:c:[*]):c:[*]

Feature Contribution

Top features for positive contribution

Fingerprint	Bit/Smiles	Feature Structure	Score
FCFP_6	1	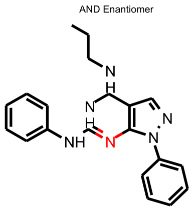 [*]N=[*]	0.234

FCFP_6	-885550502	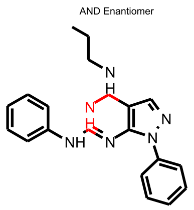 [*]C([*])NC(=[*])[*]	0.229
FCFP_6	203677720	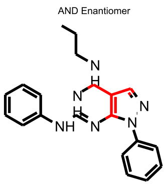 [*]C([*])[c]1:[cH]:[*]:[*]:[c]:1[*]	0.137

Top Features for negative contribution

Fingerprint	Bit/Smiles	Feature Structure	Score
FCFP_6	-1272709286	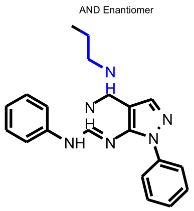 [*]NCC	-0.526
FCFP_6	991735244	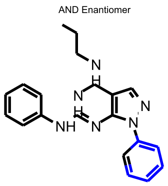 [*][c]1:[*]:[cH]:[cH]:[cH]:[cH]:1	-0.422
FCFP_6	-2093839777	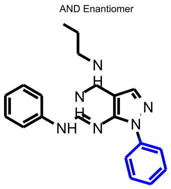 [*][c]1:[cH]:[cH]:[cH]:[cH]:[cH]:1	-0.378

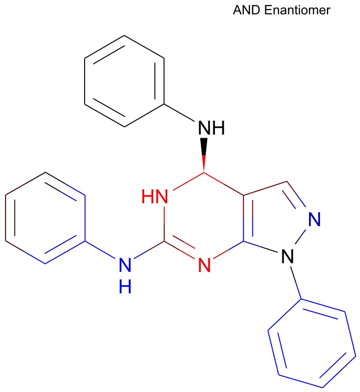

$C_{23}H_{20}N_6$

Molecular Weight: 380.4451

ALogP: 4.544

Rotatable Bonds: 5

Acceptors: 5

Donors: 3

Model Prediction

Prediction: 4.05

Unit: mg/kg_body_weight/day

Mahalanobis Distance: 16.3

Mahalanobis Distance p-value: 4.05e-015

Mahalanobis Distance: The Mahalanobis distance (MD) is a generalization of the Euclidean distance that accounts for correlations among the X properties. It is calculated as the distance to the center of the training data. The larger the MD, the less trustworthy the prediction.
Mahalanobis Distance p-value: The p-value gives the fraction of training data with an MD greater than or equal to the one for the given sample, assuming normally distributed data. The smaller the p-value, the less trustworthy the prediciton. For highly non-normal X properties (e.g., fingerprints), the MD p-value is wildly inaccurate.

Structural Similar Compounds

Name	Phenolphthalein	913	Fluvastatin
Structure	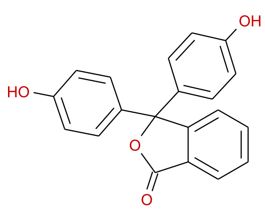	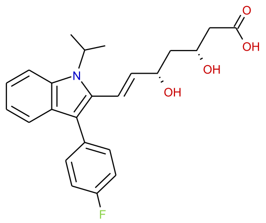	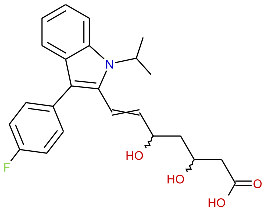
Actual Endpoint (-log C)	2.54766	3.51742	3.51742
Predicted Endpoint (-log C)	3.7508	5.41573	5.41573
Distance	0.745	0.773	0.773
Reference	CPDB	CPDB	CPDB

Model Applicability

Unknown features are fingerprint features in the query molecule, but not found or appearing too infrequently in the training set.

1. All properties and OPS components are within expected ranges.
2. Unknown FCFP_2 feature: 179977000: [*][c]1:[*]:[*]:n:n:1[c](:[*]):[*]
3. Unknown FCFP_2 feature: -1732563065: [*]:n(:[*])[c](:c:[*]):c:[*]

Feature Contribution

Top features for positive contribution			
**Fingerprint**	**Bit/Smiles**	**Feature Structure**	**Score**
FCFP_6	1	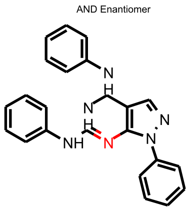 [*]N=[*]	0.234

FCFP_6	-885550502	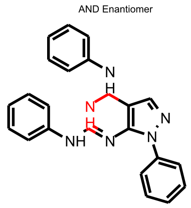 [*]C([*])NC(=[*])[*]	0.229
FCFP_6	203677720	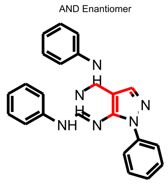 [*]C([*])[c]1:[cH]:[*]:[*]:[c]:1[*]	0.137

Top Features for negative contribution

Fingerprint	Bit/Smiles	Feature Structure	Score
FCFP_6	991735244	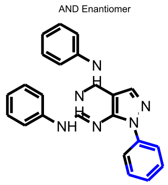 [*][c]1:[*]:[cH]:[cH] :[cH]:[cH]:1	-0.422
FCFP_6	-2093839777	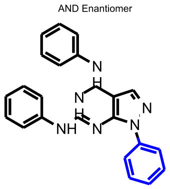 [*][c]1:[cH]:[cH]:[cH]:[cH]:[cH]:1	-0.378
FCFP_6	16	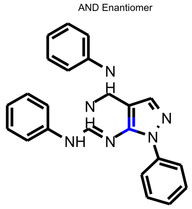 [*][c](:[*]):[*]	-0.354

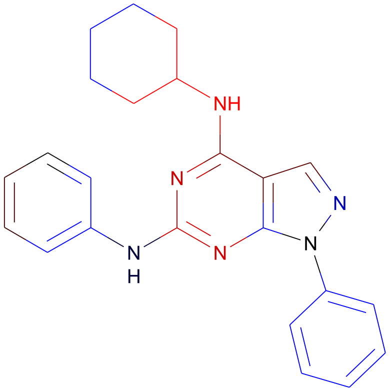

$C_{23}H_{24}N_6$
Molecular Weight: 384.47686
ALogP: 5.953
Rotatable Bonds: 5
Acceptors: 5
Donors: 2

Model Prediction

Prediction: 1.53
Unit: mg/kg_body_weight/day
Mahalanobis Distance: 17.3
Mahalanobis Distance p-value: 8.31e-019

Mahalanobis Distance: The Mahalanobis distance (MD) is a generalization of the Euclidean distance that accounts for correlations among the X properties. It is calculated as the distance to the center of the training data. The larger the MD, the less trustworthy the prediction.
Mahalanobis Distance p-value: The p-value gives the fraction of training data with an MD greater than or equal to the one for the given sample, assuming normally distributed data. The smaller the p-value, the less trustworthy the prediciton. For highly non-normal X properties (e.g., fingerprints), the MD p-value is wildly inaccurate.

Structural Similar Compounds

Name	Phenolphthalein	Indomethacin	646
Structure	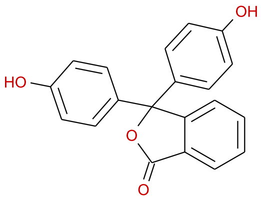	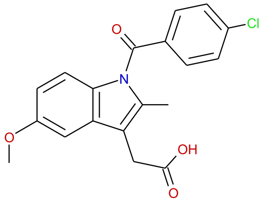	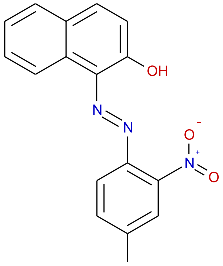
Actual Endpoint (-log C)	2.54766	5.49293	2.41938
Predicted Endpoint (-log C)	3.7508	4.9569	3.77987
Distance	0.762	0.786	0.796
Reference	CPDB	CPDB	CPDB

Model Applicability

Unknown features are fingerprint features in the query molecule, but not found or appearing too infrequently in the training set.

1. All properties and OPS components are within expected ranges.
2. Unknown FCFP_2 feature: 179977000: [*][c]1:[*]:[*]:n:n:1[c](:[*]):[*]
3. Unknown FCFP_2 feature: -1732563065: [*]:n(:[*])[c](:c:[*]):c:[*]

Feature Contribution

Top features for positive contribution			
**Fingerprint**	**Bit/Smiles**	**Feature Structure**	**Score**
FCFP_6	-1043250487	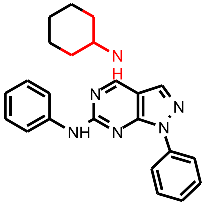 [*]CC(C[*])N[*]	1.15

FCFP_6	-1151914249	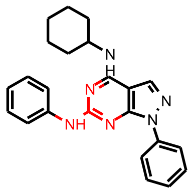 [*]N[c](:n:[*]):n:[*]	0.204
FCFP_6	307419094	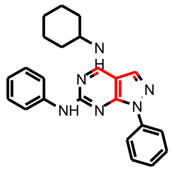 [*][c](:[*]):[c]1:[cH]:[*]:[*]:[c]:1:[*]	0.121
**Top Features for negative contribution**			
**Fingerprint**	**Bit/Smiles**	**Feature Structure**	**Score**
FCFP_6	1175638033	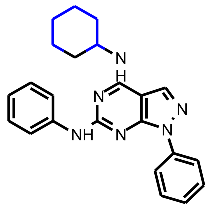 [*]C1[*]CCCC1	-0.512
FCFP_6	991735244	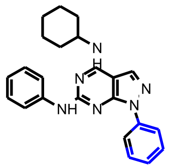 [*][c]1:[*]:[cH]:[cH] :[cH]:[cH]:1	-0.422
FCFP_6	-2093839777	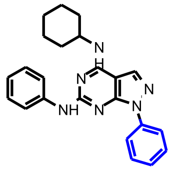 [*][c]1:[cH]:[cH]:[cH]:[cH]:[cH]:1	-0.378

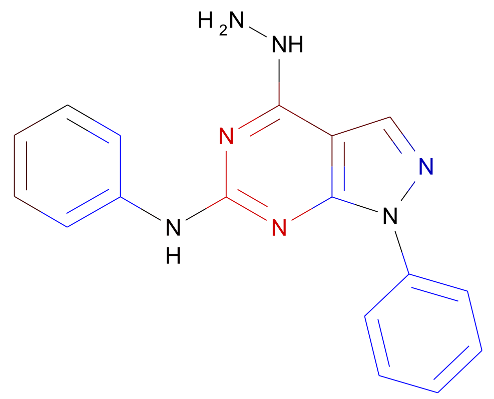

$C_{17}H_{15}N_7$
Molecular Weight: 317.3479
ALogP: 3.562
Rotatable Bonds: 4
Acceptors: 6
Donors: 3

Structural Similar Compounds

Name	4-(2-Hydroxyethylamino)-2-(5-nitro-2-thienyl)quinazoline	796	Phenolphthalein
Structure	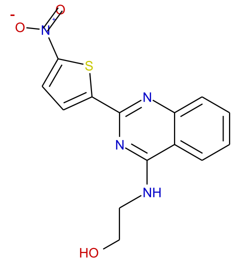	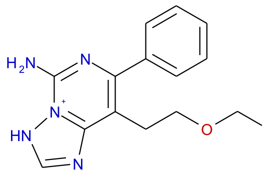	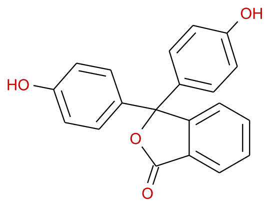
Actual Endpoint (-log C)	5.22831	2.71505	2.54766
Predicted Endpoint (-log C)	4.31976	4.45918	3.7508
Distance	0.712	0.732	0.756
Reference	CPDB	CPDB	CPDB

Model Prediction

Prediction: 18.7
Unit: mg/kg_body_weight/day
Mahalanobis Distance: 16.4
Mahalanobis Distance p-value: 1.25e-015

Mahalanobis Distance: The Mahalanobis distance (MD) is a generalization of the Euclidean distance that accounts for correlations among the X properties. It is calculated as the distance to the center of the training data. The larger the MD, the less trustworthy the prediction.
Mahalanobis Distance p-value: The p-value gives the fraction of training data with an MD greater than or equal to the one for the given sample, assuming normally distributed data. The smaller the p-value, the less trustworthy the prediciton. For highly non-normal X properties (e.g., fingerprints), the MD p-value is wildly inaccurate.

Model Applicability

Unknown features are fingerprint features in the query molecule, but not found or appearing too infrequently in the training set.

1. All properties and OPS components are within expected ranges.
2. Unknown FCFP_2 feature: 179977000: [*][c]1:[*]:[*]:n:n:1[c](:[*]):[*]
3. Unknown FCFP_2 feature: -1732563065: [*]:n(:[*])[c](:c:[*]):c:[*]

Feature Contribution

Top features for positive contribution

Fingerprint	Bit/Smiles	Feature Structure	Score
FCFP_6	-1151914249	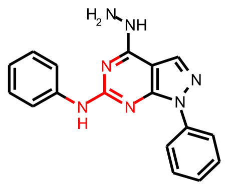 [*]N[c](:n:[*]):n:[*]	0.204

FCFP_6	307419094	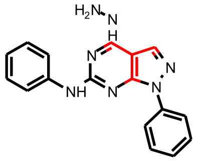 [*][c](:[*]):[c]1:[cH]:[*]:[*]:[c]:1:[*]	0.121
FCFP_6	3	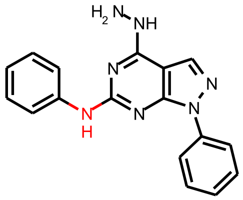 [*]N[*]	0.064
**Top Features for negative contribution**			
**Fingerprint**	**Bit/Smiles**	**Feature Structure**	**Score**
FCFP_6	991735244	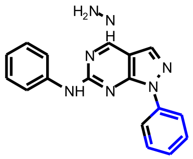 [*][c]1:[*]:[cH]:[cH]:[cH]:[cH]:1	-0.422
FCFP_6	-2093839777	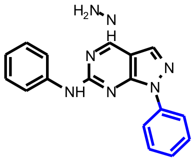 [*][c]1:[cH]:[cH]:[cH]:[cH]:[cH]:1	-0.378
FCFP_6	16	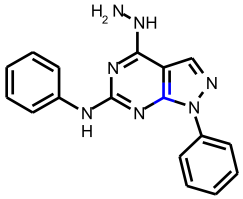 [*][c](:[*]):[*]	-0.354

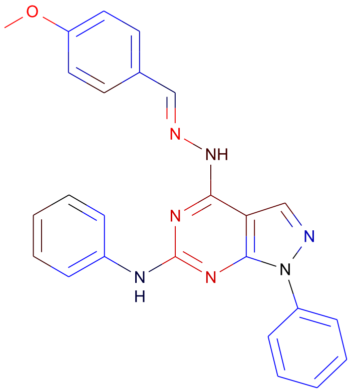

$C_{25}H_{21}N_7O$
Molecular Weight: 435.48054
ALogP: 5.889
Rotatable Bonds: 7
Acceptors: 7
Donors: 2

Model Prediction

Prediction: 1.66
Unit: mg/kg_body_weight/day
Mahalanobis Distance: 18.5
Mahalanobis Distance p-value: 5.79e-023

Mahalanobis Distance: The Mahalanobis distance (MD) is a generalization of the Euclidean distance that accounts for correlations among the X properties. It is calculated as the distance to the center of the training data. The larger the MD, the less trustworthy the prediction.
Mahalanobis Distance p-value: The p-value gives the fraction of training data with an MD greater than or equal to the one for the given sample, assuming normally distributed data. The smaller the p-value, the less trustworthy the prediciton. For highly non-normal X properties (e.g., fingerprints), the MD p-value is wildly inaccurate.

Structural Similar Compounds

Name	C.I. direct brown 95	Fluvastatin	913
Structure	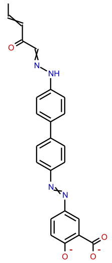	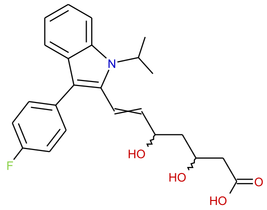	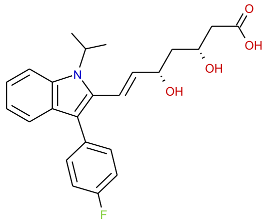
Actual Endpoint (-log C)	5.31387	3.51742	3.51742
Predicted Endpoint (-log C)	4.30266	5.41573	5.41573
Distance	0.856	0.897	0.897
Reference	CPDB	CPDB	CPDB

Model Applicability

Unknown features are fingerprint features in the query molecule, but not found or appearing too infrequently in the training set.

1. All properties and OPS components are within expected ranges.
2. Unknown FCFP_2 feature: 179977000: [*][c]1:[*]:[*]:n:n:1[c](:[*]):[*]
3. Unknown FCFP_2 feature: -1732563065: [*]:n(:[*])[c](:c:[*]):c:[*]

Feature Contribution

Top features for positive contribution

Fingerprint	Bit/Smiles	Feature Structure	Score
FCFP_6	136627117	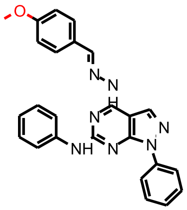 [*]OC	0.69

FCFP_6	1	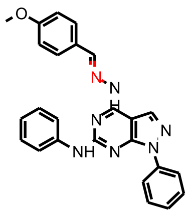 [*]N=[*]	0.234
FCFP_6	-1151914249	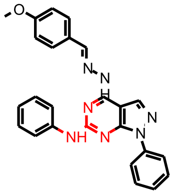 [*]N[c](:n:[*]):n:[*]	0.204
**Top Features for negative contribution**			
**Fingerprint**	**Bit/Smiles**	**Feature Structure**	**Score**
FCFP_6	991735244	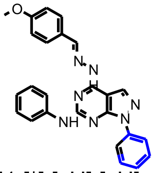 [*][c]1:[*]:[cH]:[cH]:[cH]:[cH]:1	-0.422
FCFP_6	-2093839777	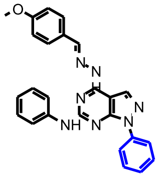 [*][c]1:[cH]:[cH]:[cH]:[cH]:[cH]:1	-0.378
FCFP_6	16	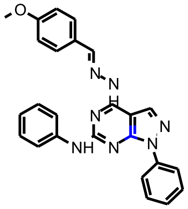 [*][c](:[*]):[*]	-0.354

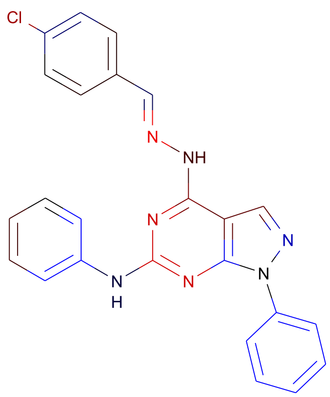

$C_{24}H_{18}ClN_7$
Molecular Weight: 439.89962
ALogP: 6.57
Rotatable Bonds: 6
Acceptors: 6
Donors: 2

Model Prediction

Prediction: 2.68
Unit: mg/kg_body_weight/day
Mahalanobis Distance: 18.8
Mahalanobis Distance p-value: 2.17e-024

Mahalanobis Distance: The Mahalanobis distance (MD) is a generalization of the Euclidean distance that accounts for correlations among the X properties. It is calculated as the distance to the center of the training data. The larger the MD, the less trustworthy the prediction.
Mahalanobis Distance p-value: The p-value gives the fraction of training data with an MD greater than or equal to the one for the given sample, assuming normally distributed data. The smaller the p-value, the less trustworthy the prediciton. For highly non-normal X properties (e.g., fingerprints), the MD p-value is wildly inaccurate.

Structural Similar Compounds

Name	Fluvastatin	913	Indomethacin
Structure	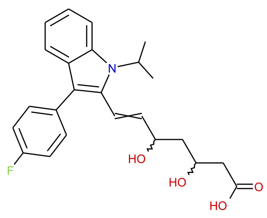	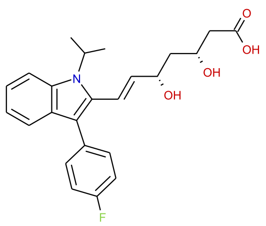	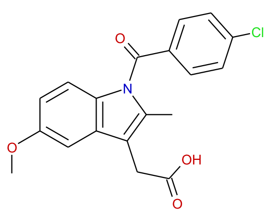
Actual Endpoint (-log C)	3.51742	3.51742	5.49293
Predicted Endpoint (-log C)	5.41573	5.41573	4.9569
Distance	0.863	0.863	0.865
Reference	CPDB	CPDB	CPDB

Model Applicability

Unknown features are fingerprint features in the query molecule, but not found or appearing too infrequently in the training set.

1. All properties and OPS components are within expected ranges.
2. Unknown FCFP_2 feature: 179977000: [*][c]1:[*]:[*]:n:n:1[c](:[*]):[*]
3. Unknown FCFP_2 feature: -1732563065: [*]:n(:[*])[c](:c:[*]):c:[*]

Feature Contribution

Top features for positive contribution

Fingerprint	Bit/Smiles	Feature Structure	Score
FCFP_6	1	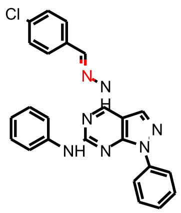 [*]N=[*]	0.234

FCFP_6	-1151914249	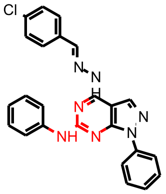<br>[*]N[c](:n:[*]):n:[*]	0.204
FCFP_6	32	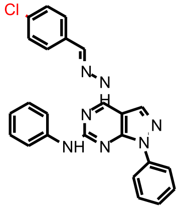<br>[*]Cl	0.154
**Top Features for negative contribution**			
**Fingerprint**	**Bit/Smiles**	**Feature Structure**	**Score**
FCFP_6	991735244	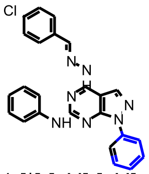<br>[*][c]1:[*]:[cH]:[cH]:[cH]:[cH]:1	-0.422
FCFP_6	-2093839777	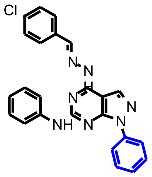<br>[*][c]1:[cH]:[cH]:[cH]:[cH]:[cH]:1	-0.378
FCFP_6	16	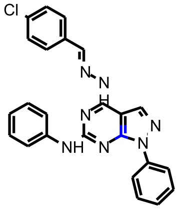<br>[*][c](:[*]):[*]	-0.354

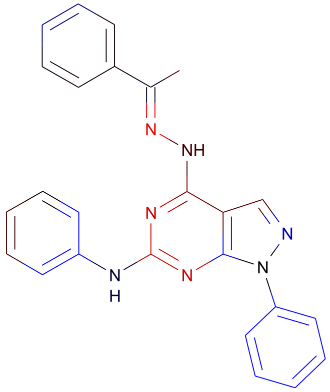

$C_{25}H_{21}N_7$
Molecular Weight: 419.48114
ALogP: 5.886
Rotatable Bonds: 6
Acceptors: 6
Donors: 2

Model Prediction

Prediction: 3.79
Unit: mg/kg_body_weight/day
Mahalanobis Distance: 17.7
Mahalanobis Distance p-value: 3.33e-020

Mahalanobis Distance: The Mahalanobis distance (MD) is a generalization of the Euclidean distance that accounts for correlations among the X properties. It is calculated as the distance to the center of the training data. The larger the MD, the less trustworthy the prediction.
Mahalanobis Distance p-value: The p-value gives the fraction of training data with an MD greater than or equal to the one for the given sample, assuming normally distributed data. The smaller the p-value, the less trustworthy the prediciton. For highly non-normal X properties (e.g., fingerprints), the MD p-value is wildly inaccurate.

Structural Similar Compounds

Name	913	Fluvastatin	Phenolphthalein
Structure	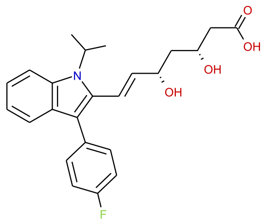	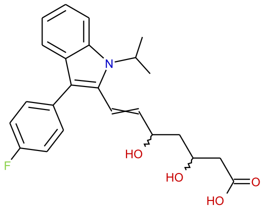	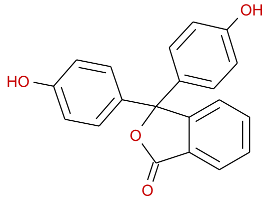
Actual Endpoint (-log C)	3.51742	3.51742	2.54766
Predicted Endpoint (-log C)	5.41573	5.41573	3.7508
Distance	0.869	0.869	0.874
Reference	CPDB	CPDB	CPDB

Model Applicability

Unknown features are fingerprint features in the query molecule, but not found or appearing too infrequently in the training set.

1. All properties and OPS components are within expected ranges.
2. Unknown FCFP_2 feature: 179977000: [*][c]1:[*]:[*]:n:n:1[c](:[*]):[*]
3. Unknown FCFP_2 feature: -1732563065: [*]:n(:[*])[c](:c:[*]):c:[*]

Feature Contribution

Top features for positive contribution			
**Fingerprint**	**Bit/Smiles**	**Feature Structure**	**Score**
FCFP_6	1	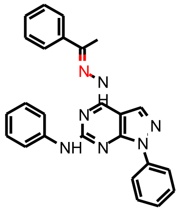 [*]N=[*]	0.234

FCFP_6	-1151914249	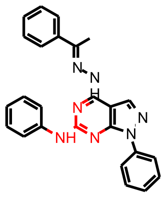<br>[*]N[c](:n:[*]):n:[*]	0.204
FCFP_6	203677720	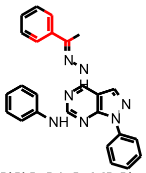<br>[*]C([*])[c]1:[cH]:[*]:[*]:[c]:1[*]	0.137
**Top Features for negative contribution**			
**Fingerprint**	**Bit/Smiles**	**Feature Structure**	**Score**
FCFP_6	991735244	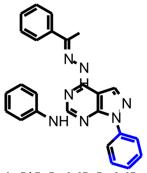<br>[*][c]1:[*]:[cH]:[cH]:[cH]:[cH]:1	-0.422
FCFP_6	-2093839777	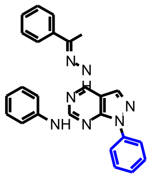<br>[*][c]1:[cH]:[cH]:[cH]:[cH]:[cH]:1	-0.378
FCFP_6	16	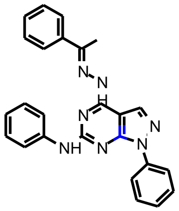<br>[*][c](:[*]):[*]	-0.354

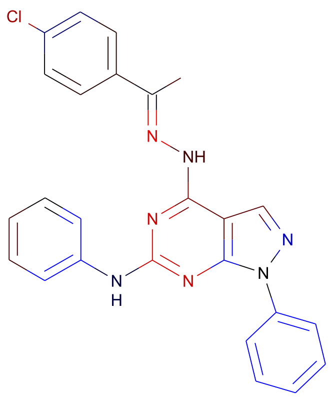

$C_{25}H_{20}ClN_7$
Molecular Weight: 453.9262
ALogP: 6.55
Rotatable Bonds: 6
Acceptors: 6
Donors: 2

Model Prediction

Prediction: 2.32
Unit: mg/kg_body_weight/day
Mahalanobis Distance: 19
Mahalanobis Distance p-value: 4.94e-025

Mahalanobis Distance: The Mahalanobis distance (MD) is a generalization of the Euclidean distance that accounts for correlations among the X properties. It is calculated as the distance to the center of the training data. The larger the MD, the less trustworthy the prediction.
Mahalanobis Distance p-value: The p-value gives the fraction of training data with an MD greater than or equal to the one for the given sample, assuming normally distributed data. The smaller the p-value, the less trustworthy the prediciton. For highly non-normal X properties (e.g., fingerprints), the MD p-value is wildly inaccurate.

Structural Similar Compounds

Name	Fluvastatin	913	Indomethacin
Structure	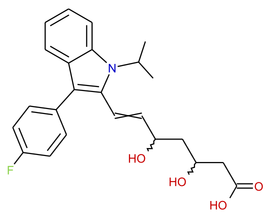	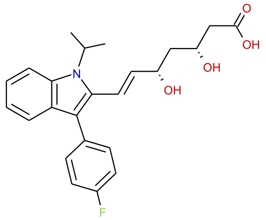	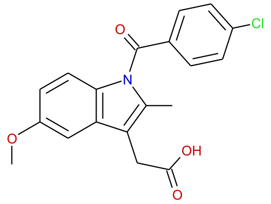
Actual Endpoint (-log C)	3.51742	3.51742	5.49293
Predicted Endpoint (-log C)	5.41573	5.41573	4.9569
Distance	0.861	0.861	0.872
Reference	CPDB	CPDB	CPDB

Model Applicability

Unknown features are fingerprint features in the query molecule, but not found or appearing too infrequently in the training set.

1. All properties and OPS components are within expected ranges.
2. Unknown FCFP_2 feature: 179977000: [*][c]1:[*]:[*]:n:n:1[c](:[*]):[*]
3. Unknown FCFP_2 feature: -1732563065: [*]:n(:[*])[c](:c:[*]):c:[*]

Feature Contribution

Top features for positive contribution

Fingerprint	Bit/Smiles	Feature Structure	Score
FCFP_6	1	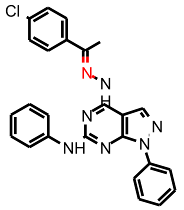 [*]N=[*]	0.234

FCFP_6	-1151914249	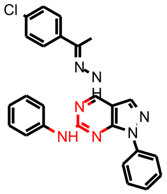<br>[*]N[c](:n:[*]):n:[*]	0.204
FCFP_6	32	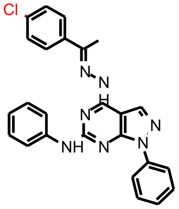<br>[*]Cl	0.154
**Top Features for negative contribution**			
**Fingerprint**	**Bit/Smiles**	**Feature Structure**	**Score**
FCFP_6	991735244	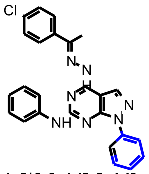<br>[*][c]1:[*]:[cH]:[cH]<br>:[cH]:[cH]:1	-0.422
FCFP_6	-2093839777	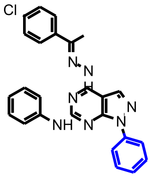<br>[*][c]1:[cH]:[cH]:[cH<br>]:[cH]:[cH]:1	-0.378
FCFP_6	16	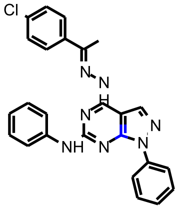<br>[*][c](:[*]):[*]	-0.354

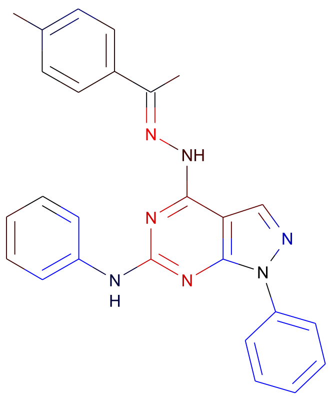

$C_{26}H_{23}N_7$
Molecular Weight: 433.50772
ALogP: 6.372
Rotatable Bonds: 6
Acceptors: 6
Donors: 2

Model Prediction

Prediction: 2.98
Unit: mg/kg_body_weight/day
Mahalanobis Distance: 18.1
Mahalanobis Distance p-value: 1.02e-021

Mahalanobis Distance: The Mahalanobis distance (MD) is a generalization of the Euclidean distance that accounts for correlations among the X properties. It is calculated as the distance to the center of the training data. The larger the MD, the less trustworthy the prediction.
Mahalanobis Distance p-value: The p-value gives the fraction of training data with an MD greater than or equal to the one for the given sample, assuming normally distributed data. The smaller the p-value, the less trustworthy the prediciton. For highly non-normal X properties (e.g., fingerprints), the MD p-value is wildly inaccurate.

Structural Similar Compounds

Name	913	Fluvastatin	646
Structure	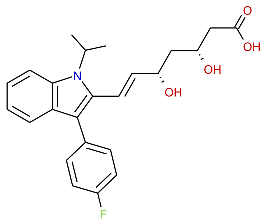	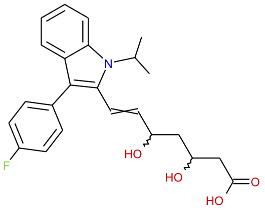	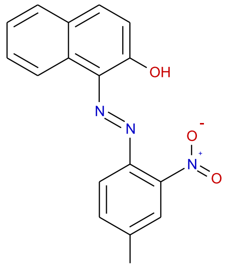
Actual Endpoint (-log C)	3.51742	3.51742	2.41938
Predicted Endpoint (-log C)	5.41573	5.41573	3.77987
Distance	0.881	0.881	0.888
Reference	CPDB	CPDB	CPDB

Model Applicability

Unknown features are fingerprint features in the query molecule, but not found or appearing too infrequently in the training set.

1. All properties and OPS components are within expected ranges.
2. Unknown FCFP_2 feature: 179977000: [*][c]1:[*]:[*]:n:n:1[c](:[*]):[*]
3. Unknown FCFP_2 feature: -1732563065: [*]:n(:[*])[c](:c:[*]):c:[*]

Feature Contribution

Top features for positive contribution

Fingerprint	Bit/Smiles	Feature Structure	Score
FCFP_6	1	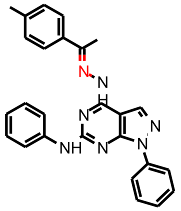 [*]N=[*]	0.234

FCFP_6	-1151914249	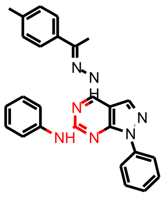 [*]N[c](:n:[*]):n:[*]	0.204
FCFP_6	203677720	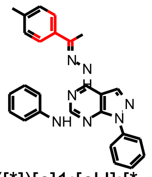 [*]C([*])[c]1:[cH]:[*]:[*]:[c]:1[*]	0.137

Top Features for negative contribution

Fingerprint	Bit/Smiles	Feature Structure	Score
FCFP_6	991735244	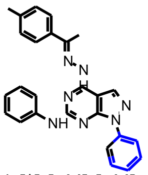 [*][c]1:[*]:[cH]:[cH] :[cH]:[cH]:1	-0.422
FCFP_6	-2093839777	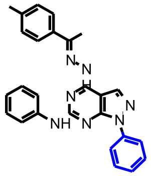 [*][c]1:[cH]:[cH]:[cH]:[cH]:[cH]:1	-0.378
FCFP_6	16	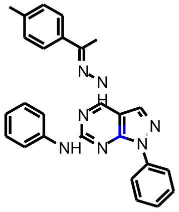 [*][c](:[*]):[*]	-0.354

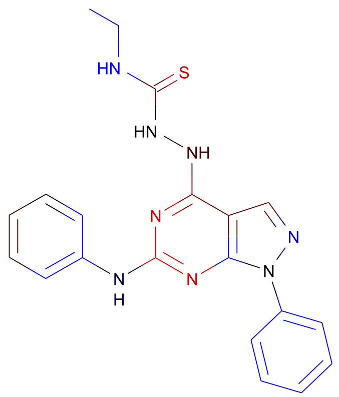

$C_{20}H_{20}N_8S$
Molecular Weight: 404.4914
ALogP: 4.99
Rotatable Bonds: 8
Acceptors: 6
Donors: 4

Model Prediction

Prediction: 32
Unit: mg/kg_body_weight/day
Mahalanobis Distance: 18.2
Mahalanobis Distance p-value: 3.91e-022

Mahalanobis Distance: The Mahalanobis distance (MD) is a generalization of the Euclidean distance that accounts for correlations among the X properties. It is calculated as the distance to the center of the training data. The larger the MD, the less trustworthy the prediction.
Mahalanobis Distance p-value: The p-value gives the fraction of training data with an MD greater than or equal to the one for the given sample, assuming normally distributed data. The smaller the p-value, the less trustworthy the prediciton. For highly non-normal X properties (e.g., fingerprints), the MD p-value is wildly inaccurate.

Structural Similar Compounds

Name	Fluvastatin	913	Ochratoxin A
Structure	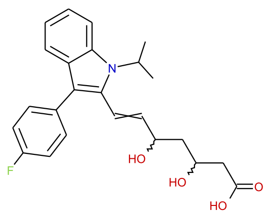	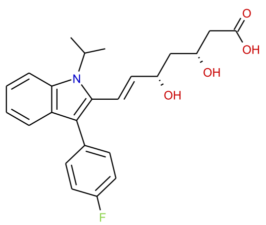	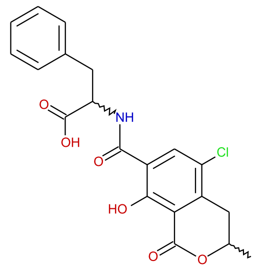
Actual Endpoint (-log C)	3.51742	3.51742	6.47264
Predicted Endpoint (-log C)	5.41573	5.41573	5.06501
Distance	0.750	0.750	0.803
Reference	CPDB	CPDB	CPDB

Model Applicability

Unknown features are fingerprint features in the query molecule, but not found or appearing too infrequently in the training set.

1. All properties and OPS components are within expected ranges.
2. Unknown FCFP_2 feature: 179977000: [*][c]1:[*]:[*]:n:n:1[c](:[*]):[*]
3. Unknown FCFP_2 feature: -1732563065: [*]:n(:[*])[c](:c:[*]):c:[*]

Feature Contribution

Top features for positive contribution

Fingerprint	Bit/Smiles	Feature Structure	Score
FCFP_6	1	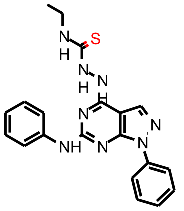 [*]N=[*]	0.234

FCFP_6	-885550502	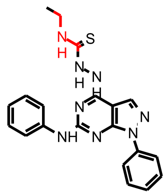 [*]C([*])NC(=[*])[*]	0.229
FCFP_6	-1151914249	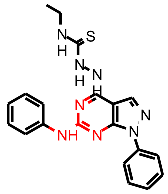 [*]N[c](:n:[*]):n:[*]	0.204

Top Features for negative contribution

Fingerprint	Bit/Smiles	Feature Structure	Score
FCFP_6	-1272709286	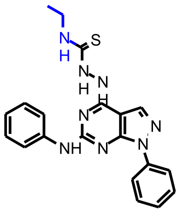 [*]NCC	-0.526
FCFP_6	991735244	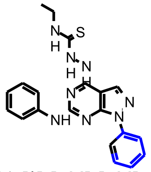 [*][c]1:[*]:[cH]:[cH]:[cH]:[cH]:1	-0.422
FCFP_6	-2093839777	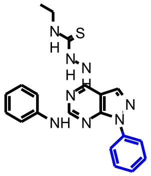 [*][c]1:[cH]:[cH]:[cH]:[cH]:[cH]:1	-0.378

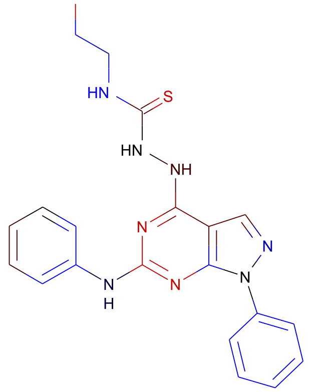

$C_{21}H_{22}N_8S$
Molecular Weight: 418.51798
ALogP: 5.514
Rotatable Bonds: 9
Acceptors: 6
Donors: 4

Model Prediction

Prediction: 24.7
Unit: mg/kg_body_weight/day
Mahalanobis Distance: 18.6
Mahalanobis Distance p-value: 1.22e-023

Mahalanobis Distance: The Mahalanobis distance (MD) is a generalization of the Euclidean distance that accounts for correlations among the X properties. It is calculated as the distance to the center of the training data. The larger the MD, the less trustworthy the prediction.
Mahalanobis Distance p-value: The p-value gives the fraction of training data with an MD greater than or equal to the one for the given sample, assuming normally distributed data. The smaller the p-value, the less trustworthy the prediciton. For highly non-normal X properties (e.g., fingerprints), the MD p-value is wildly inaccurate.

Structural Similar Compounds

Name	Fluvastatin	913	4-Bis(2-hydroxyethyl)amino-2-(5-nitro-2-thienyl)quinazoline
Structure	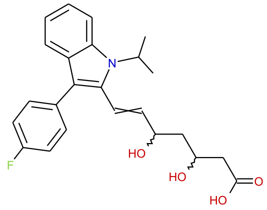	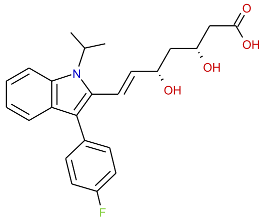	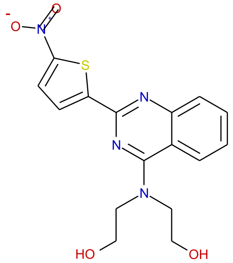
Actual Endpoint (-log C)	3.51742	3.51742	5.05984
Predicted Endpoint (-log C)	5.41573	5.41573	4.23808
Distance	0.757	0.757	0.834
Reference	CPDB	CPDB	CPDB

Model Applicability

Unknown features are fingerprint features in the query molecule, but not found or appearing too infrequently in the training set.

1. All properties and OPS components are within expected ranges.
2. Unknown FCFP_2 feature: 179977000: [*][c]1:[*]:[*]:n:n:1[c](:[*]):[*]
3. Unknown FCFP_2 feature: -1732563065: [*]:n(:[*])[c](:c:[*]):c:[*]

Feature Contribution

Top features for positive contribution

Fingerprint	Bit/Smiles	Feature Structure	Score
FCFP_6	1	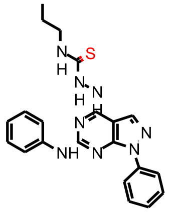 [*]N=[*]	0.234

FCFP_6	-885550502	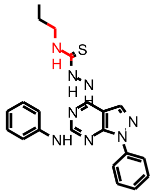 [*]C([*])NC(=[*])[*]	0.229
FCFP_6	-1151914249	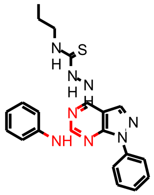 [*]N[c](:n:[*]):n:[*]	0.204

Top Features for negative contribution

Fingerprint	Bit/Smiles	Feature Structure	Score
FCFP_6	-1272709286	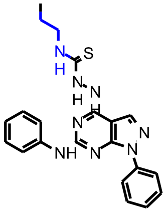 [*]NCC	-0.526
FCFP_6	991735244	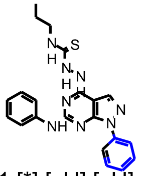 [*][c]1:[*]:[cH]:[cH]:[cH]:[cH]:1	-0.422
FCFP_6	-2093839777	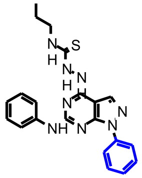 [*][c]1:[cH]:[cH]:[cH]:[cH]:[cH]:1	-0.378

Erlotinib

TOPKAT_Carcinogenic_Potency_TD50_Rat

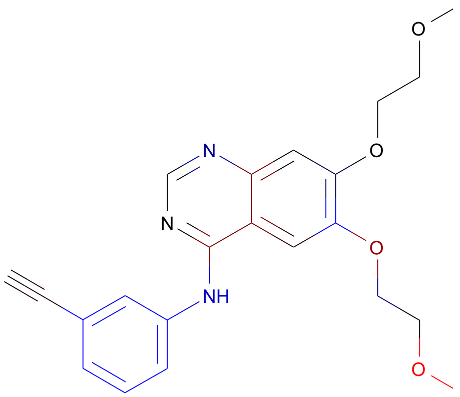

$C_{22}H_{23}N_3O_4$

Molecular Weight: 393.43572

ALogP: 4.309

Rotatable Bonds: 10

Acceptors: 7

Donors: 1

Model Prediction

Prediction: 8.06

Unit: mg/kg_body_weight/day

Mahalanobis Distance: 17.7

Mahalanobis Distance p-value: 3.99e-020

Mahalanobis Distance: The Mahalanobis distance (MD) is a generalization of the Euclidean distance that accounts for correlations among the X properties. It is calculated as the distance to the center of the training data. The larger the MD, the less trustworthy the prediction.
Mahalanobis Distance p-value: The p-value gives the fraction of training data with an MD greater than or equal to the one for the given sample, assuming normally distributed data. The smaller the p-value, the less trustworthy the prediciton. For highly non-normal X properties (e.g., fingerprints), the MD p-value is wildly inaccurate.

Structural Similar Compounds

Name	Loxtidine	C.I. direct brown 95	Omeprazole
Structure	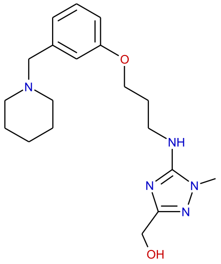	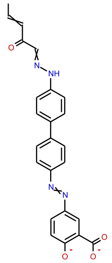	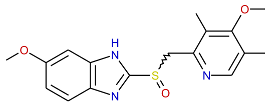
Actual Endpoint (-log C)	2.87532	5.31387	3.4628
Predicted Endpoint (-log C)	3.63996	4.30266	4.7324
Distance	0.685	0.715	0.741
Reference	CPDB	CPDB	CPDB

Model Applicability

Unknown features are fingerprint features in the query molecule, but not found or appearing too infrequently in the training set.

1. All properties and OPS components are within expected ranges.
2. Unknown FCFP_2 feature: 902193919: [*]:[c](:[*])C#C

Feature Contribution

Top features for positive contribution

Fingerprint	Bit/Smiles	Feature Structure	Score
FCFP_6	136627117	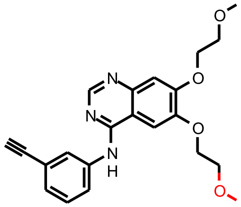 [*]OC	0.69

FCFP_6	1	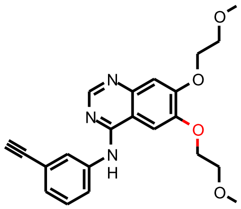 [*]N=[*]	0.234
FCFP_6	203677720	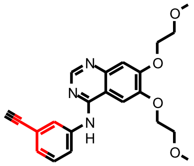 [*]C([*])[c]1:[cH]:[*]:[*]:[c]:1[*]	0.137
**Top Features for negative contribution**			
**Fingerprint**	**Bit/Smiles**	**Feature Structure**	**Score**
FCFP_6	991735244	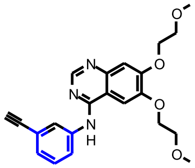 [*][c]1:[*]:[cH]:[cH] :[cH]:[cH]:1	-0.422
FCFP_6	16	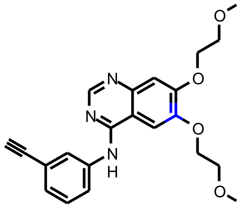 [*][c](:[*]):[*]	-0.354
FCFP_6	590925877	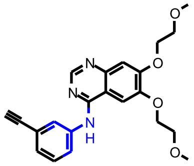 [*]N[c](:[cH]:[*]):[c H]:[*]	-0.323

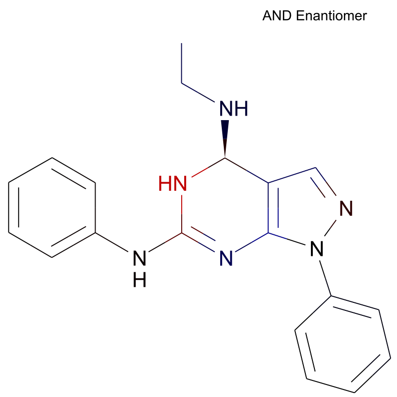

$C_{19}H_{20}N_6$
Molecular Weight: 332.4023
ALogP: 3.039
Rotatable Bonds: 5
Acceptors: 5
Donors: 3

Model Prediction

Prediction: 0.287
Unit: g/kg_body_weight
Mahalanobis Distance: 11
Mahalanobis Distance p-value: 8.84e-007

Mahalanobis Distance: The Mahalanobis distance (MD) is a generalization of the Euclidean distance that accounts for correlations among the X properties. It is calculated as the distance to the center of the training data. The larger the MD, the less trustworthy the prediction.
Mahalanobis Distance p-value: The p-value gives the fraction of training data with an MD greater than or equal to the one for the given sample, assuming normally distributed data. The smaller the p-value, the less trustworthy the prediciton. For highly non-normal X properties (e.g., fingerprints), the MD p-value is wildly inaccurate.

Structural Similar Compounds

Name	DISPERSE YELLOW 3	PHENOLPHTHALEIN	BENZIDINE,3,3'-DIMETHOXY-
Structure	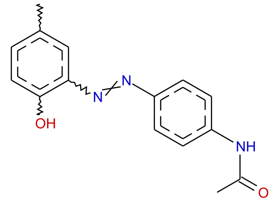	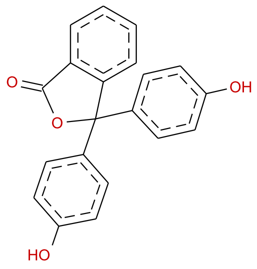	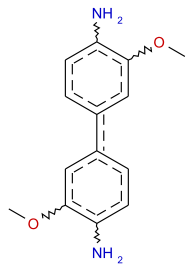
Actual Endpoint (-log C)	2.77703	2.20184	4.06569
Predicted Endpoint (-log C)	2.80195	2.8857	3.57405
Distance	0.655	0.665	0.735
Reference	NCI/NTP TR-222	NCI/NTP TR-465	NCI/NTP Report 10, Nov. 1987

Model Applicability

Unknown features are fingerprint features in the query molecule, but not found or appearing too infrequently in the training set.

1. All properties and OPS components are within expected ranges.
2. Unknown FCFP_2 feature: 179977000: [*][c]1:[*]:[*]:n:n:1[c](:[*]):[*]
3. Unknown FCFP_2 feature: 2119857014: [*]NC(N[*])[c](:[*]):[*]
4. Unknown FCFP_2 feature: -1732563065: [*]:n(:[*])[c](:c:[*]):c:[*]

Feature Contribution

Top features for positive contribution

Fingerprint	Bit/Smiles	Feature Structure	Score
FCFP_2	-885550502	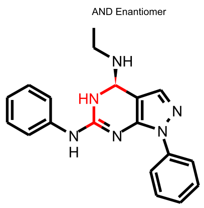 [*]C([*])NC(=[*])[*]	0.115

FCFP_2	3	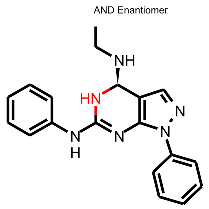 [*]N[*]	0.0737
FCFP_2	17	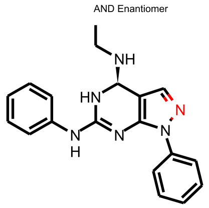 [*]:n:[*]	0.0441

Top Features for negative contribution

**Fingerprint**	**Bit/Smiles**	**Feature Structure**	**Score**
FCFP_2	203677720	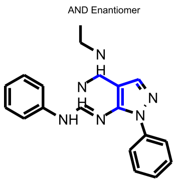 [*]C([*])[c]1:[cH]:[*]:[*]:[c]:1[*]	-0.0829
FCFP_2	1	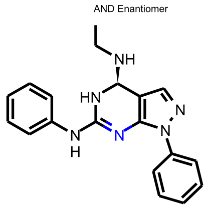 [*]N=[*]	-0.0796
FCFP_2	16	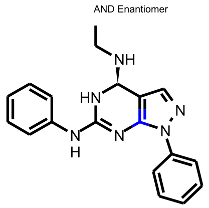 [*][c](:[*]):[*]	-0.0512

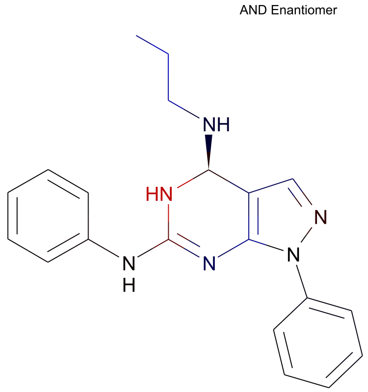

$C_{20}H_{22}N_6$

Molecular Weight: 346.42888

ALogP: 3.562

Rotatable Bonds: 6

Acceptors: 5

Donors: 3

Model Prediction

Prediction: 0.401

Unit: g/kg_body_weight

Mahalanobis Distance: 11.5

Mahalanobis Distance p-value: 9.08e-008

Mahalanobis Distance: The Mahalanobis distance (MD) is a generalization of the Euclidean distance that accounts for correlations among the X properties. It is calculated as the distance to the center of the training data. The larger the MD, the less trustworthy the prediction.
Mahalanobis Distance p-value: The p-value gives the fraction of training data with an MD greater than or equal to the one for the given sample, assuming normally distributed data. The smaller the p-value, the less trustworthy the prediciton. For highly non-normal X properties (e.g., fingerprints), the MD p-value is wildly inaccurate.

Structural Similar Compounds

Name	DISPERSE YELLOW 3	PHENOLPHTHALEIN	BENZIDINE,3,3'-DIMETHOXY-
Structure	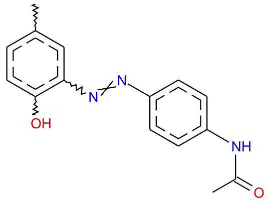	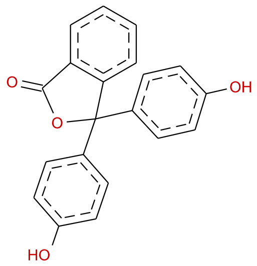	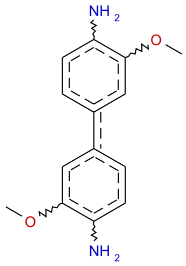
Actual Endpoint (-log C)	2.77703	2.20184	4.06569
Predicted Endpoint (-log C)	2.80195	2.8857	3.57405
Distance	0.688	0.700	0.786
Reference	NCI/NTP TR-222	NCI/NTP TR-465	NCI/NTP Report 10, Nov. 1987

Model Applicability

Unknown features are fingerprint features in the query molecule, but not found or appearing too infrequently in the training set.

1. All properties and OPS components are within expected ranges.
2. Unknown FCFP_2 feature: 179977000: [*][c]1:[*]:[*]:n:n:1[c](:[*]):[*]
3. Unknown FCFP_2 feature: 2119857014: [*]NC(N[*])[c](:[*]):[*]
4. Unknown FCFP_2 feature: -1732563065: [*]:n(:[*])[c](:c:[*]):c:[*]

Feature Contribution

Top features for positive contribution

Fingerprint	Bit/Smiles	Feature Structure	Score
FCFP_2	-885550502	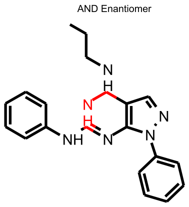 [*]C([*])NC(=[*])[*]	0.115

FCFP_2	3	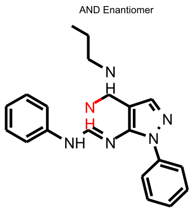 [*]N[*]	0.0737
FCFP_2	17	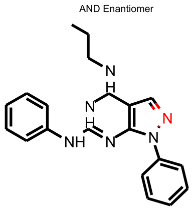 [*]:n:[*]	0.0441

Top Features for negative contribution

Fingerprint	Bit/Smiles	Feature Structure	Score
FCFP_2	-1272798659	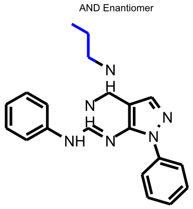 [*]CCC	-0.111
FCFP_2	203677720	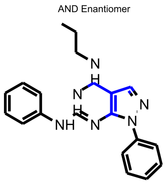 [*]C([*])[c]1:[cH]:[*]:[*]:[c]:1[*]	-0.0829
FCFP_2	1	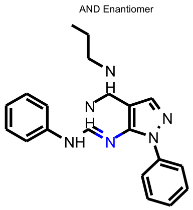 [*]N=[*]	-0.0796

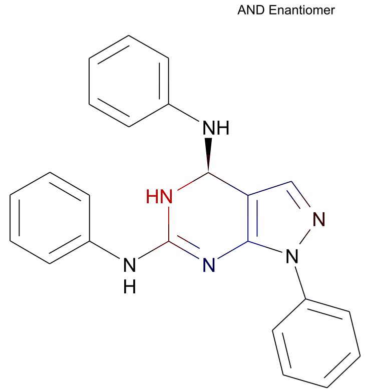

$C_{23}H_{20}N_6$
Molecular Weight: 380.4451
ALogP: 4.544
Rotatable Bonds: 5
Acceptors: 5
Donors: 3

Model Prediction

Prediction: 0.266
Unit: g/kg_body_weight
Mahalanobis Distance: 11.4
Mahalanobis Distance p-value: 1.75e-007

Mahalanobis Distance: The Mahalanobis distance (MD) is a generalization of the Euclidean distance that accounts for correlations among the X properties. It is calculated as the distance to the center of the training data. The larger the MD, the less trustworthy the prediction.
Mahalanobis Distance p-value: The p-value gives the fraction of training data with an MD greater than or equal to the one for the given sample, assuming normally distributed data. The smaller the p-value, the less trustworthy the prediciton. For highly non-normal X properties (e.g., fingerprints), the MD p-value is wildly inaccurate.

Structural Similar Compounds

Name	PHENOLPHTHALEIN	C.I.PIGMENT RED 3	DISPERSE YELLOW 3
Structure	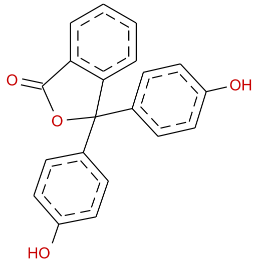	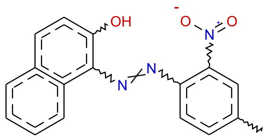	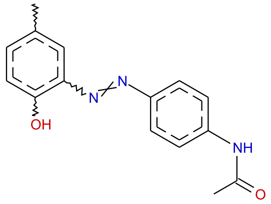
Actual Endpoint (-log C)	2.20184	2.65635	2.77703
Predicted Endpoint (-log C)	2.8857	2.97957	2.80195
Distance	0.740	0.838	0.861
Reference	NCI/NTP TR-465	NCI/NTP TR-407	NCI/NTP TR-222

Model Applicability

Unknown features are fingerprint features in the query molecule, but not found or appearing too infrequently in the training set.

1. All properties and OPS components are within expected ranges.
2. Unknown FCFP_2 feature: 179977000: [*][c]1:[*]:[*]:n:n:1[c](:[*]):[*]
3. Unknown FCFP_2 feature: 2119857014: [*]NC(N[*])[c](:[*]):[*]
4. Unknown FCFP_2 feature: -1732563065: [*]:n(:[*])[c](:c:[*]):c:[*]

Feature Contribution

Top features for positive contribution

Fingerprint	Bit/Smiles	Feature Structure	Score
FCFP_2	-885550502	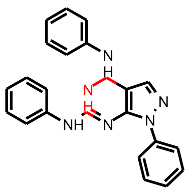 AND Enantiomer [*]C([*])NC(=[*])[*]	0.115

FCFP_2	3	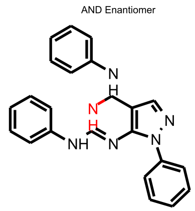 [*]N[*]	0.0737
FCFP_2	17	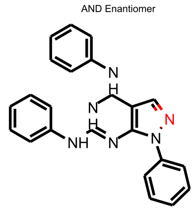 [*]:n:[*]	0.0441
**Top Features for negative contribution**			
**Fingerprint**	**Bit/Smiles**	**Feature Structure**	**Score**
FCFP_2	203677720	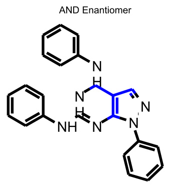 [*]C([*])[c]1:[cH]:[*]:[*]:[c]:1[*]	-0.0829
FCFP_2	1	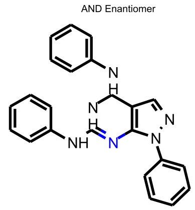 [*]N=[*]	-0.0796
FCFP_2	16	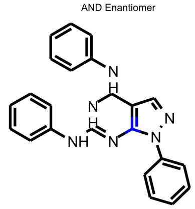 [*][c](:[*]):[*]	-0.0512

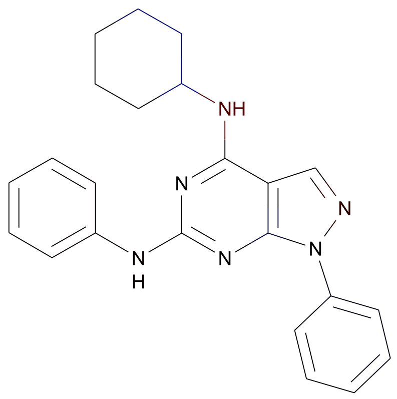

$C_{23}H_{24}N_6$
Molecular Weight: 384.47686
ALogP: 5.953
Rotatable Bonds: 5
Acceptors: 5
Donors: 2

Model Prediction

Prediction: 0.213
Unit: g/kg_body_weight
Mahalanobis Distance: 10.7
Mahalanobis Distance p-value: 2.75e-006

Mahalanobis Distance: The Mahalanobis distance (MD) is a generalization of the Euclidean distance that accounts for correlations among the X properties. It is calculated as the distance to the center of the training data. The larger the MD, the less trustworthy the prediction.
Mahalanobis Distance p-value: The p-value gives the fraction of training data with an MD greater than or equal to the one for the given sample, assuming normally distributed data. The smaller the p-value, the less trustworthy the prediciton. For highly non-normal X properties (e.g., fingerprints), the MD p-value is wildly inaccurate.

Structural Similar Compounds

Name	C.I.PIGMENT RED 3	PHENOLPHTHALEIN	ANILAZINE
Structure	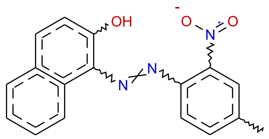	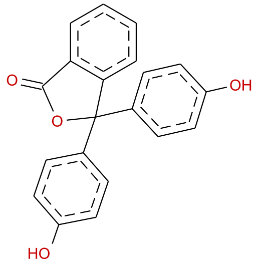	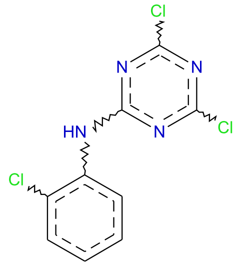
Actual Endpoint (-log C)	2.65635	2.20184	3.78694
Predicted Endpoint (-log C)	2.97957	2.8857	3.42114
Distance	0.777	0.796	0.869
Reference	NCI/NTP TR-407	NCI/NTP TR-465	NCI/NTP TR-104

Model Applicability

Unknown features are fingerprint features in the query molecule, but not found or appearing too infrequently in the training set.

1. OPS PC9 out of range. Value: 3.74. Training min, max, SD, explained variance: -2.8548, 3.3954, 1.263, 0.0360.
2. Unknown FCFP_2 feature: 179977000: [*][c]1:[*]:[*]:n:n:1[c](:[*]):[*]
3. Unknown FCFP_2 feature: -1732563065: [*]:n(:[*])[c](:c:[*]):c:[*]

Feature Contribution

Top features for positive contribution

Fingerprint	Bit/Smiles	Feature Structure	Score
FCFP_2	3	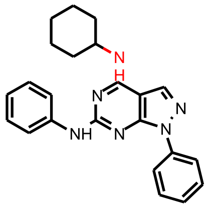 [*]N[*]	0.0737

FCFP_2	17	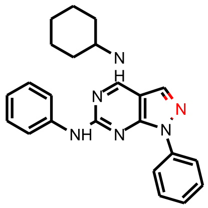<br>[*]:n:[*]	0.0441
FCFP_2	590925877	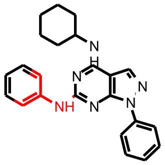<br>[*]N[c](:[cH]:[*]):[c<br>H]:[*]	0.00762

Top Features for negative contribution

**Fingerprint**	**Bit/Smiles**	**Feature Structure**	**Score**
FCFP_2	-1272798659	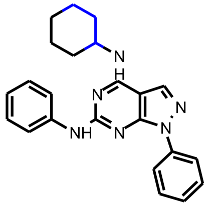<br>[*]CCC	-0.111
FCFP_2	16	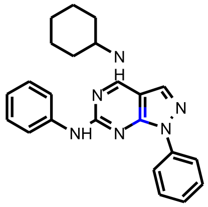<br>[*][c](:[*]):[*]	-0.0512
FCFP_2	0	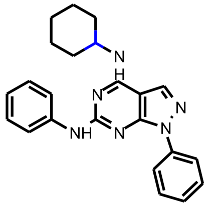<br>[*]C(=[*])[*]	-0.0314

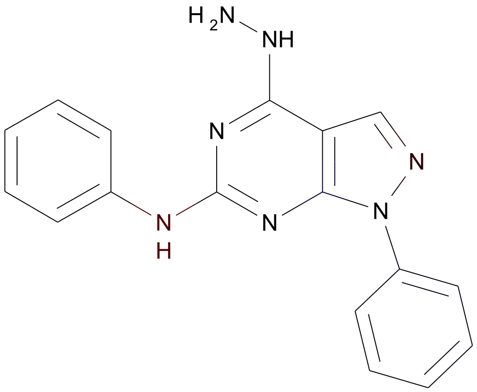

$C_{17}H_{15}N_7$
Molecular Weight: 317.3479
ALogP: 3.562
Rotatable Bonds: 4
Acceptors: 6
Donors: 3

Model Prediction

Prediction: 0.291
Unit: g/kg_body_weight
Mahalanobis Distance: 8.26
Mahalanobis Distance p-value: 0.0125

Mahalanobis Distance: The Mahalanobis distance (MD) is a generalization of the Euclidean distance that accounts for correlations among the X properties. It is calculated as the distance to the center of the training data. The larger the MD, the less trustworthy the prediction.
Mahalanobis Distance p-value: The p-value gives the fraction of training data with an MD greater than or equal to the one for the given sample, assuming normally distributed data. The smaller the p-value, the less trustworthy the prediciton. For highly non-normal X properties (e.g., fingerprints), the MD p-value is wildly inaccurate.

Structural Similar Compounds

Name	TRIAMTERENE	PHENOLPHTHALEIN	SALICYLAZOSULFAPYRIDINE
Structure	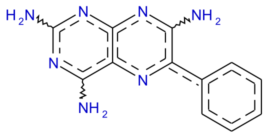	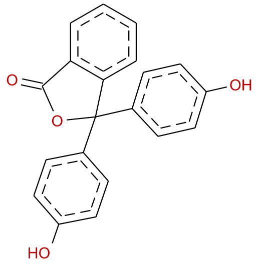	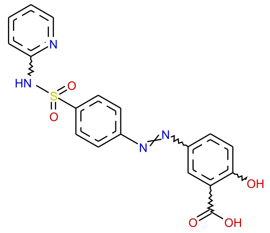
Actual Endpoint (-log C)	4.00564	2.20184	3.375
Predicted Endpoint (-log C)	3.1992	2.8857	2.80292
Distance	0.764	0.821	0.828
Reference	NCI/NTP TR-420	NCI/NTP TR-465	NCI/NTP TR-457

Model Applicability

Unknown features are fingerprint features in the query molecule, but not found or appearing too infrequently in the training set.

1. All properties and OPS components are within expected ranges.
2. Unknown FCFP_2 feature: 179977000: [*][c]1:[*]:[*]:n:n:1[c](:[*]):[*]
3. Unknown FCFP_2 feature: -1732563065: [*]:n(:[*])[c](:c:[*]):c:[*]
4. Unknown FCFP_2 feature: 1070150408: [*]NN

Feature Contribution

Top features for positive contribution

Fingerprint	Bit/Smiles	Feature Structure	Score
FCFP_2	3	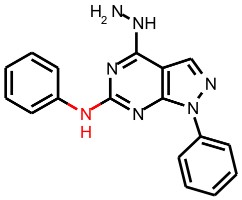 [*]N[*]	0.0737

FCFP_2	17	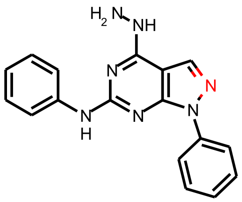 [*]:n:[*]	0.0441
FCFP_2	590925877	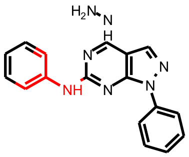 [*]N[c](:[cH]:[*]):[cH]:[*]	0.00762
**Top Features for negative contribution**			
**Fingerprint**	**Bit/Smiles**	**Feature Structure**	**Score**
FCFP_2	16	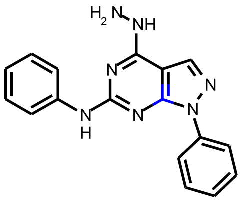 [*][c](:[*]):[*]	-0.0512
FCFP_2	1618154665	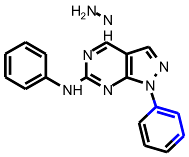 [*][c](:[*]):[cH]:[cH]:[*]	-0.0165

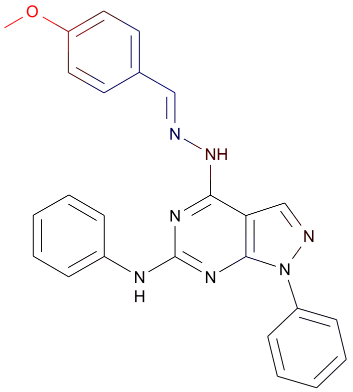

$C_{25}H_{21}N_7O$
Molecular Weight: 435.48054
ALogP: 5.889
Rotatable Bonds: 7
Acceptors: 7
Donors: 2

Model Prediction

Prediction: 0.152
Unit: g/kg_body_weight
Mahalanobis Distance: 10.3
Mahalanobis Distance p-value: 1.32e-005

Mahalanobis Distance: The Mahalanobis distance (MD) is a generalization of the Euclidean distance that accounts for correlations among the X properties. It is calculated as the distance to the center of the training data. The larger the MD, the less trustworthy the prediction.
Mahalanobis Distance p-value: The p-value gives the fraction of training data with an MD greater than or equal to the one for the given sample, assuming normally distributed data. The smaller the p-value, the less trustworthy the prediciton. For highly non-normal X properties (e.g., fingerprints), the MD p-value is wildly inaccurate.

Structural Similar Compounds

Name	C.I.PIGMENT RED 23	C.I.PIGMENT RED 3	SALICYLAZOSULFAPYRIDINE
Structure	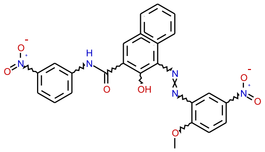	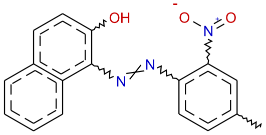	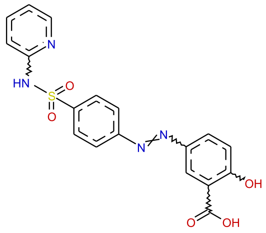
Actual Endpoint (-log C)	2.30052	2.65635	3.375
Predicted Endpoint (-log C)	3.55333	2.97957	2.80292
Distance	0.939	0.957	0.977
Reference	NCI/NTP TR-411	NCI/NTP TR-407	NCI/NTP TR-457

Model Applicability

Unknown features are fingerprint features in the query molecule, but not found or appearing too infrequently in the training set.

1. Num_AromaticRings out of range. Value: 5. Training min, max, mean, SD: 0, 4, 1.1685, 0.8469.
2. OPS PC9 out of range. Value: 3.8835. Training min, max, SD, explained variance: -2.8548, 3.3954, 1.263, 0.0360.
3. Unknown FCFP_2 feature: 179977000: [*][c]1:[*]:[*]:n:n:1[c](:[*]):[*]
4. Unknown FCFP_2 feature: 1294285001: [*]=NN[c](:[*]):[*]
5. Unknown FCFP_2 feature: -1732563065: [*]:n(:[*])[c](:c:[*]):c:[*]

Feature Contribution

Top features for positive contribution

Fingerprint	Bit/Smiles	Feature Structure	Score
FCFP_2	136627117	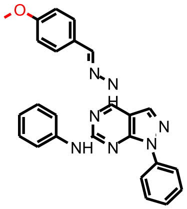 [*]OC	0.173

FCFP_2	1036089772	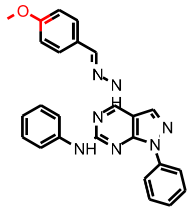 [*]:[c](:[*])OC	0.0749
FCFP_2	3	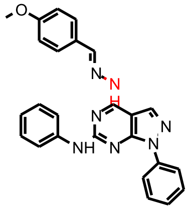 [*]N[*]	0.0737
**Top Features for negative contribution**			
**Fingerprint**	**Bit/Smiles**	**Feature Structure**	**Score**
FCFP_2	203677720	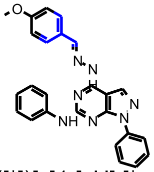 [*]C([*])[c]1:[cH]:[*]:[*]:[c]:1[*]	-0.0829
FCFP_2	1	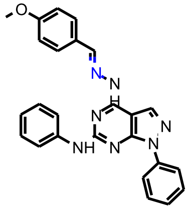 [*]N=[*]	-0.0796
FCFP_2	16	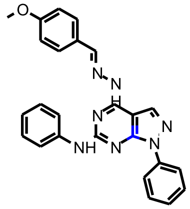 [*][c](:[*]):[*]	-0.0512

11b TOPKAT_Rat_Maximum_Tolerated_Dose_Feed

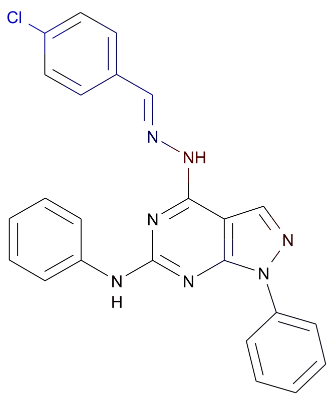

$C_{24}H_{18}ClN_7$
Molecular Weight: 439.89962
ALogP: 6.57
Rotatable Bonds: 6
Acceptors: 6
Donors: 2

Model Prediction

Prediction: 0.391
Unit: g/kg_body_weight
Mahalanobis Distance: 11.7
Mahalanobis Distance p-value: 4.87e-008

Mahalanobis Distance: The Mahalanobis distance (MD) is a generalization of the Euclidean distance that accounts for correlations among the X properties. It is calculated as the distance to the center of the training data. The larger the MD, the less trustworthy the prediction.
Mahalanobis Distance p-value: The p-value gives the fraction of training data with an MD greater than or equal to the one for the given sample, assuming normally distributed data. The smaller the p-value, the less trustworthy the prediciton. For highly non-normal X properties (e.g., fingerprints), the MD p-value is wildly inaccurate.

Structural Similar Compounds

Name	C.I.PIGMENT RED 3	PHENOLPHTHALEIN	SALICYLAZOSULFAPYRIDINE
Structure	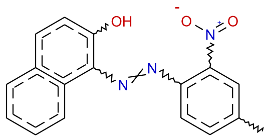	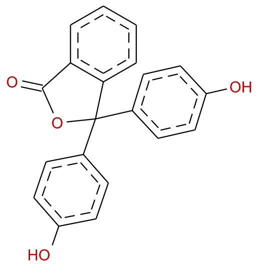	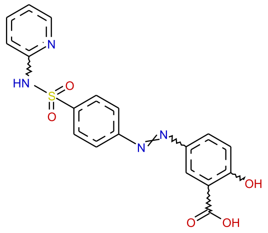
Actual Endpoint (-log C)	2.65635	2.20184	3.375
Predicted Endpoint (-log C)	2.97957	2.8857	2.80292
Distance	0.956	1.009	1.056
Reference	NCI/NTP TR-407	NCI/NTP TR-465	NCI/NTP TR-457

Model Applicability

Unknown features are fingerprint features in the query molecule, but not found or appearing too infrequently in the training set.

1. Num_AromaticRings out of range. Value: 5. Training min, max, mean, SD: 0, 4, 1.1685, 0.8469.
2. Unknown FCFP_2 feature: 179977000: [*][c]1:[*]:[*]:n:n:1[c](:[*]):[*]
3. Unknown FCFP_2 feature: 1294285001: [*]=NN[c](:[*]):[*]
4. Unknown FCFP_2 feature: -1732563065: [*]:n(:[*])[c](:c:[*]):c:[*]

Feature Contribution

Top features for positive contribution

Fingerprint	Bit/Smiles	Feature Structure	Score
FCFP_2	3	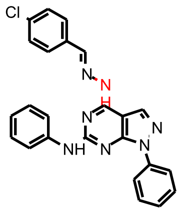 [*]N[*]	0.0737

FCFP_2	17	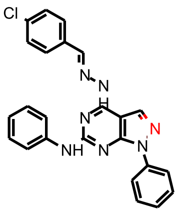 [*]:n:[*]	0.0441
FCFP_2	590925877	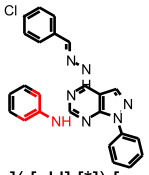 [*]N[c](:[cH]:[*]):[cH]:[*]	0.00762
**Top Features for negative contribution**			
**Fingerprint**	**Bit/Smiles**	**Feature Structure**	**Score**
FCFP_2	71476542	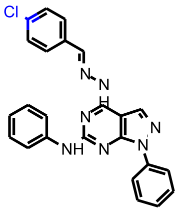 [*]:[c](:[*])Cl	-0.134
FCFP_2	203677720	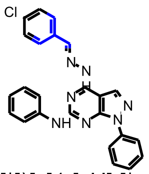 [*]C([*])[c]1:[cH]:[*]:[*]:[c]:1[*]	-0.0829
FCFP_2	1	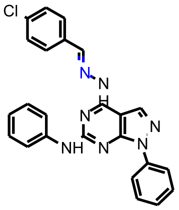 [*]N=[*]	-0.0796

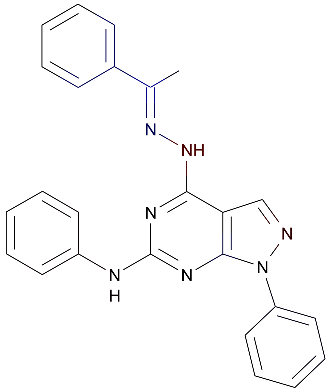

$C_{25}H_{21}N_7$

Molecular Weight: 419.48114

ALogP: 5.886

Rotatable Bonds: 6

Acceptors: 6

Donors: 2

Model Prediction

Prediction: 0.294

Unit: g/kg_body_weight

Mahalanobis Distance: 11.3

Mahalanobis Distance p-value: 2.89e-007

Mahalanobis Distance: The Mahalanobis distance (MD) is a generalization of the Euclidean distance that accounts for correlations among the X properties. It is calculated as the distance to the center of the training data. The larger the MD, the less trustworthy the prediction.
Mahalanobis Distance p-value: The p-value gives the fraction of training data with an MD greater than or equal to the one for the given sample, assuming normally distributed data. The smaller the p-value, the less trustworthy the prediciton. For highly non-normal X properties (e.g., fingerprints), the MD p-value is wildly inaccurate.

Structural Similar Compounds

Name	C.I.PIGMENT RED 3	PHENOLPHTHALEIN	SALICYLAZOSULFAPYRIDINE
Structure	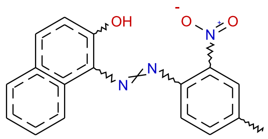	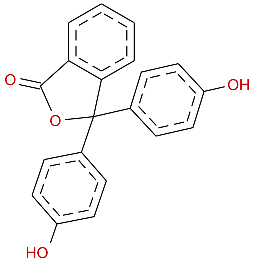	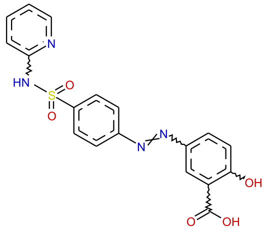
Actual Endpoint (-log C)	2.65635	2.20184	3.375
Predicted Endpoint (-log C)	2.97957	2.8857	2.80292
Distance	0.927	0.971	1.022
Reference	NCI/NTP TR-407	NCI/NTP TR-465	NCI/NTP TR-457

Model Applicability

Unknown features are fingerprint features in the query molecule, but not found or appearing too infrequently in the training set.

1. Num_AromaticRings out of range. Value: 5. Training min, max, mean, SD: 0, 4, 1.1685, 0.8469.
2. Unknown FCFP_2 feature: 179977000: [*][c]1:[*]:[*]:n:n:1[c](:[*]):[*]
3. Unknown FCFP_2 feature: 1294285001: [*]=NN[c](:[*]):[*]
4. Unknown FCFP_2 feature: -1732563065: [*]:n(:[*])[c](:c:[*]):c:[*]

Feature Contribution

Top features for positive contribution

Fingerprint	Bit/Smiles	Feature Structure	Score
FCFP_2	3	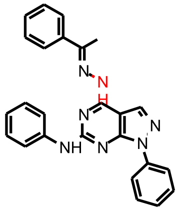 [*]N[*]	0.0737

FCFP_2	17	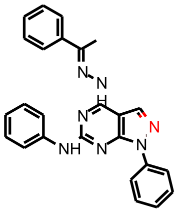<br>[*]:n:[*]	0.0441
FCFP_2	590925877	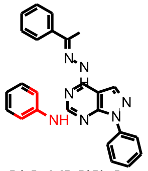<br>[*]N[c](:[cH]:[*]):[cH]:[*]	0.00762
**Top Features for negative contribution**			
**Fingerprint**	**Bit/Smiles**	**Feature Structure**	**Score**
FCFP_2	203677720	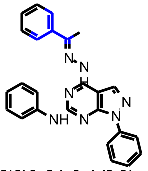<br>[*]C([*])[c]1:[cH]:[*]:[*]:[c]:1[*]	-0.0829
FCFP_2	1	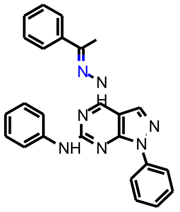<br>[*]N=[*]	-0.0796
FCFP_2	16	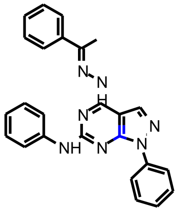<br>[*][c](:[*]):[*]	-0.0512

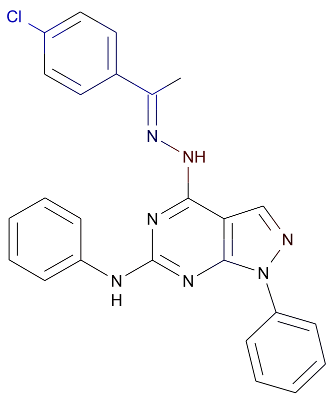

$C_{25}H_{20}ClN_7$
Molecular Weight: 453.9262
ALogP: 6.55
Rotatable Bonds: 6
Acceptors: 6
Donors: 2

Model Prediction

Prediction: 0.358
Unit: g/kg_body_weight
Mahalanobis Distance: 12.5
Mahalanobis Distance p-value: 1.67e-009

Mahalanobis Distance: The Mahalanobis distance (MD) is a generalization of the Euclidean distance that accounts for correlations among the X properties. It is calculated as the distance to the center of the training data. The larger the MD, the less trustworthy the prediction.
Mahalanobis Distance p-value: The p-value gives the fraction of training data with an MD greater than or equal to the one for the given sample, assuming normally distributed data. The smaller the p-value, the less trustworthy the prediciton. For highly non-normal X properties (e.g., fingerprints), the MD p-value is wildly inaccurate.

Structural Similar Compounds

Name	C.I.PIGMENT RED 3	PHENOLPHTHALEIN	SALICYLAZOSULFAPYRIDINE
Structure	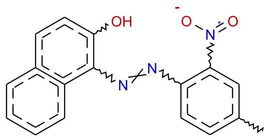	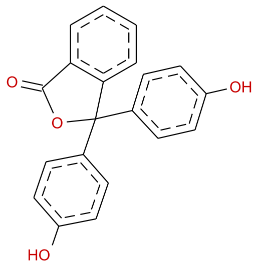	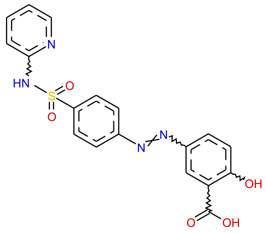
Actual Endpoint (-log C)	2.65635	2.20184	3.375
Predicted Endpoint (-log C)	2.97957	2.8857	2.80292
Distance	0.969	1.019	1.062
Reference	NCI/NTP TR-407	NCI/NTP TR-465	NCI/NTP TR-457

Model Applicability

Unknown features are fingerprint features in the query molecule, but not found or appearing too infrequently in the training set.

1. Num_AromaticRings out of range. Value: 5. Training min, max, mean, SD: 0, 4, 1.1685, 0.8469.
2. Unknown FCFP_2 feature: 179977000: [*][c]1:[*]:[*]:n:n:1[c](:[*]):[*]
3. Unknown FCFP_2 feature: 1294285001: [*]=NN[c](:[*]):[*]
4. Unknown FCFP_2 feature: -1732563065: [*]:n(:[*])[c](:c:[*]):c:[*]

Feature Contribution

Top features for positive contribution

Fingerprint	Bit/Smiles	Feature Structure	Score
FCFP_2	3	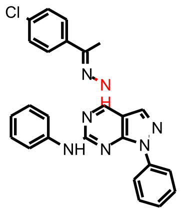 [*]N[*]	0.0737

FCFP_2	17	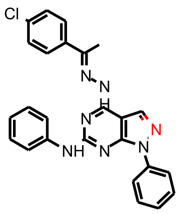 [*]:n:[*]	0.0441
FCFP_2	590925877	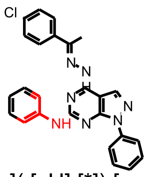 [*]N[c](:[cH]:[*]):[cH]:[*]	0.00762

Top Features for negative contribution

Fingerprint	Bit/Smiles	Feature Structure	Score
FCFP_2	71476542	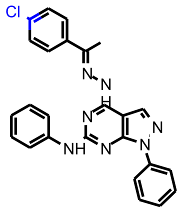 [*]:[c](:[*])Cl	-0.134
FCFP_2	203677720	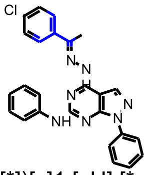 [*]C([*])[c]1:[cH]:[*]:[*]:[c]:1[*]	-0.0829
FCFP_2	1	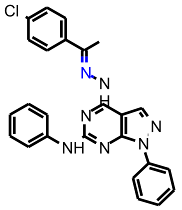 [*]N=[*]	-0.0796

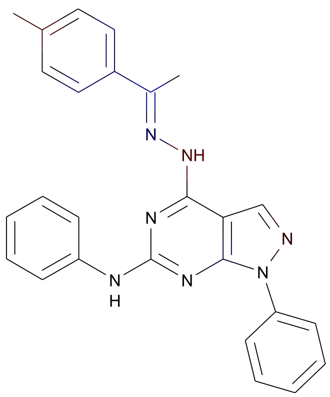

$C_{26}H_{23}N_7$

Molecular Weight: 433.50772

ALogP: 6.372

Rotatable Bonds: 6

Acceptors: 6

Donors: 2

Model Prediction

Prediction: 0.242

Unit: g/kg_body_weight

Mahalanobis Distance: 11.6

Mahalanobis Distance p-value: 6.27e-008

Mahalanobis Distance: The Mahalanobis distance (MD) is a generalization of the Euclidean distance that accounts for correlations among the X properties. It is calculated as the distance to the center of the training data. The larger the MD, the less trustworthy the prediction.
Mahalanobis Distance p-value: The p-value gives the fraction of training data with an MD greater than or equal to the one for the given sample, assuming normally distributed data. The smaller the p-value, the less trustworthy the prediciton. For highly non-normal X properties (e.g., fingerprints), the MD p-value is wildly inaccurate.

Structural Similar Compounds

Name	C.I.PIGMENT RED 3	PHENOLPHTHALEIN	SALICYLAZOSULFAPYRIDINE
Structure	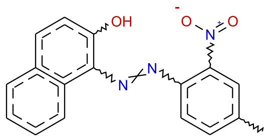	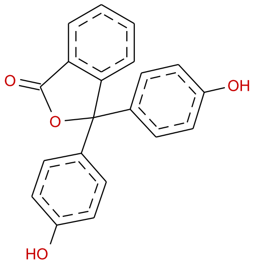	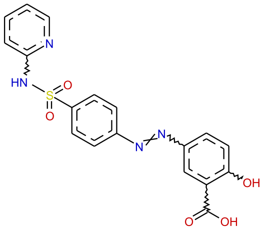
Actual Endpoint (-log C)	2.65635	2.20184	3.375
Predicted Endpoint (-log C)	2.97957	2.8857	2.80292
Distance	0.926	0.996	1.044
Reference	NCI/NTP TR-407	NCI/NTP TR-465	NCI/NTP TR-457

Model Applicability

Unknown features are fingerprint features in the query molecule, but not found or appearing too infrequently in the training set.

1. Num_AromaticRings out of range. Value: 5. Training min, max, mean, SD: 0, 4, 1.1685, 0.8469.
2. OPS PC8 out of range. Value: 3.9303. Training min, max, SD, explained variance: -3.8548, 3.9137, 1.331, 0.0400.
3. Unknown FCFP_2 feature: 179977000: [*][c]1:[*]:[*]:n:n:1[c](:[*]):[*]
4. Unknown FCFP_2 feature: 1294285001: [*]=NN[c](:[*]):[*]
5. Unknown FCFP_2 feature: -1732563065: [*]:n(:[*])[c](:c:[*]):c:[*]

Feature Contribution

Top features for positive contribution

Fingerprint	Bit/Smiles	Feature Structure	Score
FCFP_2	3	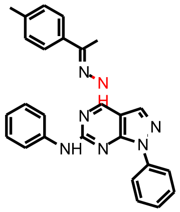 [*]N[*]	0.0737

FCFP_2	136120670	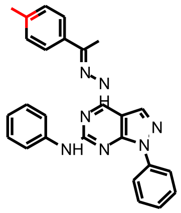 [*]:[c](:[*])C	0.064
FCFP_2	17	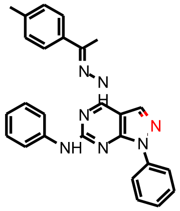 [*]:n:[*]	0.0441

Top Features for negative contribution

**Fingerprint**	**Bit/Smiles**	**Feature Structure**	**Score**
FCFP_2	203677720	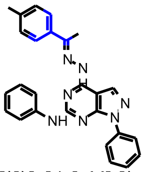 [*]C([*])[c]1:[cH]:[*<br>]:[*]:[c]:1[*]	-0.0829
FCFP_2	1	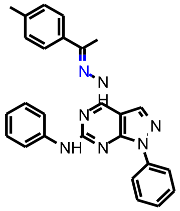 [*]N=[*]	-0.0796
FCFP_2	16	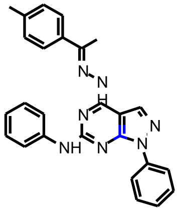 [*][c](:[*]):[*]	-0.0512

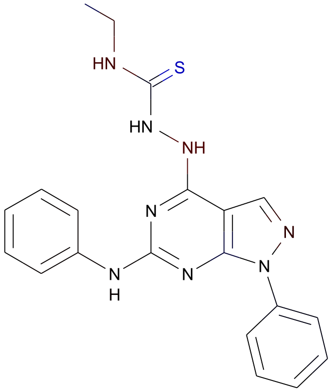

$C_{20}H_{20}N_8S$
Molecular Weight: 404.4914
ALogP: 4.99
Rotatable Bonds: 8
Acceptors: 6
Donors: 4

Model Prediction

Prediction: 0.53
Unit: g/kg_body_weight
Mahalanobis Distance: 12.1
Mahalanobis Distance p-value: 7.52e-009

Mahalanobis Distance: The Mahalanobis distance (MD) is a generalization of the Euclidean distance that accounts for correlations among the X properties. It is calculated as the distance to the center of the training data. The larger the MD, the less trustworthy the prediction.
Mahalanobis Distance p-value: The p-value gives the fraction of training data with an MD greater than or equal to the one for the given sample, assuming normally distributed data. The smaller the p-value, the less trustworthy the prediciton. For highly non-normal X properties (e.g., fingerprints), the MD p-value is wildly inaccurate.

Structural Similar Compounds

Name	SALICYLAZOSULFAPYRIDINE	C.I.PIGMENT RED 23	FUROSEMIDE
Structure	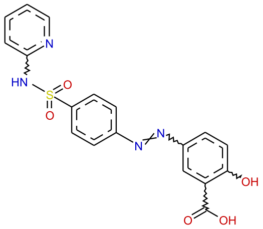	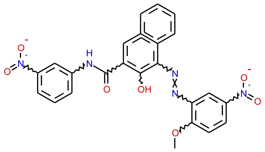	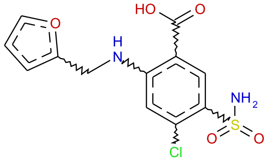
Actual Endpoint (-log C)	3.375	2.30052	4.04236
Predicted Endpoint (-log C)	2.80292	3.55333	2.8614
Distance	0.731	0.887	0.959
Reference	NCI/NTP TR-457	NCI/NTP TR-411	NCI/NTP TR-356

Model Applicability

Unknown features are fingerprint features in the query molecule, but not found or appearing too infrequently in the training set.

1. All properties and OPS components are within expected ranges.
2. Unknown FCFP_2 feature: 179977000: [*][c]1:[*]:[*]:n:n:1[c](:[*]):[*]
3. Unknown FCFP_2 feature: -1732563065: [*]:n(:[*])[c](:c:[*]):c:[*]
4. Unknown FCFP_2 feature: -885461129: [*]NNC(=[*])[*]

Feature Contribution

Top features for positive contribution

Fingerprint	Bit/Smiles	Feature Structure	Score
FCFP_2	-885550502	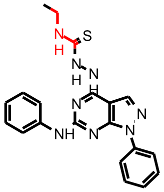 [*]C([*])NC(=[*])[*]	0.115

FCFP_2	3	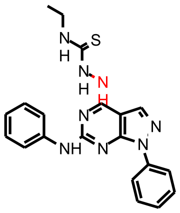 [*]N[*]	0.0737
FCFP_2	17	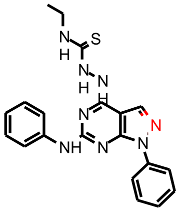 [*]:n:[*]	0.0441
**Top Features for negative contribution**			
**Fingerprint**	**Bit/Smiles**	**Feature Structure**	**Score**
FCFP_2	1872154524	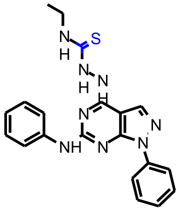 [*]C(=S)[*]	-0.105
FCFP_2	1	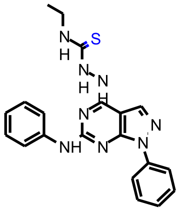 [*]N=[*]	-0.0796
FCFP_2	16	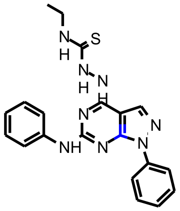 [*][c](:[*]):[*]	-0.0512

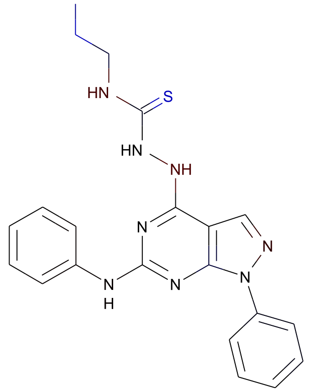

$C_{21}H_{22}N_8S$

Molecular Weight: 418.51798

ALogP: 5.514

Rotatable Bonds: 9

Acceptors: 6

Donors: 4

Model Prediction

Prediction: 0.735

Unit: g/kg_body_weight

Mahalanobis Distance: 12.6

Mahalanobis Distance p-value: 8.68e-010

Mahalanobis Distance: The Mahalanobis distance (MD) is a generalization of the Euclidean distance that accounts for correlations among the X properties. It is calculated as the distance to the center of the training data. The larger the MD, the less trustworthy the prediction.
Mahalanobis Distance p-value: The p-value gives the fraction of training data with an MD greater than or equal to the one for the given sample, assuming normally distributed data. The smaller the p-value, the less trustworthy the prediciton. For highly non-normal X properties (e.g., fingerprints), the MD p-value is wildly inaccurate.

Structural Similar Compounds

Name	SALICYLAZOSULFAPYRIDINE	C.I.PIGMENT RED 23	FUROSEMIDE
Structure	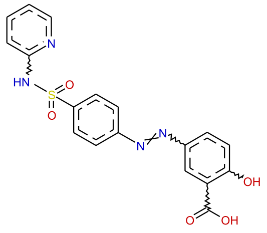	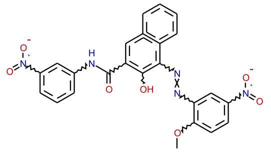	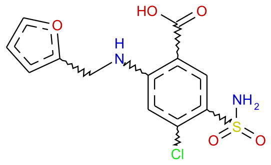
Actual Endpoint (-log C)	3.375	2.30052	4.04236
Predicted Endpoint (-log C)	2.80292	3.55333	2.8614
Distance	0.774	0.892	1.011
Reference	NCI/NTP TR-457	NCI/NTP TR-411	NCI/NTP TR-356

Model Applicability

Unknown features are fingerprint features in the query molecule, but not found or appearing too infrequently in the training set.

1. OPS PC12 out of range. Value: -2.7568. Training min, max, SD, explained variance: -2.364, 2.9228, 1.079, 0.0263.
2. Unknown FCFP_2 feature: 179977000: [*][c]1:[*]:[*]:n:n:1[c](:[*]):[*]
3. Unknown FCFP_2 feature: -1732563065: [*]:n(:[*])[c](:c:[*]):c:[*]
4. Unknown FCFP_2 feature: -885461129: [*]NNC(=[*])[*]

Feature Contribution

Top features for positive contribution

Fingerprint	Bit/Smiles	Feature Structure	Score
FCFP_2	-885550502	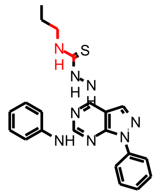 [*]C([*])NC(=[*])[*]	0.115

FCFP_2	3	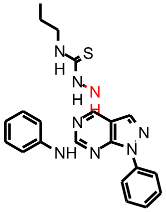<br>[*]N[*]	0.0737
FCFP_2	17	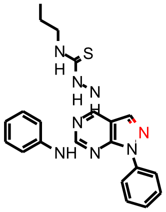<br>[*]:n:[*]	0.0441

Top Features for negative contribution

Fingerprint	Bit/Smiles	Feature Structure	Score
FCFP_2	-1272798659	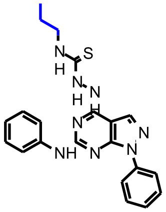<br>[*]CCC	-0.111
FCFP_2	1872154524	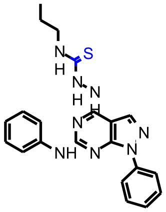<br>[*]C(=S)[*]	-0.105
FCFP_2	1	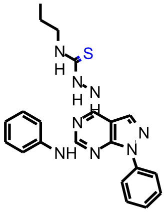<br>[*]N=[*]	-0.0796

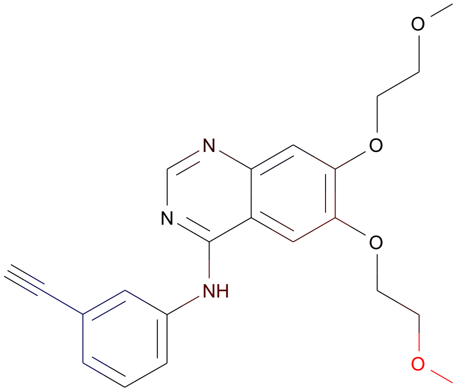

$C_{22}H_{23}N_3O_4$

Molecular Weight: 393.43572

ALogP: 4.309

Rotatable Bonds: 10

Acceptors: 7

Donors: 1

Structural Similar Compounds

Name	BUTYL BENZYL PHTHALATE	3,3'-DIMETHOXYBENZIDINE-4,4'-DIISOCYANATE	PYRILAMINE
Structure	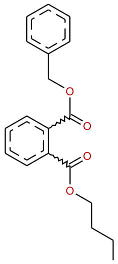	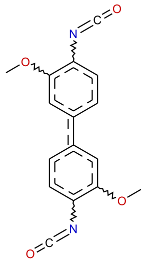	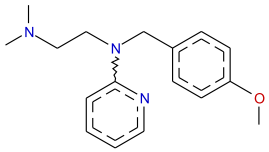
Actual Endpoint (-log C)	2.79569	2.17504	3.32511
Predicted Endpoint (-log C)	3.18498	3.78717	3.65163
Distance	0.758	0.795	0.820
Reference	NCI/NTP TR-458	NCI/NTP TR-128	NCI/NTP Report 10, Nov. 1987

Model Prediction

Prediction: 0.0828

Unit: g/kg_body_weight

Mahalanobis Distance: 9.37

Mahalanobis Distance p-value: 0.000417

Mahalanobis Distance: The Mahalanobis distance (MD) is a generalization of the Euclidean distance that accounts for correlations among the X properties. It is calculated as the distance to the center of the training data. The larger the MD, the less trustworthy the prediction.

Mahalanobis Distance p-value: The p-value gives the fraction of training data with an MD greater than or equal to the one for the given sample, assuming normally distributed data. The smaller the p-value, the less trustworthy the prediciton. For highly non-normal X properties (e.g., fingerprints), the MD p-value is wildly inaccurate.

Model Applicability

Unknown features are fingerprint features in the query molecule, but not found or appearing too infrequently in the training set.

1. All properties and OPS components are within expected ranges.
2. Unknown FCFP_2 feature: -124685461: [*]:n:c:n:[*]
3. Unknown FCFP_2 feature: 902193919: [*]:[c](:[*])C#C
4. Unknown FCFP_2 feature: 131784192: [*]C#C

Feature Contribution

Top features for positive contribution

Fingerprint	Bit/Smiles	Feature Structure	Score
FCFP_2	136627117	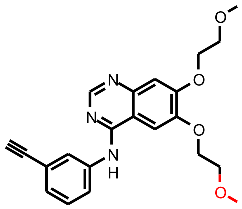 [*]OC	0.173

FCFP_2	-1143715940	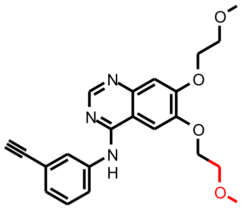 [*]COC	0.095
FCFP_2	1036089772	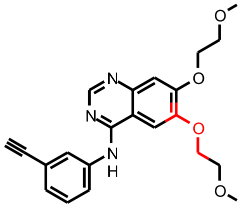 [*]:[c](:[*])OC	0.0749
**Top Features for negative contribution**			
**Fingerprint**	**Bit/Smiles**	**Feature Structure**	**Score**
FCFP_2	203677720	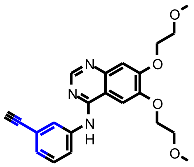 [*]C([*])[c]1:[cH]:[*]:[*]:[c]:1[*]	-0.0829
FCFP_2	1	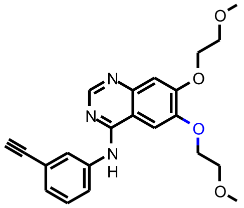 [*]N=[*]	-0.0796
FCFP_2	16	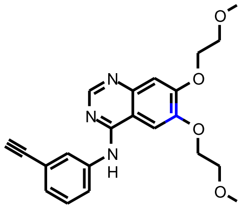 [*][c](:[*]):[*]	-0.0512

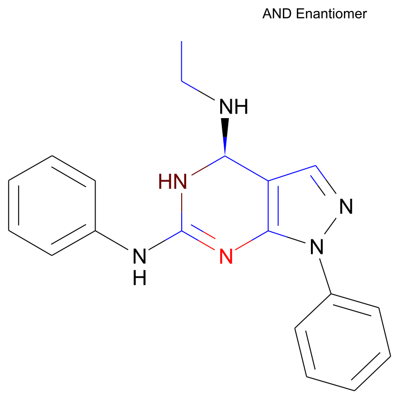

$C_{19}H_{20}N_6$

Molecular Weight: 332.4023

ALogP: 3.039

Rotatable Bonds: 5

Acceptors: 5

Donors: 3

Structural Similar Compounds

Name	SULFISOOXAZOLE	OCHRATOXIN	HC RED 3
Structure	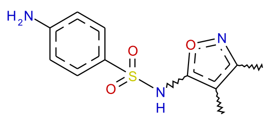	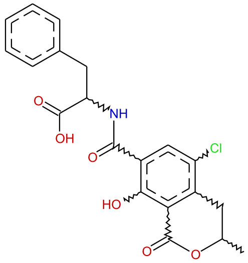	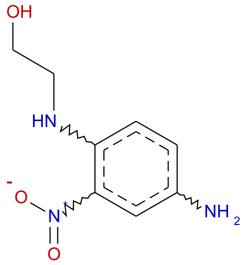
Actual Endpoint (-log C)	2.82494	6.28396	2.59592
Predicted Endpoint (-log C)	3.0705	5.12358	3.285
Distance	0.883	0.886	1.069
Reference	NCI/NTP TR-138	NCI/NTP TR-358	NCI/NTP TR-281

Model Prediction

Prediction: 0.0109

Unit: g/kg_body_weight

Mahalanobis Distance: 15.3

Mahalanobis Distance p-value: 1.83e-013

Mahalanobis Distance: The Mahalanobis distance (MD) is a generalization of the Euclidean distance that accounts for correlations among the X properties. It is calculated as the distance to the center of the training data. The larger the MD, the less trustworthy the prediction.
Mahalanobis Distance p-value: The p-value gives the fraction of training data with an MD greater than or equal to the one for the given sample, assuming normally distributed data. The smaller the p-value, the less trustworthy the prediciton. For highly non-normal X properties (e.g., fingerprints), the MD p-value is wildly inaccurate.

Model Applicability

Unknown features are fingerprint features in the query molecule, but not found or appearing too infrequently in the training set.

1. Num_AromaticRings out of range. Value: 3. Training min, max, mean, SD: 0, 2, 0.5625, 0.693.
2. Unknown FCFP_2 feature: -1410049896: [*]n1:[*]:[*]:[c]([*]):[c]:1N=[*]
3. Unknown FCFP_2 feature: 179977000: [*][c]1:[*]:[*]:n:n:1[c](:[*]):[*]
4. Unknown FCFP_2 feature: 580453787: [*]C(=N[c](:[*]):[*])[*]
5. Unknown FCFP_2 feature: 1499521844: [*]NC(=N[*])N[*]
6. Unknown FCFP_2 feature: 2119857014: [*]NC(N[*])[c](:[*]):[*]
7. Unknown FCFP_2 feature: -1732563065: [*]:n(:[*])[c](:c:[*]):c:[*]

Feature Contribution

Top features for positive contribution			
Fingerprint	Bit/Smiles	Feature Structure	Score

FCFP_2	1	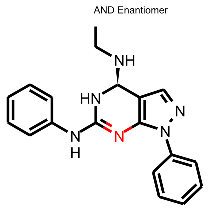 [*]N=[*]	0.511
FCFP_2	3	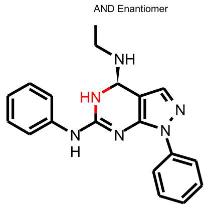 [*]N[*]	0.104

Top Features for negative contribution

Fingerprint	Bit/Smiles	Feature Structure	Score
FCFP_2	136597326	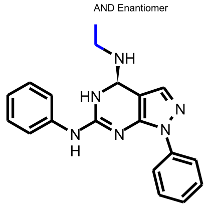 [*]CC	-0.489
FCFP_2	203677720	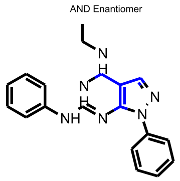 [*]C([*])[c]1:[cH]:[*]:[*]:[c]:1[*]	-0.406
FCFP_2	0	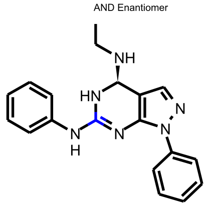 [*]C(=[*])[*]	-0.29

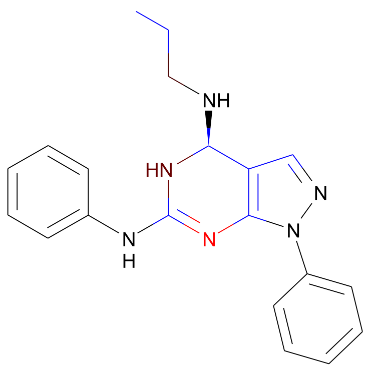

$C_{20}H_{22}N_6$

Molecular Weight: 346.42888

ALogP: 3.562

Rotatable Bonds: 6

Acceptors: 5

Donors: 3

Structural Similar Compounds

Name	OCHRATOXIN	SULFISOOXAZOLE	PROBENECID
Structure	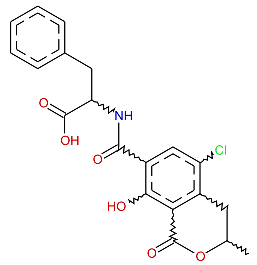	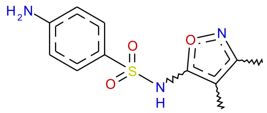	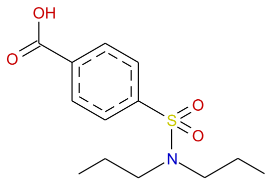
Actual Endpoint (-log C)	6.28396	2.82494	2.85333
Predicted Endpoint (-log C)	5.12358	3.0705	2.4258
Distance	0.886	0.938	1.102
Reference	NCI/NTP TR-358	NCI/NTP TR-138	NCI/NTP TR-395

Model Prediction

Prediction: 0.0103

Unit: g/kg_body_weight

Mahalanobis Distance: 15.6

Mahalanobis Distance p-value: 8.14e-014

Mahalanobis Distance: The Mahalanobis distance (MD) is a generalization of the Euclidean distance that accounts for correlations among the X properties. It is calculated as the distance to the center of the training data. The larger the MD, the less trustworthy the prediction.
Mahalanobis Distance p-value: The p-value gives the fraction of training data with an MD greater than or equal to the one for the given sample, assuming normally distributed data. The smaller the p-value, the less trustworthy the prediciton. For highly non-normal X properties (e.g., fingerprints), the MD p-value is wildly inaccurate.

Model Applicability

Unknown features are fingerprint features in the query molecule, but not found or appearing too infrequently in the training set.

1. Num_AromaticRings out of range. Value: 3. Training min, max, mean, SD: 0, 2, 0.5625, 0.693.
2. Unknown FCFP_2 feature: -1410049896: [*]n1:[*]:[*]:[c]([*]):[c]:1N=[*]
3. Unknown FCFP_2 feature: 179977000: [*][c]1:[*]:[*]:n:n:1[c](:[*]):[*]
4. Unknown FCFP_2 feature: 580453787: [*]C(=N[c](:[*]):[*])[*]
5. Unknown FCFP_2 feature: 1499521844: [*]NC(=N[*])N[*]
6. Unknown FCFP_2 feature: 2119857014: [*]NC(N[*])[c](:[*]):[*]
7. Unknown FCFP_2 feature: -1732563065: [*]:n(:[*])[c](:c:[*]):c:[*]

Feature Contribution

Top features for positive contribution			
Fingerprint	Bit/Smiles	Feature Structure	Score

FCFP_2	1	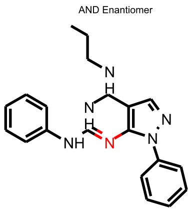<br>[*]N=[*]	0.511
FCFP_2	3	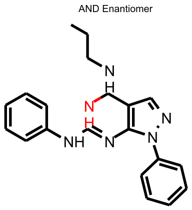<br>[*]N[*]	0.104
FCFP_2	-1272798659	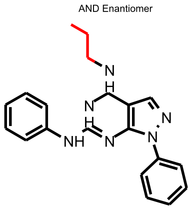<br>[*]CCC	0.0703
**Top Features for negative contribution**			
**Fingerprint**	**Bit/Smiles**	**Feature Structure**	**Score**
FCFP_2	136597326	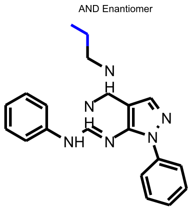<br>[*]CC	-0.489
FCFP_2	203677720	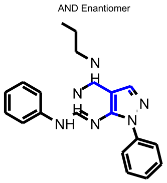<br>[*]C([*])[c]1:[cH]:[*<br>]:[*]:[c]:1[*]	-0.406

FCFP_2	0	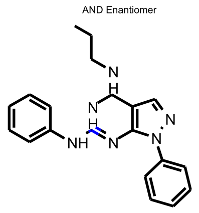 [*]C(=[*])[*]	-0.29

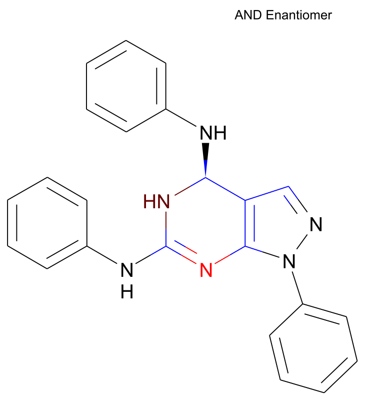

$C_{23}H_{20}N_6$

Molecular Weight: 380.4451

ALogP: 4.544

Rotatable Bonds: 5

Acceptors: 5

Donors: 3

Model Prediction

Prediction: 0.00144

Unit: g/kg_body_weight

Mahalanobis Distance: 18.1

Mahalanobis Distance p-value: 4.38e-017

Mahalanobis Distance: The Mahalanobis distance (MD) is a generalization of the Euclidean distance that accounts for correlations among the X properties. It is calculated as the distance to the center of the training data. The larger the MD, the less trustworthy the prediction.
Mahalanobis Distance p-value: The p-value gives the fraction of training data with an MD greater than or equal to the one for the given sample, assuming normally distributed data. The smaller the p-value, the less trustworthy the prediciton. For highly non-normal X properties (e.g., fingerprints), the MD p-value is wildly inaccurate.

Structural Similar Compounds

Name	OCHRATOXIN	SULFISOOXAZOLE	PHENYLBUTAZONE
Structure	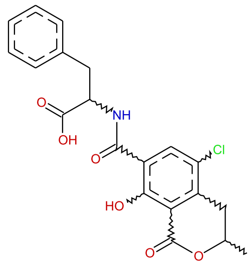	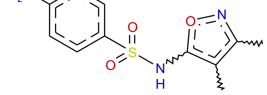	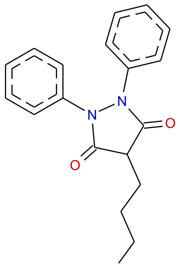
Actual Endpoint (-log C)	6.28396	2.82494	3.48909
Predicted Endpoint (-log C)	5.12358	3.0705	3.17333
Distance	1.065	1.108	1.338
Reference	NCI/NTP TR-358	NCI/NTP TR-138	NCI/NTP TR-367

Model Applicability

Unknown features are fingerprint features in the query molecule, but not found or appearing too infrequently in the training set.

1. Num_AromaticRings out of range. Value: 4. Training min, max, mean, SD: 0, 2, 0.5625, 0.693.
2. OPS PC6 out of range. Value: -2.6667. Training min, max, SD, explained variance: -2.4321, 2.9885, 1.256, 0.0488.
3. Unknown FCFP_2 feature: -1410049896: [*]n1:[*]:[*]:[c]([*]):[c]:1N=[*]
4. Unknown FCFP_2 feature: 179977000: [*][c]1:[*]:[*]:n:n:1[c](:[*]):[*]
5. Unknown FCFP_2 feature: 580453787: [*]C(=N[c](:[*]):[*])[*]
6. Unknown FCFP_2 feature: 1499521844: [*]NC(=N[*])N[*]
7. Unknown FCFP_2 feature: 2119857014: [*]NC(N[*])[c](:[*]):[*]
8. Unknown FCFP_2 feature: -1732563065: [*]:n(:[*])[c](:c:[*]):c:[*]

Feature Contribution

Top features for positive contribution			
**Fingerprint**	**Bit/Smiles**	**Feature Structure**	**Score**

FCFP_2	1	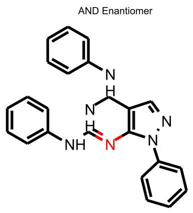 [*]N=[*]	0.511
FCFP_2	3	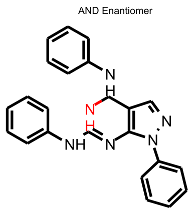 [*]N[*]	0.104

Top Features for negative contribution

Fingerprint	Bit/Smiles	Feature Structure	Score
FCFP_2	203677720	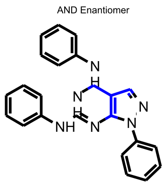 [*]C([*])[c]1:[cH]:[*]:[*]:[c]:1[*]	-0.406
FCFP_2	0	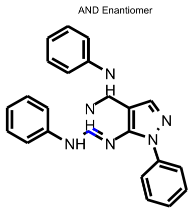 [*]C(=[*])[*]	-0.29

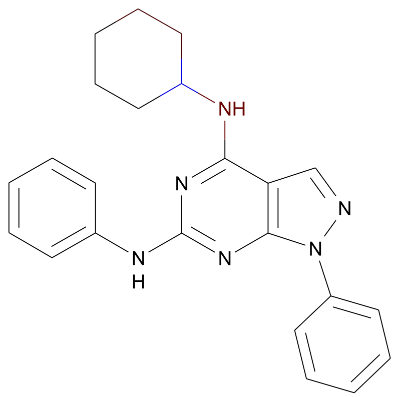

$C_{23}H_{24}N_6$

Molecular Weight: 384.47686

ALogP: 5.953

Rotatable Bonds: 5

Acceptors: 5

Donors: 2

Model Prediction

Prediction: 0.000921

Unit: g/kg_body_weight

Mahalanobis Distance: 17.7

Mahalanobis Distance p-value: 1.45e-016

Mahalanobis Distance: The Mahalanobis distance (MD) is a generalization of the Euclidean distance that accounts for correlations among the X properties. It is calculated as the distance to the center of the training data. The larger the MD, the less trustworthy the prediction.
Mahalanobis Distance p-value: The p-value gives the fraction of training data with an MD greater than or equal to the one for the given sample, assuming normally distributed data. The smaller the p-value, the less trustworthy the prediciton. For highly non-normal X properties (e.g., fingerprints), the MD p-value is wildly inaccurate.

Structural Similar Compounds

Name	OCHRATOXIN	SULFISOOXAZOLE	PHENYLBUTAZONE
Structure	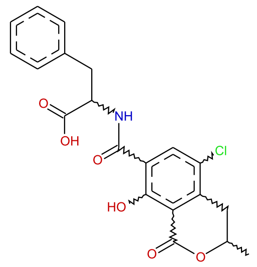	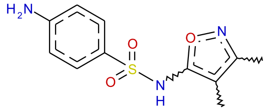	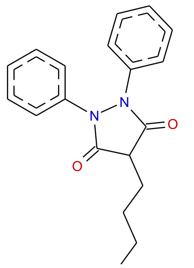
Actual Endpoint (-log C)	6.28396	2.82494	3.48909
Predicted Endpoint (-log C)	5.12358	3.0705	3.17333
Distance	1.170	1.198	1.239
Reference	NCI/NTP TR-358	NCI/NTP TR-138	NCI/NTP TR-367

Model Applicability

Unknown features are fingerprint features in the query molecule, but not found or appearing too infrequently in the training set.

1. Num_AromaticRings out of range. Value: 4. Training min, max, mean, SD: 0, 2, 0.5625, 0.693.
2. Unknown FCFP_2 feature: -1564473960: [*]n1:[*]:[*]:[c](:[*]):[c]:1:n:[*]
3. Unknown FCFP_2 feature: 179977000: [*][c]1:[*]:[*]:n:n:1[c](:[*]):[*]
4. Unknown FCFP_2 feature: -1151914249: [*]N[c](:n:[*]):n:[*]
5. Unknown FCFP_2 feature: -1732563065: [*]:n(:[*])[c](:c:[*]):c:[*]
6. Unknown FCFP_2 feature: 1293778554: [*]:[c](:[*])N[c](:[*]):[*]

Feature Contribution

Top features for positive contribution			
Fingerprint	Bit/Smiles	Feature Structure	Score
FCFP_2	3	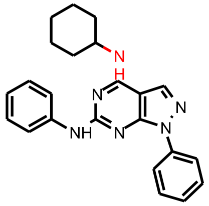 [*]N[*]	0.104

FCFP_2	-1272798659	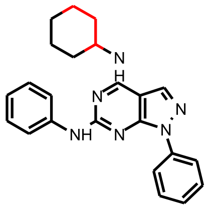 [*]CCC	0.0703
**Top Features for negative contribution**			
**Fingerprint**	**Bit/Smiles**	**Feature Structure**	**Score**
FCFP_2	0	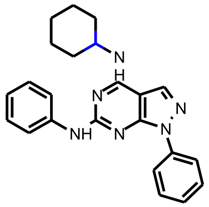 [*]C(=[*])[*]	-0.29

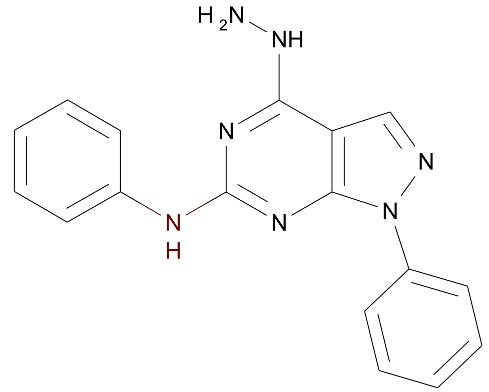

$C_{17}H_{15}N_7$
Molecular Weight: 317.3479
ALogP: 3.562
Rotatable Bonds: 4
Acceptors: 6
Donors: 3

Model Prediction

Prediction: 0.000633
Unit: g/kg_body_weight
Mahalanobis Distance: 15.2
Mahalanobis Distance p-value: 3.04e-013

Mahalanobis Distance: The Mahalanobis distance (MD) is a generalization of the Euclidean distance that accounts for correlations among the X properties. It is calculated as the distance to the center of the training data. The larger the MD, the less trustworthy the prediction.
Mahalanobis Distance p-value: The p-value gives the fraction of training data with an MD greater than or equal to the one for the given sample, assuming normally distributed data. The smaller the p-value, the less trustworthy the prediciton. For highly non-normal X properties (e.g., fingerprints), the MD p-value is wildly inaccurate.

Structural Similar Compounds

Name	OCHRATOXIN	SULFISOOXAZOLE	2-MERCAPTOBENZOTHIAZOLE
Structure	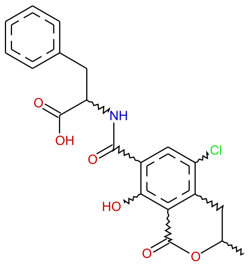	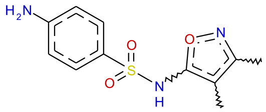	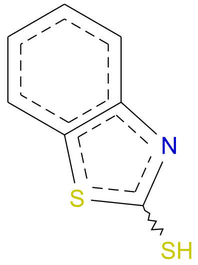
Actual Endpoint (-log C)	6.28396	2.82494	2.34829
Predicted Endpoint (-log C)	5.12358	3.0705	3.82125
Distance	1.028	1.047	1.305
Reference	NCI/NTP TR-358	NCI/NTP TR-138	NCI/NTP TR-332

Model Applicability

Unknown features are fingerprint features in the query molecule, but not found or appearing too infrequently in the training set.

1. Num_AromaticRings out of range. Value: 4. Training min, max, mean, SD: 0, 2, 0.5625, 0.693.
2. OPS PC9 out of range. Value: -2.8216. Training min, max, SD, explained variance: -2.7086, 2.9267, 1.019, 0.0321.
3. Unknown FCFP_2 feature: -1564473960: [*]n1:[*]:[*]:[c](:[*]):[c]:1:n:[*]
4. Unknown FCFP_2 feature: 179977000: [*][c]1:[*]:[*]:n:n:1[c](:[*]):[*]
5. Unknown FCFP_2 feature: -1151914249: [*]N[c](:n:[*]):n:[*]
6. Unknown FCFP_2 feature: -1732563065: [*]:n(:[*])[c](:c:[*]):c:[*]
7. Unknown FCFP_2 feature: 1293778554: [*]:[c](:[*])N[c](:[*]):[*]
8. Unknown FCFP_2 feature: 1294344583: [*]:[c](:[*])NN
9. Unknown FCFP_2 feature: 1070150408: [*]NN

Feature Contribution

Top features for positive contribution			
Fingerprint	Bit/Smiles	Feature Structure	Score

FCFP_2	3	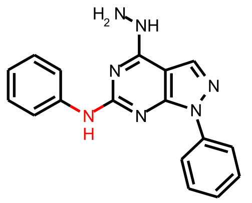<br>[*]N[*]	0.104

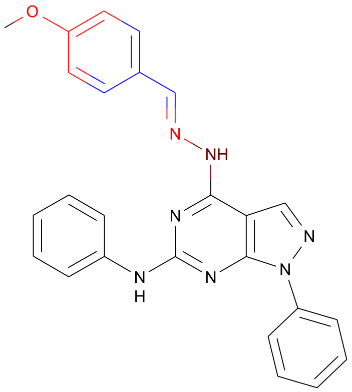

$C_{25}H_{21}N_7O$

Molecular Weight: 435.48054

ALogP: 5.889

Rotatable Bonds: 7

Acceptors: 7

Donors: 2

Model Prediction

Prediction: 2.58e-005

Unit: g/kg_body_weight

Mahalanobis Distance: 17.2

Mahalanobis Distance p-value: 5.78e-016

Mahalanobis Distance: The Mahalanobis distance (MD) is a generalization of the Euclidean distance that accounts for correlations among the X properties. It is calculated as the distance to the center of the training data. The larger the MD, the less trustworthy the prediction.
Mahalanobis Distance p-value: The p-value gives the fraction of training data with an MD greater than or equal to the one for the given sample, assuming normally distributed data. The smaller the p-value, the less trustworthy the prediciton. For highly non-normal X properties (e.g., fingerprints), the MD p-value is wildly inaccurate.

Structural Similar Compounds

Name	OCHRATOXIN	SULFISOOXAZOLE	8-METHOXYPSORALEN
Structure	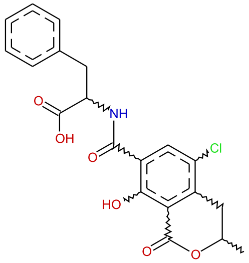	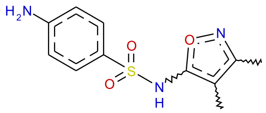	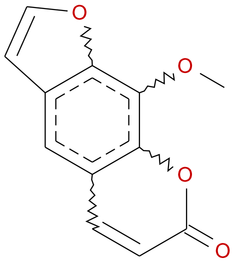
Actual Endpoint (-log C)	6.28396	2.82494	3.45978
Predicted Endpoint (-log C)	5.12358	3.0705	4.14745
Distance	1.331	1.441	1.539
Reference	NCI/NTP TR-358	NCI/NTP TR-138	NCI/NTP TR-359

Model Applicability

Unknown features are fingerprint features in the query molecule, but not found or appearing too infrequently in the training set.

1. Molecular_Weight out of range. Value: 435.48. Training min, max, mean, SD: 68.074, 434.63, 171.13, 85.06.
2. Num_H_Acceptors out of range. Value: 7. Training min, max, mean, SD: 0, 6, 1.6146, 1.644.
3. Num_AromaticRings out of range. Value: 5. Training min, max, mean, SD: 0, 2, 0.5625, 0.693.
4. OPS PC6 out of range. Value: -4.1283. Training min, max, SD, explained variance: -2.4321, 2.9885, 1.256, 0.0488.
5. Unknown FCFP_2 feature: -1564473960: [*]n1:[*]:[*]:[c](:[*]):[c]:1:n:[*]
6. Unknown FCFP_2 feature: 179977000: [*][c]1:[*]:[*]:n:n:1[c](:[*]):[*]
7. Unknown FCFP_2 feature: -1151914249: [*]N[c](:n:[*]):n:[*]
8. Unknown FCFP_2 feature: 1294285001: [*]=NN[c](:[*]):[*]
9. Unknown FCFP_2 feature: -1732563065: [*]:n(:[*])[c](:c:[*]):c:[*]
10. Unknown FCFP_2 feature: 581019816: [*]N\N=C\[*]
11. Unknown FCFP_2 feature: 1293778554: [*]:[c](:[*])N[c](:[*]):[*]

Feature Contribution

Top features for positive contribution

Fingerprint	Bit/Smiles	Feature Structure	Score

FCFP_2	332760439	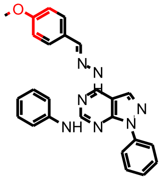 [*]O[c](:[cH]:[*]):[cH]:[*]	0.672
FCFP_2	1	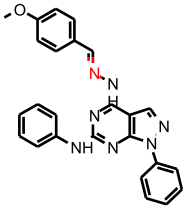 [*]N=[*]	0.511
FCFP_2	3	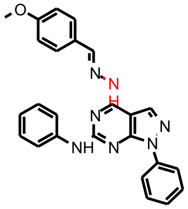 [*]N[*]	0.104
**Top Features for negative contribution**			
**Fingerprint**	**Bit/Smiles**	**Feature Structure**	**Score**
FCFP_2	203677720	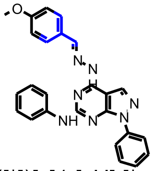 [*]C([*])[c]1:[cH]:[*]:[*]:[c]:1[*]	-0.406
FCFP_2	0	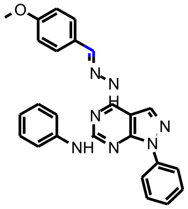 [*]C(=[*])[*]	-0.29

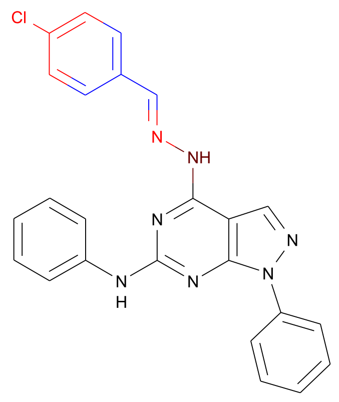

$C_{24}H_{18}ClN_7$
Molecular Weight: 439.89962
ALogP: 6.57
Rotatable Bonds: 6
Acceptors: 6
Donors: 2

Model Prediction

Prediction: 2.65e-005
Unit: g/kg_body_weight
Mahalanobis Distance: 18.5
Mahalanobis Distance p-value: 1.08e-017

Mahalanobis Distance: The Mahalanobis distance (MD) is a generalization of the Euclidean distance that accounts for correlations among the X properties. It is calculated as the distance to the center of the training data. The larger the MD, the less trustworthy the prediction.
Mahalanobis Distance p-value: The p-value gives the fraction of training data with an MD greater than or equal to the one for the given sample, assuming normally distributed data. The smaller the p-value, the less trustworthy the prediciton. For highly non-normal X properties (e.g., fingerprints), the MD p-value is wildly inaccurate.

Structural Similar Compounds

Name	OCHRATOXIN	SULFISOOXAZOLE	CHLORPHENIRAMINE MALEATE
Structure	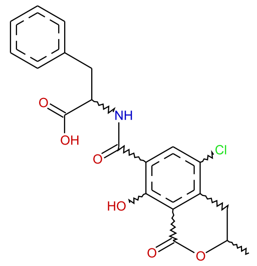	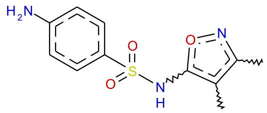	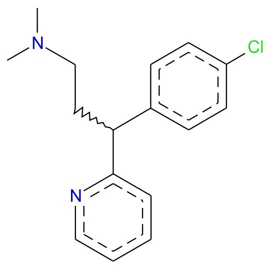
Actual Endpoint (-log C)	6.28396	2.82494	3.96188
Predicted Endpoint (-log C)	5.12358	3.0705	3.83117
Distance	1.313	1.452	1.516
Reference	NCI/NTP TR-358	NCI/NTP TR-138	NCI/NTP TR-317

Model Applicability

Unknown features are fingerprint features in the query molecule, but not found or appearing too infrequently in the training set.

1. Molecular_Weight out of range. Value: 439.9. Training min, max, mean, SD: 68.074, 434.63, 171.13, 85.06.
2. Num_AromaticRings out of range. Value: 5. Training min, max, mean, SD: 0, 2, 0.5625, 0.693.
3. OPS PC7 out of range. Value: -3.4174. Training min, max, SD, explained variance: -2.8003, 2.9332, 1.16, 0.0416.
4. Unknown FCFP_2 feature: -1564473960: [*]n1:[*]:[*]:[c](:[*]):[c]:1:n:[*]
5. Unknown FCFP_2 feature: 179977000: [*][c]1:[*]:[*]:n:n:1[c](:[*]):[*]
6. Unknown FCFP_2 feature: -1151914249: [*]N[c](:n:[*]):n:[*]
7. Unknown FCFP_2 feature: 1294285001: [*]=NN[c](:[*]):[*]
8. Unknown FCFP_2 feature: -1732563065: [*]:n(:[*])[c](:c:[*]):c:[*]
9. Unknown FCFP_2 feature: 581019816: [*]N\N=C\[*]
10. Unknown FCFP_2 feature: 1293778554: [*]:[c](:[*])N[c](:[*]):[*]

Feature Contribution

Top features for positive contribution			
Fingerprint	Bit/Smiles	Feature Structure	Score

FCFP_2	32	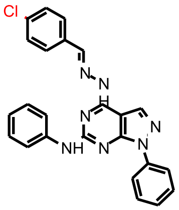 [*]Cl	0.526
FCFP_2	1	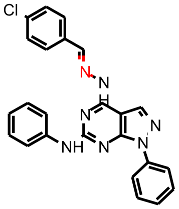 [*]N=[*]	0.511
FCFP_2	367998008	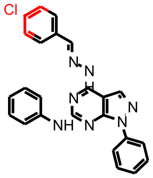 [*]:[cH]:[c](Cl):[cH]:[*]	0.413
**Top Features for negative contribution**			
**Fingerprint**	**Bit/Smiles**	**Feature Structure**	**Score**
FCFP_2	203677720	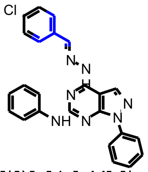 [*]C([*])[c]1:[cH]:[*]:[*]:[c]:1[*]	-0.406
FCFP_2	0	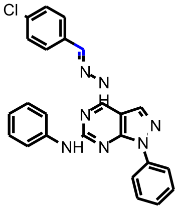 [*]C(=[*])[*]	-0.29

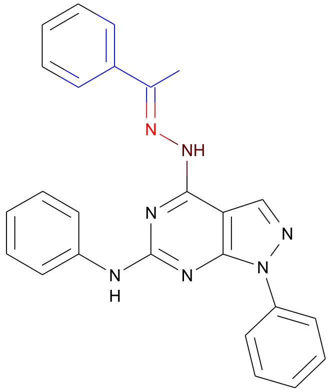

$C_{25}H_{21}N_7$
Molecular Weight: 419.48114
ALogP: 5.886
Rotatable Bonds: 6
Acceptors: 6
Donors: 2

Model Prediction

Prediction: 0.00104
Unit: g/kg_body_weight
Mahalanobis Distance: 20.2
Mahalanobis Distance p-value: 8.96e-020

Mahalanobis Distance: The Mahalanobis distance (MD) is a generalization of the Euclidean distance that accounts for correlations among the X properties. It is calculated as the distance to the center of the training data. The larger the MD, the less trustworthy the prediction.
Mahalanobis Distance p-value: The p-value gives the fraction of training data with an MD greater than or equal to the one for the given sample, assuming normally distributed data. The smaller the p-value, the less trustworthy the prediciton. For highly non-normal X properties (e.g., fingerprints), the MD p-value is wildly inaccurate.

Structural Similar Compounds

Name	OCHRATOXIN	SULFISOOXAZOLE	PHENYLBUTAZONE
Structure	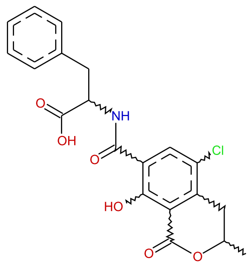	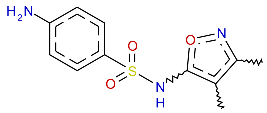	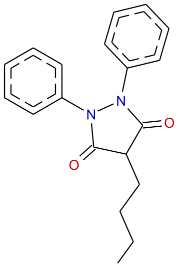
Actual Endpoint (-log C)	6.28396	2.82494	3.48909
Predicted Endpoint (-log C)	5.12358	3.0705	3.17333
Distance	1.311	1.388	1.478
Reference	NCI/NTP TR-358	NCI/NTP TR-138	NCI/NTP TR-367

Model Applicability

Unknown features are fingerprint features in the query molecule, but not found or appearing too infrequently in the training set.

1. Num_AromaticRings out of range. Value: 5. Training min, max, mean, SD: 0, 2, 0.5625, 0.693.
2. OPS PC6 out of range. Value: -3.2033. Training min, max, SD, explained variance: -2.4321, 2.9885, 1.256, 0.0488.
3. Unknown FCFP_2 feature: -1564473960: [*]n1:[*]:[*]:[c](:[*]):[c]:1:n:[*]
4. Unknown FCFP_2 feature: 179977000: [*][c]1:[*]:[*]:n:n:1[c](:[*]):[*]
5. Unknown FCFP_2 feature: -1151914249: [*]N[c](:n:[*]):n:[*]
6. Unknown FCFP_2 feature: 1294285001: [*]=NN[c](:[*]):[*]
7. Unknown FCFP_2 feature: -1732563065: [*]:n(:[*])[c](:c:[*]):c:[*]
8. Unknown FCFP_2 feature: 581019816: [*]N\N=C\[*]
9. Unknown FCFP_2 feature: -1549192822: [*]\N=C(/C)\[c](:[*]):[*]
10. Unknown FCFP_2 feature: 1293778554: [*]:[c](:[*])N[c](:[*]):[*]

Feature Contribution

Top features for positive contribution

Fingerprint	Bit/Smiles	Feature Structure	Score

FCFP_2	1	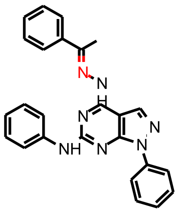 [*]N=[*]	0.511
FCFP_2	3	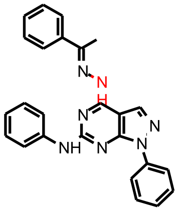 [*]N[*]	0.104
**Top Features for negative contribution**			
**Fingerprint**	**Bit/Smiles**	**Feature Structure**	**Score**
FCFP_2	136597326	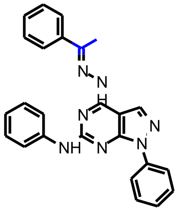 [*]CC	-0.489
FCFP_2	203677720	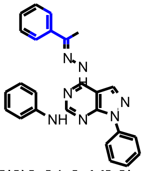 [*]C([*])[c]1:[cH]:[*<br>]:[*]:[c]:1[*]	-0.406
FCFP_2	0	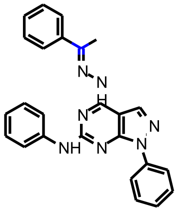 [*]C(=[*])[*]	-0.29

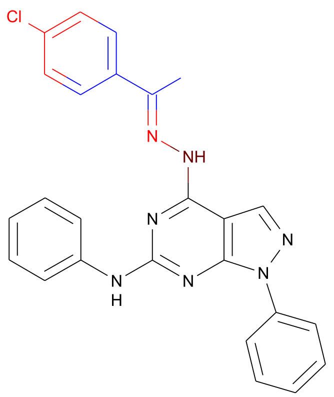

$C_{25}H_{20}ClN_7$
Molecular Weight: 453.9262
ALogP: 6.55
Rotatable Bonds: 6
Acceptors: 6
Donors: 2

Model Prediction

Prediction: 8.34e-005
Unit: g/kg_body_weight
Mahalanobis Distance: 18.1
Mahalanobis Distance p-value: 3.65e-017

Mahalanobis Distance: The Mahalanobis distance (MD) is a generalization of the Euclidean distance that accounts for correlations among the X properties. It is calculated as the distance to the center of the training data. The larger the MD, the less trustworthy the prediction.
Mahalanobis Distance p-value: The p-value gives the fraction of training data with an MD greater than or equal to the one for the given sample, assuming normally distributed data. The smaller the p-value, the less trustworthy the prediciton. For highly non-normal X properties (e.g., fingerprints), the MD p-value is wildly inaccurate.

Structural Similar Compounds

Name	OCHRATOXIN	SULFISOOXAZOLE	PHENYLBUTAZONE
Structure	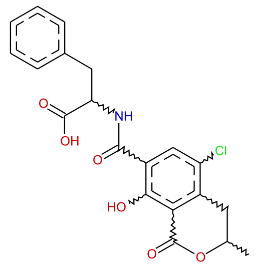	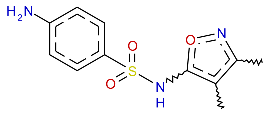	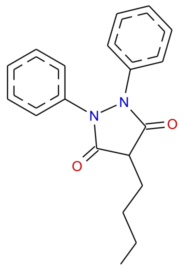
Actual Endpoint (-log C)	6.28396	2.82494	3.48909
Predicted Endpoint (-log C)	5.12358	3.0705	3.17333
Distance	1.302	1.466	1.521
Reference	NCI/NTP TR-358	NCI/NTP TR-138	NCI/NTP TR-367

Model Applicability

Unknown features are fingerprint features in the query molecule, but not found or appearing too infrequently in the training set.

1. Molecular_Weight out of range. Value: 453.93. Training min, max, mean, SD: 68.074, 434.63, 171.13, 85.06.
2. Num_AromaticRings out of range. Value: 5. Training min, max, mean, SD: 0, 2, 0.5625, 0.693.
3. OPS PC7 out of range. Value: -3.2841. Training min, max, SD, explained variance: -2.8003, 2.9332, 1.16, 0.0416.
4. Unknown FCFP_2 feature: -1564473960: [*]n1:[*]:[*]:[c](:[*]):[c]:1:n:[*]
5. Unknown FCFP_2 feature: 179977000: [*][c]1:[*]:[*]:n:n:1[c](:[*]):[*]
6. Unknown FCFP_2 feature: -1151914249: [*]N[c](:n:[*]):n:[*]
7. Unknown FCFP_2 feature: 1294285001: [*]=NN[c](:[*]):[*]
8. Unknown FCFP_2 feature: -1732563065: [*]:n(:[*])[c](:c:[*]):c:[*]
9. Unknown FCFP_2 feature: 581019816: [*]N\N=C\[*]
10. Unknown FCFP_2 feature: -1549192822: [*]\N=C(/C)\[c](:[*]):[*]
11. Unknown FCFP_2 feature: 1293778554: [*]:[c](:[*])N[c](:[*]):[*]

Feature Contribution

Top features for positive contribution

Fingerprint	Bit/Smiles	Feature Structure	Score

FCFP_2	32	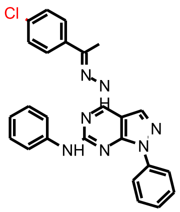<br>[*]Cl	0.526
FCFP_2	1	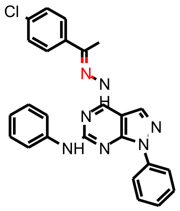<br>[*]N=[*]	0.511
FCFP_2	367998008	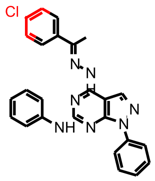<br>[*]:[cH]:[c](Cl):[cH]:[*]	0.413

Top Features for negative contribution

Fingerprint	Bit/Smiles	Feature Structure	Score
FCFP_2	136597326	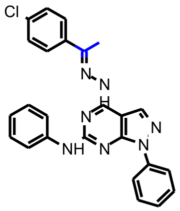<br>[*]CC	-0.489
FCFP_2	203677720	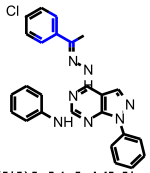<br>[*]C([*])[c]1:[cH]:[*]:[*]:[c]:1[*]	-0.406

FCFP_2	0	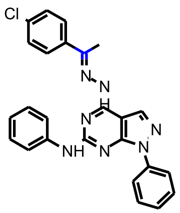 [*]C(=[*])[*]	-0.29

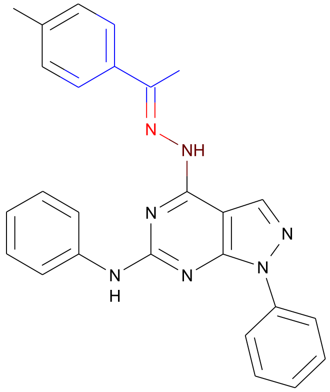

$C_{26}H_{23}N_7$
Molecular Weight: 433.50772
ALogP: 6.372
Rotatable Bonds: 6
Acceptors: 6
Donors: 2

Model Prediction

Prediction: 0.000791
Unit: g/kg_body_weight
Mahalanobis Distance: 20.3
Mahalanobis Distance p-value: 6.49e-020

Mahalanobis Distance: The Mahalanobis distance (MD) is a generalization of the Euclidean distance that accounts for correlations among the X properties. It is calculated as the distance to the center of the training data. The larger the MD, the less trustworthy the prediction.
Mahalanobis Distance p-value: The p-value gives the fraction of training data with an MD greater than or equal to the one for the given sample, assuming normally distributed data. The smaller the p-value, the less trustworthy the prediciton. For highly non-normal X properties (e.g., fingerprints), the MD p-value is wildly inaccurate.

Structural Similar Compounds

Name	OCHRATOXIN	SULFISOOXAZOLE	PHENYLBUTAZONE
Structure	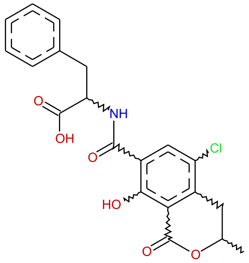	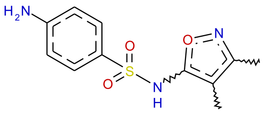	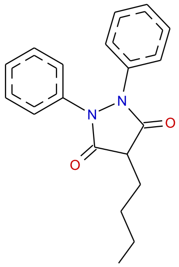
Actual Endpoint (-log C)	6.28396	2.82494	3.48909
Predicted Endpoint (-log C)	5.12358	3.0705	3.17333
Distance	1.333	1.407	1.500
Reference	NCI/NTP TR-358	NCI/NTP TR-138	NCI/NTP TR-367

Model Applicability

Unknown features are fingerprint features in the query molecule, but not found or appearing too infrequently in the training set.

1. Num_AromaticRings out of range. Value: 5. Training min, max, mean, SD: 0, 2, 0.5625, 0.693.
2. OPS PC6 out of range. Value: -3.2672. Training min, max, SD, explained variance: -2.4321, 2.9885, 1.256, 0.0488.
3. Unknown FCFP_2 feature: -1564473960: [*]n1:[*]:[*]:[c](:[*]):[c]:1:n:[*]
4. Unknown FCFP_2 feature: 179977000: [*][c]1:[*]:[*]:n:n:1[c](:[*]):[*]
5. Unknown FCFP_2 feature: -1151914249: [*]N[c](:n:[*]):n:[*]
6. Unknown FCFP_2 feature: 1294285001: [*]=NN[c](:[*]):[*]
7. Unknown FCFP_2 feature: -1732563065: [*]:n(:[*])[c](:c:[*]):c:[*]
8. Unknown FCFP_2 feature: 581019816: [*]N\N=C\[*]
9. Unknown FCFP_2 feature: -1549192822: [*]\N=C(/C)\[c](:[*]):[*]
10. Unknown FCFP_2 feature: 1293778554: [*]:[c](:[*])N[c](:[*]):[*]

Feature Contribution

Top features for positive contribution			
Fingerprint	Bit/Smiles	Feature Structure	Score

FCFP_2	1	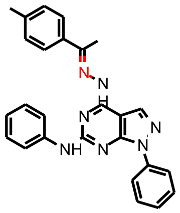 [*]N=[*]	0.511
FCFP_2	3	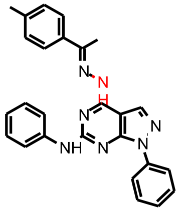 [*]N[*]	0.104

Top Features for negative contribution

Fingerprint	Bit/Smiles	Feature Structure	Score
FCFP_2	136597326	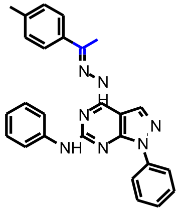 [*]CC	-0.489
FCFP_2	203677720	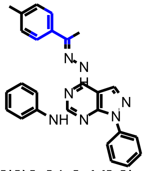 [*]C([*])[c]1:[cH]:[*]:[*]:[c]:1[*]	-0.406
FCFP_2	0	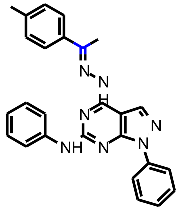 [*]C(=[*])[*]	-0.29

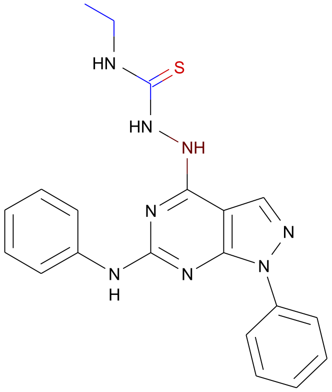

$C_{20}H_{20}N_8S$
Molecular Weight: 404.4914
ALogP: 4.99
Rotatable Bonds: 8
Acceptors: 6
Donors: 4

Structural Similar Compounds

Name	OCHRATOXIN	SULFISOOXAZOLE	PENICILLIN VK
Structure	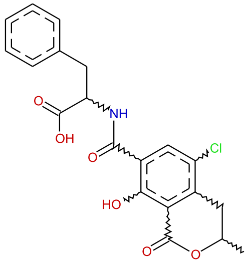	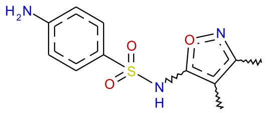	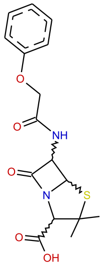
Actual Endpoint (-log C)	6.28396	2.82494	2.54455
Predicted Endpoint (-log C)	5.12358	3.0705	3.9702
Distance	1.017	1.273	1.406
Reference	NCI/NTP TR-358	NCI/NTP TR-138	NCI/NTP TR-336

Model Prediction

Prediction: 0.00851
Unit: g/kg_body_weight
Mahalanobis Distance: 16.6
Mahalanobis Distance p-value: 3.93e-015

Mahalanobis Distance: The Mahalanobis distance (MD) is a generalization of the Euclidean distance that accounts for correlations among the X properties. It is calculated as the distance to the center of the training data. The larger the MD, the less trustworthy the prediction.
Mahalanobis Distance p-value: The p-value gives the fraction of training data with an MD greater than or equal to the one for the given sample, assuming normally distributed data. The smaller the p-value, the less trustworthy the prediciton. For highly non-normal X properties (e.g., fingerprints), the MD p-value is wildly inaccurate.

Model Applicability

Unknown features are fingerprint features in the query molecule, but not found or appearing too infrequently in the training set.

1. Num_H_Donors out of range. Value: 4. Training min, max, mean, SD: 0, 3, 0.4375, 0.8311.
2. Num_AromaticRings out of range. Value: 4. Training min, max, mean, SD: 0, 2, 0.5625, 0.693.
3. OPS PC9 out of range. Value: -2.7312. Training min, max, SD, explained variance: -2.7086, 2.9267, 1.019, 0.0321.
4. Unknown FCFP_2 feature: -1564473960: [*]n1:[*]:[*]:[c](:[*]):[c]:1:n:[*]
5. Unknown FCFP_2 feature: 179977000: [*][c]1:[*]:[*]:n:n:1[c](:[*]):[*]
6. Unknown FCFP_2 feature: -1151914249: [*]N[c](:n:[*]):n:[*]
7. Unknown FCFP_2 feature: 1294344583: [*]:[c](:[*])NN
8. Unknown FCFP_2 feature: -1732563065: [*]:n(:[*])[c](:c:[*]):c:[*]
9. Unknown FCFP_2 feature: -885461129: [*]NNC(=[*])[*]
10. Unknown FCFP_2 feature: 1499521844: [*]NC(=N[*])N[*]
11. Unknown FCFP_2 feature: 1293778554: [*]:[c](:[*])N[c](:[*]):[*]

Feature Contribution

Top features for positive contribution			
**Fingerprint**	**Bit/Smiles**	**Feature Structure**	**Score**

FCFP_2	1	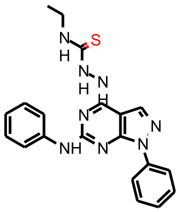 [*]N=[*]	0.511
FCFP_2	3	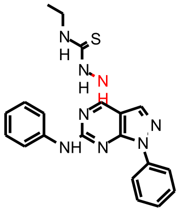 [*]N[*]	0.104
**Top Features for negative contribution**			
**Fingerprint**	**Bit/Smiles**	**Feature Structure**	**Score**
FCFP_2	136597326	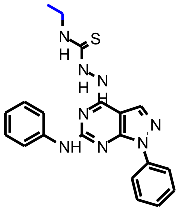 [*]CC	-0.489
FCFP_2	1872154524	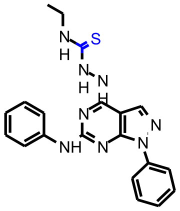 [*]C(=S)[*]	-0.307
FCFP_2	0	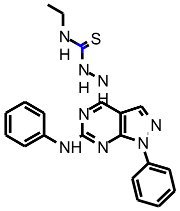 [*]C(=[*])[*]	-0.29

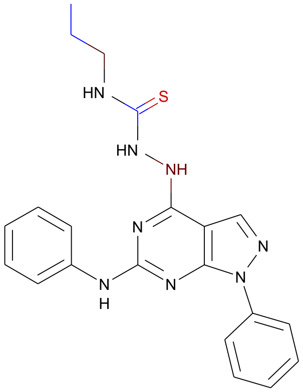

$C_{21}H_{22}N_8S$
Molecular Weight: 418.51798
ALogP: 5.514
Rotatable Bonds: 9
Acceptors: 6
Donors: 4

Model Prediction

Prediction: 0.00796
Unit: g/kg_body_weight
Mahalanobis Distance: 17.1
Mahalanobis Distance p-value: 8.57e-016

Mahalanobis Distance: The Mahalanobis distance (MD) is a generalization of the Euclidean distance that accounts for correlations among the X properties. It is calculated as the distance to the center of the training data. The larger the MD, the less trustworthy the prediction.
Mahalanobis Distance p-value: The p-value gives the fraction of training data with an MD greater than or equal to the one for the given sample, assuming normally distributed data. The smaller the p-value, the less trustworthy the prediciton. For highly non-normal X properties (e.g., fingerprints), the MD p-value is wildly inaccurate.

Structural Similar Compounds

Name	OCHRATOXIN	SULFISOOXAZOLE	PENICILLIN VK
Structure	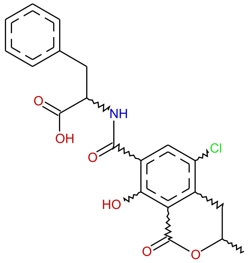	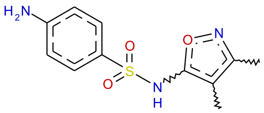	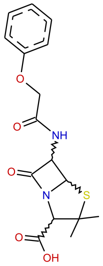
Actual Endpoint (-log C)	6.28396	2.82494	2.54455
Predicted Endpoint (-log C)	5.12358	3.0705	3.9702
Distance	1.061	1.338	1.452
Reference	NCI/NTP TR-358	NCI/NTP TR-138	NCI/NTP TR-336

Model Applicability

Unknown features are fingerprint features in the query molecule, but not found or appearing too infrequently in the training set.

1. Num_H_Donors out of range. Value: 4. Training min, max, mean, SD: 0, 3, 0.4375, 0.8311.
2. Num_AromaticRings out of range. Value: 4. Training min, max, mean, SD: 0, 2, 0.5625, 0.693.
3. OPS PC9 out of range. Value: -3.081. Training min, max, SD, explained variance: -2.7086, 2.9267, 1.019, 0.0321.
4. Unknown FCFP_2 feature: -1564473960: [*]n1:[*]:[*]:[c](:[*]):[c]:1:n:[*]
5. Unknown FCFP_2 feature: 179977000: [*][c]1:[*]:[*]:n:n:1[c](:[*]):[*]
6. Unknown FCFP_2 feature: -1151914249: [*]N[c](:n:[*]):n:[*]
7. Unknown FCFP_2 feature: 1294344583: [*]:[c](:[*])NN
8. Unknown FCFP_2 feature: -1732563065: [*]:n(:[*])[c](:c:[*]):c:[*]
9. Unknown FCFP_2 feature: -885461129: [*]NNC(=[*])[*]
10. Unknown FCFP_2 feature: 1499521844: [*]NC(=N[*])N[*]
11. Unknown FCFP_2 feature: 1293778554: [*]:[c](:[*])N[c](:[*]):[*]

Feature Contribution

Top features for positive contribution

Fingerprint	Bit/Smiles	Feature Structure	Score

FCFP_2	1	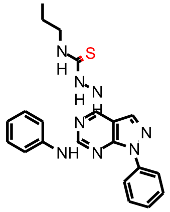 [*]N=[*]	0.511
FCFP_2	3	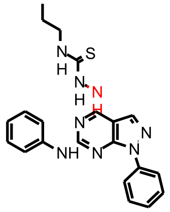 [*]N[*]	0.104
FCFP_2	-1272798659	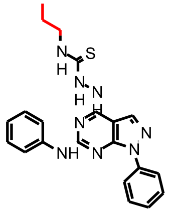 [*]CCC	0.0703
**Top Features for negative contribution**			
**Fingerprint**	**Bit/Smiles**	**Feature Structure**	**Score**
FCFP_2	136597326	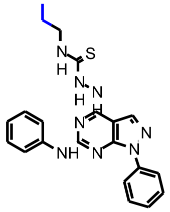 [*]CC	-0.489
FCFP_2	1872154524	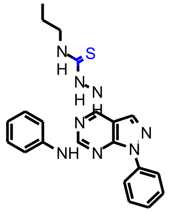 [*]C(=S)[*]	-0.307

FCFP_2	0	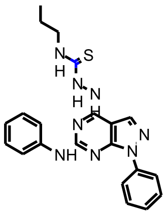 [*]C(=[*])[*]	-0.29

Erlotinib

TOPKAT_Rat_Maximum_Tolerated_Dose_Gavage

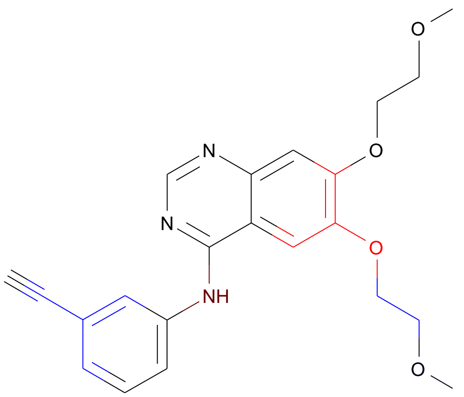

$C_{22}H_{23}N_3O_4$

Molecular Weight: 393.43572

ALogP: 4.309

Rotatable Bonds: 10

Acceptors: 7

Donors: 1

Model Prediction

Prediction: 0.000344

Unit: g/kg_body_weight

Mahalanobis Distance: 11.7

Mahalanobis Distance p-value: 2.74e-008

Mahalanobis Distance: The Mahalanobis distance (MD) is a generalization of the Euclidean distance that accounts for correlations among the X properties. It is calculated as the distance to the center of the training data. The larger the MD, the less trustworthy the prediction.

Mahalanobis Distance p-value: The p-value gives the fraction of training data with an MD greater than or equal to the one for the given sample, assuming normally distributed data. The smaller the p-value, the less trustworthy the prediciton. For highly non-normal X properties (e.g., fingerprints), the MD p-value is wildly inaccurate.

Structural Similar Compounds

Name	DIALLYL PHTHALATE	OCHRATOXIN	PROBENECID
Structure	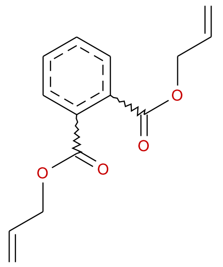	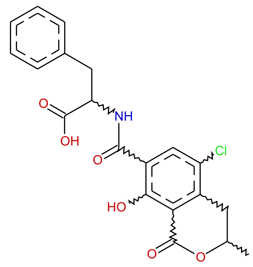	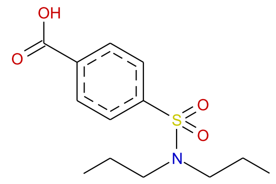
Actual Endpoint (-log C)	3.3914	6.28396	2.85333
Predicted Endpoint (-log C)	3.50093	5.12358	2.4258
Distance	1.113	1.136	1.152
Reference	NCI/NTP TR-284	NCI/NTP TR-358	NCI/NTP TR-395

Model Applicability

Unknown features are fingerprint features in the query molecule, but not found or appearing too infrequently in the training set.

1. Num_H_Acceptors out of range. Value: 7. Training min, max, mean, SD: 0, 6, 1.6146, 1.644.
2. Num_AromaticRings out of range. Value: 3. Training min, max, mean, SD: 0, 2, 0.5625, 0.693.
3. OPS PC6 out of range. Value: -3.0997. Training min, max, SD, explained variance: -2.4321, 2.9885, 1.256, 0.0488.
4. Unknown FCFP_2 feature: -124685461: [*]:n:c:n:[*]
5. Unknown FCFP_2 feature: 1293778554: [*]:[c](:[*])N[c](:[*]):[*]
6. Unknown FCFP_2 feature: 902193919: [*]:[c](:[*])C#C
7. Unknown FCFP_2 feature: 131784192: [*]C#C

Feature Contribution

Top features for positive contribution			
Fingerprint	Bit/Smiles	Feature Structure	Score

FCFP_2	332760439	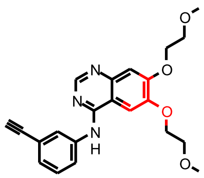 [*]O[c](:[cH]:[*]):[c H]:[*]	0.672
FCFP_2	1	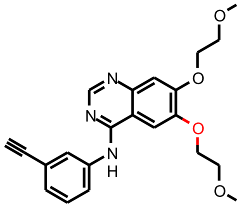 [*]N=[*]	0.511
FCFP_2	3	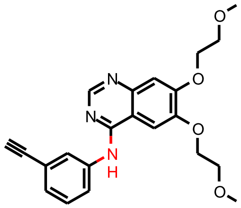 [*]N[*]	0.104

Top Features for negative contribution

Fingerprint	Bit/Smiles	Feature Structure	Score
FCFP_2	203677720	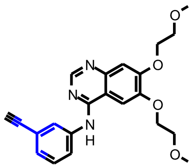 [*]C([*])[c]1:[cH]:[*]:[*]:[c]:1[*]	-0.406
FCFP_2	0	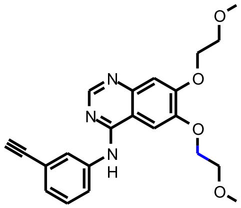 [*]C(=[*])[*]	-0.29

FCFP_2	-1272768868	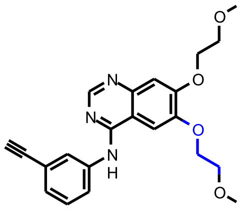<br>[*]CCO[*]	-0.271
